# A Modular Synthetic Strategy toward Fast-Growing Poly(amide-carbosilane) Dendrimers Based on Click Chemistry and Organic Solvent Nanofiltration

**DOI:** 10.1021/acspolymersau.5c00171

**Published:** 2026-01-16

**Authors:** Antonín Edr, Martin Konhefr, Alena Krupková, Lucie Červenková Št́astná, Jana Bernášková, Olga Kočková, Věra Vlčková, Zuzana Walterová, Lívia Kanizsová, Jan Lang, Jakub Žváček, Marek Malý, Tomáš Strašák

**Affiliations:** † 86876 The Czech Academy of Sciences, Institute of Chemical Process Fundamentals, Rozvojová 1/135, Prague 165 00, Czech Republic; ‡ The Czech Academy of Sciences, Institute of Macromolecular Chemistry, Heyrovského nám. 2, Prague 6 162 00, Czech Republic; § Department of Physics, University of Jan Evangelista Purkyně in Ústí nad Labem, Pasteurova 15, Ústí nad Labem 400 96, Czech Republic; ∥ Department of Organic Chemistry, University of Chemistry and Technology, Technická 5, Prague 6 166 28, Czech Republic; ⊥ Department of Low Temperature Physics, Faculty of Mathematics and Physics, 138735 Charles University, V Holešovičkách 747/2, Prague 180 00, Czech Republic

**Keywords:** polycationic dendrimers, organic solvent nanofiltration, accelerated growth, modular synthesis, molecular dynamics simulation

## Abstract

Dendrimers, constituting a prominent class of monodisperse and multivalent macromolecular compounds with outstanding properties, are characterized by regular and highly branched three-dimensional architectures and well-defined chemical structures. Within each structural type, the skeletal diversity is typically limited to the range of generations. Here, we introduce a novel modular synthetic strategy enabling an increase in the diversity of the dendrimer interior while maintaining its chemical nature. Resulting poly­(amide-carbosilane) (PAMCAS) dendrimers can be fine-tuned within one generation in terms of size, number, and density of end groups, as well as interior free volume. Using a tetravalent core and two building blocks—dendritic wedges with branching degrees 3 and 6—we demonstrate the potency of this strategy by producing a family of dendrimers through a controlled iterative process that combines highly chemoselective amidic coupling and thiol–ene click reaction (TEC). Within three generations, we prepared 14 structural analogs of PAMCAS dendrimers, systematically varying the order of building blocks and thus their structural profile. The solution properties of the obtained materials were studied by DLS, A4F, diffusion NMR, and molecular modeling. When using exclusively the AB_6_ module, the dendritic growth is accelerated and allows straightforward access to structures with extremely high valency in a given generation. As the modular synthetic strategy poses a considerable purification challenge, we implemented organic solvent nanofiltration (OSN) as the main separation tool. Herein, we demonstrate proof-of-principle experiments to evaluate the scope and limits of the use of OSN as an effective separation method in synthetic macromolecular chemistry.

## Graphical abstract

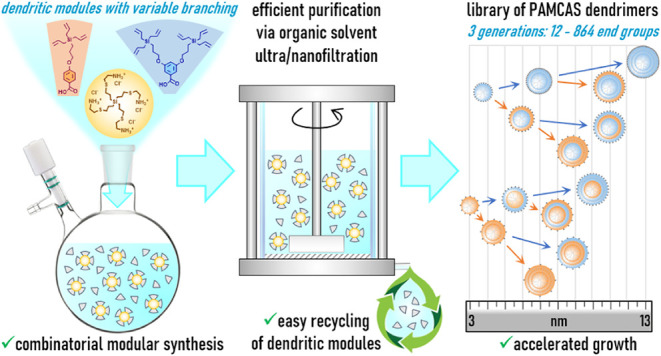

## 1 Introduction

The demand for biomimetic and bioinspired materials has drawn attention to more precisely defined macromolecular structures with low dispersity.^1^ The most widely used synthetic macromolecules, polymers, are often prepared by conventional free-radical polymerization (RP) as it is relatively easy to implement, with many suitable monomers available.^2^ The main limitation of RP is poor control over molecular weight, dispersity, end functionality, chain architecture, and composition. Better-defined polymers with more controlled structural parameters are accessible through ionic living polymerization, which, however, requires stringent conditions and is limited to a relatively small number of monomers. With an appropriate choice of reagents and reaction conditions, radical polymerization may also take on much of the character of a living polymerization (so-called reversible-deactivation radical polymerization).^3,4^ Nevertheless, living polymerizations remain statistical in nature. Innovative approaches providing sequential control of monomers during polymerization have enabled to define the position of the monomer in the chain to a certain extent; however, even these sequence-controlled polymers (SCPs), including gradient, periodic, and altering polymers, are not completely uniform.^5–7^

The progress in organic chemistry approaches opened up a possibility to switch from a statistically controlled polymerization to a sequence of exactly defined high molecular weight substances with a uniform monodisperse structure.^8,9^ The solid-phase peptide synthesis (SPPS) approach enables the stepwise attachment of individual monomer units to a solid support, yielding peptide sequences with high precision. Xu et al. emphasize the efficacy of SPPS, highlighting that similar techniques can be employed for the synthesis of diverse sequence-defined macromolecules.^10^ The progress in chemoselective and orthogonal reactions and the concept of “click” chemistry stimulated the boost of methodologies based on traditional step-by-step organic reactions and unlocked access to cutting-edge, rationally designed materials.^11–14^ These approaches have been applied to prepare sequence-defined polymers, uniform SCPs characterized as monodisperse macromolecules with perfectly defined monomer order.^15–17^

Dendrimers (DDMs), the most prominent example of *in principle* monodisperse macromolecules, are receiving increased attention due to the vast possibilities of manipulating their structure and size in a defined way.^18–20^ Since Tomalia^21^ and Vögtle^22^ introduced the first types of DDMs, many other examples and synthetic strategies have been developed.^18^ As the traditional methods of DDM preparation are considerably time-consuming when aiming at higher generations (G_*n*_), several improvements have been proposed to simplify the process and shorten the synthetic time. Methods commonly referred to as “accelerated approaches” are primarily based on “click” chemistry.^23^ In their effort to accelerate the synthesis, Malkoch and coworkers optimized fluoride-promoted esterification for a divergent construction of polyester dendrimers up to G_6_.^24^ To speed up the growth of triazine dendrimers, Simanek et al. designed a macromonomer and thus generated two generations per iterative reaction cycle. This strategy facilitated the preparation of G_9_ dendrimer with 1536 terminal groups.^25^ Fernandez-Megia et al. combined the use of atom-economical noncatalyzed thermal Huisgen azide–alkyne cycloaddition of internal alkynes and azide substitution to produce dendrimers up to G_5_ with 96 terminal groups in less than 12 h.^26,27^

Further efforts focus on tailoring the fundamental properties of dendrimers while maintaining their monodisperse nature. For example, heterofunctionalization of DDMs, which had been predominantly achieved through a random statistical approach, was shown by Baker and coworkers to result in a complex mixture of products, from which only a small percentage has the desired multifunctionality.^28^ This has stimulated, among others, Weck and Ornelas, to develop methodologies that allow the heterofunctionalization of polyamide-based dendrimers via a highly controllable orthogonal approach.^29^ However, the properties of dendrimers are not essentially related only to their terminal groups; the internal structure can also play an important active role.^30^ In order to systematically change the inner structure of the branched interior, Roy et al. adopted an “onion peel” approach, employing different building blocks at each stage of the dendritic growth.^31,32^

As we have also experienced during our long-standing effort in the field of carbosilane dendrimer synthesis, the most challenging problem often lies in the separation of pure product from the reaction mixture. Conventional separation techniques, such as extraction or column chromatography, sometimes failed due to the similar nature of the product and reagent or resulted in huge losses. As the biggest difference between the target and impurities in the dendrimer synthesis is their volume, the utilization of size-exclusion techniques, especially those based on membranes, could be advantageous. Surprisingly, membrane techniques are still used only to a limited extent, and they are mostly limited to aqueous dialysis.

In the last years, we have tested the potential of organic solvent nanofiltration (OSN) in the purification of carbosilane dendrimers designed for several applications, ranging from catalysis^33^ to drug delivery^34^ and multivalent dendritic ligands and receptors.^35,36^ Recently, we published a paper summarizing our experience with the implementation of OSN in dendrimer synthesis. The study included a detailed description of the behavior and separation characteristics of commercially available nanoporous regenerated cellulose (RC) membranes and provided guidance that can help to transfer the OSN method to other macromolecular systems and establish it as an efficient separation technique in the field of nanoparticle research.^37^

While carbosilane dendrimers possess many beneficial properties, including chemical and physiological stability,^38,39^ they have a relatively conservative structure without significant potential for modification. Moreover, their preparation is challenging. Particularly, the handling of toxic and unstable chlorosilanes and the catalytic hydrosilylation, highly sensitive to reaction conditions, can contribute to the nonreproducibility of the obtained materials. With the potency of the OSN separation method in hand, we decided to develop a methodology for a modular synthesis of a new type of dendrimer, combining carbosilane and amidic fragments. These poly­(amide-carbosilane) (PAMCAS) scaffolds are constructed stepwise, each time using one of the two basic building blocks having either three or six end groups. We have fully exploited the wide possibilities of their controlled combination and prepared a series of dendrimers in the range of three generations. So far, it has been typical for dendrimers that within a particular structural type, the diversification is realized by peripheral functionalization and by the generational (vertical) growth. With the new combinatorial approach, we can substantially expand the structural variability and realize a multitude of variants in a single generation, thus changing the molecular size and number of end groups not only in the vertical direction (generations) but also horizontally. The obtained compounds demonstrate variations in the branching of the internal structure, the surface density of peripheral groups (PGs), and compactness within the same generation. The described method allowed us to create dendrimers with an unprecedented number of PGs (up to 864) already in the third generation.

## 2 Materials and Methods

### 2.1 General Remarks on Synthesis and Characterization

All commercially available chemicals were used without further purification unless otherwise noted. Chemicals were purchased from the following distributors: cysteamine hydrochloride (CA-HCl) from TCI; *N*,*N*-diisopropylethylamine (DIPEA) from Acros Organics; 2,2-dimethoxy-2-phenylacetophenone (DMPA) from Fluorochem; *O*-(benzotriazol-1-yl)-*N*,*N*,*N*′,*N*′-tetramethyluronium tetrafluoroborate (TBTU) from Carbosynth; anhydrous DMF (99.8%) from Thermo Scientific; CDCl_3_ and DMSO-*d*_6_ from VWR International; CD_3_OD from Acros Organics. Literature procedures were followed in the preparation of tetraallylsilane^40^ and the AB_6_ module.^41^ All other chemicals were from laboratory stock. For nanofiltration, p.a. grade solvents were used; dichloromethane (DCM) was further purified by distillation. Experiments in an inert atmosphere were performed by using the standard septum technique. For filtration through a short chromatographic column, 60 Å (70–230 mesh) silica gel from Lach-ner was utilized.

### 2.2 Nuclear Magnetic Resonance (NMR)

NMR spectra were measured on a Bruker Avance 400 spectrometer (^1^H at 400.1 MHz; ^13^C­{^1^H} at 100.6 MHz; ^29^Si­{^1^H} gated decoupling at 79.5 MHz) at 25 °C (or 100 °C where noted). ^1^H and ^13^C NMR signals were assigned to the corresponding atoms utilizing HSQC, COSY, and HMBC 2D NMR correlation spectra. ^1^H and ^13^C chemical shifts (δ/ppm) are given relative to solvent signals (δ_H_/δ_C_: DMSO-*d*_6_ 2.50/39.52, CDCl_3_ 7.26/77.16, CD_3_OD 3.31/49.00); ^29^Si spectra were referenced to external standard hexamethyldisilane (δ−19.87 ppm). CDCl_3_ and DMSO-*d*_6_ were dried by molecular sieves. CD_3_OD was used as received.

### 2.3 High Resolution Mass Spectrometry (HRMS)

HRMS spectra were acquired using a QTOF mass spectrometer, Bruker Impact II VIP (Bruker Daltonics GmbH & Co. KG, Bremen, Germany), equipped with a heated electrospray ion source and coupled to an Elute PLUS UHPLC system (Bruker Daltonics GmbH & Co. KG, Bremen, Germany). The samples were injected via the UHPLC system without any chromatographic column, allowing direct infusion of the analyte into the ion source. The analysis was performed under isocratic conditions, using a mobile phase consisting of 0.1% (v/v) formic acid in water and 0.1% (v/v) formic acid in methanol in a 10:90 v/v ratio, at a flow rate of 0.3 mL/min. Mass spectra were measured in positive or negative mode, and the mass range of the full MS scan was set from *m*/*z* 150 to 15,000 Da. Ionization parameters were set as follows: capillary voltage: 2500 V; end plate offset: 500 V. The flow of the drying gas was set to 8.0 L/min, dry temperature was set to 260 °C, probe gas temperature was set to 300 °C, and probe gas flow was set to 4.0 L/min. Nebulizer pressure was set to 2.5 bar. HRMS spectra were recorded using Compass 2023b for QTOF Series, otofControl Version 6.3 (Bruker Daltonics GmbH & Co. KG, Bremen, Germany), and processed using Compass DataAnalysis Version 6.1 (Bruker Daltonics GmbH & Co. KG, Bremen, Germany). For calibration of accurate masses, ESI-L Low Concentration Tuning Mix (Agilent Technologies) or ammonium formate clusters were used in each run.

### 2.4 Matrix-Assisted Laser Desorption Ionization Time-of-Flight Mass Spectrometry (MALDI-TOF MS)

MALDI-TOF MS spectra were acquired with the UltrafleXtreme TOF–TOF mass spectrometer (Bruker Daltonics, Bremen, Germany), equipped with a 2000 Hz smartbeam-II laser (355 nm), using the positive ion reflectron mode or linear mode (for molar masses <9 kDa). Panoramic pulsed ion extraction and external calibration were used for molar mass assignment. The dried droplet method was used for both types of samples. In the case of the samples with allyl end groups, the solutions of the sample (10 mg/mL), matrix DCTB (*trans*-2-[3-(4-*tert*-butyl-phenyl)-2-methyl-2-propenylidene]­malonitrile, Sigma-Aldrich, 10 mg/mL), and ionizing agent sodium trifluoroacetate (CF_3_COONa; Sigma-Aldrich, 10 mg/mL) in THF (Sigma-Aldrich, anhydrous, 99.9%) were mixed in the volume ratio 4:20:1. 1 μL of the mixture was deposited on the ground-steel target. In the case of the samples with charged end groups, the solutions of the sample (10 mg/mL), matrix DHB (2,5-dihydroxybenzoic acid; Sigma-Aldrich, 98%, 20 mg/mL), and ionizing agent sodium chloride (NaCl; Sigma-Aldrich, 10 mg/mL) in H_2_O were mixed in a volume ratio of 4:20:1. 1 μL of the mixture was deposited on the ground-steel target.

For Dynamic Light Scattering (DLS), diffusion NMR, Asymmetric Flow Field-Flow Fractionation (A4F), and Molecular modeling, see SI Sections 3, 4, 5 and 6, respectively.

### 2.5 General Procedure of Amidic Coupling (GP1)

A dendrimer with terminal ammonium functional groups was put into a round-bottom flask, and AB_3_ or AB_6_ module (1.25 equiv per ammonium group) and TBTU (1.25 equiv per ammonium group) were added. The flask was filled with argon, and dry DMF (5 mL per 0.1 g of dendrimer) was added. The mixture was stirred at 50 °C for 10 min. DIPEA (3 equiv per ammonium group) was slowly added, and the reaction mixture was stirred at 50 °C for 2–3 h. After cooling to laboratory temperature, EtOAc (2–5× the volume of DMF) was added, then, the solution was washed (2 × 2% aq sol. of KOH, 2 × 1 M aq sol. of HCl, 2× water, and 1× brine) and dried by anhydrous MgSO_4_. The solvents were removed on a rotary evaporator, and the residue was purified by OSN in DCM with as high a content of MeOH as possible to keep the solution clear (see below for details). The products were obtained as brownish viscous substances in lower generations or as brownish resin-like solids in higher generations in 80% to quantitative. Particular yields and analytical data of all products are given in SI.

### 2.6 General Procedure of Thiol–Ene Click (TEC) Reaction (GP2)

A dendrimer with terminal allyl functional groups was put into a thin-walled vial. CA-HCl (1.33 equiv per double bond), DMPA (0.1 equiv per double bond), and a minimum volume of DMF (approximately 1–2 mL per 0.1 g of dendrimer) were added, and the reaction mixture was purged with argon for 10 min during stirring. The reaction mixture was irradiated by a 400 W mercury lamp through a PYREX filter (main transmitted wavelength, 350 nm) at room temperature while stirring for 20 min. After the first phase, the solvent was removed on rotary evaporator, new portions of CA-HCl (0.66 equiv per double bond), DMPA (0.1 equiv per double bond), and a minimum volume of MeOH (approximately 1–2 mL per 0.1 g of dendrimer) were added, the reaction mixture was purged with argon for 10 min during stirring, and the TEC was carried out again for 20 min. If the traces of double bonds were observed in the crude mixture (multiplets at 4.75–4.90 and 5.70–5.80 ppm in ^1^H NMR in DMSO-*d*_6_), the second phase was repeated (usually no more than once). Once the conversion was quantitative, the product was isolated from the crude mixture by OSN in MeOH (see below for details), the solvent was evaporated, a minimum volume of deionized water (1–2 mL) was added, and the solution was frozen and lyophilized to obtain white powder in 83% to quantitative yields. Particular yields and analytical data of all products are given in SI.

### 2.7 Organic Solvent Nanofiltration (OSN)

OSN was carried out using a solvent-resistant stirred cell (Millipore) equipped with 1, 3, or 10 kDa MWCO (depending on dendrimer molar mass) regenerated cellulose ultrafiltration discs, Ultracel (Millipore, Merck KGaA, Darmstadt, Germany), and PTFE-encapsulated O-rings (ERIKS), with nitrogen as a driving gas at transmembrane pressure of 4.5–5.0 bar.^37^ *Batch mode:* the separated mixture was quantitatively transferred into the cell, diluted with the solvent to a total volume of 10–30 mL depending on the batch size, and passed through the membrane until approximately 1/10 of the initial volume was left. The cycle was repeated until sufficient purity was reached according to ^1^H NMR (4–20 cycles). *Continuous mode:* the separated mixture was quantitatively transferred into the cell, and the retentate volume was reduced in the batch mode to a minimum (at least 2 mL to enable efficient stirring). Then, the solvent reservoir was attached to the cell, and the pressure difference was set to balance the inflow of fresh solvent and the outflow of filtrate, maintaining a constant retentate volume. The purity of the retentate was regularly checked by ^1^H NMR. Removal of solvents was performed on a rotary evaporator at a maximum of 50 °C, if not stated otherwise. *Transmission tests* were carried out in the batch mode, with the initial solute concentration of 5 mg/mL and an initial volume of 9 mL, starting from pure DCM. After 2.7 mL of the filtrate was collected and discarded, a 0.25 mL filtrate sample was collected, and the filtration was stopped. A 50 μL sample was taken from the retentate, the retentate was diluted to 9 mL to obtain the next tested solvent ratio, and the procedure was repeated. After performing the test at the highest applicable polarity (2:1 MeOH/DCM), the cell was disassembled, and the membrane was washed and sonicated several times. The sequence was repeated on the same membrane twice more, once going in the opposite polarity direction and once again with increasing polarity to check the reproducibility of the results; for each sequence, a fresh starting solution was prepared. The samples were diluted as necessary, and the concentration of solutes was quantified by UV spectroscopy. Transmission of solute *r* was then calculated from its concentration in filtrate (*c*_*f*_) and retentate (*c*_*r*_) as

1 $$r=c_{f}/c_{r}$$

## 3 Results and Discussion

The protocol for the synthesis of new dendrimers was designed to fulfill several requirements. First, we looked for a straightforward strategy that would facilitate the generation of rationally designed dendrimers suitable for use in various areas of functional materials research including biomedical applications. The synthesis based on a modular approach allows us to control and finely manipulate the structure during each step of its construction. We also envisioned fully utilizing the OSN as the main separation method. The two employed building blocks with a carboxyl group at the focal point and three (AB_3_) or six (AB_6_) allyl groups at the periphery have a limited molecular weight (MW = 330 and 539 g/mol, respectively) so as to theoretically pass through a tight ultrafiltration RC membrane with a nominal 1 kDa molecular weight cutoff (MWCO). This was also advantageous in discovering the capabilities and limits of the OSN method. Two possible variants of building blocks AB_3_ or AB_6_ allow for the control of the branching of the interior structure and density of peripheral groups. AB_6_ module provides an unprecedented branching, enabling acceleration of the growth of dendrimer molecular weights and the number of peripheral groups. After appropriate workup, the unreacted building blocks applied in excess and subsequently separated by the OSN could also be efficiently reused for further reactions, thus adding to the sustainability of the synthesis.

### 3.1 Dendrimers G_1_

All dendrimers grow out from a tetravalent core of G_0_-N with silicon as a branching center. The core was simply prepared from tetraallylsilane by thiol–ene click (TEC) addition of cysteamine hydrochloride (CA-HCl). Both building blocks AB_3_ and AB_6_ consist of one or two flexible wedges featuring *sp* ^3^ carbon chains and a silicon branching atom, connected to a rigid aromatic ring bearing a carboxylic group as an anchoring point. The iterative procedure based on a sequence of amidic coupling and atom-economic TEC reaction, each followed by OSN separation and purification step, will be described in detail for the first generation of dendrimers. This sequence is then repeated in the appropriate order to obtain higher generations.

The synthesis starts with the AB_3_ or AB_6_ macromonomer attachment to the core, employing amidic coupling. In both variants, we selected a well-established combination of TBTU and DIPEA as an efficient coupling strategy^42^ and applied a 25% excess of the macromonomer. The course of the reaction was monitored by NMR spectroscopy. As a result of amide formation, the well-isolated triplet assigned to methylene protons adjacent to the reacting ammonium group was shifted from 2.93 to 3.59 ppm. Typically, full conversion was achieved within 2 h at 50 °C. Aqueous extraction was applied to remove the major part of unreacted TBTU and its end products, and after subsequent OSN separation of remaining impurities and excess building blocks, the polyallylic dendrimers G_1_-3-A and G_1_-6-A were obtained in high yields (89% and 95%, respectively). Both compounds are thick oils, highly soluble in DMF, DMSO, THF, chlorinated solvents, and their mix with methanol up to 65 vol %, and they are chemically stable toward weak acids and bases in a pH range of 2–12. NMR and HRMS spectroscopy confirmed both of these structures. The amidic coupling was considered quantitative, as no structures containing unreacted ammonium groups were detected by any of these methods.

Interestingly, we observed a difference in the stability of the activated macromonomer substrates: the AB_3_ block was isolated from the OSN filtrate as a stable adduct with a TBTU fragment (activated benzotriazole ester BtO-AB_3_, analytical data included in SI), whereas the AB_6_ block was present mostly as a free acid. This difference is undoubtedly due to the diverse substitution pattern of the aromatic ring: the electron-donating alkoxy group in the para position supports the stability of the activated ester BtO-AB_3_, while its counterpart BtO-AB_6_ is always decomposed during the aqueous workup due to its higher reactivity, originating from double *meta-*alkoxy substitution. In spite of this reactivity difference, the coupling is equally efficient in both cases, likely because the higher reactivity of the larger block compensates for its increased steric demands and slower diffusion. Both AB_3_ and AB_6_ modules can be recovered from OSN filtrates almost quantitatively and reused (for details, see SI Section 3, Figure S23). The introduction of ammonium groups to the periphery in the second reaction step was achieved through the UV-initiated TEC reaction promoted by the photoinitiator DMPA. Given the significant change in dendrimer polarity in the course of the reaction, resulting in different solubilities of the starting material and the product, the reaction had to be carried out as a two-step process. The first step was accomplished within 20 min in DMF at room temperature with excess CA-HCl (1.33 equiv per double bond). Then, the solvent was removed on a rotary evaporator, and new portions of CA-HCl (0.66 equiv per double bond) and MeOH, a more polar solvent, were added. The TEC was carried out under an argon atmosphere for the next 20 min. If any traces of double bonds were observed in the crude mixture (well-isolated multiplets at 4.75–4.90 and 5.70–5.80 ppm in ^1^H NMR in DMSO-*d*_6_), the second phase was repeated (usually not more than once). After the use of the OSN, the isolated yields of cationic dendrimers G_1_-3-N and G_1_-6-N were 91% and 98%, respectively. Both ammonium dendrimers are solids well soluble in DMSO, methanol, and water. They can be lyophilized to fine white powders and long-term stored in ambient conditions without degradation or moistening. When treated with a base, they lose their polycationic character and become insoluble in water.

Both polycationic dendrimers were characterized by MALDI-TOF and ESI-QTOF HRMS (for details, see SI Section 2, Figures S1–S22). The overall defectiveness of G_1_-3-N and G_1_-6-N, as estimated from mass spectra, was lower than 1% of the missing end groups.

### 3.2 Nanofiltration of Dendrimers

Since the OSN is an integral part of the presented synthetic protocol, its optimization was crucial both for the purity and yields of the dendritic materials and for the throughput of the designed synthetic pathway. From this point of view, the first generation of dendrimers posed a considerable challenge because of a very unfavorable MW ratio between the separated species: 4.9:1 for the G_1_-6-A:AB_6_ pair and only 3.9:1 in the case of G_1_-3-A:BtO-AB_3_. To the best of our knowledge, there are no documented examples of the use of an OSN in this way, so extensive optimization was necessary.

The pore size and, therefore, the separation ability of nanoporous RC membranes were proved to be solvent-sensitive.^37^ In this case, because of the low polarity of the separated molecules, the highest applicable solvent polarity reached a 1:1 to 1:2 DCM:methanol ratio, depending mostly on the building block type and its quantity in the separated mixture. To find the optimum separation conditions and predict the necessary number of cycles, we evaluated the transmission (i.e., instant filtrate-to-retentate concentration ratio) of both macromonomers in the applicable solvent polarity range (Figure 1a,c), using UV spectroscopy for quantification. As the activated ester BtO-AB_3_ slowly decomposes in methanolic solution to give the corresponding methyl ester and HOBt, which would make the data evaluation very complex, the transmission of the AB_3_ block was tested on its methyl ester. The AB_6_ block was studied in the form of a dimethylamide (Me_2_N-AB_6_, a product of the reaction of the activated ester with traces of dimethylamine inherently present in DMF), as the largest and most stable form contained in the filtered reaction mixtures after aqueous workup, along with the free acid and methyl ester. For both tested solutes, we observed solvent-dependent behavior consistent with previous findings.^37^ On a 1 kDa membrane, the smaller solute MeO-AB_3_ shows a maximum transmission for the maximal applied solvent polarity and the transmission minimum for 4:1 DCM:methanol ratio, which is typical for esters under a certain membrane-dependent size (Figure 1a). Preliminary experiments showed that the transmission of the larger AB_6_ block through a 1 kDa MWCO membrane was negligible; fortunately, the G_1_-6-A dendrimer was found to be large enough to be efficiently retained in a nonaqueous environment by a 3 kDa MWCO membrane. On this membrane, Me_2_N-AB_6_ shows a gradual increase in transmission with an increasing ratio of methanol in the solvent mixture, which results from both its larger size and lower polarity (Figure 1c). The experiments were repeated three times, starting at the lowest polarity and reversing the polarity direction after each set of points. The data show good reproducibility, implying a low level of membrane fouling under the applied conditions. Observed transmission values are very low, ranging from 0.02 to 0.12 for the larger block on the 3 kDa membrane and from 0.07 to 0.30 for the smaller block on the 1 kDa membrane. The major target for the OSN separation in the case of AB_3_ blocks, the activated ester BtO-AB_3_, is expected to show a lower transmission due to its lower polarity and larger size compared with the methyl ester.

**Figure 1:**
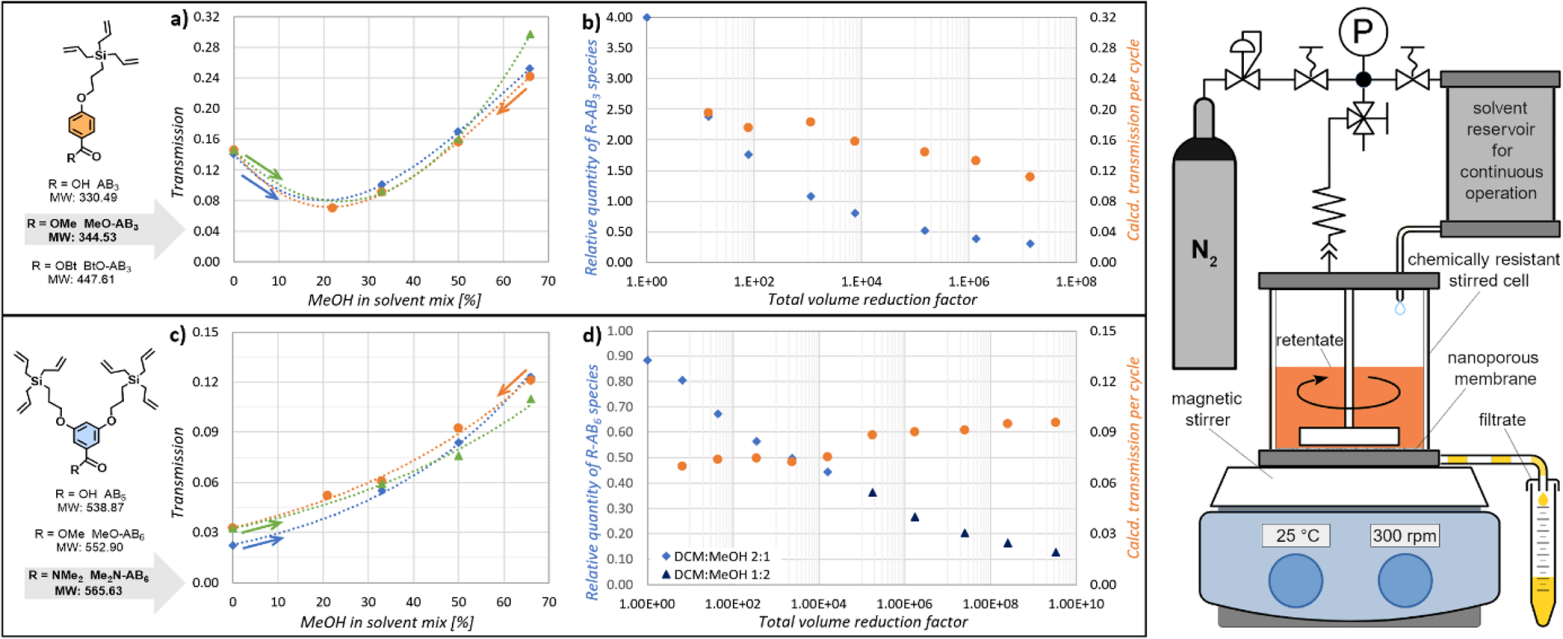
Nanofiltration of AB_*x*_ modules in binary mixtures of DCM and methanol. AB_3_ module/1 kDa membrane: solvent-dependent transmission of MeO-AB_3_ (a); concentration of impurities and mean transmission of the module (sum of all forms) during purification of G_1_-3-A dendrimer in a 1:1 mixture (b). AB_6_ module/3 kDa membrane: solvent-dependent transmission of Me_2_N-AB_6_ (c); concentration and mean transmission of the module (sum of all forms) during purification of the G_1_-6-A dendrimer (d). In graphs (a,c), color is used to distinguish the test series: 1st series with increasing polarity (blue diamonds); 2nd series with decreasing polarity (orange circles); and 3rd series with increasing polarity (green triangles). The nanofiltration apparatus setup is depicted on the right.

For comparison, Figure 1b,d shows the results of the nanofiltration of actual reaction mixtures. In this case, the UV method was not suitable due to the presence of more solutes with overlapping absorbance spectra in the samples, so the quantification relied on less precise ^1^H NMR. In the case of the AB_3_ module (Figure 1b), also the NMR quantification was problematic due to the close proximity of the signals of interest in the spectra and a slow conversion of BtO-AB_3_ to the corresponding methyl ester, which resulted in the fluctuation of the mean transmission value per cycle. Nevertheless, the overall mean transmission (0.17) is close to the value obtained in transmission tests for pure MeO-AB_3_. In the case of the AB_6_ module (Figure 1d), there is a good agreement between the mean transmission of the sum of all AB_6_ derivatives calculated for each cycle according to the model^37^ and the value from the transmission tests of Me_2_N-AB_6_ for a given solvent mix: an increase in purification efficiency after increasing the solvent polarity at the sixth cycle is clearly apparent. As the low transmission values resulted in slow removal of the macromonomers, requiring many (8–20) filtration cycles and consuming a lot of solvent and time, we sought a way to increase the nanofiltration efficiency. We adapted the dead-end stirred cell for a continuous operation by adding a pressurized solvent reservoir and performed the separation in a diafiltration mode at the lowest possible solution volume. This was especially useful for larger product batches (up to 5 g), which markedly decreased the solvent consumption and the required time.

The purification of polycationic dendrimers after TEC reaction was much easier, as the largest separated reagent, DMPA photoinitiator, has a MW of only 256 g/mol. However, we encountered unprecedented behavior in these polyelectrolytes, demonstrating extreme solvent affinity. The solutions could not be concentrated to low volumes, as the flow of the filtrate decreased at a much lower retentate concentration than in the case of their nonpolar precedents. This effect can be attributed to two factors: (1) markedly higher viscosity of the solutions of polyammonium dendrimers compared to their polyallylic counterparts, which hinders the mass transport, and (2) the nonnegligible influence of osmotic pressure in the case of ammonium polyelectrolytes, which can be expected to show some level of dissociation even in methanolic solution. So, the continuous nanofiltration mode was also beneficial for the purification of these charged species because the efficiency of the batch mode relies on volume minimization.

### 3.3 Dendrimers G_2_ and G_3_

When we verified the feasibility of the proposed protocol for the first generation of PAMCAS dendrimers, we proceeded with the construction of the second generation by repetition of both optimized reaction sequences. First, building on G_1_-3-N and G_1_-6-N scaffolds, homolayered allylic G_2_-3-3-A and G_2_-6-6-A dendrimers were synthesized accordingly in high yields (93% and 98%). These were subsequently converted to the corresponding ammonium dendrimers G_2_-3-3-N and G_2_-6-6-N in 98% and 94% yields, respectively. Due to the very similar surroundings of the first and second layers, the signals of the corresponding groups from both layers often overlap in the NMR spectra. The best resolution was observed for G_2_-3-3-A, where proton signals of OCH_2_C*H*_2_CH_2_Si groups from both layers are separated even in the 1D spectrum, and all different CH_2_Si groups can be distinguished in the 2D spectra. Also, some ^13^C signals are clearly resolved (by 0.1–0.2 ppm), namely those due to the aromatic rings, adjacent carbonyl groups, and propyleneoxy spacers. Whereas for both G_2_-3-3-A and G_2_-3-3-N, all three signals attributed to different silicon atoms are visible in ^29^Si NMR, the signal of the core silicon was not detectable for larger 6-6 dendrimers due to an unfavorable ratio; G_2_-6-6-N displays only a single silicon signal.

Already in the second generation, there is a huge gap in the number of peripheral groups between the AB_3_ and AB_6_ homolayered series (36 vs 144). Two commutable variants of macromonomers allow for a simple combinatorial approach to building the interior of dendrimers and consequently tuning both the internal density and the density of peripheral groups. Both possible combinations leading to heterolayered polyallylic dendrimers G_2_-6-3-A and G_2_-3-6-A were prepared. Then, ammonium groups were introduced by the TEC reaction to provide G_2_-6-3-N and G_2_-3-6-N. These two pairs of dendrimers carry the same number of peripheral groups, but they can be expected to substantially differ in the density profile of their interior. Different substitution pattern of aromatic rings in both layers allows for a better resolution of particular groups in NMR spectra, not only the aromatic protons and carbons but also the carbonyl groups and some of the methylene groups in adjacent aliphatic chains. Also, all the different silicon atoms are well resolved.

The third generation of dendrimers fully demonstrates the potential of the adopted approach. Generally, from two different macromonomers, we can obtain 2^*n*^ unique structures for a given generation *n* by their sequential combination, which translates to eight compounds in the third generation (Scheme 1). In each case, the number of structures with equal number of end groups can be derived from Pascal’s triangle; for this particular generation, it means one of each of the homolayered structures, the smallest (108 PGs) and the largest one (864 PGs), three compounds with 216 PGs, and another three having 432 PGs (1:3:3:1). Again, the dendrimers sharing a number of end groups should differ in the density distribution within their molecule. The library of all 8 prepared compounds depicted in Figure 2 covers a broad range of MW from 32 kDa to more than 200 kDa. The homolayered AB_6_ line is unique among all dendrimers reported to date in terms of the growth rate of the molecular weight and the number of peripheral functional groups. For comparison, widely used PAMAM dendrimers have 16 (3256 g/mol) and 32 (6909 g/mol) PGs in the second and third generations, respectively;^43^ more compact classical polyallylcarbosilane dendrimers with maximum branching degree can achieve 36 and 108 end PGs, while being comparable in size to PAMAM type (2626 and 8101 g/mol, respectively).^40^ The exceptionality of the AB_6_ module lies in its double branching: the first one on the aromatic ring and the second one at silicon atoms. Schematic structures of all representatives in Figure 2 show the different compositions of layers within the third generation of PAMCAS dendrimers and the corresponding aromatic regions of the ^1^H NMR spectra. The derivatives are ordered upward by increasing the ratio of AB_3_ to AB_6_. The aromatic systems of AB_3_ and AB_6_ represent spin systems AA′XX′ and A_2_X, respectively, with adequate intensity of proton signals. Increasing the MW of dendrimers and the related number of branching points is accompanied by the broadening of signals caused by the shortening of T_2_ relaxation.

**Scheme 1:**
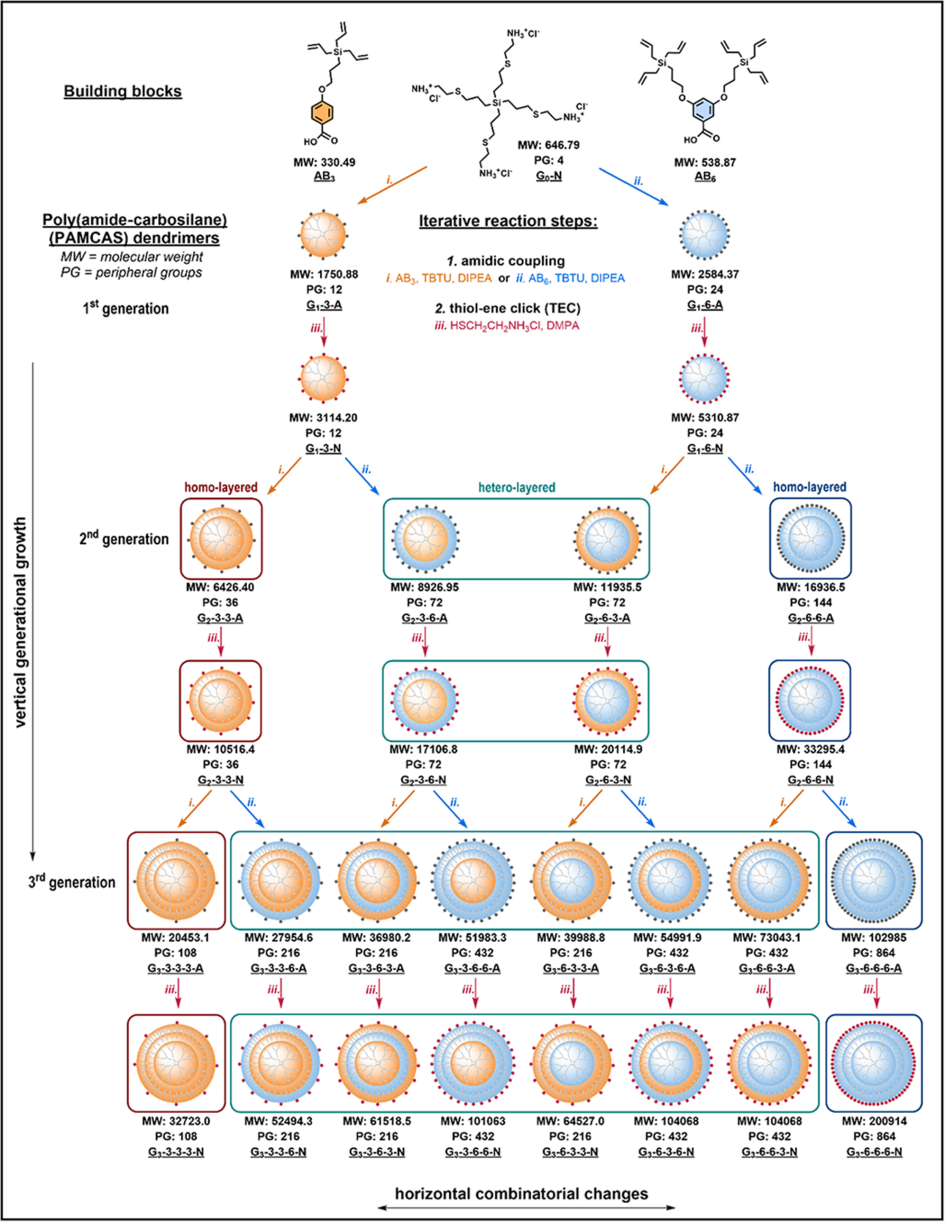
Synthetic Protocol for PAMCAS Dendrimers and Overview of All Prepared Compounds

**Figure 2:**
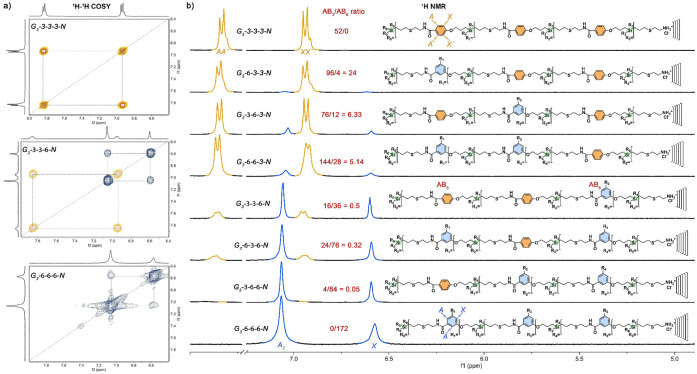
NMR characterization of the aromatic region of the 3rd gen. PAMCAS dendrimers. ^1^H–^1^H COSY (a), ^1^H NMR and linear representation of the corresponding structures (b).

The properties of higher-generation dendrimers (solubility, stability) are similar to those of their smaller analogs. An increasing density of allyl groups at the periphery as a result of replacing the AB_3_ motif with AB_6_ in the structure leads to an apparent lowering of polarity and decreases the tolerance of the compounds for methanol in solvent mixtures with DCM (from 65 vol. % for G_2_-3-3-A to 40 vol. % for G_3_-6-6-6-A). Ammonium dendrimers retain their solubility regardless of the number of PGs, but with increasing density of ammonium groups, their concentrated solutions become turbid, and they show an increasing hygroscopic tendency in the solid state.

Based on the NMR and MS data (SI Sections 1, 2, and 8), it can be concluded that the defectiveness of the periphery of higher-generation dendrimers is similar to that of the first generation. Again, polyallyl dendrimers showed complete conversion of the amidic coupling, while some defect structures corresponding to a max. 2% of unreacted end groups were detected in the MS spectra of ammonium dendrimers. At this level, the products seem defect-free in ^1^H NMR (no apparent signals of allyl groups). Nevertheless, repeating the TEC steps in pure MeOH should increase the conversion even more if higher purity is needed.

### 3.4 Solution Properties of Ammonium Dendrimers

The synthesis and characterization of the PAMCAS dendrimers were followed by the study of their behavior in solution. We used multiangle dynamic light scattering (MADLS) to determine the hydrodynamic (more generally solvodynamic) diameters (*d*_h_) of all ammonium dendrimers (Figure 3b). Although all of them are water-soluble, those with a peripheral layer built of AB_6_ module showed intense aggregation in standard physiological solution (0.9% NaCl in water) into particles larger than 100 nm, which suppressed the signals of the dendrimers themselves. Thus, in order to obtain data comparable across the series, we decided to study all compounds in a 0.25% NaCl solution in methanol. In this medium, only G_3_-6-3-6-N showed approximately 10 vol. % of the mass aggregated into particles of sizes between 30 and 150 nm, and four other AB_6_ ended dendrimers contained traces of aggregates, but the solvodynamic diameter of the individual molecules could be measured with high precision (Figures S25–S38). When comparing the sizes of the AB_3_ ended dendrimers determined in methanol to the results obtained previously in physiological solution, the values did not differ by more than 7%. The overall size of the dendrimers covers a range between 3.0 and 13.0 nm (Figure 3b). The values consistently reflect the generation and structure of the molecules (Table 1). The dendrimer size increases with the growing generation, as expected, but we can also observe subtle variations in the horizontal direction within each generation, reflecting the change in the composition of the layers. When AB_6_ substitutes the AB_3_ layer, the size increases by 0.8–2.9 nm, with the greatest influence in the highest generation when substituting the innermost layer (Table S1).

**Figure 3:**
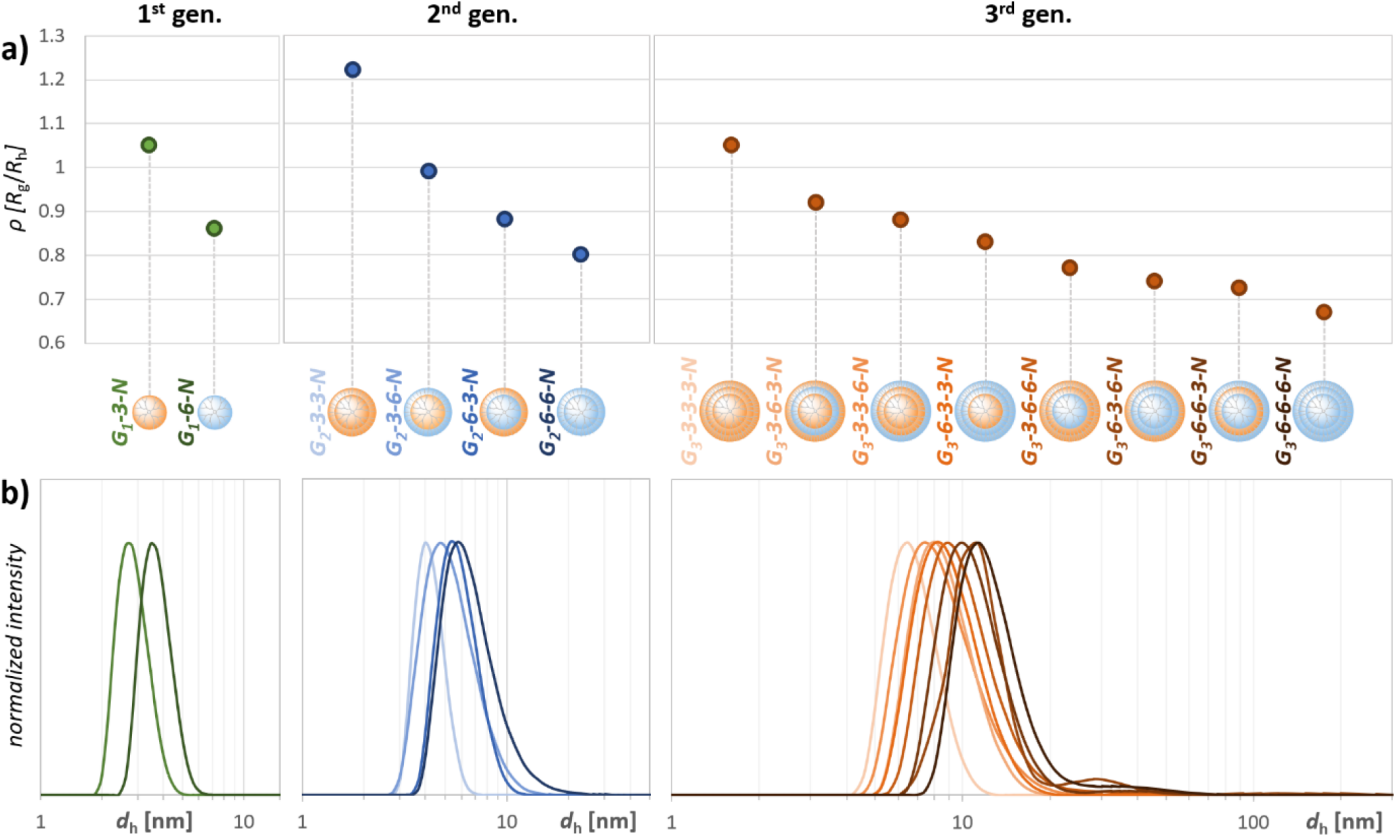
Shape and size characteristics of ammonium PAMCAS dendrimers: (a) shape factor ρ = *R*_g_/*R*_h_ (*R*_g_ from molecular simulations, *R*_h_ = *d*_h_/2 from MADLS); (b) intensity-weighted size distribution from MADLS. Structures are arranged in ascending order according to their experimentally determined diameter.

**Table 1:**
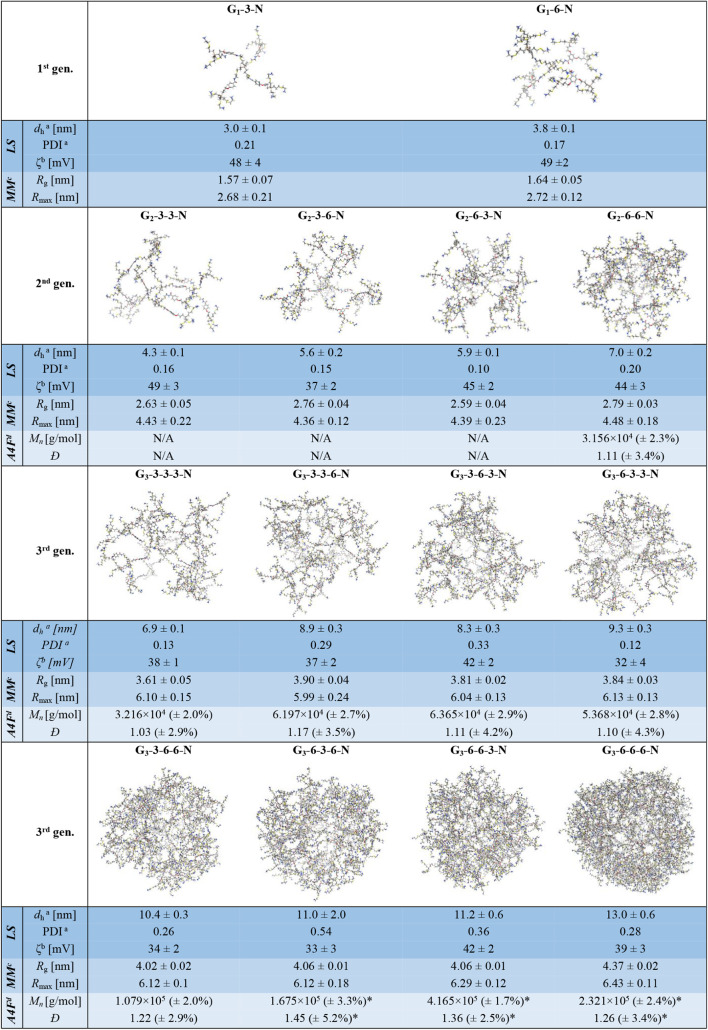
Structure-Related Data of Ammonium Dendrimers from Experiments and Molecular Dynamics Simulations

a Solvodynamic diameter (*d*_h_) and polydispersity index (PDI): data from MADLS measured in 0.25% NaCl solution in MeOH.

b Zeta potential (ζ) measured in physiological solution (0.9% NaCl in water).

c Average radius of gyration (*R*_g_) and average maximal distance of the dendrimer atoms from the dendrimer center of geometry (for spherical molecules, an estimate of their radius) (*R*_max_), simulated using molecular dynamics in 0.25% NaCl solution in MeOH.

d Number-average molecular weight (*M*_n_) and dispersity (*Đ* = *M*_w_/*M*_n_): data from A4F in water; *aggregation.

Figure 4a shows the relationship between the hydrodynamic diameter of dendrimers determined by MADLS and the cubic root of their theoretical MW, which is proportional to the particle size. Observed nearly linear dependence points to only negligible variation in the density.

**Figure 4:**
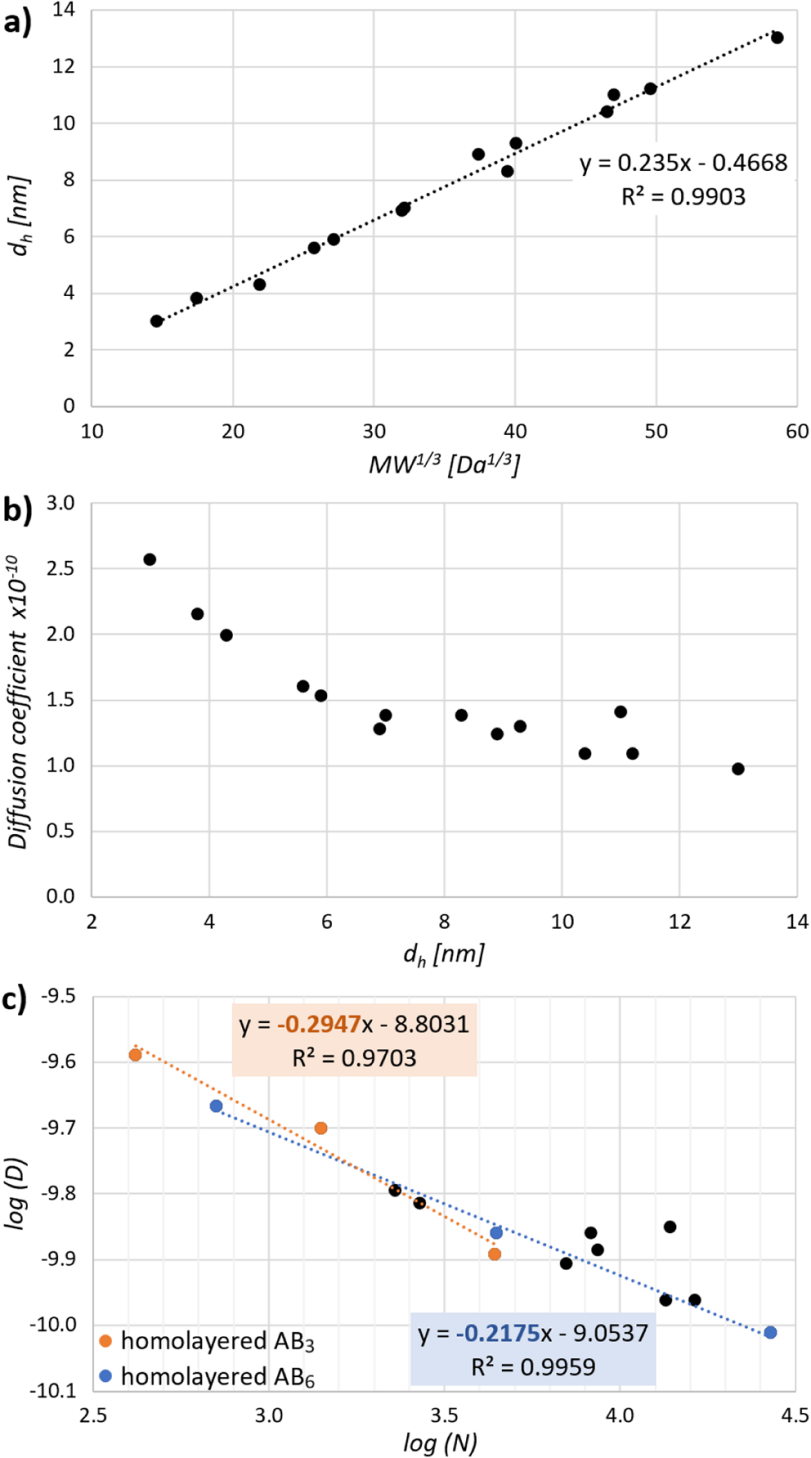
Relationships between experimental and theoretical values related to the dendrimer size: (a) hydrodynamic radius of dendrimers determined by MADLS as a function of the cubic root of their molecular weight; (b) relation between the diffusion coefficient obtained from diffusion NMR and the hydrodynamic radius of dendrimers determined by MADLS in the same solvent; (c) log–log representation of the diffusion coefficients vs the theoretical number of atoms for perfect dendrimers.

To obtain more information on solution behavior of ammonium dendrimers, we performed a diffusion NMR study using the deuterated analogue of solvent used for light scattering experiments. This enabled a direct comparison of the results from both methods. Figure 4b shows the relation between the diffusion coefficient calculated for the particular dendrimers from the diffusion NMR measurements and the respective hydrodynamic diameters. The Stokes–Einstein equation postulates an inverse proportion between the diffusion coefficient and the particle radius in solution, but its validity is restricted to spherical particles.^45^ For irregular particles, deviations from linearity could be expected. Previous papers report an exponential dependence of diffusion coefficient on the number of atoms for a series of structurally related macromolecules. The exponent can be derived from the slope of the log–log representation of the respective function (Figure 4c). As our dendrimers do not constitute a structurally uniform group, combining two different building blocks in their scaffold, also, the values are somewhat scattered. But, taking into account only the homolayered structures, we can calculate two scaling constants for AB_3_ and AB_6_ series with the values approximately −0.29 and −0.22, respectively. A direct comparison of these values with the values reported for other dendrimer types, such as PAMAMs (−0.35) and polyamide dendrimers^45,46^ (−0.36) is problematic, as they are not obtained in the same solvent, but they are consistent with the expected more compact structure of the AB_6_ series compared to AB_3_.

Subsequently, we measured the zeta potential of the ammonium derivatives in physiological solution to determine the charge density at the dendrimer surface (Table 1). All studied compounds show zeta potential in the range of 32–49 mV, which corresponds to good colloidal stability of the dendrimers and their aggregates in physiological solution.^47^ The observed values do not show a simple correlation with the size of dendrimers or theoretical PG density; nevertheless, some features related to the structure can be recognized. The values decrease with increasing generation, and considering the structures bearing the same number of PGs, the ones having a layer sequence ending in a 6-3 pattern show the highest zeta potential. Both effects can originate in the extent of backfolding of charged end groups into the inner structure, which is clearly higher for larger structures but limited by the presence of denser AB_6_ layers in the interior.

Next, we used multidetector asymmetric flow field-flow fractionation (A4F) to determine the molecular weight characteristics and dispersities of G_2_ and G_3_ ammonium dendrimers. We considered A4F analysis as superior to GPC since, as reported by Podzimek et al.,^48^ highly branched macromolecules can show anomalous behavior in GPC, whereas no such artifacts are found in asymmetric flow field-flow fractionation due to the different separation mechanism. Moreover, our attempts to use GPC for dendrimer characterization failed even for the low-generation ones due to strong adsorption of the compounds to the column (PL aquagel–OH). An optimization of operating parameters, especially the cross-flow value and the focusing and relaxation times, was necessary to suppress aggregation; still, some results were significantly affected by the presence of aggregates, probably due to focusing, which is essential for analysis but can induce aggregation.

Since the *dn/dc* values of the dendrimers were not known for the A4F analysis, the assumption of accurate concentration and 100% sample mass recovery (meaning 100% retention of the dendrimers in the separation channel) was used for the MW calculation. However, due to the molecular weight cutoff of the membrane used in the separation channel of the instrument (10 kDa MWCO means only 90% rejection of 10 kDa molecules), this was not the case for most of the G_2_ dendrimers. The analysis of G_2_-3-6-N (theoretical MW 17.107 g/mol) showed highly overestimated results and high dispersity, clearly as a consequence of incomplete retention, so we decided to analyze only the samples having a theoretical MW over 30 kDa (Figures S39–S47). The results for G_2_-6-6-N and all G_3_ ammonium dendrimers are presented in Table 1. The number-average molecular weight values (*M*_*n*_), which represent the numerically most abundant molecular weight, are very close to the expected values for samples showing a low dispersity. These samples are characterized by the absence of intermediate-sized particles; i.e., a complete separation of the population of single molecules from the population of aggregates is achieved in the course of measurement. In other cases, we observed large positive deviations in *M*_*n*_ evincing the presence of small dendritic clusters, which is also apparent from the data from the A4F light scattering detector (the tailing of peak 1 and the poor baseline separation of peaks is especially apparent in Figures S45–S47).

To complete the information on the structure and dynamics, the PAMCAS dendrimers were also studied on a molecular level by using molecular simulations in a 0.25% NaCl solution in methanol. All technical details regarding simulations can be found in the Supporting Information and in refs 41,49–57 . Computational models show that all generations of dendrimers maintain a spherical shape with the variability in size and density of the inner structure. Detailed structural information about the individual dendrimers is provided by radial distribution functions (RDF) of their representative atoms. The distributions of methanol and ions with respect to the central Si atom are also described in this way (Figures S49–S62). Certain RDF profiles, especially those involving terminal N atoms, provide valuable information about the dimensions of individual molecules and additional structural details. In the case of the G_2_ dendrimers, RDF of the terminal N atoms with respect to the central Si atom revealed more pronounced backfolding for dendrimers containing AB_3_ module in the inner layer (G_2_-3-3-N, G_2_-3-6-N) compared to the remaining structures (G_2_-6-3-N, G_2_-6-6-N) with more crowded AB_6_ motif in the same shell. Higher interior density obviously limits the possibility that the branch ends penetrate the dendrimer center (Figures S51–S54); the same point resulted from the zeta potential values (Section 3.4). It can be assumed that the structures with larger inner voids, enabling easier backfolding of the end groups, would also exhibit higher loading capacity for small neutral molecules, e.g., lipophilic drugs. Selected size characteristics—mean maximal distance of the dendrimer atoms from the molecular center of geometry (*R*_max_) and mean radius of gyration (*R*_g_)—are presented in Table 1. For compact spherical molecules, *R*_max_ estimates well their *R*_h_, as we can see especially in the case of G_3_-6-6-6-N. While *R*_max_ value is almost constant within one generation due to the same number and type of bonds between the dendrimer core and periphery, *R*_g_, which is defined as the square root of the mass-weighted mean of the squared distances of all constituent particles from the center of mass of the system and provides a measure of how the mass is spatially distributed around the center of mass, reflects more the subtle changes in dendrimer structure, especially in the third generation.

Calculation of the shape factor (ρ = *R*_g_/*R*_h_) provides a quantitative indicator of the molecular conformation. The expected value for a hard sphere (globular particles) is 0.775, which means that *R*_g_ is smaller than *R*_h_.^58^ When molecules deviate from globular to elongated shape, *R*_g_ becomes larger than *R*_h_ and ρ tends to increase. Its value corresponds to the degree of flexibility or rigidity as higher flexibility implies larger variability of conformations differing in the degree of deviation from the spherical shape.^59,60^ In our case, the ρ values reflect well the gradual changes in the radial density profile of the dendrimers (Figure 3a). With the increasing generation, we encounter more rigid, spherically shaped derivatives (for the three consecutive generations of homolayered AB_6_ dendrimers, ρ = 0.86, 0.80, and 0.67). Similarly, we can see a gradual decrease in the shape factor in the horizontal direction (within one generation) as the AB_3_ layers are substituted by AB_6_. Some values are slightly below the theoretical value for a hard sphere. Interpretation of these results should be done carefully, focusing rather on general trends than on the values themselves, as we can expect some systematic error due to the different data sources for *R*_g_ (theoretical) and *R*_h_ (experimental, most likely including the solvation envelope). All dendrimer structures are fairly permeable to methanol, which is present in nearly bulk density already at distances approximately 3–4 Å from the central Si atom (Figures S49–S62). The distribution of Cl^–^ anions closely follows the distribution of positively charged terminal NH_3_^+^ groups.

## 4 Conclusion

The presented synthetic strategy constitutes a robust, accelerated, scalable, and tunable approach to large, low-dispersity dendritic structures with variable density of peripheral functional groups. Using only three building blocks (core and two branched monomers), we synthesized a series of horizontally stratified homologues up to the third generation. All reactions are high-yielding, reproducible, and easy to perform. Nanofiltration purification proved to be a key technique in this modular synthesis, as applied after each step, it ensures high purity and excellent yields of products, and at the same time, it also enables the recycling of excess used dendritic modules which adds to the economy of the method. Currently, we investigate the size limitations of the reported protocol in order to obtain soluble, low-dispersity macromolecules with MW over 10^6^ Da and with sizes approaching virion particles.

The prepared dendrimers represent a novel structural type, characterized by a medium-polar interior capable of hydrogen bonding and π–π stacking, as well as of hydrophobic interactions. Unlike PAMAMs, PAMCAS dendrimers do not contain protonable amines in their interior; therefore, considering the same peripheral groups in both cases, their cytotoxicity should be lower.

Experiments with ammonium PAMCAS dendrimers in solution revealed their low dispersity, stability of covalent structure, compact globular shape, and medium-high positive zeta potential. Their tendency to form aggregates in an aqueous environment needs to be taken into account for specific uses. Their properties, together with the easy tunability and possibility of subsequent peripheral functionalization, make them good candidates for many nanotechnology applications, such as nanotherapeutic formulations, innovative functional materials, or models to study interactions and effects of multivalency.

## Supplementary Material



## Data Availability

FIDs of nuclear magnetic resonance spectra, molecular modeling data and their associated meta data are available at Zenodo repository at doi.org/10.5281/zenodo.17340560.

## Notes

Detailed synthesis and characterization of compounds and recycling of modules AB_3_ and AB_6_, DLS, diffusion NMR, A4F, and molecular modeling (PDF)
