# Supplementary material for: A Modular Synthetic Strategy toward Fast-Growing Poly(amide-carbosilane) Dendrimers Based on Click Chemistry and Organic Solvent Nanofiltration
Source: ACS Polym Au. 2026 Jan 16;6(1):454–67. doi: 10.1021/acspolymersau.5c00171 (PMC12903430; doi:10.1021/acspolymersau.5c00171)
Supplement: Supplementary file 1 [file lg5c00171_si_001.pdf]

Supplementary Information

A modular synthetic strategy towards fast-growing poly(amide-carbosilane) dendrimers based on click chemistry and organic solvent nanofiltration

Antonín Edr[1], Martin Konhefr[1], Alena Krupková[1], Lucie Červenková Šťastná[1], Jana Bernášková[1], Olga Kočková[2], Věra Vlčková[2], Zuzana Walterová[2], Lívia Kanizsová[2], Jan Lang[5], Jakub Žváček[1,4], Marek Malý[3], Tomáš Strašák[1]*

[1] The Czech Academy of Sciences, Institute of Chemical Process Fundamentals, Rozvojová 1/135, 165 00 Prague, Czech Republic

[2] The Czech Academy of Sciences, Institute of Macromolecular Chemistry, Heyrovského nám. 2, 162 00 Prague 6, Czech Republic

[3]Department of Physics, University of Jan Evangelista Purkyně in Ústí nad Labem, Pasteurova 15, 400 96 Ústí nad Labem, Czech Republic

[4] Department of Organic Chemistry, University of Chemistry and Technology, Technická 5, 166 28 Prague 6, Czech Republic

[5] Department of Low Temperature Physics, Faculty of Mathematics and Physics, Charles University, V

Table of Contents:

1. Detailed synthesis and characterization of compounds 2
2. MALDI-TOF and ESI-QTOF MS analysis 19
3. Recycling of modules AB_3 and AB_6 27
4. Dynamic light scattering (DLS) 29
5. Diffusion NMR 37
6. Asymmetric Flow Field-Flow Fractionation (A4F) 38
7. Molecular modelling 48
8. References 57
9. NMR spectra 59

1. Detailed synthesis and characterization of compounds

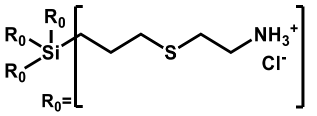

Dendritic core G_0-N. Cysteamine hydrochloride (2.11 g, 18.6 mmol, 4.00 eq.) and DMPA (0.18 g, 0.69 mmol, 0.15 eq.) were put into a 40 mL vial, MeOH (25 mL) and tetraallylsilane (892 mg, 4.64 mmol, 1.00 eq.) were added and the reaction mixture was purged with argon during stirring for 10 min. The reaction mixture was irradiated in the TEC reaction (see General procedure GP2) for 15 min while stirring. The solvent was removed on a rotary evaporator to afford G_0-N (3.00 g, quantitative yield, beige powder, further used without any purification with DMPA). **^{1}H NMR** (400 MHz, DMSO-d_6, ^{1}H-^{1}H COSY): δ 8.22 (br s, 12H, NH_3), 2.93 (t, J = 7.4 Hz, 8H, CH_2N), 2.74 (t, J = 7.4 Hz, 8H, CH_2CH$_2$N), 2.56 (t, J = 7.1 Hz, 8H, CH_2(CH$_2$)$_2$Si), 1.54–1.47 (m, 8H, SiCH$_2$CH_2), 0.63–0.59 (m, 8H, SiCH_2). **^{13}C {^{1}H} NMR** (101 MHz, DMSO-d_6, ^{1}H-^{13}C HSQC, ^{1}H-^{13}C HMBC): δ 38.6 (NCH$_2$), 34.4 (Si(CH$_2$)$_2$CH$_2$), 27.8 (NCH$_2$CH$_2$), 23.7 (SiCH$_2$CH$_2$), 11.1 (SiCH$_2$). **^{29}Si {^{1}H} NMR** (79 MHz, DMSO-d_6): δ 3.66. **HRMS** (ESI$^+$): *m/z* (monoisotopic) calcd. for $[C_{20}H_{49}N_4S_4Si]^+$ 501.2604, found 501.2590 [M-3HCl-Cl]$^+$.

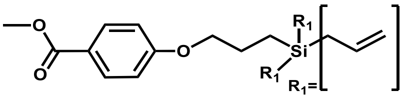

Methyl 4-(3-(triallylsilyl)propoxy)benzoate, **MeO-AB$_3$ module.** Methyl 4-hydroxybenzoate (5.00 g, 32.9 mmol) and calcinated K_2CO_3 (9.08 g, 65.7 mmol) were dissolved in MeCN (375 mL) and triallyl(3-iodopropyl)silane (9.46 g, 29.6 mmol) was added. The reaction mixture was stirred at reflux for 20 h. Then, the solution was filtered through a filter paper (washed with DCM) and the solvents were removed on a rotary evaporator. The obtained mixture was separated on a short column of silica gel (50 g) in eluent pentane/Et$_2$O 10:1 (the product came as the first fraction). The solvents were removed giving yellow-orange viscous liquid (9.74 g, 95 %). **^{1}H NMR** (400 MHz, CDCl$_3$, ^{1}H-^{1}H COSY): δ 7.98 (m, 2H, CH_{Ph}C$_{q(Ph)}$CONH), 6.89 (m, 2H, CH_{Ph}), 5.80 (ddt, J = 16.9, 10.1, 8.1 Hz, 3H, CHCH$_2$), 4.94–4.87 (m, 6H, CHCH_2), 3.95 (t, J = 6.7 Hz, 2H, OCH_2), 3.88 (s, 3H, *Me*), 1.86–1.81 (m, 2H, OCH$_2$CH_2), 1.63 (dt, J = 8.1, 1.2 Hz, 6H, SiCH_2CH), 0.74–0.69 (m, 2H, SiCH_2CH$_2$). **^{13}C {^{1}H} NMR** (101 MHz, CDCl$_3$, ^{1}H-^{13}C HSQC, ^{1}H-^{13}C HMBC): δ 167.0 (*C*OOMe), 162.9 ($C_{q(Ph)}$OCH$_2$), 134.2 (*C*HCH$_2$), 131.7 (HNCOC$_{q(Ph)}$$CH_{Ph}$), 122.5 ($C_{q(Ph)}$), 114.2 ($CH_{Ph}$), 114.0 (CH$CH_2$), 70.7 (O$CH_2$), 52.0 (*Me*), 23.4 (OCH$_2$$CH_2$), 19.6 (Si$CH_2$CH), 7.5 (Si$CH_2CH_2$). **^{29}Si {^{1}H} NMR** (79 MHz, CDCl$_3$): δ –0.11. **HRMS** (ESI$^+$): *m/z* (monoisotopic) calcd. for $[C_{20}H_{29}O_3Si]^+$ 345.1880, found 345.1875 [M+H]$^+$; calcd. for $[C_{20}H_{28}NaO_3Si]^+$ 367.1700, found 367.1700 [M+Na]$^+$; calcd. for $[C_{40}H_{56}NaO_6Si_2]^+$ 711.3508, found 711.3488 [2M+Na]$^+$.

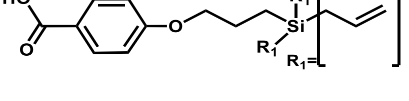

4-(3-(Triallylsilyl)propoxy)benzoic acid, **AB$_3$ module.** Methyl 4-(3-(triallylsilyl)propoxy)benzoate (9.74 g, 28.3 mmol) and KOH (7.93 g, 141 mmol) were dissolved in MeOH (190 mL) and the reaction mixture was stirred at reflux for 24 h. The solvent was reduced by half, DCM was added (500 mL) and the solution was washed (2 × 200 mL 1M HCl, 2 × 400 mL 3% aq. sol. of NaCl) and dried by anhydrous MgSO$_4$. The solvents were removed giving AB$_3$ module as a white amorphous solid (9.32 g, 98 %). **^{1}H NMR** (400 MHz, CDCl$_3$, ^{1}H-^{1}H COSY): δ 8.06 (m, 2H, CH_{Ph}C$_{q(Ph)}$CONH), 6.93 (m, 2H, CH_{Ph}), 5.80 (ddt, J = 16.9, 10.1, 8.1 Hz, 3H, CHCH$_2$), 4.94–4.87 (m, 6H, CHCH_2), 3.98 (t, J = 6.7 Hz, 2H, OCH_2), 1.87–1.83 (m, 2H, OCH$_2$CH_2), 1.64 (dt, J = 8.1, 1.2 Hz, 6H, SiCH_2CH), 0.75–0.71 (m, 2H, SiCH_2CH$_2$). **^{13}C {^{1}H} NMR**

(101 MHz, $CDCl_3$, ^{1}H-^{13}C HSQC, ^{1}H-^{13}C HMBC): δ 171.9 (*C*OOH), 163.7 ($C_{q(Ph)}OCH_2$), 134.2 (*C*HCH$_2$), 132.5 (HNCOC$_{q(Ph)}$*C*H$_{Ph}$), 121.6 ($C_{q(Ph)}$), 114.3 (*C*H$_{Ph}$), 114.1 (CH*C*H$_2$), 70.8 (O*C*H$_2$), 23.4 (OCH$_2$*C*H$_2$), 19.7 (Si*C*H$_2$CH), 7.6 (Si*C*H$_2$). **^{29}Si {^{1}H} NMR** (79 MHz, $CDCl_3$): δ –0.11. **HRMS** (ESI$^-$): *m/z* (monoisotopic) calcd. for $[C_{19}H_{25}O_3Si]^-$ 329.1578, found 329.1568 $[M-H]^-$; calcd. for $[C_{38}H_{51}O_6Si_2]^+$ 659.3230, found 659.3177 $[2M-H]^-$.

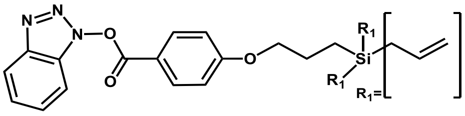

Benzotriazolyl 4-(3-(triallylsilyl)propoxy)benzoate, activated **BtO-AB$_3$ module.** 4-(3-(triallylsilyl)propoxy)benzoic acid (2.49 g, 7.54 mmol, 1.10 eq. and more per amide bond) was activated as BtO-AB$_3$ module in reaction with TBTU (2.42 g, 7.54 mmol, 1.10 eq. and more per amide bond) and DIPEA (2.66 g, 20.63 mmol, 3.00 eq. per amide bond) during the synthesis of allylic dendrimers according to *General procedure GP1*. During the OSN, several filtrates containing solely the activated BtO-AB$_3$ module were collected, solvent was spontaneously evaporated under ambient air giving off-white needles in various yields depending on the excess in the reaction, 0.10 eq. and more per amide bond. The needles without further purification could be reused for the next synthesis of allylic dendrimer without further addition of TBTU (see *Recycling of modules AB$_3$ and AB$_6$* below). **^{1}H NMR** (400 MHz, $CDCl_3$, ^{1}H-^{1}H COSY): δ 8.22 (m, 2H, C*H*$_{Ph}$), 8.09 (dd, *J* = 8.4, 1.1 Hz, 1H, C*H*$_{BtO}$), 7.54 (dd, *J* = 8.4, 6.7 Hz, 1H, C*H*$_{BtO}$), 7.47 (d, *J* = 8.1 Hz, 1H, C*H*$_{BtO}$), 7.43 (ddd, *J* = 8.1, 6.7, 1.1 Hz, 1H, C*H*$_{BtO}$), 7.04 (m, 2H, C*H*$_{Ph}$), 5.81 (ddt, *J* = 16.4, 10.1, 8.0 Hz, 3H, C*H*CH$_2$), 4.95–4.87 (m, 6H, CHC*H*$_2$), 4.03 (t, *J* = 6.6 Hz, 2H, OC*H*$_2$), 1.92–1.85 (m, 2H, OCH$_2$C*H*$_2$), 1.65 (dt, *J* = 8.1, 1.2 Hz, 6H, SiC*H*$_2$CH), 0.77–0.73 (m, 2H, O(CH$_2$)$_2$C*H*$_2$Si). **^{13}C {^{1}H} NMR** (101 MHz, $CDCl_3$, ^{1}H-^{13}C HSQC, ^{1}H-^{13}C HMBC): δ 165.0 ($C_{q(Ph)}OCH_2$), 162.5 (*C*OO), 143.7 (C_qN_{BtO}), 134.2 ($C_{q(Ph)}$), 134.1 (*C*HCH$_2$), 133.2 (*C*H$_{Ph}$), 129.0 (C_qN_{BtO}), 128.8 (*C*H$_{BtO}$), 124.9 (*C*H$_{BtO}$), 120.6 (*C*H$_{BtO}$), 115.1 (*C*H$_{Ph}$), 114.1 (CH*C*H$_2$), 108.6 (*C*H$_{BtO}$), 71.0 (O*C*H$_2$), 23.3 (OCH$_2$*C*H$_2$), 19.6 (Si*C*H$_2$CH), 7.6 (Si*C*H$_2$CH$_2$). **HRMS** (ESI$^+$): *m/z* (monoisotopic) calcd. for $[C_{25}H_{29}N_3NaO_3Si]^+$ 470.1870, found 470.1830 $[M+Na]^+$.

1st generation dendrimers

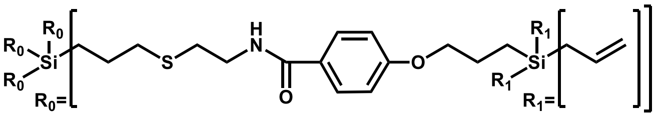

G$_1$-3-A. Dendrimer G$_1$-3-A was prepared from 1.11 g (1.71 mmol) of G$_0$-N according to the general procedure for amidic coupling (see General procedure GP1). The product was purified by OSN using a 1kDa membrane in DCM/MeOH mixture, starting at 2:1 ratio and gradually increasing the polarity up to 1:2 so as to maintain the homogeneity of the solution, and obtained as a brownish viscous substance (2.67 g, 89 %). **^{1}H NMR** (400 MHz, $CDCl_3$, ^{1}H-^{1}H COSY): δ 7.75 (m, 8H, C*H*$_{Ph}$C$_{q(Ph)}$CONH), 6.88 (m, 8H, C*H*$_{Ph}$C$_{q(Ph)}$OCH$_2$), 6.78 (t, *J* = 5.7 Hz, 4H, N*H*), 5.79 (ddt, *J* = 16.5, 10.1, 8.1 Hz, 12H, C*H*CH$_2$), 4.91–4.86 (m, 24H, CHC*H*$_2$), 3.92 (t, *J* = 6.7 Hz, 8H, OC*H*$_2$), 3.59 (td, *J* = 6.6, 5.7 Hz, 8H, HNC*H*$_2$), 2.73 (t, *J* = 6.6 Hz, 8H, HNCH$_2$C*H*$_2$), 2.52 (t, *J* = 7.2 Hz, 8H, Si(CH$_2$)$_2$C*H*$_2$S), 1.86–1.78 (m, 8H, OCH$_2$C*H*$_2$), 1.62 (dt, *J* = 8.1, 1.2 Hz, 24H, SiC*H*$_2$CH), 1.59–1.51 (m, 8H, SiCH$_2$C*H*$_2$), 0.72–0.68 (m, 8H, O(CH$_2$)$_2$C*H*$_2$), 0.63–0.59 (m, 8H, SiC*H*$_2$). **^{13}C {^{1}H} NMR** (101 MHz, $CDCl_3$, ^{1}H-^{13}C HSQC, ^{1}H-^{13}C HMBC): δ 167.1 (HN*C*O), 161.8 ($C_{q(Ph)}OCH_2$), 134.2 (*C*HCH$_2$), 128.9 (HNCOC$_{q(Ph)}$*C*H$_{Ph}$), 126.5 (HNCO*C*$_{q(Ph)}$), 114.3 (*C*H$_{Ph}$C$_{q(Ph)}$OCH$_2$), 114.0 (CH*C*H$_2$), 70.7 (O*C*H$_2$), 39.2 (HN*C*H$_2$), 35.6 (Si(CH$_2$)$_2$*C*H$_2$S), 31.9 (*C*H$_2$CH$_2$NH), 24.4 (SiCH$_2$*C*H$_2$CH$_2$S), 23.4 (OCH$_2$*C*H$_2$), 19.6 (Si*C*H$_2$CH), 11.8 (Si*C*H$_2$(CH$_2$)$_2$S), 7.6 (*C*H$_2$(CH$_2$)$_2$O). **^{29}Si {^{1}H} NMR** (79 MHz, $CDCl_3$): δ 3.65 (Si^0), –0.10 (Si^1). **HRMS** (ESI$^+$, CH_3OH): *m/z* (monoisotopic) calcd. for $[C_{96}H_{145}N_4O_8S_4Si_5]^+$ 1749.8786; found 1749.8774 $[M+H]^+$; calcd. for $[C_{192}H_{289}KN_8O_{16}S_8Si_{10}]^{2+}$ 1768.8566, found $[2M+H+K]^{2+}$; calcd. for $[C_{96}H_{144}N_4NaO_8S_4Si_5]^+$ 1771.8606, found 1771.8557 (overlap) $[M+Na]^+$; calcd. for $[C_{96}H_{146}N_4O_8S_4Si_5]^{2+}$

875.4429, found 875.4429 [M+2H]$^{2+}$; calcd. for $[C_{96}H_{145}KN_4O_8S_4Si_5]^{2+}$ 894.4209, found 894.4164 [2M+H+K]$^{2+}$. **MALDI-TOF MS** (DCTB, Na^+): *m/z* (monoisotopic) calcd. for $[C_{96}H_{144}N_4NaO_8S_4Si_5]^+$ 1771.86, found 1771.89 [M+Na]$^+$.

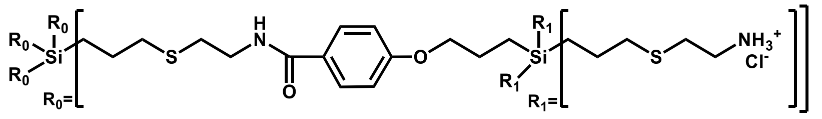

G_1-3-N. Dendrimer G_1-3-N was prepared from 1.34 g (0.765 mmol) of dendrimer G_1-3-A according to General procedure GP2. The product was purified by OSN using 3kDa membrane in MeOH followed by lyophilization and obtained as a white powder (2.16 g, 91 %). **^{1}H NMR** (400 MHz, DMSO-d_6, ^{1}H-^{1}H COSY): δ 8.57 (t, *J* = 5.7 Hz, 4H, N*H*), 8.23 (br s, 36H, NH_3^+), 7.83 (m, 8H, $CH_{Ph}C_{q(Ph)}$CONH), 6.95 (m, 8H, $CH_{Ph}C_{q(Ph)}OCH_2$), 3.97 (t, *J* = 5.8 Hz, 8H, OCH_2), 3.38 (td, *J* = 7.3, 5.7 Hz, 8H, $HNCH_2$), 2.94 (t, *J* = 7.4 Hz, 24H, $CH_2NH_3^+$), 2.75 (t, *J* = 7.4 Hz, 24H, $CH_2CH_2NH_3^+$), 2.63 (t, *J* = 7.3 Hz, 8H, SCH_2CH_2NH), 2.55 (t, *J* = 7.0 Hz, 32H, $Si(CH_2)_2CH_2S$), 1.72–1.68 (m, 8H, OCH_2CH_2), 1.57–1.49 (m, 32H, $SiCH_2CH_2$), 0.66–0.60 (m, 40H, $SiCH_2$). **^{13}C {^{1}H} NMR** (101 MHz, DMSO-d_6, ^{1}H-^{13}C HSQC, ^{1}H-^{13}C HMBC): δ 165.6 (HNCO), 160.9 ($C_{q(Ph)}OCH_2$), 129.0 ($HNCOC_{q(Ph)}CH_{Ph}$), 126.4 ($HNCOC_{q(Ph)}$), 113.9 ($CH_{Ph}C_{q(Ph)}OCH_2$), 70.2 (OCH_2), from HSQC 39.4 ($NHCH_2$), 38.6 ($CH_2NH_3^+$), 34.8 ($Si^0(CH_2)_2CH_2S$), 34.4 ($Si^1(CH_2)_2CH_2S$), 30.5 (SCH_2CH_2NH), 27.8 ($SCH_2CH_2NH_3^+$), 23.8 ($Si^0CH_2CH_2$), 23.7 ($Si^1CH_2CH_2$), 23.2 (OCH_2CH_2), 11.2 (Si^0CH_2), 11.1 ($Si^1CH_2(CH_2)_2S$), 7.7 ($O(CH_2)_2CH_2Si^1$). **^{29}Si {^{1}H} NMR** (79 MHz, DMSO-d_6): δ 4.05 (Si^1), 3.58 (Si^0). **HRMS** (ESI$^+$, CH_3OH) *m/z* (monoisotopic): calcd. for $[C_{120}H_{229}N_{16}O_8S_{16}Si_5]^+$ 2674.2376, found 2674.2255 [M-12HCl+H]$^+$; calcd. for $[C_{120}H_{230}N_{16}O_8S_{16}Si_5]^{2+}$ 1337.6225, found 1337.6234 [M-12HCl+2H]$^{2+}$; calcd. for $[C_{120}H_{231}N_{16}O_8S_{16}Si_5]^{3+}$ 892.0841, found 892.0843 [M-12HCl+3H]$^{3+}$; calcd. for $[C_{120}H_{232}N_{16}O_8S_{16}Si_5]^{4+}$ 669.3149, found 669.3150 [M-12HCl+4H]$^{4+}$; calcd. for $[C_{120}H_{233}N_{16}O_8S_{16}Si_5]^{5+}$ 535.6534, found 535.6536 [M-12HCl+5H]$^{5+}$; calcd. for $[C_{120}H_{234}N_{16}O_8S_{16}Si_5]^{6+}$ 446.5457, found 446.5456 [M-12HCl+6H]$^{6+}$. **MALDI-TOF MS** (DHB, Na^+) *m/z* (monoisotopic): calcd. for $[C_{120}H_{229}N_{16}O_8S_{16}Si_5]^+$ 2674.24, found 2674.20 [M+H]$^+$; calcd. for $[C_{120}H_{228}N_{16}NaO_8S_{16}Si_5]^+$ 2696.22, found 2696.19 [M+Na]$^+$.

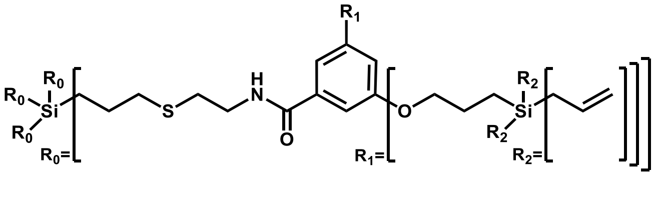

G_1-6-A. Dendrimer G_1-6-A was prepared from 748 mg (1.16 mmol) of G_0-N according to General procedure GP1. The product was purified by OSN using 3kDa membrane in DCM/MeOH mixture, starting at 2:1 ratio and gradually increasing the polarity up to 1:2 so as to maintain the homogeneity of the solution, and obtained as a brownish viscous substance (2.86 g, 95 %). **^{1}H NMR** (400 MHz, $CDCl_3$, ^{1}H-^{1}H COSY): δ 6.89 (d, *J* = 2.3 Hz, 8H, CH_{Ph}), 6.70 (t, *J* = 5.7 Hz, 4H, N*H*), 6.54 (t, *J* = 2.3 Hz, 4H, CH_{Ph}), 5.79 (ddt, *J* = 16.4, 10.0, 8.1 Hz, 24H, $CHCH_2$), 4.93–4.86 (m, 48H, $CHCH_2$), 3.89 (t, *J* = 6.6 Hz, 16H, OCH_2), 3.59 (td, *J* = 6.6, 5.7 Hz, 8H, $HNCH_2$), 2.73 (t, *J* = 6.6 Hz, 8H, $HNCH_2CH_2$), 2.54 (t, *J* = 7.2 Hz, 8H, $SiCH_2CH_2CH_2S$), 1.81–1.77 (m, 16H, OCH_2CH_2), 1.62 (dt, *J* = 8.1, 1.3 Hz, 48H, $SiCH_2CH$), 1.63–1.61 (m, 8H, $SiCH_2CH_2$), 0.71–0.66 (m, 24H, $SiCH_2$). **^{13}C {^{1}H} NMR** (101 MHz, $CDCl_3$, ^{1}H-^{13}C HSQC, ^{1}H-^{13}C HMBC): δ 167.4 (HNCO), 160.4 ($C_{q(Ph)}OCH_2$), 136.6 ($HNCOC_{q(Ph)}$), 134.2 ($CHCH_2$), 114.0 ($CHCH_2$), 105.6 ($HNCOC_{q(Ph)}CH_{Ph}$), 104.5 (CH_{Ph}), 70.9 (OCH_2), 39.3 ($HNCH_2$), 35.6 ($Si(CH_2)_2CH_2S$), 31.8 (CH_2CH_2NH), 24.4 ($SiCH_2CH_2CH_2S$), 23.5 (OCH_2CH_2), 19.6 ($SiCH_2CH$), 11.9 ($SiCH_2(CH_2)_2S$), 7.7 ($CH_2(CH_2)_2O$). **^{29}Si {^{1}H} NMR** (79 MHz, $CDCl_3$): δ 3.68 (Si^0), –0.11 (Si^1). **MALDI-TOF MS** (DHB, Na^+): *m/z* (monoisotopic) calcd. for $[C_{144}H_{224}N_4NaO_{12}S_4Si_9]^+$ 2604.37, found 2604.32 [M+Na]$^+$.

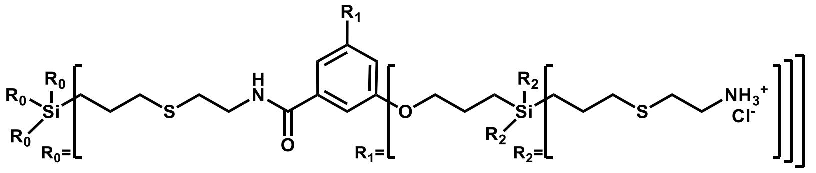

G_1-6-N. Dendrimer G_1-6-N was prepared from 1.44 g (0.557 mmol) of dendrimer G_1-6-A according to General procedure GP2. The product was purified by OSN using 3kDa membrane in MeOH followed by lyophilization and obtained as off-white powder (2.92 g, 98 %). **^{1}H NMR** (400 MHz, DMSO-d_6, ^{1}H-^{1}H COSY): δ 8.79 (br s, 4H, N*H*), 8.30 (br s, 72H, NH_3^+), 7.03 (br s, 8H, CH_{Ph}), 6.58 (br s, 4H, CH_{Ph}), 3.94 (t, *J* = 6.1 Hz, 16H, OCH_2), 3.37 (br s, 8H, CH_2NH), 2.94 (t, *J* = 7.3 Hz, 48H, $CH_2NH_3^+$), 2.76 (t, *J* = 7.3 Hz, 48H, $SCH_2CH_2NH_3^+$), 2.63 (t, *J* = 6.9 Hz, 8H, SCH_2CH_2NH), 2.55 (t, *J* = 6.4 Hz, 56H, $Si(CH_2)_2CH_2S$), 1.72–1.64 (m, 16H, OCH_2CH_2), 1.55–1.51 (m, 56H, $SiCH_2CH_2$), 0.66–0.62 (m, 64H, $SiCH_2$). **^{13}C {^{1}H} NMR** (101 MHz, DMSO-d_6, ^{1}H-^{13}C HSQC, ^{1}H-^{13}C HMBC): δ 165.6 (NHCO), 159.6 ($C_{q(Ph)}OCH_2$), 136.2 ($HNCOC_{q(Ph)}$), 105.6 ($HNCOC_{q(Ph)}CH_{Ph}$), 103.8 (CH_{Ph}), 70.4 (OCH_2), from HSQC 39.4 (CH_2NH), 38.7 ($CH_2NH_3^+$), 34.8 ($Si^0(CH_2)_2CH_2S$), 34.5 ($Si^1(CH_2)_2CH_2S$), 30.4 (SCH_2CH_2NH), 27.8 ($SCH_2CH_2NH_3^+$), 23.8 ($Si^0CH_2CH_2$), 23.7 ($Si^1CH_2CH_2$), 23.3 (OCH_2CH_2), 11.3 (Si^0CH_2), 11.2 ($Si^1CH_2(CH_2)_2S$), 7.8 ($O(CH_2)_2CH_2Si^1$). **^{29}Si {^{1}H} NMR** (79 MHz, DMSO-d_6): δ 4.02 (Si^1), 3.60 (Si^0). **HRMS** (ESI$^+$): *m/z* (100 % centroid) calcd. for $[C_{192}H_{394}N_{28}O_{12}S_{28}Si_9]^{2+}$ 2218.5595, found 2218.5551 (96 %) $[M\text{-}24HCl+2H]^{2+}$; calcd. for $[C_{192}H_{395}N_{28}O_{12}S_{28}Si_9]^{3+}$ 1479.3754, found 1479.3750 $[M\text{-}24HCl+3H]^{3+}$; calcd. for $[C_{192}H_{396}N_{28}O_{12}S_{28}Si_9]^{4+}$ 1109.7834, found 1109.7828 $[M\text{-}24HCl+4H]^{4+}$; calcd. for $[C_{192}H_{397}N_{28}O_{12}S_{28}Si_9]^{5+}$ 888.0282, found 888.0278 $[M\text{-}24HCl+5H]^{5+}$; calcd. for $[C_{192}H_{398}N_{28}O_{12}S_{28}Si_9]^{6+}$ 740.1914, found 740.1912 $[M\text{-}24HCl+6H]^{6+}$; calcd. for $[C_{192}H_{399}N_{28}O_{12}S_{28}Si_9]^{7+}$ 634.5936, found 634.5937 $[M\text{-}24HCl+7H]^{7+}$; calcd. for $[C_{192}H_{400}N_{28}O_{12}S_{28}Si_9]^{8+}$ 555.3953, found 555.3954 $[M\text{-}24HCl+8H]^{8+}$. **MALDI-TOF MS** (DHB, Na^+): *m/z* (100 % centroid) calcd. for $[C_{192}H_{393}N_{28}O_{12}S_{28}Si_9]^+$ 4436.11, found 4436.22 $[M+H]^+$; calcd. for $[C_{192}H_{392}N_{28}NaO_{12}S_{28}Si_9]^+$ 4458.09, found 4458.17 $[M+Na]^+$.

2nd generation dendrimers

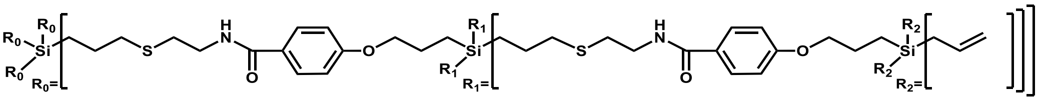

G_2-3-3-A. Dendrimer G_2-3-3-A was prepared from 1.17 g (0.376 mmol) of G_1-3-N according to General procedure GP1. The product was purified by OSN using 3kDa membrane in DCM/MeOH mixture, starting at 1:1 ratio and gradually increasing the polarity up to 1:2 so as to maintain the homogeneity of the solution, and obtained as a brownish viscous substance (2.26 g, 93 %). **^{1}H NMR** (400 MHz, $CDCl_3$, ^{1}H-^{1}H COSY): δ 7.75 (m, 32H, $CH_{Ph}C_{q(Ph)}CONH$), 7.03 (t, *J* = 5.7 Hz, 4H, N*H*), 6.87 (m, 32H, $CH_{Ph}C_{q(Ph)}OCH_2$), 6.85 (t, *J* = 5.7 Hz, 12H, N*H*), 5.78 (ddt, *J* = 16.5, 10.1, 8.1 Hz, 36H, $CHCH_2$), 4.92–4.85 (m, 72H, $CHCH_2$), 3.91 (t, *J* = 6.6 Hz, 32H, OCH_2), 3.57 (td, *J* = 6.6, 5.7 Hz, 32H, CH_2NH), 2.71 (t, *J* = 6.6 Hz, 32H, SCH_2CH_2NH), 2.52 (t, *J* = 7.2 Hz, 32H, $SCH_2(CH_2)_2Si$), 1.85–1.77 (m, 24H, $OCH_2CH_2CH_2Si^2$), 1.76–1.70 (m, 8H, $OCH_2CH_2CH_2Si^1$), 1.62 (dt, *J* = 8.1, 1.2 Hz, 72H, $SiCH_2CH$), 1.58–1.49 (m, 32H, $SiCH_2CH_2CH_2S$), 0.72–0.67 (m, 24H, $O(CH_2)_2CH_2Si^2$), 0.66–0.60 (m, 8H, $O(CH_2)_2CH_2Si^1$, 24H, $Si^1CH_2(CH_2)_2S$), 0.58–0.56 (m, 8H, Si^0CH_2). **^{13}C {^{1}H} NMR** (101 MHz, $CDCl_3$, ^{1}H-^{13}C HSQC, ^{1}H-^{13}C HMBC): δ 167.2 (HN^0CO), 167.1 (HN^1CO), 161.8 ($C_{q(Ph)}O^1CH_2$), 161.6 ($C_{q(Ph)}O^0CH_2$), 134.2 ($CHCH_2$), 129.0 ($HN^0COC_{q(Ph)}CH_{Ph}$), 128.9 ($HN^1COC_{q(Ph)}CH_{Ph}$), 126.7 ($HN^0COC_{q(Ph)}$), 126.5 ($HN^1COC_{q(Ph)}$), 114.3 ($CH_{Ph}CO^{0/1}CH_2$), 114.0 ($CHCH_2$), 70.7 (O^1CH_2), 70.6 (O^0CH_2), 39.3 (CH_2N^0H), 39.2 (CH_2N^1H), 35.6 ($Si(CH_2)_2CH_2S$), 31.9 (SCH_2CH_2NH), 24.4 ($SiCH_2CH_2CH_2S$), 23.7 ($O^0CH_2CH_2$), 23.4 ($O^1CH_2CH_2$), 19.6 ($SiCH_2CH$), 11.8 ($SiCH_2(CH_2)_2S$), 8.2 ($O^0(CH_2)_2CH_2$), 7.6 ($O^1(CH_2)_2CH_2$). **^{29}Si {^{1}H} NMR** (79 MHz, $CDCl_3$): δ 4.05 (Si^1), 3.61 (Si^0), –0.11 (Si^2).

MALDI-TOF MS (DCTB, Na^+): *m/z* (100 % centroid) calcd. for $[C_{348}H_{516}N_{16}NaO_{32}S_{16}Si_{17}]^+$ 6449.08, found 6448.94 $[M+Na]^+$.

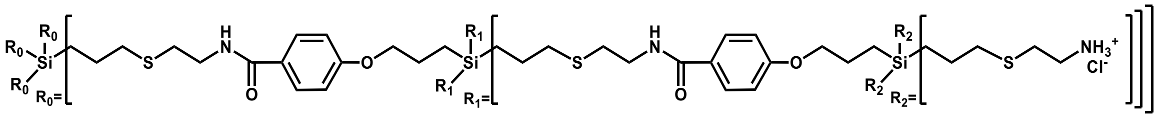

G_2-3-3-N. Dendrimer G_2-3-3-N was prepared from 1.50 g (0.233 mmol) of dendrimer G_2-3-3-A according to General procedure GP2. The product was purified by OSN using 3kDa membrane in MeOH followed by lyophilization and obtained as a white powder (2.41 g, 98 %). **^{1}H NMR** (400 MHz, DMSO-d_6, ^{1}H-^{1}H COSY): δ 8.59 (t, *J* = 6.0 Hz, 16H, N*H*), 8.25 (br s, 108H, NH_3^+), 7.84 (m, 32H, $CH_{Ph}C_{q(Ph)}$CONH), 6.94 (m, 32H, $CH_{Ph}C_{q(Ph)}OCH_2$), 3.95 (t, *J* = 6.3 Hz, 32H, OCH_2), 3.41–3.35 overlapped with H_2O (32H, CH_2NH), 2.94 (t, *J* =7.3 Hz, 72H, $CH_2NH_3^+$), 2.75 (t, *J* = 7.3 Hz, 72H, $CH_2CH_2NH_3^+$), 2.64 (t, *J* = 6.7 Hz, 32H, SCH_2CH_2NH), 2.55 (t, *J* = 7.0 Hz, 104H, $SCH_2(CH_2)_2$Si), 1.71–1.66 (m, 32H, OCH_2CH_2), 1.56–1.48 (m, 104H, $SiCH_2CH_2CH_2S$), 0.66–0.59 (m, 136H, $SiCH_2$). **^{13}C {^{1}H} NMR** (101 MHz, DMSO-d_6, ^{1}H-^{13}C HSQC, ^{1}H-^{13}C HMBC): δ 165.6 (HN*C*O), 160.9 ($C_{q(Ph)}OCH_2$), 129.1 ($HNCOC_{q(Ph)}CH_{Ph}$), 126.4 ($HNCOC_{q(Ph)}$), 113.9 ($CH_{Ph}C_{q(Ph)}OCH_2$), 70.2 (OCH_2), from HSQC 39.5 (CH_2NH), 38.6 ($CH_2NH_3^+$), 34.8 ($CH_2S(CH_2)_2$NH), 34.5 ($CH_2S(CH_2)_2NH_3^+$), 30.5 (SCH_2CH_2NH), 27.8 ($SCH_2CH_2NH_3^+$), 23.8 ($CH_2CH_2S(CH_2)_2$NH), 23.7 ($CH_2CH_2S(CH_2)_2NH_3^+$), 23.2 (OCH_2CH_2), 11.2 ($CH_2(CH)_2S(CH_2)_2$NH), 11.1 ($CH_2(CH_2)_2S(CH_2)_2NH_3^+$), 7.7 ($O(CH_2)_2CH_2$). **^{29}Si {^{1}H} NMR** (79 MHz, DMSO-d_6): δ 4.04 (Si^2), 3.99 (Si^1), 3.57 (Si^0). **HRMS** (ESI$^+$, CH_3OH): *m/z* (100 % centroid) calcd. for $[C_{420}H_{772}N_{52}O_{32}S_{52}Si_{17}]^{4+}$ 2301.7989, found 2301.7956 (81 %) $[M-36HCl+4H]^{4+}$; calcd. for $[C_{420}H_{773}N_{52}O_{32}S_{52}Si_{17}]^{5+}$ 1841.6406, found 1841.6404 (97 %) $[M-36HCl+5H]^{5+}$; calcd. for $[C_{420}H_{774}N_{52}O_{32}S_{52}Si_{17}]^{6+}$ 1534.8684, found 1534.8672 (96 %) $[M-36HCl+6H]^{6+}$; calcd. for $[C_{420}H_{775}N_{52}O_{32}S_{52}Si_{17}]^{7+}$ 1315.7454, found 1315.7449 (99 %) $[M-36HCl+7H]^{7+}$; calcd. for $[C_{420}H_{776}N_{52}O_{32}S_{52}Si_{17}]^{8+}$ 1151.4031, found 1151.4025 (94 %) $[M-36HCl+8H]^{8+}$; calcd. for $[C_{420}H_{777}N_{52}O_{32}S_{52}Si_{17}]^{9+}$ 1023.5813, found 1023.5810 (99 %) $[M-36HCl+9H]^{9+}$; calcd. for $[C_{420}H_{778}N_{52}O_{32}S_{52}Si_{17}]^{10+}$ 921.3239, found 921.3234 (92 %) $[M-36HCl+10H]^{10+}$; calcd. for $[C_{420}H_{779}N_{52}O_{32}S_{52}Si_{17}]^{11+}$ 837.6588, found 837.6588 (100 %) $[M-36HCl+11H]^{11+}$; calcd. for $[C_{420}H_{780}N_{52}O_{32}S_{52}Si_{17}]^{12+}$ 767.9378, found 767.9370 (100 %, overlap) $[M-36HCl+12H]^{12+}$; calcd. for $[C_{420}H_{781}N_{52}O_{32}S_{52}Si_{17}]^{13+}$ 708.9432, found 708.9418 (96 %) $[M-36HCl+13H]^{13+}$. **MALDI-TOF MS** (DHB, Na^+): *m/z* (avg. mass) calcd. for $[C_{420}H_{769}N_{52}O_{32}S_{52}Si_{17}]^+$ 9204.5, found 9204.9 $[M+H]^+$.

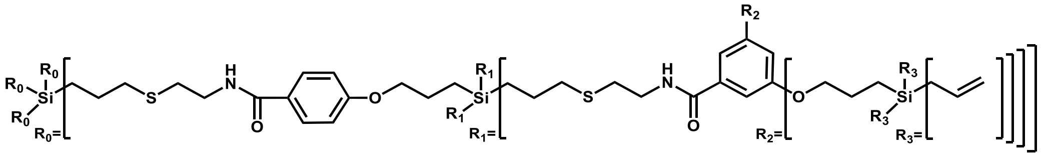

G_2-3-6-A. Dendrimer G_2-3-6-A was prepared from 250 mg (80.9 µmol) of G_1-3-N according to General procedure GP1. The product was purified by OSN using 3kDa membrane in DCM/MeOH mixture 1:1 and obtained as a brownish viscous substance (665 mg, 92 %). **^{1}H NMR** (400 MHz, $CDCl_3$, ^{1}H-^{1}H COSY): δ 7.75 (m, 8H, $HNCOC_{q(Ph)}CH_{Ph}$), 6.89 (d, *J* = 2.3 Hz, 24H, $HNCOC_{q(Ph)}CH_{Ph}$), 6.88 (m, 8H, CH_{Ph}), overlapped 6.87 (4H, N*H*), 6.77 (t, *J* = 5.7 Hz, 12H, N*H*), 6.53 (t, *J* = 2.3 Hz, 12H, CH_{Ph}), 5.78 (ddt, *J* = 16.5, 10.1, 8.1 Hz, 72H, C*H*CH_2), 4.92–4.86 (m, 144H, $CHCH_2$), 3.96–3.88 (t, *J* = 6.7 Hz, 56H, OCH_2), 3.58 (td, *J* = 6.6, 5.7 Hz, 32H, CH_2NH), 2.72 (t, *J* = 6.6 Hz, 32H, SCH_2CH_2NH), 2.54 (t, *J* = 7.1 Hz, 32H, $SCH_2(CH_2)_2$Si), 1.82–1.74 (m, 48H, $OCH_2CH_2CH_2Si^2$), 1.64–1.54 (m, 8H, ($OCH_2CH_2CH_2Si^1$), 1.61 (dt, *J* = 8.1, 1.1 Hz, 144H, $SiCH_2$CH), 0.70–0.64 (m, 88H, $SiCH_2$). **^{13}C {^{1}H} NMR** (101 MHz, $CDCl_3$, ^{1}H-^{13}C HSQC, ^{1}H-^{13}C HMBC): δ 167.4 ($HNCOC_{q(Ph)}CH_{Ph}C_{q(Ph)}$), 167.2 ($HNCOC_{q(Ph)}(CH_{Ph})_2$), 161.7 ($(CH_{Ph})_2C_{q(Ph)}OCH_2$), 160.4 ($C_{q(Ph)}CH_{Ph}C_{q(Ph)}OCH_2$),

136.6 (HNCO*C*$_{q(Ph)}$CH$_{Ph}$C$_{q(Ph)}$), 134.2 (*C*HCH$_2$), 129.0 (HNCOC$_{q(Ph)}$*C*H$_{Ph}$CH$_{Ph}$), 126.7 (HNCO*C*$_{q(Ph)}$(CH$_{Ph}$)$_2$), 114.4 (CH$_{Ph}$*C*H$_{Ph}$C$_{q(Ph)}$OCH$_2$), 114.0 (CH*C*H$_2$), 105.6 (HNCOC$_{q(Ph)}$*C*H$_{Ph}$C$_{q(Ph)}$), 104.5 (CH$_2$OC$_{q(Ph)}$*C*H$_{Ph}$C$_{q(Ph)}$OCH$_2$), 70.8 (O*C*H$_2$(CH$_2$)$_2$Si^2), 70.6 (O*C*H$_2$(CH$_2$)$_2$Si^1), 39.3 (*C*H$_2$NH), 35.6 (Si(CH$_2$)$_2$*C*H$_2$S), 31.9 (S^1*C*H$_2$CH$_2$NH), 31.8 (S^2*C*H$_2$CH$_2$NH), 24.4 (SiCH$_2$*C*H$_2$CH$_2$S), 23.7 (OCH$_2$*C*H$_2$CH$_2$Si^1), 23.5 (OCH$_2$*C*H$_2$CH$_2$Si^2), 19.6 (Si*C*H$_2$CH), 11.9 (Si*C*H$_2$(CH$_2$)$_2$S), 8.3 (O(CH$_2$)$_2$*C*H$_2$Si^1), 7.6 (O(CH$_2$)$_2$*C*H$_2$Si^2). **^{29}Si {^{1}H} NMR** (79 MHz, CDCl$_3$): *δ* 4.07 (Si^1), 3.62 (Si^0), –0.11 (Si^2). **MALDI-TOF MS** (DCTB, Na$^+$): *m/z* (avg. mass) calcd. for [$C_{492}H_{756}N_{16}NaO_{44}S_{16}Si_{29}$]$^+$ 8949.7 (avg. mass), found 8949.4 [M+Na]$^+$.

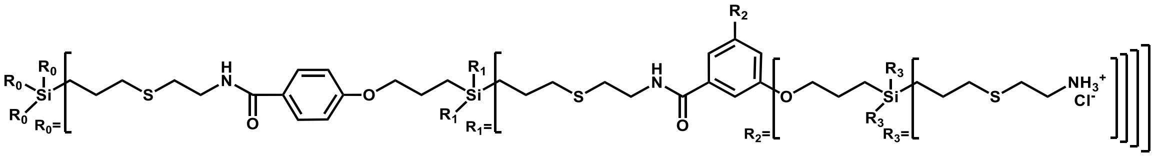

G$_2$-3-6-N. Dendrimer G$_2$-3-6-N was prepared from 460 mg (51.5 µmol) of dendrimer G$_2$-3-6-A according to General procedure GP2. The product was purified by OSN using 3kDa membrane in MeOH followed by lyophilization and obtained as off-white powder (867 mg, 98 %). **^{1}H NMR** (400 MHz, DMSO-*d*$_6$, ^{1}H-^{1}H COSY): *δ* 8.80 (br s, 12H, N*H*COC$_{q(Ph)}$CH$_{Ph}$C$_{q(Ph)}$), 8.66 (br s, 4H, N*H*COC$_{q(Ph)}$(CH$_{Ph}$)$_2$), 8.31 (br s, 216H, N*H*$_3$$^+$), 7.85 (m, 8H, NHCOC$_{q(Ph)}$C*H*$_{Ph}$), 7.04 (s, 24H, NHCOC$_{q(Ph)}$C*H*$_{Ph}$), 6.94 (m, 8H, C*H*$_{Ph}C_{q(Ph)}OCH_2$), 6.58 (s, 12H, C*H*$_{Ph}$), 3.94 (t, *J* = 7.0 Hz, 56H, OC*H*$_2$), overlapped with water (32H, C*H*$_2$NH), 2.94 (t, *J* = 7.4 Hz, 144H, C*H*$_2$NH$_3$$^+$), 2.76 (t, *J* = 7.4 Hz, 144H, C*H*$_2$CH$_2$NH$_3$$^+$), 2.65 (t, *J* = 7.1 Hz, 32H, C*H*$_2$CH$_2$NH), 2.55 (t, *J* = 7.1 Hz, 176H, SC*H*$_2$(CH$_2$)$_2$Si), 1.70–1.66 (m, 56H, OCH$_2$C*H*$_2$CH$_2$Si), 1.56–1.49 (m, 176H, SiCH$_2$C*H*$_2$CH$_2$S), 0.66–0.61 (m, 232H, SiC*H*$_2$). **^{13}C {^{1}H} NMR** (101 MHz, DMSO-*d*$_6$, ^{1}H-^{13}C HSQC, ^{1}H-^{13}C HMBC): *δ* 165.9 (HN*C*OC$_{q(Ph)}$CH$_{Ph}$C$_{q(Ph)}$), not detected or overlapped (HN*C*OC$_{q(Ph)}$(CH$_{Ph}$)$_2$), 161.0 ((CH$_{Ph}$)$_2$*C*$_{q(Ph)}$OCH$_2$), 159.6 (C$_{q(Ph)}$(CH$_{Ph}$)*C*$_{q(Ph)}$OCH$_2$), 136.2 (HNCO*C*$_{q(Ph)}$CH$_{Ph}$C$_{q(Ph)}$), 129.1 (HNCOC$_{q(Ph)}$*C*H$_{Ph}$CH$_{Ph}$), 126.3 (HNCO*C*$_{q(Ph)}$(CH$_{Ph}$)$_2$), 113.8 (CH$_{Ph}$*C*H$_{Ph}$C$_{q(Ph)}$OCH$_2$), 105.7 (HNCOC$_{q(Ph)}$*C*H$_{Ph}$C$_{q(Ph)}$), 103.9 (OC$_{q(Ph)}$*C*H$_{Ph}$C$_{q(Ph)}$O), 70.4 (O*C*H$_2$(CH$_2$)$_2$Si), from HSQC 39.5 (*C*H$_2$NH), 38.7 (*C*H$_2$NH$_3$$^+$), 34.8 (*C*H$_2$S(CH$_2$)$_2$NH), 34.5 (*C*H$_2$S(CH$_2$)$_2NH_3$$^+$), 30.4 (*C*H$_2CH_2$NH), 27.8 (*C*H$_2CH_2NH_3$$^+$), 23.7 (*C*H$_2CH_2$S(CH$_2$)$_2$NH), 23.3 (*C*H$_2CH_2$O), 11.1 (Si*C*H$_2$(CH$_2$)$_2$S), 7.8 (Si*C*H$_2$(CH$_2$)$_2$O). **^{29}Si {^{1}H} NMR** (79 MHz, DMSO-*d*$_6$): *δ* 4.02 (Si^2), not detected or overlapped (Si$^{0/1}$). **MALDI-TOF MS** (DHB, Na$^+$): *m/z* (avg. mass) calcd. for [$C_{636}H_{1261}N_{88}O_{44}S_{88}Si_{29}$]$^+$ 14482.2, found 14484.3 [M+H]$^+$.

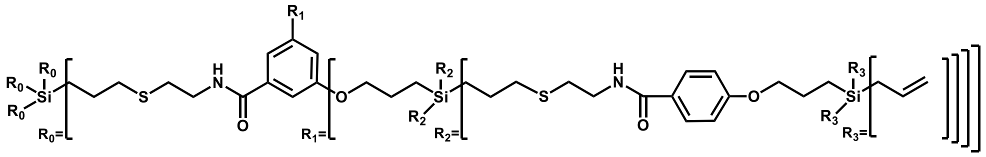

G$_2$-6-3-A. Dendrimer G$_2$-6-3-A was prepared from 318 mg (59.9 µmol) of G$_1$-6-N according to General procedure GP1. The product was purified by OSN using 3kDa membrane in DCM/MeOH mixture, starting at 2:3 ratio and gradually increasing the polarity up to 1:2 so as to maintain the homogeneity of the solution, and obtained as a brownish viscous substance (601 mg, 84 %). **^{1}H NMR** (400 MHz, CDCl$_3$, ^{1}H-^{1}H COSY): *δ* 7.76 (m, 48H, HNCOC*H*$_{Ph}$), 7.05 (t, *J* = 5.9 Hz, 24H, N*H*), 6.89 (d, *J* = 2.2 Hz, 8H, HNCOC*H*$_{Ph}$), 6.89 (t, *J* = 5.9 Hz, 4H, N*H*), 6.85 (m, 48H, C*H*$_{Ph}$), 6.53 (t, *J* = 2.2 Hz, 4H, C*H*$_{Ph}$), 5.78 (ddt, *J* = 16.4, 10.1, 8.1 Hz, 72H, C*H*CH$_2$), 4.92–4.84 (m, 144H, CHC*H*$_2$), 3.90 (t, *J* = 6.7 Hz, 48H, OC*H*$_2$), 3.84 (t, *J* = 6.6 Hz, 16H, OC*H*$_2$), 3.56 (td, *J* = 6.8, 5.9 Hz, 56H, C*H*$_2$NH), 2.71 (t, *J* = 6.8 Hz, 56H, SC*H*$_2$CH$_2$NH), 2.51 (t, *J* = 7.2 Hz, 56H, SC*H*$_2$(CH$_2$)$_2$Si), 1.84–1.76 (m, 48H, OCH$_2$C*H*$_2$CH$_2$Si^2), 1.70–1.67 (m, 16H, OCH$_2$C*H*$_2$CH$_2$Si^1), 1.61 (dt, *J* = 8.1, 1.1 Hz, 144H, Si^{2}C*H*$_2$CH), 1.56–1.54 (m, 56H, SiCH$_2$C*H*$_2$CH$_2$S), 0.71–0.59 (m, 120H, SiC*H*$_2$). **^{13}C {^{1}H} NMR** (101 MHz, CDCl$_3$, ^{1}H-^{13}C HSQC, ^{1}H-^{13}C HMBC): *δ* 167.4 (HN*C*OC$_{q(Ph)}$CH$_{Ph}$C$_{q(Ph)}$), 167.2 (HN*C*OC$_{q(Ph)}$(CH$_{Ph}$)$_2$), 161.8 ((CH$_{Ph}$)$_2$*C*$_{q(Ph)}$OCH$_2$), 160.3 (C$_{q(Ph)}$CH$_{Ph}$*C*$_{q(Ph)}$OCH$_2$), 136.6 (HNCO*C*$_{q(Ph)}$CH$_{Ph}$C$_{q(Ph)}$),

134.2 ($CHCH_2$), 129.0 ($HNCOC_{q(Ph)}CH_{Ph}CH_{Ph}$), 126.5 ($HNCOC_{q(Ph)}(CH_{Ph})_2$), 114.3 ($CH_{Ph}CH_{Ph}C_{q(Ph)}OCH_2$), 114.0 ($CHCH_2$), 105.9 ($HNCOC_{q(Ph)}CH_{Ph}C_{q(Ph)}$), 104.7 ($CH_2OC_{q(Ph)}CH_{Ph}C_{q(Ph)}OCH_2$), 70.8 ($OCH_2(CH_2)_2Si^1$), 70.7 ($OCH_2(CH_2)_2Si^2$), 39.9 ($CH_2NHCOC_{q(Ph)}CH_{Ph}C_{q(Ph)}$), 39.4 ($CH_2NHCOC_{q(Ph)}(CH_{Ph})_2$), 35.6 ($Si(CH_2)_2CH_2S$), 31.8 ($SCH_2CH_2NH$), 24.4 ($SiCH_2CH_2CH_2S$), 23.8 ($OCH_2CH_2CH_2Si^1$), 23.4 ($OCH_2CH_2CH_2Si^2$), 19.6 ($SiCH_2CH$), 11.8 ($SiCH_2(CH_2)_2S$), 8.3 ($O(CH_2)_2CH_2Si^1$), 7.6 ($O(CH_2)_2CH_2Si^2$). **^{29}Si {^{1}H} NMR** (79 MHz, $CDCl_3$): *δ* 4.02 (Si^1), 3.62 (Si^0), –0.11 (Si^2). **MALDI-TOF MS** (DCTB, Na^+): *m/z* (avg. mass) calcd. for $[C_{648}H_{968}N_{28}NaO_{60}S_{28}Si_{33}]^+$ 11958.1, found 11958.2 $[M+Na]^+$.

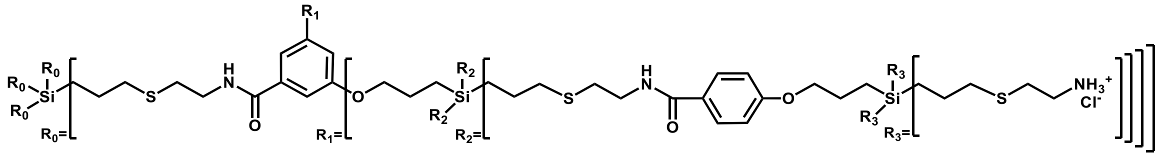

G_2-6-3-N. Dendrimer G_2-6-3-N was prepared from 200 mg (16.8 μmol) of dendrimer G_2-6-3-A according to General procedure GP2. The product was purified by OSN using 3kDa membrane in MeOH followed by lyophilization and obtained as a white powder (315 mg, 93 %). **^{1}H NMR** (400 MHz, DMSO-d_6, ^{1}H-^{1}H COSY): *δ* 8.75 (br s, 4H, N*H*), 8.62 (br s, 24H, N*H*), 8.29 (br s, 256H, NH_3^+), 7.85 (m, 48H, CH_{Ph}), 7.03 (br s, 8H, CH_{Ph}), 6.94 (m, 48H, CH_{Ph}), 6.59 (br s, 4H, CH_{Ph}), 3.94 (t, *J* = 7.0 Hz, 64H, OCH_2), 3.38 (br s, 56H, CH_2NH), 2.94 (t, *J* = 7.3 Hz, 144H, $CH_2NH_3^+$), 2.76 (t, *J* = 7.3 Hz, 144H, $CH_2CH_2NH_3^+$), 2.63 (t, *J* = 7.2 Hz, 56H, SCH_2CH_2NH), 2.54 (t, *J* = 7.1 Hz, 200H, $Si(CH_2)_2CH_2S$), 1.68 (br s, 64H, OCH_2CH_2), 1.56–1.48 (m, 200H, $SiCH_2CH_2$), 0.65–0.60 (m, 264H, $SiCH_2$). **^{13}C {^{1}H} NMR** (101 MHz, DMSO-d_6, ^{1}H-^{13}C HSQC, ^{1}H-^{13}C HMBC): *δ* 165.8 ($HNCOC_{q(Ph)}CH_{Ph}C_{q(Ph)}$), 165.6 ($HNCOC_{q(Ph)}(CH_{Ph})_2$), 160.9 ($(CH_{Ph})_2C_{q(Ph)}OCH_2$), 159.7 ($C_{q(Ph)}CH_{Ph}C_{q(Ph)}OCH_2$), 136.1 ($HNCOC_{q(Ph)}CH_{Ph}C_{q(Ph)}$), 129.1 ($HNCOC_{q(Ph)}CH_{Ph}CH_{Ph}$), 126.3 ($HNCOC_{q(Ph)}(CH_{Ph})_2$), not detected ($HNCOC_{q(Ph)}CH_{Ph}C_{q(Ph)}CH_{Ph}$), 70.2 ($OCH_2$), from HSQC 39.5 ($CH_2NH$), 38.7 ($CH_2NH_3^+$), 34.8 ($Si^{0/1}(CH_2)_2CH_2S$), 34.5 ($Si^2(CH_2)_2CH_2S$), 30.5 ($SCH_2CH_2NH$), 27.8 ($SCH_2CH_2NH_3^+$), 23.8 ($Si^{0/1}CH_2CH_2CH_2S$), 23.7 ($Si^2CH_2CH_2CH_2S$), 23.2 ($OCH_2CH_2$), 11.1 ($SiCH_2(CH_2)_2S$), 7.7 ($O(CH_2)_2CH_2Si$). **^{29}Si {^{1}H} NMR** (79 MHz, DMSO-d_6): *δ* not detected or overlapped (Si^0), 4.03 (Si^2), 3.94 (Si^1). **MALDI-TOF MS** (DHB, Na^+): *m/z* (avg. mass) calcd. for $[C_{792}H_{1472}N_{100}NaO_{60}S_{100}Si_{33}]^+$ 17512.7, found 17513.2 $[M+Na]^+$.

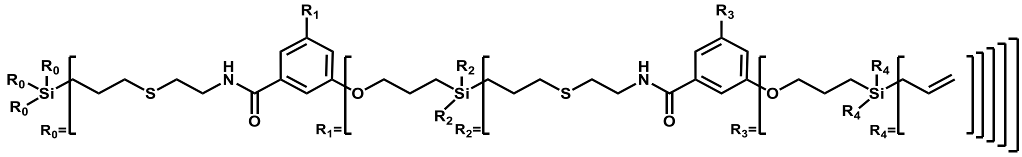

G_2-6-6-A. Dendrimer G_2-6-6-A was prepared from 1.00 g (188 μmol) of G_1-6-N according to General procedure GP1. The product was purified by OSN using 3kDa membrane in DCM/MeOH mixture 1:1 and obtained as a yellow viscous substance (3.13 g, 98 %). **^{1}H NMR** (400 MHz, $CDCl_3$, ^{1}H-^{1}H COSY): *δ* 6.95 (br s, 28H, N*H*), 6.90 (d, *J* = 2.2 Hz, 56H, CH_{Ph}), 6.52 (t, *J* = 2.2 Hz, 28H, CH_{Ph}), 5.77 (ddt, *J* = 16.4, 10.2, 8.1 Hz, 144H, $CHCH_2$), 4.90–4.85 (m, 288H, $CHCH_2$), 3.85 (t, 112H, *J* = 6.5 Hz, OCH_2), 3.58 (td, *J* = 6.8, 5.9 Hz, 56H, CH_2NH), 2.72 (t, *J* = 6.8 Hz, 56H, SCH_2CH_2NH), 2.53 (t, *J* = 7.2 Hz, 56H, $SCH_2(CH_2)_2Si$), 1.80–1.72 (m, 112H, $OCH_2CH_2CH_2Si$), 1.60 (dt, *J* = 8.1, 1.1 Hz, 288H, $SiCH_2CH$), 1.59–1.54 (m, 56H, $SiCH_2CH_2CH_2S$), 0.68–0.60 (m, 168H, $SiCH_2$). **^{13}C {^{1}H} NMR** (101 MHz, $CDCl_3$, ^{1}H-^{13}C HSQC, ^{1}H-^{13}C HMBC): *δ* 167.4 (HN*C*O), 160.4 ($C_{q(Ph)}OCH_2$), 136.5 ($HNCOC_{q(Ph)}$), 134.2 ($CHCH_2$), 114.0 ($CHCH_2$), 105.6 ($HNCOC_{q(Ph)}CH_{Ph}C_{q(Ph)}$), 104.6 ($CH_2OC_{q(Ph)}CH_{Ph}C_{q(Ph)}OCH_2$), 70.8 ($OCH_2$), 39.4 ($CH_2NH$), 35.6 ($Si(CH_2)_2CH_2S$), 31.7 ($SCH_2CH_2NH$), 24.4 ($SiCH_2CH_2CH_2S$), 23.9 ($OCH_2CH_2CH_2Si^1$), 23.5 ($OCH_2CH_2CH_2Si^2$), 19.6 ($SiCH_2CH$), 11.8 ($SiCH_2(CH_2)_2S$), 8.4 ($Si^1CH_2(CH_2)_2O$), 7.6 ($Si^2CH_2(CH_2)_2O$). **^{29}Si {^{1}H} NMR** (79 MHz, $CDCl_3$): *δ* 4.06 (Si^1), –0.12 (Si^2), not detected or overlapped (Si^0). **MALDI-TOF MS** (DCTB, Na^+): *m/z* (avg. mass) calcd. for $[C_{936}H_{1448}N_{28}NaO_{84}S_{28}Si_{57}]^+$ 16959.3, found 16960.6 $[M+Na]^+$.

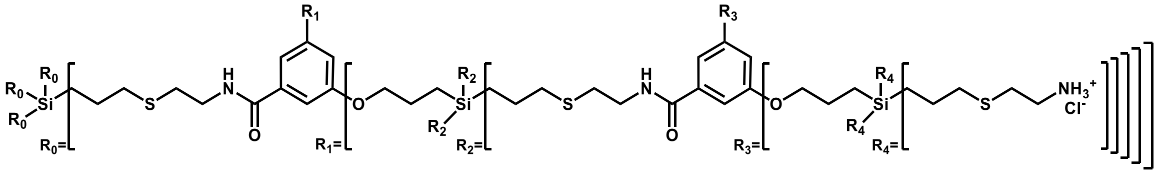

G$_2$-6-6-N. Dendrimer G$_2$-6-6-N was prepared from 200 mg (11.8 µmol) of dendrimer G$_2$-6-6-A according to General procedure GP2. The product was purified by OSN using 3kDa membrane in MeOH followed by lyophilization and obtained as off-white powder (370 mg, 94 %). **^{1}H NMR** (400 MHz, DMSO-d_6, ^{1}H-^{1}H COSY): δ 8.83 (br s, 28H, N*H*), 8.32 (br s, 432H, NH_3^+), 7.04 (br s, 56H, CH_{Ph}), 6.57 (br s, 28H, CH_{Ph}), 3.93 (br s, 112H, OCH_2), overlapped with water (56H, CH_2NH), 2.95 (t, J = 7.3 Hz, 298H, CH_2NH$_3^+$), 2.77 (t, J = 7.4 Hz, 298H, CH_2CH$_2$NH$_3^+$), 2.66 (br s, 56H, SCH_2CH$_2$NH), 2.55 (t, J = 7.0 Hz, 354H, Si(CH$_2$)$_2$CH_2S), 1.67 (br s, 112H, OCH$_2$CH_2), 1.56–1.48 (m, 354H, SiCH$_2$CH_2), 0.65–0.62 (m, 466H, SiCH_2). **^{13}C {^{1}H} NMR** (101 MHz, DMSO-d_6, ^{1}H-^{13}C HSQC, ^{1}H-^{13}C HMBC): δ 165.6 (NH*C*O), 159.7 ($C_{q(Ph)}$OCH$_2$), 136.2 (HNCO$C_{q(Ph)}$), 105.7 (HNCOC$_{q(Ph)}$$CH_{Ph}$), 103.9 ($CH_{Ph}$), 70.4 (O$CH_2$), from HSQC 37.9 ($CH_2$NH), 38.7 ($CH_2NH_3^+$), 35.0 (Si$^{0/1}$(CH$_2$)$_2$$CH_2$S), 34.5 (Si^2(CH$_2$)$_2$$CH_2$S), 30.4 (S$CH_2CH_2$NH), 27.8 (S$CH_2CH_2NH_3^+$), 23.8 (SiCH$_2$$CH_2$), 23.3 (OCH$_2$$CH_2$), 11.2 (Si$CH_2$(CH$_2$)$_2$S), 7.8 (O(CH$_2$)$_2$$CH_2$Si). **^{29}Si {^{1}H} NMR** (79 MHz, DMSO-d_6): δ 4.01 (Si^2), not detected or overlapped (Si$^{0/1}$). **MALDI-TOF MS** (DHB, Na$^+$): *m/z* (avg. mass) calcd. for $[C_{1218}H_{2436}N_{169}O_{84}S_{169}Si_{57}]^+$ 27814.9, calcd. for $[C_{1218}H_{2435}N_{169}NaO_{84}S_{169}Si_{57}]^+$ 27836.8, found 27830.5 overlap of [M-3x(HSCH$_2$CH$_2$NH$_2$)+H]$^+$ and [M-3x(HSCH$_2$CH$_2$NH$_2$)+Na]$^+$.

3rd generation dendrimers

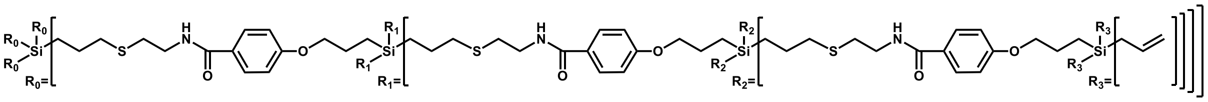

G$_3$-3-3-3-A. Dendrimer G$_3$-3-3-3-A was prepared from 450 mg (42.8 µmol) of G$_2$-3-3-N according to General procedure GP1. The product was purified by OSN using 3kDa membrane in DCM/MeOH mixture 1:1 and obtained as a brownish viscous substance (810 mg, 92 %). **^{1}H NMR** (400 MHz, CDCl$_3$, ^{1}H-^{1}H COSY): δ 7.75 (m, 108H, CH_{Ph}C$_{q(Ph)}$CONH), 7.19 (br s, 4H, N*H*), 7.11 (t, J = 5.5 Hz, 12H, N*H*), 6.93 (t, J = 5.5 Hz, 36H, N*H*), 6.86 (m, 108H, CH_{Ph}C$_{q(Ph)}$OCH$_2$), 5.78 (ddt, J = 16.5, 10.1, 8.1 Hz, 108H, C*H*CH$_2$), 4.92–4.85 (m, 216H, CHCH_2), 3.90 (t, J = 6.7 Hz, 104H, OCH_2), 3.57 (td, J = 6.6, 5.5 Hz, 104H, CH_2NH), 2.71 (t, J = 6.6 Hz, 104H, SCH_2CH$_2$NH), 2.51 (t, J = 7.1 Hz, 104H, SCH_2(CH$_2$)$_2$Si), 1.84–1.72 (m, 72H, OCH$_2$CH_2CH$_2$Si^3), 1.76–1.69 (m, 32H, OCH$_2$CH_2CH$_2$Si$^{1/2}$), 1.61 (dt, J = 8.1, 1.1 Hz, 216H, SiCH_2CH), 1.57–1.53 (m, 104H, SiCH$_2$CH_2CH$_2$S), 0.71–0.61 (m, 208H, SiCH_2). **^{13}C {^{1}H} NMR** (101 MHz, CDCl$_3$, ^{1}H-^{13}C HSQC, ^{1}H-^{13}C HMBC): δ 167.3 (HN$^{0/1}$*C*O), 167.2 (HN^2*C*O), 161.8 ($C_{q(Ph)}$O$^{0/1}$CH$_2$), 161.6 ($C_{q(Ph)}$O^{2}CH$_2$), 134.2 (CHCH$_2$), 129.04 (HN$^{0/1}$COC$_{q(Ph)}$$CH_{Ph}$), 128.95 (HN^{2}COC$_{q(Ph)}$$CH_{Ph}$), 126.6 (HN$^{0/1}COC_{q(Ph)}$), 126.5 (HN^{2}CO$C_{q(Ph)}$), 114.3 ($CH_{Ph}$COCH$_2$), 114.0 (CH$CH_2$), 70.7 (O^2$CH_2$), 70.6 (O$^{0/1}$$CH_2$), 39.4 ($CH_2N^{0/1}$H), 39.2 ($CH_2$N^{2}H), 35.64 (Si$^{0/1}$(CH$_2$)$_2$$CH_2$S), 35.61 (Si^2(CH$_2$)$_2$$CH_2$S), 31.9 (S$CH_2CH_2$N^{2}H), 31.8 (S$CH_2CH_2N^{0/1}$H), 24.4 (SiCH$_2$$CH_2CH_2$S), 23.7 (O$^{0/1}CH_2$$CH_2$), 23.4 (O^{2}CH$_2$$CH_2$), 19.6 (Si$CH_2$CH), 11.8 (Si$CH_2$(CH$_2$)$_2$S), 8.3 (O$^{0/1}$(CH$_2$)$_2$$CH_2$), 7.6 (O^2(CH$_2$)$_2$$CH_2$). **^{29}Si {^{1}H} NMR** (79 MHz, CDCl$_3$): δ 4.04 (Si^2), 4.03 (Si^1), not detected or overlapped (Si^0), –0.11 (Si^3). **MALDI-TOF MS** (DCTB, Na$^+$): *m/z* (avg. mass) calcd. for $[C_{1104}H_{1633}N_{52}O_{104}S_{52}Si_{53}]^+$ 20453.6, found 20456.8 [M+H]$^+$.

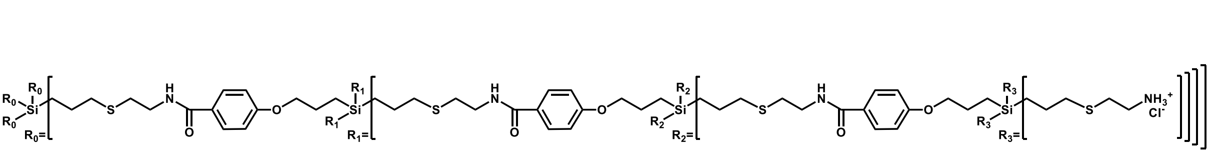

G_3-3-3-3-N. Dendrimer G_3-3-3-3-N was prepared from 597 mg (29.2 µmol) of dendrimer G_3-3-3-3-A according to General procedure GP2. The product was purified by OSN using 3kDa membrane in MeOH followed by lyophilization and obtained as a white powder (946 mg, 99 %). **^{1}H NMR** (400 MHz, **100 °C**, DMSO-d_6, ^{1}H-^{1}H COSY): δ 8.27 (br s, 52H, N*H*, 324H, $N\mathit{H}_3^+$), 7.83 (m, 104H, $C\mathit{H}_{Ph}C_{q(Ph)}CONH$), 6.93 (m, 104H, $C\mathit{H}_{Ph}C_{q(Ph)}OCH_2$), 4.00 (t, *J* = 6.3 Hz, 72H, $OC\mathit{H}_2(CH_2)_2Si^3$), 3.97 (t, *J* = 6.3 Hz, 32H, $OC\mathit{H}_2(CH_2)_2Si^3$), 3.43 (ddd, *J* = 8.2, 6.3, 5.8 Hz, 104H, $C\mathit{H}_2NH$), 2.98 (dd, *J* = 8.6, 6.2 Hz, 216H, $C\mathit{H}_2NH_3^+$), 2.83 (dd, *J* = 8.6, 6.2 Hz, 216H, $C\mathit{H}_2CH_2NH_3^+$), 2.69 (dd, *J* = 8.2, 6.3 Hz, 104H, $SC\mathit{H}_2CH_2NH$), 2.58 (t, *J* = 7.4 Hz, 216H, $SC\mathit{H}_2(CH_2)_2Si^3$), 2.56 (t, *J* = 7.4 Hz, 104H, $SC\mathit{H}_2(CH_2)_2Si^{2/1}$), 1.78–1.71 (m, 104H, $OCH_2C\mathit{H}_2$), 1.63–1.55 (m, 320H, $SiCH_2C\mathit{H}_2CH_2S$), 0.70–0.64 (m, 424H, $SiC\mathit{H}_2$). **^{1}H NMR** (400 MHz, DMSO-d_6, ^{1}H-^{1}H COSY): δ 8.61 (br s, 52H, N*H*), 8.30 (br s, 324H, $N\mathit{H}_3^+$), 7.84 (m, 104H, $C\mathit{H}_{Ph}C_{q(Ph)}CONH$), 6.94 (m, 104H, $C\mathit{H}_{Ph}C_{q(Ph)}OCH_2$), 3.94 (t, *J* = 6.3 Hz, 104H, $OC\mathit{H}_2$), 3.41–3.35 overlapped with H_2O (m, 104H, $C\mathit{H}_2NH$), 2.93 (t, *J* = 7.4 Hz, 216H, $C\mathit{H}_2NH_3^+$), 2.75 (t, *J* = 7.4 Hz, 216H, $C\mathit{H}_2CH_2NH_3^+$), 2.63 (t, *J* = 7.2 Hz, 104H, $SC\mathit{H}_2CH_2NH$), 2.54 (t, *J* = 7.2 Hz, 320H, $SC\mathit{H}_2(CH_2)_2Si$), 1.70–1.66 (m, 104H, $OCH_2C\mathit{H}_2$), 1.55–1.47 (m, 320H, $SiCH_2C\mathit{H}_2CH_2S$), 0.70–0.60 (m, 424H, $SiC\mathit{H}_2$). **^{13}C {^{1}H} NMR** (101 MHz, DMSO-d_6, ^{1}H-^{13}C HSQC, ^{1}H-^{13}C HMBC): δ 165.6 (HN*C*O), 160.9 ($\mathit{C}_{q(Ph)}OCH_2$), 129.1 ($HNCOC_{q(Ph)}\mathit{C}H_{Ph}$), 126.3 ($HNCO\mathit{C}_{q(Ph)}$), 113.9 ($\mathit{C}H_{Ph}C_{q(Ph)}OCH_2$), 70.2 ($O\mathit{C}H_2$), from HSQC 39.6 ($\mathit{C}H_2NH$), 38.7 ($\mathit{C}H_2NH_3^+$), 34.8 ($\mathit{C}H_2S(CH_2)_2NH$), 34.5 ($\mathit{C}H_2S(CH_2)_2NH_3^+$), 30.5 ($S\mathit{C}H_2CH_2NH$), 27.8 ($S\mathit{C}H_2CH_2NH_3^+$), 23.8 ($\mathit{C}H_2CH_2S(CH_2)_2NH$), 23.7 ($\mathit{C}H_2CH_2S(CH_2)_2NH_3^+$), 23.2 ($OCH_2\mathit{C}H_2$), 11.2, 11.1 ($Si\mathit{C}H_2(CH)_2S$), 7.7 ($O(CH_2)_2\mathit{C}H_2$). **^{29}Si {^{1}H} NMR** (79 MHz, DMSO-d_6): δ overlapped or not detected ($Si^{0/1}$), 4.03 (Si^3), 3.97 (Si^2). **MALDI-TOF MS** (DHB, Na^+): *m/z* (avg. mass) calcd. for $[C_{1316}H_{2374}N_{158}O_{104}S_{158}Si_{53}]^+$ 28653.3, found 28651.3 $[M-2x(HSCH_2CH_2NH_2)+Na]^+$.

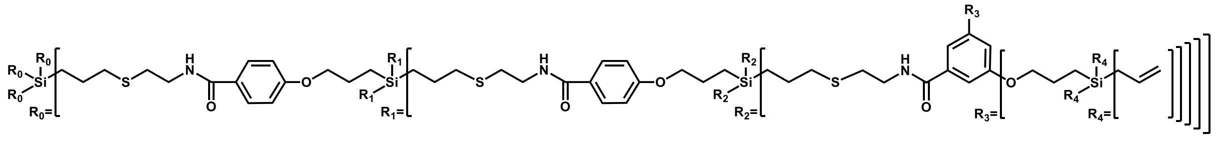

G_3-3-3-6-A. Dendrimer G_2-3-3-6-A was prepared from 200 mg (19.0 µmol) of G_2-3-3-N according to General procedure GP1. The product was purified by OSN using 3kDa membrane in DCM/MeOH mixture 1:1 and obtained as a brownish amorphous solid (485 mg, 91 %). **^{1}H NMR** (400 MHz, $CDCl_3$, ^{1}H-^{1}H COSY): δ 7.74 (m, 32H, $HNCOC_{q(Ph)}C\mathit{H}_{Ph}CH_{Ph}$), 6.89 (d, *J* = 2.2 Hz, 36H, $HNCOC_{q(Ph)}C\mathit{H}_{Ph}C_{q(Ph)}$), 6.88 (m, 32H, $C\mathit{H}_{Ph}$), 6.83 (br s, 52H, N*H*), 6.53 (t, *J* = 2.2 Hz, 36H, $C\mathit{H}_{Ph}$), 5.77 (ddt, *J* = 16.3, 9.9, 8.1 Hz, 216H, $C\mathit{H}CH_2$), 4.91–4.85 (m, 432H, $CHC\mathit{H}_2$), 3.92, 3.87 (2×t, *J* = 6.1 Hz, 176H, $OC\mathit{H}_2$), 3.58 (td, *J* = 6.5, 5.7 Hz, 104H, $C\mathit{H}_2NH$), 2.72 (t, *J* = 6.5 Hz, 104H, $SC\mathit{H}_2CH_2NH$), 2.53 (t, *J* = 7.2 Hz, 104H, $SC\mathit{H}_2(CH_2)_2Si$), 1.81–1.74 (m, 176H, $OCH_2C\mathit{H}_2CH_2Si$), 1.61–1.57 (m, 104H, $SiCH_2C\mathit{H}_2CH_2S$), 1.60 (dt, *J* = 8.1, 1.1 Hz, 432H, $SiC\mathit{H}_2CH$), 0.69–0.63 (m, 280H, $SiC\mathit{H}_2$). **^{13}C {^{1}H} NMR** (101 MHz, $CDCl_3$, ^{1}H-^{13}C HSQC, ^{1}H-^{13}C HMBC): δ 167.4 ($HN\mathit{C}OC_{q(Ph)}CH_{Ph}C_{q(Ph)}$), 167.2 ($HN\mathit{C}OC_{q(Ph)}(CH_{Ph})_2$), 161.7 ($(CH_{Ph})_2\mathit{C}_{q(Ph)}OCH_2$), 160.4 ($C_{q(Ph)}CH_{Ph}\mathit{C}_{q(Ph)}OCH_2$), 136.5 ($HNCO\mathit{C}_{q(Ph)}CH_{Ph}C_{q(Ph)}$), 134.2 ($\mathit{C}HCH_2$), 129.0 ($HNCOC_{q(Ph)}\mathit{C}H_{Ph}CH_{Ph}$), 126.6 ($HNCO\mathit{C}_{q(Ph)}(CH_{Ph})_2$), 114.3 ($CH_{Ph}\mathit{C}H_{Ph}C_{q(Ph)}OCH_2$), 114.0 ($CH\mathit{C}H_2$), 105.6 ($HNCOC_{q(Ph)}\mathit{C}H_{Ph}C_{q(Ph)}$), 104.5 ($CH_2OC_{q(Ph)}\mathit{C}H_{Ph}C_{q(Ph)}OCH_2$), 70.8 ($O\mathit{C}H_2(CH_2)_2Si^3$), 70.6 ($O\mathit{C}H_2(CH_2)_2Si^{1/2}$), 39.3 ($\mathit{C}H_2NH$), 35.6 ($Si(CH_2)_2\mathit{C}H_2S$), 31.8 ($S\mathit{C}H_2CH_2NH$), 24.4 ($SiCH_2\mathit{C}H_2CH_2S$), 23.7 ($OCH_2\mathit{C}H_2CH_2Si^{1/2}$), 23.4 ($OCH_2\mathit{C}H_2CH_2Si^3$), 19.6 ($Si\mathit{C}H_2CH$), 11.8 ($Si\mathit{C}H_2(CH_2)_2S$), 8.3 ($O(CH_2)_2\mathit{C}H_2Si^{1/2}$), 7.62 ($O(CH_2)_2\mathit{C}H_2Si^3$). **^{29}Si {^{1}H} NMR** (79 MHz, $CDCl_3$): δ not detected or overlapped ($Si^{0/1}$), 4.07 (Si^2), –0.12 (Si^3).

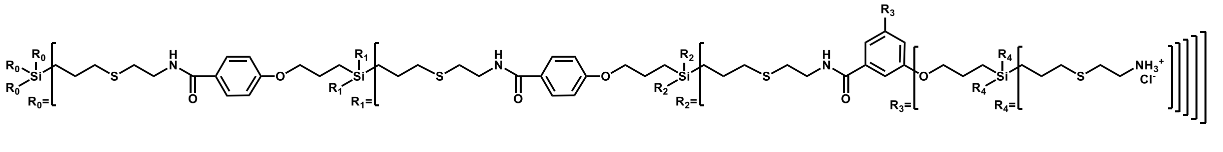

G$_3$-3-3-6-N. Dendrimer G$_3$-3-3-6-N was prepared from 160 mg (5.72 µmol) of dendrimer G$_3$-3-3-6-A according to General procedure GP2. The product was purified by OSN using 3kDa membrane in MeOH followed by lyophilization and obtained as an off-white powder (267 mg, 89 %). **^{1}H NMR** (400 MHz, **100 °C**, DMSO-*d*$_6$, ^{1}H-^{1}H COSY): *δ* 8.50 (br s, 52H, N*H*), 8.30 (br s, 648H, N*H*$_3^+$), 7.85 (m, 32H, NHCOC$_{q(Ph)}$C*H*$_{Ph}$CH$_{Ph}$), 7.06 (d, *J* = 2.2 Hz, 72H, NHCOC$_{q(Ph)}$C*H*$_{Ph}$C$_{q(Ph)}$), 6.95 (m, 32H, CH$_{Ph}$C*H*$_{Ph}$C$_{q(Ph)}$OCH$_2$), 6.60 (t, *J* = 2.2 Hz, 36H, NHCOC$_{q(Ph)}$C*H*$_{Ph}$C$_{q(Ph)}$OCH$_2$), 4.00 (t, *J* = 6.6 Hz, 176H, OC*H*$_2$), 3.45 (td, *J* = 7.3, 5.6 Hz, 104H, C*H*$_2$NH), 3.00 (t, *J* = 7.4 Hz, 432H, C*H*$_2$NH$_3^+$), 2.85 (t, *J* = 7.4 Hz, 432H, C*H*$_2$CH$_2$NH$_3^+$), 2.72 (t, *J* = 7.3 Hz, 104H, C*H*$_2$CH$_2$NH), 2.60 (t, *J* = 7.1 Hz, 536H, SC*H*$_2$(CH$_2$)$_2$Si), 1.77–1.71 (m, 176H, OCH$_2$C*H*$_2$CH$_2$Si), 1.64–1.57 (m, 536H, SiCH$_2$C*H*$_2$CH$_2$S), 0.72–0.66 (m, 712H, SiC*H*$_2$). **^{1}H NMR** (400 MHz, DMSO-*d*$_6$, ^{1}H-^{1}H COSY): *δ* 8.81 (br s, 36H, N*H*COC$_{q(Ph)}$CH$_{Ph}$C$_{q(Ph)}$), 8.66 (br s, 16H, N*H*COC$_{q(Ph)}$(CH$_{Ph}$)$_2$), 8.33 (br s, 648H, N*H*$_3^+$), 7.85 (br s, 32H, NHCOC$_{q(Ph)}$C*H*$_{Ph}$CH$_{Ph}$), 7.03 (s, 72H, NHCOC$_{q(Ph)}$C*H*$_{Ph}$C$_{q(Ph)}$), 6.93 (br s, 32H, CH$_{Ph}$C*H*$_{Ph}$C$_{q(Ph)}$OCH$_2$), 6.57 (br s, 36H, NHCOC$_{q(Ph)}$C*H*$_{Ph}$C$_{q(Ph)}$OCH$_2$), 3.94 (br s, 176H, OC*H*$_2$), overlapped with water (C*H*$_2$NH), 2.94 (t, *J* = 7.4 Hz, 432H, C*H*$_2$NH$_3^+$), 2.76 (t, *J* = 7.4 Hz, 432H, C*H*$_2$CH$_2$NH$_3^+$), 2.63 (t, *J* = 7.3 Hz, 104H, C*H*$_2$CH$_2$NH), 2.55 (t, *J* = 7.1 Hz, 536H, SC*H*$_2$(CH$_2$)$_2$Si), 1.67 (br s, 176H, OCH$_2$C*H*$_2$CH$_2$Si), 1.56–1.48 (m, 536H, SiCH$_2$C*H*$_2$CH$_2$S), 0.65–0.61 (m, 712H, SiC*H*$_2$). **^{13}C {^{1}H} NMR** (101 MHz, DMSO-*d*$_6$, ^{1}H-^{13}C HSQC, ^{1}H-^{13}C HMBC): *δ* 165.6 (HN*C*OC$_{q(Ph)}$CH$_{Ph}$C$_{q(Ph)}$), not detected (HN*C*OC$_{q(Ph)}$(CH$_{Ph}$)$_2$), 160.9 ((CH$_{Ph}$)$_2$*C*$_{q(Ph)}$OCH$_2$), 159.6 (C$_{q(Ph)}$(CH$_{Ph}$)*C*$_{q(Ph)}$OCH$_2$), 136.2 (HNCO*C*$_{q(Ph)}$CH$_{Ph}$C$_{q(Ph)}$), 129.0 (HNCOC$_{q(Ph)}$*C*H$_{Ph}$CH$_{Ph}$), 126.2 (HNCO*C*$_{q(Ph)}$(CH$_{Ph}$)$_2$), 113.8 (CH$_{Ph}$*C*H$_{Ph}$C$_{q(Ph)}$OCH$_2$), 105.7 (HNCOC$_{q(Ph)}$*C*H$_{Ph}$C$_{q(Ph)}$), 103.9 (OC$_{q(Ph)}$*C*H$_{Ph}$C$_{q(Ph)}$O), 70.4 (O*C*H$_2$(CH$_2$)$_2$Si), from HSQC 39.7 (*C*H$_2$NH), 38.7 (*C*H$_2$NH$_3^+$), 34.8 (*C*H$_2$S(CH$_2$)$_2$NH), 34.5 (*C*H$_2$S(CH$_2$)$_2$NH$_3^+$), 30.4 (*C*H$_2$CH$_2$NH), 27.8 (*C*H$_2$CH$_2$NH$_3^+$), 23.7 (*C*H$_2$CH$_2$S(CH$_2$)$_2$NH), 23.3 (*C*H$_2$CH$_2$O), 11.1 (Si*C*H$_2$(CH$_2$)$_2$S), 7.8 (Si*C*H$_2$(CH$_2$)$_2$O). **^{29}Si {^{1}H} NMR** (79 MHz, DMSO-*d*$_6$): *δ* 4.01 (Si^3), not detected or overlapped (Si$^{0/1/2}$).

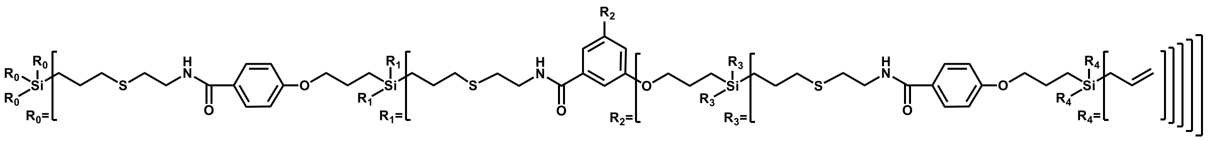

G$_3$-3-6-3-A. Dendrimer G$_3$-3-6-3-A was prepared from 100 mg (5.85 µmol) of G$_2$-3-6-N according to General procedure GP1. The product was purified by OSN using 3kDa membrane in DCM/MeOH mixture, starting at 1:1 ratio and gradually increasing the polarity up to 1:2 so as to maintain the homogeneity of the solution, and obtained as a brownish amorphous solid (207 mg, 95 %). **^{1}H NMR** (400 MHz, CDCl$_3$, ^{1}H-^{1}H COSY): *δ* 7.76 (m, 152H, HNCOC$_{q(Ph)}$C*H*$_{Ph}$CH$_{Ph}$), 7.10 (br s, 88H, N*H*), 6.98 (br s, 24H, HNCOC$_{q(Ph)}$C*H*$_{Ph}$C$_{q(Ph)}$), 6.84 (m, 152H, C*H*$_{Ph}$), 6.53 (br s, 12H, C*H*$_{Ph}$), 5.77 (ddt, *J* = 16.4, 10.1, 8.1 Hz, 216H, C*H*CH$_2$), 4.91–4.84 (m, 432H, CHC*H*$_2$), 3.91 (2×t, *J* = 6.7 Hz, 200H, OC*H*$_2$), 3.56 (td, *J* = 6.8, 5.7 Hz, 176H, C*H*$_2$NH), 2.70 (t, *J* = 6.8 Hz, 176H, SC*H*$_2$CH$_2$NH), 2.50 (t, *J* = 6.9 Hz, 176H, SC*H*$_2$(CH$_2$)$_2$Si), 1.83–1.76 (m, 152H, OCH$_2$C*H*$_2$CH$_2$Si$^{0/3}$), 1.67 (br s, 48H, OCH$_2$C*H*$_2$CH$_2$Si^2), 1.60 (dt, *J* = 8.1, 1.1 Hz, 432H, SiC*H*$_2$CH), 1.59–1.52 (m, 176H, SiCH$_2$C*H*$_2$CH$_2$S), 0.70–0.56 (m, 152H, Si$^{0/3}$C*H*$_2$(CH$_2$)$_2$O), 0.63–0.59 (m, 176H, SiC*H*$_2$(CH$_2$)$_2$S, 48H, Si^{2}C*H*$_2$(CH$_2$)$_2$O). **^{13}C {^{1}H} NMR** (101 MHz, CDCl$_3$, ^{1}H-^{13}C HSQC, ^{1}H-^{13}C HMBC): *δ* 167.6 (HN*C*OC$_{q(Ph)}$CH$_{Ph}$C$_{q(Ph)}$), 167.2 (HN*C*OC$_{q(Ph)}$(CH$_{Ph}$)$_2$), 161.8 ((CH$_{Ph}$)$_2$*C*$_{q(Ph)}$OCH$_2$), 160.3 (C$_{q(Ph)}$CH$_{Ph}$*C*$_{q(Ph)}$OCH$_2$), 136.6 (HNCO*C*$_{q(Ph)}$CH$_{Ph}$C$_{q(Ph)}$), 134.2 (*C*HCH$_2$), 129.0 (HNCOC$_{q(Ph)}$*C*H$_{Ph}$CH$_{Ph}$), 126.5 (HNCO*C*$_{q(Ph)}$(CH$_{Ph}$)$_2$), 114.3 (CH$_{Ph}$*C*H$_{Ph}$C$_{q(Ph)}$OCH$_2$), 114.0 (CH*C*H$_2$), 105.8 (HNCOC$_{q(Ph)}$*C*H$_{Ph}$C$_{q(Ph)}$), 104.6 (CH$_2$OC$_{q(Ph)}$*C*H$_{Ph}$C$_{q(Ph)}$OCH$_2$), 70.8 (O*C*H$_2$(CH$_2$)$_2$Si^2), 70.7 (O*C*H$_2$(CH$_2$)$_2$Si$^{1/3}$), 39.4 (*C*H$_2$NH), 35.6 (Si(CH$_2$)$_2$*C*H$_2$S), 31.8 (S*C*H$_2$CH$_2$NH), 24.4 (SiCH$_2$*C*H$_2$CH$_2$S), 23.8 (OCH$_2$*C*H$_2$CH$_2$Si^2), 23.4 (OCH$_2$*C*H$_2$CH$_2$Si$^{1/3}$), 19.6 (Si*C*H$_2$CH), 11.8 (Si*C*H$_2$(CH$_2$)$_2$S), 8.3 (O(CH$_2$)$_2$*C*H$_2$Si^2), 7.6 (O(CH$_2$)$_2$*C*H$_2$Si$^{1/3}$). **^{29}Si {^{1}H} NMR** (79 MHz, CDCl$_3$): *δ* not detected or overlapped (Si$^{0/1}$), 4.02 (Si^2), –0.11 (Si^3).

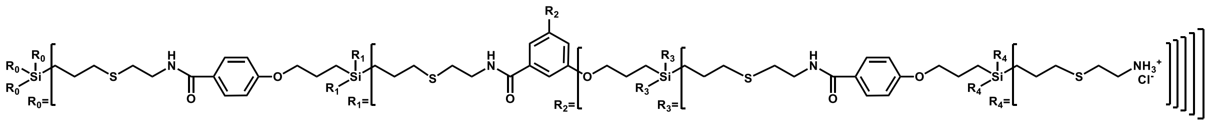

G_3-3-6-3-N. Dendrimer G_3-3-6-3-N was prepared from 150 mg (4.06 µmol) of dendrimer G_3-3-6-3-A according to General procedure GP2. The product was purified by OSN using 3kDa membrane in MeOH followed by lyophilization and obtained as an off-white powder (244 mg, 97 %). **^{1}H NMR** (400 MHz, **100 °C**, DMSO-d_6, ^{1}H-^{1}H COSY): δ 8.32 (br s, 736H, N*H*, NH_3^+), 7.85 (m, 152H, NHCOC$_{q(Ph)}$C*H*$_{Ph}$CH$_{Ph}$), 7.03 (s, 24H, NHCOC$_{q(Ph)}$C*H*$_{Ph}$C$_{q(Ph)}$), 6.93 (m, 152H, CH$_{Ph}$C*H*$_{Ph}$C$_{q(Ph)}$OCH$_2$), 6.59 (s, 12H, NHCOC$_{q(Ph)}$C*H*$_{Ph}$C$_{q(Ph)}$OCH$_2$), 3.99 (t, *J* = 6.6 Hz, 200H, OCH_2), (td, *J* = 7.3, 5.8 Hz, 176H, CH_2NH), 2.99 (t, *J* = 7.4 Hz, 432H, CH_2NH_3^+), 2.84 (t, *J* = 7.4 Hz, 432H, CH_2CH$_2$NH$_3^+$), 2.69 (t, *J* = 7.3 Hz, 176H, CH_2CH$_2$NH), 2.58 (t, *J* = 7.1 Hz, 608H, SCH_2(CH$_2$)$_2$Si), 1.77–1.73 (m, 200H, OCH$_2$CH_2CH$_2$Si), 1.62–1.55 (m, 608H, SiCH$_2$CH_2CH$_2$S), 0.70–0.64 (m, 808H, SiCH_2). **^{1}H NMR** (400 MHz, DMSO-d_6, ^{1}H-^{1}H COSY): δ 8.72 (br s, 12H, N*H*COC$_{q(Ph)}$CH$_{Ph}$C$_{q(Ph)}$), 8.64 (br s, 76H, N*H*COC$_{q(Ph)}$(CH$_{Ph}$)$_2$), 8.32 (br s, 648H, NH_3^+), 7.85 (m, 152H, NHCOC$_{q(Ph)}$C*H*$_{Ph}$CH$_{Ph}$), 7.03 (s, 24H, NHCOC$_{q(Ph)}$C*H*$_{Ph}$C$_{q(Ph)}$), 6.93 (m, 152H, CH$_{Ph}$C*H*$_{Ph}$C$_{q(Ph)}$OCH$_2$), 6.58 (s, 12H, NHCOC$_{q(Ph)}$C*H*$_{Ph}$C$_{q(Ph)}$OCH$_2$), 3.93 (br s, 200H, OCH_2), overlapped with water (br s, 176H, CH_2NH), 2.94 (t, *J* = 7.4 Hz, 432H, CH_2NH$_3^+$), 2.76 (t, *J* = 7.4 Hz, 432H, CH_2CH$_2$NH$_3^+$), 2.62 (t, *J* = 7.3 Hz, 176H, CH_2CH$_2$NH), 2.54 (t, *J* = 7.0 Hz, 608H, SCH_2(CH$_2$)$_2$Si), 1.67 (br s, 200H, OCH$_2$CH_2CH$_2$Si), 1.53–1.49 (m, 608H, SiCH$_2$CH_2CH$_2$S), 0.64–0.59 (m, 808H, SiCH_2). **^{13}C {^{1}H} NMR** (101 MHz, DMSO-d_6, ^{1}H-^{13}C HSQC, ^{1}H-^{13}C HMBC): δ not detected (HN*C*OC$_{q(Ph)}$CH$_{Ph}$C$_{q(Ph)}$), 165.6 (HN*C*OC$_{q(Ph)}$(CH$_{Ph}$)$_2$), 160.9 ((CH$_{Ph}$)$_2$*C*$_{q(Ph)}$OCH$_2$), 159.6 (C$_{q(Ph)}$(CH$_{Ph}$)*C*$_{q(Ph)}$OCH$_2$), not detected (HNCO*C*$_{q(Ph)}$CH$_{Ph}$C$_{q(Ph)}$), 129.1 (HNCOC$_{q(Ph)}$*C*H$_{Ph}$CH$_{Ph}$), 126.3 (HNCO*C*$_{q(Ph)}$(CH$_{Ph}$)$_2$), 113.8 (CH$_{Ph}$*C*H$_{Ph}$C$_{q(Ph)}$OCH$_2$), from HSQC at 100 °C 105.4 (HNCOC$_{q(Ph)}$*C*H$_{Ph}$C$_{q(Ph)}$), not detected (OC$_{q(Ph)}$*C*H$_{Ph}$C$_{q(Ph)}$O), 70.2 (O*C*H$_2$(CH$_2$)$_2$Si), from HSQC 39.6 (*C*H$_2$NHCO), 38.7 (*C*H$_2$NH$_3^+$), 34.8 (*C*H$_2$S(CH$_2$)$_2$NH), 34.5 (*C*H$_2$S(CH$_2$)$_2$NH$_3^+$), 30.5 (*C*H$_2$CH$_2$NH), 27.8 (*C*H$_2$CH$_2$NH$_3^+$), 23.8 (*C*H$_2$CH$_2$S(CH$_2$)$_2$NH), 23.2 (*C*H$_2$CH$_2$O), 11.1 (Si*C*H$_2$(CH$_2$)$_2$S), 7.7 (Si*C*H$_2$(CH$_2$)$_2$O). **^{29}Si {^{1}H} NMR** (79 MHz, DMSO-d_6): δ 4.02 (Si^3), 3.93 (Si^2), not detected or overlapped (Si$^{0/1}$).

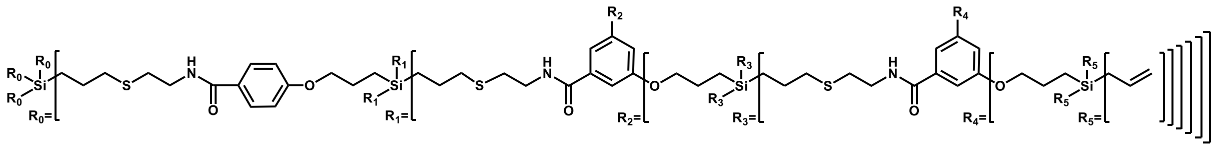

G_3-3-6-6-A. Dendrimer G_3-3-6-6-A was prepared from 206 mg (12.0 µmol) of G_2-3-6-N according to General procedure GP1. The product was purified by OSN using 3kDa membrane in DCM/MeOH mixture 1:1 and obtained as a brownish amorphous solid (622 mg, 99 %). **^{1}H NMR** (400 MHz, $CDCl_3$, ^{1}H-^{1}H COSY): δ 7.75 (m, 8H, $HNCOC_{q(Ph)}CH_{Ph}CH_{Ph}$), 6.99–6.89 (m, 88H, N*H*, 168H, $HNCOC_{q(Ph)}CH_{Ph}C_{q(Ph)}$, 8H, $HNCOC_{q(Ph)}CH_{Ph}CH_{Ph}$), 6.52 (t, *J* = 2.2 Hz, 84H, CH_{Ph}), 5.76 (ddt, *J* = 16.7, 10.1, 8.1 Hz, 432H, $CHCH_2$), 4.90–4.84 (m, 864H, $CHCH_2$), 3.85 (t, *J* = 6.8 Hz, 344H, OCH_2), 3.56 (td, *J* = 6.8, 5.7 Hz, 176H, CH_2NH), 2.71 (t, *J* = 6.8 Hz, 176H, SCH_2CH_2NH), 2.52 (t, *J* = 7.3 Hz, 176H, $SCH_2(CH_2)_2Si$), 1.79–1.71 (m, 344H, $OCH_2CH_2CH_2Si$), 1.59 (dt, *J* = 8.1, 1.1 Hz, 864H, $SiCH_2CH$), 1.60–1.58 (m, 176H, $SiCH_2CH_2CH_2S$), 0.67–0.63 (m, 520H, $SiCH_2$). **^{13}C {^{1}H} NMR** (101 MHz, $CDCl_3$, ^{1}H-^{13}C HSQC, ^{1}H-^{13}C HMBC): δ 167.4 ($HNCOC_{q(Ph)}CH_{Ph}C_{q(Ph)}$), not detected ($HNCOC_{q(Ph)}(CH_{Ph})_2$), from HMB 161.7 ($(CH_{Ph})_2C_{q(Ph)}OCH_2$), 160.3 ($C_{q(Ph)}CH_{Ph}C_{q(Ph)}OCH_2$), 136.5 ($HNCOC_{q(Ph)}CH_{Ph}C_{q(Ph)}$), 134.2 ($CHCH_2$), 128.9 ($HNCOC_{q(Ph)}CH_{Ph}CH_{Ph}$), 126.5 ($HNCOC_{q(Ph)}(CH_{Ph})_2$), 114.3 ($CH_{Ph}CH_{Ph}C_{q(Ph)}OCH_2$), 114.0 ($CHCH_2$), 105.5 ($HNCOC_{q(Ph)}CH_{Ph}C_{q(Ph)}$), 104.6 ($CH_2OC_{q(Ph)}CH_{Ph}C_{q(Ph)}OCH_2$), 70.8 ($OCH_2(CH_2)_2Si$), 39.4 ($CH_2NH$), 35.6 ($Si(CH_2)_2CH_2S$), 31.7 ($SCH_2CH_2NH$), 24.4 ($SiCH_2CH_2CH_2S$), 23.8 ($OCH_2CH_2CH_2Si^1$), 23.4 ($OCH_2CH_2CH_2Si^{2/3}$), 19.6 ($SiCH_2CH$), 11.8 ($SiCH_2(CH_2)_2S$), 8.4 ($O(CH_2)_2CH_2Si^1$), 7.6 ($O(CH_2)_2CH_2Si^{2/3}$). **^{29}Si {^{1}H} NMR** (79 MHz, $CDCl_3$): δ not detected or overlapped ($Si^{0/1}$), 4.06 (Si^2), –0.13 (Si^3).

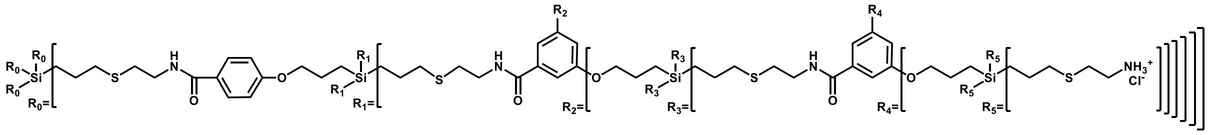

G_3-3-6-6-N. Dendrimer G_3-3-6-6-N was prepared from 198 mg (3.81 µmol) of dendrimer G_3-3-6-6-A according to General procedure GP2. The product was purified by OSN using 3kDa membrane in MeOH followed by lyophilization and obtained as an off-white powder (329 mg, 85 %). **^{1}H NMR** (400 MHz, **100 °C**, DMSO-d_6, ^{1}H-^{1}H COSY): δ 8.55 (s, 88H, N*H*), 8.34 (br s, 1296H, NH_3^+), 7.83 (m, 8H, $NHCOC_{q(Ph)}CH_{Ph}CH_{Ph}$), 7.06 (s, 168H, $NHCOC_{q(Ph)}CH_{Ph}C_{q(Ph)}$), 6.95 (m, 8H, $CH_{Ph}CH_{Ph}C_{q(Ph)}OCH_2$), 6.59 (s, 84H, $NHCOC_{q(Ph)}CH_{Ph}C_{q(Ph)}OCH_2$), 3.99 (t, *J* = 6.5 Hz, 344H, OCH_2), 3.46–3.44 (m, 176H, CH_2NH), 3.01 (t, *J* = 7.4 Hz, 864H, $CH_2NH_3^+$), 2.86 (t, *J* = 7.4 Hz, 864H, $CH_2CH_2NH_3^+$), 2.73 (t, *J* = 7.3 Hz, 176H, CH_2CH_2NH), 2.60 (t, *J* = 7.1 Hz, 1040H, $SCH_2(CH_2)_2Si$), 1.78–1.72 (m, 344H, $OCH_2CH_2CH_2Si$), 1.64–1.56 (m, 1040H, $SiCH_2CH_2CH_2S$), 0.72–0.66 (m, 1384H, $SiCH_2$). **^{1}H NMR** (400 MHz, DMSO-d_6, ^{1}H-^{1}H COSY): δ 8.88 (br s, 88H, N*H*), 8.35 (br s, 1296H, NH_3^+), 7.82 (m, 8H, $NHCOC_{q(Ph)}CH_{Ph}CH_{Ph}$), 7.04 (s, 168H, $NHCOC_{q(Ph)}CH_{Ph}C_{q(Ph)}$), 6.93 (m, 8H, $CH_{Ph}CH_{Ph}C_{q(Ph)}OCH_2$), 6.56 (s, 84H, $NHCOC_{q(Ph)}CH_{Ph}C_{q(Ph)}OCH_2$), 3.93 (br s, 344H, OCH_2), overlapped with water (br s, 176H, CH_2NH), 2.94 (t, *J* = 7.4 Hz, 864H, $CH_2NH_3^+$), 2.76 (t, *J* = 7.4 Hz, 864H, $CH_2CH_2NH_3^+$), 2.63 (br s, 176H, CH_2CH_2NH), 2.54 (t, *J* = 7.7 Hz, 1040H, $SCH_2(CH_2)_2Si$), 1.69 (br s, 344H, $OCH_2CH_2CH_2Si$), 1.51 (br s, 1040H, $SiCH_2CH_2CH_2S$), 0.63–0.60 (m, 1384H, $SiCH_2$). **^{13}C {^{1}C} NMR** (101 MHz, DMSO-d_6, ^{1}H-^{13}C HSQC, ^{1}H-^{13}C HMBC): δ 165.6 ($HNCOC_{q(Ph)}CH_{Ph}C_{q(Ph)}$), not detected ($HNCOC_{q(Ph)}(CH_{Ph})_2$), not detected ($(CH_{Ph})_2C_{q(Ph)}OCH_2$), 159.6 ($C_{q(Ph)}(CH_{Ph})C_{q(Ph)}OCH_2$), 136.1 ($HNCOC_{q(Ph)}CH_{Ph}C_{q(Ph)}$), from HSQC 130.1 ($HNCOC_{q(Ph)}CH_{Ph}CH_{Ph}$), not detected ($HNCOC_{q(Ph)}(CH_{Ph})_2$), from HSQC 114.0 ($CH_{Ph}CH_{Ph}C_{q(Ph)}OCH_2$), 105.6 ($HNCOC_{q(Ph)}CH_{Ph}C_{q(Ph)}$), 103.9 ($OC_{q(Ph)}CH_{Ph}C_{q(Ph)}O$), 70.4 ($OCH_2(CH_2)_2Si$), from HSQC 39.7 ($CH_2NH$), 38.9 ($CH_2NH_3^+$), 34.9 ($CH_2S(CH_2)_2NH$), 34.5 ($CH_2S(CH_2)_2NH_3^+$), 30.4 ($CH_2CH_2NH$), 27.8 ($CH_2CH_2NH_3^+$), 23.7 ($CH_2CH_2S(CH_2)_2NH$), 23.3 ($CH_2CH_2O$), 11.2 ($SiCH_2(CH_2)_2S$), 7.8 ($SiCH_2(CH_2)_2O$). **^{29}Si {^{1}H} NMR** (79 MHz, DMSO-d_6): δ 4.00 (Si^3), not detected or overlapped ($Si^{0/1/2}$).

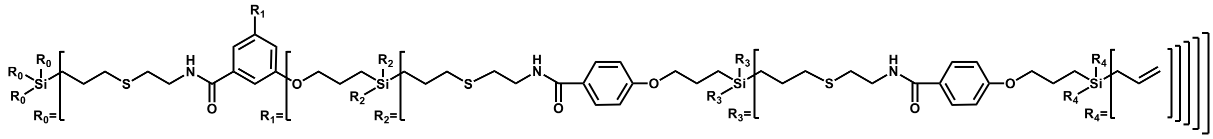

G_3-6-3-3-A. Dendrimer G_3-6-3-3-A was prepared from 193 mg (9.59 µmol) of G_2-6-3-N according to General procedure GP1. The product was purified by OSN using 3kDa membrane in DCM/MeOH mixture 2:3 and obtained as a yellowish foam (359 mg, 93 %). **^{1}H NMR** (400 MHz, $CDCl_3$, ^{1}H-^{1}H COSY): δ 7.76 (m, 192H, $HNCOC_{q(Ph)}CH_{Ph}CH_{Ph}$), 7.36 (br s, 16H, N*H*), 7.10 (br s, 72H, N*H*), 6.98 (br s, 8H, $HNCOC_{q(Ph)}CH_{Ph}C_{q(Ph)}$), 6.84 (m, 192H, $HNCOC_{q(Ph)}CH_{Ph}CH_{Ph}$), 6.53 (br s, 4H, $CH_2OC_{q(Ph)}CH_{Ph}C_{q(Ph)}OCH_2$), 5.77 (ddt, *J* = 16.4, 10.0, 8.1 Hz, 216H, C*H*CH_2), 4.91–4.84 (m, 432H, CHC*H*$_2$), 3.89 (t, *J* = 6.8 Hz, 208H, OC*H*$_2$), 3.56 (td, *J* = 6.8, 5.7 Hz, 200H, C*H*$_2$NH), 2.70 (t, *J* = 6.8 Hz, 200H, SCH_2CH_2NH), 2.51 (t, *J* = 7.1 Hz, 200H, $SCH_2(CH_2)_2Si$), 1.83–1.74 (m, 208H, $OCH_2CH_2CH_2Si$), 1.60 (dt, *J* = 8.1, 1.1 Hz, 432H, $SiCH_2CH$), 1.59–1.54 (m, 200H, $SiCH_2CH_2CH_2S$), 0.70–0.66 (m, 192H, $(O(CH_2)_2CH_2Si^{2/3})$), 0.62–0.60 (m, 216H, $SiCH_2(CH_2)_2S$, $(O(CH_2)_2CH_2Si^1)$. **^{13}C {^{1}H} NMR** (101 MHz, $CDCl_3$, ^{1}H-^{13}C HSQC, ^{1}H-^{13}C HMBC): δ 167.3 ($HNCOC_{q(Ph)}CH_{Ph}C_{q(Ph)}$), 167.2 ($HNCOC_{q(Ph)}(CH_{Ph})_2$), 161.8, 161.7 ($(CH_{Ph})_2C_{q(Ph)}OCH_2$), not detected ($C_{q(Ph)}CH_{Ph}C_{q(Ph)}OCH_2$), not detected ($HNCOC_{q(Ph)}CH_{Ph}C_{q(Ph)}$), 134.2 ($CHCH_2$), 129.1, 129.0 ($HNCOC_{q(Ph)}CH_{Ph}CH_{Ph}$), 126.6, 126.5 ($HNCOC_{q(Ph)}(CH_{Ph})_2$), 114.3 ($CH_{Ph}CH_{Ph}C_{q(Ph)}OCH_2$), 114.0 ($CHCH_2$), not detected ($HNCOC_{q(Ph)}CH_{Ph}C_{q(Ph)}$), not detected ($CH_2OC_{q(Ph)}CH_{Ph}C_{q(Ph)}OCH_2$), 70.7 ($OCH_2(CH_2)_2Si$), 39.4 ($CH_2NH$), 35.7 ($Si(CH_2)_2CH_2S$), 31.9 ($SCH_2CH_2NH$), 24.4 ($SiCH_2CH_2CH_2S$), 23.4 ($OCH_2CH_2CH_2Si$), 19.6 ($SiCH_2CH$), 11.6 ($SiCH_2(CH_2)_2S$), 8.3 ($O(CH_2)_2CH_2Si^1$), 7.6 ($O(CH_2)_2CH_2Si^{2/3}$). **^{29}Si {^{1}H} NMR** (79 MHz, $CDCl_3$): δ not detected (Si^0), 4.03 (Si^2), 4.00 (Si^1), –0.11 (Si^3).

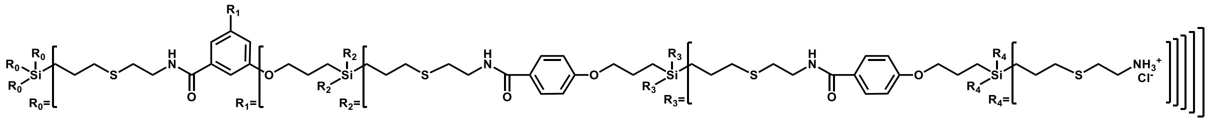

G_3-6-3-3-N. Dendrimer G_3-6-3-3-N was prepared from 208 mg (5.20 µmol) of dendrimer G_3-6-3-3-A according to General procedure GP2. The product was purified by OSN using 3kDa membrane in MeOH followed by lyophilization and obtained as a white powder (290 mg, 86 %). **^{1}H NMR** (400 MHz, **100 °C**, DMSO-*d*$_6$, ^{1}H-^{1}H COSY): δ 8.47 (br s, 4H, $NHCOC_{q(Ph)}CH_{Ph}C_{q(Ph)}$), 8.30 (br s, 96H, $NHCOC_{q(Ph)}(CH_{Ph})_2$, 648H, NH_3Cl), 7.85 (m, 192H, $NHCOC_{q(Ph)}CH_{Ph}CH_{Ph}$), 7.05 (8H, $NHCOC_{q(Ph)}CH_{Ph}C_{q(Ph)}$), 6.93 (m, 192H, $CH_{Ph}CH_{Ph}C_{q(Ph)}OCH_2$), 6.63 (4H, $NHCOC_{q(Ph)}CH_{Ph}C_{q(Ph)}OCH_2$), 3.99, 3.95 (2×t, *J* = 6.4 Hz, 208H, OC*H*$_2$), 3.44 (td, *J* = 7.3, 5.8 Hz, 200H, C*H*$_2$NHCO), 2.99 (t, *J* = 7.4 Hz, 432H, $CH_2NH_3^+$), 2.84 (t, *J* = 7.4 Hz, 432H, $CH_2CH_2NH_3^+$), 2.70 (t, *J* = 7.3 Hz, 200H, CH_2CH_2NH), 2.58 (t, *J* = 7.1 Hz, 632H, $SCH_2(CH_2)_2Si$), 1.78–1.72 (m, 208H, $OCH_2CH_2CH_2Si$), 1.63–1.55 (m, 632H, $SiCH_2CH_2CH_2S$), 0.70–0.64 (m, 840H, $SiCH_2$). **^{1}H NMR** (400 MHz, DMSO-*d*$_6$, ^{1}H-^{1}H COSY): δ 8.79 (br s, 4H, $NHCOC_{q(Ph)}CH_{Ph}C_{q(Ph)}$), 8.64 (br s, 96H, $NHCOC_{q(Ph)}(CH_{Ph})_2$), 8.31 (br s, 648H, NH_3Cl), 7.85 (m, 192H, $NHCOC_{q(Ph)}CH_{Ph}CH_{Ph}$), not detected (8H, $NHCOC_{q(Ph)}CH_{Ph}C_{q(Ph)}$), 6.93 (m, 192H, $CH_{Ph}CH_{Ph}C_{q(Ph)}OCH_2$), not detected (4H, $NHCOC_{q(Ph)}CH_{Ph}C_{q(Ph)}OCH_2$), 3.93 (br s, 208H, OC*H*$_2$), overlapped with water (C*H*$_2$NHCO), 2.94 (t, *J* = 7.4 Hz, 432H, $CH_2NH_3^+$), 2.76 (t, *J* = 7.4 Hz, 432H, $CH_2CH_2NH_3^+$), 2.63 (t, *J* = 7.3 Hz, 200H, CH_2CH_2NH), 2.54 (t, *J* = 7.3 Hz, 632H, $SCH_2(CH_2)_2Si$), 1.67 (br s, 208H, $OCH_2CH_2CH_2Si$), 1.55–1.47 (m, 632H, $SiCH_2CH_2CH_2S$), 0.64–0.60 (m, 840H, $SiCH_2$). **^{13}C {^{1}H} NMR** (101 MHz, DMSO-*d*$_6$, ^{1}H-^{13}C HSQC, ^{1}H-^{13}C HMBC): δ 165.6 ($HNCOC_{q(Ph)}(CH_{Ph})_2$), not detected ($HNCOC_{q(Ph)}CH_{Ph}C_{q(Ph)}$), 160.9 ($(CH_{Ph})_2C_{q(Ph)}OCH_2$), not detected ($C_{q(Ph)}(CH_{Ph})C_{q(Ph)}OCH_2$), not detected ($HNCOC_{q(Ph)}CH_{Ph}C_{q(Ph)}$), 129.1 ($HNCOC_{q(Ph)}CH_{Ph}CH_{Ph}$), 126.3 ($HNCOC_{q(Ph)}(CH_{Ph})_2$), 113.8 ($CH_{Ph}CH_{Ph}C_{q(Ph)}OCH_2$), not detected ($HNCOC_{q(Ph)}CH_{Ph}C_{q(Ph)}$), not detected ($OC_{q(Ph)}CH_{Ph}C_{q(Ph)}O$), 70.2 ($OCH_2(CH_2)_2Si$), from HSQC 39.4 ($CH_2NH$), 38.7 ($CH_2NH_3^+$), 34.8 ($CH_2S(CH_2)_2NH$), 34.5 ($CH_2S(CH_2)_2NH_3^+$), 30.5 ($CH_2CH_2NH$), 27.8 ($CH_2CH_2NH_3^+$), 23.7 ($CH_2CH_2S(CH_2)_2NH$), 23.2 ($CH_2CH_2OC_{q(Ph)}$), 11.1 ($SiCH_2(CH_2)_2S$), 7.7 ($SiCH_2(CH_2)_2O$). **^{29}Si {^{1}H} NMR** (79 MHz, DMSO-*d*$_6$): δ 4.02 (Si^3), 3.96 (Si^2), not detected or overlapped ($Si^{0/1}$).

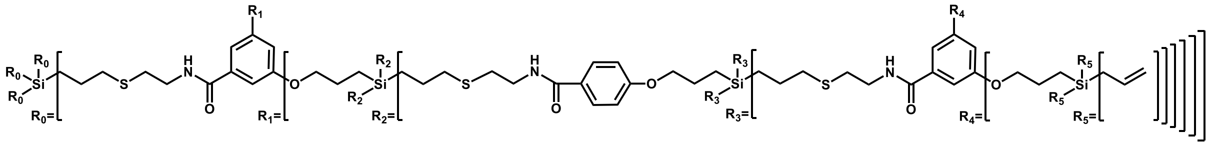

G_3-6-3-6-A. Dendrimer G_3-6-3-6-A was prepared from 121 mg (6.02 µmol) of G_2-6-3-N according to General procedure GP1. The product was purified by OSN using 3kDa membrane in DCM/MeOH mixture 1:1 and obtained as a yellowish amorphous substance (266 mg, 80 %). **^{1}H NMR** (400 MHz, $CDCl_3$, ^{1}H-^{1}H COSY): δ 7.74 (m, 48H, $HNCOC_{q(Ph)}C\textit{H}_{Ph}CH_{Ph}$), 7.09 (br s, 24H, N*H*), 6.99 (br s, 76H, N*H*), 6.91 (d, *J* = 2.2 Hz, 152H, $HNCOC_{q(Ph)}C\textit{H}_{Ph}C_{q(Ph)}$), 6.84 (m, 48H, $HNCOC_{q(Ph)}CH_{Ph}C\textit{H}_{Ph}$), 6.52 (t, *J* = 2.2 Hz, 76H, $CH_2OC_{q(Ph)}C\textit{H}_{Ph}C_{q(Ph)}OCH_2$), 5.76 (ddt, *J* = 16.7, 10.1, 8.1 Hz, 432H, C*H*CH_2), 4.90–4.84 (m, 864H, $CHC\textit{H}_2$), 3.88, 3.85 (2×t, *J* = 6.8 Hz, 352H, $OC\textit{H}_2$), 3.57 (td, *J* = 6.8, 5.7 Hz, 200H, $C\textit{H}_2NH$), 2.71 (t, *J* = 6.8 Hz, 200H, $SC\textit{H}_2CH_2NH$), 2.53 (t, *J* = 7.2 Hz, 200H, $SC\textit{H}_2(CH_2)_2Si$), 1.79–1.72 (m, 352H, $OCH_2C\textit{H}_2CH_2Si$), 1.59 (dt, *J* = 8.1, 1.1 Hz, 864H, $SiC\textit{H}_2CH$), 1.59–1.52 (m, 200H, $SiCH_2C\textit{H}_2CH_2S$), 0.67–0.63 (m, 552H, $SiC\textit{H}_2$). **^{13}C {^{1}H} NMR** (101 MHz, $CDCl_3$, ^{1}H-^{13}C HSQC, ^{1}H-^{13}C HMBC): δ 167.4 ($HN\textit{C}OC_{q(Ph)}CH_{Ph}C_{q(Ph)}$), 167.2 ($HN\textit{C}OC_{q(Ph)}(CH_{Ph})_2$), 161.7 ($(CH_{Ph})_2\textit{C}_{q(Ph)}OCH_2$), 160.4 ($C_{q(Ph)}CH_{Ph}\textit{C}_{q(Ph)}OCH_2$), 136.5 ($HNCO\textit{C}_{q(Ph)}CH_{Ph}C_{q(Ph)}$), 134.2 (*C*$HCH_2$), 129.1 ($HNCOC_{q(Ph)}\textit{C}H_{Ph}CH_{Ph}$), 126.6 ($HNCO\textit{C}_{q(Ph)}(CH_{Ph})_2$), 114.3 ($CH_{Ph}\textit{C}H_{Ph}C_{q(Ph)}OCH_2$), 114.0 ($CH\textit{C}H_2$), 105.6 ($HNCOC_{q(Ph)}\textit{C}H_{Ph}C_{q(Ph)}$), 104.6 ($CH_2OC_{q(Ph)}\textit{C}H_{Ph}C_{q(Ph)}OCH_2$), 70.8 ($O\textit{C}H_2(CH_2)_2Si$), 39.4 ($\textit{C}H_2NH$), 35.6 ($Si(CH_2)_2\textit{C}H_2S$), 31.8 ($S\textit{C}H_2CH_2NH$), 24.4 ($SiCH_2\textit{C}H_2CH_2S$), 23.8 ($OCH_2\textit{C}H_2CH_2Si^{2}$), 23.4 ($OCH_2\textit{C}H_2CH_2Si^{1/3}$), 19.6 ($Si\textit{C}H_2CH$), 11.8 ($Si\textit{C}H_2(CH_2)_2S$), 8.4 ($O(CH_2)_2\textit{C}H_2Si^{1}$), 7.6 ($O(CH_2)_2\textit{C}H_2Si^{2/3}$). **^{29}Si {^{1}H} NMR** (79 MHz, $CDCl_3$): δ not detected or overlapped ($Si^{0/1}$), 4.07 (Si^{2}), –0.12 (Si^{3}).

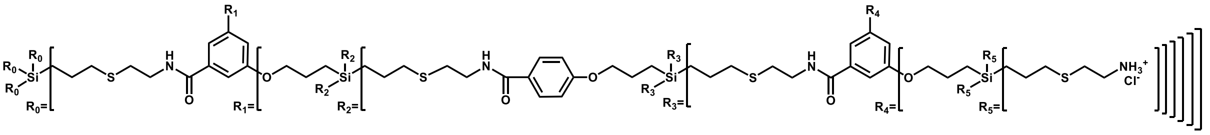

G_3-6-3-6-N. Dendrimer G_3-6-3-6-N was prepared from 191 mg (3.47 µmol) of dendrimer G_3-6-3-6-A according to General procedure GP2. The product was purified by OSN using 3kDa membrane in MeOH followed by lyophilization and obtained as an off-white powder (303 mg, 83 %). **^{1}H NMR** (400 MHz, **100 °C,** DMSO-*d*$_6$, ^{1}H-^{1}H COSY): δ 8.53 (br s, 24H, $N\textit{H}COC_{q(Ph)}(CH_{Ph})_2$), 8.33 (br s, 76H, $N\textit{H}COC_{q(Ph)}CH_{Ph}C_{q(Ph)}$, 1296H, $N\textit{H}_3^+$), 7.87 (br s, 48H, $NHCOC_{q(Ph)}C\textit{H}_{Ph}CH_{Ph}$), 7.06 (br s, 152H, $NHCOC_{q(Ph)}C\textit{H}_{Ph}C_{q(Ph)}$), 6.94 (br s, 48H, $CH_{Ph}C\textit{H}_{Ph}C_{q(Ph)}OCH_2$), 6.59 (br s, 76H, $NHCOC_{q(Ph)}C\textit{H}_{Ph}C_{q(Ph)}OCH_2$), 3.99 (t, *J* = 6.7 Hz, 352H, $OC\textit{H}_2$), 3.45 (br s, 200H, $C\textit{H}_2NH$), 3.01 (t, *J* = 7.4 Hz, 864H, $C\textit{H}_2NH_3^+$), 2.85 (t, *J* = 7.4 Hz, 864H, $C\textit{H}_2CH_2NH_3^+$), 2.72 (t, *J* = 7.4 Hz, 200H, $C\textit{H}_2CH_2NH$), 2.59 (t, *J* = 7.1 Hz, 864H, $SC\textit{H}_2(CH_2)_2Si$), 1.74–1.72 (m, 352H, $OCH_2C\textit{H}_2CH_2Si$), 1.64–1.56 (m, 1064H, $SiCH_2C\textit{H}_2CH_2S$), 0.71–0.67 (m, 1416H, $SiC\textit{H}_2$). **^{1}H NMR** (400 MHz, DMSO-*d*$_6$, ^{1}H-^{1}H COSY): δ 8.84 (br s, 24H, $N\textit{H}COC_{q(Ph)}(CH_{Ph})_2$), 8.35 (br s, 76H, $N\textit{H}COC_{q(Ph)}CH_{Ph}C_{q(Ph)}$, 1296H, $N\textit{H}_3^+$), 7.86 (br s, 48H, $NHCOC_{q(Ph)}C\textit{H}_{Ph}CH_{Ph}$), 7.05 (br s, 152H, $NHCOC_{q(Ph)}C\textit{H}_{Ph}C_{q(Ph)}$), 6.94 (br s, 48H, $CH_{Ph}C\textit{H}_{Ph}C_{q(Ph)}OCH_2$), 6.57 (br s, 76H, $NHCOC_{q(Ph)}C\textit{H}_{Ph}C_{q(Ph)}OCH_2$), 3.94 (br s, 352H, $OC\textit{H}_2$), overlapped with water (br s, 200H, $C\textit{H}_2NH$), 2.95 (t, *J* = 7.4 Hz, 864H, $C\textit{H}_2NH_3^+$), 2.78 (t, *J* = 7.4 Hz, 864H, $C\textit{H}_2CH_2NH_3^+$), 2.64 (br s, 200H, $C\textit{H}_2CH_2NH$), 2.55 (t, *J* = 7.0 Hz, 864H, $SC\textit{H}_2(CH_2)_2Si$), 1.67 (br s, 352H, $OCH_2C\textit{H}_2CH_2Si$), 1.52 (br s, 1064H, $SiCH_2C\textit{H}_2CH_2S$), 0.62 (m, 1416H, $SiC\textit{H}_2$). **^{13}C {^{1}H} NMR** (101 MHz, DMSO-*d*$_6$, ^{1}H-^{13}C HSQC, ^{1}H-^{13}C HMBC): δ not detected ($HN\textit{C}OC_{q(Ph)}(CH_{Ph})_2$), 165.6 ($HN\textit{C}OC_{q(Ph)}CH_{Ph}C_{q(Ph)}$), not detected ($(CH_{Ph})_2\textit{C}_{q(Ph)}OCH_2$), 159.6 ($C_{q(Ph)}(CH_{Ph})\textit{C}_{q(Ph)}OCH_2$), 136.2 ($HNCO\textit{C}_{q(Ph)}CH_{Ph}C_{q(Ph)}$), 129.1 ($HNCOC_{q(Ph)}\textit{C}H_{Ph}CH_{Ph}$), 126.3 ($HNCO\textit{C}_{q(Ph)}(CH_{Ph})_2$), 113.8 ($CH_{Ph}\textit{C}H_{Ph}C_{q(Ph)}OCH_2$), 105.7 ($HNCOC_{q(Ph)}\textit{C}H_{Ph}C_{q(Ph)}$), 103.9 ($OC_{q(Ph)}\textit{C}H_{Ph}C_{q(Ph)}O$), 70.4 ($O\textit{C}H_2(CH_2)_2Si$), from HSQC 39.6 ($\textit{C}H_2NH$), 38.7 ($\textit{C}H_2NH_3^+$), 34.9 ($\textit{C}H_2S(CH_2)_2NH$), 34.5 ($\textit{C}H_2S(CH_2)_2NH_3^+$), 30.4 ($\textit{C}H_2CH_2NH$), 27.8 ($\textit{C}H_2CH_2NH_3^+$), 23.7 ($\textit{C}H_2CH_2S(CH_2)_2NH$), 23.3 ($\textit{C}H_2CH_2O$), 11.2

(Si*C*H$_2$(CH$_2$)$_2$S), 7.8 (Si*C*H$_2$(CH$_2$)$_2$O). **^{29}Si {^{1}H} NMR** (79 MHz, DMSO-*d*$_6$): *δ* 4.01 (Si^3), not detected or overlapped (Si$^{0/1/2}$).

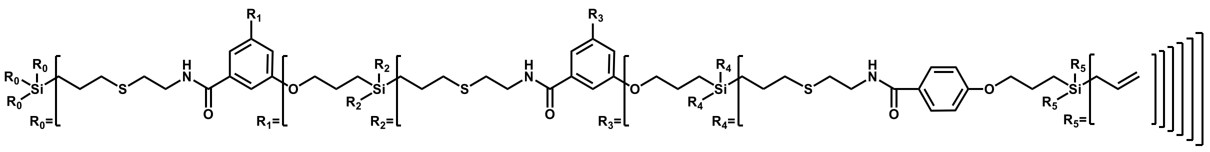

G$_3$-6-6-3-A. Dendrimer G$_3$-6-6-3-A was prepared from 125 mg (3.76 µmol) of G$_2$-6-6-N according to General procedure GP1. The product was purified by OSN using 3kDa membrane in DCM/MeOH mixture, starting at 1:1 ratio and gradually increasing the polarity up to 3:5 so as to maintain the homogeneity of the solution, and obtained as brownish foam (235 mg, 85 %). **^{1}H NMR** (400 MHz, CDCl$_3$, ^{1}H-^{1}H COSY): *δ* 7.76 (m, 288H, HNCOC$_{q(Ph)}$C*H*$_{Ph}$CH$_{Ph}$), 7.37 (br s, 152H, N*H*), 7.00 (br s, 56H, HNCOC$_{q(Ph)}$C*H*$_{Ph}$C$_{q(Ph)}$), 6.81 (m, 288H, HNCOC$_{q(Ph)}$CH$_{Ph}$C*H*$_{Ph}$), 6.52 (br s, 28H, CH$_2$OC$_{q(Ph)}$C*H*$_{Ph}$C$_{q(Ph)}$OCH$_2$), 5.76 (ddt, *J* = 16.7, 10.1, 8.1 Hz, 432H, C*H*CH$_2$), 4.89–4.83 (m, 864H, CHC*H*$_2$), 3.87 (t, *J* = 6.8 Hz, 400H, OC*H*$_2$), 3.54 (td, *J* = 6.7, 5.7 Hz, 344H, C*H*$_2$N), 2.69 (t, *J* = 6.7 Hz, 344H, SC*H*$_2$CH$_2$NH), 2.49 (t, *J* = 6.9 Hz, 344H, SC*H*$_2$(CH$_2$)$_2$Si), 1.81–1.73 (m, 400H, OCH$_2$C*H*$_2$CH$_2$Si), 1.59 (dt, *J* = 8.1, 1.1 Hz, 864H, SiC*H*$_2$CH), 1.59–1.52 (m, 344H, SiCH$_2$C*H*$_2$CH$_2$S), 0.68–0.59 (m, 744H, SiC*H*$_2$). **^{13}C {^{1}H} NMR** (101 MHz, CDCl$_3$, ^{1}H-^{13}C HSQC, ^{1}H-^{13}C HMBC): *δ* 167.3 (HN*C*OC$_{q(Ph)}$CH$_{Ph}$C$_{q(Ph)}$, HN*C*OC$_{q(Ph)}$(CH$_{Ph}$)$_2$), 161.7 ((CH$_{Ph}$)$_2$*C*$_{q(Ph)}$OCH$_2$), 160.3 (C$_{q(Ph)}$CH$_{Ph}$*C*$_{q(Ph)}$OCH$_2$), 136.5 (HNCO*C*$_{q(Ph)}$CH$_{Ph}$C$_{q(Ph)}$), 134.2 (*C*HCH$_2$), 129.1 (HNCOC$_{q(Ph)}$*C*H$_{Ph}$CH$_{Ph}$), 126.5 (HNCO*C*$_{q(Ph)}$(CH$_{Ph}$)$_2$), 114.3 (CH$_{Ph}$*C*H$_{Ph}$C$_{q(Ph)}$OCH$_2$), 114.0 (CH*C*H$_2$), 105.8 (HNCOC$_{q(Ph)}$*C*H$_{Ph}$C$_{q(Ph)}$), 104.7 (CH$_2$OC$_{q(Ph)}$*C*H$_{Ph}$C$_{q(Ph)}$OCH$_2$), 70.6 (O*C*H$_2$(CH$_2$)$_2$Si), 39.7 (*C*H$_2$NH), 35.7 (Si(CH$_2$)$_2$*C*H$_2$S), 31.8 (S*C*H$_2$CH$_2$NH), 24.4 (SiCH$_2$*C*H$_2$CH$_2$S), 23.8 (OCH$_2$*C*H$_2$CH$_2$Si$^{1/2}$), 23.4 (OCH$_2$*C*H$_2$CH$_2$Si^3), 19.6 (Si*C*H$_2$CH), 11.8 (Si*C*H$_2$(CH$_2$)$_2$S), 8.3 (O(CH$_2$)$_2$*C*H$_2$Si$^{1/2}$), 7.6 (O(CH$_2$)$_2$*C*H$_2$Si^3). **^{29}Si {^{1}H} NMR** (79 MHz, CDCl$_3$): *δ* 4.11 (Si^1), 3.99 (Si^2), –0.12 (Si^3), not detected or overlapped (Si^0).

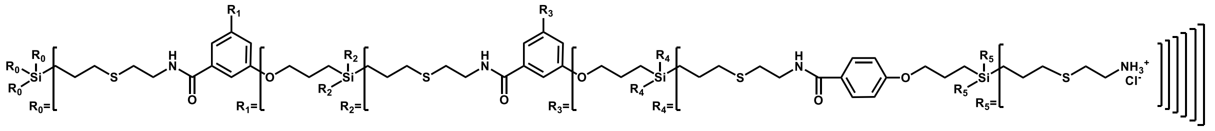

G$_3$-6-6-3-N. Dendrimer G$_3$-6-6-3-N was prepared from 187 mg (2.56 µmol) of dendrimer G$_3$-6-6-3-A according to General procedure GP2. The product was purified by OSN using 3kDa membrane in MeOH followed by lyophilization and obtained as off-white powder (298 mg, 95 %). **^{1}H NMR** (400 MHz, **100 °C**, DMSO-*d*$_6$, ^{1}H-^{1}H COSY): *δ* 8.51 (br s, 28H, N*H*COC$_{q(Ph)}$CH$_{Ph}$C$_{q(Ph)}$), 8.33 (br s, 144H, N*H*COC$_{q(Ph)}$(CH$_{Ph}$)$_2$, 1296H, N*H*$_3^+$), 7.86 (m, 288H, NHCOC$_{q(Ph)}$C*H*$_{Ph}$CH$_{Ph}$), 7.04 (s, 56H, NHCOC$_{q(Ph)}$C*H*$_{Ph}$C$_{q(Ph)}$), 6.92 (m, 288H, CH$_{Ph}$C*H*$_{Ph}$C$_{q(Ph)}$OCH$_2$), 6.59 (28H, NHCOC$_{q(Ph)}$C*H*$_{Ph}$C$_{q(Ph)}$OCH$_2$), 3.97 (br s, 400H, OC*H*$_2$), 3.44 (dt, *J* = 7.0, 6.5 Hz, 344H, C*H*$_2$NH), 3.00 (t, *J* = 7.4 Hz, 864H, C*H*$_2$NH$_3^+$), 2.85 (t, *J* = 7.4 Hz, 864H, C*H*$_2$CH$_2$NH$_3^+$), 2.69 (t, *J* = 7.0 Hz, 344H, C*H*$_2$CH$_2$NH), 2.58 (t, *J* = 7.1 Hz, 1208H, SC*H*$_2$(CH$_2$)$_2$Si), 1.73 (br s, 400H, OCH$_2$C*H*$_2$CH$_2$Si), 1.62–1.55 (m, 1208H, SiCH$_2$C*H*$_2$CH$_2$S), 0.70–0.63 (m, 1608H, SiC*H*$_2$). **^{1}H NMR** (400 MHz, DMSO-*d*$_6$, ^{1}H-^{1}H COSY): *δ* 8.65 (br s, 28H, N*H*COC$_{q(Ph)}$CH$_{Ph}$C$_{q(Ph)}$, 144H, N*H*COC$_{q(Ph)}$(CH$_{Ph}$)$_2$), 8.34 (s, 1296H, N*H*$_3^+$), 7.86 (m, 288H, NHCOC$_{q(Ph)}$C*H*$_{Ph}$CH$_{Ph}$), 6.91 (br s, 56H, NHCOC$_{q(Ph)}$C*H*$_{Ph}$C$_{q(Ph)}$, 288H, CH$_{Ph}$C*H*$_{Ph}$C$_{q(Ph)}$OCH$_2$), 6.58 (28H, NHCOC$_{q(Ph)}$C*H*$_{Ph}$C$_{q(Ph)}$OCH$_2$), 3.91 (br s, 400H, OC*H*$_2$), overlapped with water (C*H*$_2$NH), 2.94 (t, *J* = 7.4 Hz, 864H, C*H*$_2$NH$_3^+$), 2.77 (t, *J* = 7.4 Hz, 864H, C*H*$_2$CH$_2$NH$_3^+$), 2.62 (br s, 344H, C*H*$_2$CH$_2$NH), 2.54 (br s, 1208H, SC*H*$_2$(CH$_2$)$_2$Si), 1.63 (br s, 400H, OCH$_2$C*H*$_2$CH$_2$Si), 1.50 (br s, 1208H, SiCH$_2$C*H*$_2$CH$_2$S), 0.60 (br s, 1608H, SiC*H*$_2$). **^{13}C {^{1}H} NMR** (101 MHz, DMSO-*d*$_6$, ^{1}H-^{13}C HSQC, ^{1}H-^{13}C HMBC): *δ* 165.6 (HN*C*OC$_{q(Ph)}$(CH$_{Ph}$)$_2$), not detected (HN*C*OC$_{q(Ph)}$CH$_{Ph}$C$_{q(Ph)}$), 160.9 ((CH$_{Ph}$)$_2$*C*$_{q(Ph)}$OCH$_2$), 159.6 (C$_{q(Ph)}$(CH$_{Ph}$)*C*$_{q(Ph)}$OCH$_2$), not detected (HNCO*C*$_{q(Ph)}$CH$_{Ph}$C$_{q(Ph)}$), 129.1 (HNCOC$_{q(Ph)}$*C*H$_{Ph}$CH$_{Ph}$), 126.3 (HNCO*C*$_{q(Ph)}$(CH$_{Ph}$)$_2$), 113.8 (CH$_{Ph}$*C*H$_{Ph}$C$_{q(Ph)}$OCH$_2$), not detected (HNCOC$_{q(Ph)}$*C*H$_{Ph}$C$_{q(Ph)}$), not detected (OC$_{q(Ph)}$*C*H$_{Ph}$C$_{q(Ph)}$O), 70.2 (O*C*H$_2$(CH$_2$)$_2$Si), from HSQC 39.5

(CH$_2$NHCO), 38.7 (CH$_2$NH$_3^+$), 34.9 (CH$_2$S(CH$_2$)$_2$NH), 34.5 (CH$_2$S(CH$_2$)$_2$NH$_3^+$), 30.7 (CH$_2$CH$_2$NH), 27.8 (CH$_2$CH$_2$NH$_3^+$), 23.7 (CH$_2$CH$_2$S(CH$_2$)$_2$NH), 23.2 (CH$_2$CH$_2$O), 11.1 (SiCH$_2$(CH$_2$)$_2$S), 7.7 (SiCH$_2$(CH$_2$)$_2$O). **^{29}Si {^{1}H} NMR** (79 MHz, DMSO-d_6): δ 4.02 (Si^3), 3.91 (Si^2), not detected or overlapped (Si$^{0/1}$).

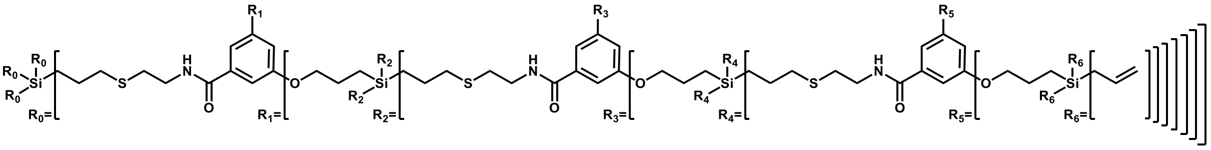

G$_3$-6-6-6-A. Dendrimer G$_3$-6-6-6-A was prepared from 800 mg (24.0 µmol) of G$_2$-6-6-N according to General procedure GP1. The product was purified by OSN using 3kDa membrane in DCM/MeOH mixture 4:3 and obtained as a brownish viscous substance (2.39 g, 96 %). **^{1}H NMR** (400 MHz, CDCl$_3$, ^{1}H-^{1}H COSY): δ 7.32 (br s, 172H, NH), 6.92 (br s, 344H, CH_{Ph}), 6.50 (br s, 172H, CH_{Ph}), 5.74 (ddt, J = 17.4, 10.2, 8.2 Hz, 824H, CHCH$_2$), 4.88–4.83 (m, 1728H, CHCH_2), 3.88–3.80 (m, 688H, OCH_2), 3.58–3.55 (m, 344H, CH_2NH), 2.70 (t, J = 6.7 Hz, 344H, SCH_2CH$_2$NH), 2.53–2.50 (m, 344H, SCH_2(CH$_2$)$_2$Si), 1.75–1.67 (m, 688H, OCH$_2$CH_2CH$_2$Si), 1.57 (dt, J = 8.2, 1.1 Hz, 1728H, SiCH_2CH), 1.58–1.56 (m, 344H, SiCH$_2$CH_2CH$_2$S), 0.67–0.59 (m, 1030H, SiCH_2). **^{13}C {^{1}H} NMR** (101 MHz, CDCl$_3$, ^{1}H-^{13}C HSQC, ^{1}H-^{13}C HMBC): δ 167.4 (HNCO), 160.3 (CH$_{Ph}$$C_{q(Ph)}OCH_2$), 136.4 (HNCO$C_{q(Ph)}CH_{Ph}C_{q(Ph)}$), 134.2 ($CHCH_2$), 114.0 (CH$CH_2$), 105.6 (HNCOC$_{q(Ph)}$$CH_{Ph}C_{q(Ph)}$), 104.6 (CH$_2OC_{q(Ph)}$$CH_{Ph}C_{q(Ph)}OCH_2$), 70.8 (O$CH_2$(CH$_2$)$_2$Si), 39.8 ($CH_2$NH), 35.7 (Si(CH$_2$)$_2$$CH_2$S), 31.7 (S$CH_2CH_2$NH), 24.4 (SiCH$_2$$CH_2CH_2$S), 23.8 (OCH$_2$$CH_2CH_2Si^{1/2}$), 23.4 (OCH$_2$$CH_2CH_2$Si^3), 19.6 (Si$CH_2$CH), 11.8 (Si$CH_2$(CH$_2$)$_2$S), 8.4 (O(CH$_2$)$_2$$CH_2Si^{1/2}$), 7.6 (O(CH$_2$)$_2$$CH_2$Si^3). **^{29}Si {^{1}H} NMR** (79 MHz, CDCl$_3$): δ 4.03 (Si^2), –0.14 (Si^3), not detected or overlapped (Si$^{0/1}$).

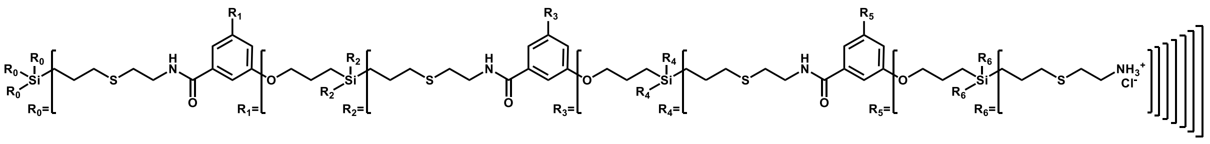

G$_3$-6-6-6-N. Dendrimer G$_3$-6-6-6-N was prepared from 132 mg (1.28 µmol) of dendrimer G$_3$-6-6-6-A according to General procedure GP2. The product was purified by OSN using 3kDa membrane in MeOH followed by lyophilization and obtained as off-white powder (240 mg, 93 %). **^{1}H NMR** (400 MHz, **100 °C**, DMSO-d_6, ^{1}H-^{1}H COSY): δ 8.57 (br s, 172H, NH), 8.35 (br s, 2592H, NH_3^+), 7.06 (br s, 344H, CH_{Ph}), 6.58 (br s, 172H, CH_{Ph}), 3.98 (br s, 688H, OCH_2), 3.46 (344H, CH_2NH), 3.04–3.01 (m, 1728H, CH_2NH$_3^+$), 2.88 (t, J = 7.7 Hz, 1728H, CH_2CH$_2$NH$_3^+$), 2.73 (br s, 344H, SCH_2CH$_2$NH), 2.60 (t, J = 7.0 Hz, 2072H, Si(CH$_2$)$_2$CH_2S), 1.72 (br s, 688H, OCH$_2$CH_2), 1.64–1.56 (m, 2072H, SiCH$_2$CH_2), 0.65–0.62 (m, 2760H, SiCH_2). **^{1}H NMR** (400 MHz, DMSO-d_6, ^{1}H-^{1}H COSY): δ 8.38 (br s, 172H, NH, 2592H, NH_3^+), 7.03 (br s, 344H, CH_{Ph}), 6.56 (br s, 172H, CH_{Ph}), 3.92 (br s, 688H, OCH_2), overlapped with water (CH_2NH), 2.96 (br s, 1728H, CH_2NH$_3^+$), 2.79 (br s, 1728H, CH_2CH$_2$NH$_3^+$), 2.55 (br s, 344H, SCH_2CH$_2$NH, 2072H, Si(CH$_2$)$_2$CH_2S), 1.64 (br s, 688H, OCH$_2$CH_2), 1.51 (m, 2072H, SiCH$_2$CH_2), 0.62 (br s, 2760H, SiCH_2). **^{13}C {^{1}H} NMR** (101 MHz, DMSO-d_6, ^{1}H-^{13}C HSQC, ^{1}H-^{13}C HMBC): δ 165.6 (NHCO), 159.7 ($C_{q(Ph)}$OCH$_2$), not detected (HNCO$C_{q(Ph)}$), not detected (HNCOC$_{q(Ph)}$$CH_{Ph}$), not detected ($CH_{Ph}$), from HSQC 70.4 (O$CH_2$), not detected ($CH_2$NH), 38.8 ($CH_2NH_3^+$), 34.6 (Si(CH$_2$)$_2$$CH_2$S), not detected (S$CH_2CH_2$NH), 27.8 (S$CH_2CH_2NH_3^+$), 23.7 (SiCH$_2$$CH_2$), 23.3 (OCH$_2$$CH_2$), 11.2 (Si$CH_2$(CH$_2$)$_2$S), 7.8 (O(CH$_2$)$_2$$CH_2$Si). **^{1}H NMR** (400 MHz, CD$_3$OD, ^{1}H-^{1}H COSY): δ 7.08 (br s, 344H, CH_{Ph}), 6.64 (br s, 172H, CH_{Ph}), 3.98 (br s, 688H, OCH_2), 3.58 (344H, CH_2NH), 3.22–3.17 (m, 1728H, CH_2NH$_3^+$), 2.94–2.90 (m, 1728H, CH_2CH$_2$NH$_3^+$), 2.84–2.80 (m, 344H, SCH_2CH$_2$NH), 2.65–2.61 (m, 2072H, Si(CH$_2$)$_2$CH_2S), 1.76 (br s, 688H, OCH$_2$CH_2), 1.64 (m, 2072H, SiCH$_2$CH_2), 0.73 (br s, 2760H, SiCH_2). **^{13}C {^{1}H} NMR** (101 MHz, CD$_3$OD, ^{1}H-^{13}C HSQC, ^{1}H-^{13}C HMBC): δ 169.3 (NHCO), 161.7 ($C_{q(Ph)}$OCH$_2$), 137.4 (HNCO$C_{q(Ph)}$), 107.1 (HNCOC$_{q(Ph)}$$CH_{Ph}$), from HSQC 105.9 ($CH_{Ph}$), 72.3 (O$CH_2$), 40.5 ($CH_2NH_3^+$), 40.3 ($CH_2$NH), 36.5 (Si(CH$_2$)$_2$$CH_2$S), 32.3 (S$CH_2CH_2$NH), 29.8 (S$CH_2CH_2NH_3^+$), 25.5 (SiCH$_2$$CH_2$),

25.0 ($OCH_2\mathit{C}H_2$), 12.7 ($Si\mathit{C}H_2(CH_2)_2S$), 9.4 ($O(CH_2)_2\mathit{C}H_2Si$). **^{29}Si {^{1}H} NMR** (79 MHz, CD_3OD): δ 3.94 (Si^3), not detected or overlapped ($Si^{0/1/2}$).

2. MALDI-TOF and ESI-QTOF MS analysis

The smallest polyallyl dendrimer G_1-3-A and both 1st generation polycationic dendrimers G_1-3-N and G_1-6-N were characterized by ESI-QTOF HRMS in addition to MALDI-TOF MS. Molar masses of the ammonium dendrimers G_1-3-N and G_1-6-N are at the upper bound of the measurable range for conventional ESI-QTOF HRMS (3114.2 and 5310.9 g/mol, resp.). As such, direct observation of their molecular peaks was unsuccessful. However, as reported for other dendritic polyelectrolytes,[1] the easily ionizable character of the structures enables their observation as a Gauss-like distribution of polycations. During ionization, the molecules lost all chlorides either in the form of HCl or as Cl^- ions, leaving part of the amino groups protonated. Thus, we could observe ions of a general structure $[M\text{-}x\text{HCl}+y\text{H}]^{y+}$, where x = number of PG and y = charge of the particular ion. The ionization optimum was around 25 % for both G_1 ammonium dendrimers, i.e., the most abundant ions were from M^{2+} to M^{4+} for G_1-3-N, and from M^{4+} to M^{7+} for G_1-6-N.

Regarding the dendritic purity of the prepared compounds, several defective structures were observed in mass spectra. Structures resulting from incomplete TEC reaction, missing one or two ammonium linkers, accompanied all ions of non-defective dendrimers in nearly constant ratios, which were in agreement with a statistical character of such defects. Assuming similar ionizabilities of the defective structures (possessing nearly the same number of ionizable ammonium PGs and similar molecular weight), it is possible to calculate the conversion ξ of the TEC reaction and thus the defectiveness of the surface of ammonium dendrimers (1-ξ) from observed intensities of non-defective and defective ions according to Eq. 2.

$$\xi = \frac{n}{n+\frac{I_{D1}}{I_{D0}}} \qquad (2)$$

where n represents the number of reacting groups per molecule and I_{Dx} stands for the peak intensity of the structure with x defects. It has to be noted that the actual quantity of unreacted groups can be lower due to a plausible fragmentation pathway, reverse elimination on a thioether bond, which would generate a thiol and the observed allyl-containing fragment.

Only the smallest G_2 ammonium dendrimer with 36 PGs gave useful ESI-QTOF HRMS data; larger ammonium dendrimers ionized in a wide distribution of differently charged ions, which, together with broadening of their isotopic multiplets at higher masses and increasing probability of defects and fragmentation, had a detrimental effect on the S/N ratio. The rest of the G_2 structures were thus analyzed only by MALDI-TOF MS, mostly in a linear mode, while most G_3 dendrimers were too large even for this ionization method. Again, polyallyl dendrimers showed complete conversion of the amidic coupling, while for polyammonium dendrimers we observed the presence of structures missing 1-3 ammonium linkers, which corresponded to ca. 98% conversion of TEC leaving 2% of unreacted allyl groups. In any case, the above remarks on the possible fragmentation of thioesters apply also to higher generations.

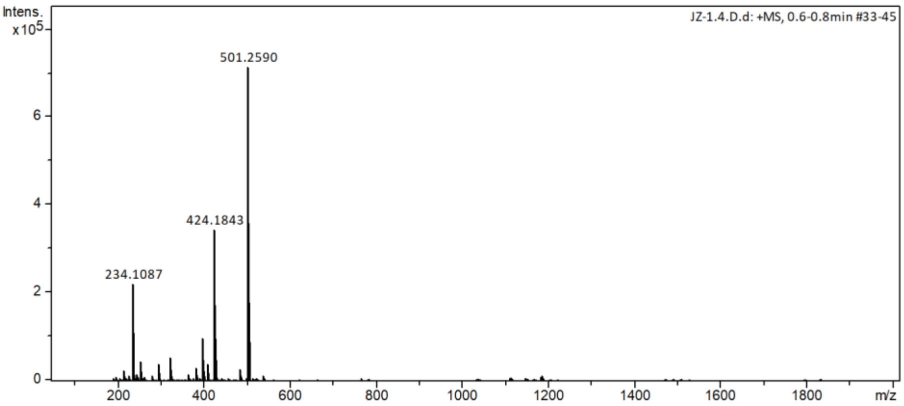

Figure S1: ESI$^+$ QTOF mass spectrum of G_0-N

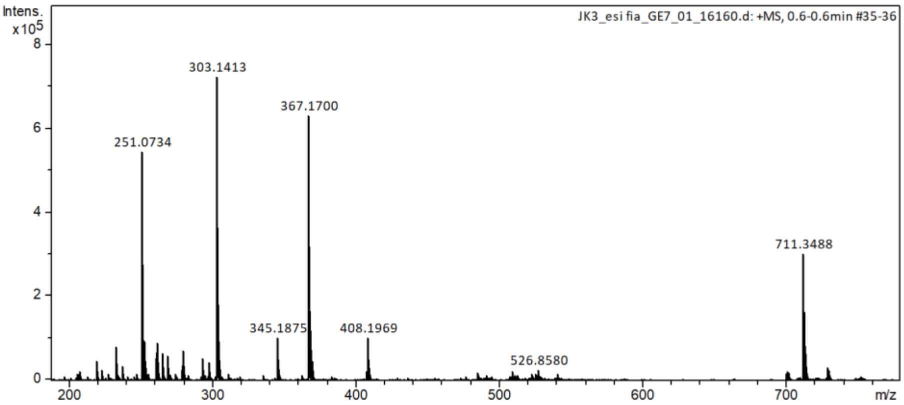

Figure S2: ESI$^+$ QTOF mass spectrum of MeO-AB_3

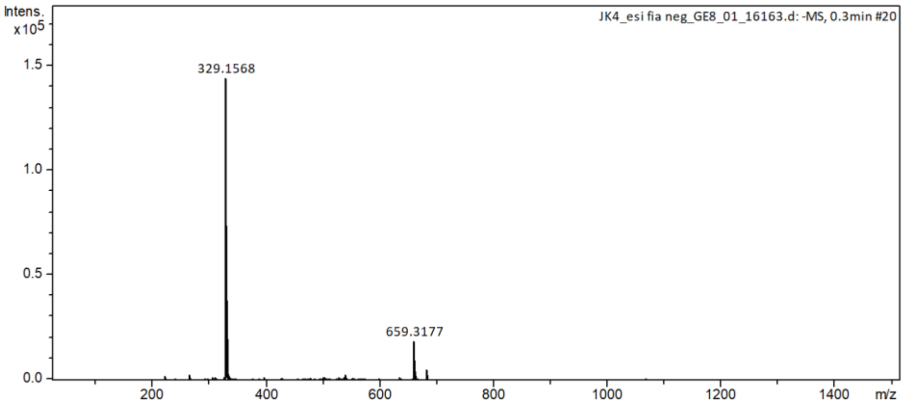

Figure S3: ESI$^-$ QTOF mass spectrum of AB_3

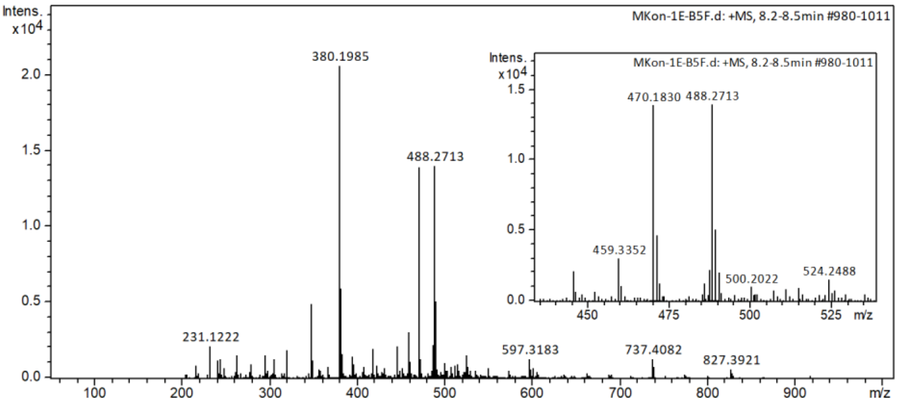

Figure S4: ESI$^+$ QTOF mass spectrum of BtO-AB_3

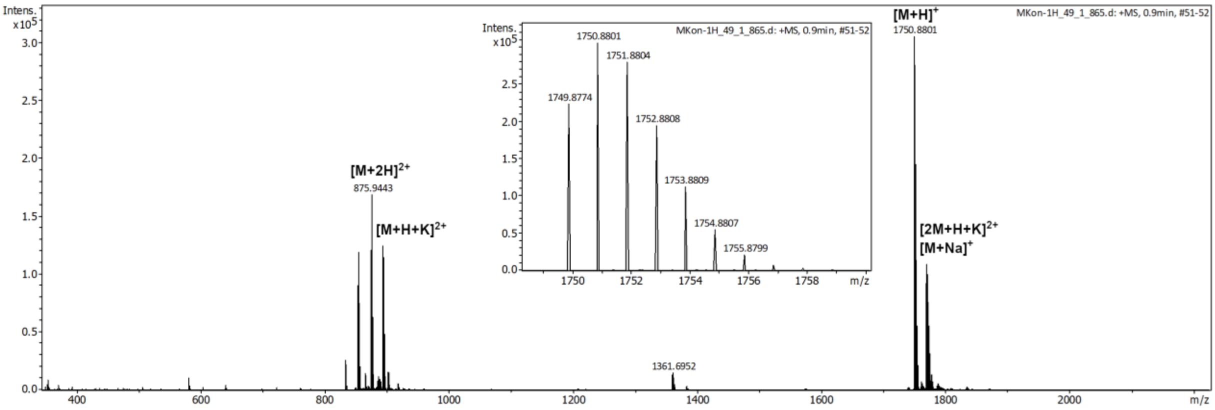

Figure S5. ESI$^+$ QTOF mass spectrum of G_1-3-A

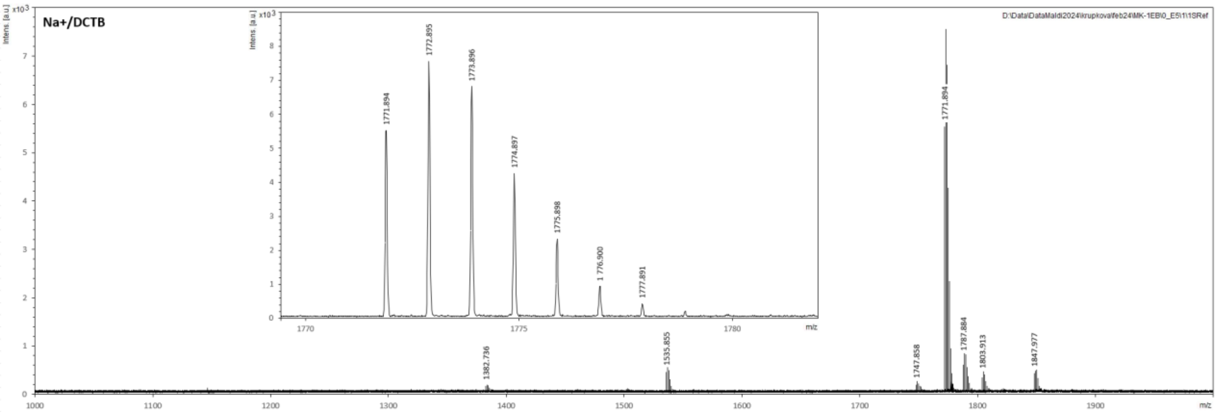

Figure S6. MALDI-TOF mass spectrum (DCTB/Na$^+$; reflectron mode) of G_1-3-A

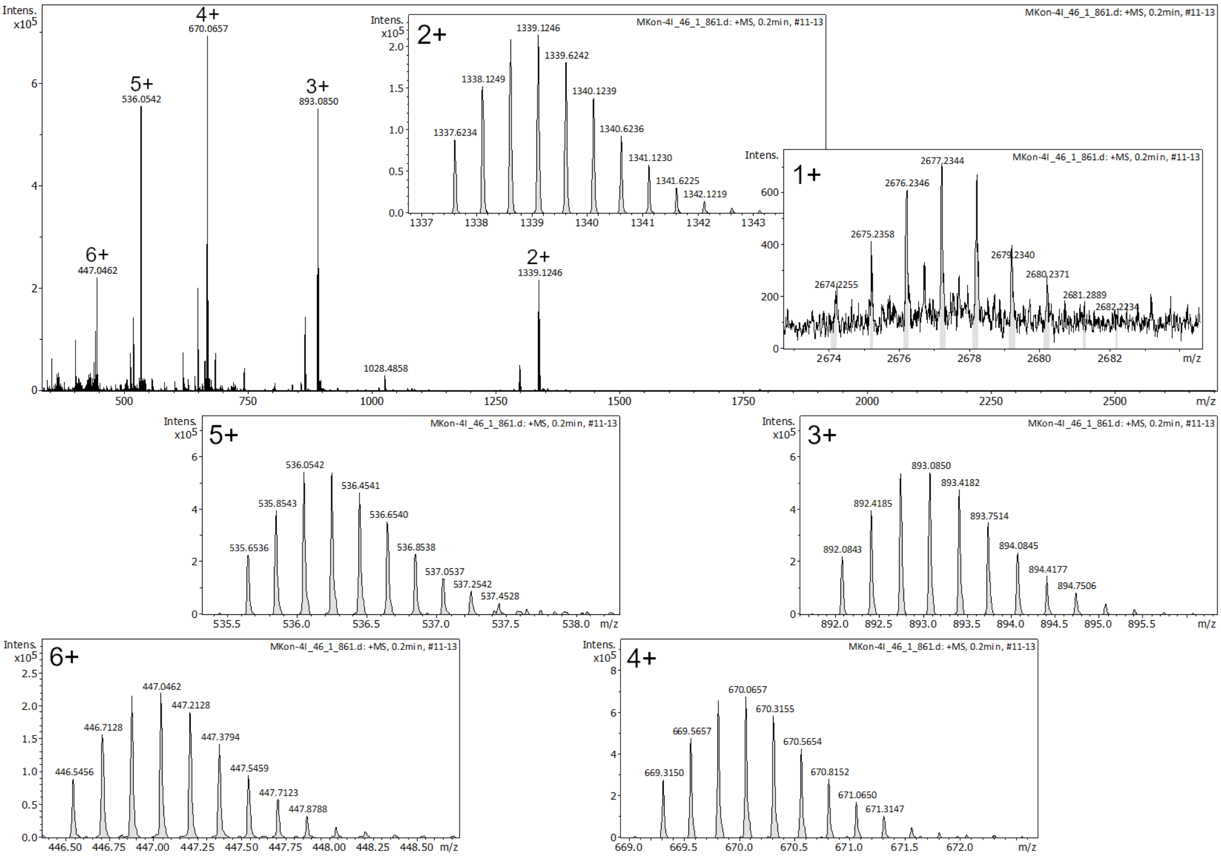

Figure S7. ESI$^+$ QTOF mass spectrum of G_1-3-N

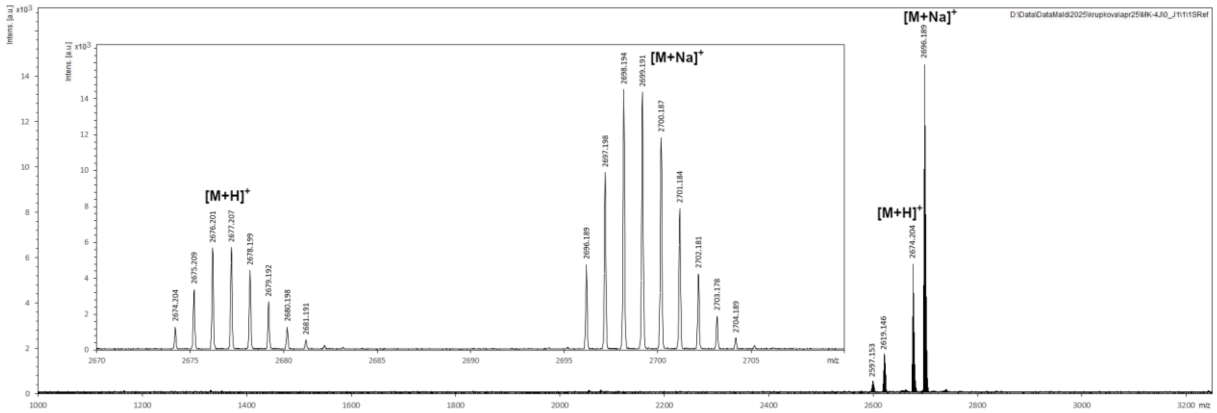

Figure S8. MALDI-TOF mass spectrum (DHB/Na^+; reflectron mode) of G_1-3-N

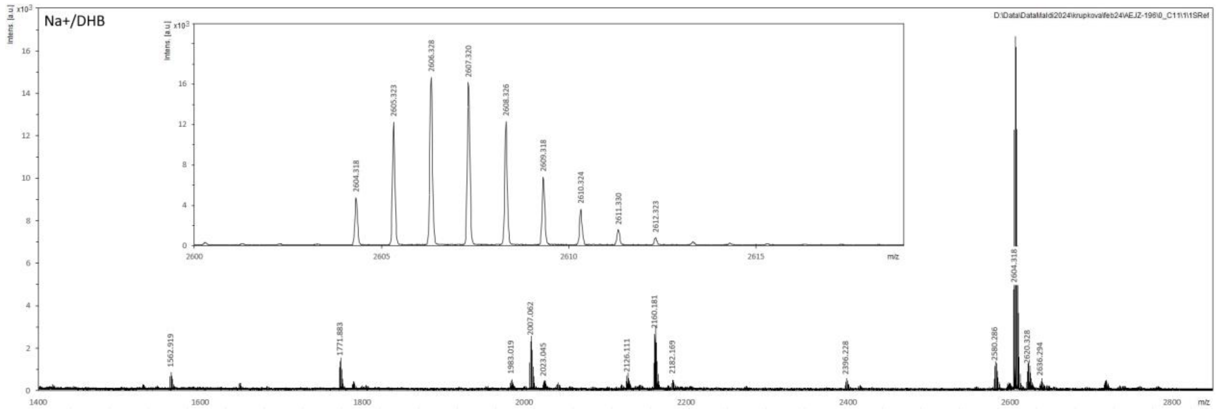

Figure S9. MALDI-TOF mass spectrum (DHB/Na^+; reflectron mode) of G_1-6-A

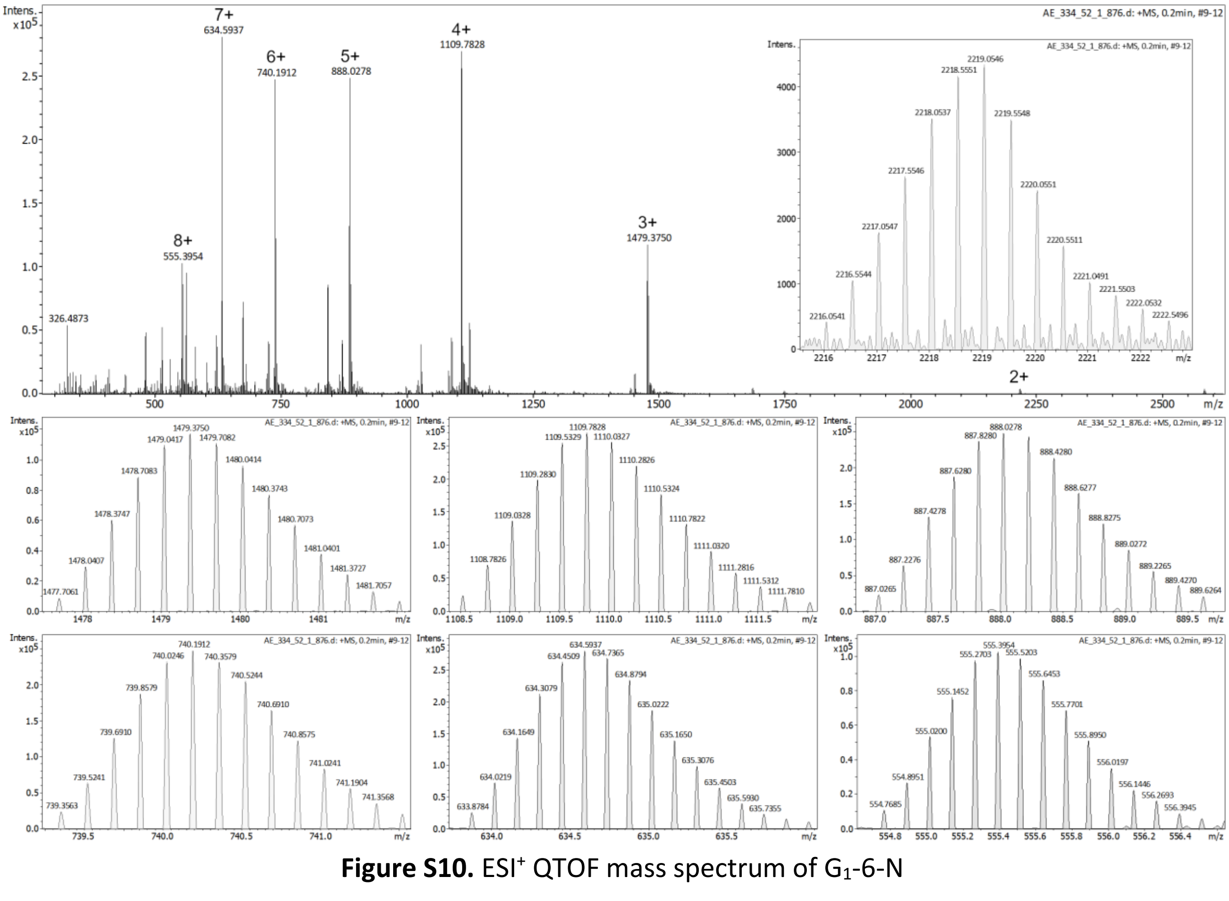

Figure S10. ESI$^+$ QTOF mass spectrum of G_1-6-N

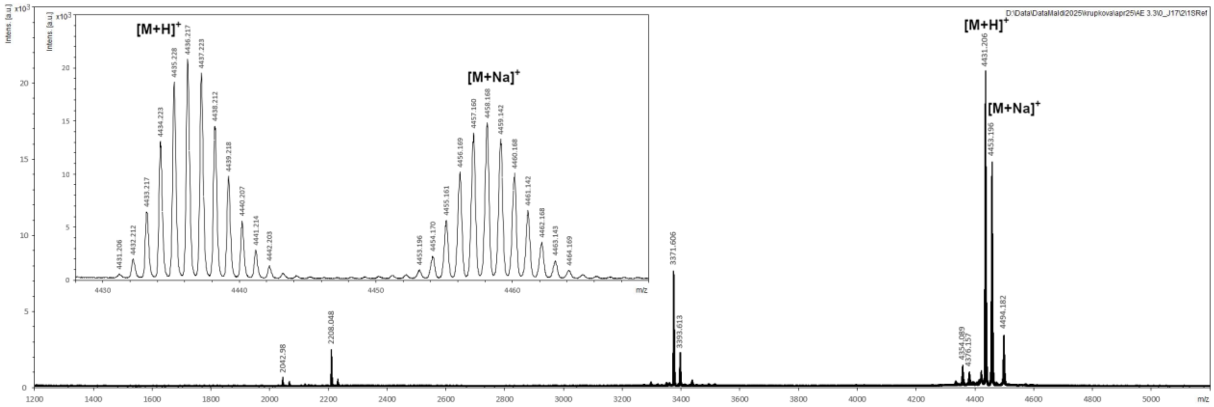

Figure S11. MALDI-TOF mass spectrum (DHB/Na^+; reflectron mode) of G_1-6-N

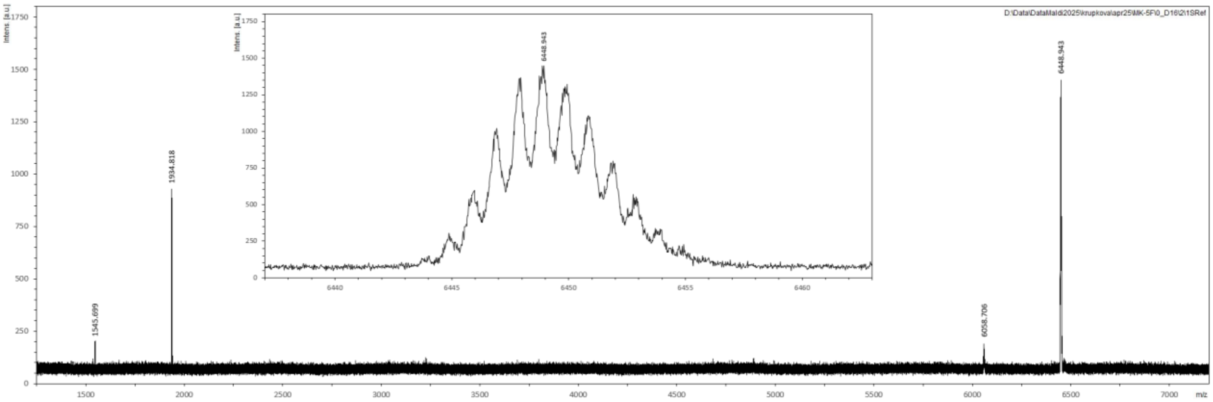

Figure S12. MALDI-TOF mass spectrum (DCTB/Na^+; reflectron mode) of G_2-3-3-A

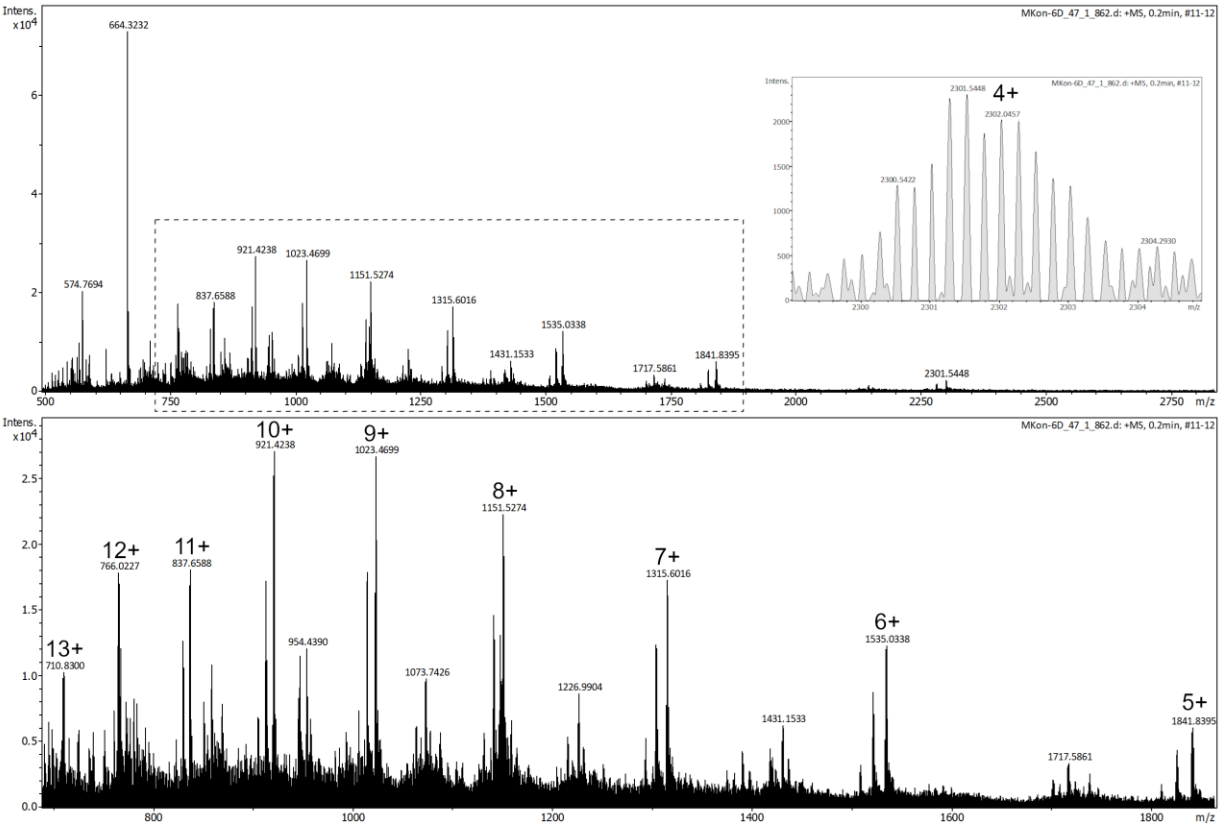

Figure S13. ESI^+ QTOF MS spectrum of G_2-3-3-N

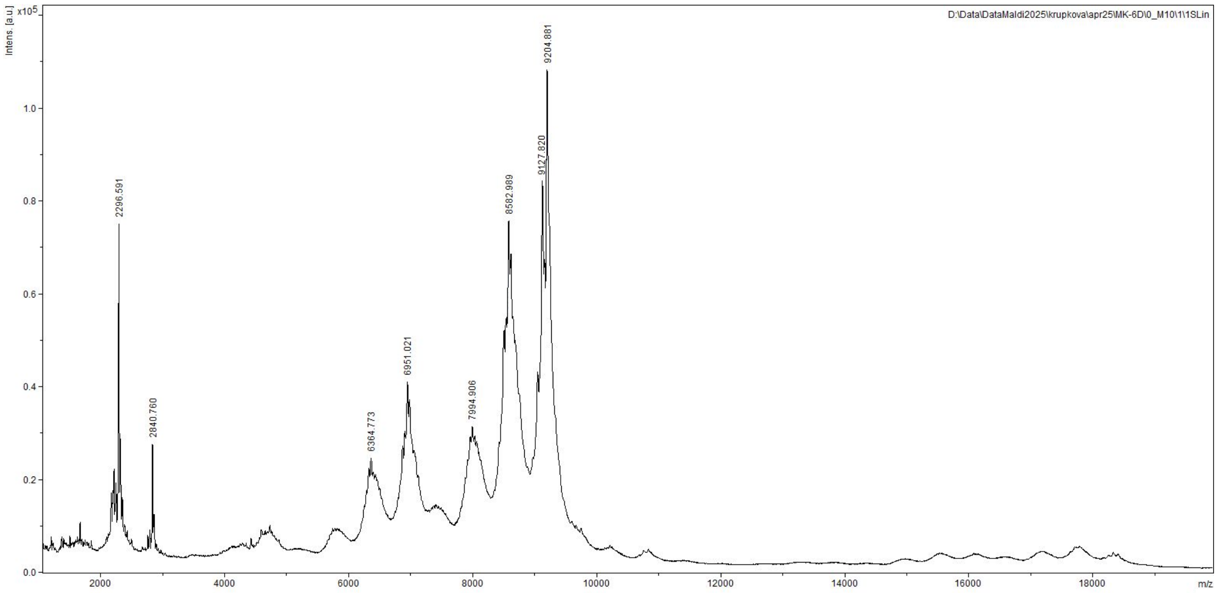

Figure S14. MALDI-TOF MS spectrum (DHB/Na^+; linear mode) of G_2-3-3-N

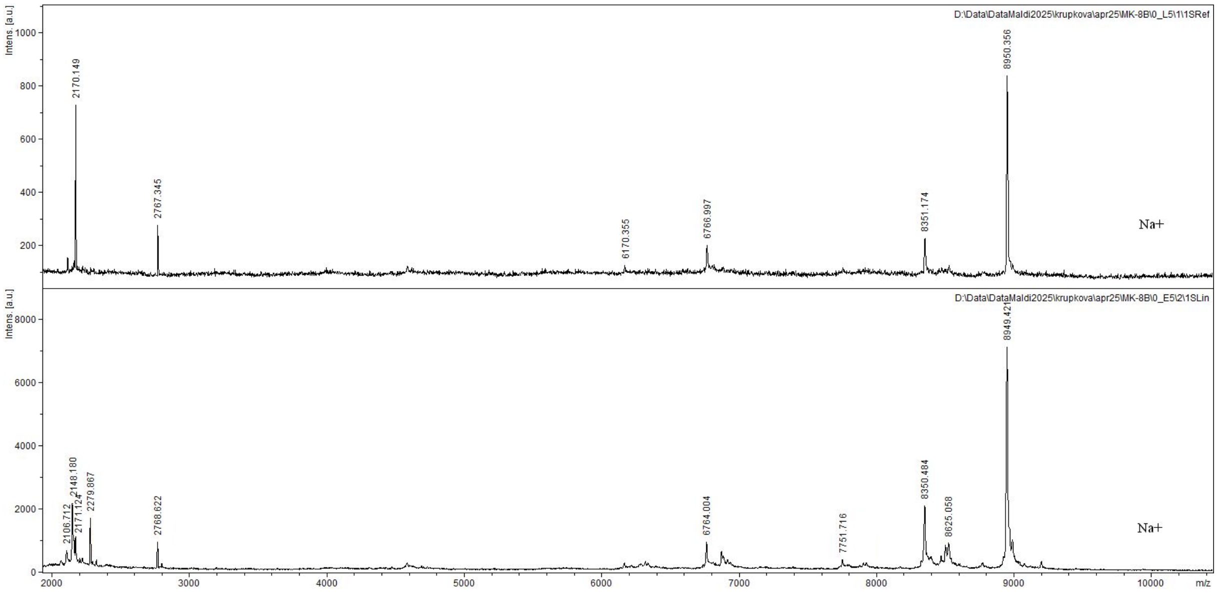

Figure S15. MALDI-TOF mass spectra (DCTB/Na^+; reflectron↑/linear↓ mode) of G_2-3-6-A

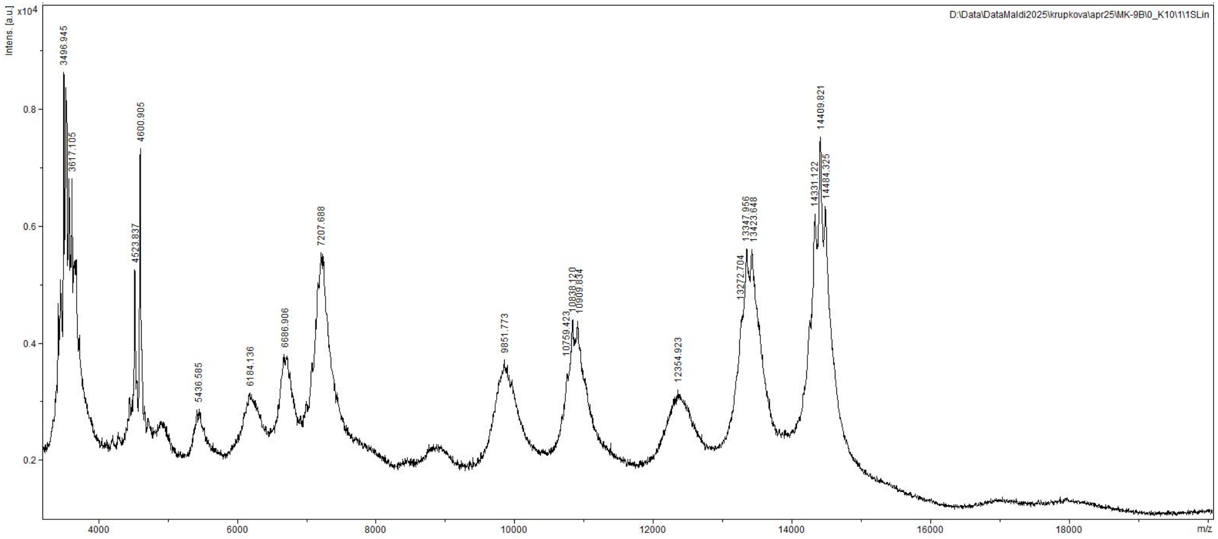

Figure S16. MALDI-TOF mass spectrum (DHB/Na^+; linear mode) of G_2-3-6-N

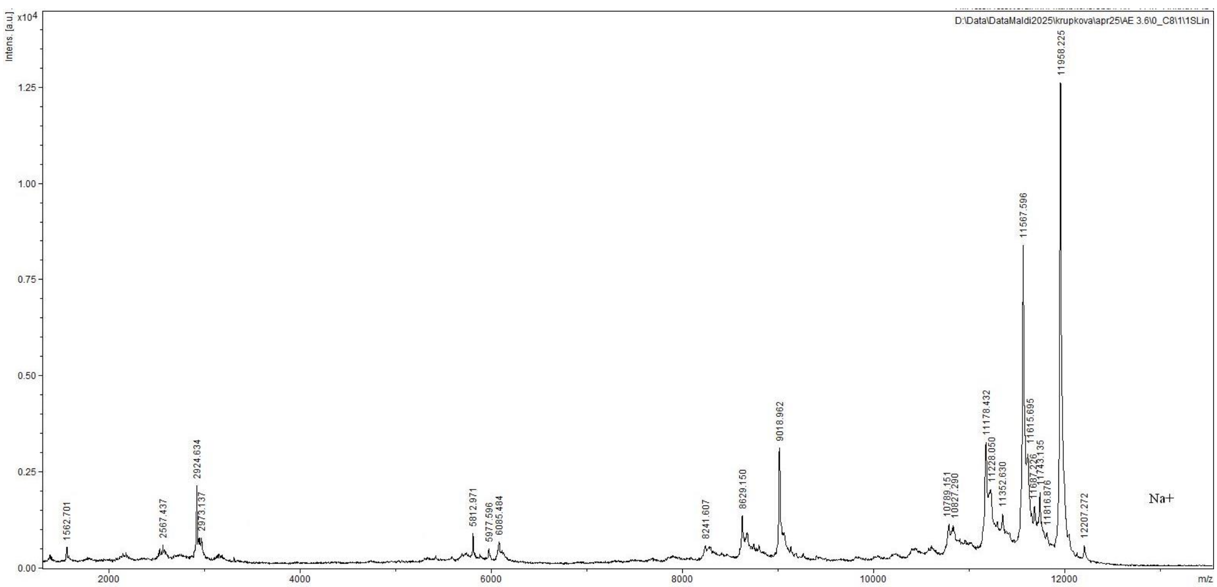

Figure S17. MALDI-TOF mass spectrum (DCTB/Na^+; linear mode) of G_2-6-3-A

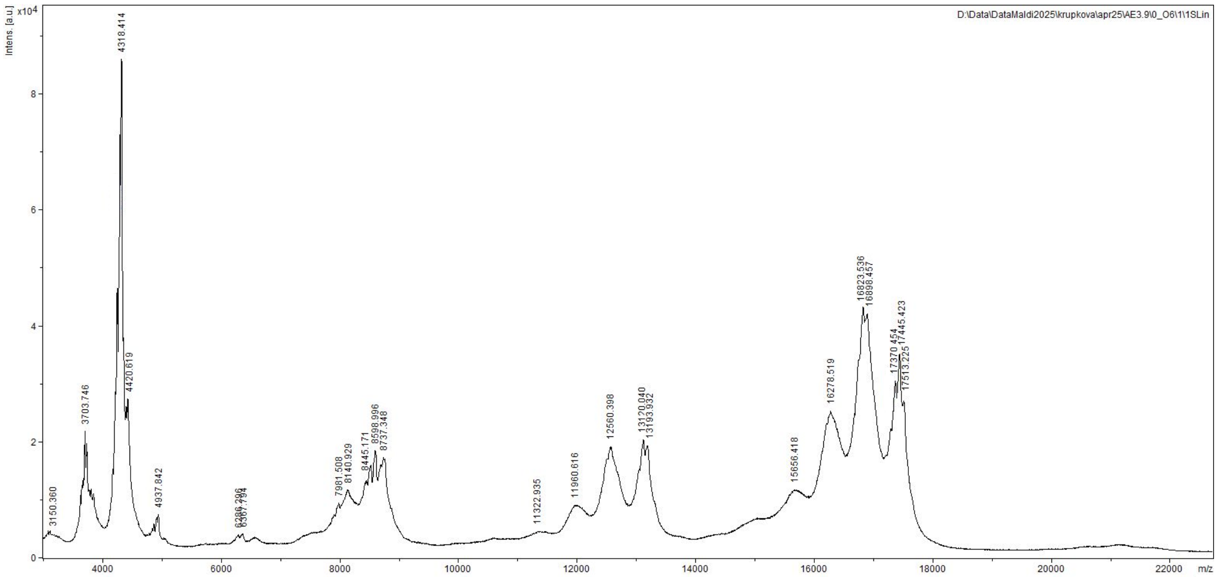

Figure S18. MALDI-TOF mass spectrum (DHB/Na^+; linear mode) of G_2-6-3-N

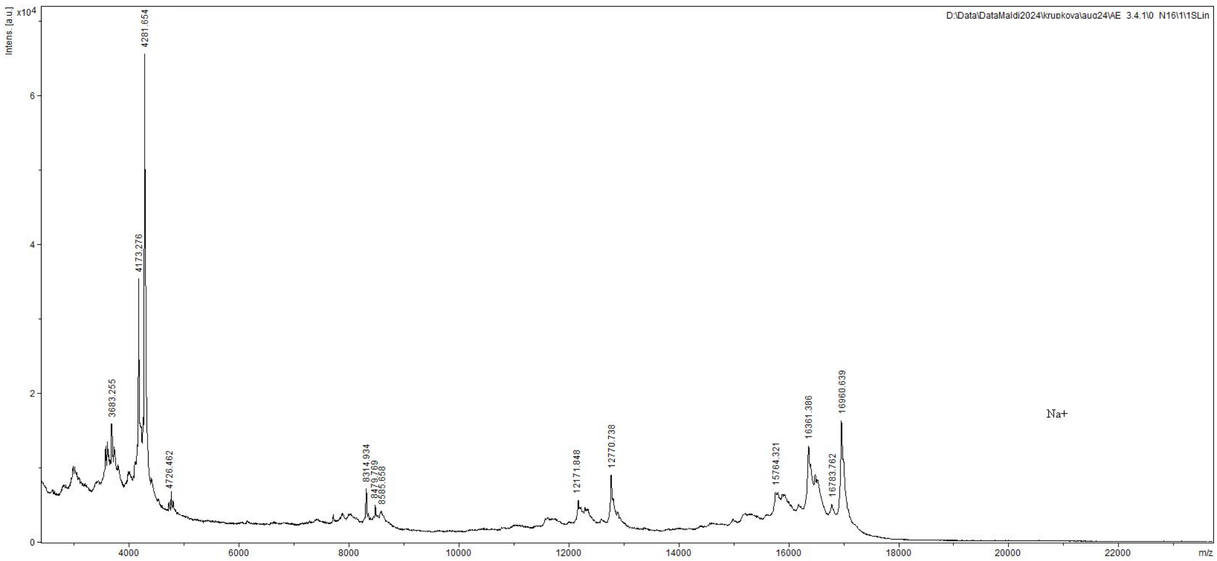

Figure S19. MALDI-TOF mass spectrum (DCTB/Na^+; linear mode) of G_2-6-6-A

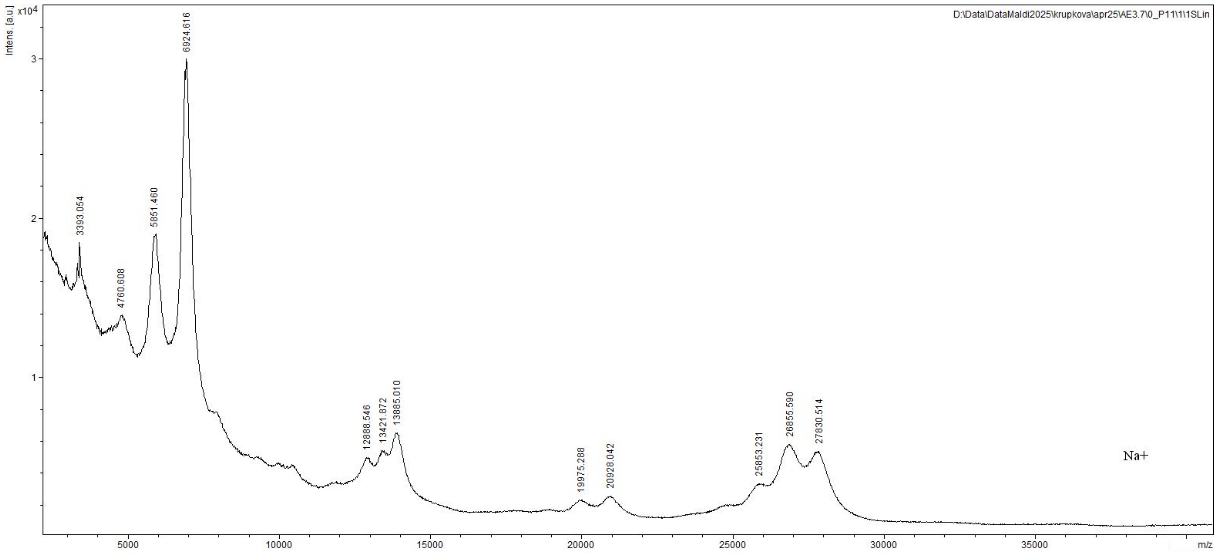

Figure S20. MALDI-TOF mass spectrum (DHB/Na^+; linear mode) of G_2-6-6-N

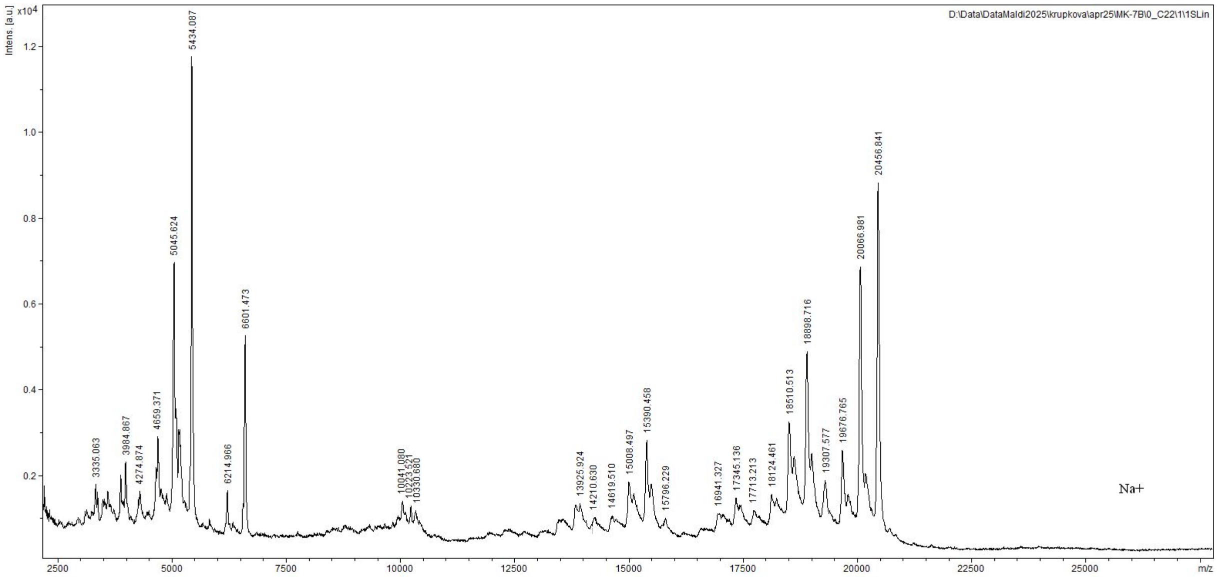

Figure S21. MALDI-TOF mass spectrum (DCTB/Na^+; linear mode) of G_3-3-3-3-A

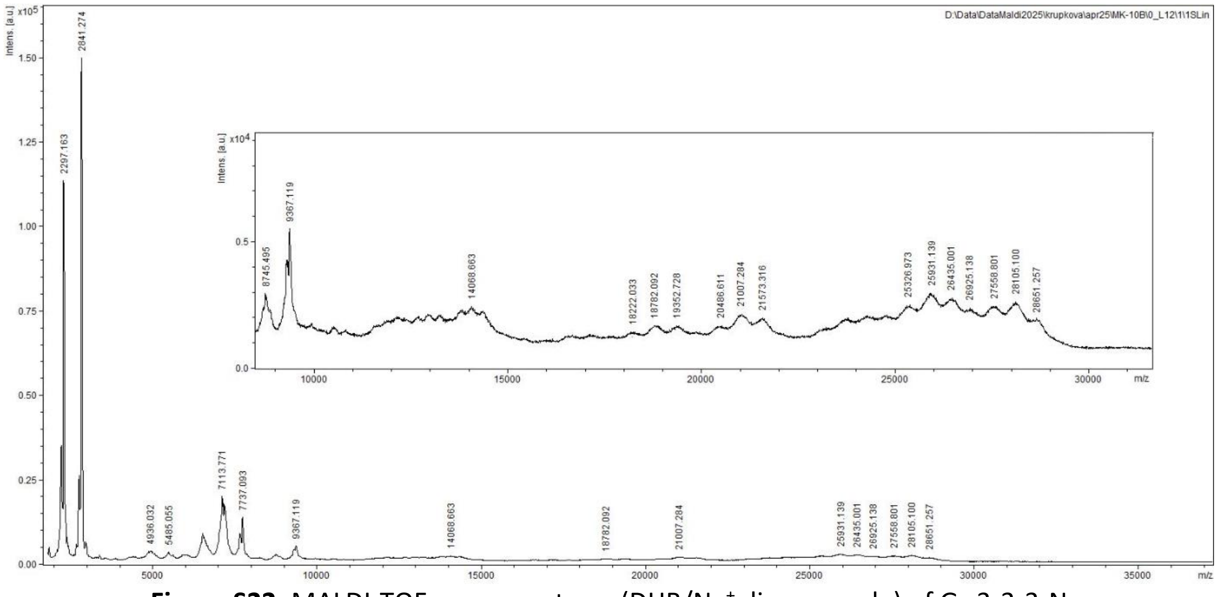

Figure S22. MALDI-TOF mass spectrum (DHB/Na^+; linear mode) of G_3-3-3-3-N

3. Recycling of modules AB_3 and AB_6

Due to the different reactivity of both activated esters, the modules are isolated from the filtrate in different forms, which entails their variant workup. All forms of the smaller AB_3 module present in the filtrates can be easily converted to the free acid, following the procedure used for its preparation from the methyl ester. In contrast, the AB_6 module is recovered as a mixture of free acid, methyl ester, and dimethylamide. Due to the higher hydrolytic stability of amides compared to esters, a longer reaction time and a change in reaction media were necessary for the conversion to AB_6 free acid, and the product was slightly contaminated with residual amide. To simplify the recycling of this module, the dimethylamide formation can presumably be suppressed by using dimethylformamide for peptide synthesis as a solvent for the coupling reaction. Nevertheless, after the aqueous workup, nanofiltration, additional washing and hydrolysis, we were able to recover 91% of the excess AB_6 module employed in the coupling reaction. Comparison of ^{1}H NMR spectra of both recycled modules with the originally prepared ones are shown below (Figures S23 and S24). Recycled AB_6 module was utilized to prepare G_1-6-A dendrimer; the product was isolated in 89% yield, confirming the applicability of the recycling.

Beneficially, the more stable activated ester BtO-AB_3 can be crystallized from the OSN filtrate and directly used for further synthesis. As proved by the reaction of BtO-AB_3 with the core molecule G_0-N, the coupling proceeds without the addition of TBTU or other activating agents under the common conditions used for amidic coupling. The target G_1-3-A dendrimer was obtained in comparable purity to that prepared from fresh AB_3.

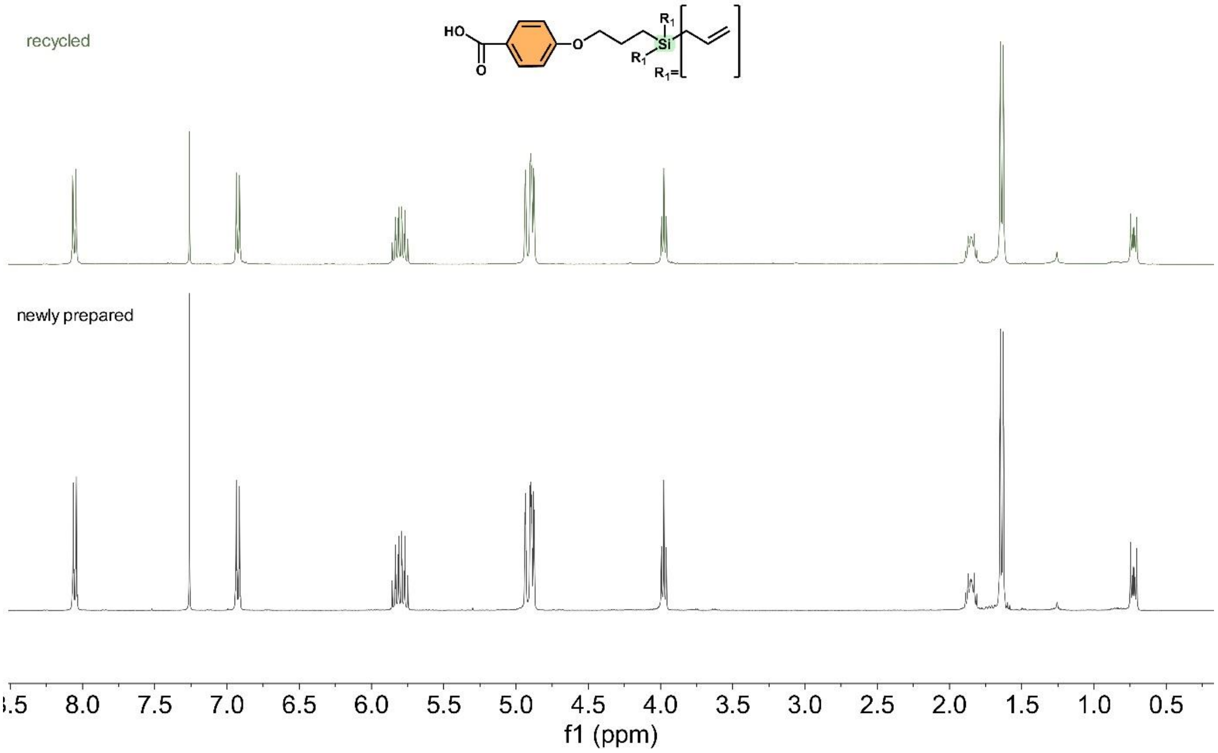

Figure S23: ^{1}H NMR (400 MHz, $CDCl_3$) Comparison of proton spectra of recycled and originally prepared (bottom) module AB_3

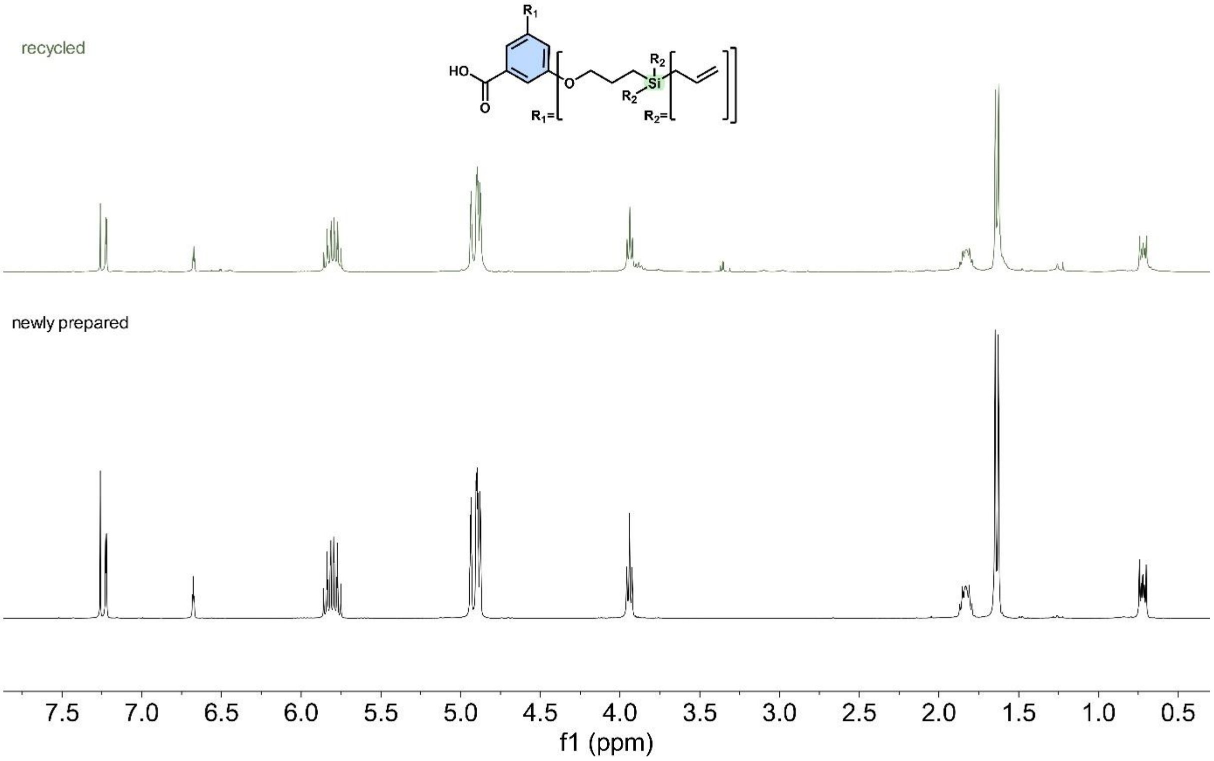

Figure S24: ^{1}H NMR (400 MHz, $CDCl_3$) Comparison of proton spectra of recycled and freshly prepared module AB_6

4. Dynamic light scattering (DLS)

Dynamic Light Scattering (DLS). The size of dendrimers was estimated by multi-angle dynamic light scattering (MADLS)[2] on Zetasizer Ultra Red (Malvern Panalytical, UK) equipped with a 632.8 nm laser applying detection angles 12.78°, 90.0° and 174.7° in DTS0012 polystyrene cuvettes (Malvern Panalytical, UK). Samples were sonicated for 30 min, filtered through 0.22 µm Nylon syringe filters (Avantor, USA), and measured at a concentration of 5.0 mg/mL in 0.25 % NaCl solution in MeOH. Dendrimers exhibited the lowest tendency to aggregate in this medium compared to pure water, 0.9 % NaCl aqueous solution, and pure MeOH. Each measurement was repeated at least 5 times and the obtained data were analyzed by ZS XPLORER v 3.3.1.5. The presented solvodynamic diameters are mean values from the volume-weighted size distribution.

Optimal concentration determination: 20.0 mg/mL solution of G_2-3-3-N was prepared and diluted 2-fold until 0.31 mg/mL (factor 64×). All prepared solutions were measured and the results compared. From 20.0 to 5.0 mg/mL the observed particle size increased by 16 %, whereas from 5.0 to 0.31 mg/mL the size increased only by less than 5 %. However, from 2.5 to 0.31 mg/mL (from 0.8 to 0.1 nmol/L) the instrument could not reach the standard 300 kcps (kilocounts per second) for side scattering (90.0°) even though setting the highest possible attenuator factor and the quality of correlograms became insufficient. Thus, 5.0 mg/mL was chosen as the optimal concentration for all measurements.

Zeta potential: Zeta potential was measured on the same instrument applying a detection angle 12.78° (forward scattering) in DTS1070 folded capillary Zeta cell (Malvern Panalytical, UK). Samples were sonicated for 30 min, filtered through 0.45 µm Nylon syringe filters (Avantor, USA) and measured at a concentration of 5.0 mg/mL in 0.9 % NaCl aqueous solution (physiological solution). Slow field reversal was avoided (by selecting „monomodal“ analysis model) to prevent electrolysis of the sample. Standard deviation was chosen as a characteristic of variability. Each measurement was repeated 10 times and the obtained data were analyzed by ZS XPLORER v 3.3.1.5.

Table S1. Size differences between dendrimers differing just in the composition of one layer

Generation	Dendrimer 1	Dendrimer 2	ΔR_h **(2–1)** [nm]	note
1	G_1-3-N	G_1-6-N	0.8	
2	G_2-3-3-N	G_2-6-3-N	*1.6*	*inner layer*
	G_2-3-3-N	G_2-3-6-N	1.3	
	G_2-3-6-N	G_2-6-6-N	*1.4*	*inner layer*
	G_2-6-3-N	G_2-6-6-N	1.1	
3	G_3-3-3-3-N	G_3-6-3-3-N	*2.4*	*inner layer*
	G_3-3-3-3-N	G_3-3-6-3-N	1.4	
	G_3-3-3-3-N	G_3-3-3-6-N	2.0	
	G_3-3-3-6-N	G_3-6-3-6-N	*2.1*	*inner layer*
	G_3-3-3-6-N	G_3-3-6-6-N	1.5	
	G_3-3-6-3-N	G_3-6-6-3-N	*2.9*	*inner layer*
	G_3-3-6-3-N	G_3-3-6-6-N	2.1	
	G_3-6-3-3-N	G_3-6-6-3-N	1.9	
	G_3-6-3-3-N	G_3-6-3-6-N	1.7	
	G_3-3-6-6-N	G_3-6-6-6-N	*2.6*	*inner layer*
	G_3-6-3-6-N	G_3-6-6-6-N	2.0	
	G_3-6-6-3-N	G_3-6-6-6-N	1.8	

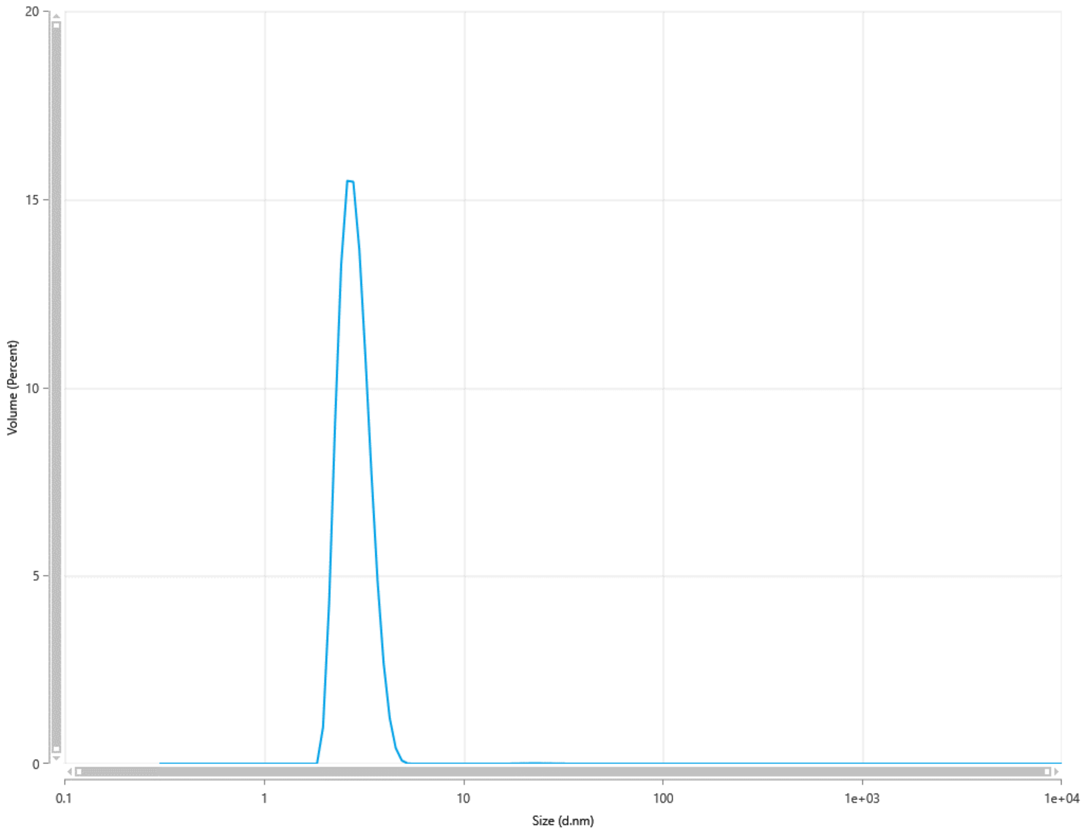

Figure S25. Volume-weighted size distribution of G_1-3-N.

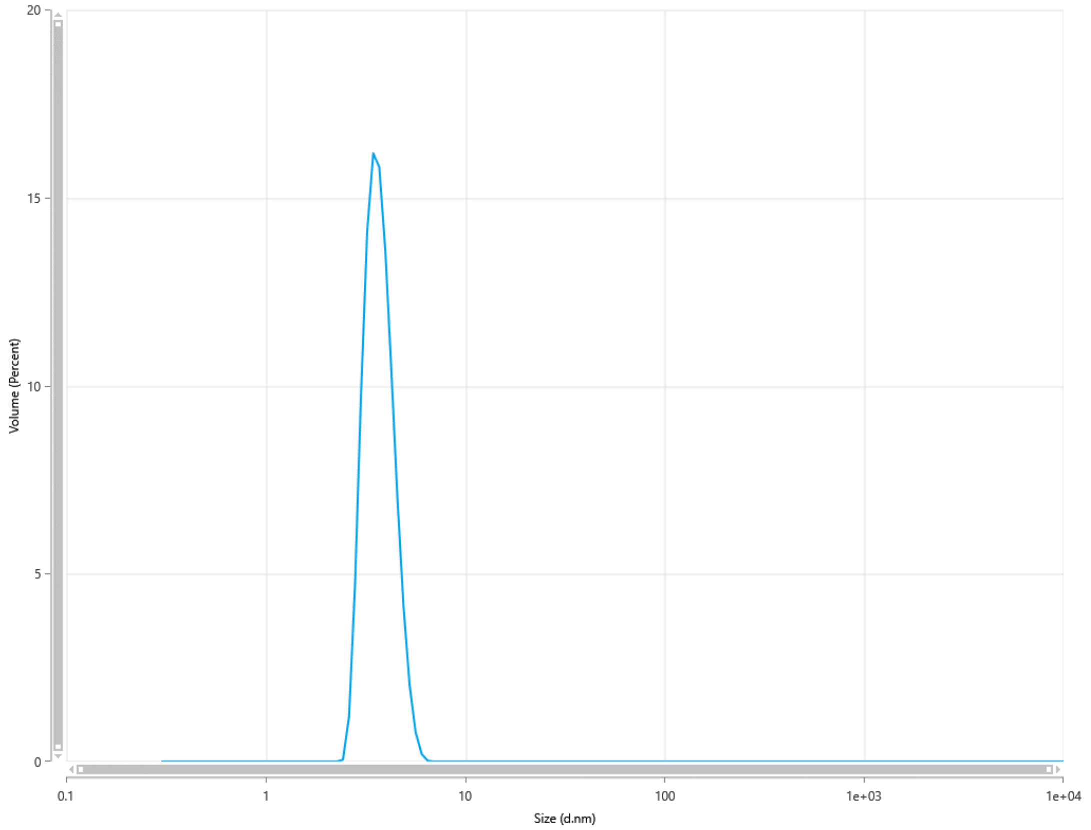

Figure S26. Volume-weighted size distribution of G_1-6-N.

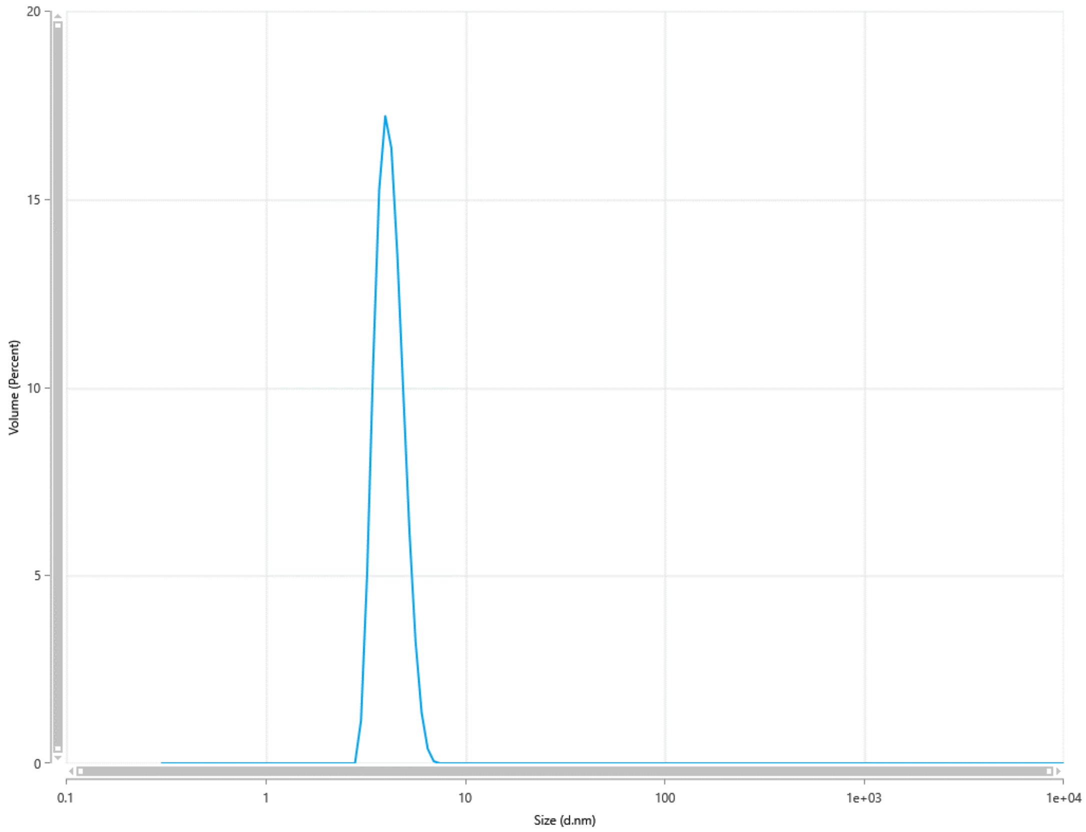

Figure S27. Volume-weighted size distribution of G_2-3-3-N.

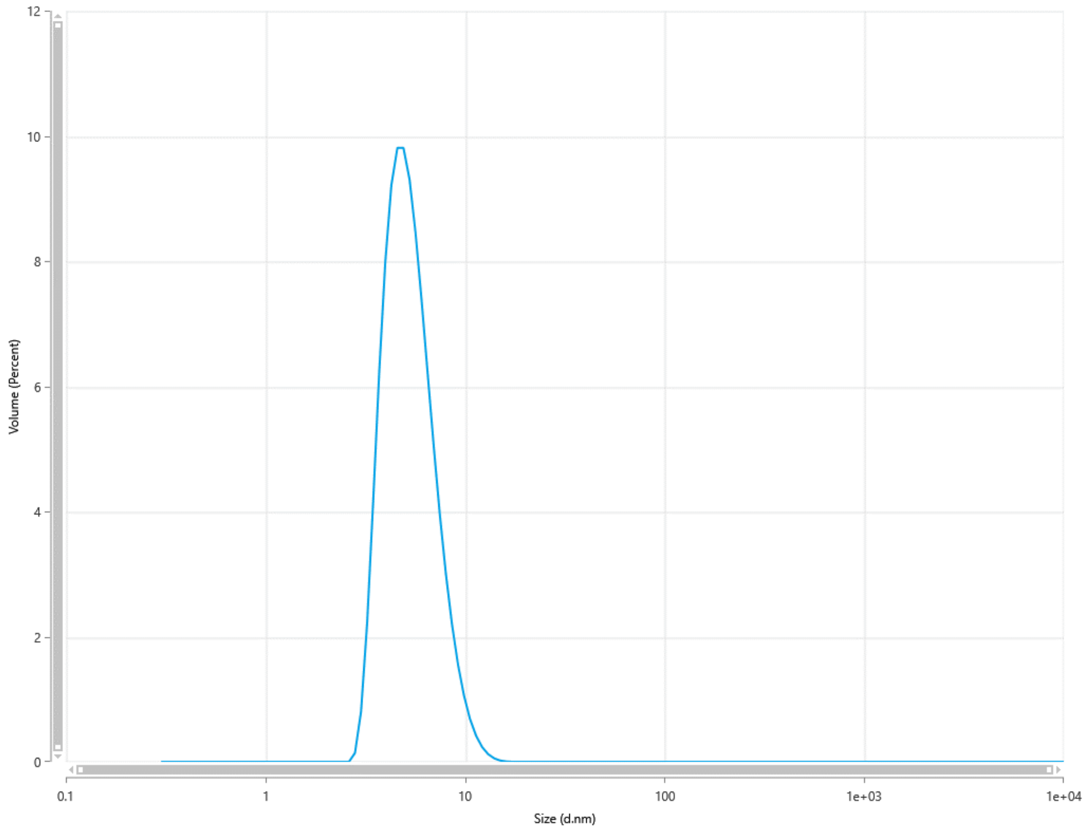

Figure S28. Volume-weighted size distribution of G_2-3-6-N.

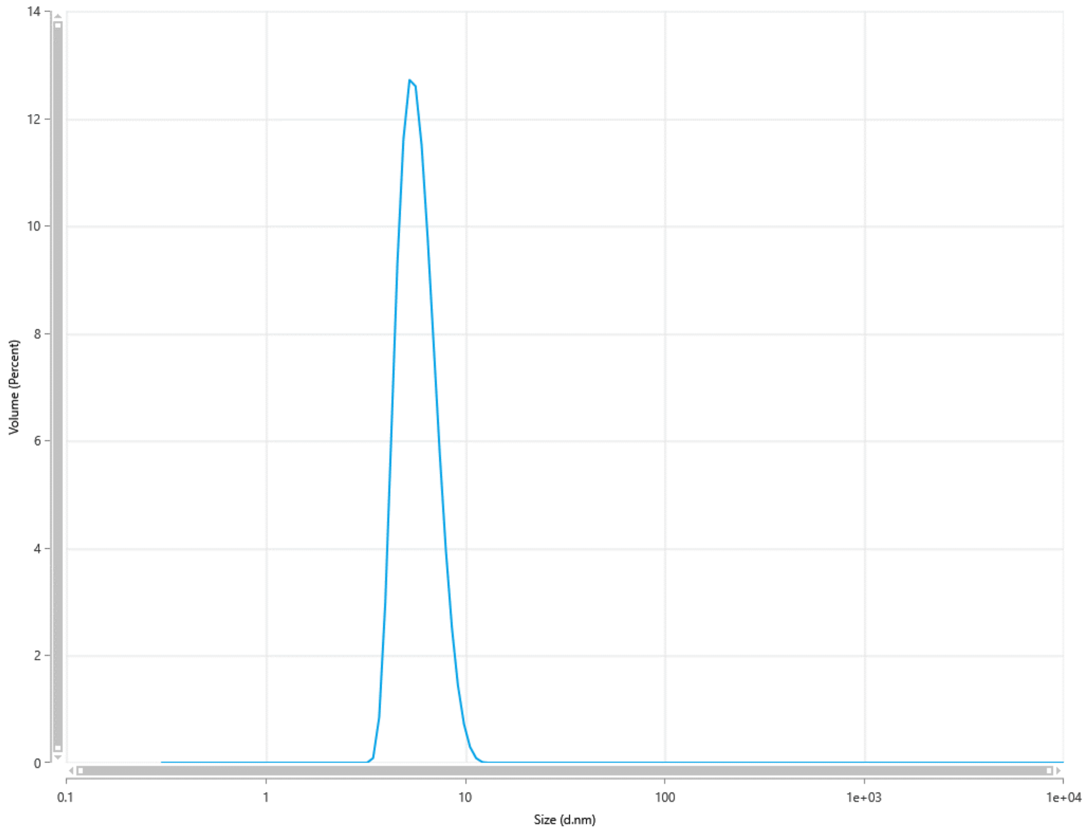

Figure S29. Volume-weighted size distribution of G_2-6-3-N.

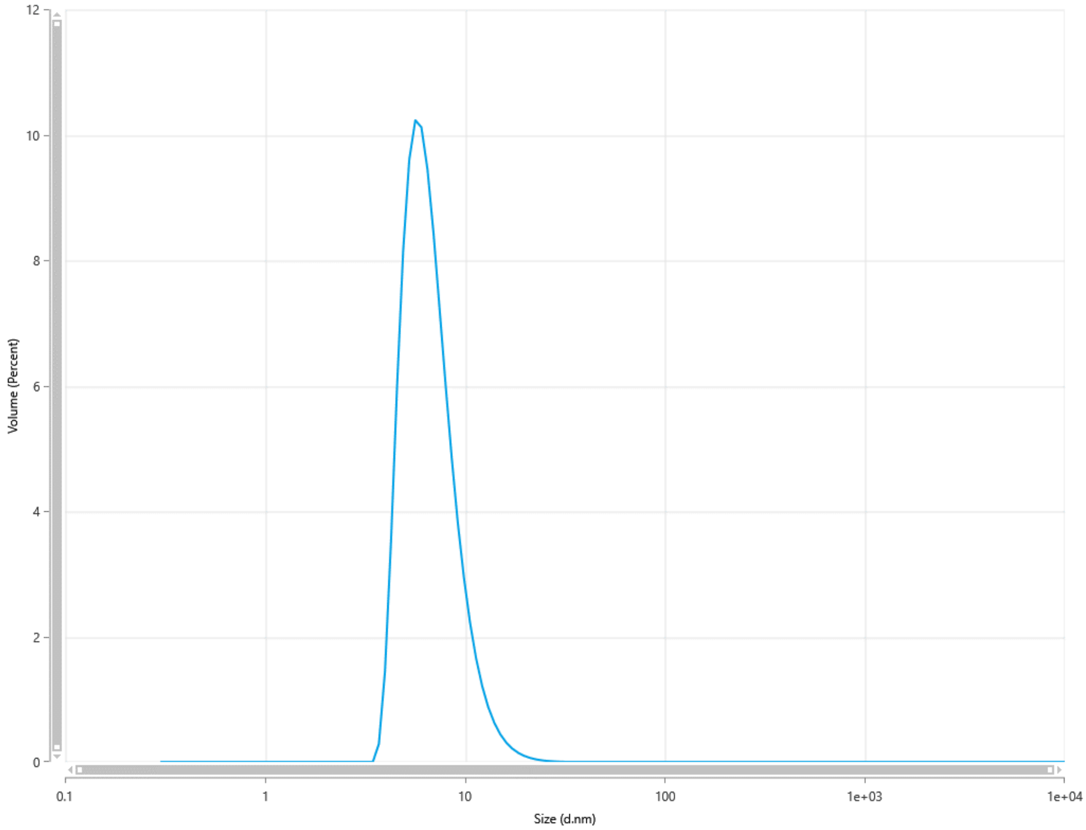

Figure S30. Volume-weighted size distribution of G_2-6-6-N.

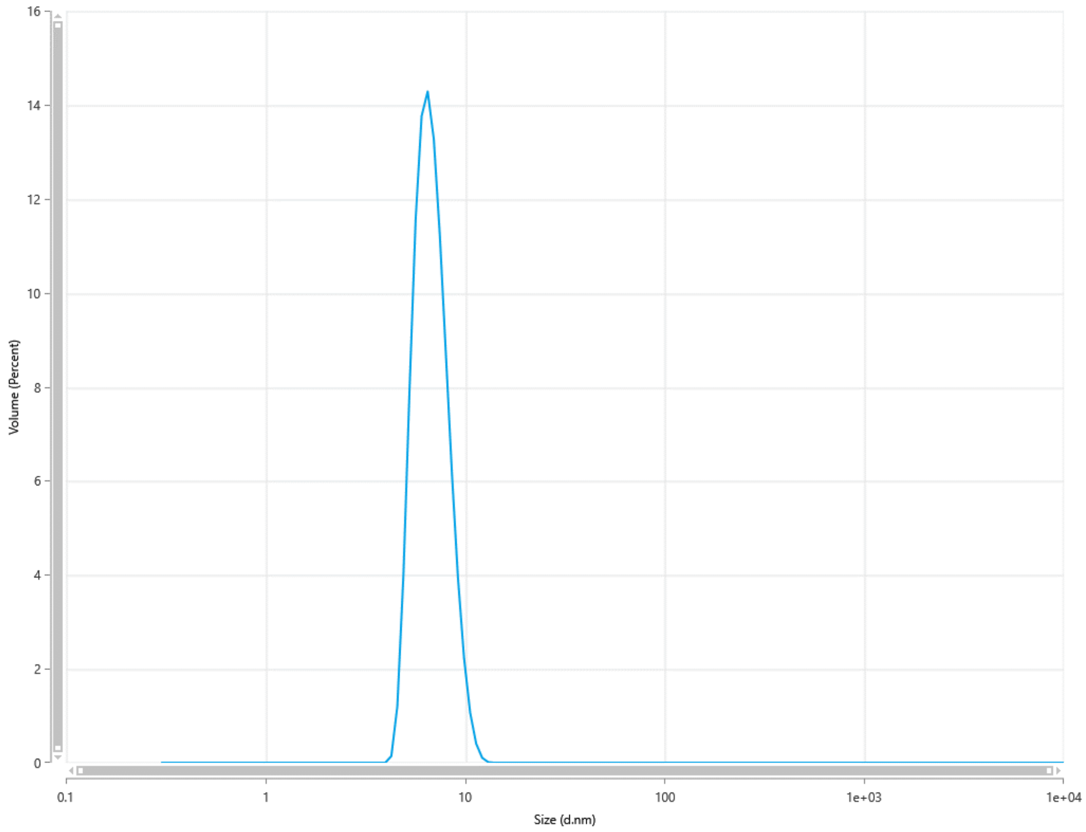

Figure S31. Volume-weighted size distribution of G_3-3-3-3-N.

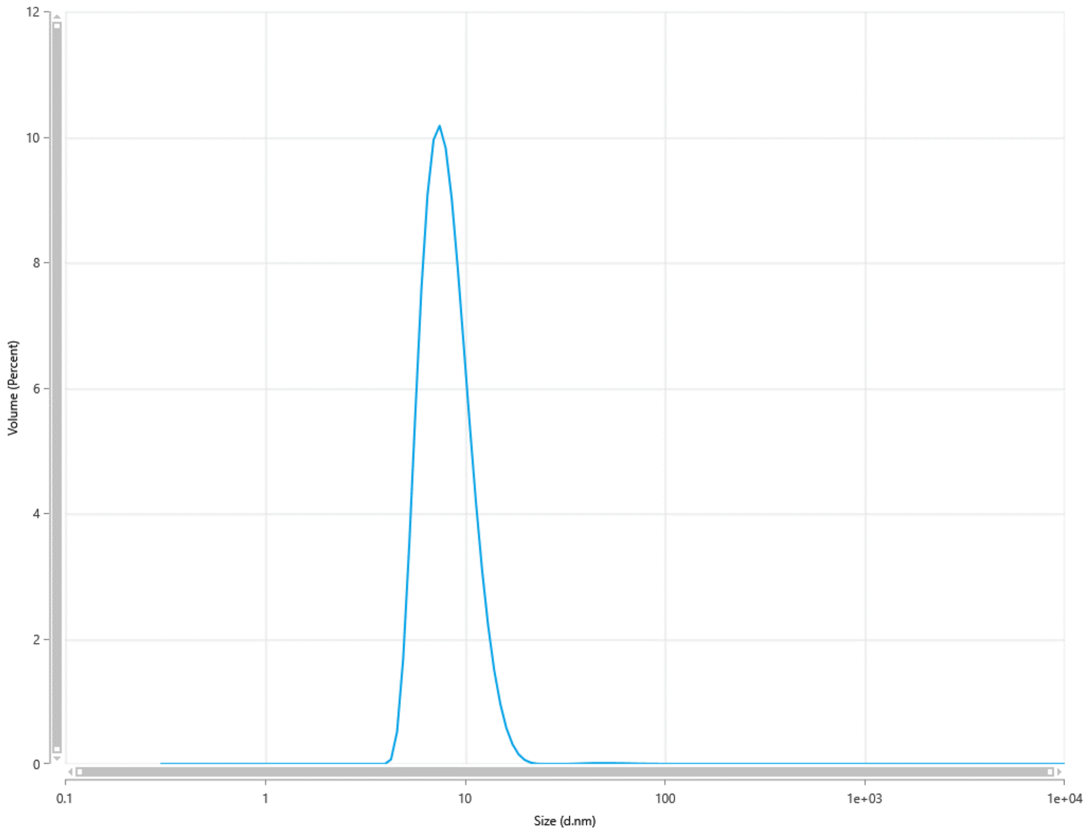

Figure S32. Volume-weighted size distribution of G_3-3-6-3-N.

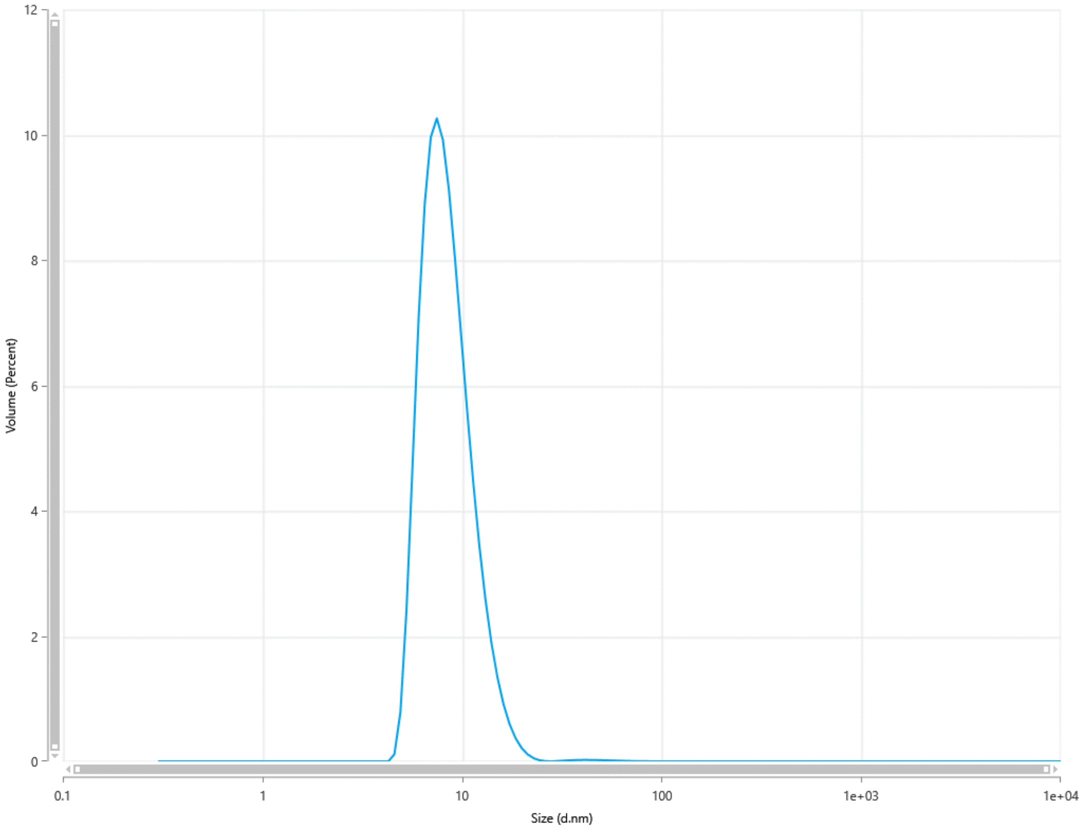

Figure S33. Volume-weighted size distribution of G_3-3-3-6-N.

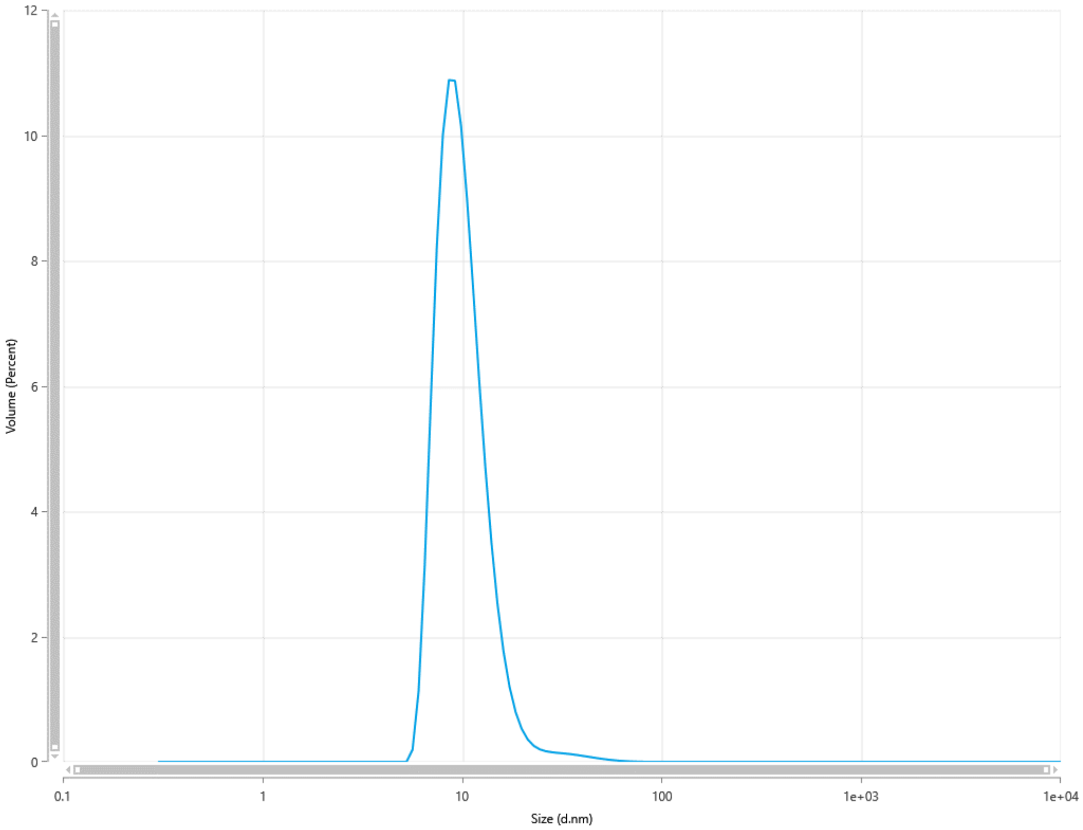

Figure S34. Volume-weighted size distribution of G_3-3-6-6-N.

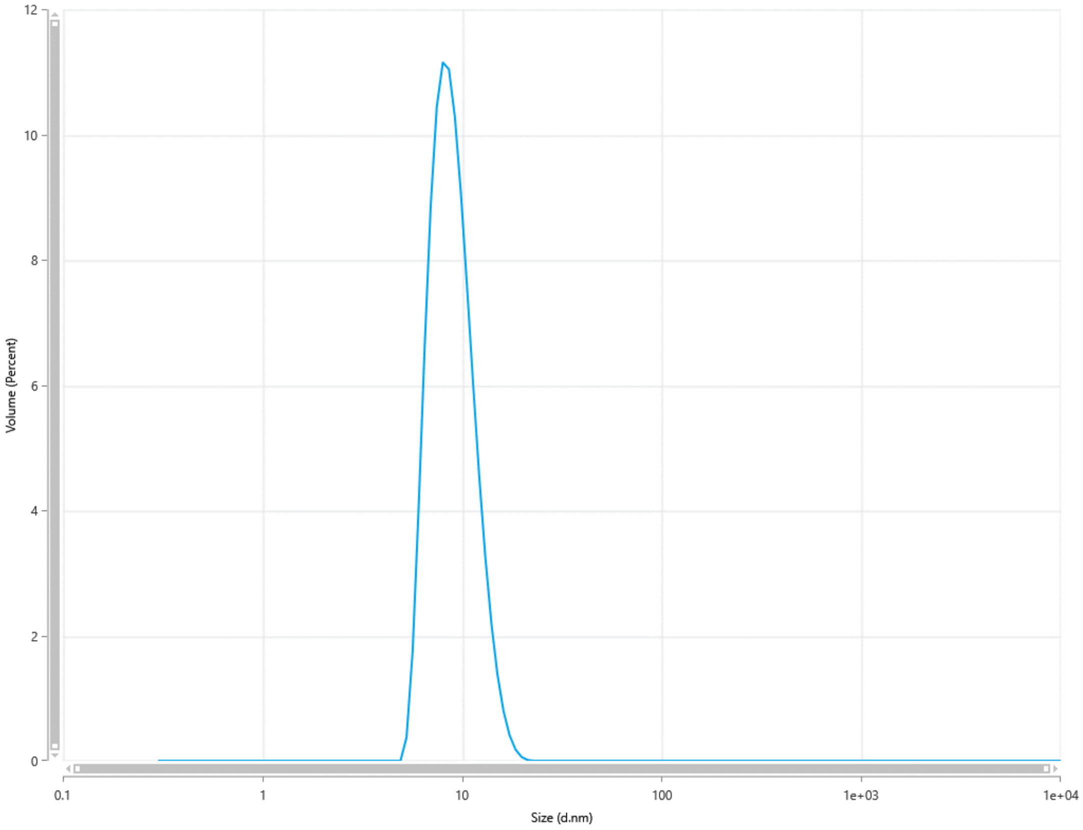

Figure S35. Volume-weighted size distribution of G_3-6-3-3-N.

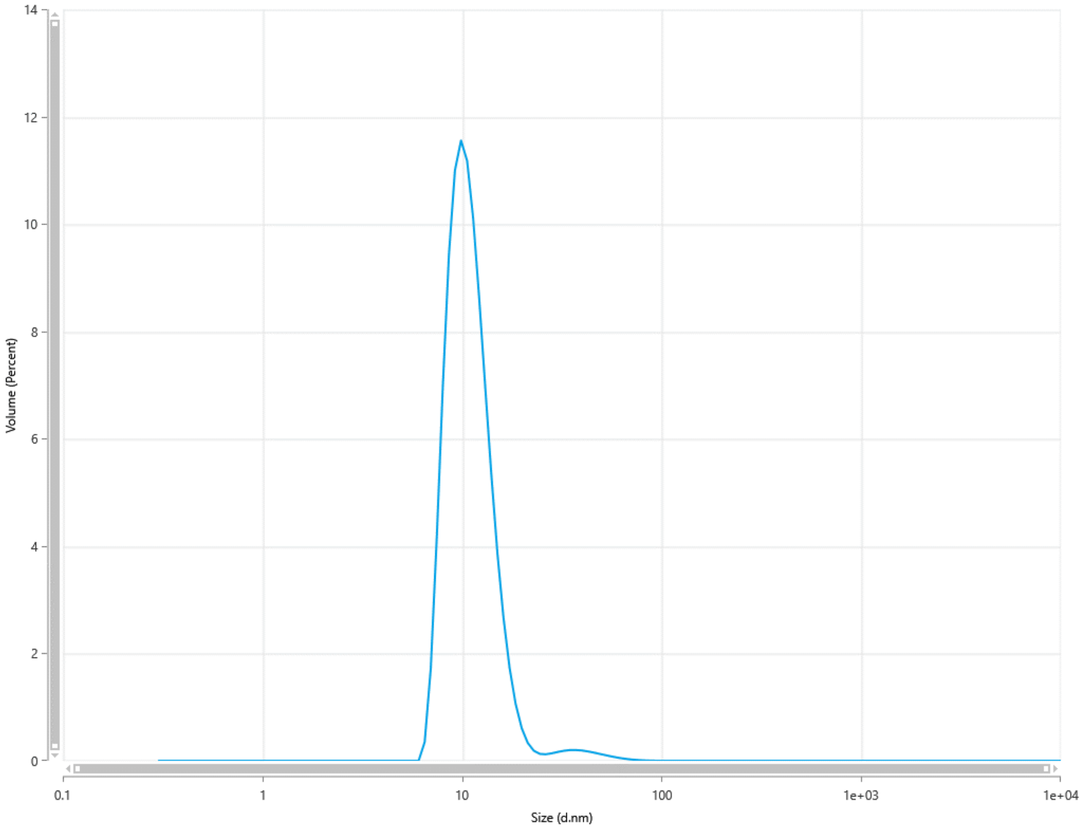

Figure S36. Volume-weighted size distribution of G_3-6-6-3-N.

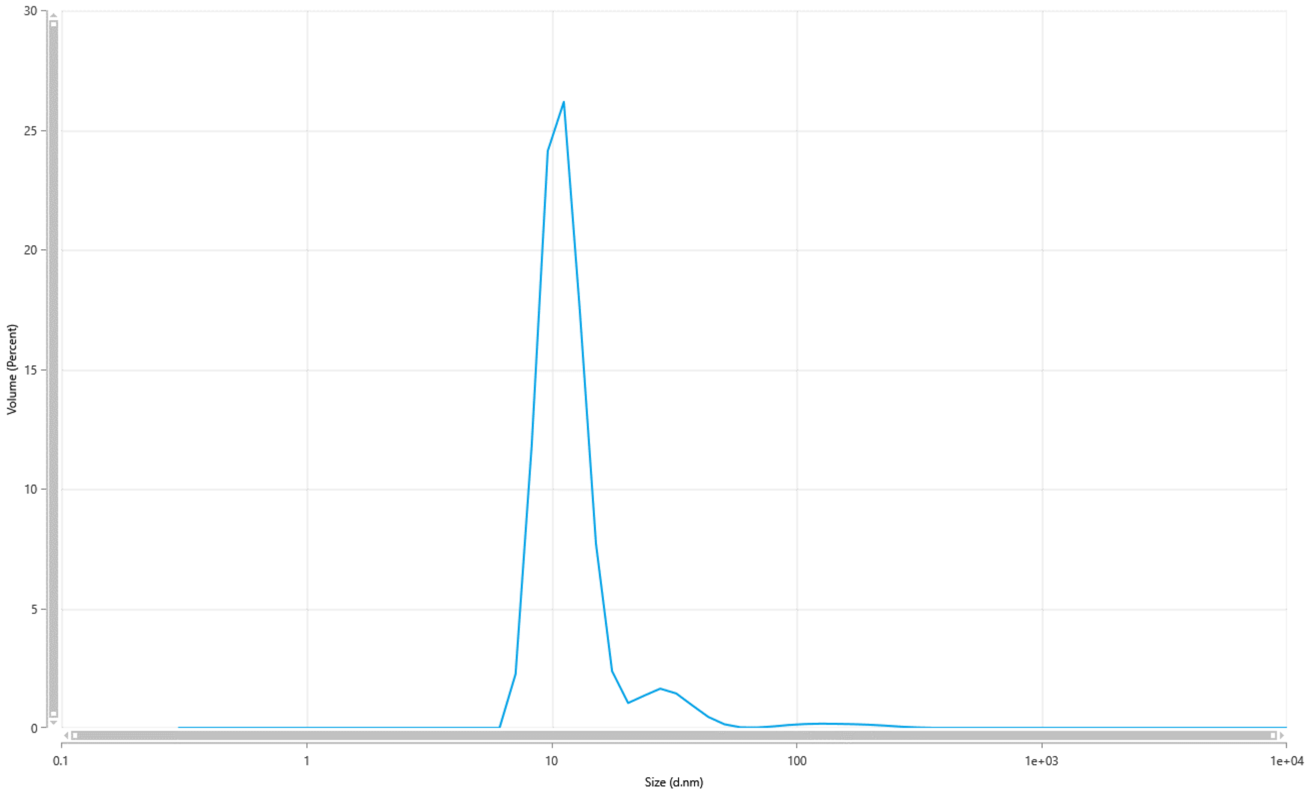

Figure S37. Volume-weighted size distribution of G_3-6-3-6-N.

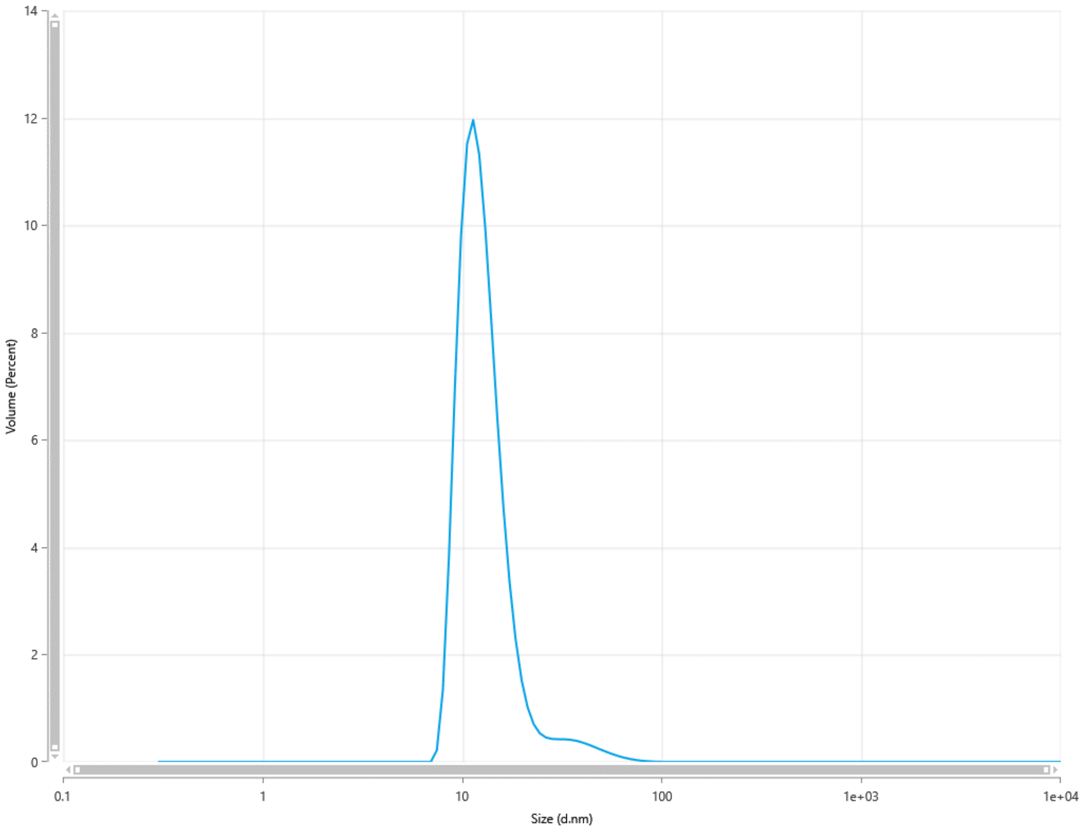

Figure S38. Volume-weighted size distribution of G_3-6-6-6-N.

5. Diffusion NMR

^{1}H NMR diffusion data at 25 °C were acquired using Bruker Avance 500 IIIHD NMR spectrometer at magnetic field of 11.75 T with Micro-5 probehead containing ^{1}H, ^{2}H radiofrequency (RF) insert and Diff50 *z*-gradient coil. ^{2}H field-frequency lock was used. The magnetic field gradient pulses were generated by Bruker GREAT accessory. The samples were prepared by dissolving 10 mg of dendrimer in 0.5 ml of CD_3OD stock solvent, which contained 1.98 mg NaCl per 1 ml of CD_3OD (i.e., NaCl concentration was 33.88 mmol.dm^{-3}). If the prepared solution appeared turbid, it was further 1/10 diluted by mixing 20 µl of the solution with 180 µl of CD_3OD stock solvent. The samples were placed into 5 mm outer diameter Shigemi tubes so that the sample lengths did not exceed 1 cm. The stimulated echo pulse sequence with 20 ms diffusion delay and 5 ms longitudinal eddy-current delay was used (these delays contained weak purging 2 ms gradient pulses). The delay between the first two RF pulses in the pulse sequence was 2.7 ms.[3,4] The gradient pulses framing the diffusion time were of sine bell shape of 1.576 ms duration. The gradient intensity was incremented in 32 steps up to 4.26 T/m at the shape maximum. The delay between subsequent scans including acquisition was 3.6 s (16 scans per measurement).

The data processing was carried out within Bruker Topspin 3.2 software. Integral intensities of separable spectral regions were subject to 1-component and 2-component exponential (Gaussian) decay fittings. The 2-component fits were found justified only for the cases of some solute/solvent peaks overlaps. Thus, only the results of 1-component analyses are presented. The self-diffusion coefficients are obtained as intensity (i.e., the spectral region integral) weighted averages of the coefficients corresponding to 7-10 spectral regions (peaks). There were smaller numbers of useful peaks in cases of the spectra of 1/10 dilute solutions. The presented diffusion coefficients are averages of 3-5 subsequent identical experiments.

6. Asymmetric Flow Field-Flow Fractionation (A4F)

The number-average molar masses (M_n) and dispersities ($Đ$) of the samples were measured by A4F. The solvent and sample delivery part of the system consisted of an Agilent G1310A pump, a G1322A degasser, and a G1329A autosampler. The field-flow fractionation long channel was assembled with a 350 µm spacer and a regenerated cellulose membrane with a cutoff of 10 000 g/mol. Three detectors were used in series: a Spectromonitor 3200 UV/VIS unit (Thermo Separation Products, Fremont, USA), a Wyatt Optilab-rEX RI detector, and a Wyatt Dawn 8+ multiangle light-scattering unit. Wyatt software Astra V (version 5.3.4.15) controlled all system components through a Wyatt ECLIPSE 3+ unit.

Water with azide was used as a solvent. The samples were filtered through a 0.45 µm PVDF filter before injection. Molar mass measurements were conducted with a constant detector flow rate of 1.0 mL/min. The focusing time was 5 min at a cross-flow of 3.5 mL/min. The injection flow was 0.2 mL/min, and 100 µL of the sample was injected in all cases. After the focusing step, the cross-flow was linearly decreased from 2.5 mL/min to 0.1 mL/min in 35 min and was then kept constant at 0.1 mL/min for the next 15 min, followed by 15 min without cross-flow.

The water of MilliQ purity was prepared by a laboratory water purification system, Milli-Q® IQ 7000 (Merck KGaA, Darmstadt, Germany), and sodium azide (NaN_3; ≥ 99%, Lach-Ner, 0.2 g/L) as an antibacterial agent was used.

Concentration: 3.152 mg/mL

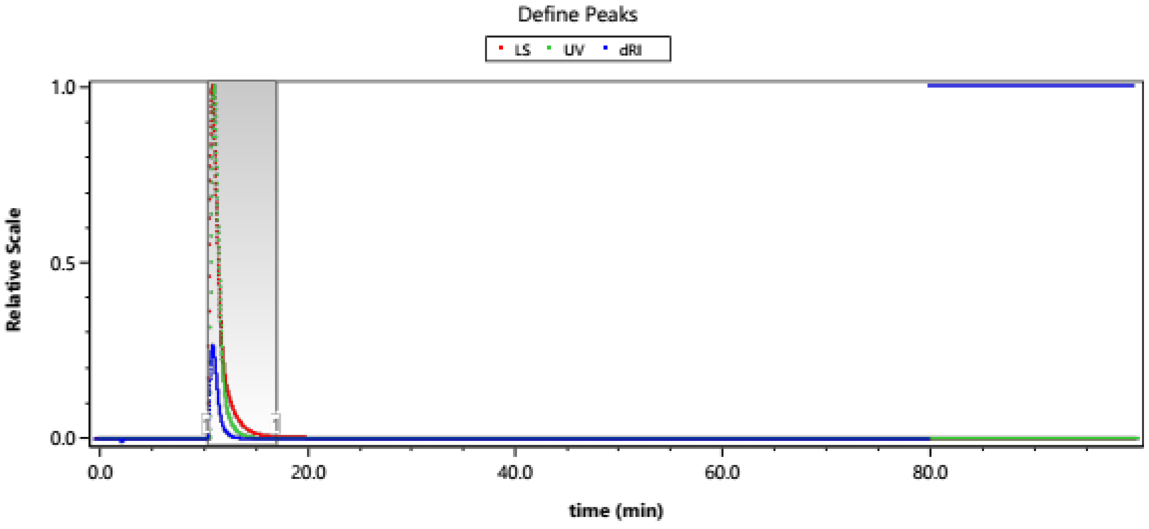

Results

Peak Results

	Peak 1
**Masses**	
Calculated Mass (µg)	315.18
Mass Recovery (%)	100.0
Mass Fraction (%)	100.0
**Molar mass moments (g/mol)**	
Mn	3.156×10^{4} (±2.328%)
Mp	2.732×10^{4} (±1.952%)
Mv	n/a
Mw	3.512×10^{4} (±2.443%)
Mz	4.614×10^{4} (±5.971%)
Mz+1	8.089×10^{4} (±6.447%)
M(avg)	3.919×10^{4} (±0.182%)
**Polydispersity**	
Mw/Mn	1.113 (±3.375%)
Mz/Mn	1.462 (±6.409%)
**rms radius moments (nm)**	
rn	n/a
rw	n/a
rz	5.0 (±358.0%)
r(avg)	12.4 (±3.9%)

Figure S39. A4F report of G_2-6-6-N

Concentration: 3.057 mg/mL

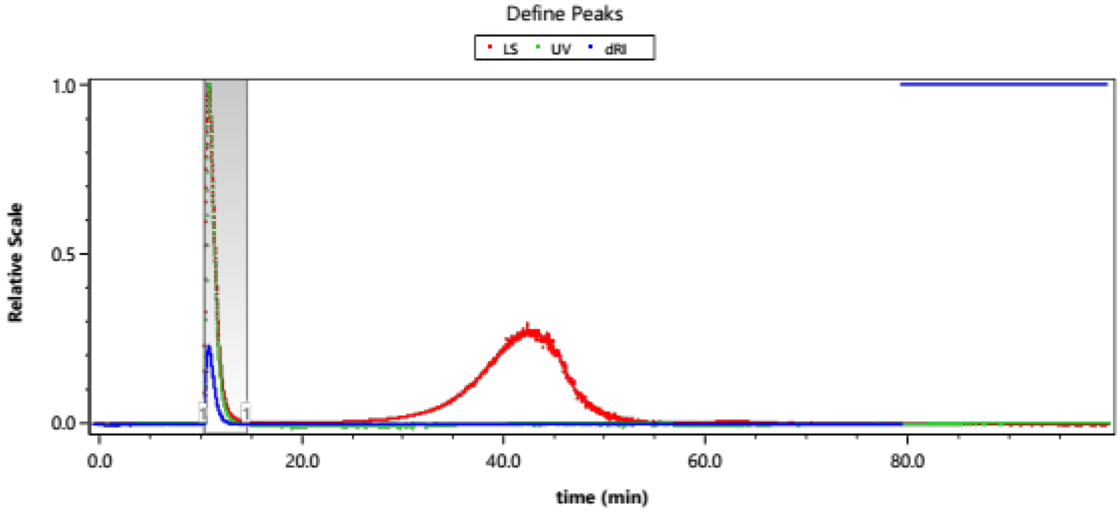

Results

Peak Results

	Peak 1
**Masses**	
Calculated Mass (μg)	305.65
Mass Recovery (%)	100.0
Mass Fraction (%)	100.0
**Molar mass moments (g/mol)**	
Mn	3.216×10^4 (±2.021%)
Mp	2.915×10^4 (±1.950%)
Mv	n/a
Mw	3.314×10^4 (±2.024%)
Mz	3.414×10^4 (±4.543%)
Mz+1	3.545×10^4 (±7.088%)
M(avg)	3.019×10^4 (±0.147%)
**Polydispersity**	
Mw/Mn	1.030 (±2.860%)
Mz/Mn	1.062 (±4.973%)
**rms radius moments (nm)**	
rn	11.6 (±59.0%)
rw	12.2 (±52.7%)
rz	12.8 (±48.4%)
r(avg)	9.5 (±7.2%)

Figure S40. A4F report of G_3-3-3-3-N

Concentration: 2.632 mg/mL

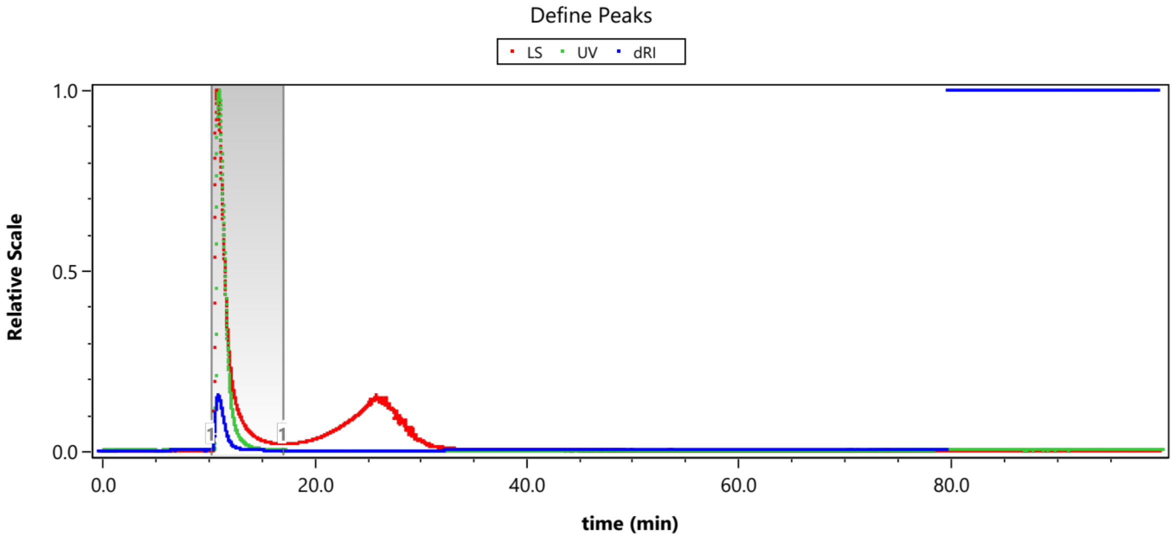

Results

Peak Results

	Peak 1
**Masses**	
**Calculated Mass (µg)**	263.20
**Mass Recovery (%)**	100.0
**Mass Fraction (%)**	100.0
**Molar mass moments (g/mol)**	
**Mn**	6.197×10^{4} (±2.743%)
**Mp**	5.748×10^{4} (±2.589%)
**Mv**	n/a
**Mw**	7.237×10^{4} (±2.163%)
**Mz**	1.153×10^{5} (±4.264%)
**Mz+1**	3.329×10^{5} (±1.991%)
**M(avg)**	8.253×10^{4} (±0.097%)
**Polydispersity**	
**Mw/Mn**	1.168 (±3.493%)
**Mz/Mn**	1.860 (±5.070%)
**rms radius moments (nm)**	
**rn**	28.7 (±18.6%)
**rw**	27.6 (±16.1%)
**rz**	27.5 (±15.4%)
**r(avg)**	23.7 (±0.6%)

Figure S41. A4F report of G_3-3-3-6-N

Concentration: 3.384 mg/mL

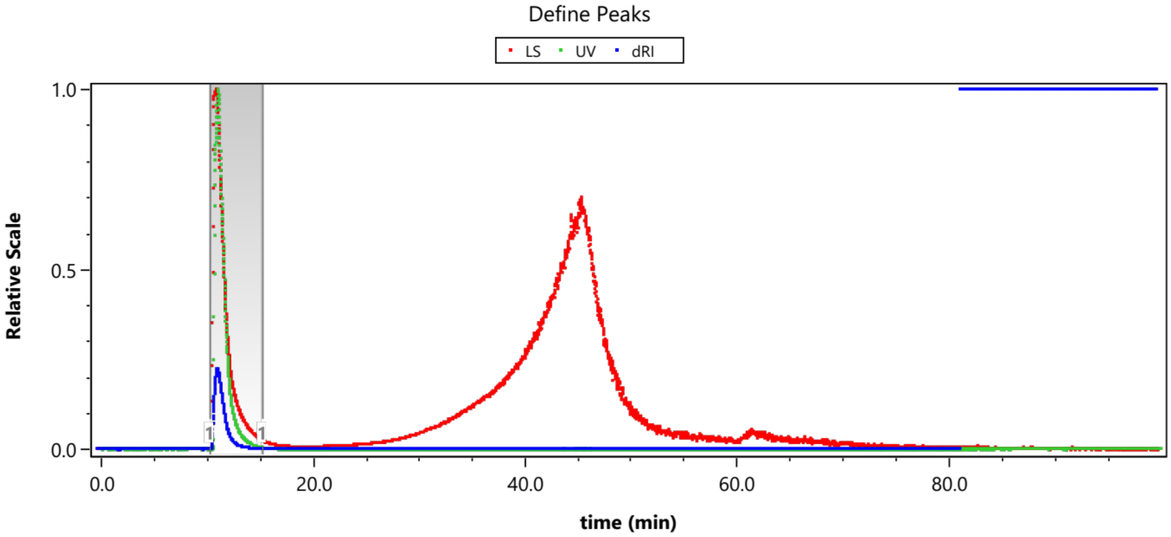

Results

Peak Results

	Peak 1
**Masses**	
**Calculated Mass (μg)**	338.41
**Mass Recovery (%)**	100.0
**Mass Fraction (%)**	100.0
**Molar mass moments (g/mol)**	
**Mn**	6.365×10^4 (±2.875%)
**Mp**	5.466×10^4 (±2.565%)
**Mv**	n/a
**Mw**	7.047×10^4 (±3.098%)
**Mz**	8.157×10^4 (±7.173%)
**Mz+1**	9.849×10^4 (±9.737%)
**M(avg)**	7.657×10^4 (±0.166%)
**Polydispersity**	
**Mw/Mn**	1.107 (±4.227%)
**Mz/Mn**	1.282 (±7.727%)
**rms radius moments (nm)**	
**rn**	21.9 (±21.0%)
**rw**	24.0 (±18.0%)
**rz**	26.4 (±15.8%)
**r(avg)**	14.0 (±2.5%)

Figure S42. A4F report of G_3-3-6-3-N

Concentration: 3.049 mg/mL

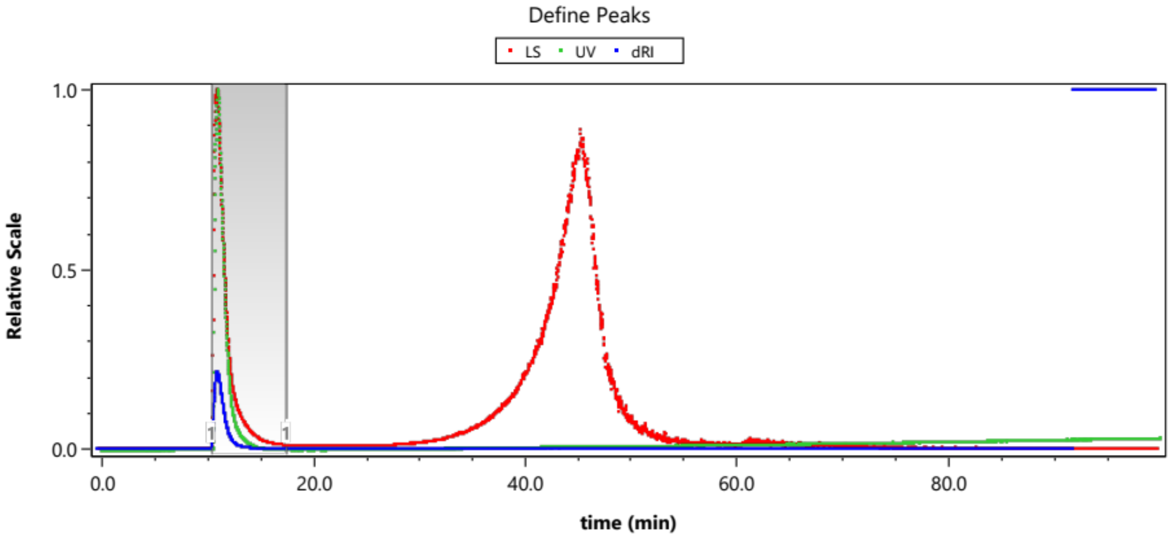

Results

Peak Results

	Peak 1
**Masses**	
**Calculated Mass (μg)**	304.92
**Mass Recovery (%)**	100.0
**Mass Fraction (%)**	100.0
**Molar mass moments (g/mol)**	
**Mn**	5.368×10^4 (±2.832%)
**Mp**	4.762×10^4 (±2.644%)
**Mv**	n/a
**Mw**	5.925×10^4 (±3.202%)
**Mz**	6.893×10^4 (±7.739%)
**Mz+1**	8.399×10^4 (±11.101%)
**M(avg)**	6.207×10^4 (±0.182%)
**Polydispersity**	
**Mw/Mn**	1.104 (±4.275%)
**Mz/Mn**	1.284 (±8.241%)
**rms radius moments (nm)**	
**rn**	9.2 (±114.9%)
**rw**	12.9 (±61.7%)
**rz**	15.8 (±44.6%)
**r(avg)**	24.2 (±4.0%)

Figure S43. A4F report of G_3-6-3-3-N

Concentration: 2.836 mg/mL

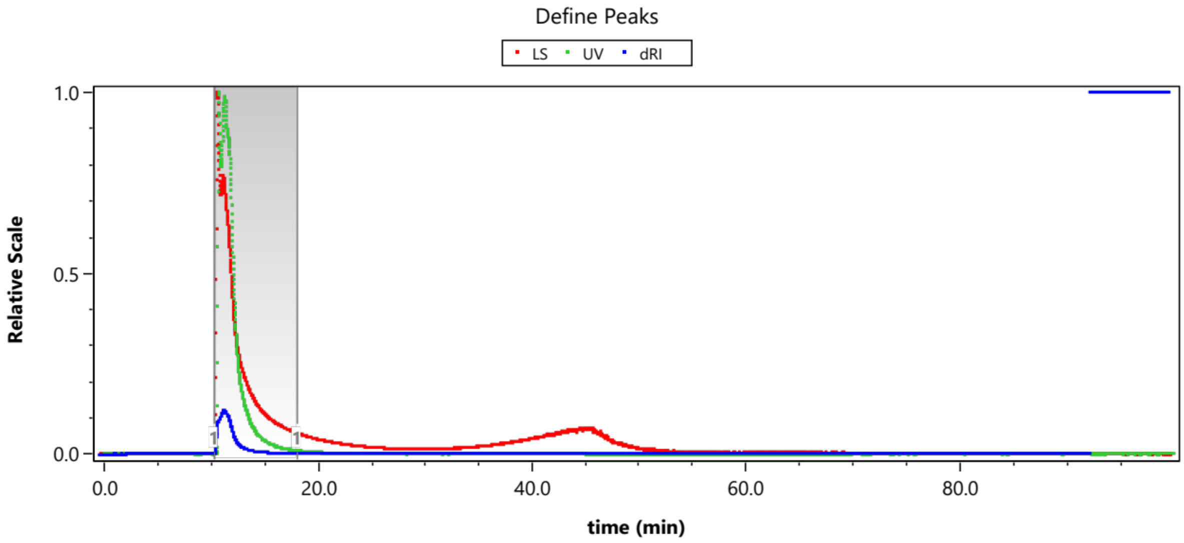

Results

Peak Results

	Peak 1
**Masses**	
**Calculated Mass (µg)**	283.63
**Mass Recovery (%)**	100.0
**Mass Fraction (%)**	100.0
**Molar mass moments (g/mol)**	
**Mn**	1.079×10^{5} (±1.980%)
**Mp**	8.815×10^{4} (±1.791%)
**Mv**	n/a
**Mw**	1.312×10^{5} (±2.105%)
**Mz**	1.859×10^{5} (±4.668%)
**Mz+1**	2.855×10^{5} (±4.744%)
**M(avg)**	1.350×10^{5} (±0.106%)
**Polydispersity**	
**Mw/Mn**	1.215 (±2.890%)
**Mz/Mn**	1.722 (±5.071%)
**rms radius moments (nm)**	
**rn**	4.9 (±291.1%)
**rw**	8.1 (±111.4%)
**rz**	10.2 (±71.9%)
**r(avg)**	8.8 (±7.0%)

Figure S44. A4F report of G_3-3-6-6-N

Concentration: 3.229 mg/mL

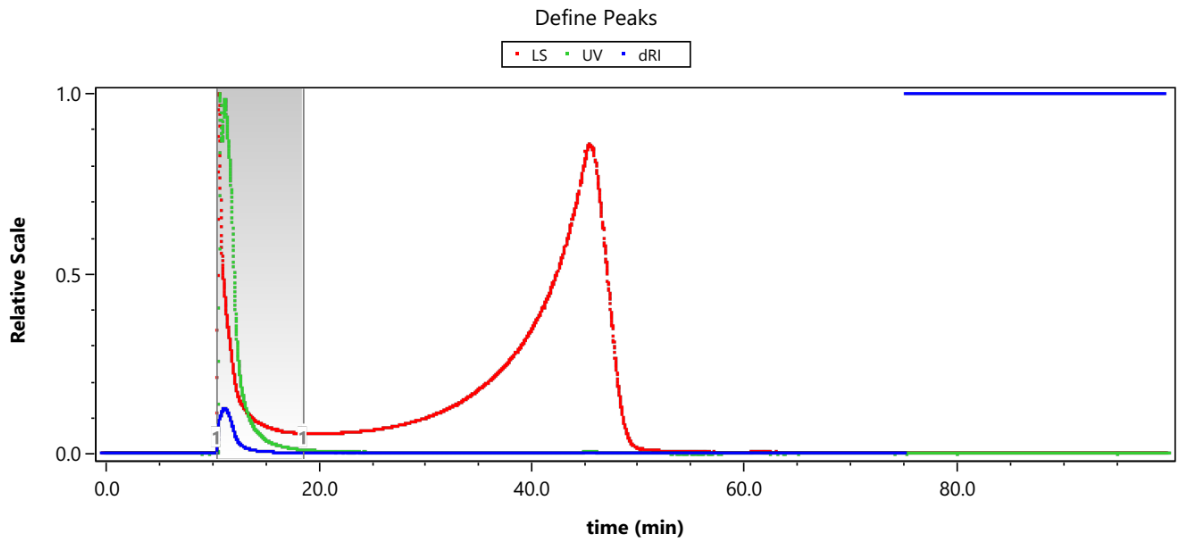

Results

Peak Results

	Peak 1
**Masses**	
Calculated Mass (µg)	322.90
Mass Recovery (%)	100.0
Mass Fraction (%)	100.0
**Molar mass moments (g/mol)**	
Mn	1.675×10^{5} (±3.350%)
Mp	1.372×10^{5} (±3.597%)
Mv	n/a
Mw	2.426×10^{5} (±4.041%)
Mz	4.202×10^{5} (±9.184%)
Mz+1	6.880×10^{5} (±8.657%)
M(avg)	1.900×10^{5} (±0.180%)
**Polydispersity**	
Mw/Mn	1.448 (±5.250%)
Mz/Mn	2.509 (±9.776%)
**rms radius moments (nm)**	
rn	22.5 (±21.2%)
rw	28.2 (±15.0%)
rz	34.5 (±11.2%)
r(avg)	13.5 (±2.4%)

Figure S45. A4F report of G_3-6-3-6-N

Concentration: 3.311 mg/mL

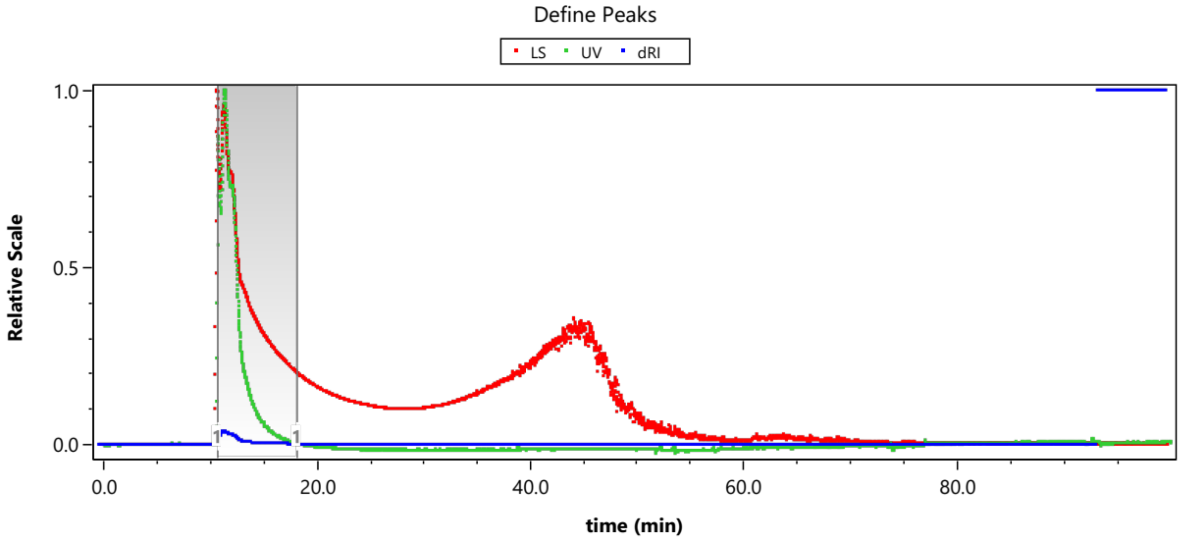

Results

Peak Results

	Peak 1
**Masses**	
**Calculated Mass (μg)**	331.06
**Mass Recovery (%)**	100.0
**Mass Fraction (%)**	100.0
**Molar mass moments (g/mol)**	
**Mn**	4.165×10^{5} (±1.713%)
**Mp**	3.288×10^{5} (±1.670%)
**Mv**	n/a
**Mw**	5.678×10^{5} (±1.778%)
**Mz**	1.034×10^{6} (±4.078%)
**Mz+1**	1.820×10^{6} (±3.804%)
**M(avg)**	4.732×10^{5} (±0.109%)
**Polydispersity**	
**Mw/Mn**	1.363 (±2.469%)
**Mz/Mn**	2.482 (±4.424%)
**rms radius moments (nm)**	
**rn**	7.7 (±120.3%)
**rw**	8.2 (±105.9%)
**rz**	9.6 (±80.4%)
**r(avg)**	10.3 (±3.4%)

Figure S46. A4F report of G_3-6-6-3-N

Concentration: 2.877 mg/mL

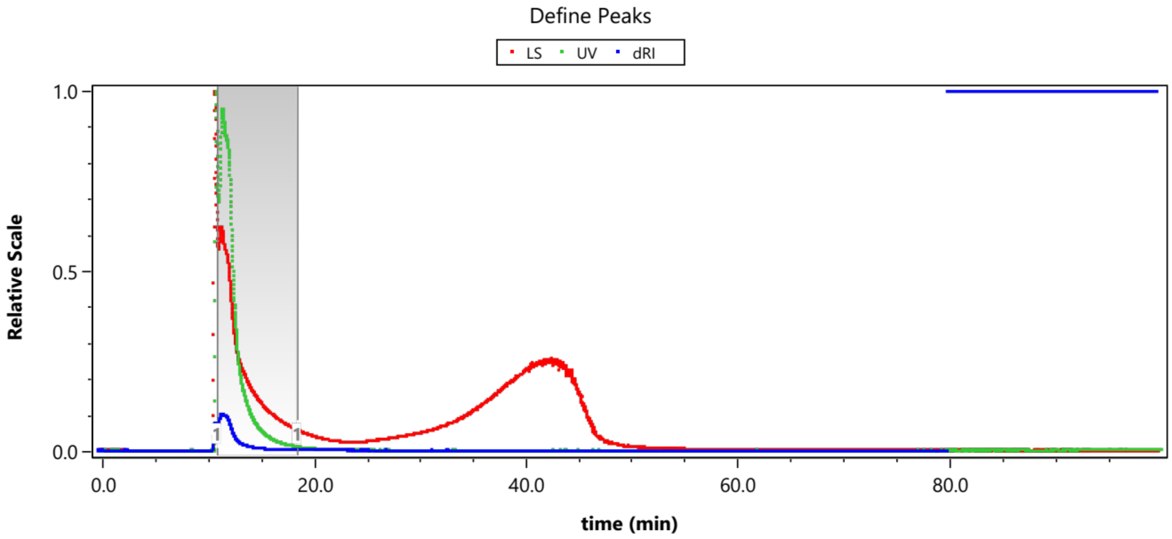

Results

Peak Results

	Peak 1
**Masses**	
**Calculated Mass (μg)**	287.67
**Mass Recovery (%)**	100.0
**Mass Fraction (%)**	100.0
**Molar mass moments (g/mol)**	
**Mn**	2.321×10^5 (±2.413%)
**Mp**	1.928×10^5 (±2.384%)
**Mv**	n/a
**Mw**	2.930×10^5 (±2.435%)
**Mz**	4.695×10^5 (±5.476%)
**Mz+1**	8.062×10^5 (±5.173%)
**M(avg)**	2.904×10^5 (±0.145%)
**Polydispersity**	
**Mw/Mn**	1.263 (±3.428%)
**Mz/Mn**	2.023 (±5.984%)
**rms radius moments (nm)**	
**rn**	6.2 (±236.5%)
**rw**	6.8 (±196.5%)
**rz**	7.8 (±150.3%)
**r(avg)**	10.2 (±4.8%)

Figure S47. A4F report of G_3-6-6-6-N

7. Molecular modelling

3D computer models of dendrimers were created using dendrimer builder, as implemented in the Materials Studio software package from BIOVIA (formerly Accelrys). The AM1-BCC technique[5] was used to calculate the partial charges of dendrimer atoms. For this charge parametrization, the Amber program Antechamber was used. The GAFF force field (General Amber Force Field)[6] was used for parameterization of all dendrimer structures. Missing force constants and energy barriers for "Si containing" force field terms were fitted by minimizing the differences between QM and force field based relative energies of 100 configurations of properly chosen molecular fragment (i.e. ff parameters that most accurately ensures the following requirement were used: $E_{i\_force\text{-}field} = E_{i\_quantum} + K$ for all configurations i - where $E_{i\_force\text{-}field}$ and $E_{i\_quantum}$ are force field and QM based energy of molecular configuration i and K is constant). Equilibrium values of "Si-containing" bonds and angles were obtained using QM optimization of the given molecular fragment. QM energies were calculated at MP2/HF/6-31G** level of theory using GAMESS[7], and fitting was accomplished using the paramfit routine from AMBER software.[8] Slightly adjusted van der Waals parameters for Si atoms from MM3 force field[9] were used in this study. All the calculated and used values of the-Si-containing force field parameters are available in the supporting information of our previous work.[10] Dendrimers were solvated in methanol in a cube simulation box – see Fig. S48. The minimal distance from dendrimer atoms to the wall of the simulation box was set to 20 Å. A proper number of Cl^- and Na^+ ions was added to preserve the neutrality of the system and to ensure the bulk concentration used in the DLS experiment (0.25 % NaCl solution in MeOH). First, the systems were minimized (5000 steps with 2 kcal/(mol Å^2) restraint + 50000 without restraint), heated (200 ps NVT) to 298 K and equilibrated using 20 ns (for G_1,G_2) and 30 ns (for G_3) long molecular dynamics simulations (NPT, T = 298 K , P = 0.1 MPa). The first 0.5 ns with restrained solute. Hydrogens were constrained with the SHAKE algorithm to allow 2 fs time step[11] and a Langevin thermostat with collision frequency 2 ps^{-1} was used for all MD runs.[12] The pressure relaxation time for weak-coupling barostat was 2 ps. Particle mesh Ewald method (PME) was used to treat long range electrostatic interactions under periodic conditions with a direct space cutoff of 10 Å. The same cutoff was used for van der Waals interactions. The pmemd.cuda module[13] from Amber20 package was used for all simulation steps. All, third generation dendrimers and some of the second generation dendrimers (G_2-6-3-N, G_2-6-6-N) were before simulation in MeOH first shortly simulated in vacuum, to obtain more extended structures (thanks to the positive net charge), which almost eliminated eventual partly intertwined branches problem of the initial structures. The eventual remaining problems were solved manually on these extended structures by proper changes of relevant bond angles or torsional angles. Structural analyses (RDF, Rg, Rmax) were performed using the CPPTRAJ routine from Amber. The last 5 ns of the MD trajectories were used for these analyses. UCSF Chimera software was used for all visualizations as well as for the manual corrections of the dendrimer structures.[14]

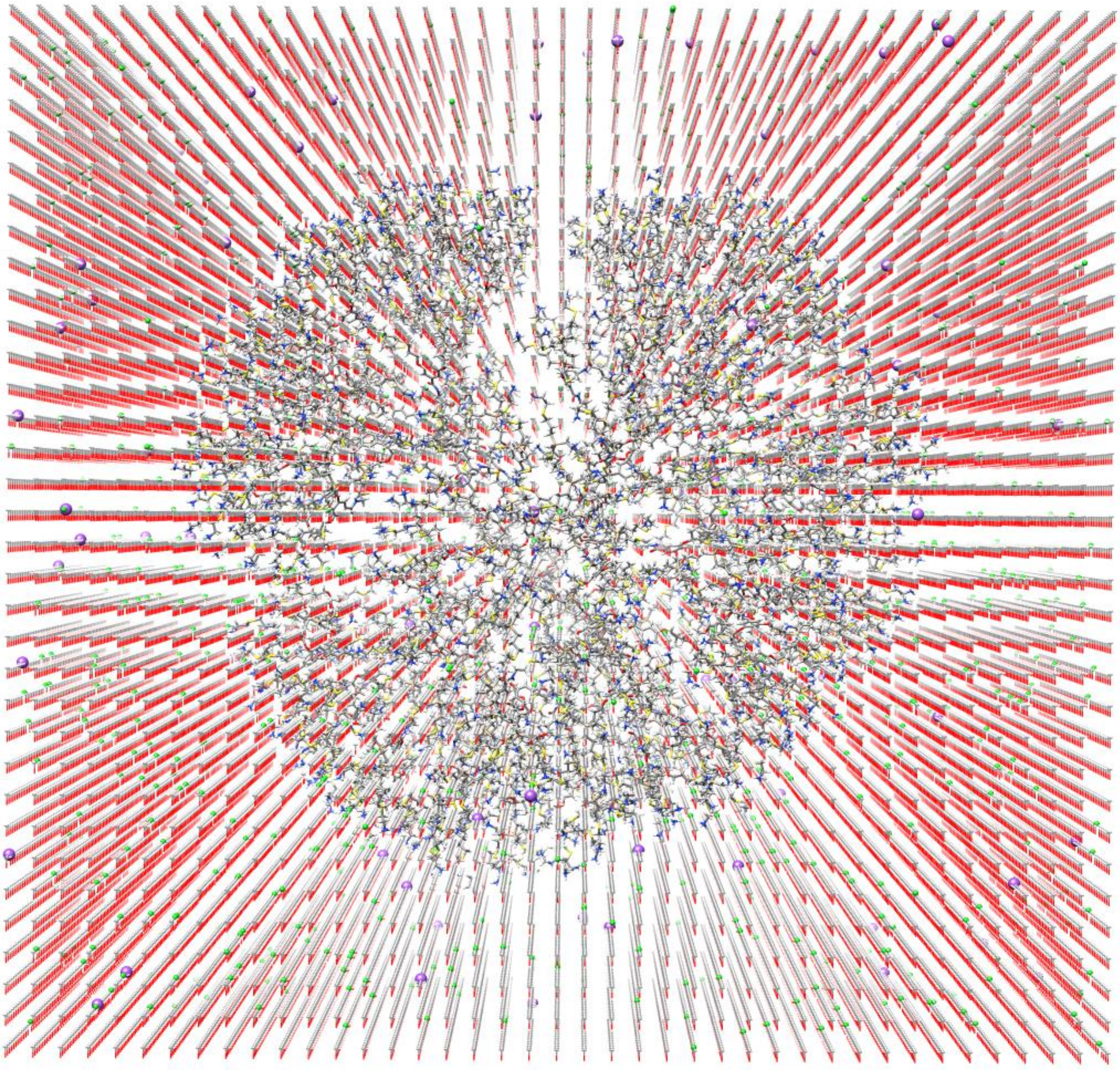

Figure S48. Example of the initial simulation box with dendrimer (G_3-6-6-6-N) solvated in MeOH with Na^+, Cl^- ions. Colors: C – grey/black; O – red; H – white; Si – beige; N – blue; S – yellow, Cl^- anions – green, Na^+ cations - purple.

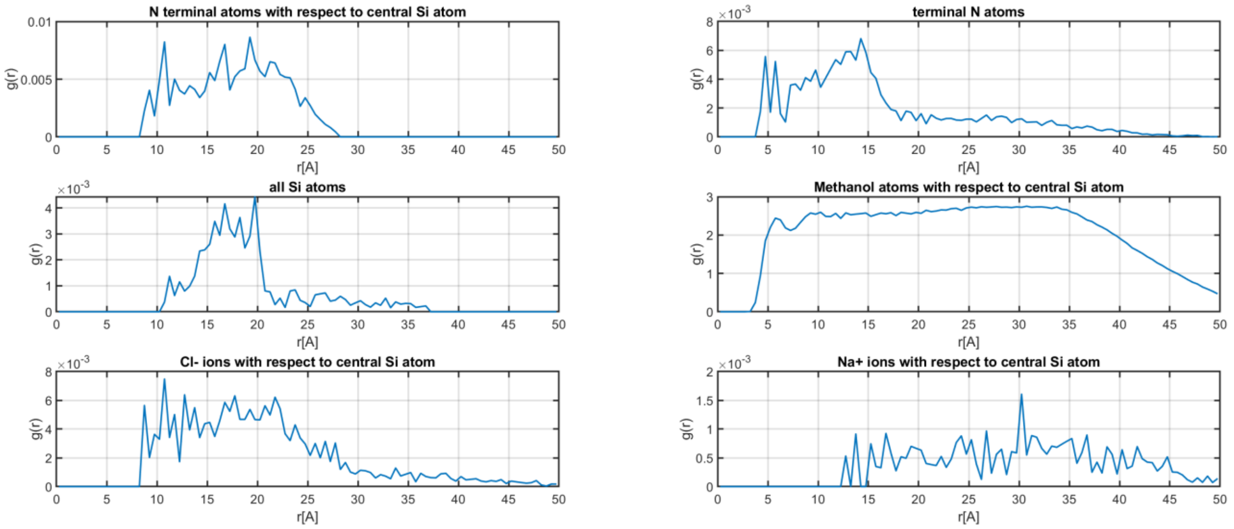

Figure S49. Radial distribution functions (relative density profiles (RDF)) of selected atoms, with respect to central Si atom, or just RDF of the given homogenous set of atoms, in case of simulated dendrimer G_1-3-N.

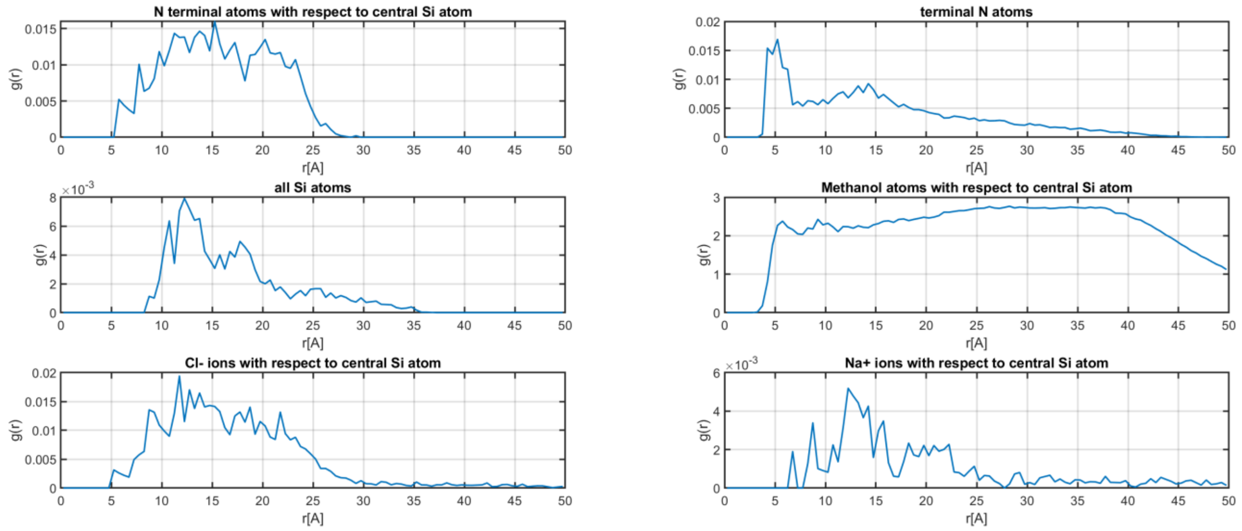

Figure S50. Radial distribution functions (relative density profiles (RDF)) of selected atoms, with respect to central Si atom, or just RDF of the given homogenous set of atoms, in case of simulated dendrimer G_1-6-N.

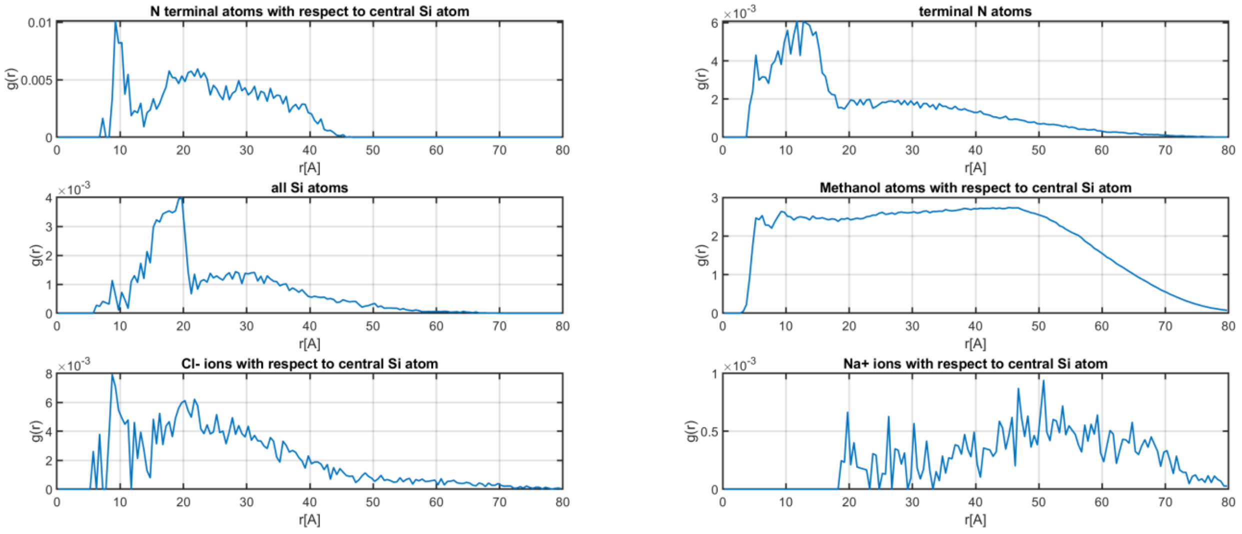

Figure S51. Radial distribution functions (relative density profiles (RDF)) of selected atoms, with respect to central Si atom, or just RDF of the given homogenous set of atoms, in case of simulated dendrimer G_2-3-3-N.

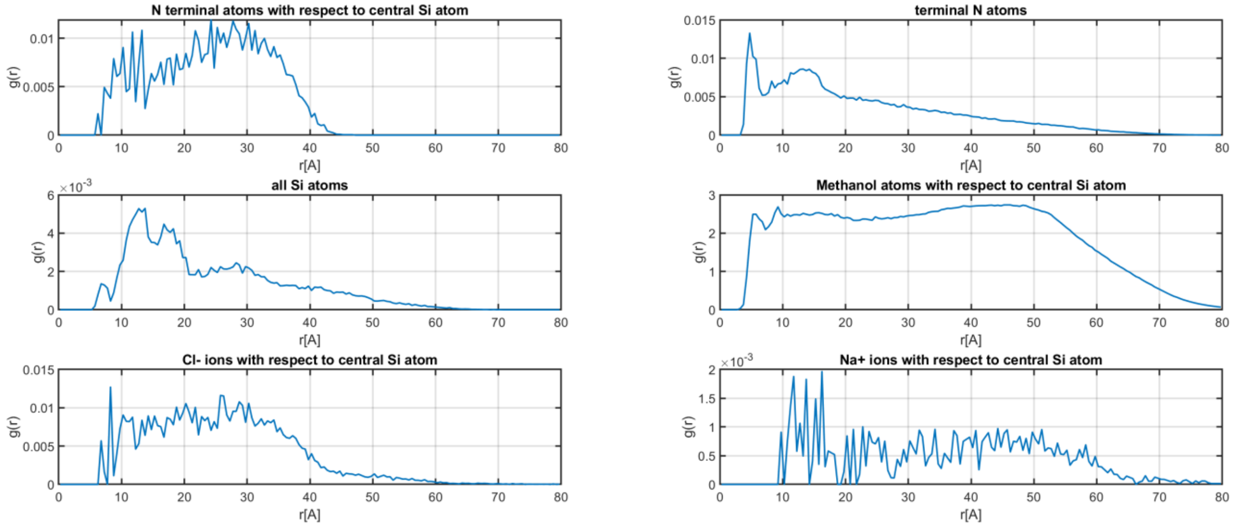

Figure S52.: Radial distribution functions (relative density profiles (RDF)) of selected atoms, with respect to central Si atom, or just RDF of the given homogenous set of atoms, in case of simulated dendrimer G_2-3-6-N.

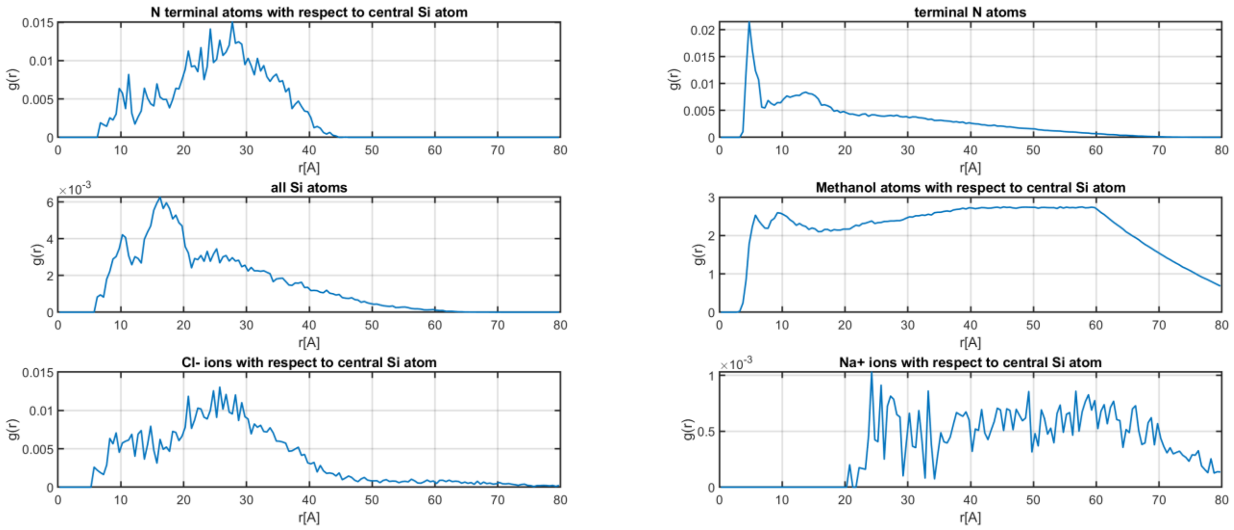

Figure S53. Radial distribution functions (relative density profiles (RDF)) of selected atoms, with respect to central Si atom, or just RDF of the given homogenous set of atoms, in case of simulated dendrimer G_2-6-3-N.

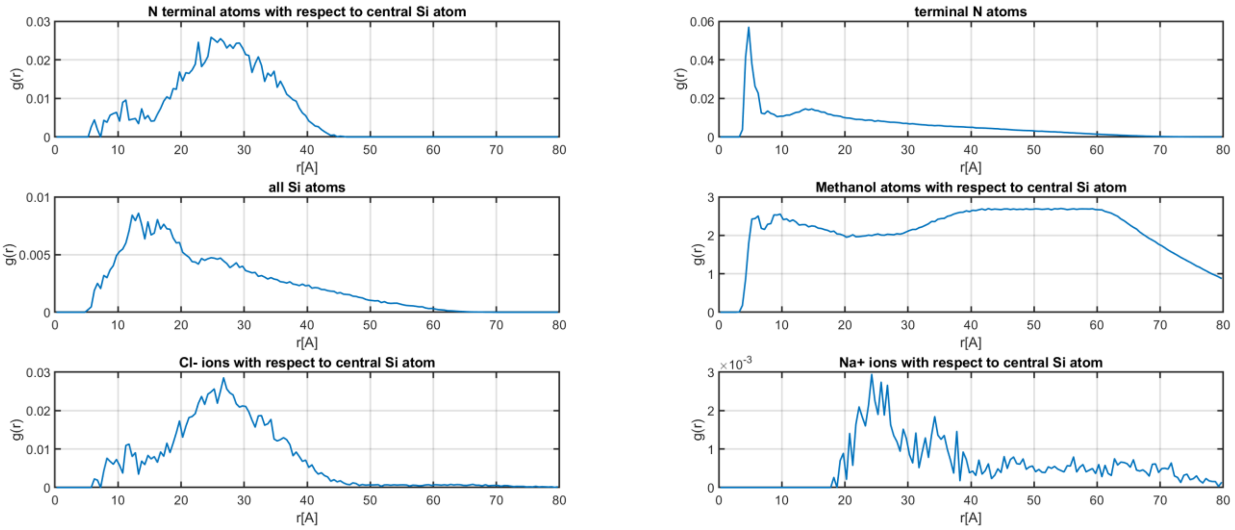

Figure S54. Radial distribution functions (relative density profiles (RDF)) of selected atoms, with respect to central Si atom, or just RDF of the given homogenous set of atoms, in case of simulated dendrimer G_2-6-6-N.

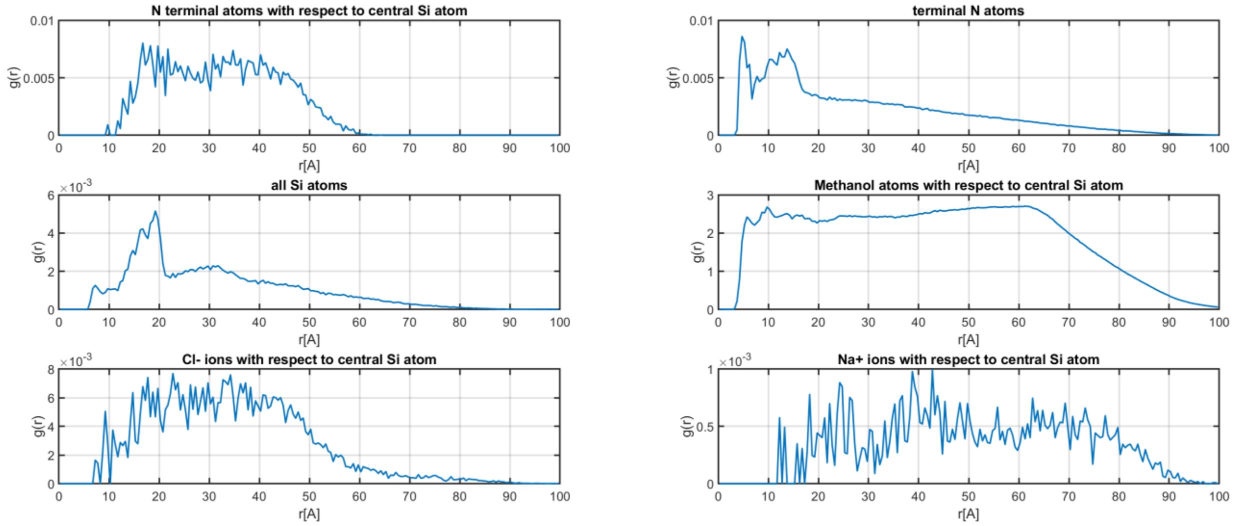

Figure S55. Radial distribution functions (relative density profiles (RDF)) of selected atoms, with respect to central Si atom, or just RDF of the given homogenous set of atoms, in case of simulated dendrimer G_3-3-3-3-N.

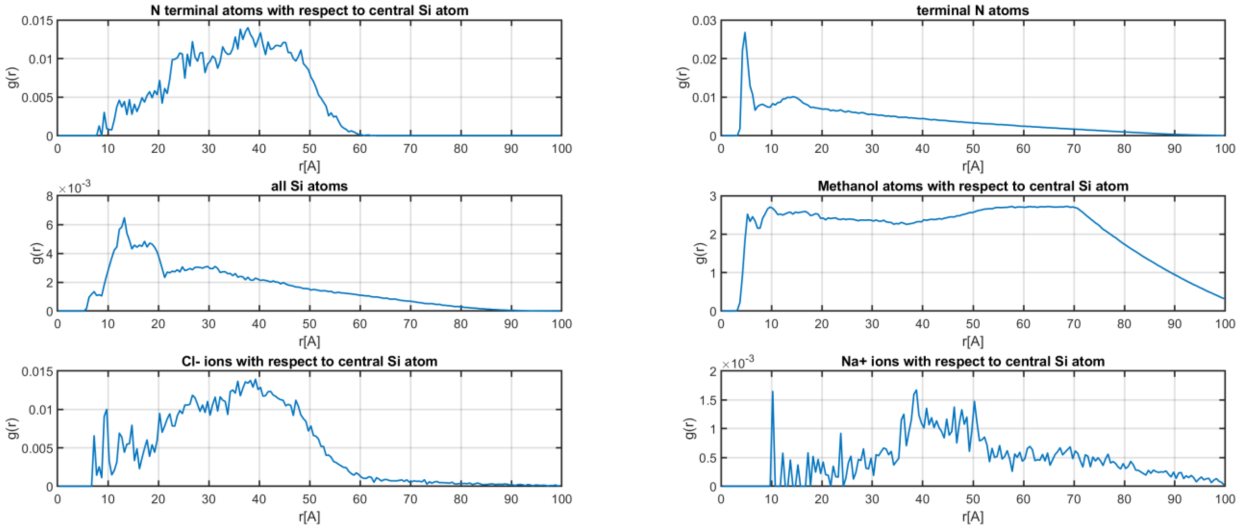

Figure S56. Radial distribution functions (relative density profiles (RDF)) of selected atoms, with respect to central Si atom, or just RDF of the given homogenous set of atoms, in case of simulated dendrimer G_3-3-3-6-N.

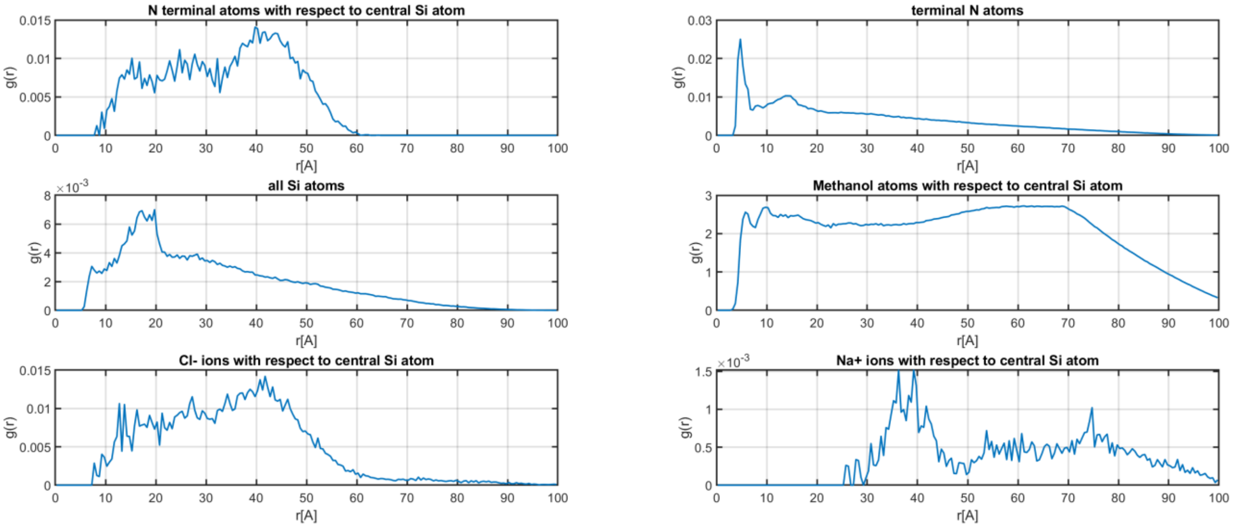

Figure S57. Radial distribution functions (relative density profiles (RDF)) of selected atoms, with respect to central Si atom, or just RDF of the given homogenous set of atoms, in case of simulated dendrimer G_3-3-6-3-N.

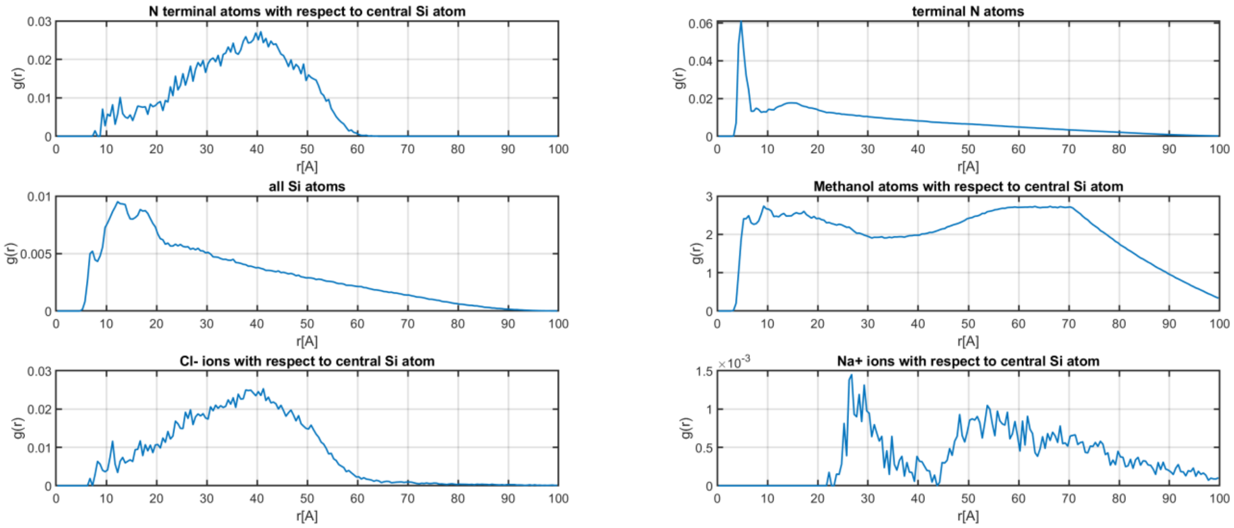

Figure S58. Radial distribution functions (relative density profiles (RDF)) of selected atoms, with respect to central Si atom, or just RDF of the given homogenous set of atoms, in case of simulated dendrimer G_3-3-6-6-N.

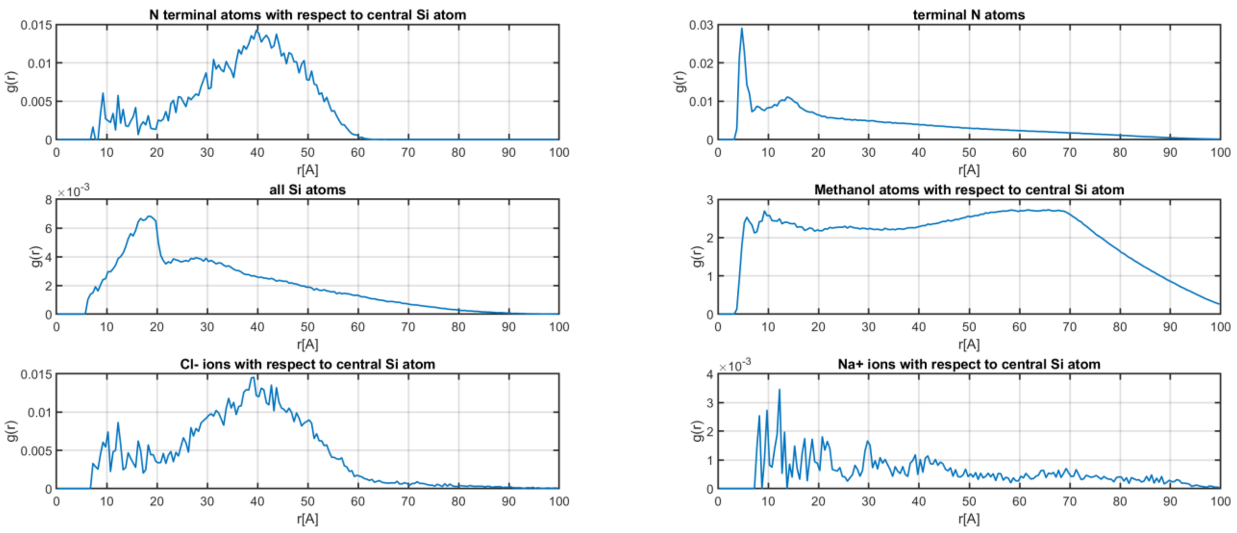

Figure S59. Radial distribution functions (relative density profiles (RDF)) of selected atoms, with respect to central Si atom, or just RDF of the given homogenous set of atoms, in case of simulated dendrimer G_3-6-3-3-N.

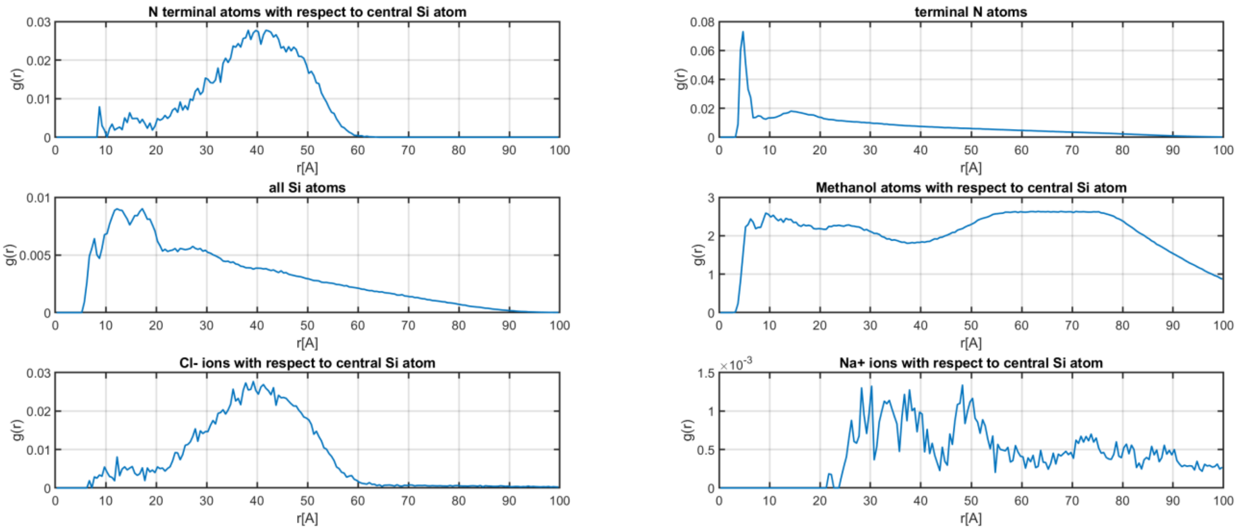

Figure S60. Radial distribution functions (relative density profiles (RDF)) of selected atoms, with respect to central Si atom, or just RDF of the given homogenous set of atoms, in case of simulated dendrimer G_3-6-3-6-N.

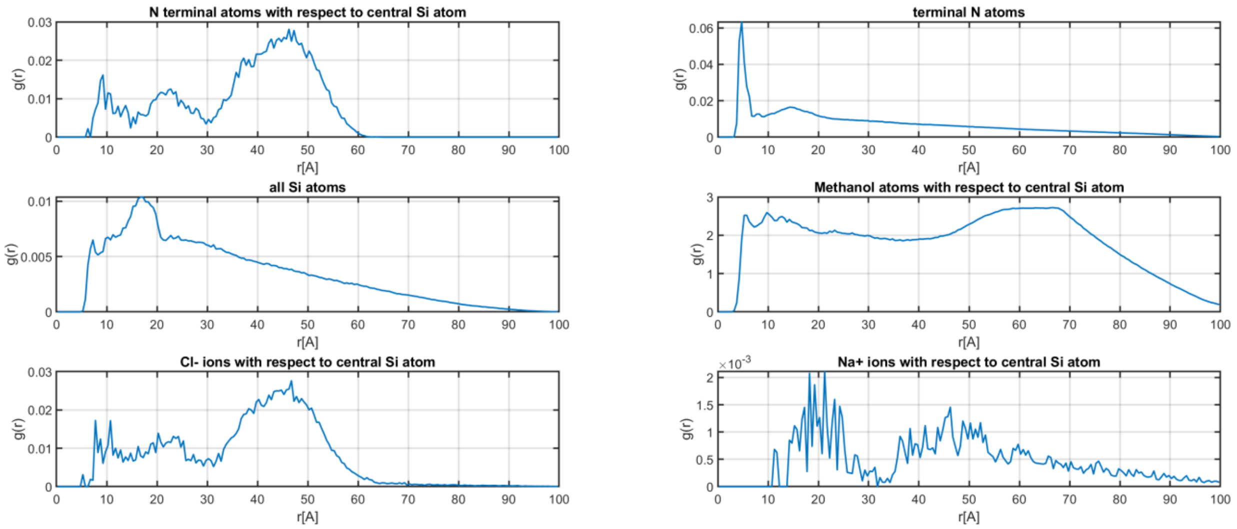

Figure S61. Radial distribution functions (relative density profiles (RDF)) of selected atoms, with respect to central Si atom, or just RDF of the given homogenous set of atoms, in case of simulated dendrimer G_3-6-6-3-N.

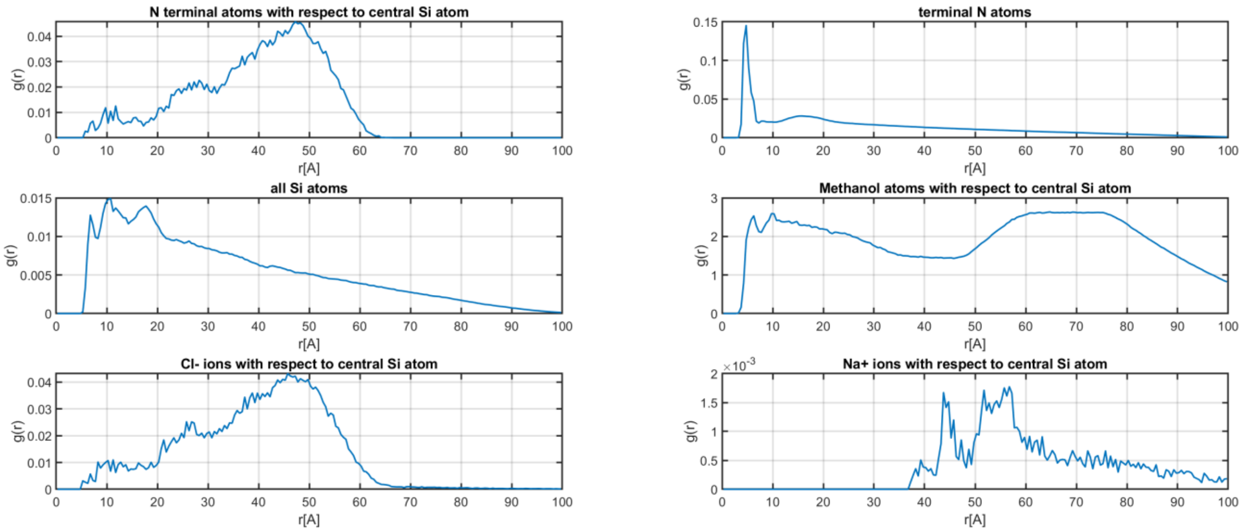

Figure S62. Radial distribution functions (relative density profiles (RDF)) of selected atoms, with respect to central Si atom, or just RDF of the given homogenous set of atoms, in case of simulated dendrimer G_3-6-6-6-N.

8. References:

(1) Curinova, P.; Krupkova, A.; Stastna, L. C.; Mullerova, M.; Cermak, J.; Strasak, T.; Cuřínová, P.; Krupková, A.; Červenková Šťastná, L.; Müllerová, M.; Čermák, J.; Strašák, T. ESI-TOF Mass Spectrometry of Cationic Carbosilane Dendrimers: A Potent Tool for Characterization of Structural Defects. *J. Mass Spectrom.* **2018**, *53* (10), 986–996. DOI: 10.1002/jms.4269.

(2) Sharma, A.; Beirne, J.; Khamar, D.; Maguire, C.; Hayden, A.; Hughes, H. Evaluation and Screening of Biopharmaceuticals Using Multi-Angle Dynamic Light Scattering. *AAPS PharmSciTech* **2023**, *24* (4), 84. DOI: 10.1208/s12249-023-02529-4.

(3) Tanner, J. E. Use of the Stimulated Echo in Nmr Diffusion Studies. *J. Chem. Phys.* **1970**, *52* (5), 2523–2526. DOI: 10.1063/1.1673336.

(4) Gibbs, S. J.; Johnson, C. S. A PFG NMR Experiment for Accurate Diffusion and Flow Studies in the Presence of Eddy Currents. *J. Magn. Reson.* **1991**, *93* (2), 395–402. DOI: 10.1016/0022-2364(91)90014-K.

(5) Jakalian, A.; Bush, B. L.; Jack, D. B.; Bayly, C. I. Fast, Efficient Generation of High-Quality Atomic Charges. AM1-BCC Model: I. Method. *J. Comput. Chem.* **2000**, *21* (2), 132–146. DOI: 10.1002/(SICI)1096-987X(20000130)21:2<132::AID-JCC5>3.0.CO;2-P.

(6) Wang, J.; Wolf, R. M.; Caldwell, J. W.; Kollman, P. A.; Case, D. A. Development and Testing of a General Amber Force Field. *J. Comput. Chem.* **2004**, *25* (9), 1157–1174. DOI: 10.1002/jcc.20035.

(7) Gordon, M. S.; Schmidt, M. W. Advances in Electronic Structure Theory: GAMESS a Decade Later. *Theory and Applications of Computational Chemistry*; Dykstra, C. E., Frenking, G., Kim, K. S., Scuseria, G. E., Eds.; Elsevier Science, 2005; pp 1167–1189. DOI: 10.1016/B978-044451719-7/50084-6.

(8) Case, D. A.; Belfon, K.; Ben-Shalom, I. Y., Brozell, S. R.; Cerutti, D. S.; Cheatham, T. E., III, Cruzeiro, V. W. D.; Darden, T. A.; Duke, R. E.; Giambasu, G.; Gilson, M. K.; Gohlke, H.; Goetz, A. W.; Harris, R.; Izadi, S.; Izmailov, S. A.; Kasavajhala, K.; Kovalenko, A.; Krasny, R.; Kurtzman, T.; Lee, T. S.; LeGrand, S.; Li, P.; Lin, C.; Liu, J.; Luchko, T.; Luo, R.; Man, V.; Merz, K. M.; Miao, Y.; Mikhailovskii, O.; Monard, G.; Nguyen, H.; Onufriev, A.; Pan, F.; Pantano, S.; Qi, R.; Roe, D. R.; Roitberg, A.; Sagui, C.; Schott-Verdugo, S., Shen, J.; Simmerling, C. L.; Skrynnikov, N. R.; Smith, J.; Swails, J.; Walker, R. C.; Wang, J.; Wilson, L.; Wolf, R. M.; Wu, X.; Xiong, Y.; Xue, Y.; York, D. M.; Kollman, P. A., *AMBER 2020*; University of California: San Francisco, 2020.(10) Lii, J.-H.; Allinger, N. L. The MM3 Force Field for Amides, Polypeptides and Proteins. *J. Comput. Chem.* **1991**, *12* (2), 186–199. https://doi.org/10.1002/jcc.540120208.

(9) Lii, J. H.; Allinger, N. L. The MM3 Force Field for Amides, Polypeptides and Proteins. *J. Comput. Chem.* **1991**, *12* (2), 186–199. DOI: 10.1002/jcc.540120208.

(10) Edr, A.; Wrobel, D.; Krupková, A.; Červenková Šťastná, L.; Cuřínová, P.; Novák, A.; Malý, J.; Kalasová, J.; Malý, J.; Malý, M.; et al. Adaptive Synthesis of Functional Amphiphilic Dendrons as a Novel Approach to Artificial Supramolecular Objects. *Int. J. Mol. Sci.* **2022**, *23* (4), 2114. DOI: 10.3390/ijms23042114.

(11) Ryckaert, J. P.; Ciccotti, G.; Berendsen, H. J. C. Numerical Integration of the Cartesian Equations of Motion of a System with Constraints: Molecular Dynamics of n-Alkanes. *J. Comput. Phys.* **1977**, *23* (3), 327–341. DOI: 10.1016/0021-9991(77)90098-5.

(12) Wu, X.; Brooks, B. R.; Vanden-Eijnden, E. Self-Guided Langevin Dynamics via Generalized Langevin Equation. *J. Comput. Chem.* **2016**, *37* (6), 595–601. DOI: 10.1002/jcc.24015.

(13) Götz, A. W.; Williamson, M. J.; Xu, D.; Poole, D.; Le Grand, S.; Walker, R. C. Routine Microsecond

Molecular Dynamics Simulations with AMBER on GPUs. 1. Generalized Born. *J. Chem. Theory Comput.* **2012**, *8* (5), 1542–1555. DOI: 10.1021/ct200909j.

(14) Pettersen, E. F.; Goddard, T. D.; Huang, C. C.; Couch, G. S.; Greenblatt, D. M.; Meng, E. C.; Ferrin, T. E. UCSF Chimera - A Visualization System for Exploratory Research and Analysis. *J. Comput. Chem.* **2004**, *25* (13), 1605–1612. https://doi.org/10.1002/jcc.20084.

9. NMR spektra

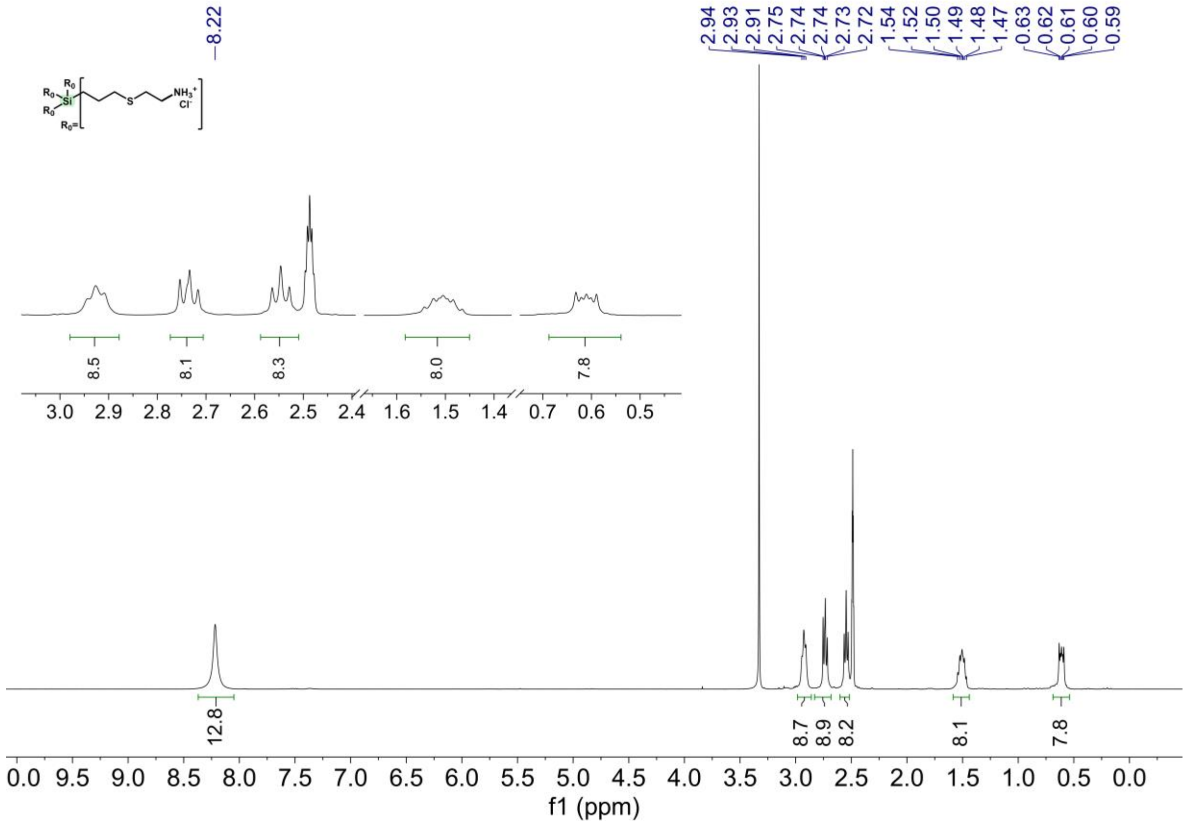

Figure S63: ^{1}H NMR (400 MHz, DMSO-d_6) **G_0-N**

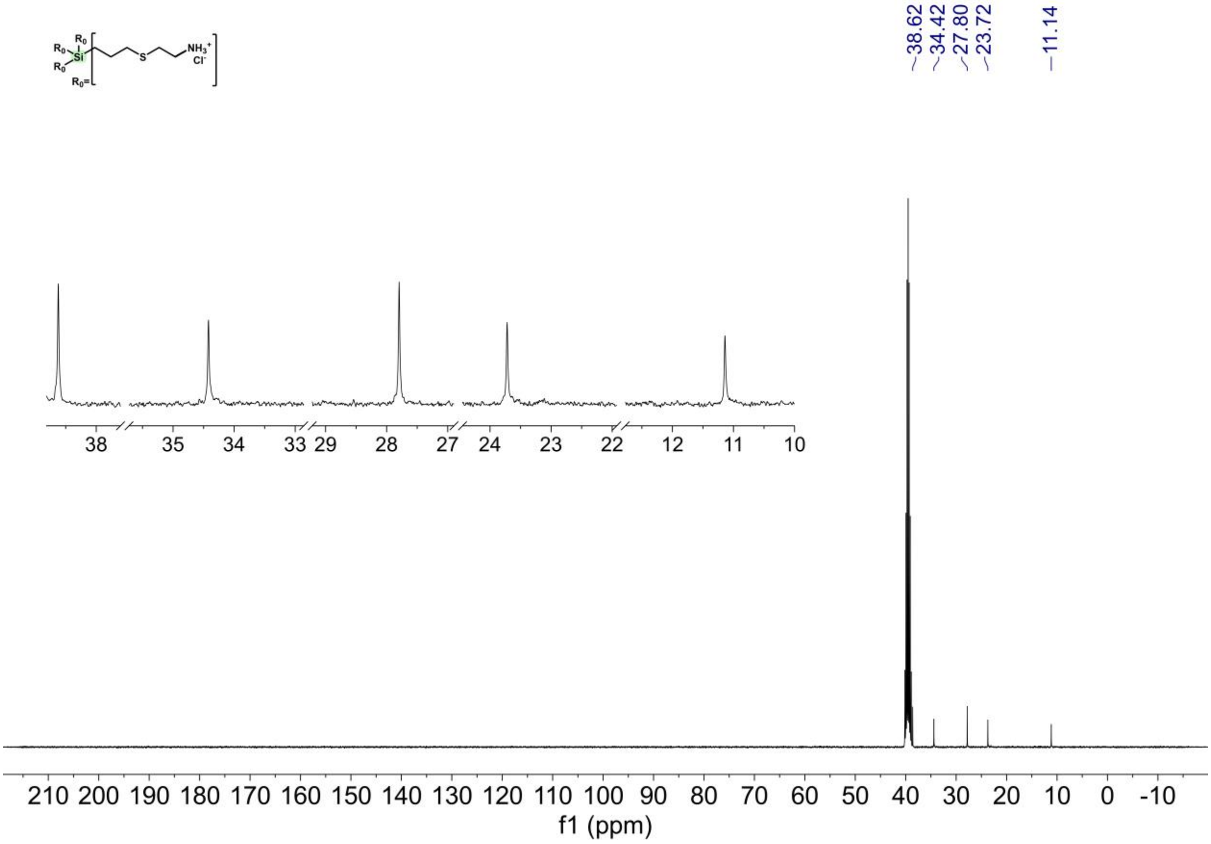

Figure S64: ^{13}C {^{1}H} NMR (101 MHz, DMSO-d_6) **G$_0$-N**

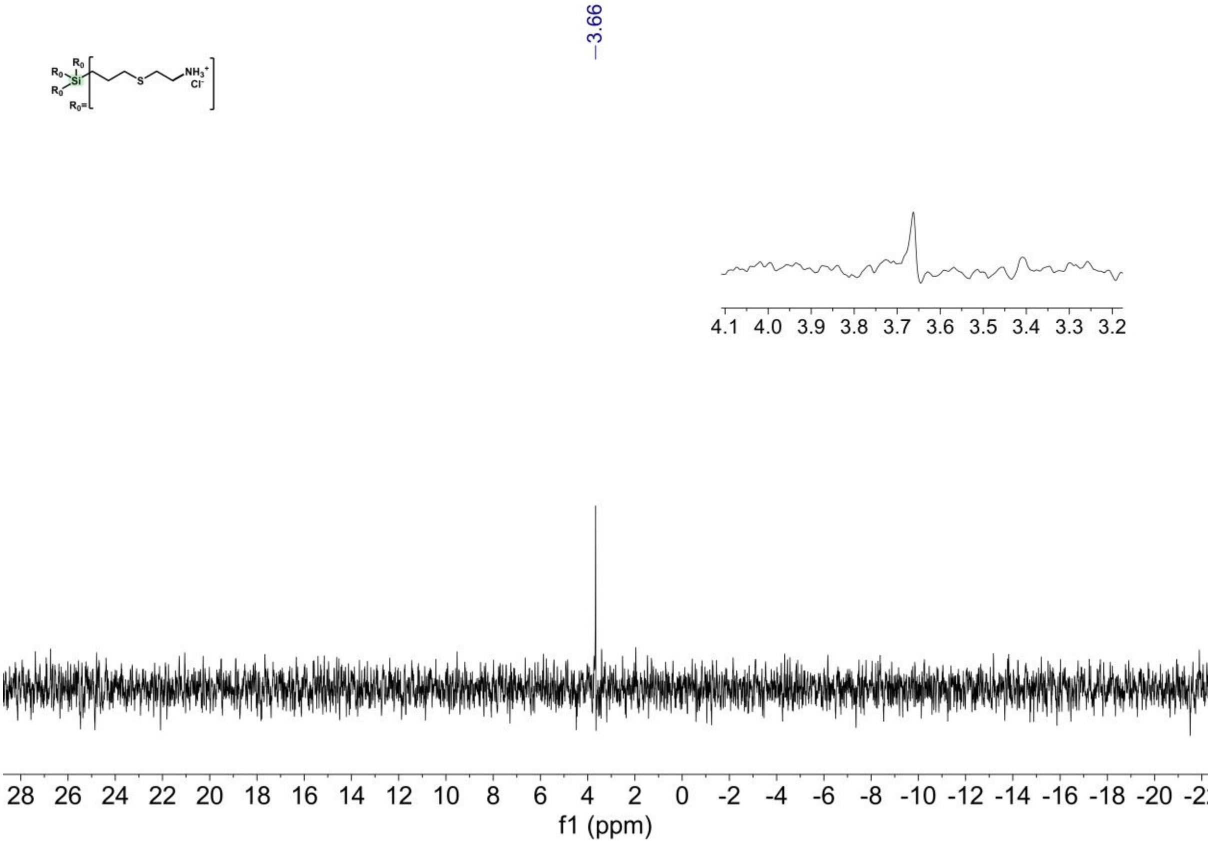

Figure S65: ^{29}C {^{1}H} NMR (79 MHz, DMSO-d_6) **G$_0$-N**

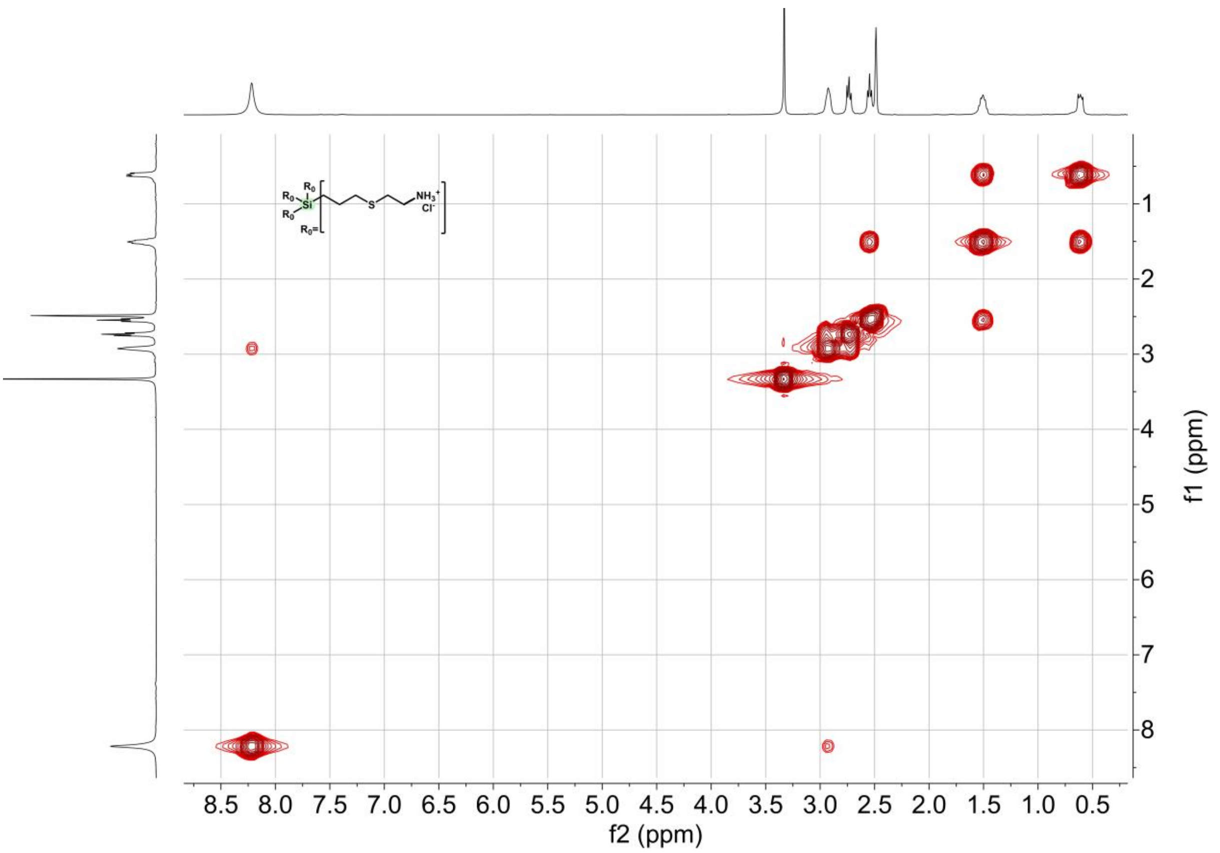

Figure S66: ^{1}H-^{1}H COSY NMR (DMSO-d_6) **G_0-N**

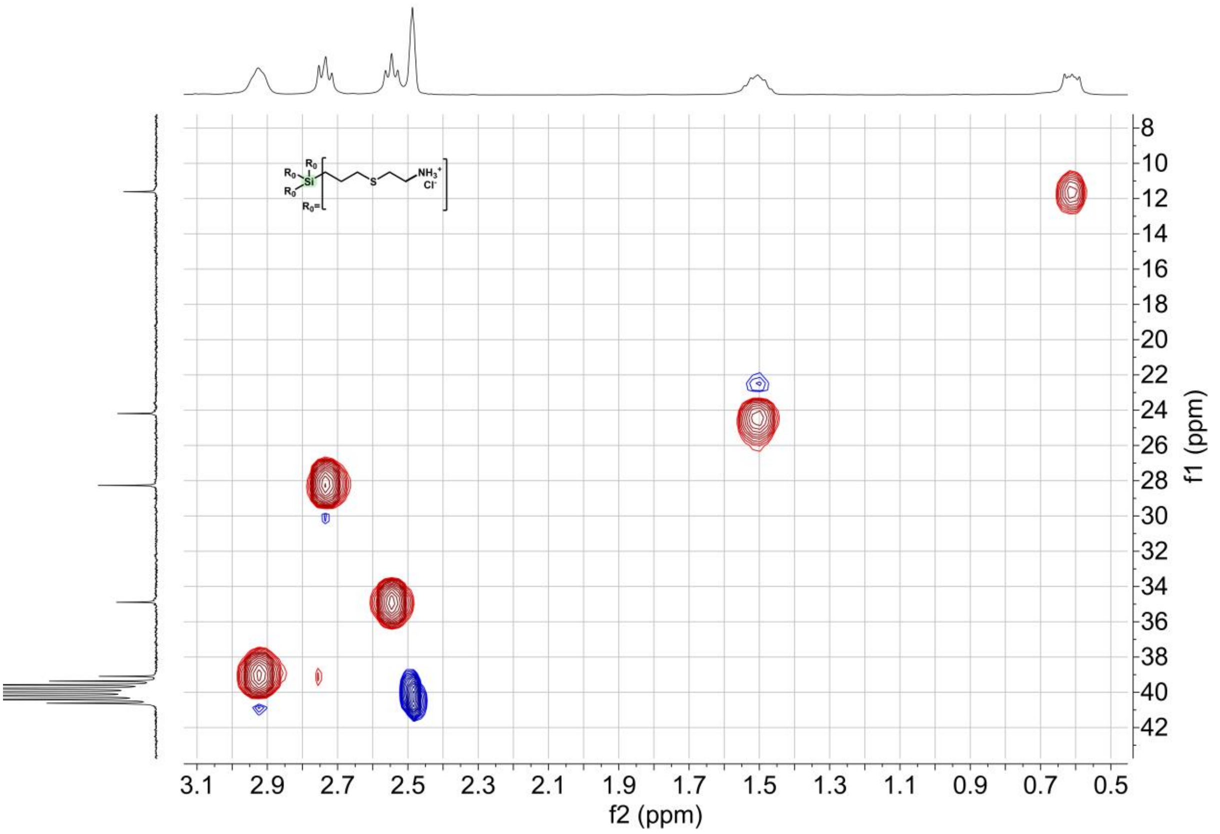

Figure S67: ^{1}H-^{13}C HSQC NMR (DMSO-d_6) **G_0-N**

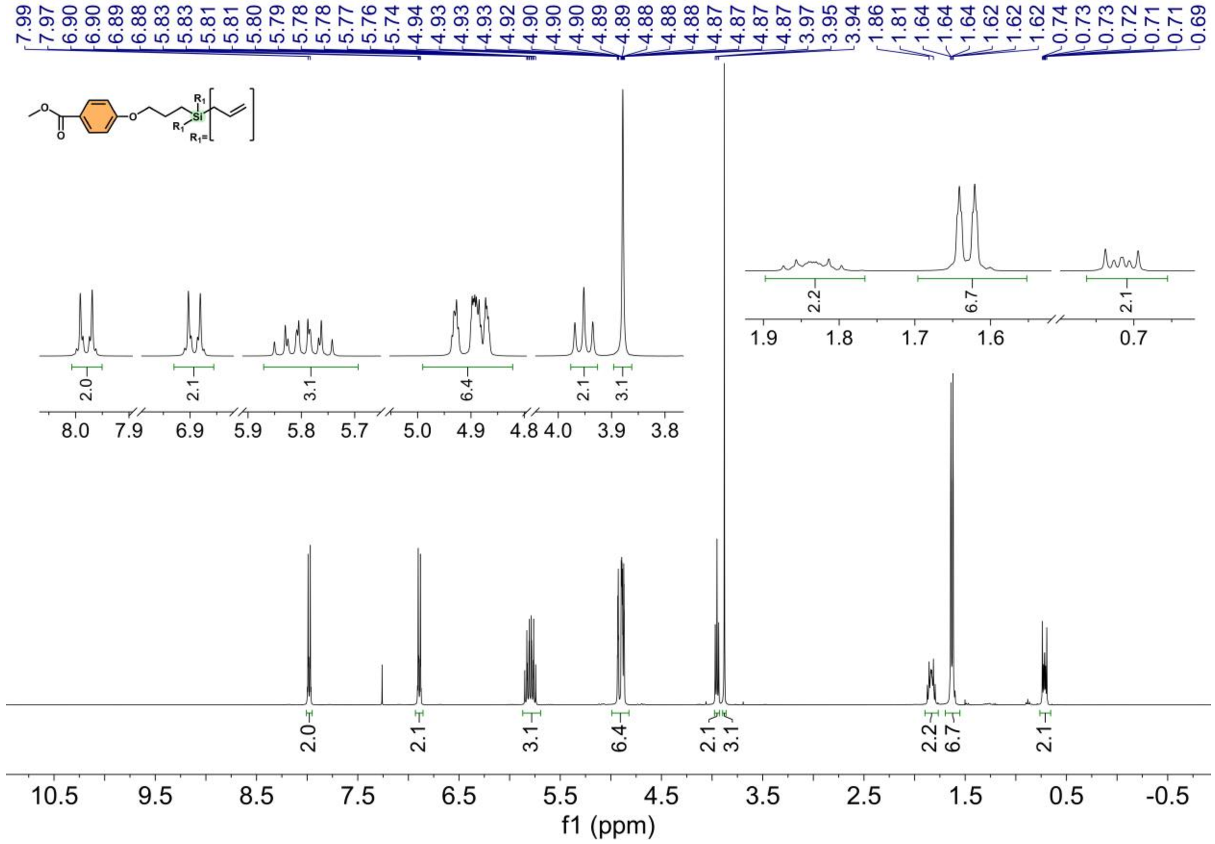

Figure S68: ^{1}H NMR (400 MHz, $CDCl_3$) **MeO-AB$_3$**

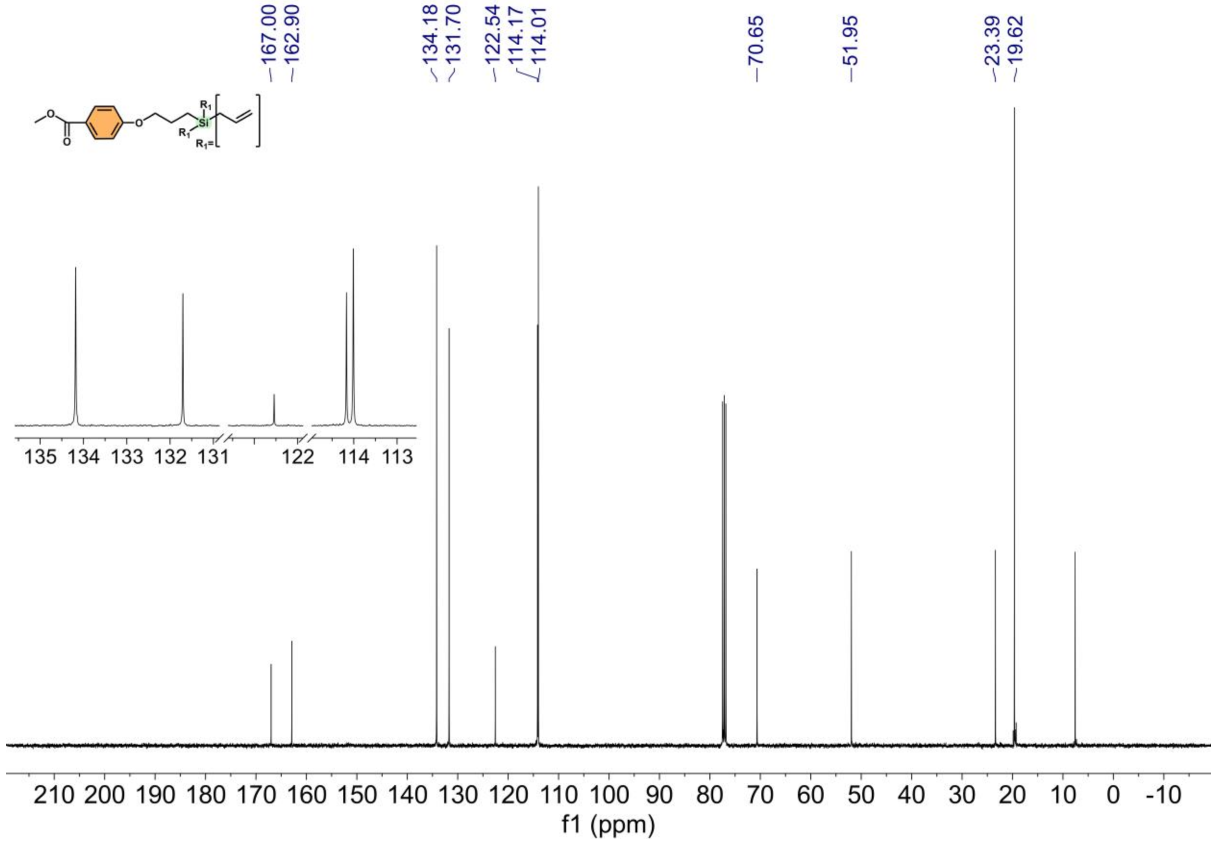

Figure S69: ^{13}C {^{1}H} NMR (101 MHz, $CDCl_3$) **MeO-AB$_3$**

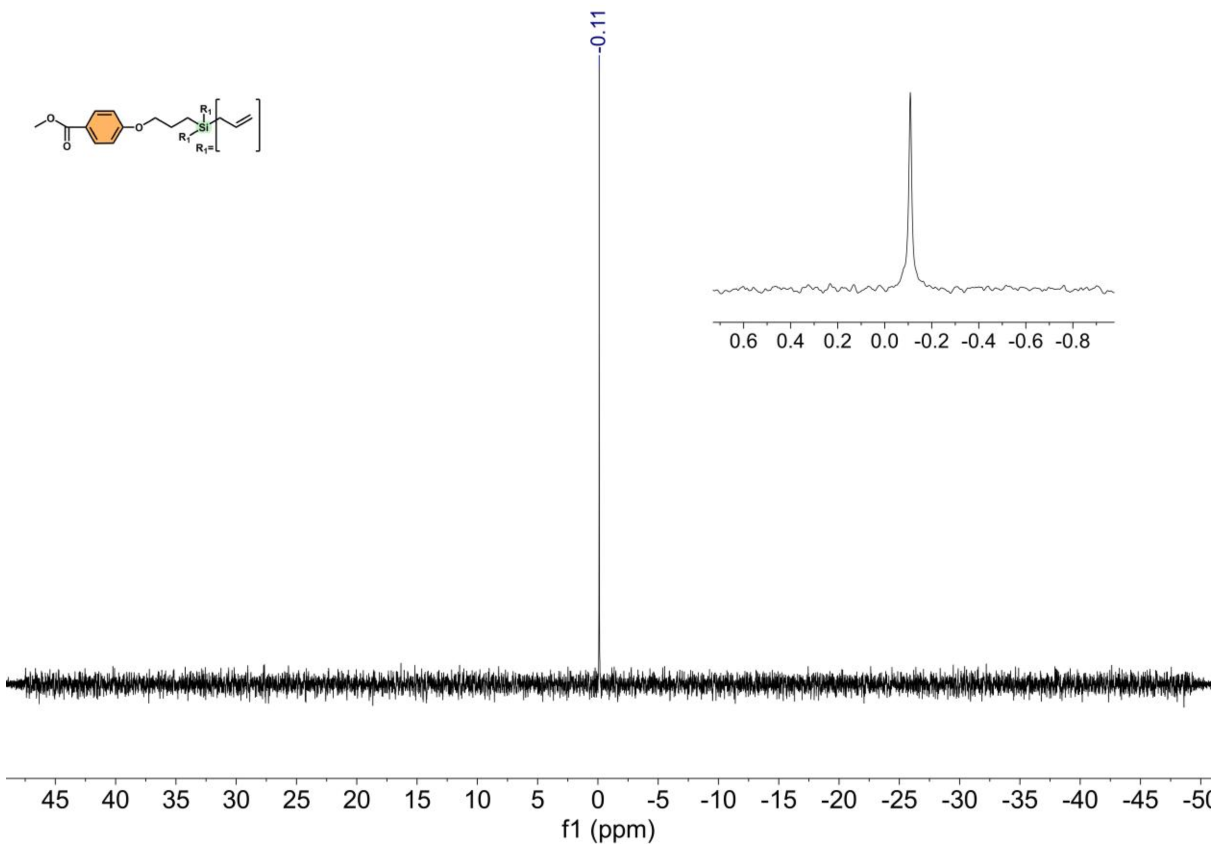

Figure S70: ^{29}Si {^{1}H} NMR (79 MHz, $CDCl_3$) **MeO-AB$_3$**

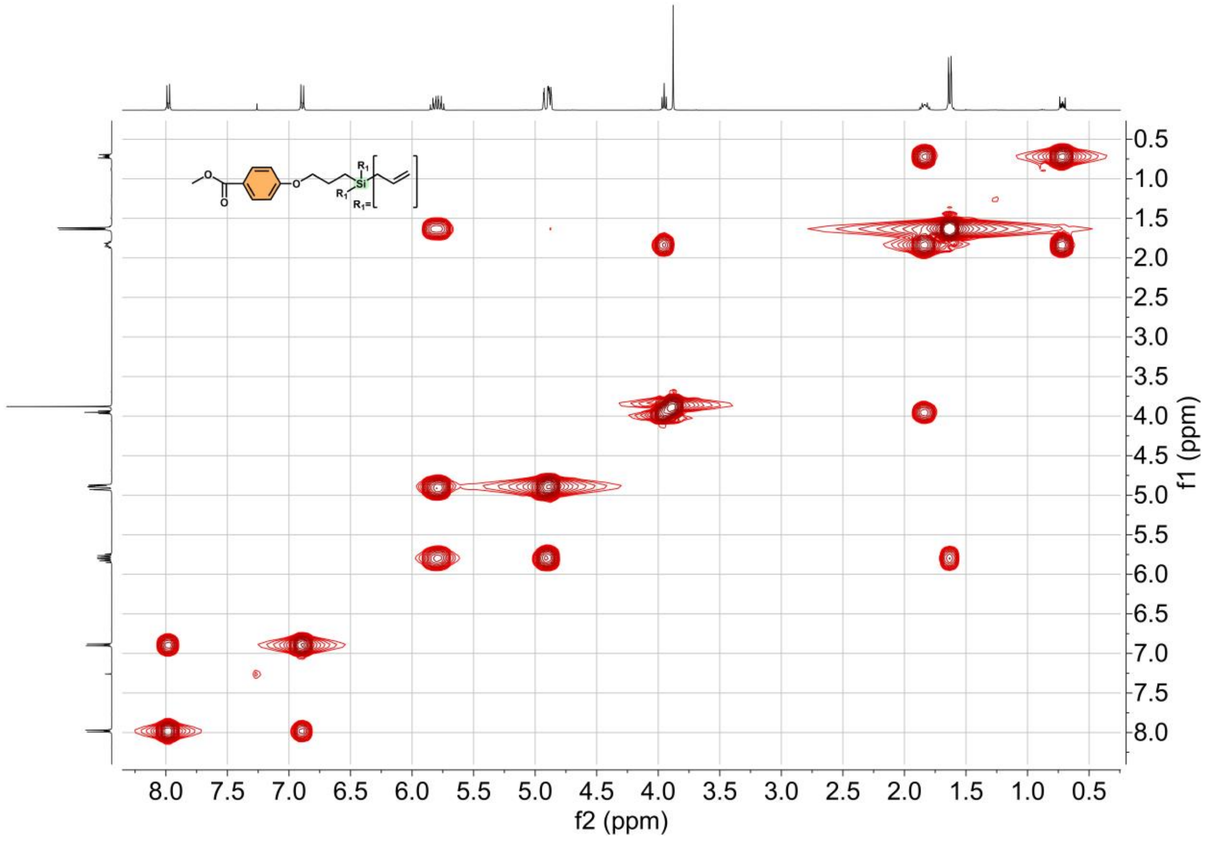

Figure S71: ^{1}H-^{1}H COSY NMR ($CDCl_3$) **MeO-AB$_3$**

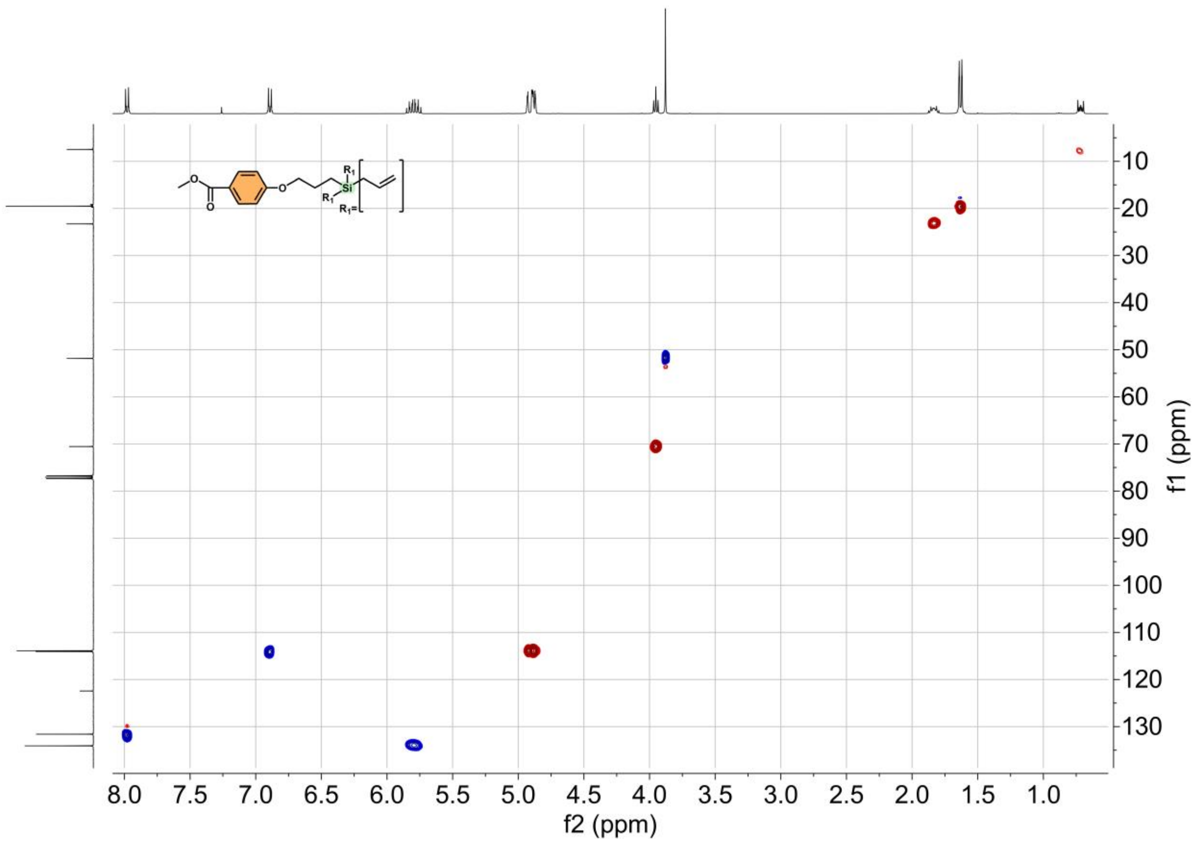

Figure S72: ^{1}H-^{13}C HSQC NMR ($CDCl_3$) **MeO-AB$_3$**

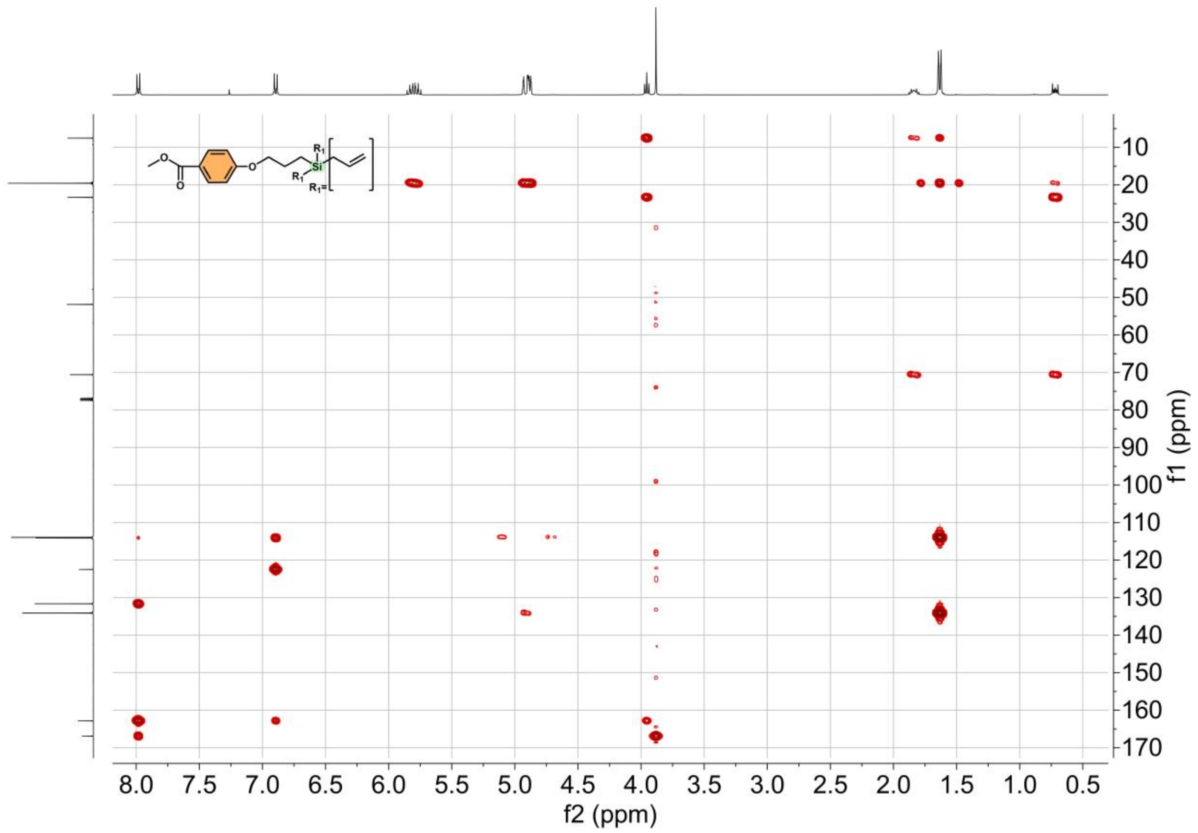

Figure S73: ^{1}H-^{13}C HMBC NMR ($CDCl_3$) **MeO-AB$_3$**

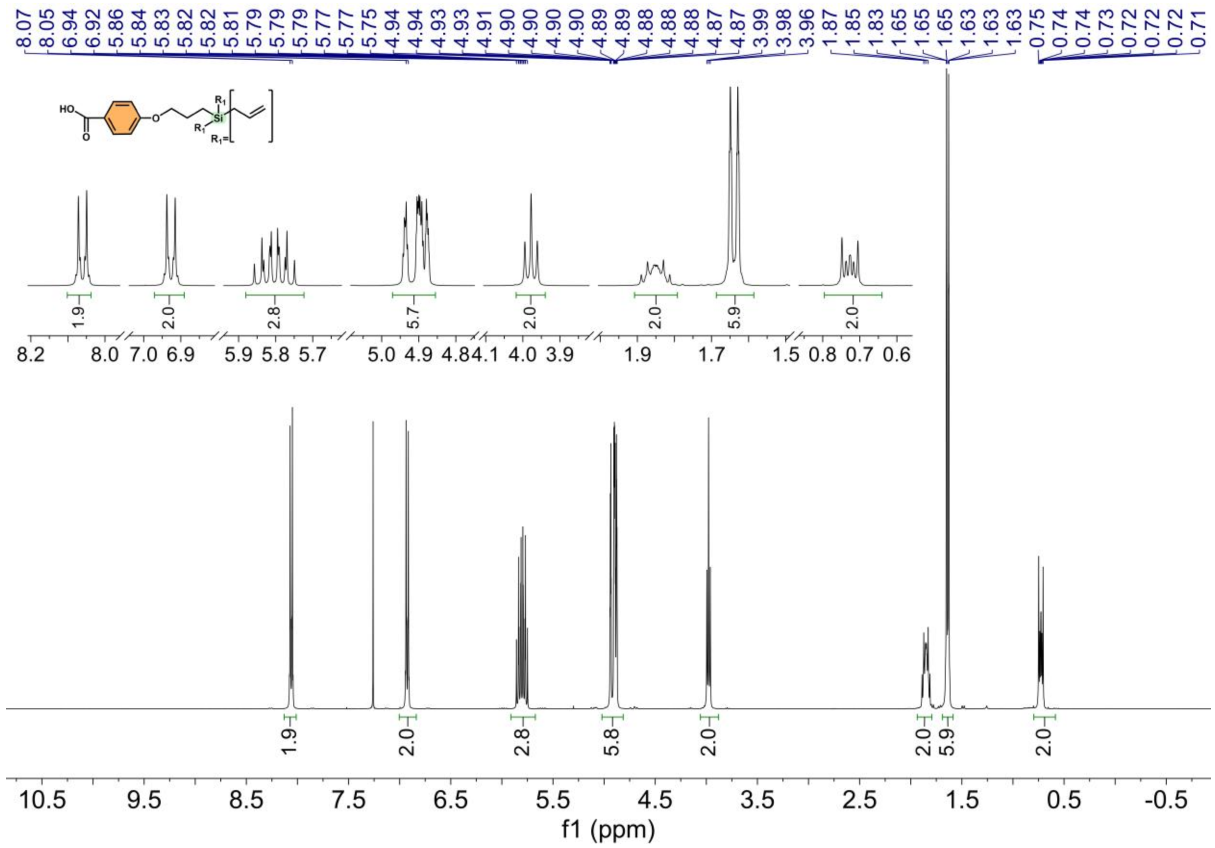

Figure S74: ^{1}H NMR (400 MHz, $CDCl_3$) **AB$_3$**

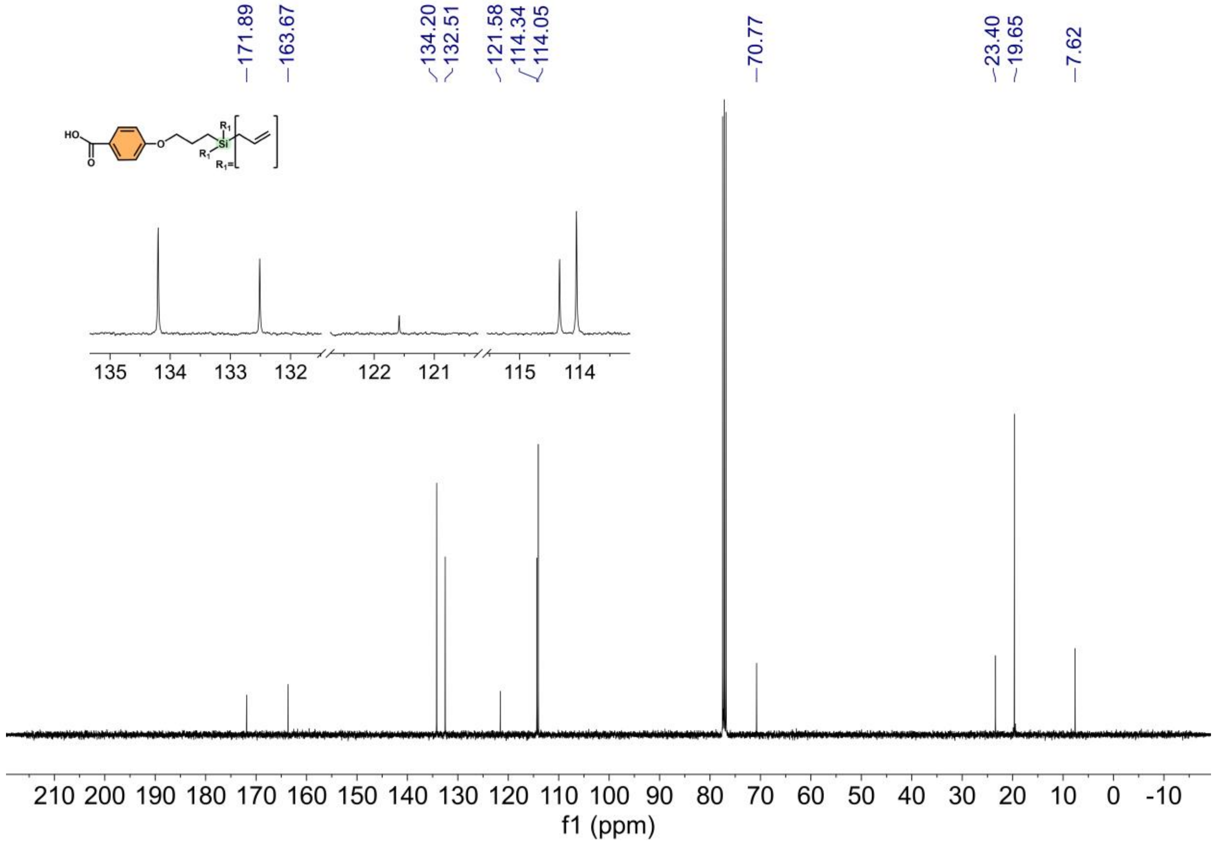

Figure S75: ^{13}C {^{1}H} NMR (101 MHz, $CDCl_3$) **AB$_3$**

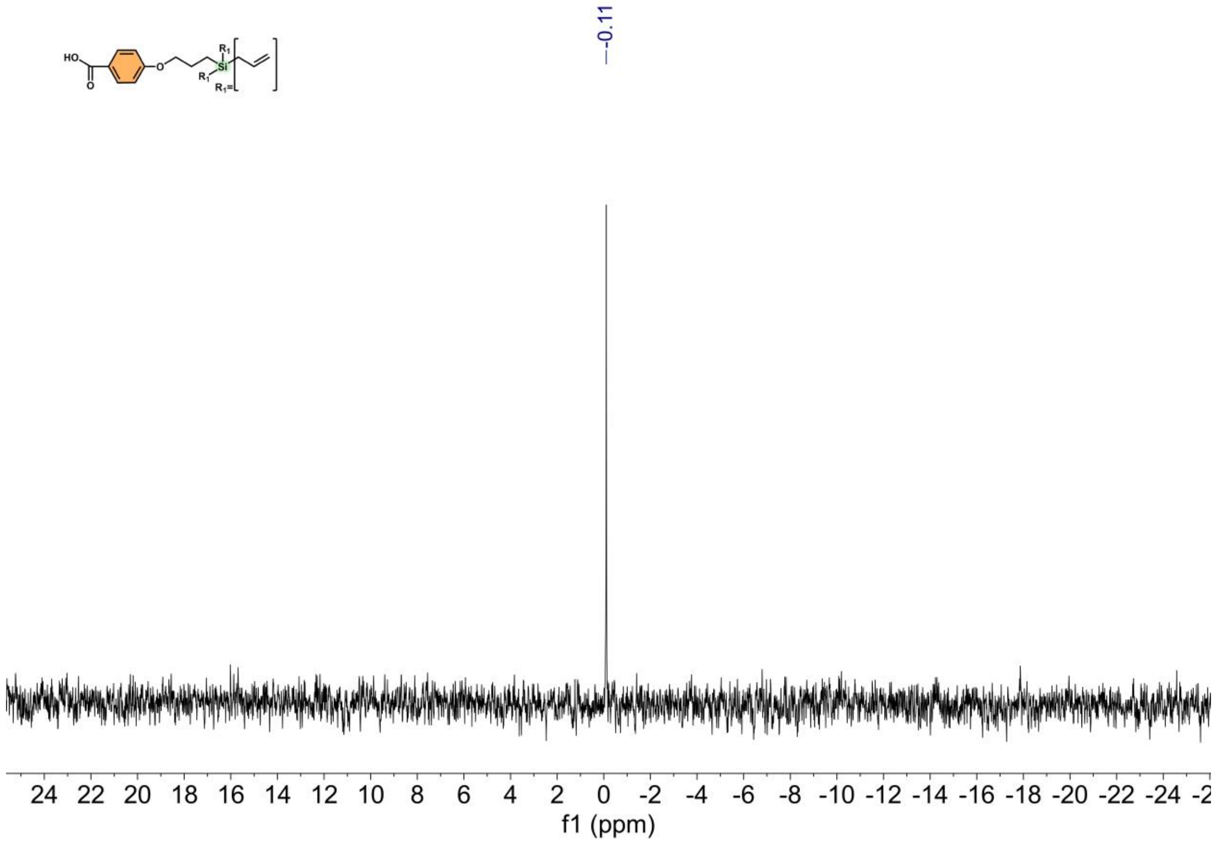

Figure S76: ^{29}Si {^{1}H} NMR (79 MHz, $CDCl_3$) **AB$_3$**

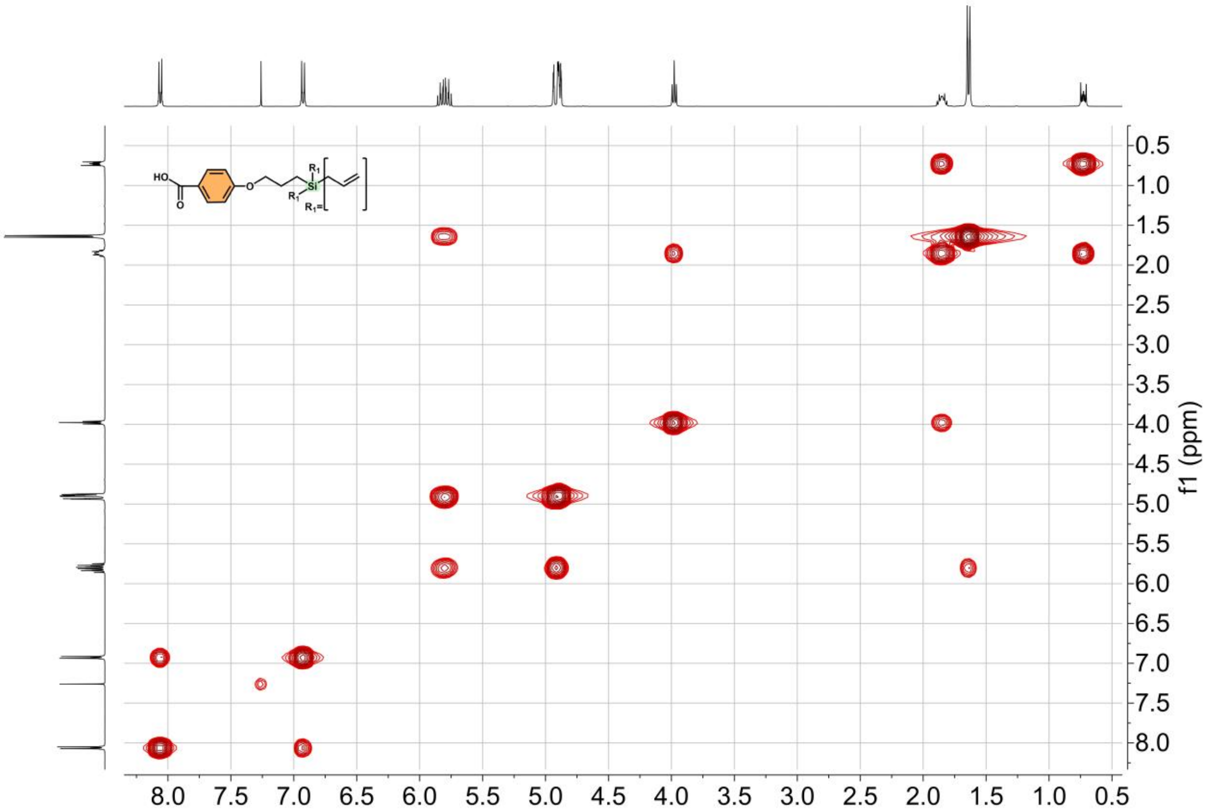

Figure S77: ^{1}H-^{1}H COSY NMR ($CDCl_3$) **AB$_3$**

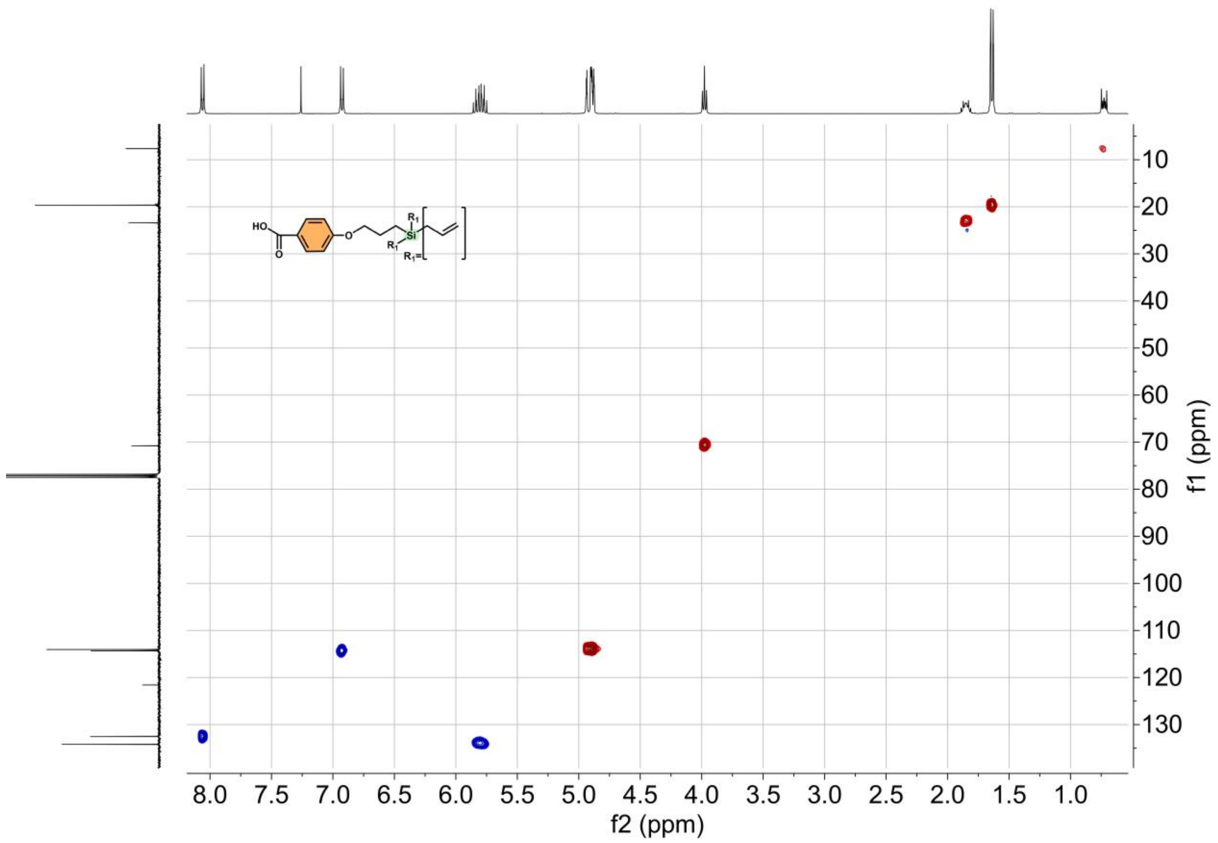

Figure S78: ^{1}H-^{13}C HSQC NMR ($CDCl_3$) **AB_3**

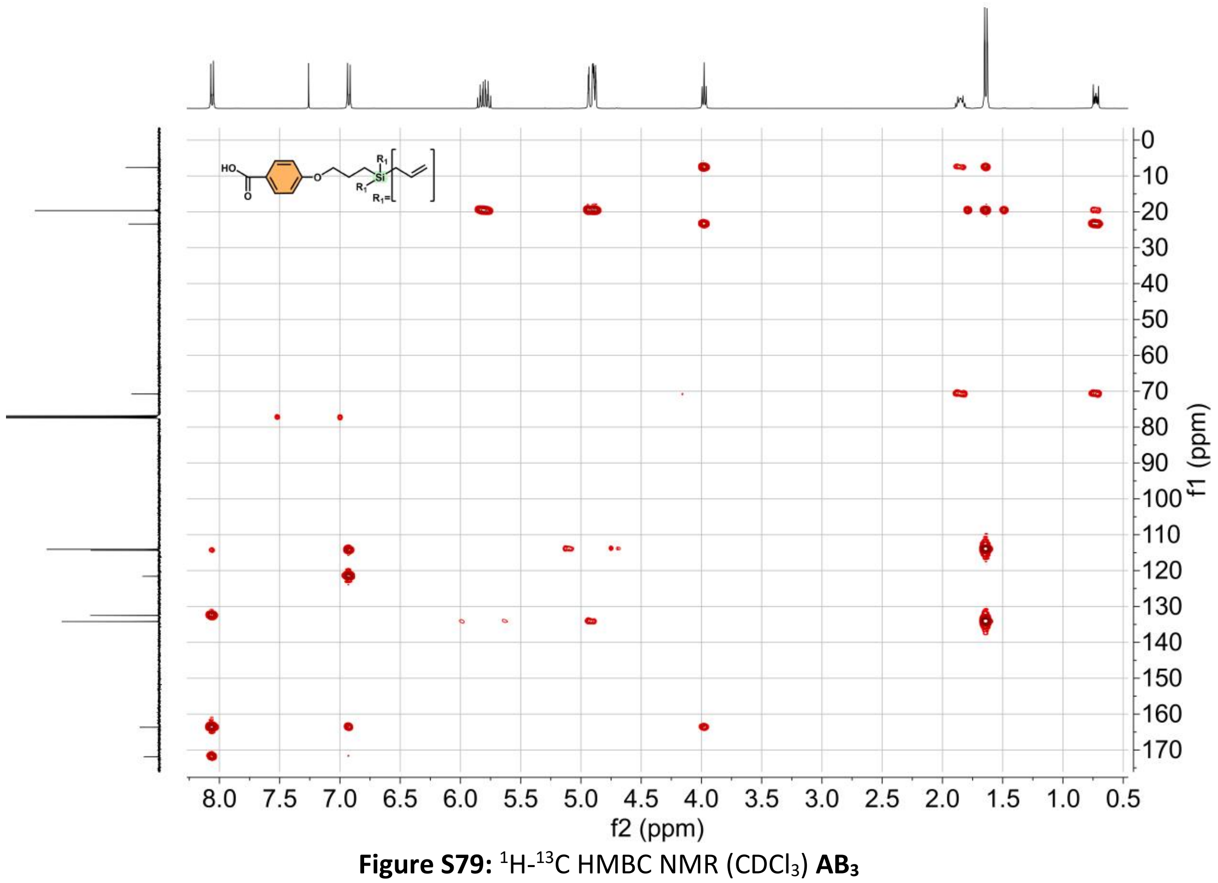

Figure S79: ^{1}H-^{13}C HMBC NMR ($CDCl_3$) **AB_3**

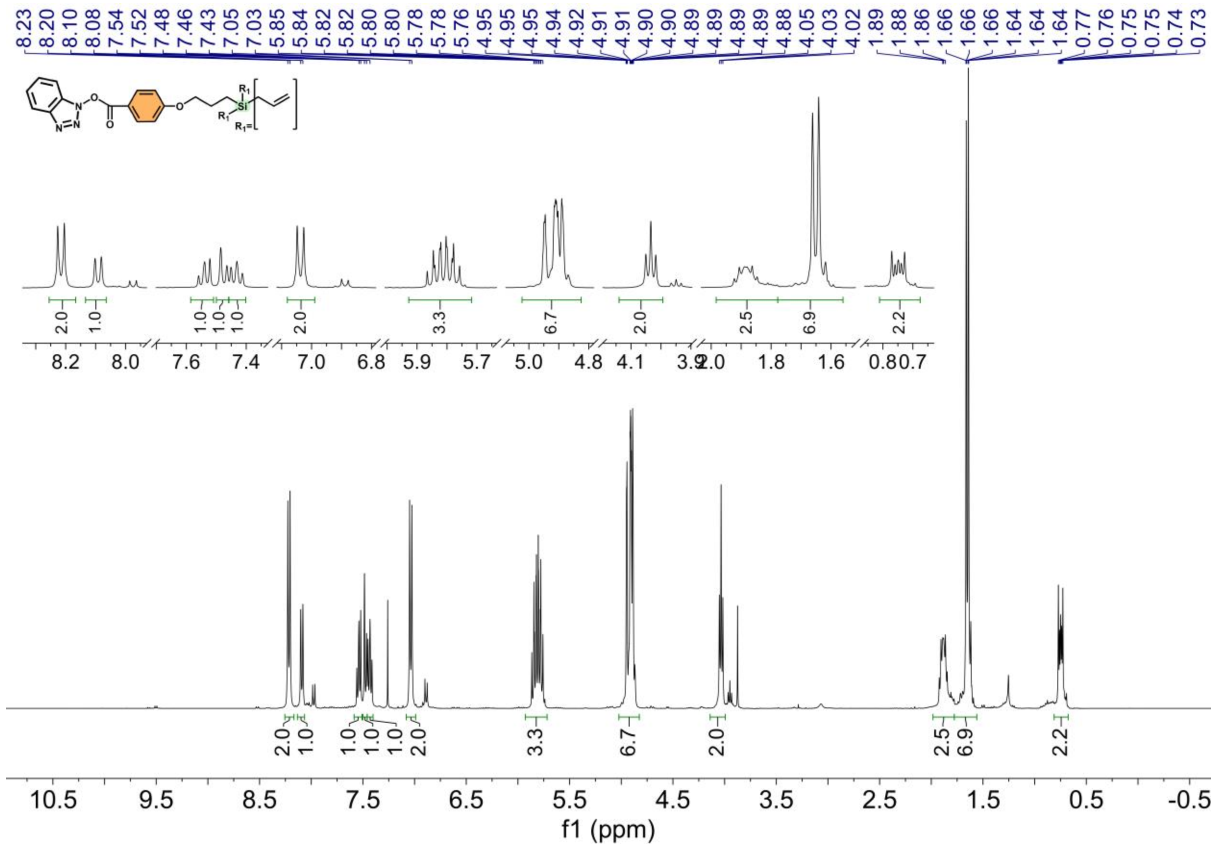

Figure S80: ^{1}H NMR (400 MHz, $CDCl_3$) **BtO-AB$_3$**

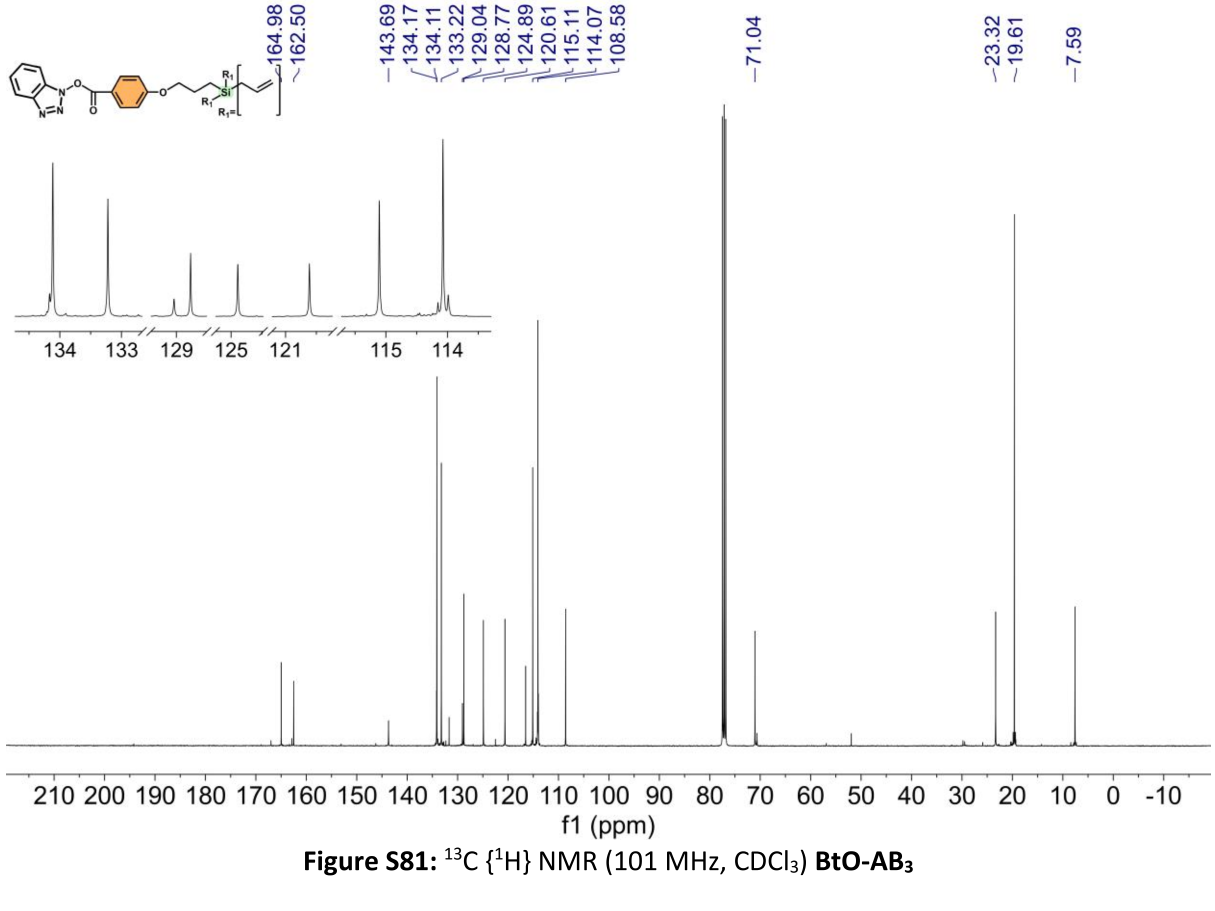

Figure S81: ^{13}C {^{1}H} NMR (101 MHz, $CDCl_3$) **BtO-AB$_3$**

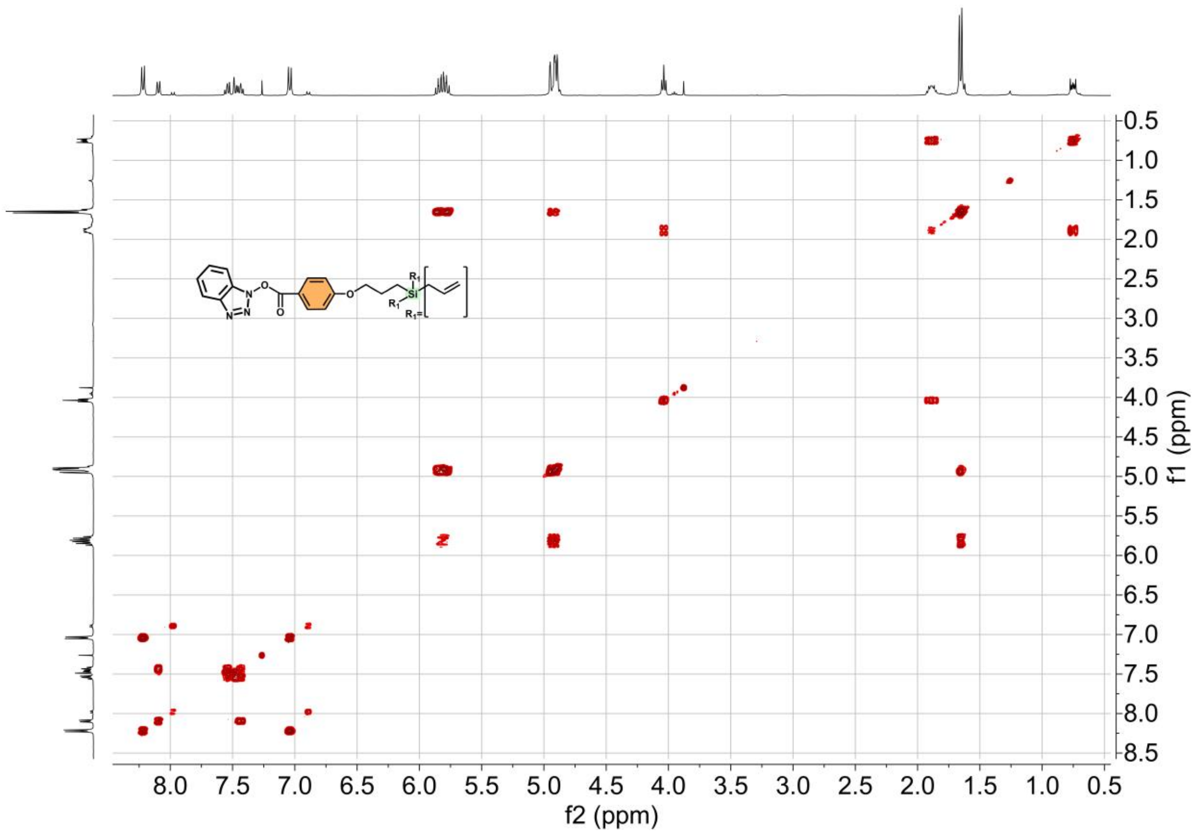

Figure S82: ^{1}H-^{1}H COSY NMR ($CDCl_3$) **BtO-AB$_3$**

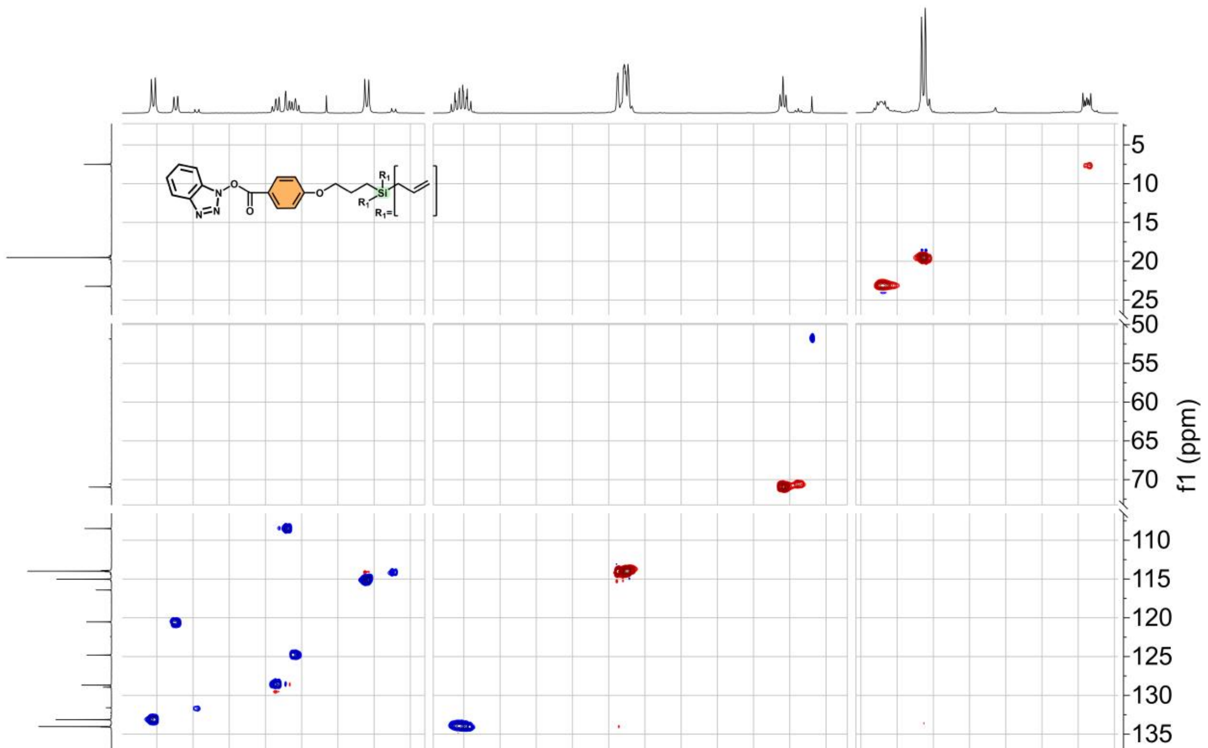

Figure S83: ^{1}H-^{13}C HSQC NMR ($CDCl_3$) **BtO-AB$_3$**

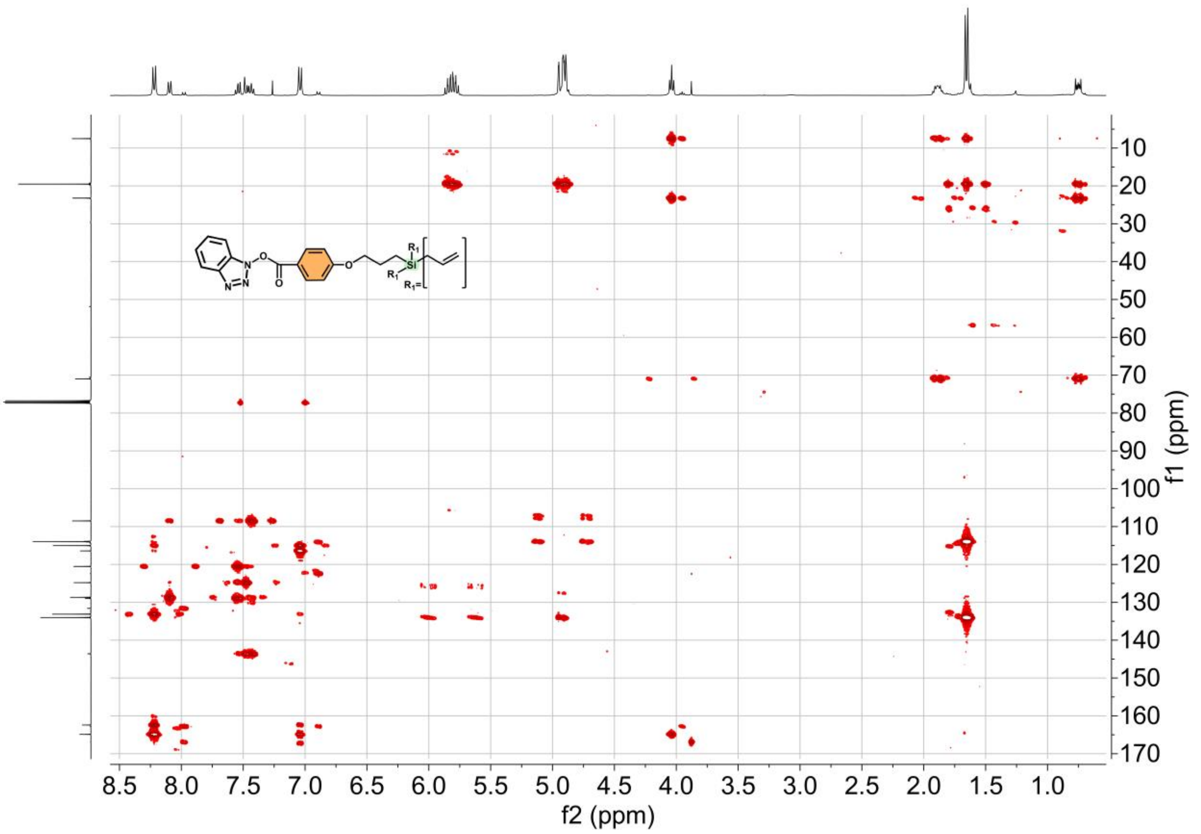

Figure S84: ^{1}H-^{13}C HMBC NMR ($CDCl_3$) **BtO-AB$_3$**

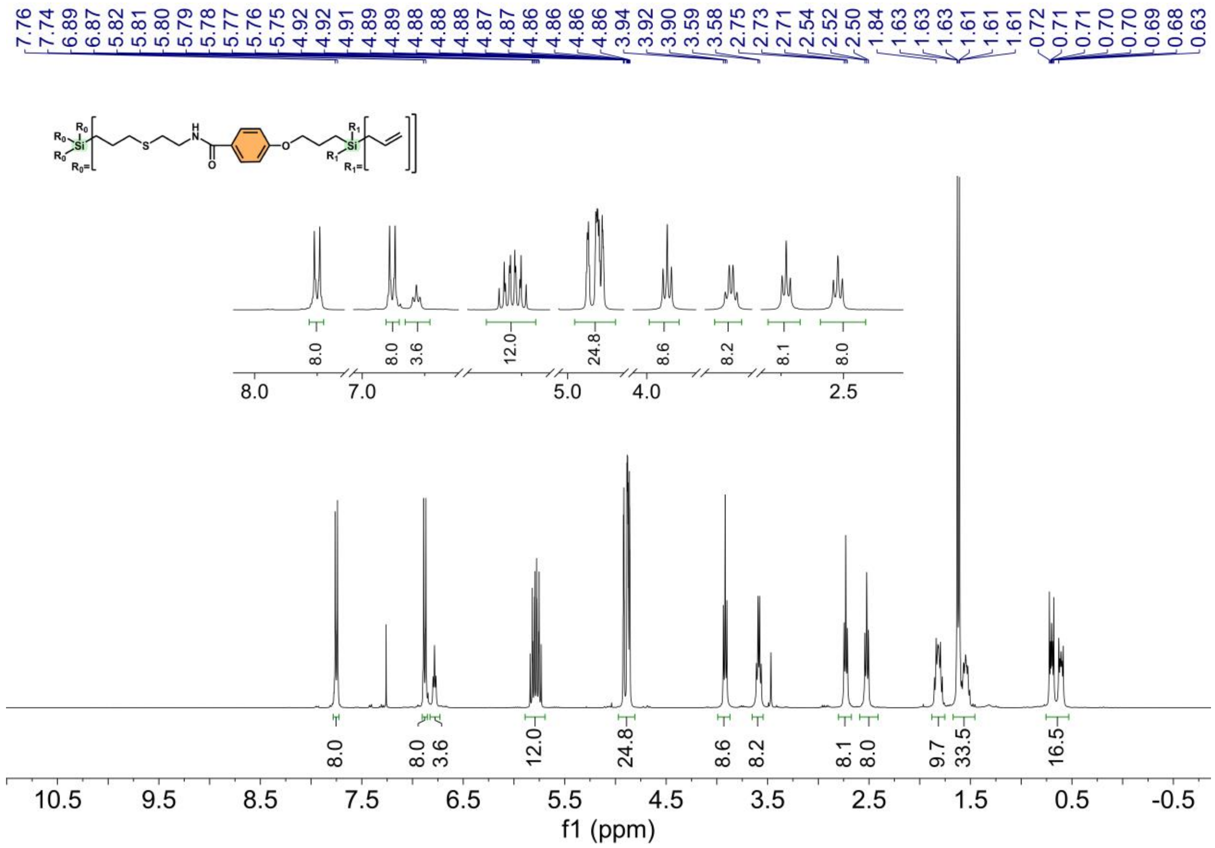

Figure S85: ^{1}H NMR (400 MHz, $CDCl_3$) **G$_1$-3-A**

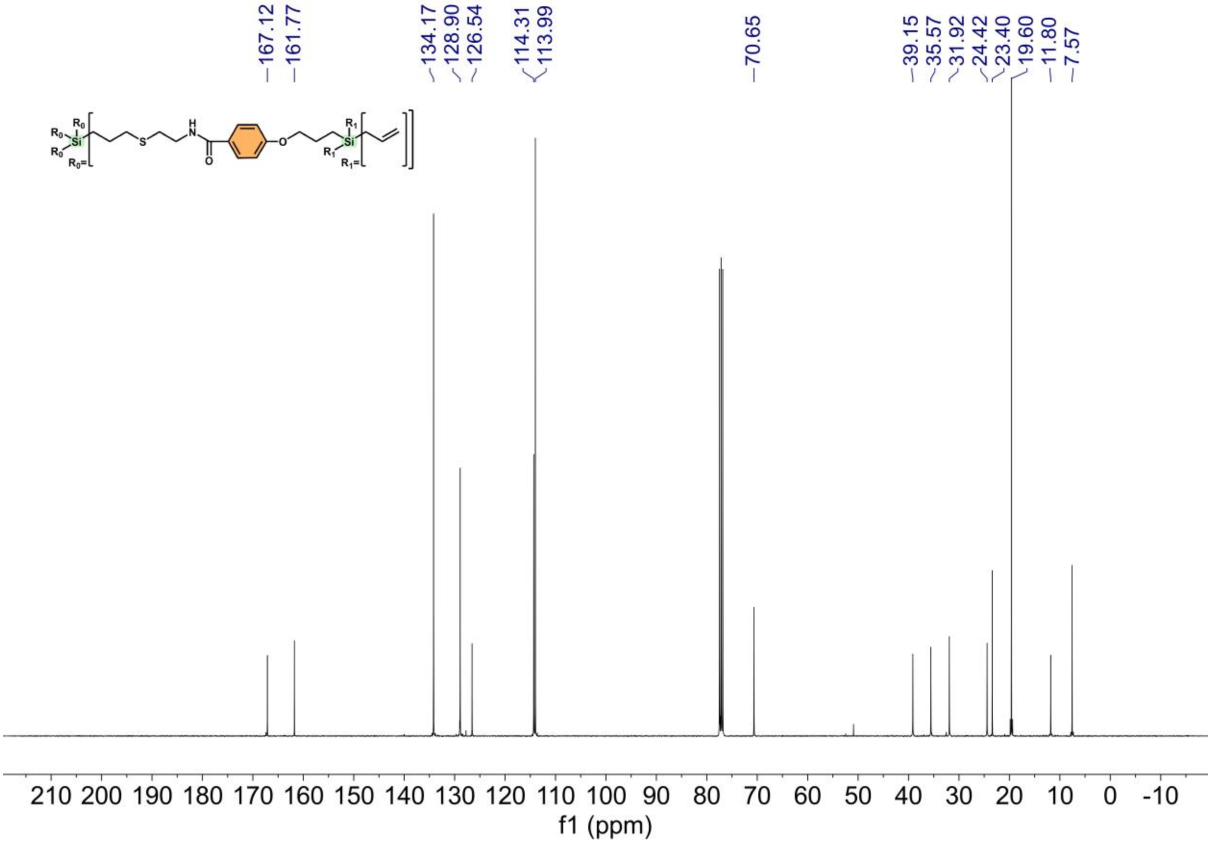

Figure S86: $^{13}C\ \{^{1}H\}$ NMR (101 MHz, $CDCl_3$) **G_1-3-A**

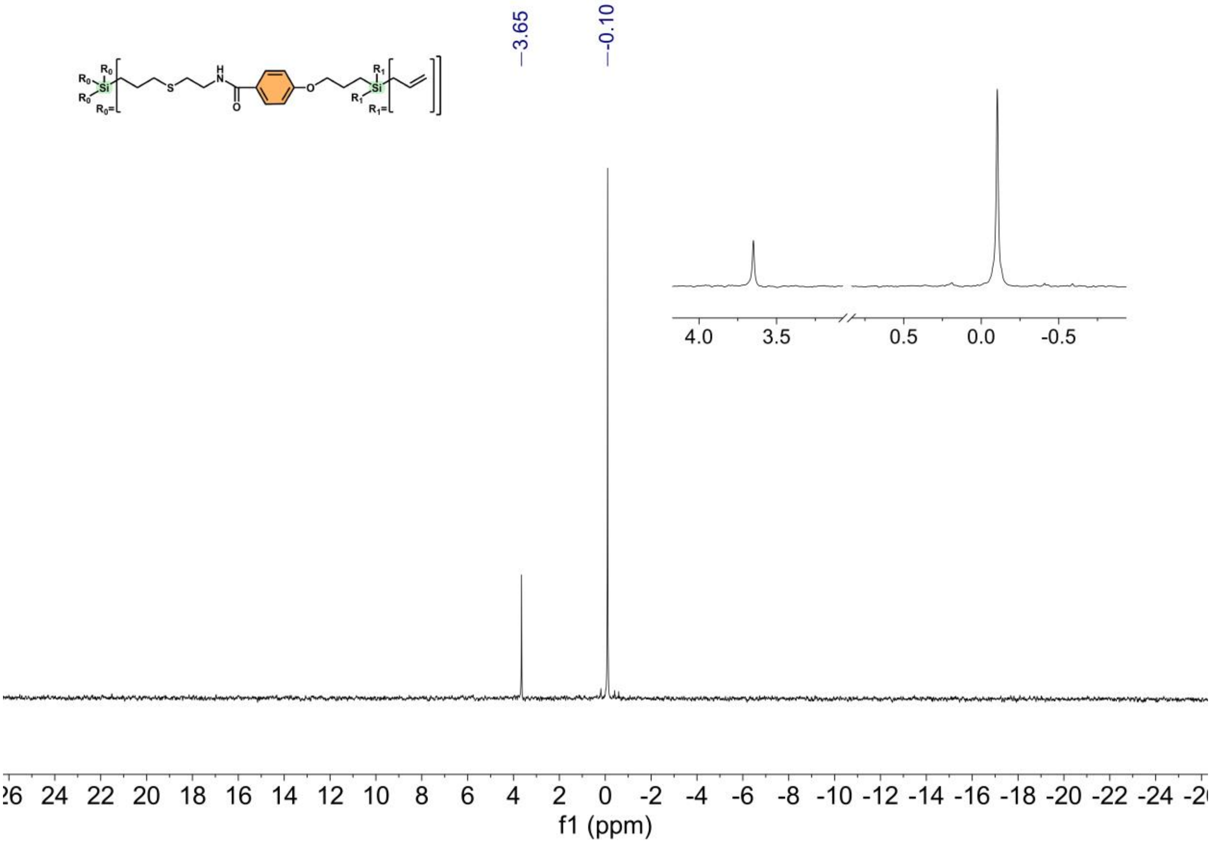

Figure S87: $^{29}Si\ \{^{1}H\}$ NMR (79 MHz, $CDCl_3$) **G_1-3-A**

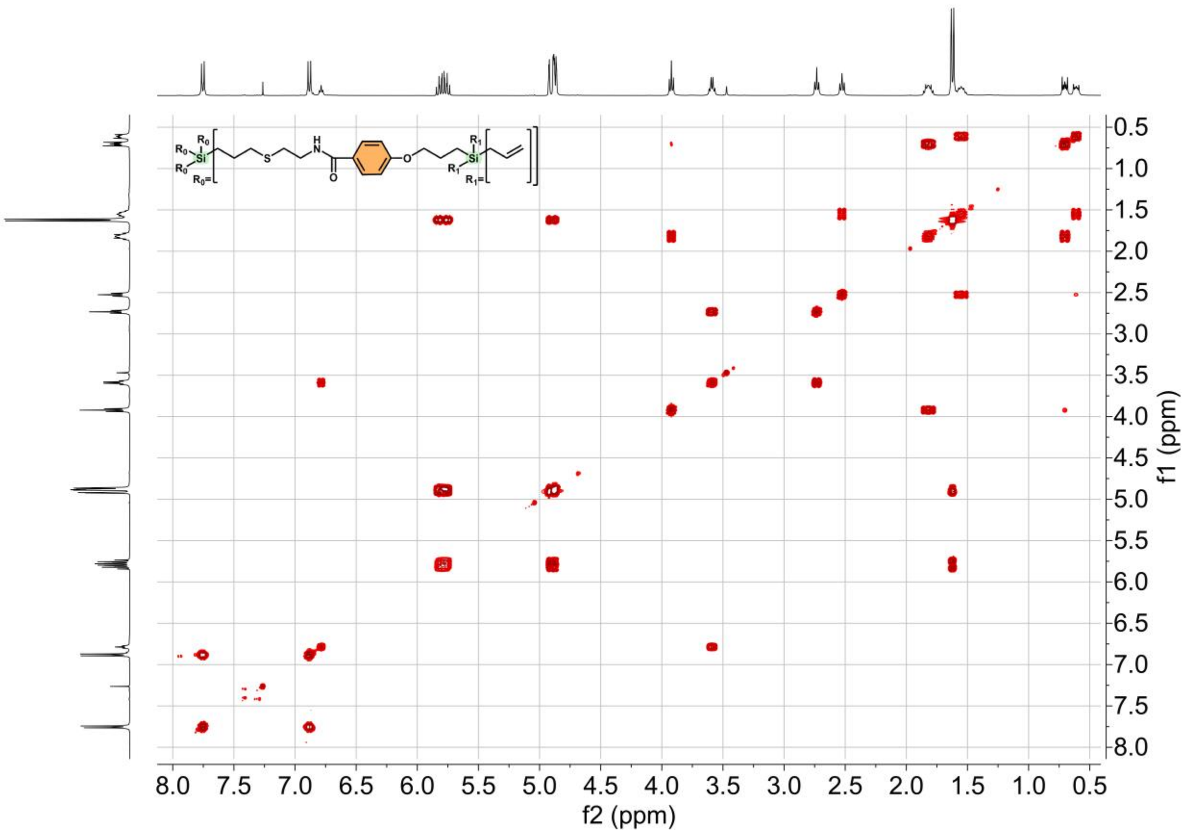

Figure S88: ^{1}H-^{1}H COSY NMR ($CDCl_3$) **G_1-3-A**

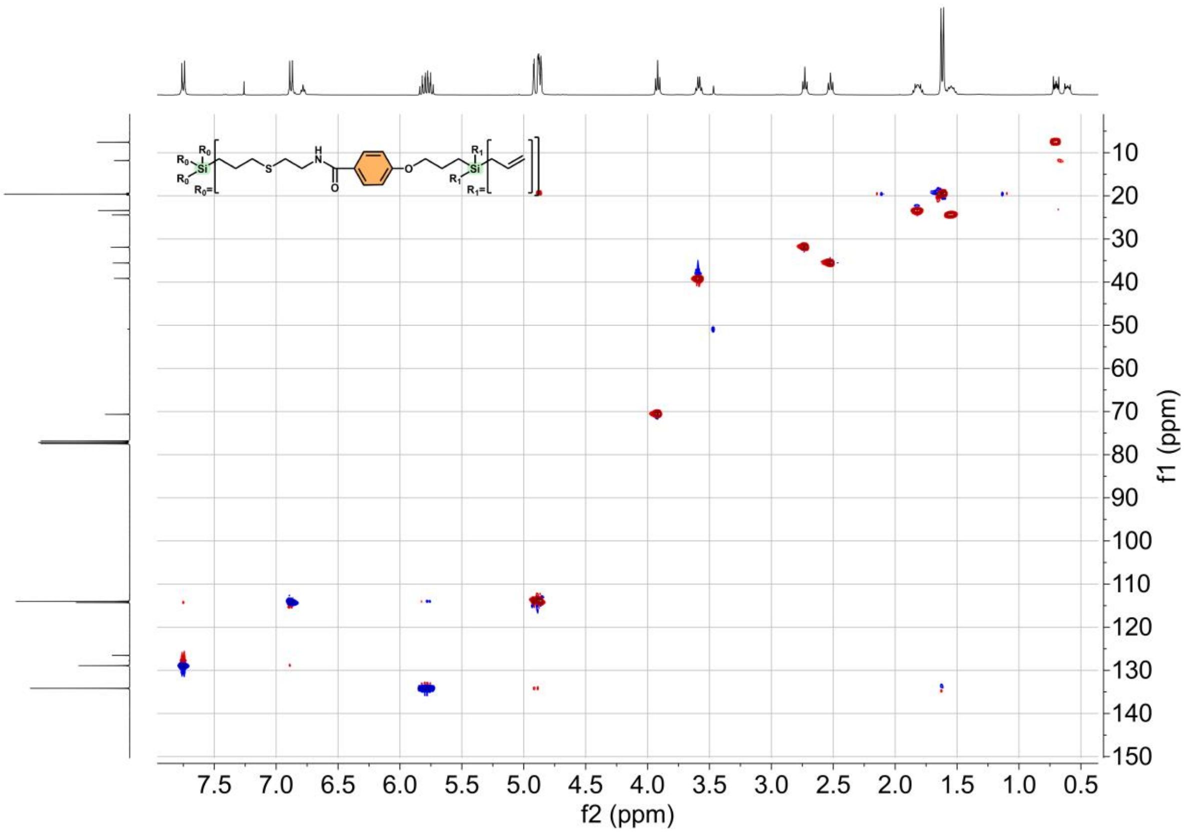

Figure S89: ^{1}H-^{13}C HSQC NMR ($CDCl_3$) **G_1-3-A**

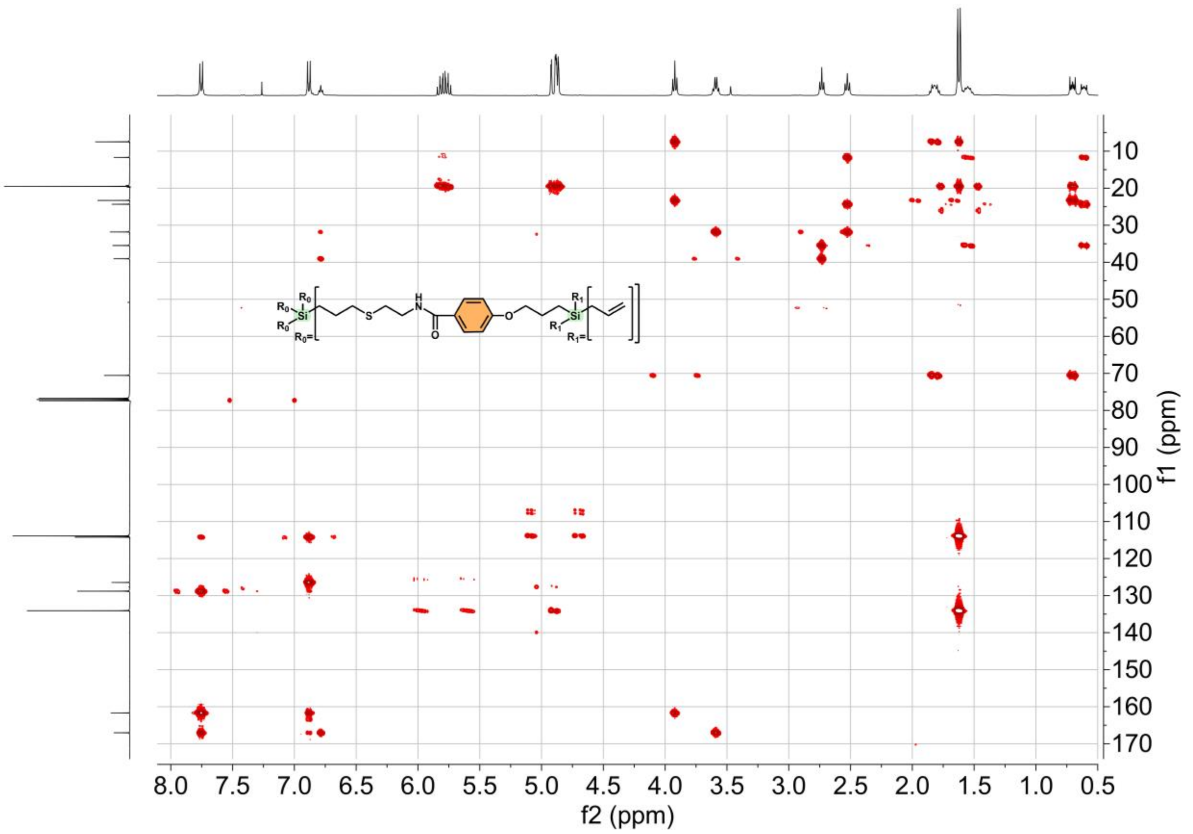

Figure S90: ^{1}H-^{13}C HMBC NMR ($CDCl_3$) **G_1-3-A**

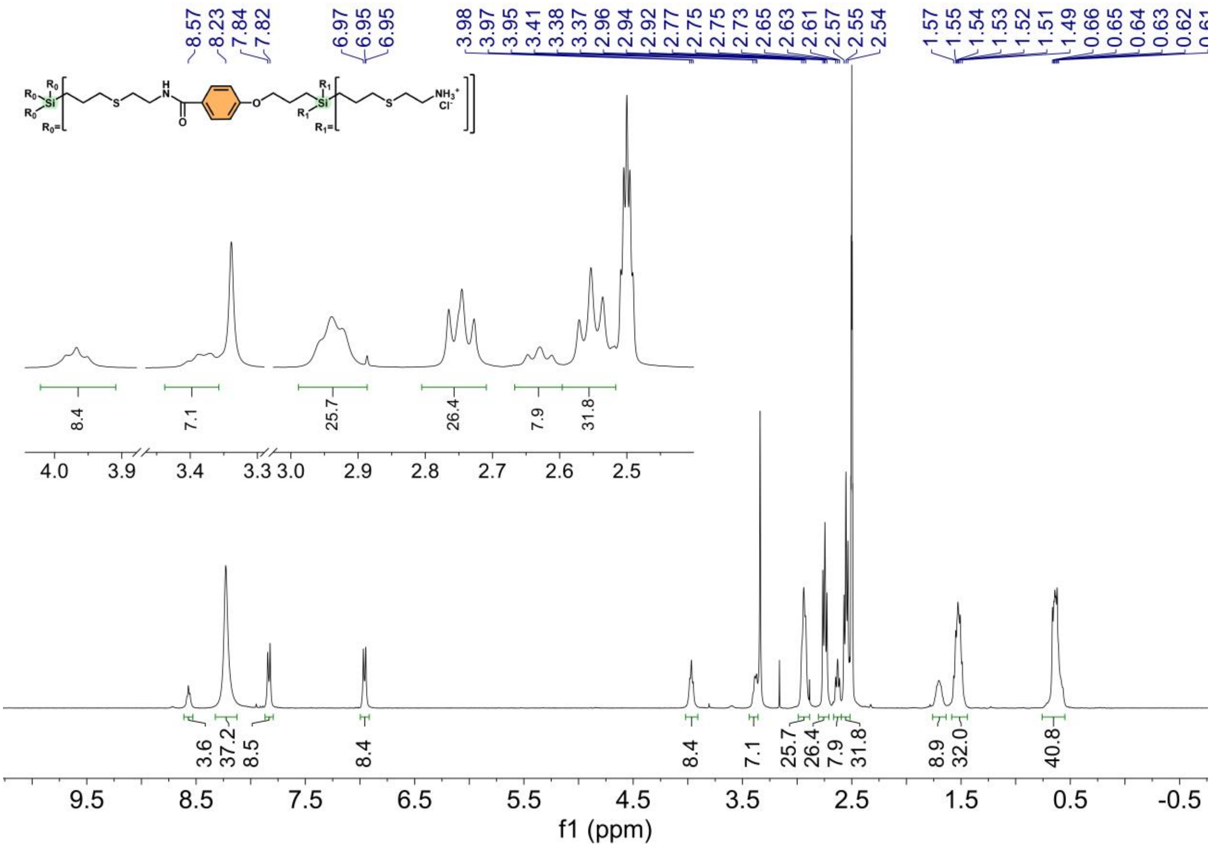

Figure S91: ^{1}H NMR (400 MHz, DMSO-d_6) **G_1-3-N**

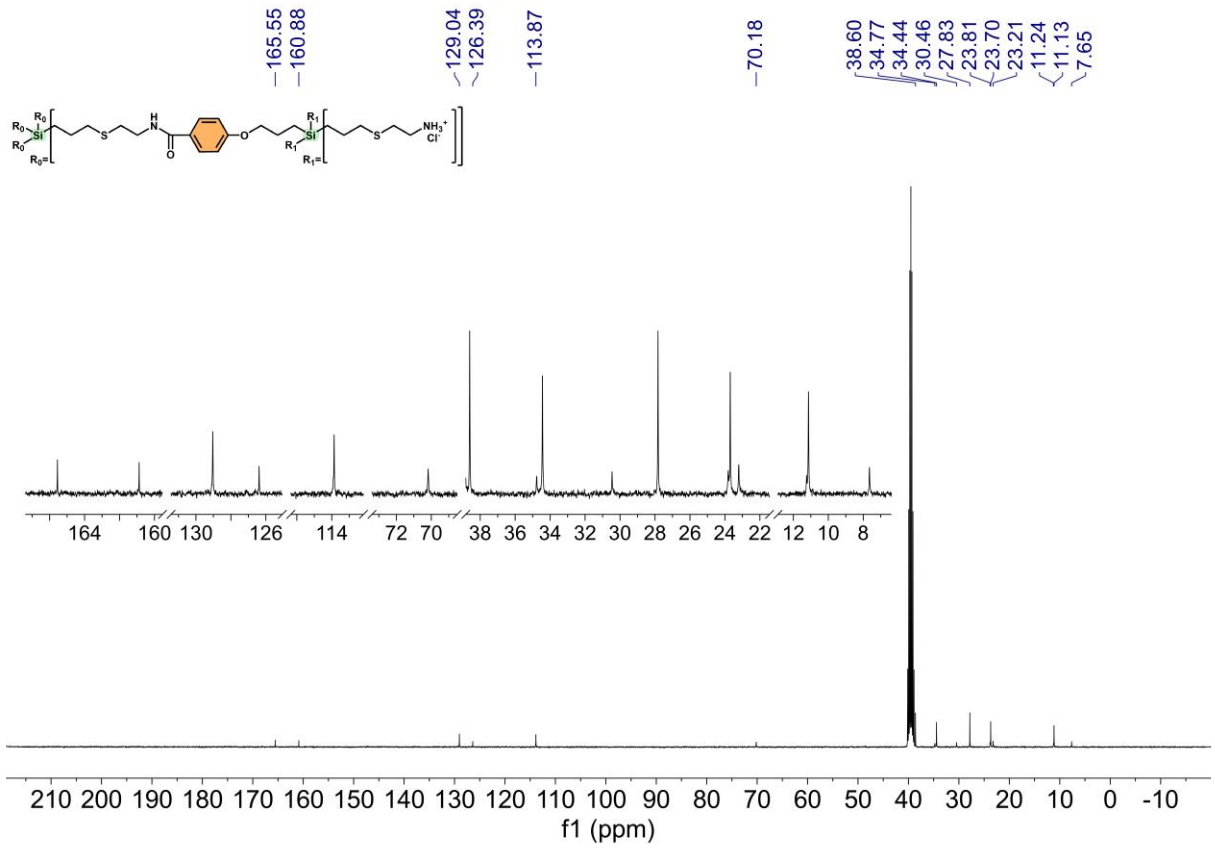

Figure S92: ^{13}C {^{1}H} NMR (101 MHz, DMSO-d_6) **G_1-3-N**

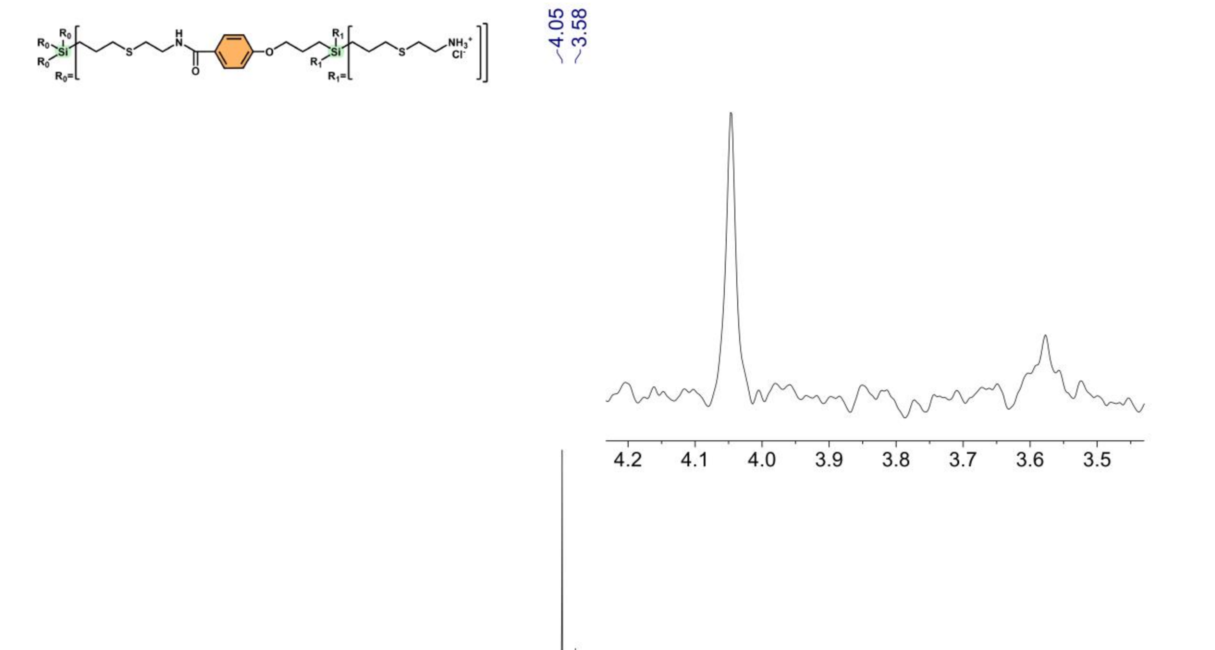

Figure S93: ^{29}Si {^{1}H} NMR (79 MHz, DMSO-d_6) **G_1-3-N**

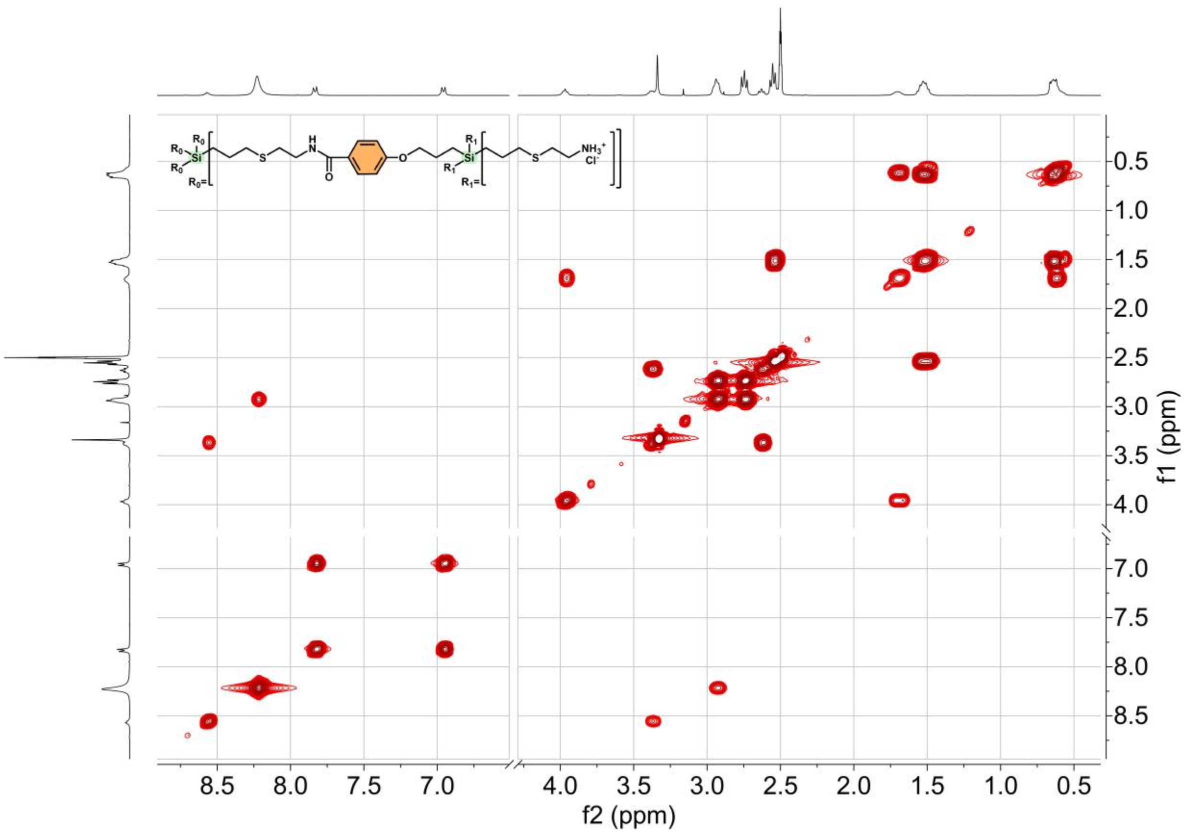

Figure S94: ^{1}H-^{1}H COSY NMR (DMSO-d_6) **G_1-3-N**

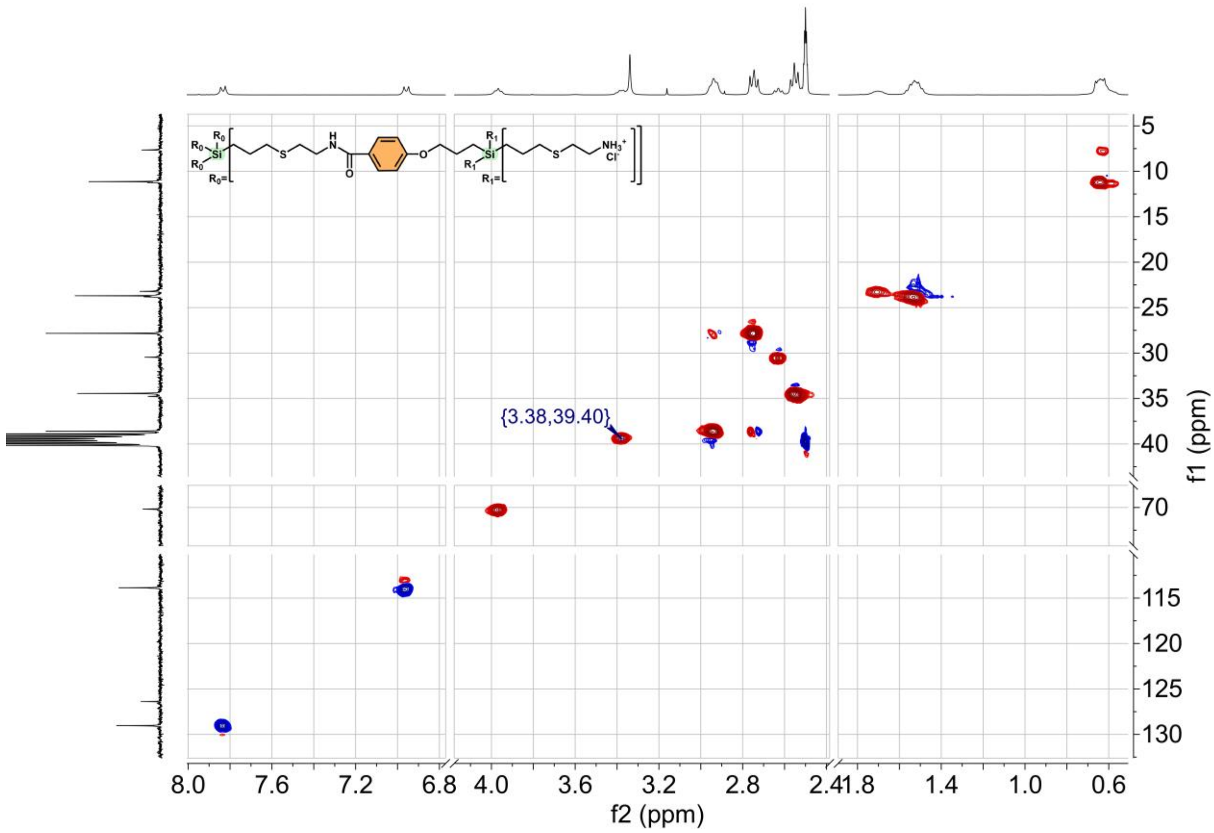

Figure S95: ^{1}H-^{13}C HSQC NMR (DMSO-d_6) **G_1-3-N**

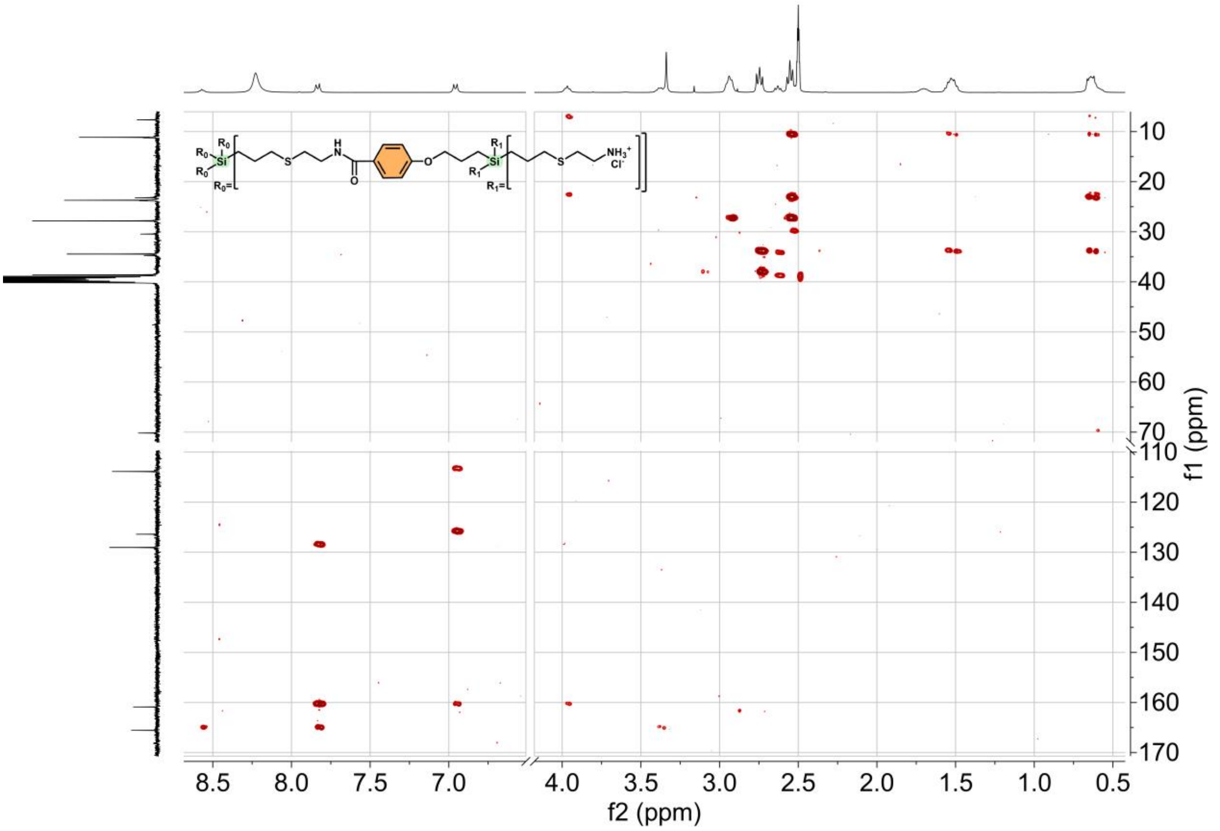

Figure S96: ^{1}H-^{13}C HMBC NMR (DMSO-d_6) **G_1-3-N**

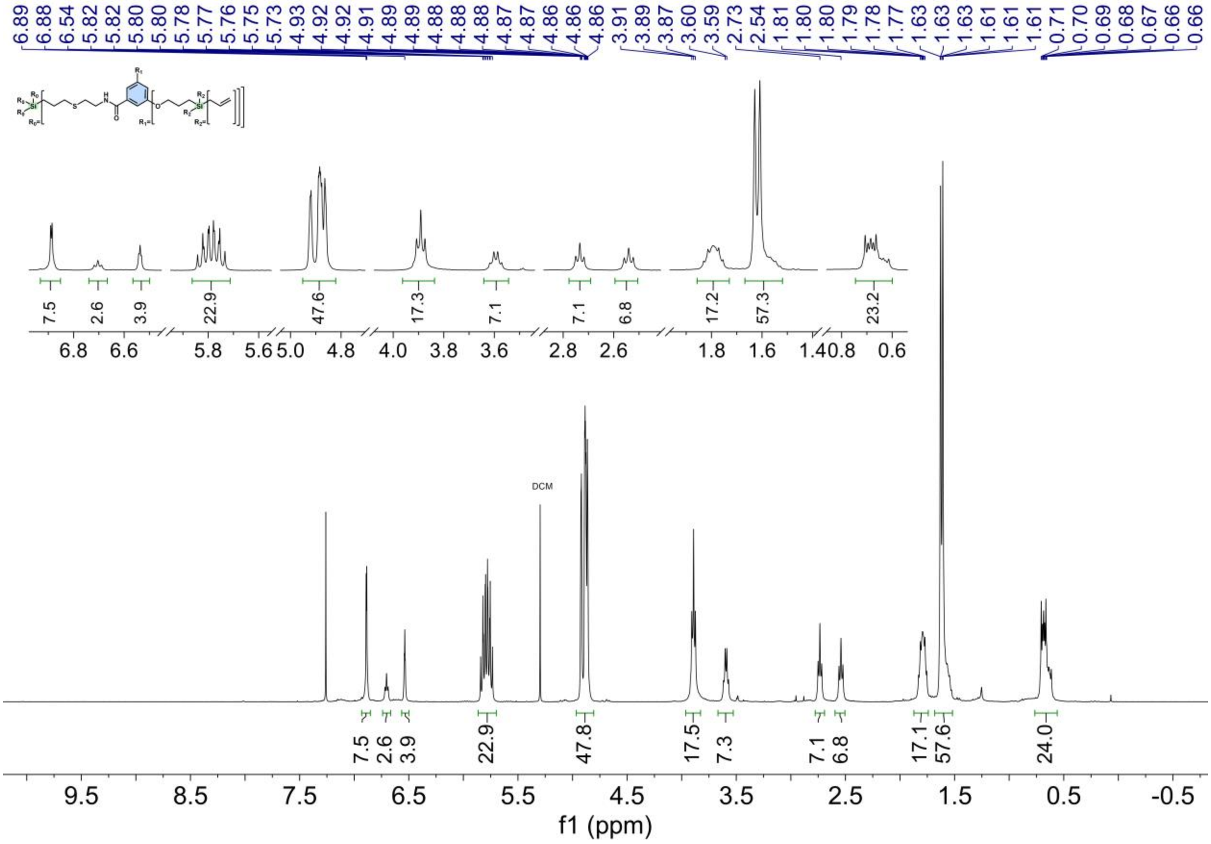

Figure S97: ^{1}H NMR (400 MHz, $CDCl_3$) **G_1-6-A**

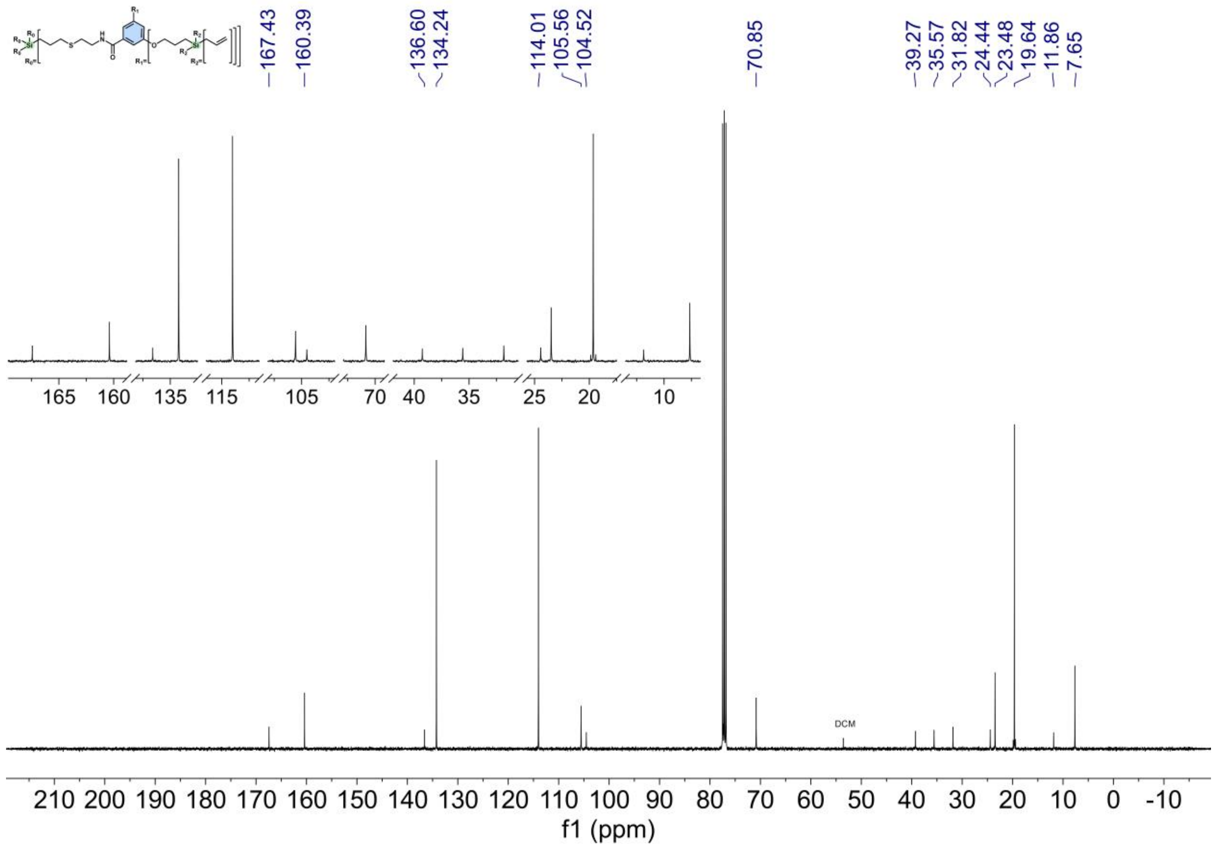

Figure S98: $^{13}C\{^1H\}$ NMR (101 MHz, $CDCl_3$) **G_1-6-A**

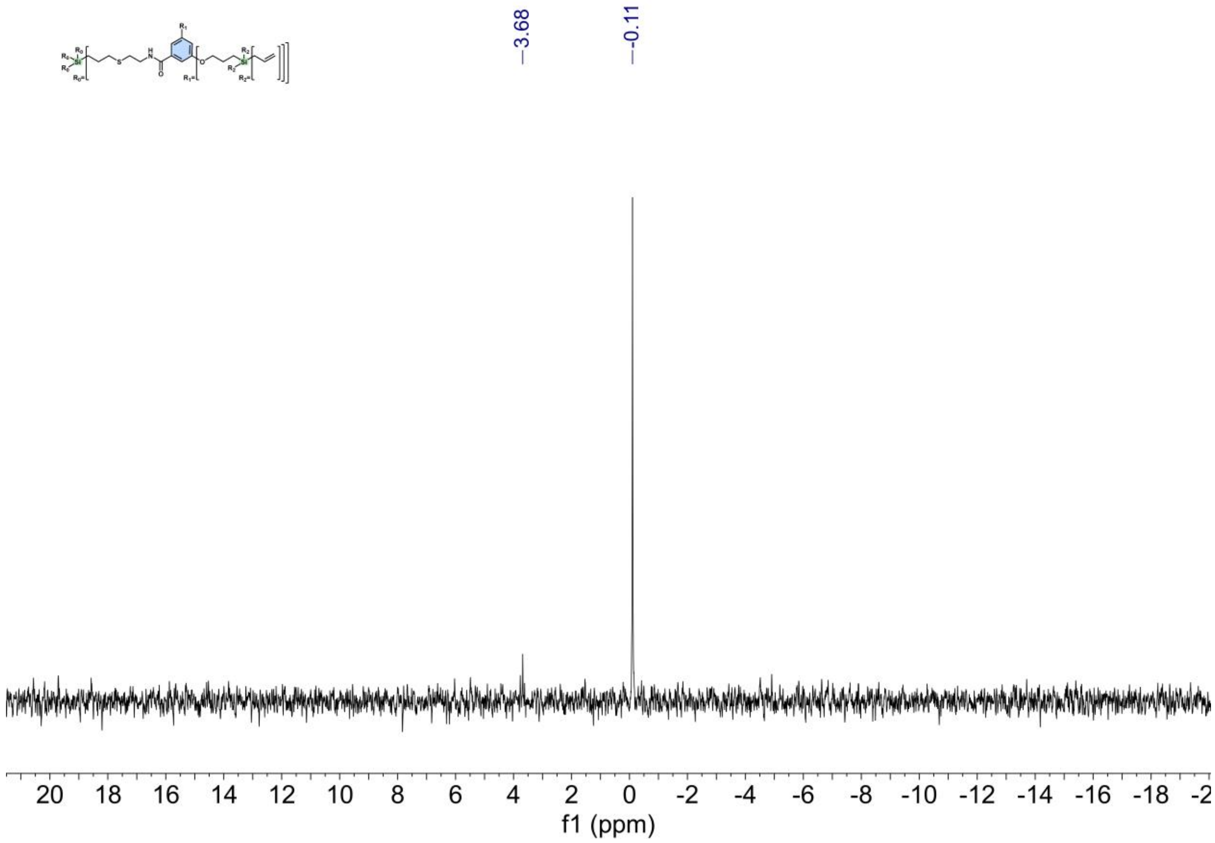

Figure S99: $^{29}Si\{^1H\}$ NMR (79 MHz, $CDCl_3$) **G_1-6-A**

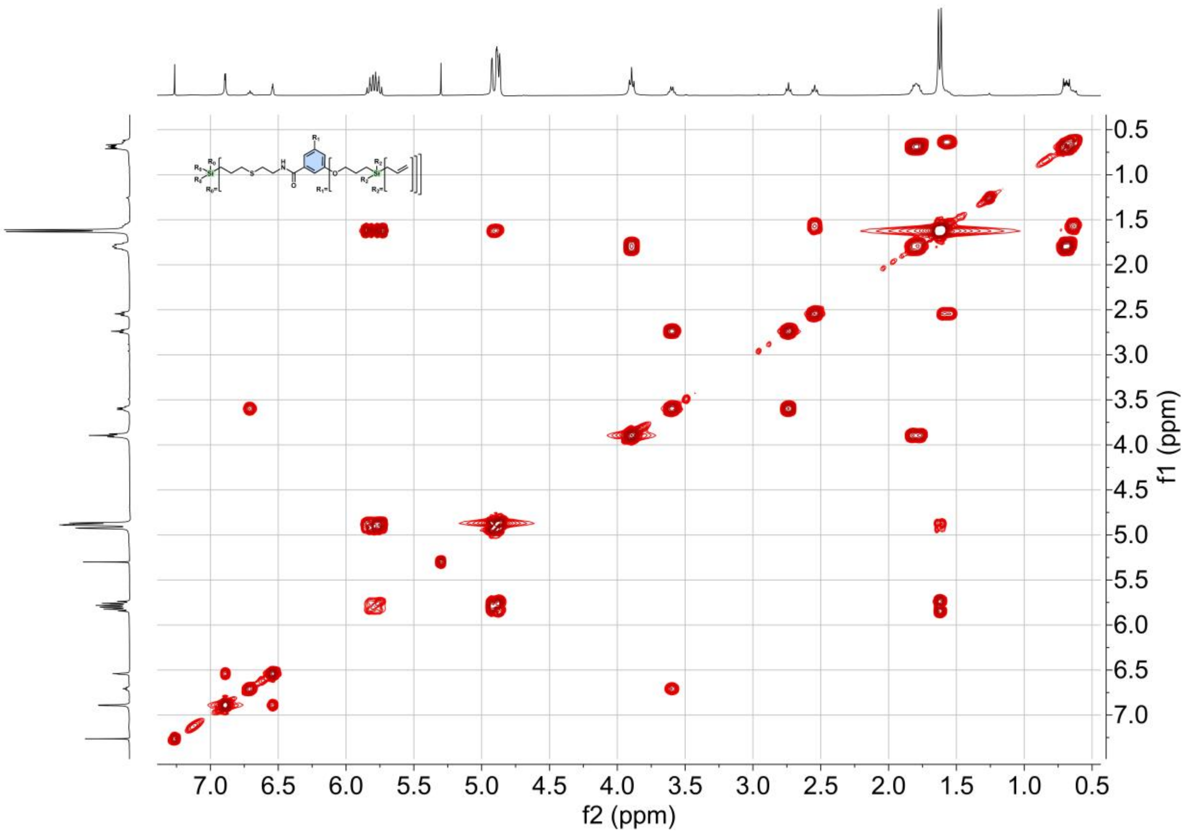

Figure S100: ^{1}H-^{1}H COSY NMR (CDCl$_3$) **G$_1$-6-A**

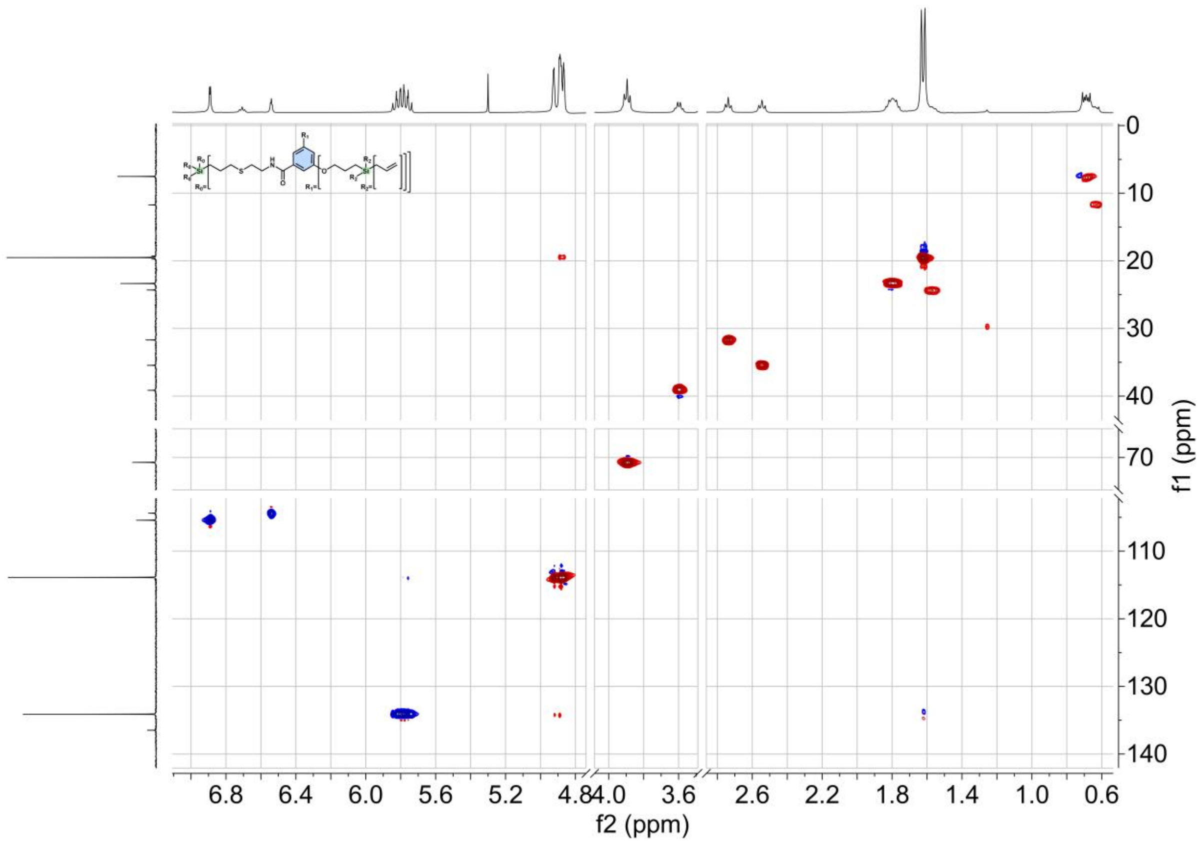

Figure S101: ^{1}H-^{13}C HSQC NMR (CDCl$_3$) **G$_1$-6-A**

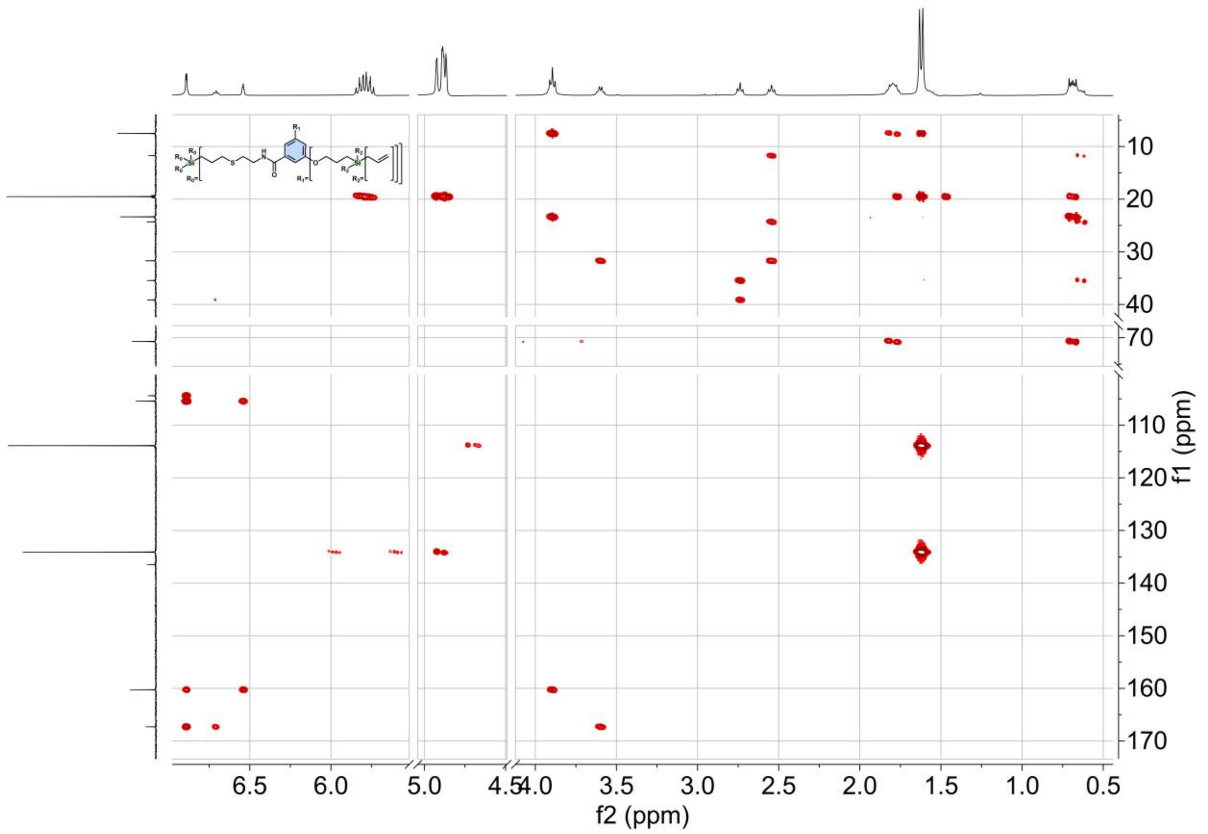

Figure S102: ^{1}H-^{13}C HMBC NMR (CDCl$_3$) **G$_1$-6-A**

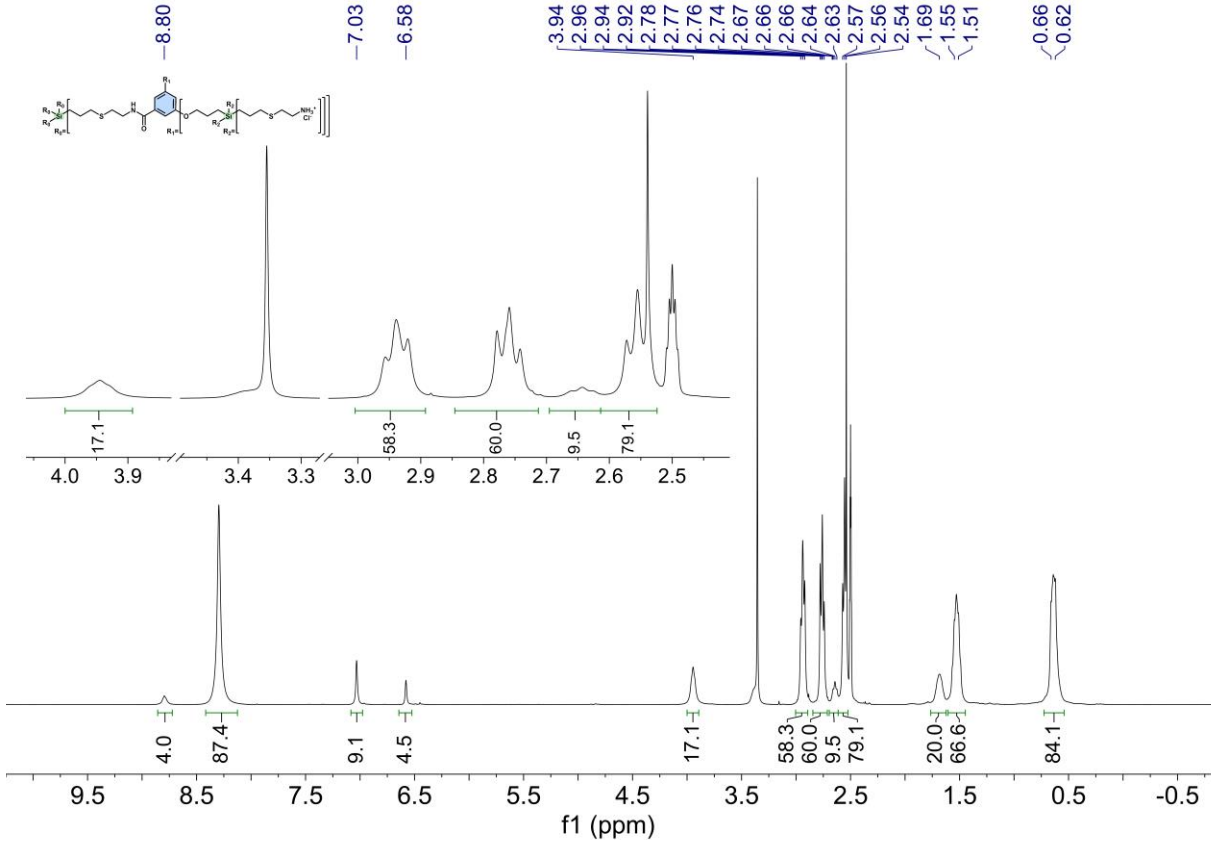

Figure S103: ^{1}H NMR (400 MHz, DMSO-d_6) **G$_1$-6-N**

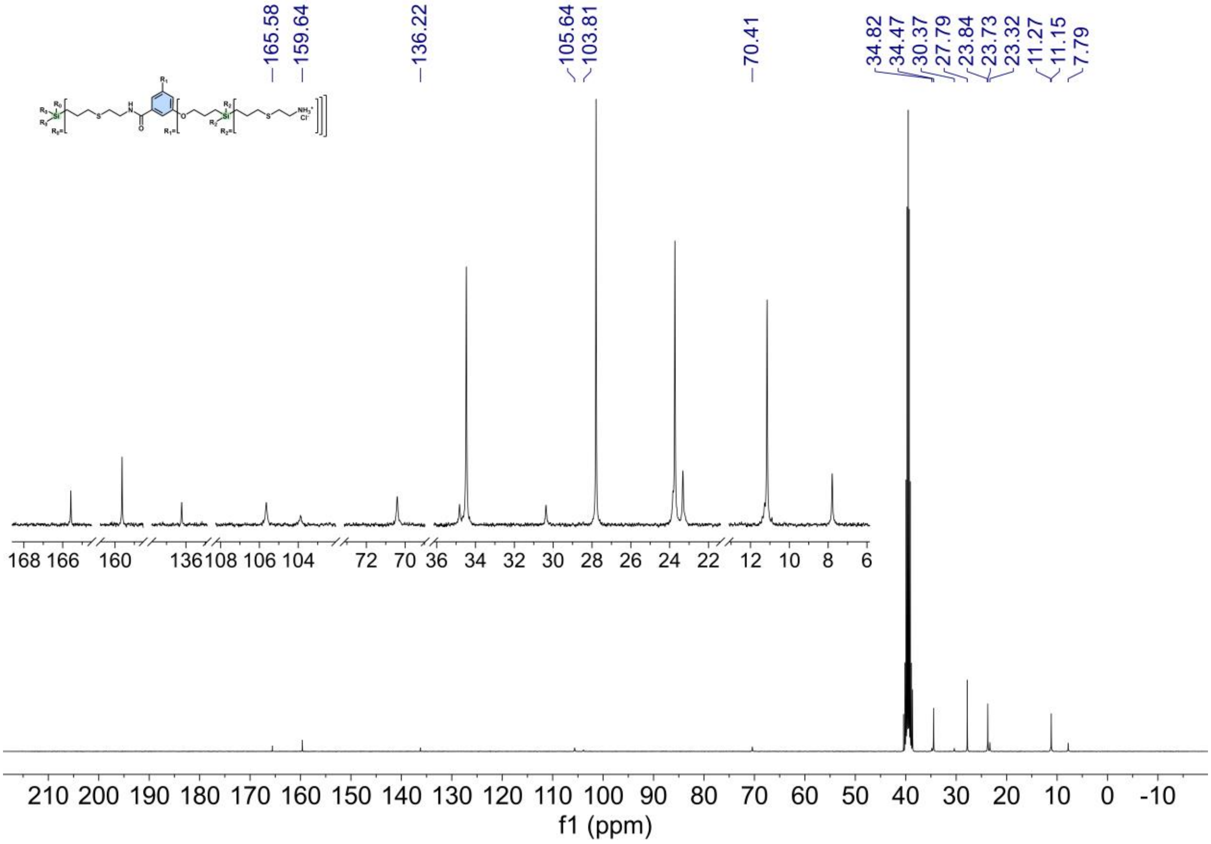

Figure S104: ^{13}C {^{1}H} NMR (101 MHz, DMSO-d_6) **G_1-6-N**

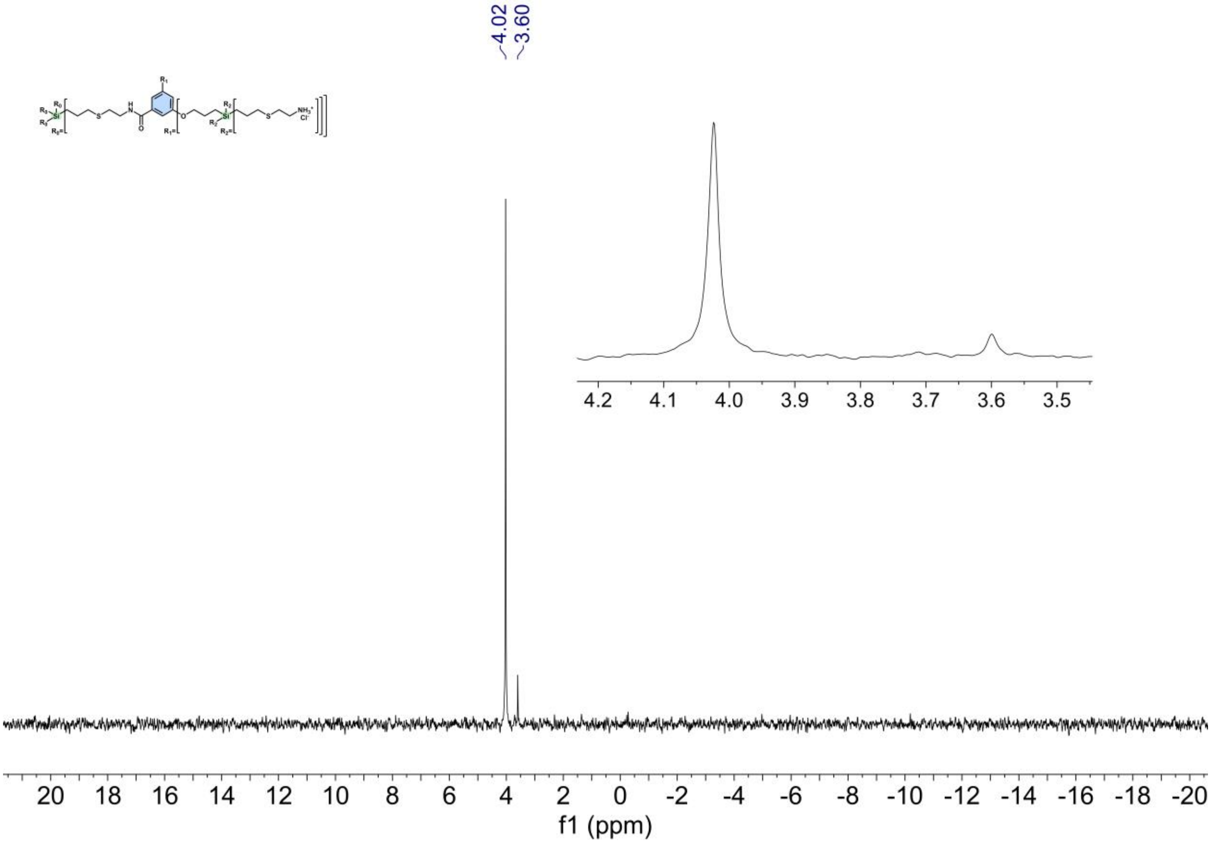

Figure S105: ^{29}Si {^{1}H} NMR (79 MHz, DMSO-d_6) **G_1-6-N**

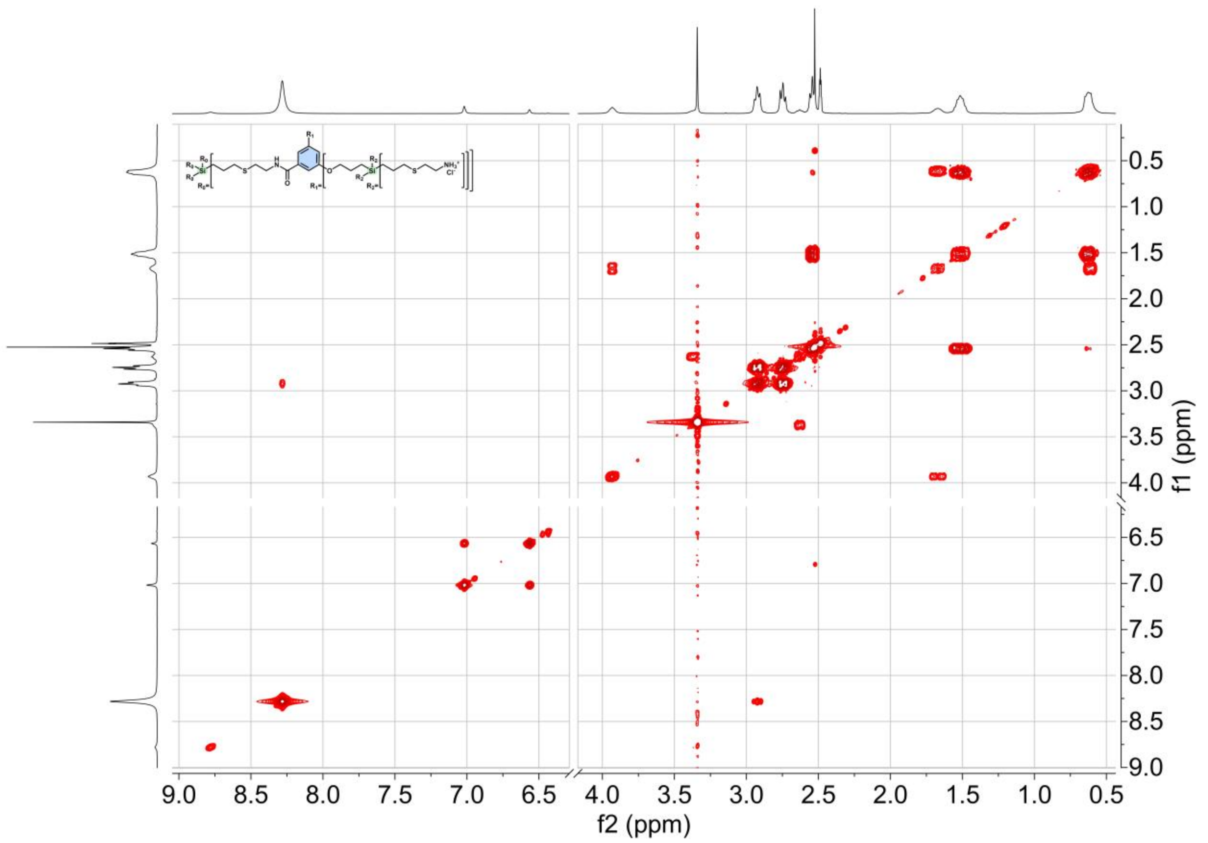

Figure S106: ^{1}H-^{1}H COSY NMR (DMSO-d_6) **G_1-6-N**

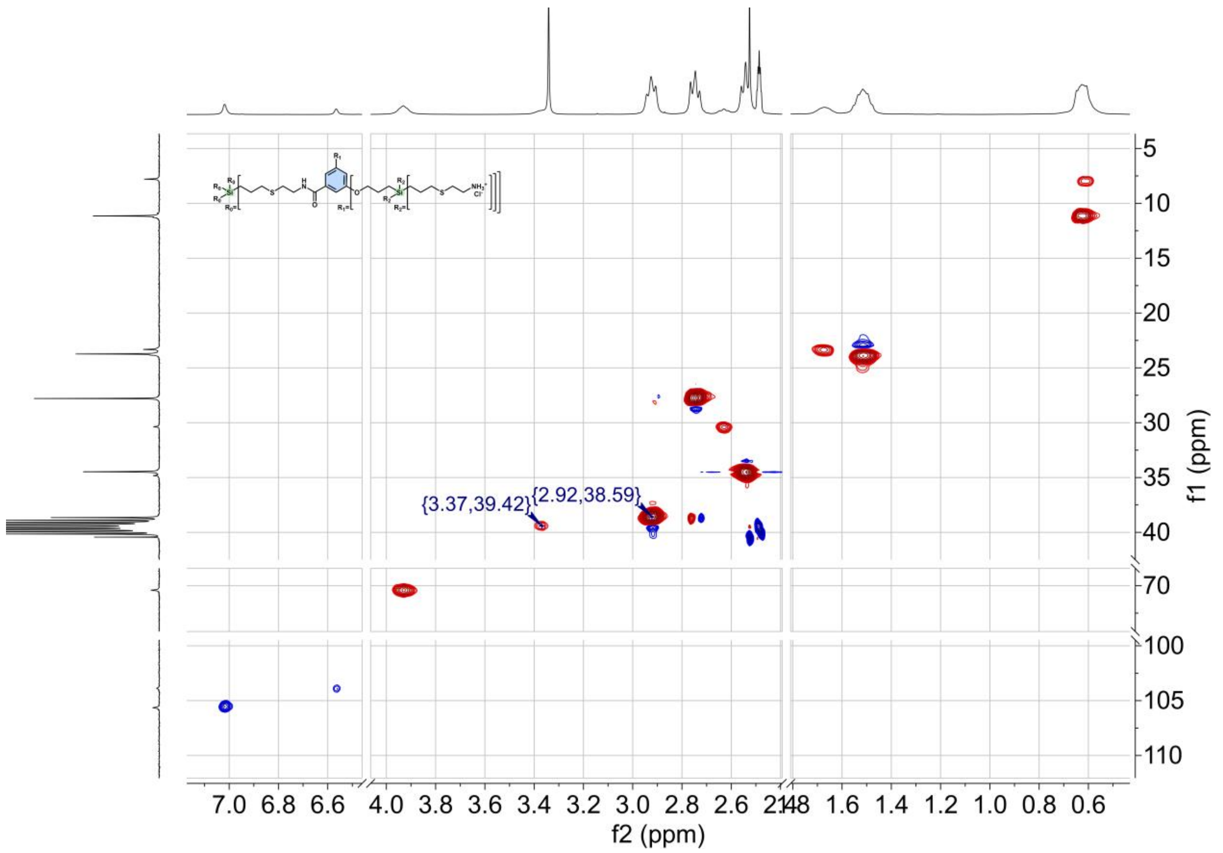

Figure S107: ^{1}H-^{13}C HSQC NMR (DMSO-d_6) **G_1-6-N**

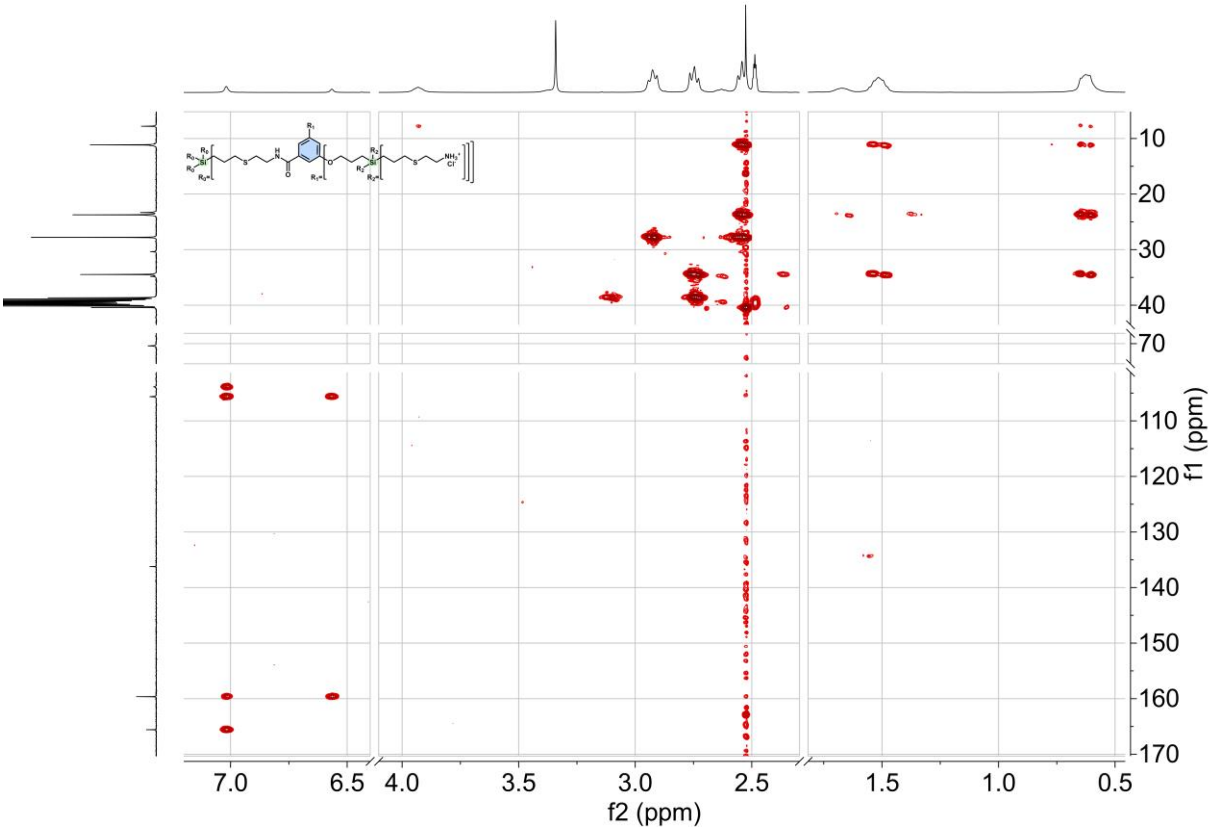

Figure S108: ^{1}H-^{13}C HMBC NMR (DMSO-d_6) **G_1-6-N**

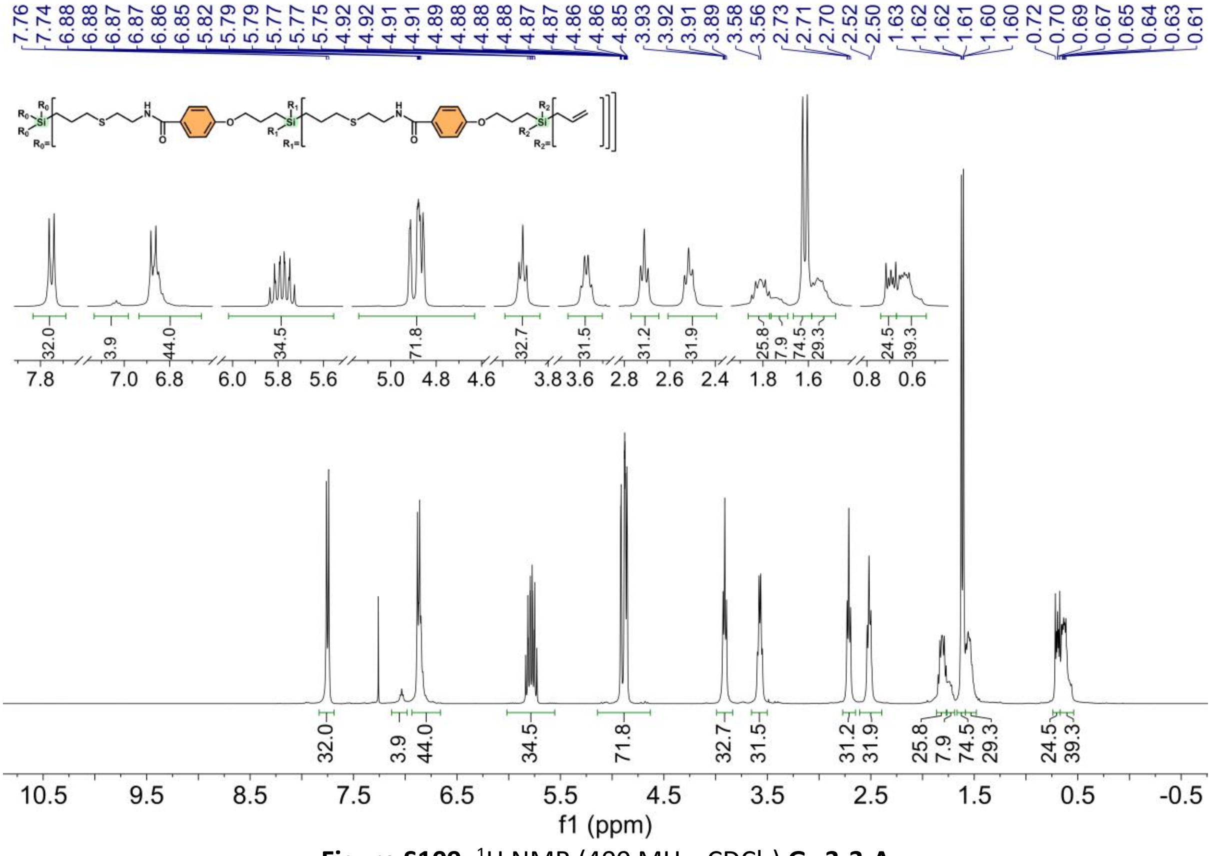

Figure S109: ^{1}H NMR (400 MHz, $CDCl_3$) **G_2-3-3-A**

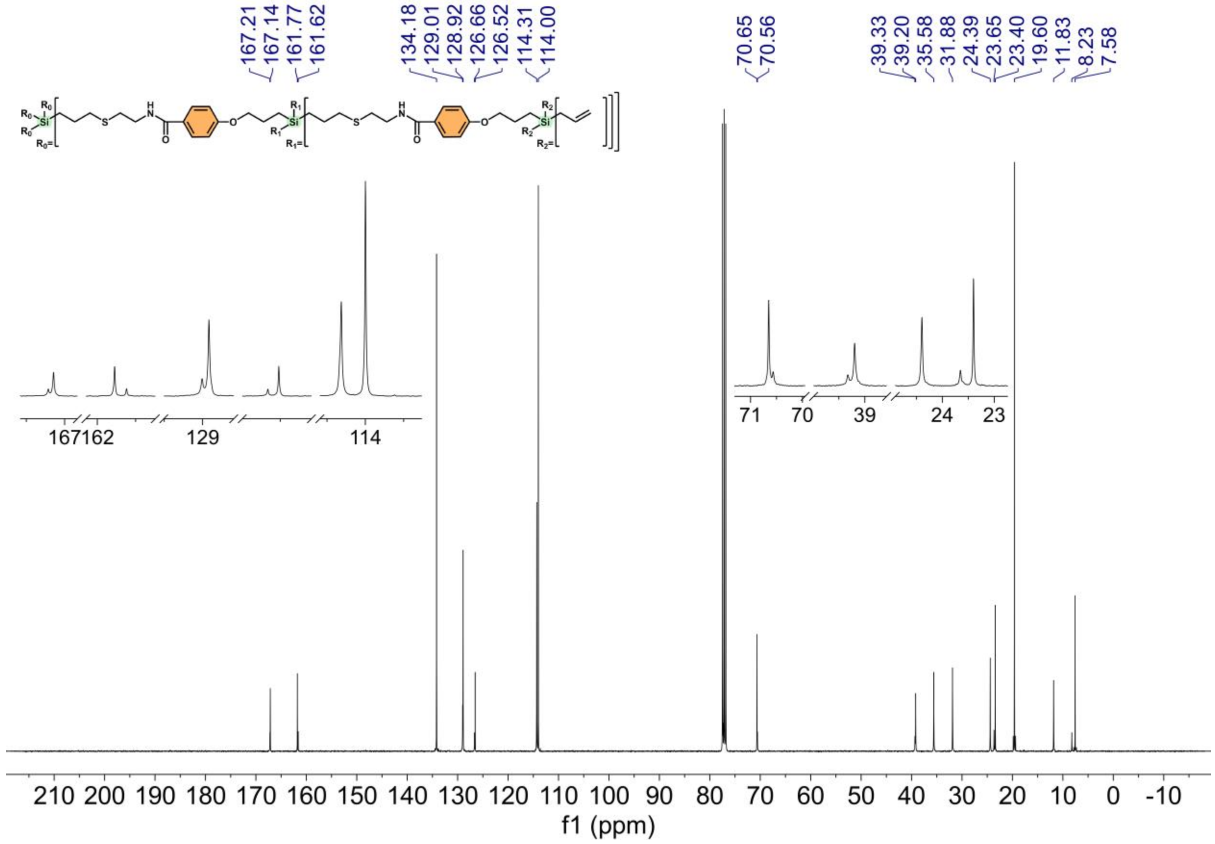

Figure S110: $^{13}C\{^{1}H\}$ NMR (101 MHz, $CDCl_3$) **G_2-3-3-A**

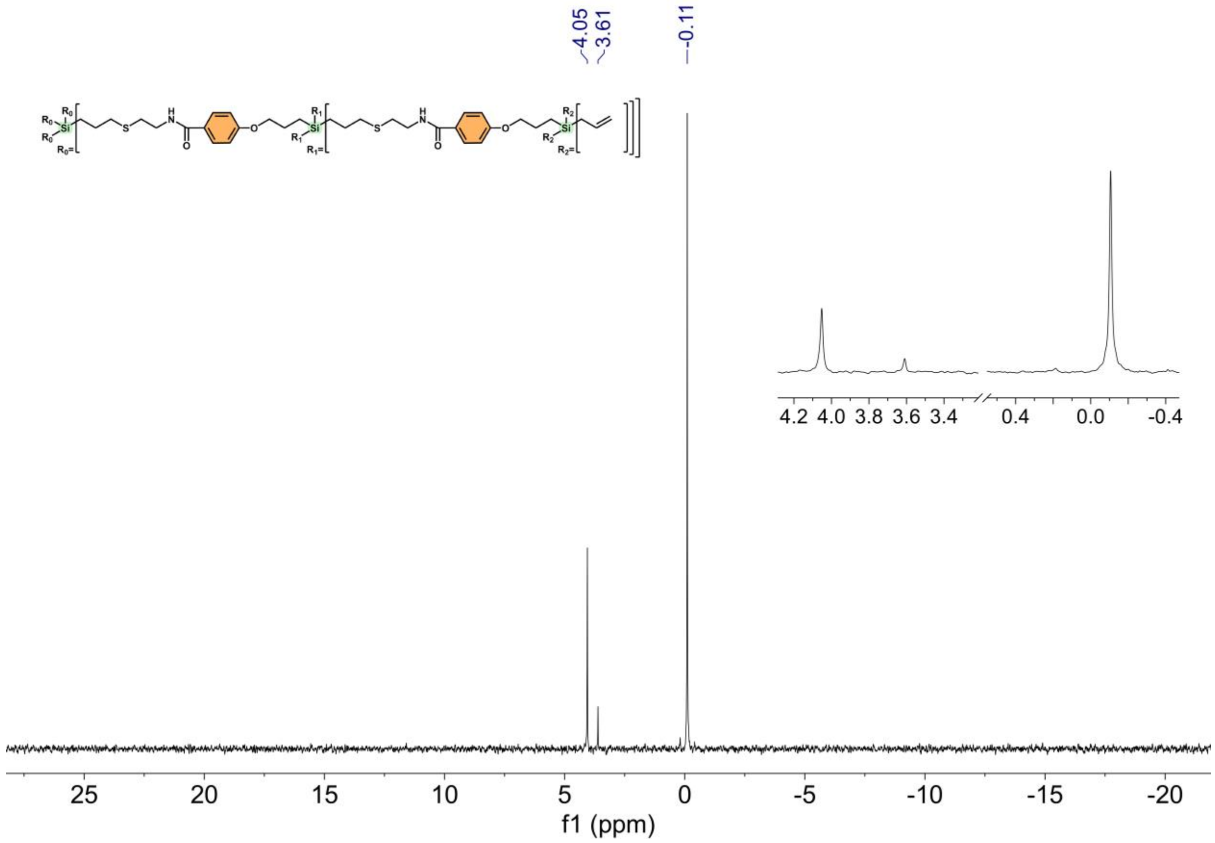

Figure S111: $^{29}Si\{^{1}H\}$ NMR (79 MHz, $CDCl_3$) **G_2-3-3-A**

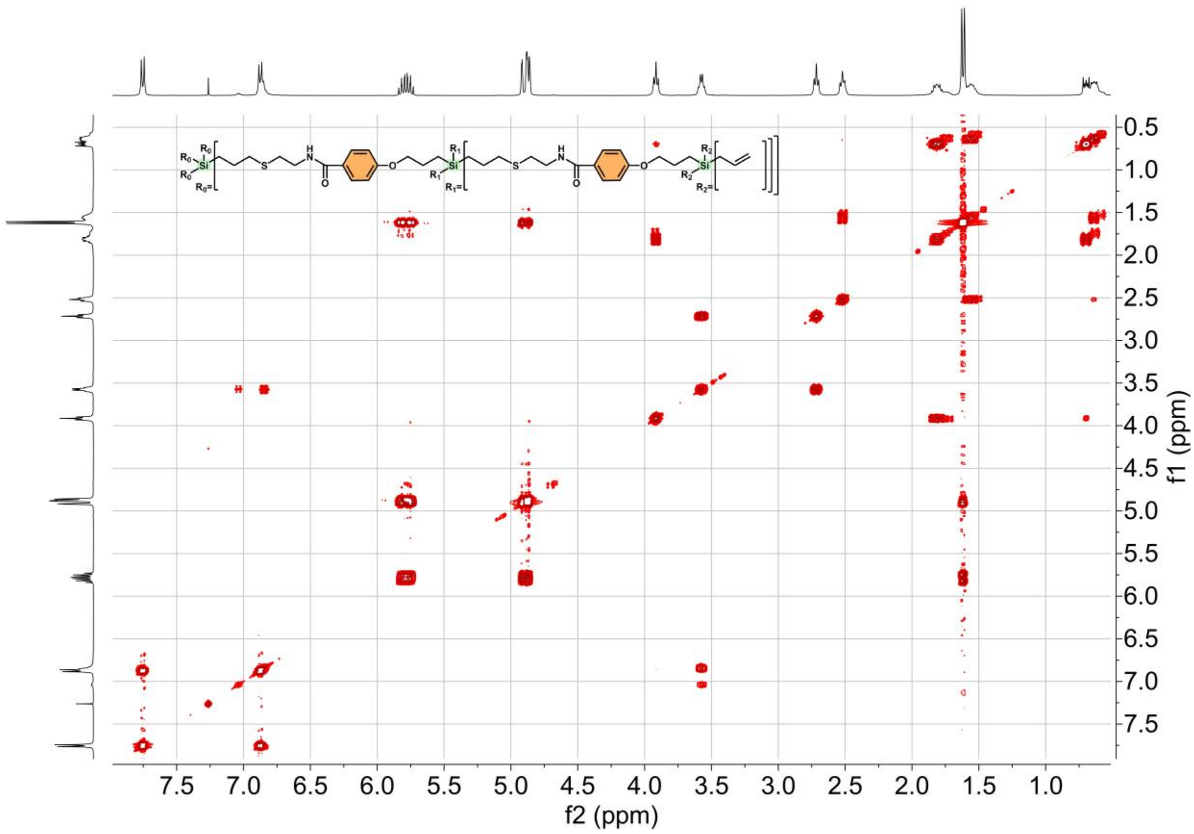

Figure S112: ^{1}H-^{1}H COSY NMR ($CDCl_3$) **G_2-3-3-A**

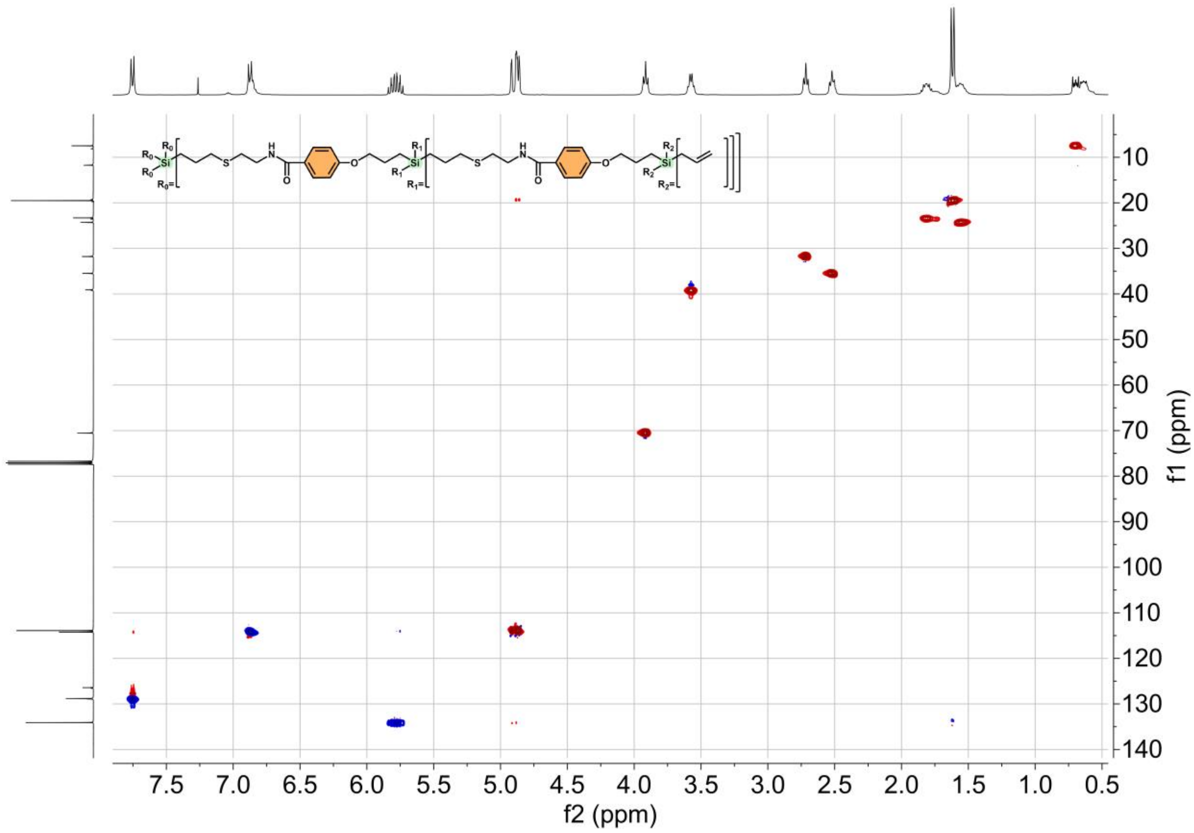

Figure S113: ^{1}H-^{13}C HSQC NMR ($CDCl_3$) **G_2-3-3-A**

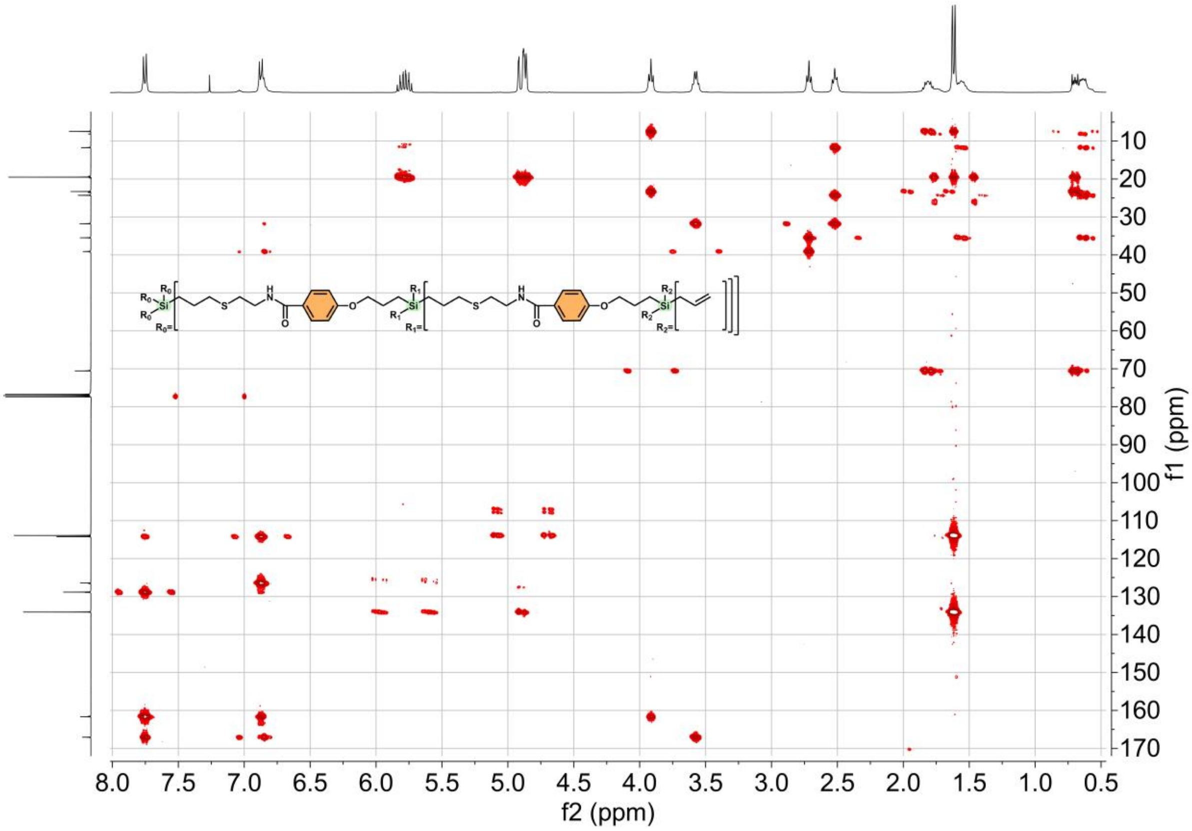

Figure S114: ^{1}H-^{13}C HMBC NMR ($CDCl_3$) **G_2-3-3-A**

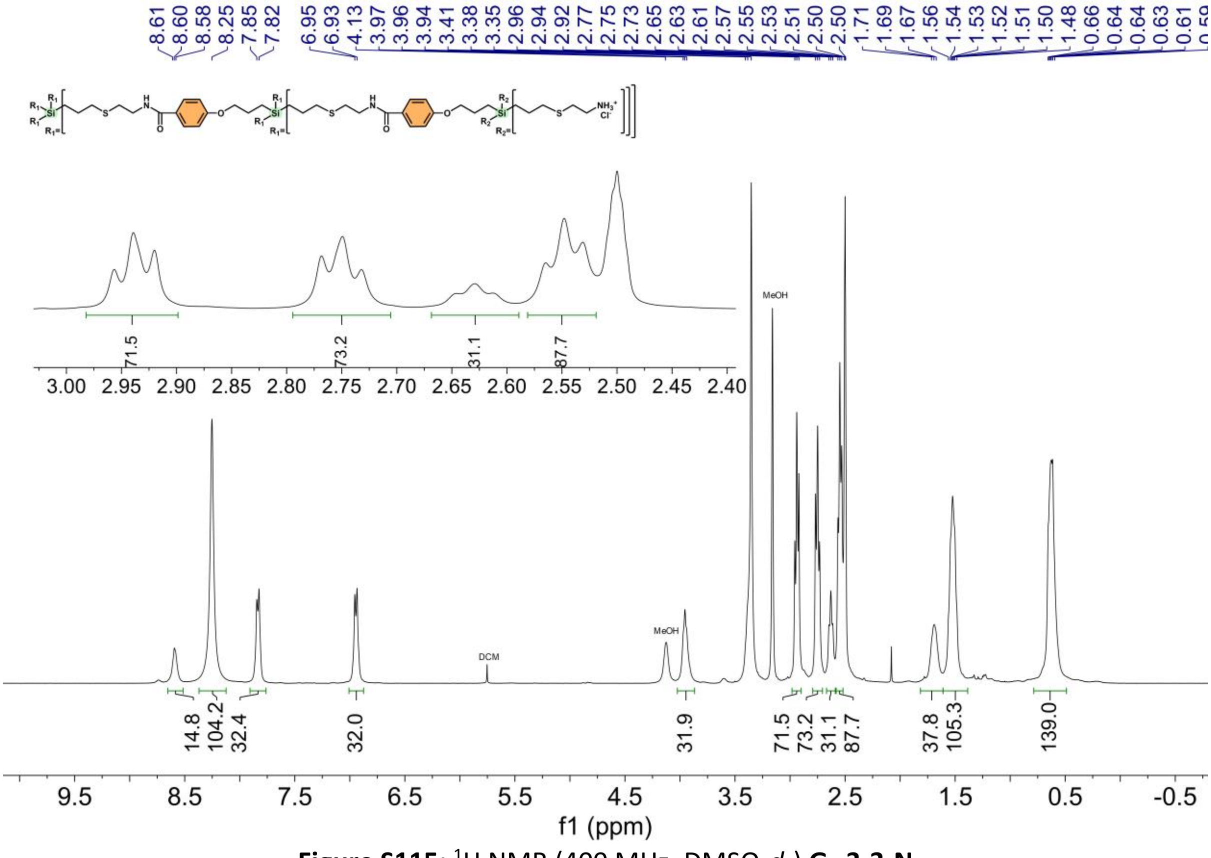

Figure S115: ^{1}H NMR (400 MHz, DMSO-d_6) **G_2-3-3-N**

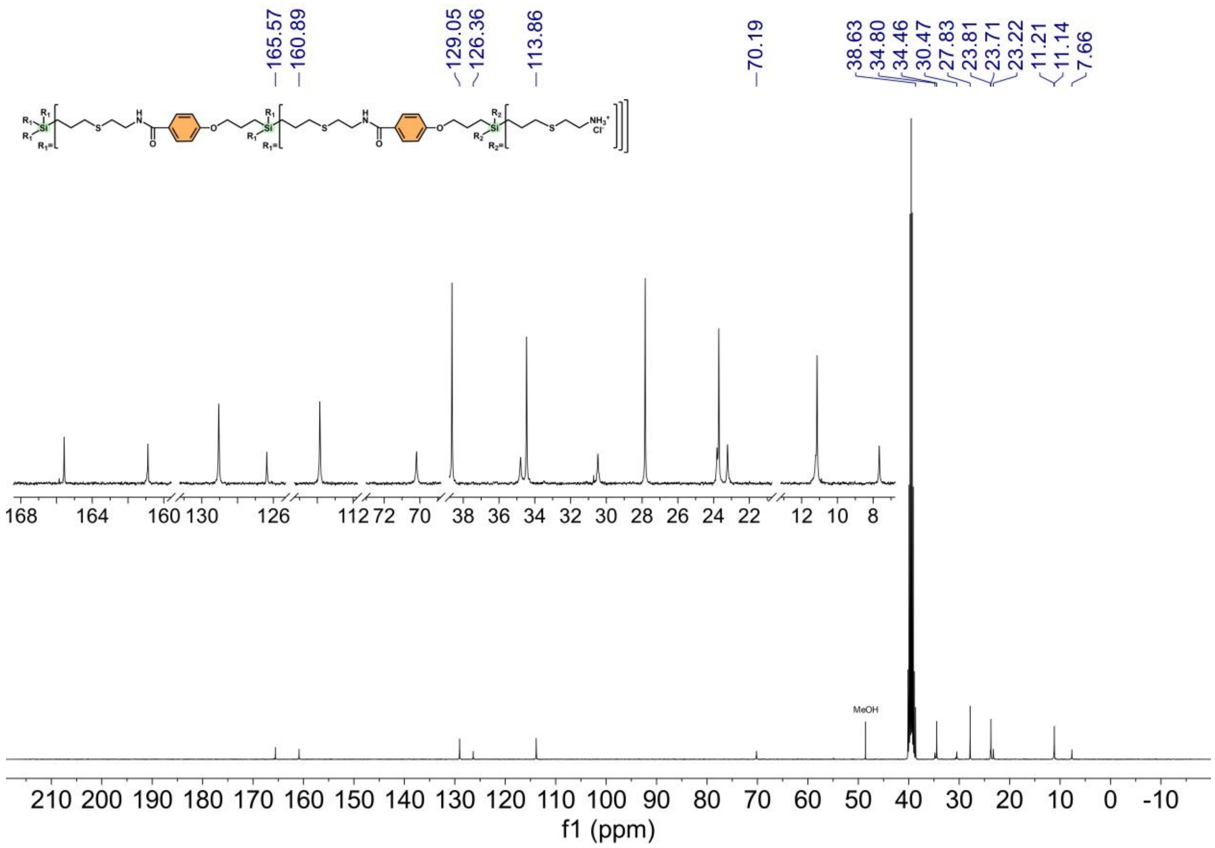

Figure S116: ^{13}C {^{1}H} NMR (101 MHz, DMSO-d_6) **G_2-3-3-N**

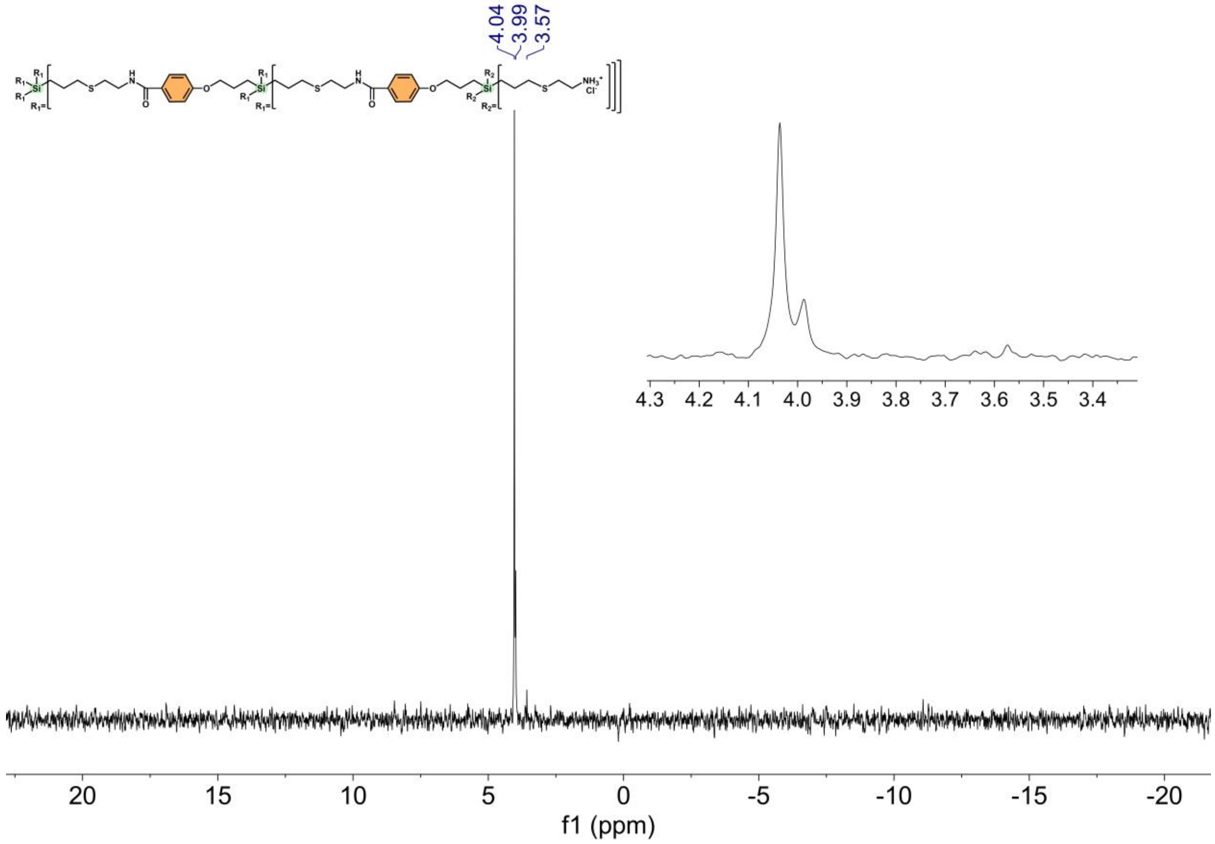

Figure S117: ^{29}Si {^{1}H} NMR (79 MHz, DMSO-d_6) **G_2-3-3-N**

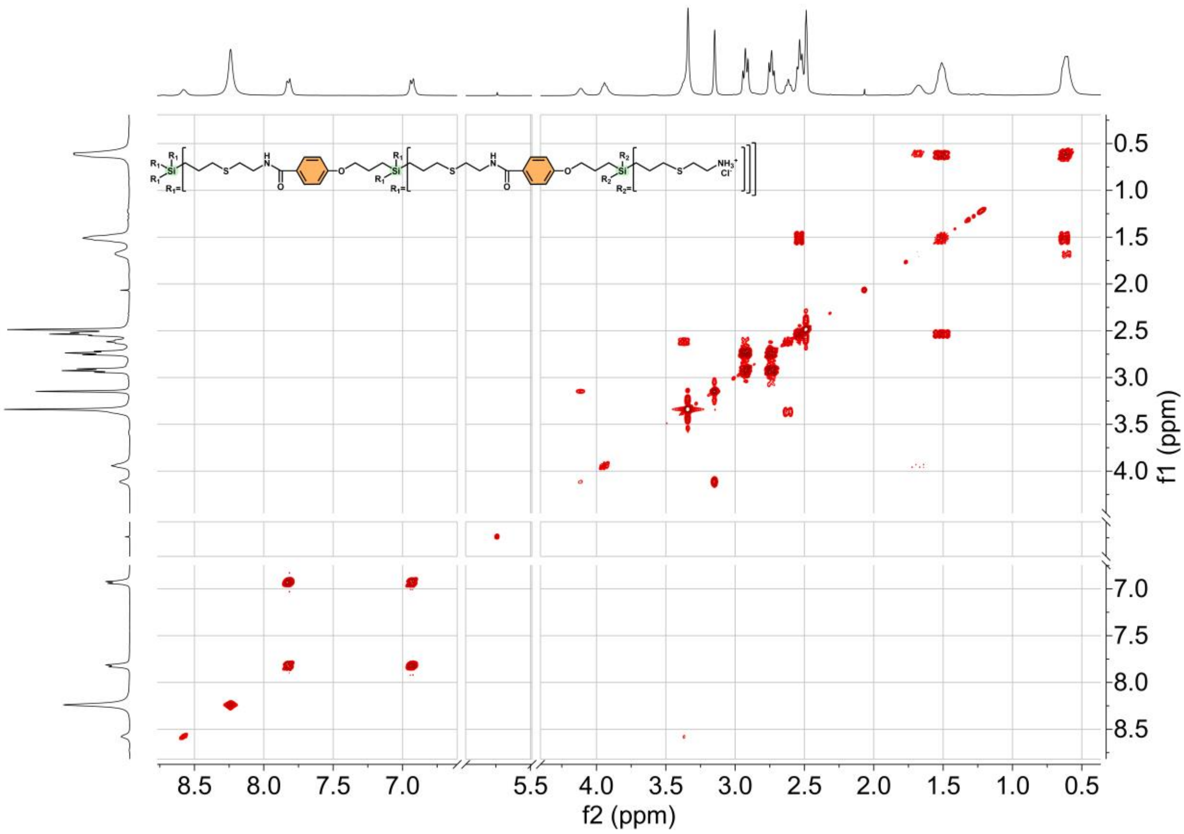

Figure S118: ^{1}H-^{1}H COSY NMR (DMSO-d_6) **G$_2$-3-3-N**

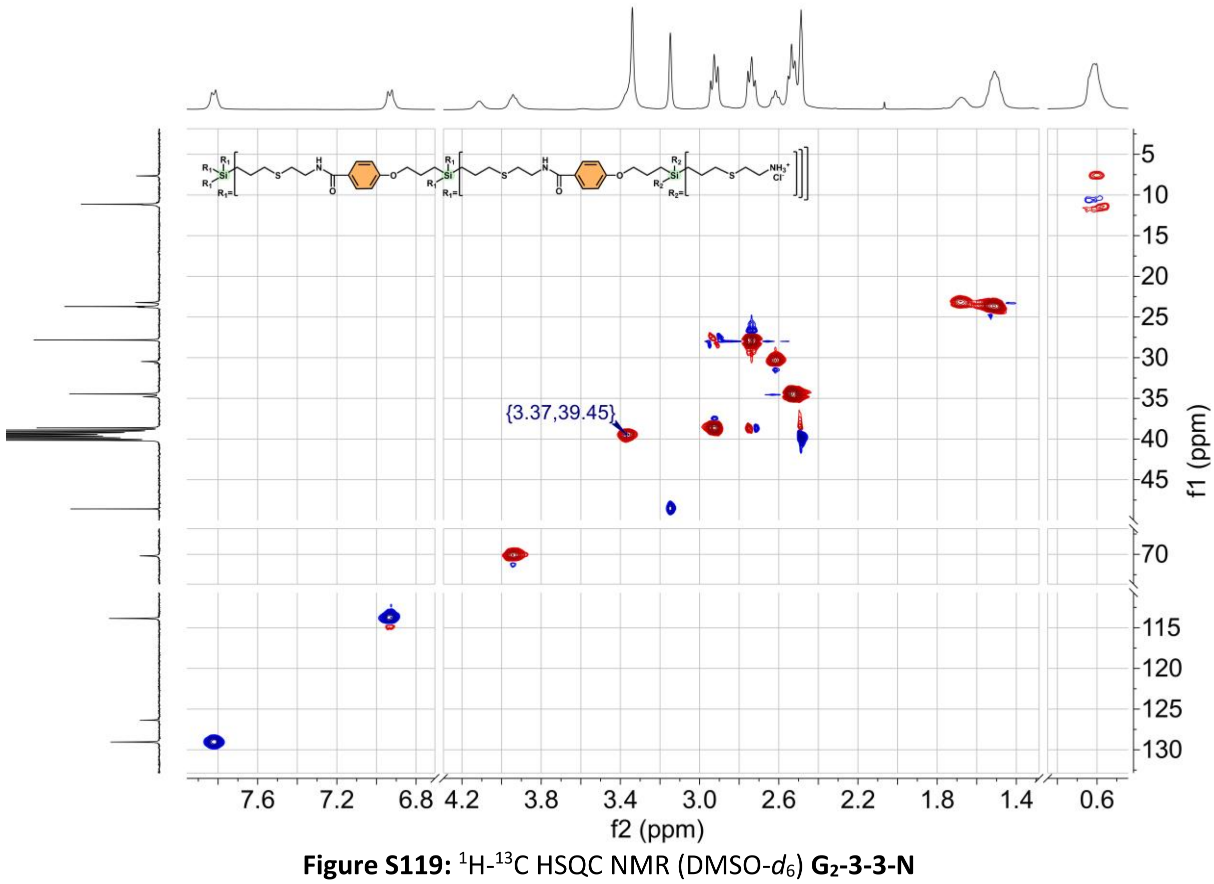

Figure S119: ^{1}H-^{13}C HSQC NMR (DMSO-d_6) **G$_2$-3-3-N**

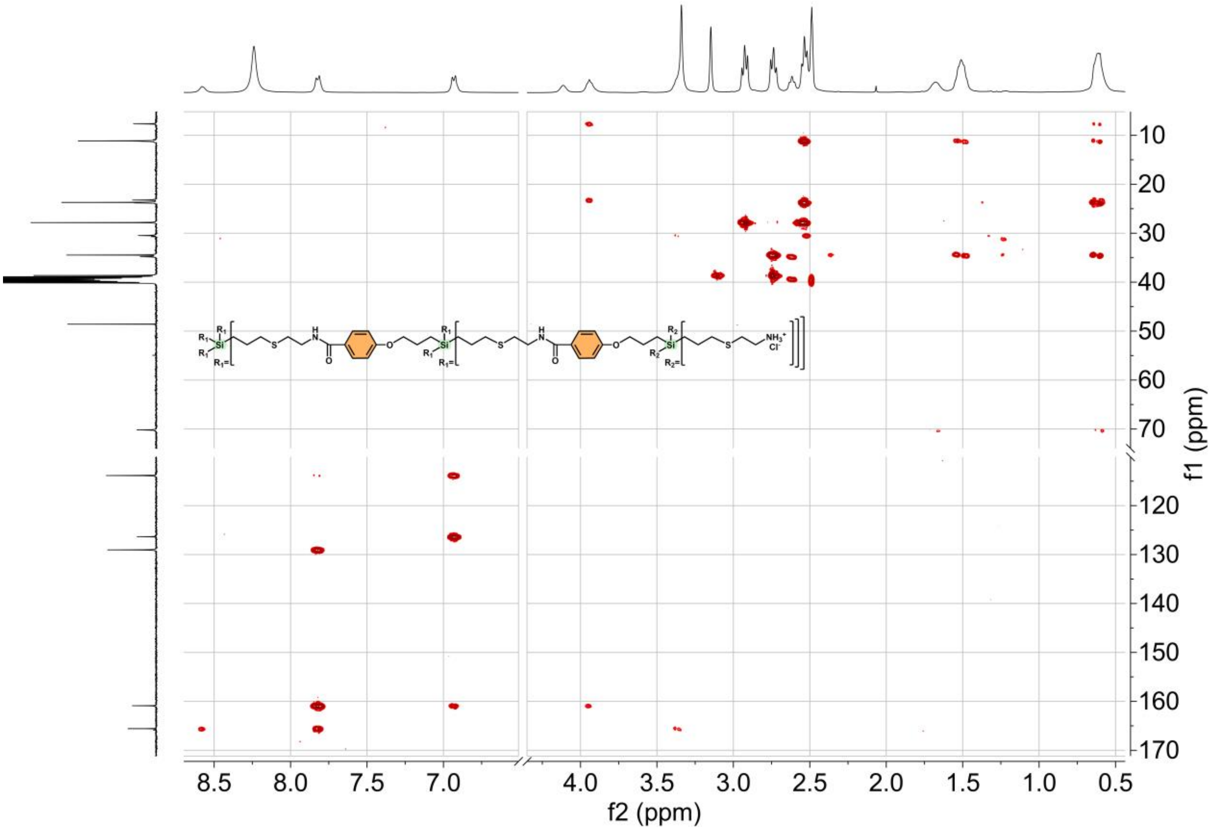

Figure S120: ^{1}H-^{13}C HMBC NMR (DMSO-d_6) **G_2-3-3-N**

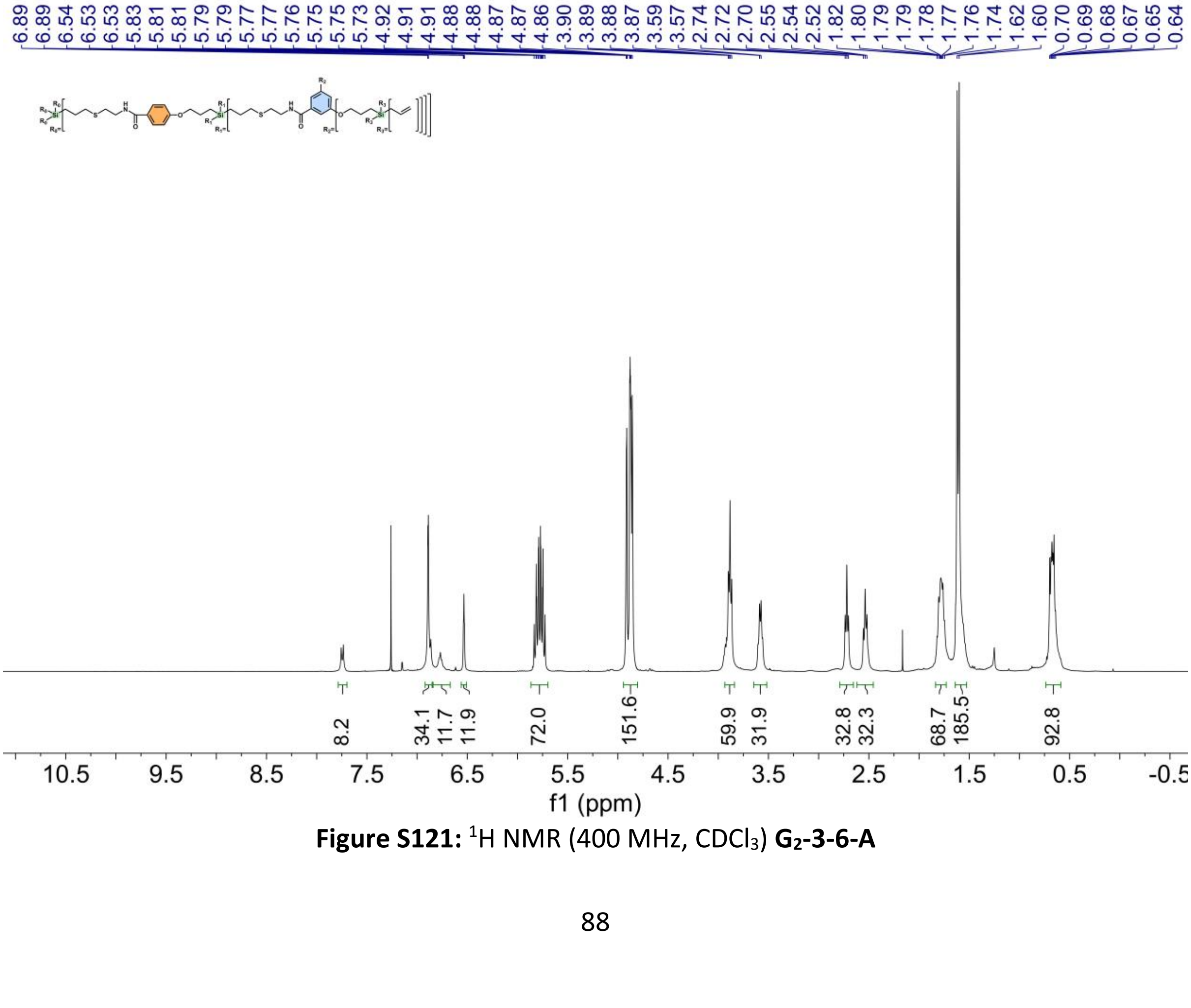

Figure S121: ^{1}H NMR (400 MHz, $CDCl_3$) **G_2-3-6-A**

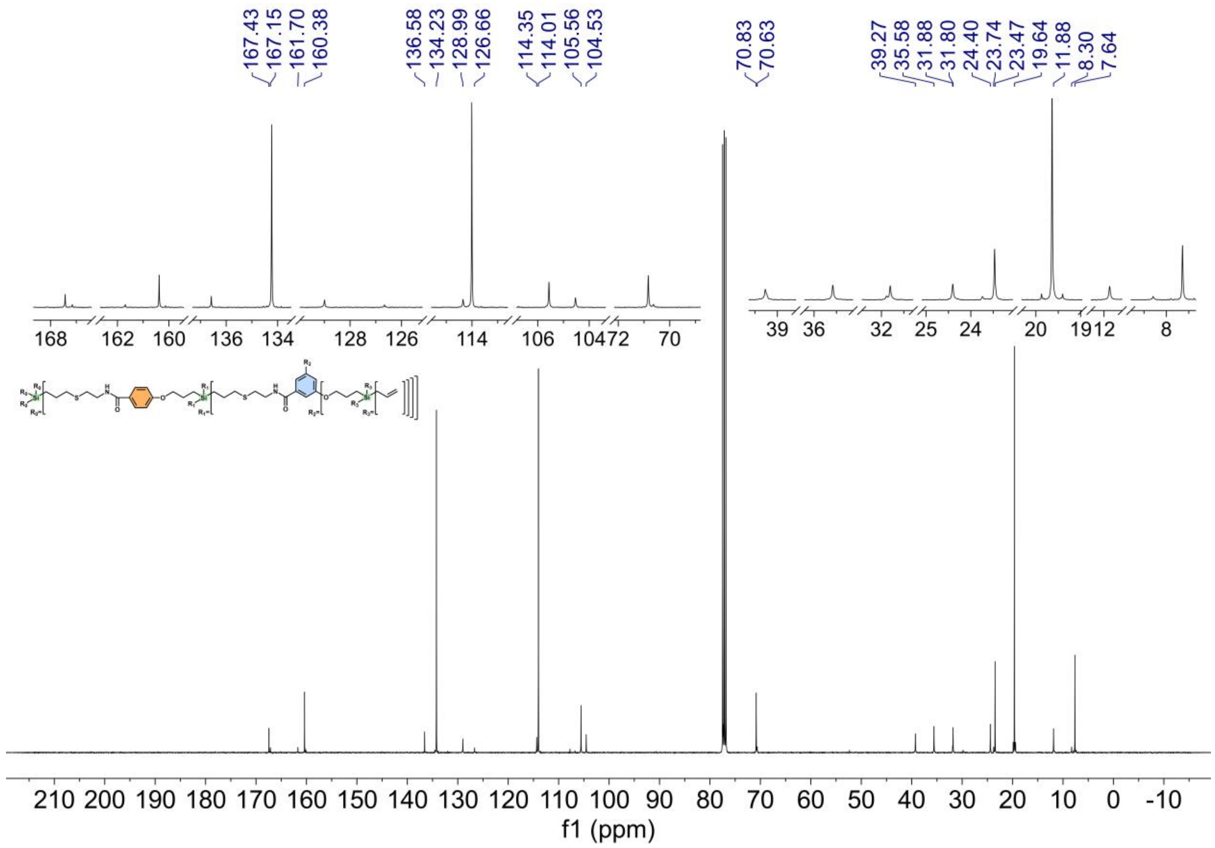

Figure S122: ^{13}C {^{1}H} NMR (101 MHz, $CDCl_3$) **G_2-3-6-A**

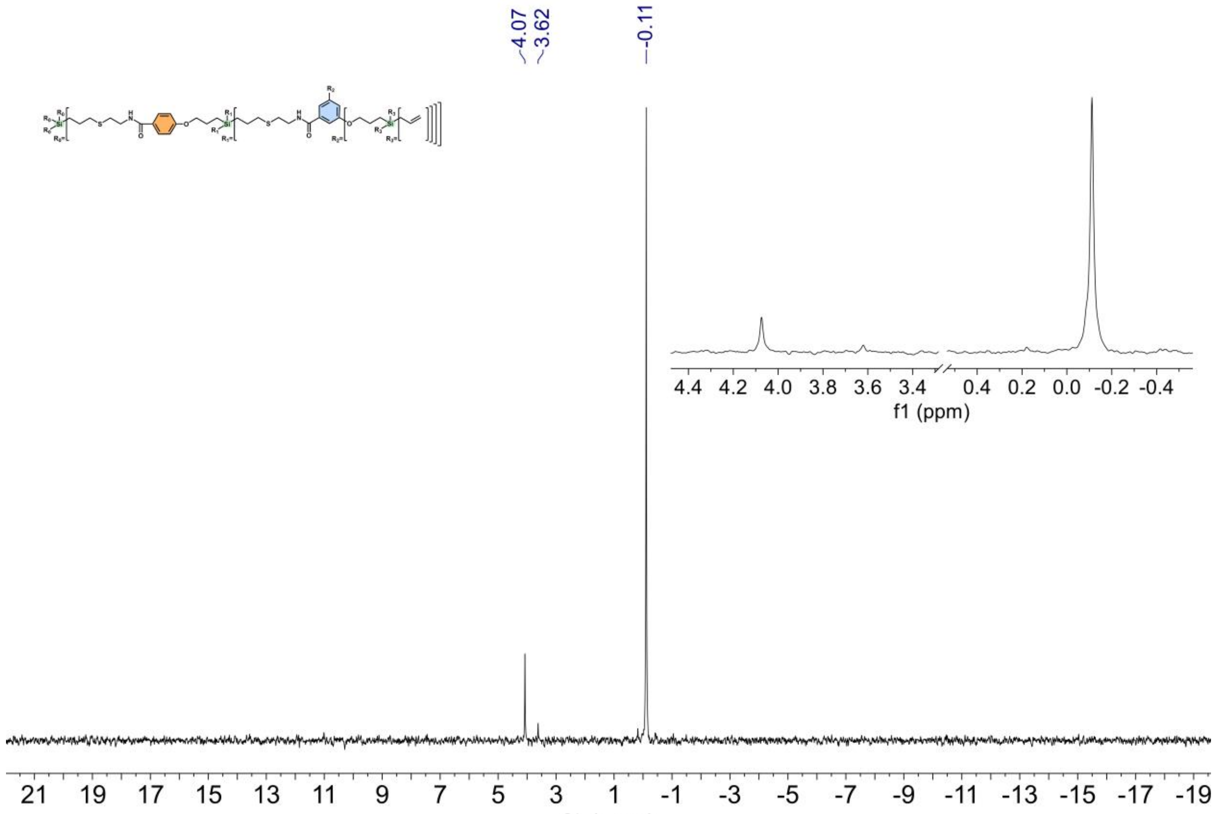

Figure S123: ^{29}Si {^{1}H} NMR (79 MHz, $CDCl_3$) **G_2-3-6-A**

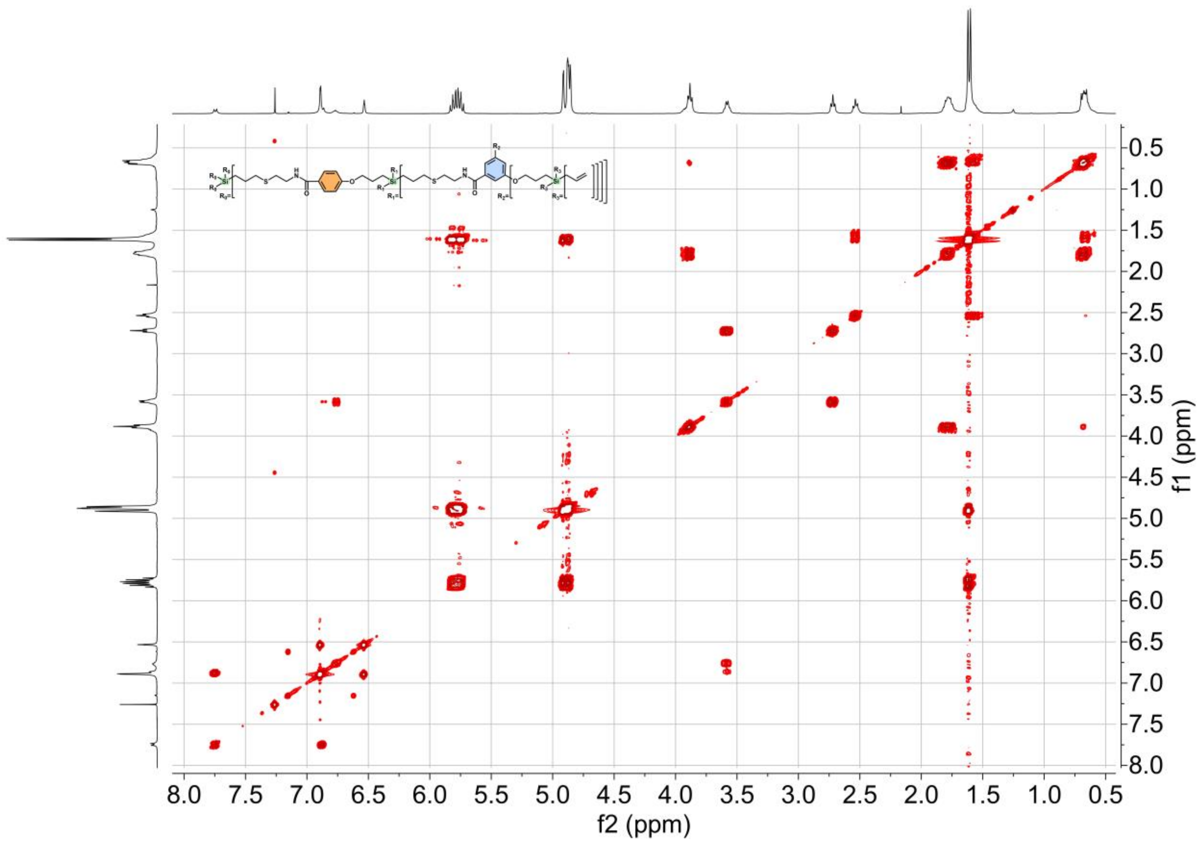

Figure S124: ^{1}H-^{1}H COSY NMR ($CDCl_3$) **G_2-3-6-A**

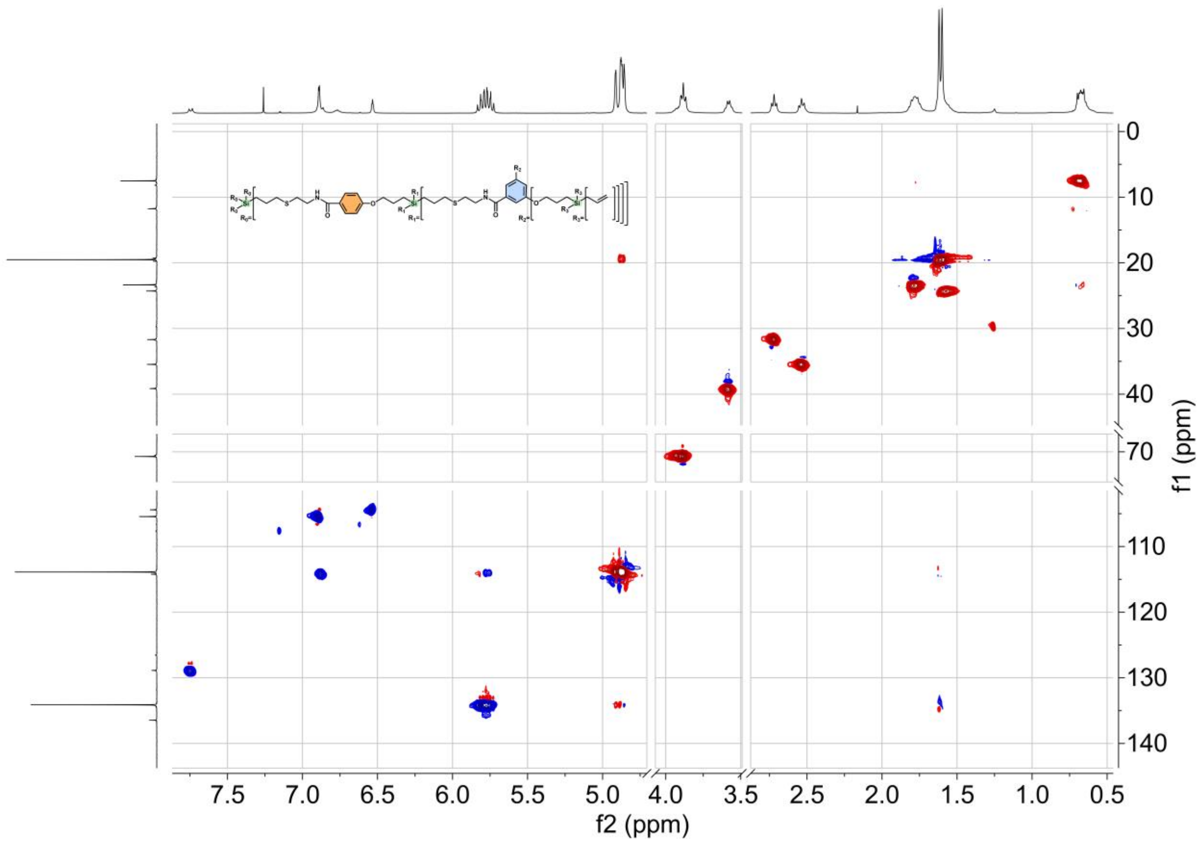

Figure S125: ^{1}H-^{13}C HSQC NMR ($CDCl_3$) **G_2-3-6-A**

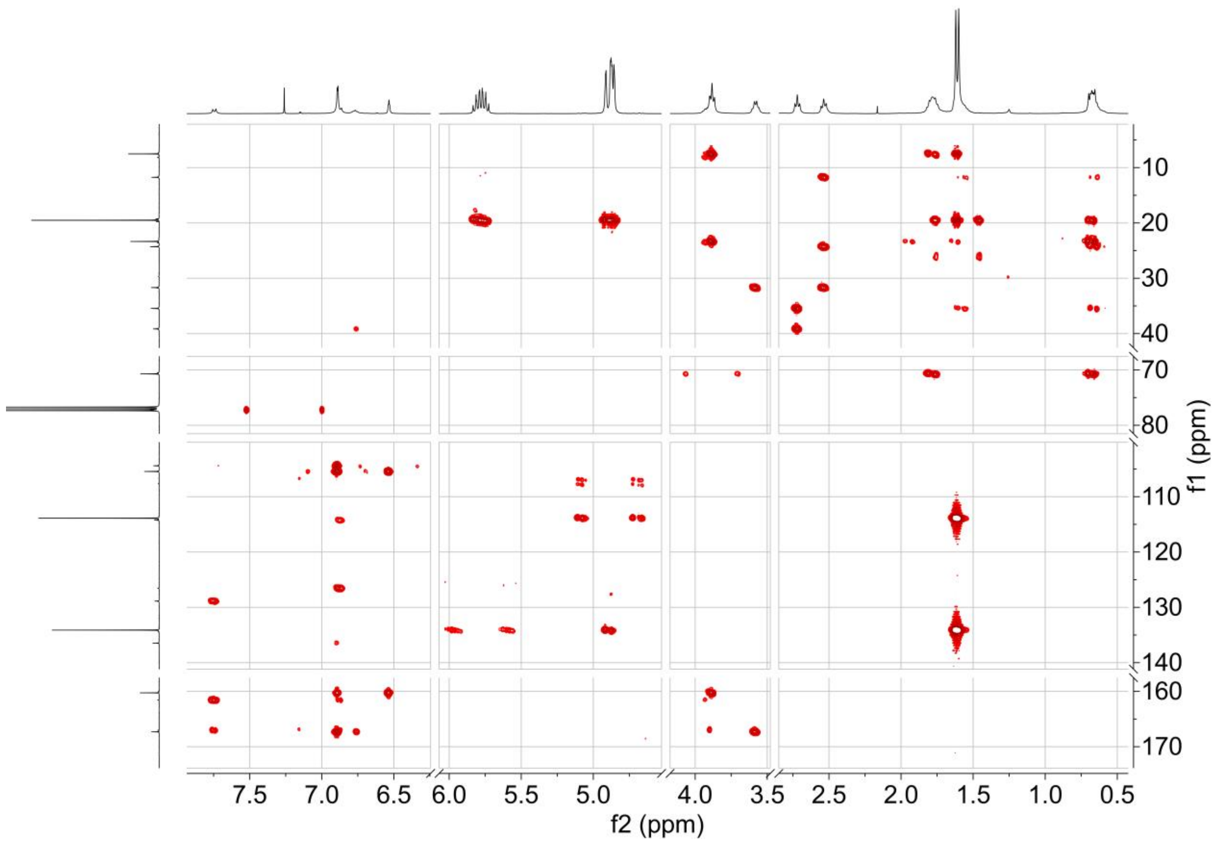

Figure S126: ^{1}H-^{13}C HMBC NMR ($CDCl_3$) **G_2-3-6-A**

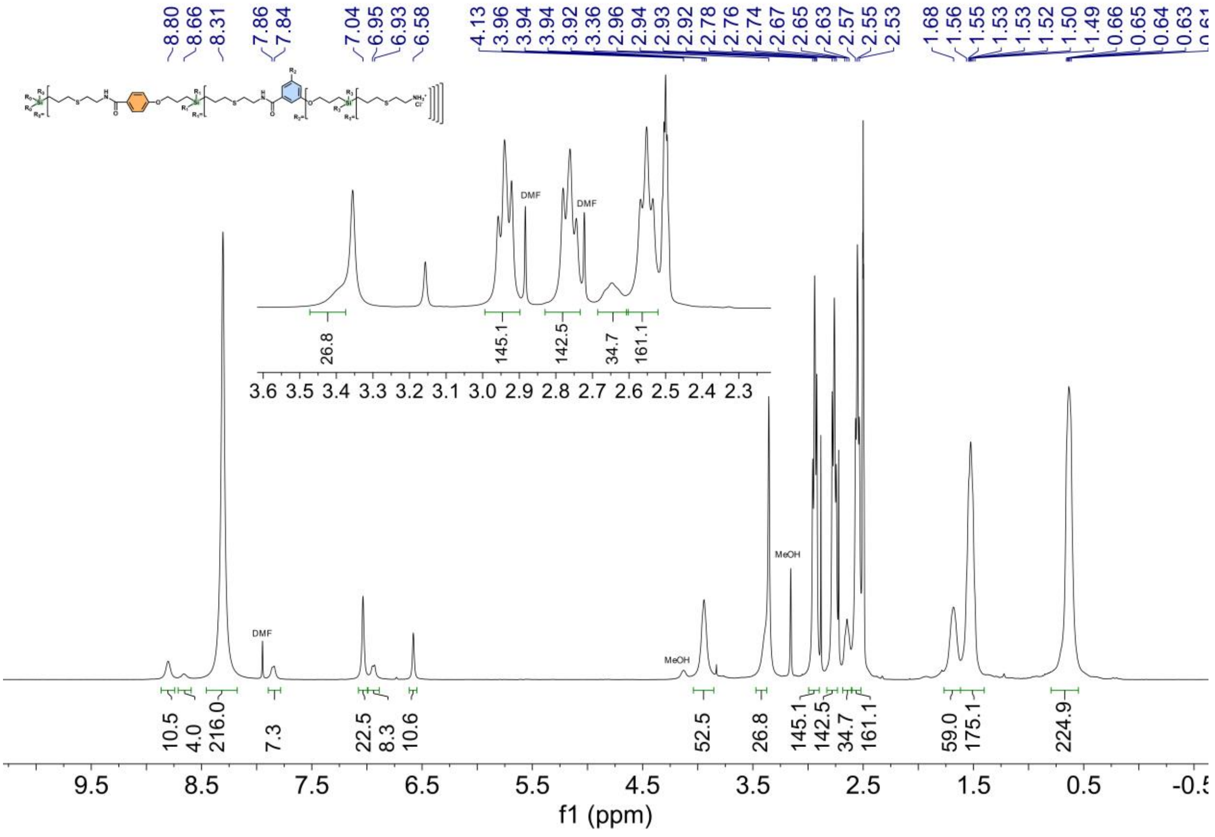

Figure S127: ^{1}H NMR (400 MHz, DMSO-d_6) **G_2-3-6-N**

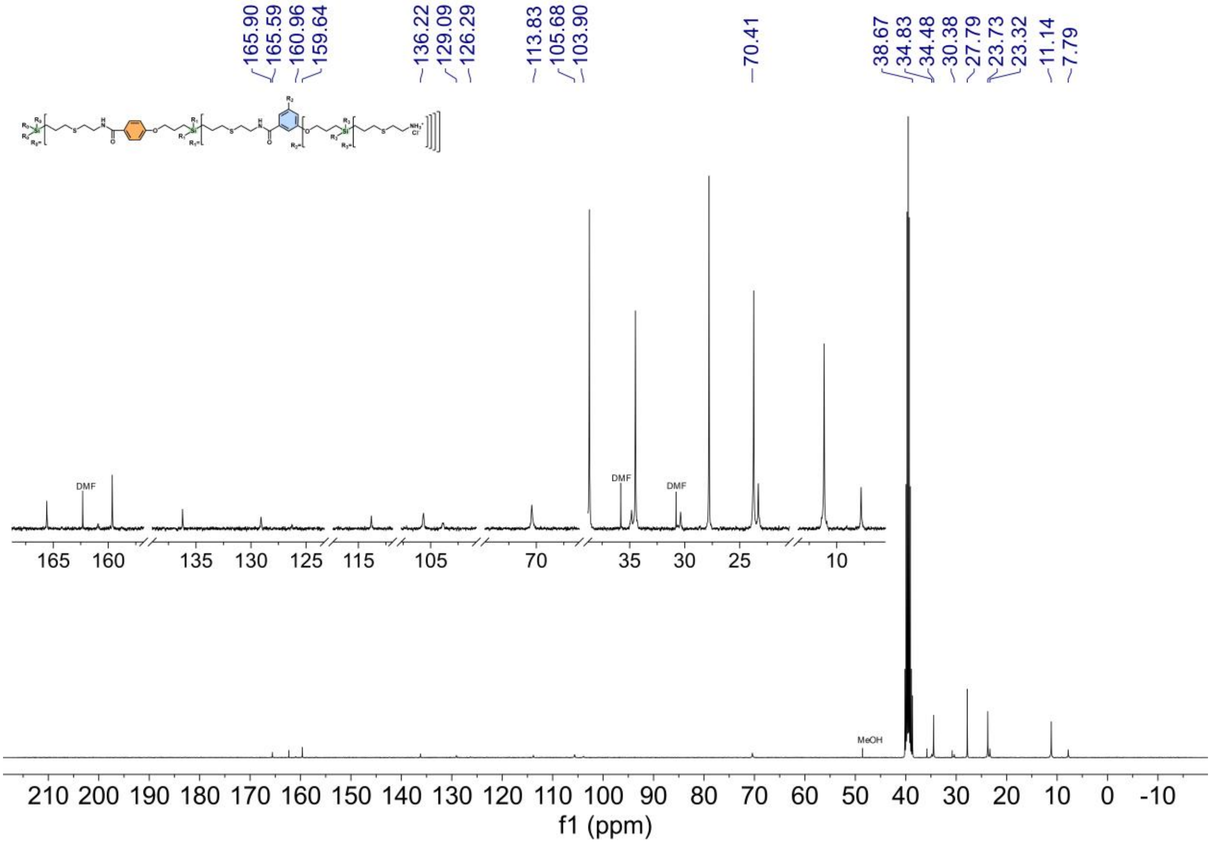

Figure S128: ^{13}C {^{1}H} NMR (101 MHz, DMSO-d_6) **G_2-3-6-N**

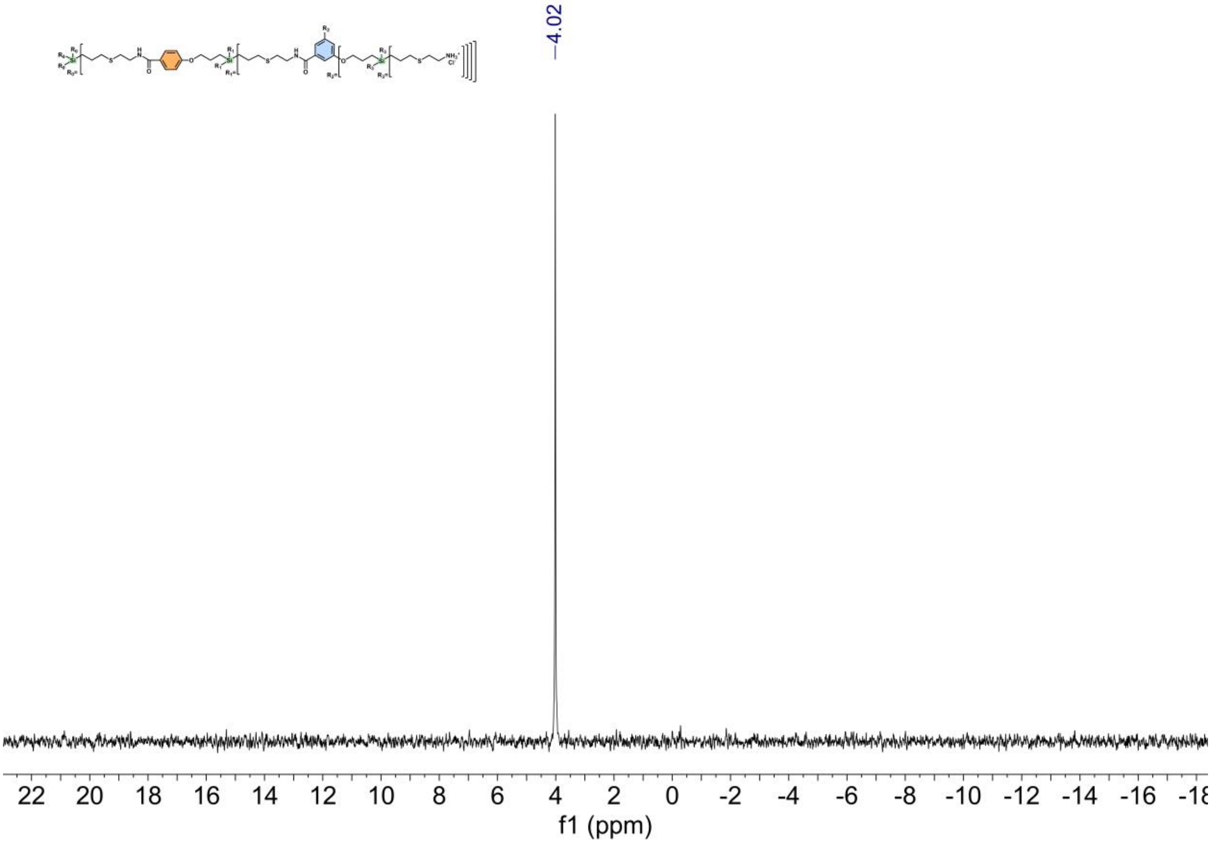

Figure S129: ^{29}Si {^{1}H} NMR (79 MHz, DMSO-d_6) **G_2-3-6-N**

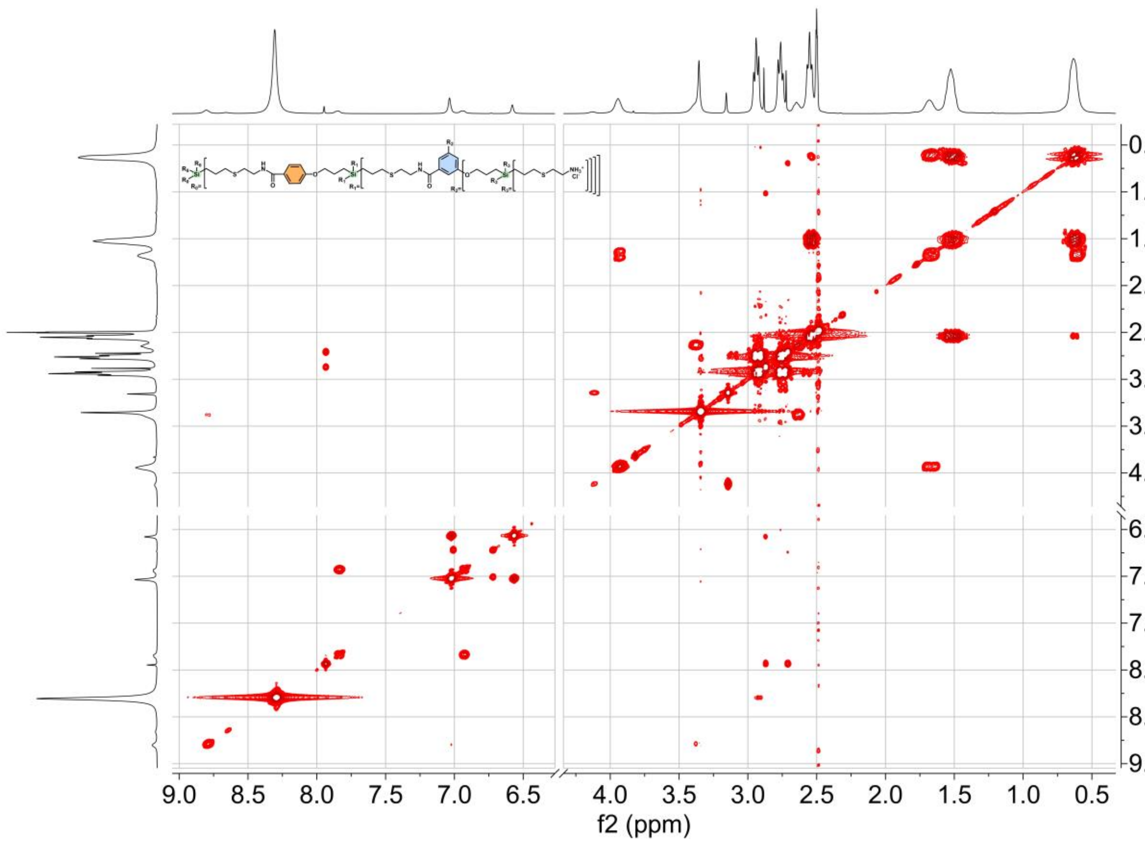

Figure S130: ^{1}H-^{1}H COSY NMR (DMSO-d_6) **G_2-3-6-N**

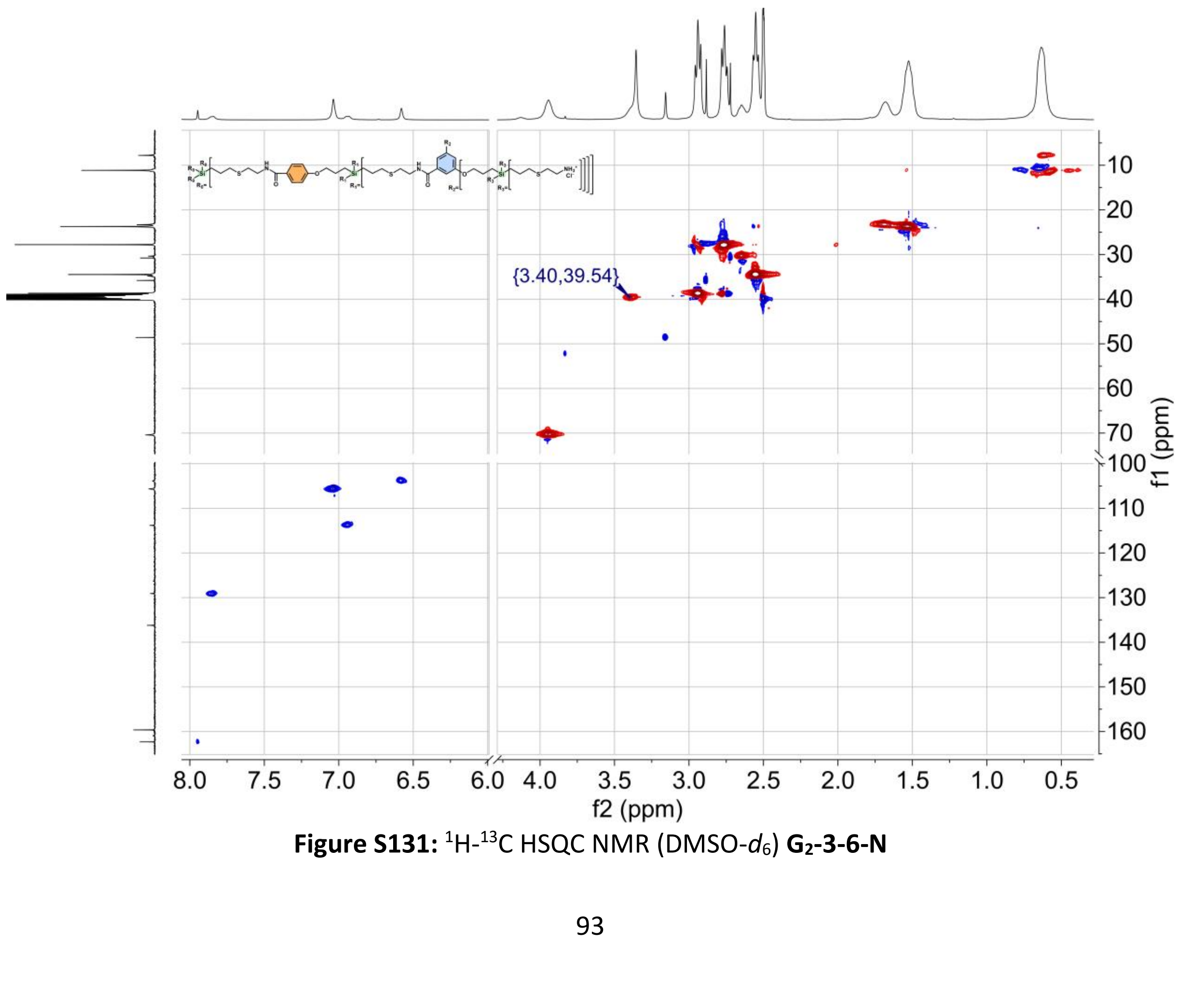

Figure S131: ^{1}H-^{13}C HSQC NMR (DMSO-d_6) **G_2-3-6-N**

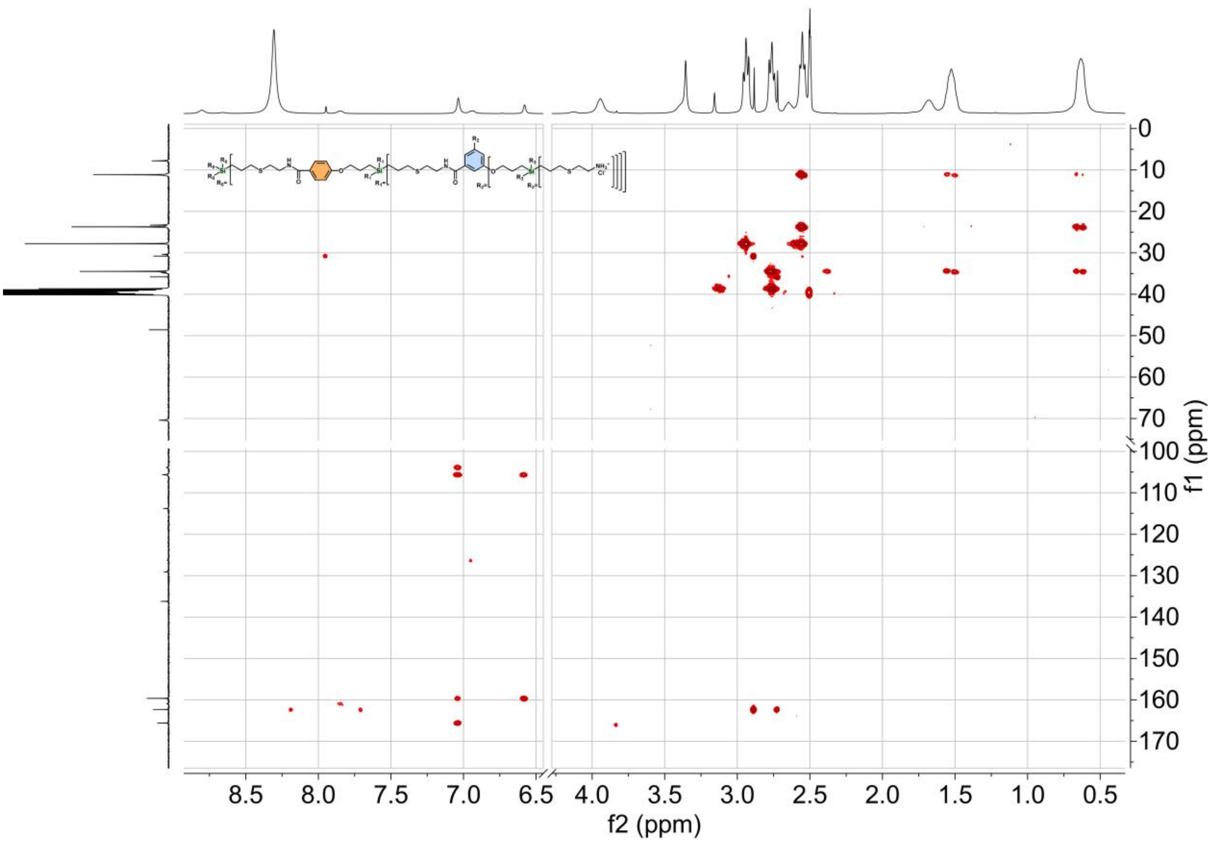

Figure S132: ^{1}H-^{13}C HMBC NMR (DMSO-d_6) **G_2-3-6-N**

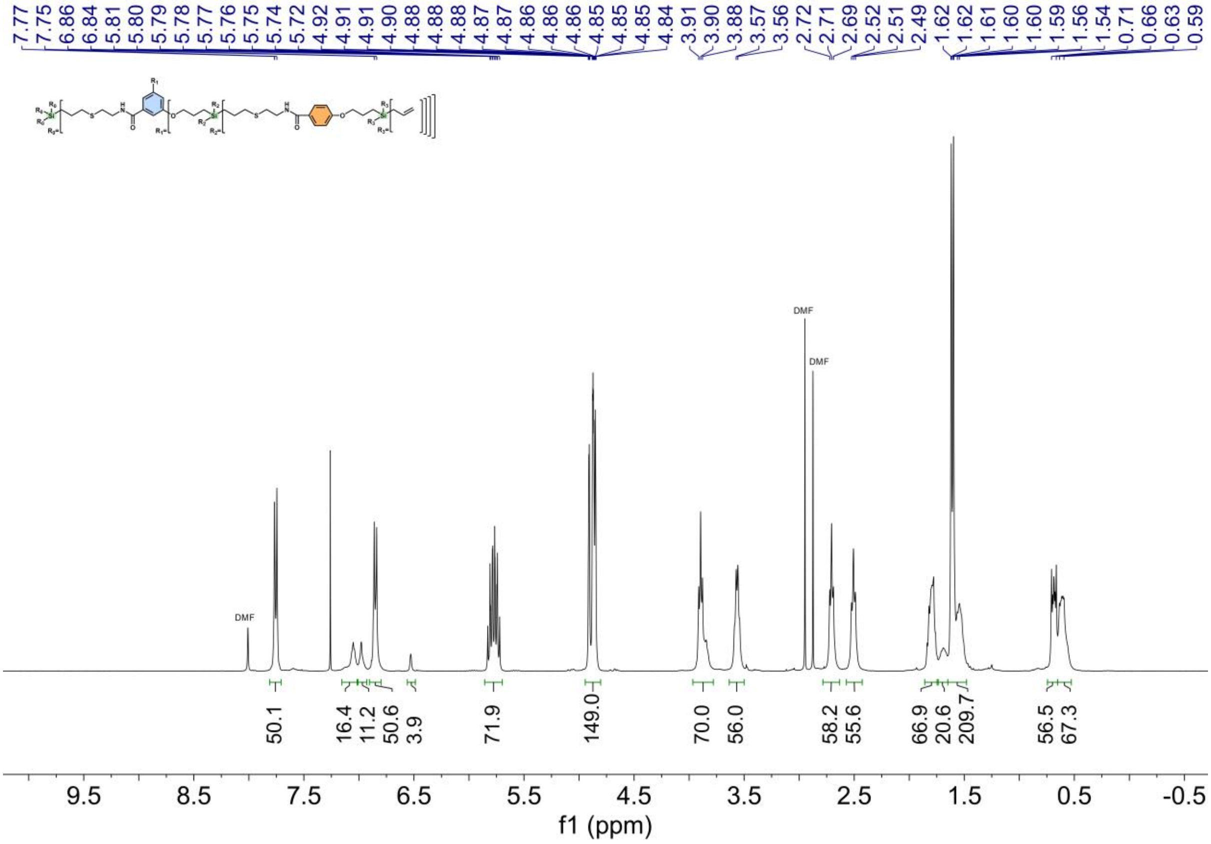

Figure S134: ^{1}H NMR (400 MHz, $CDCl_3$) **G_2-6-3-A**

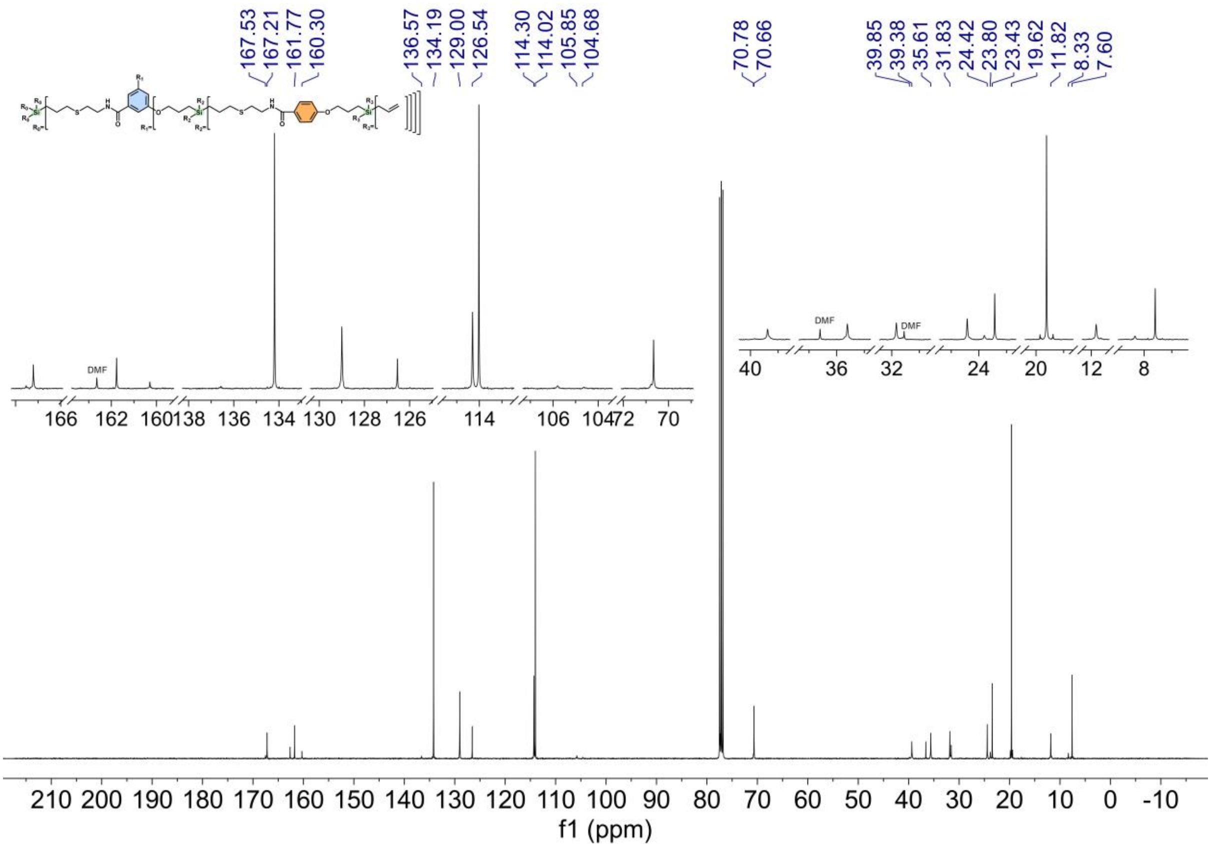

Figure S135: ^{13}C {^{1}H} NMR (101 MHz, $CDCl_3$) **G_2-6-3-A**

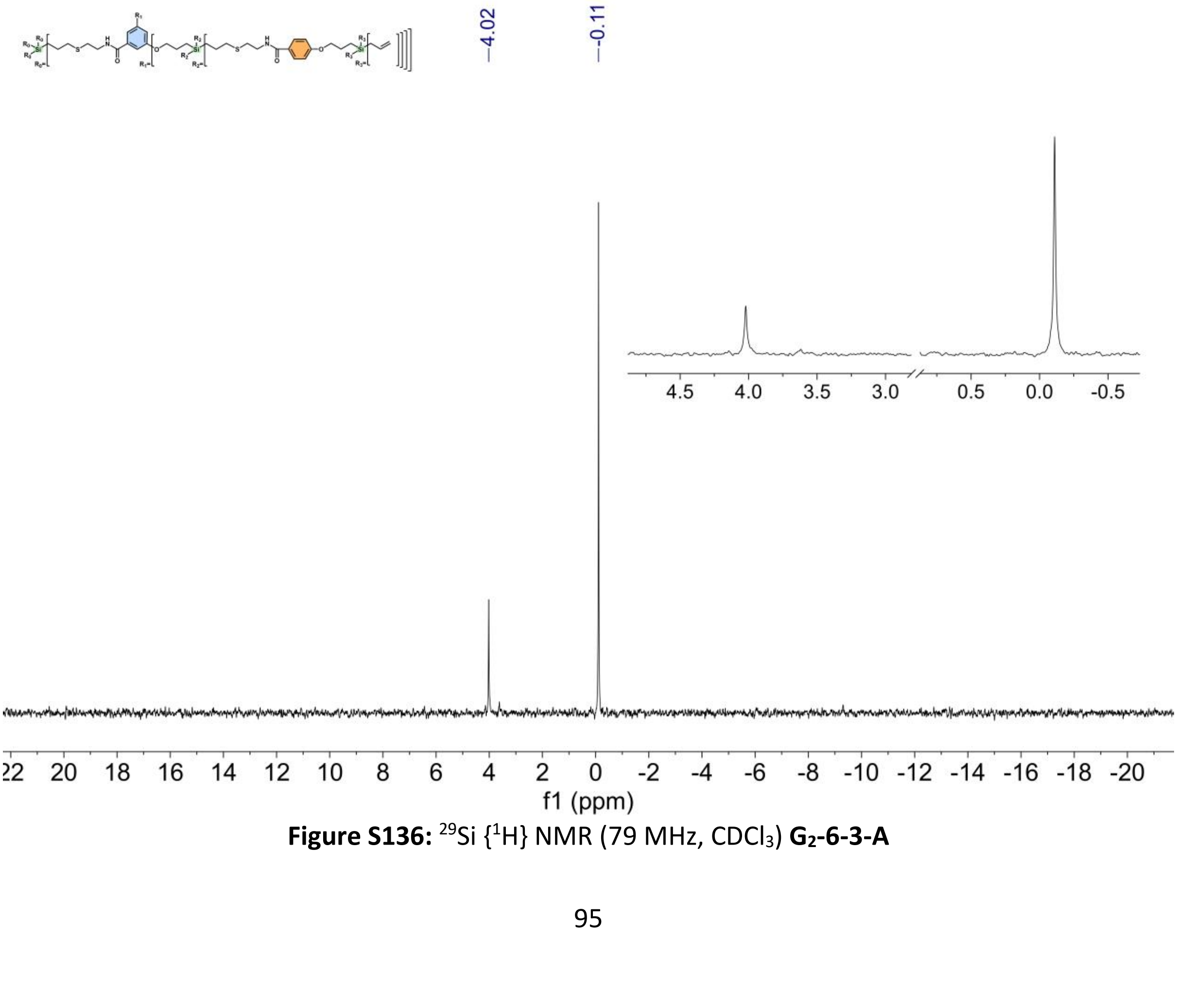

Figure S136: ^{29}Si {^{1}H} NMR (79 MHz, $CDCl_3$) **G_2-6-3-A**

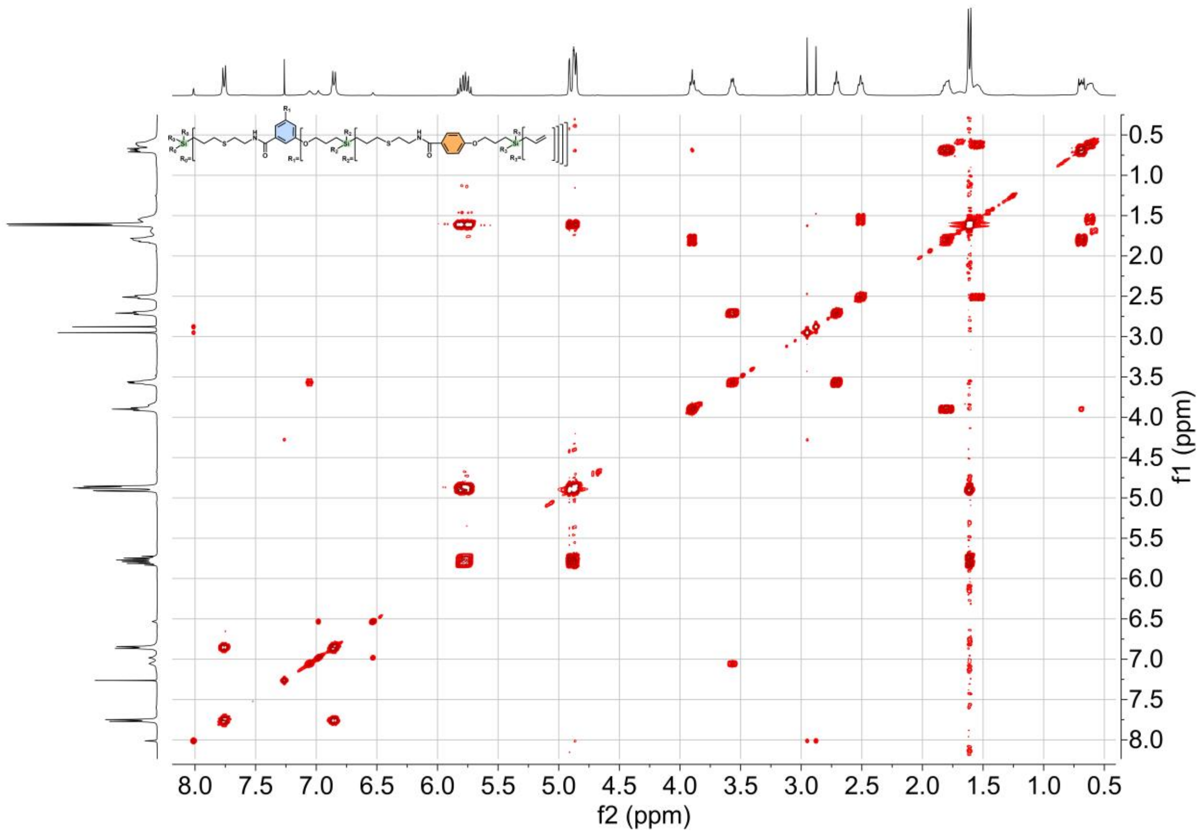

Figure S137: ^{1}H-^{1}H COSY NMR ($CDCl_3$) **G_2-6-3-A**

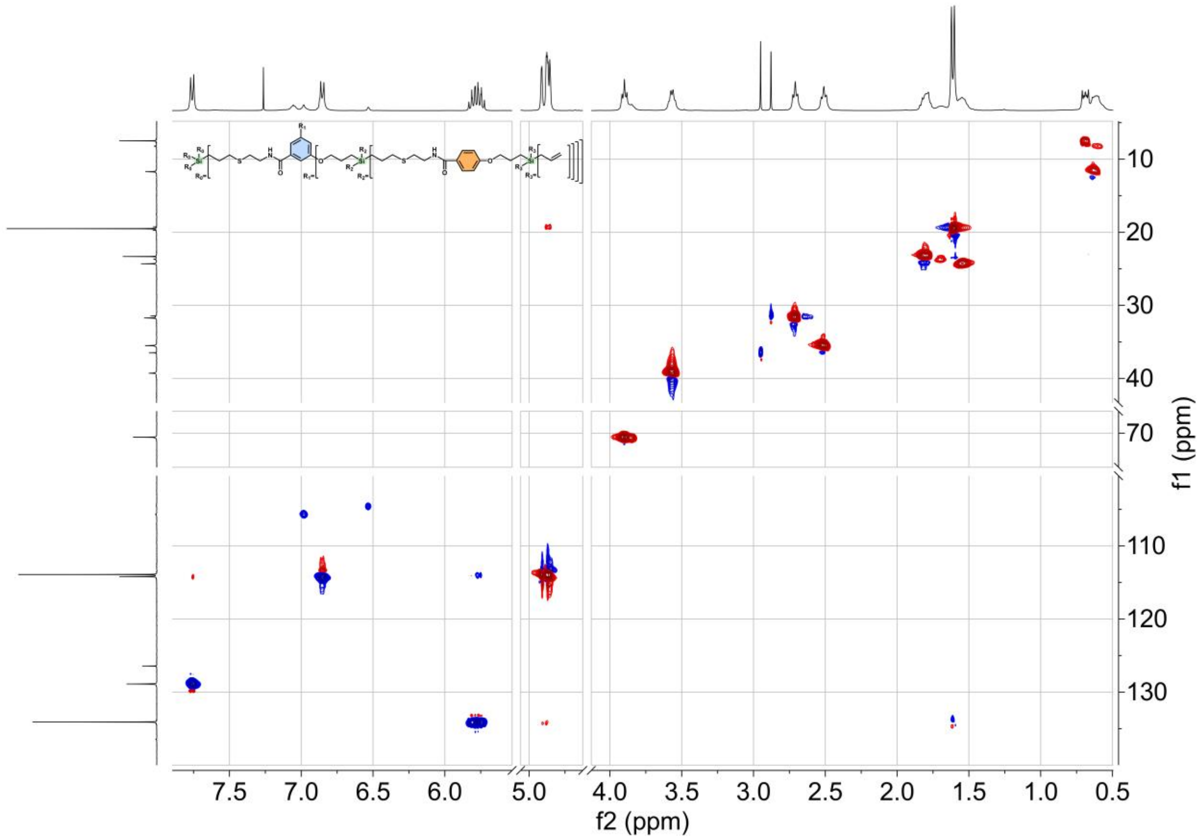

Figure S138: ^{1}H-^{13}C HSQC NMR ($CDCl_3$) **G_2-6-3-A**

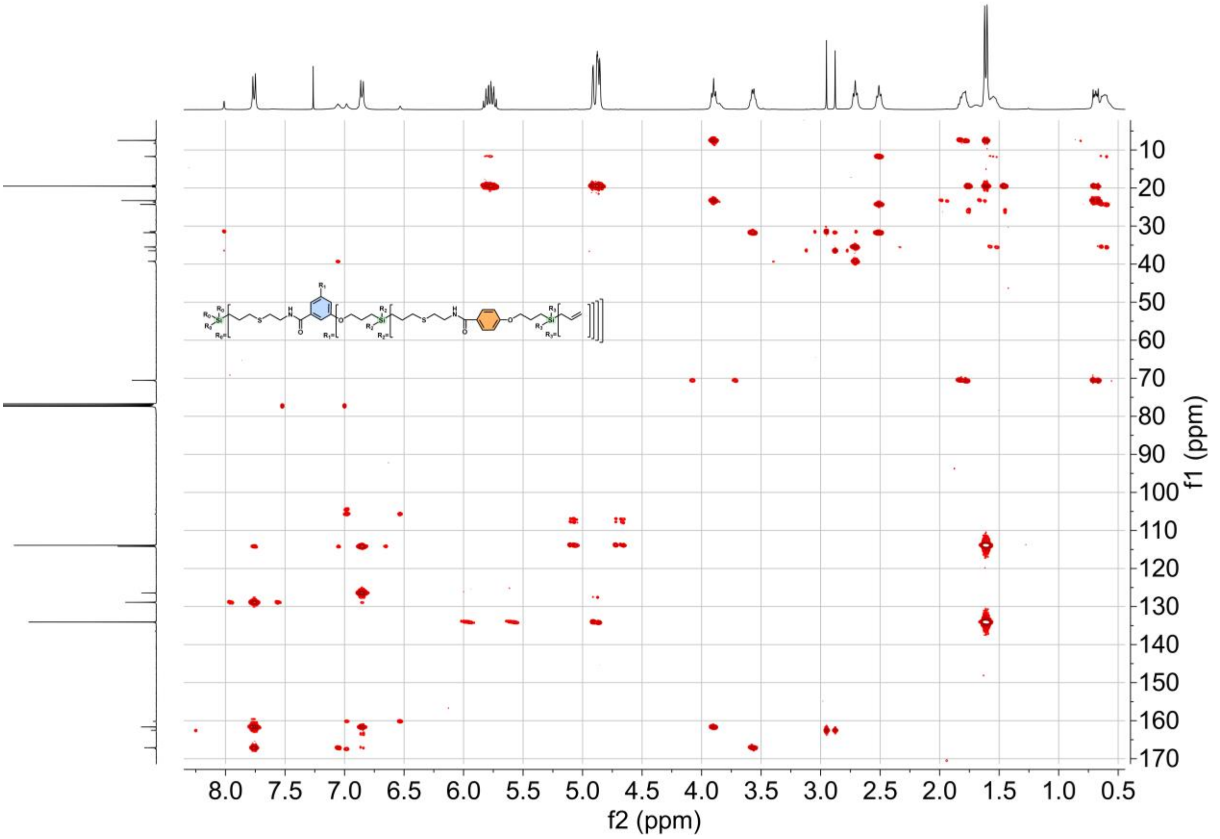

Figure S139: ^1H-^{13}C HMBC NMR ($CDCl_3$) **G_2-6-3-A**

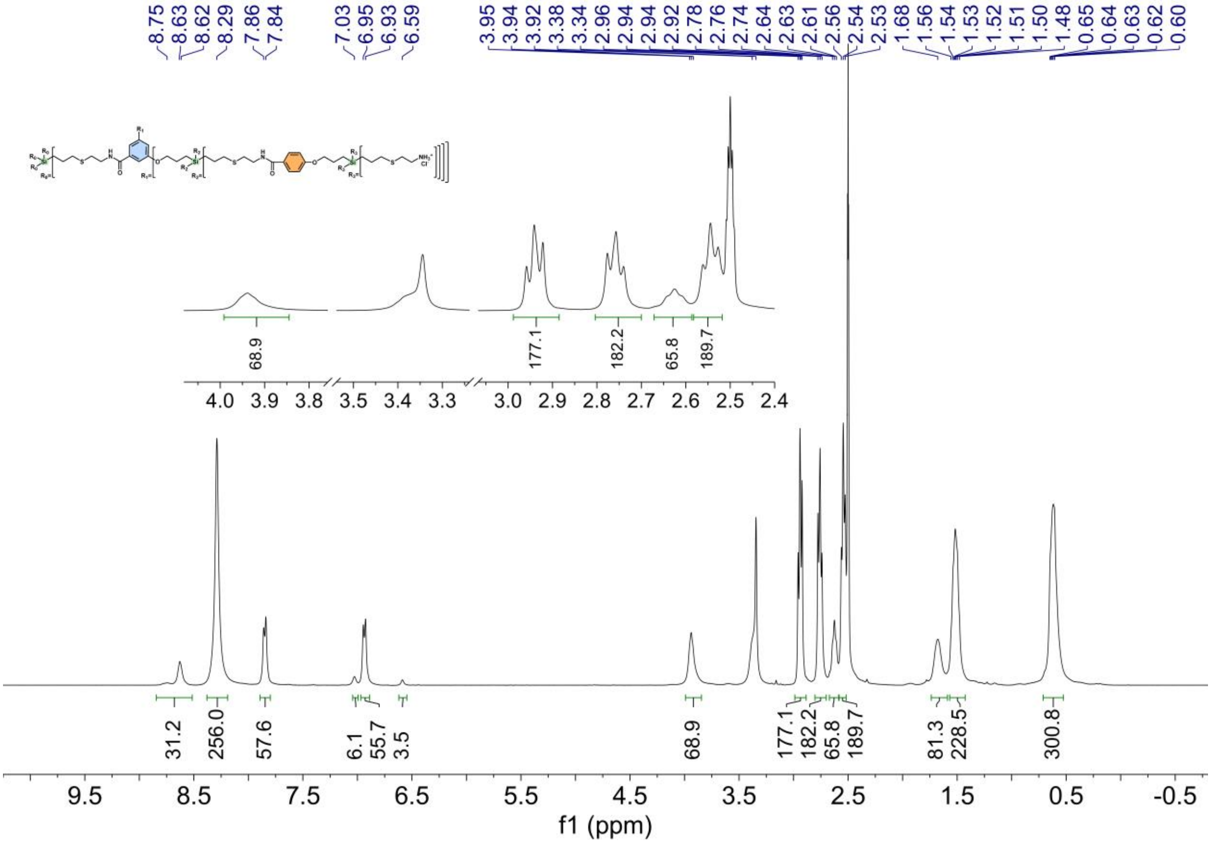

Figure S140: ^1H NMR (400 MHz, DMSO-d_6) **G_2-6-3-N**

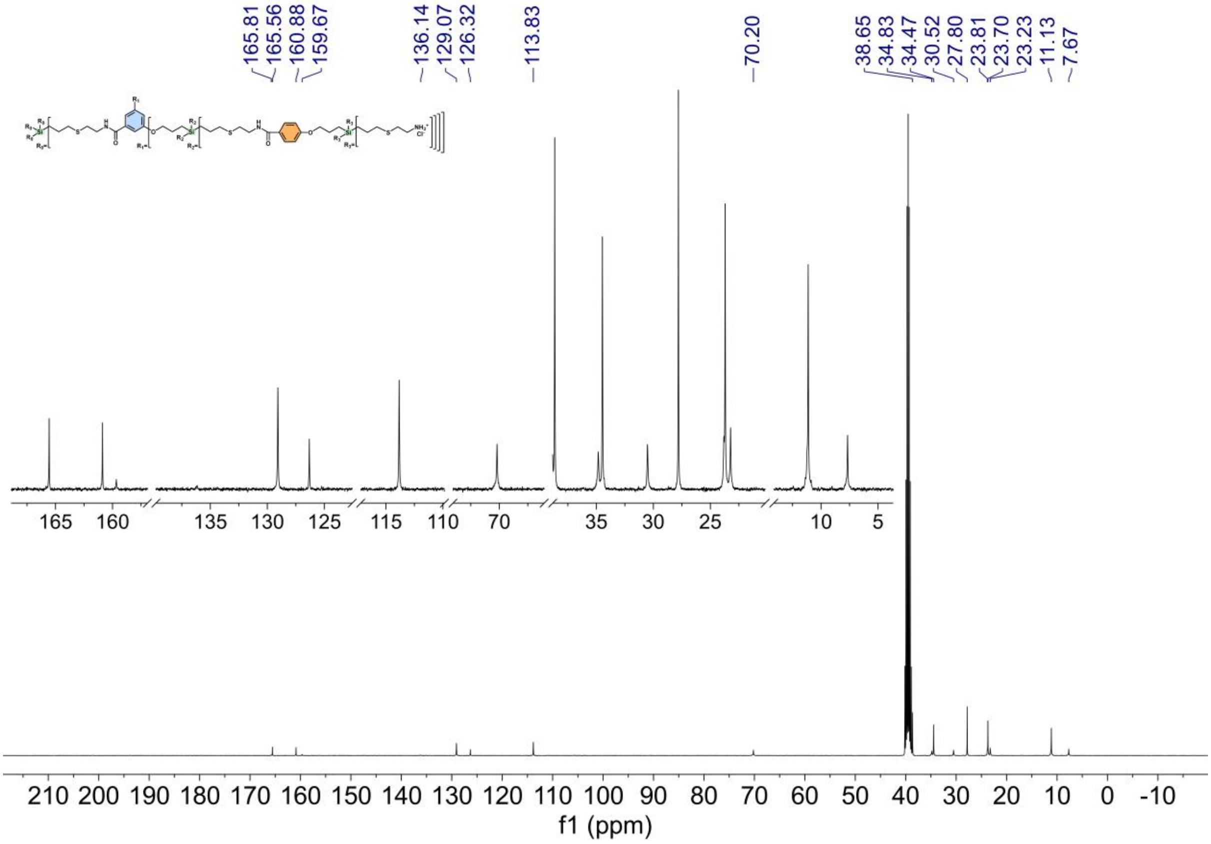

Figure S141: ^{13}C {^{1}H} NMR (101 MHz, DMSO-d_6) **G_2-6-3-N**

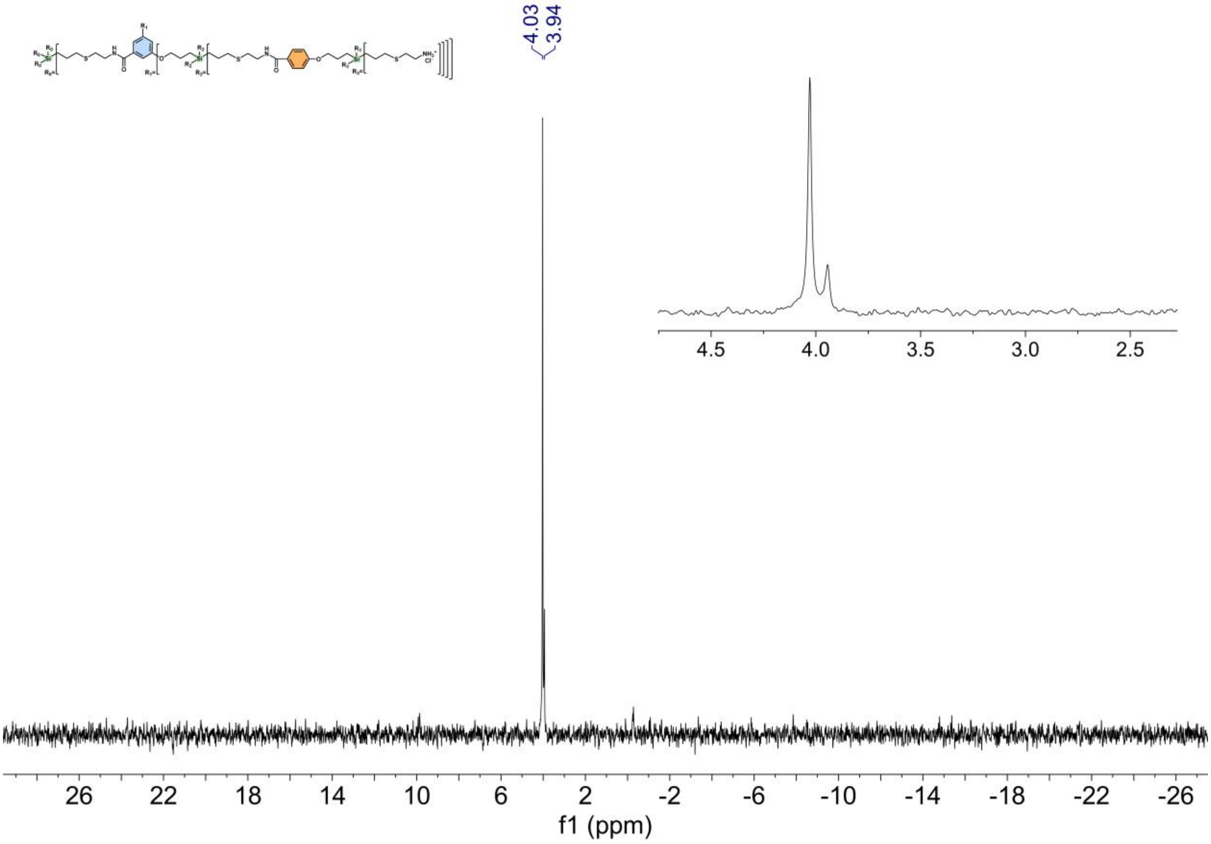

Figure S142: ^{29}Si {^{1}H} NMR (79 MHz, DMSO-d_6) **G_2-6-3-N**

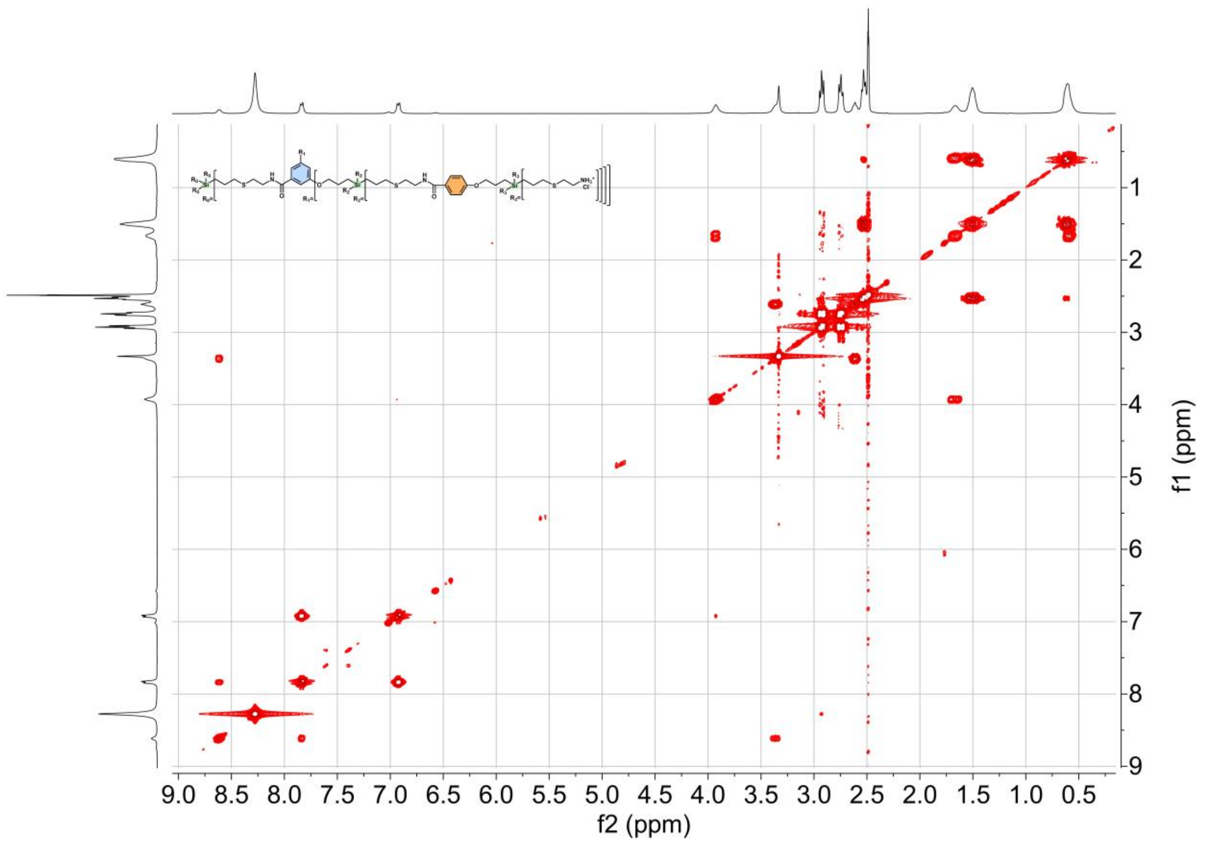

Figure S143: ^{1}H-^{1}H COSY NMR (DMSO-d_6) **G_2-6-3-N**

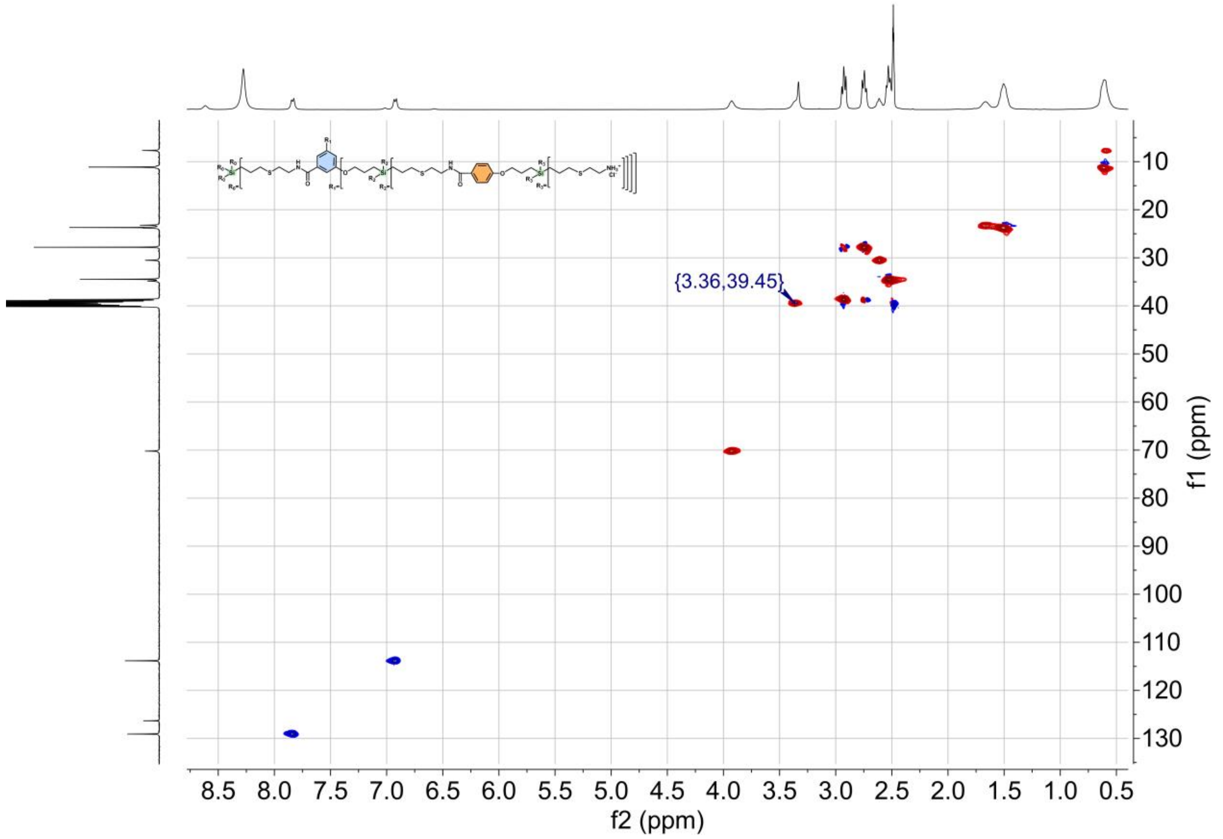

Figure S144: ^{1}H-^{13}C HSQC NMR (DMSO-d_6) **G_2-6-3-N**

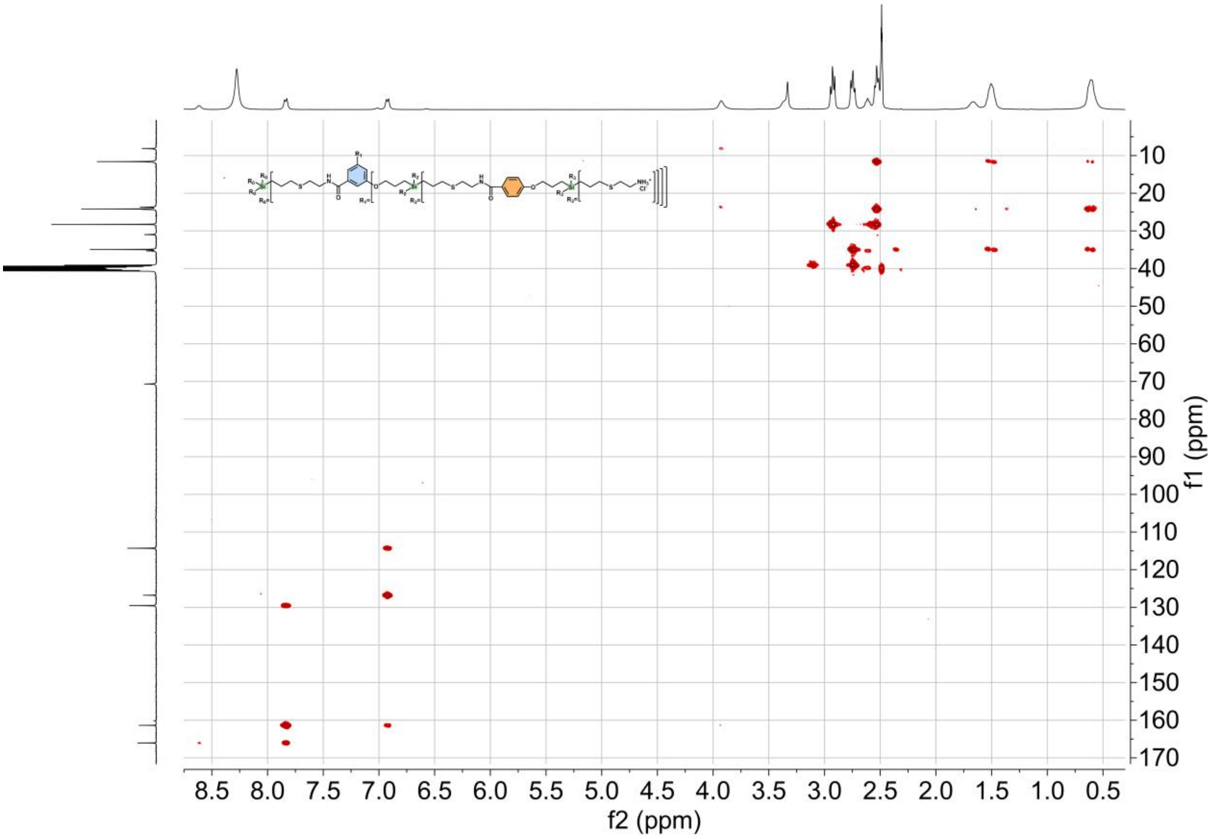

Figure S145: ^1H-^{13}C HMBC NMR (DMSO-d_6) **G_2-6-3-N**

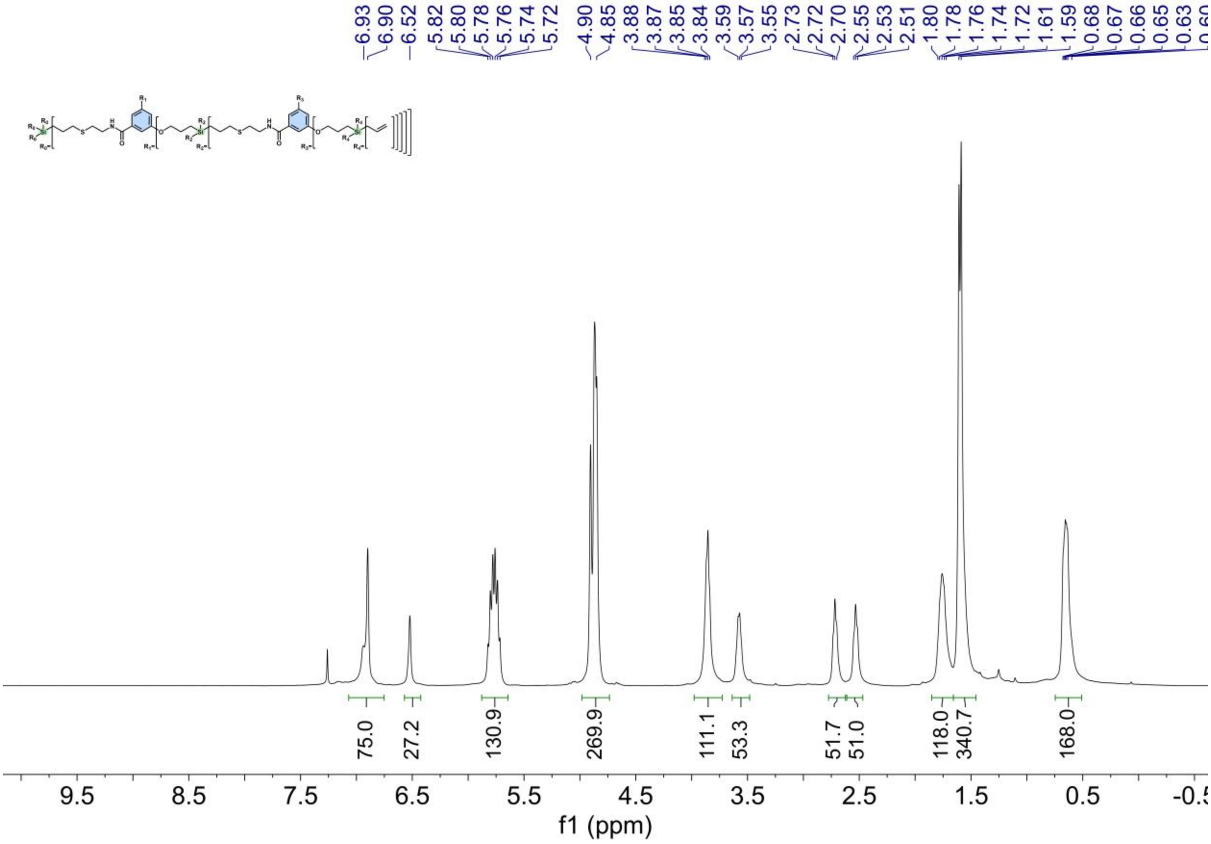

Figure S146: ^1H NMR (400 MHz, $CDCl_3$) **G_2-6-6-A**

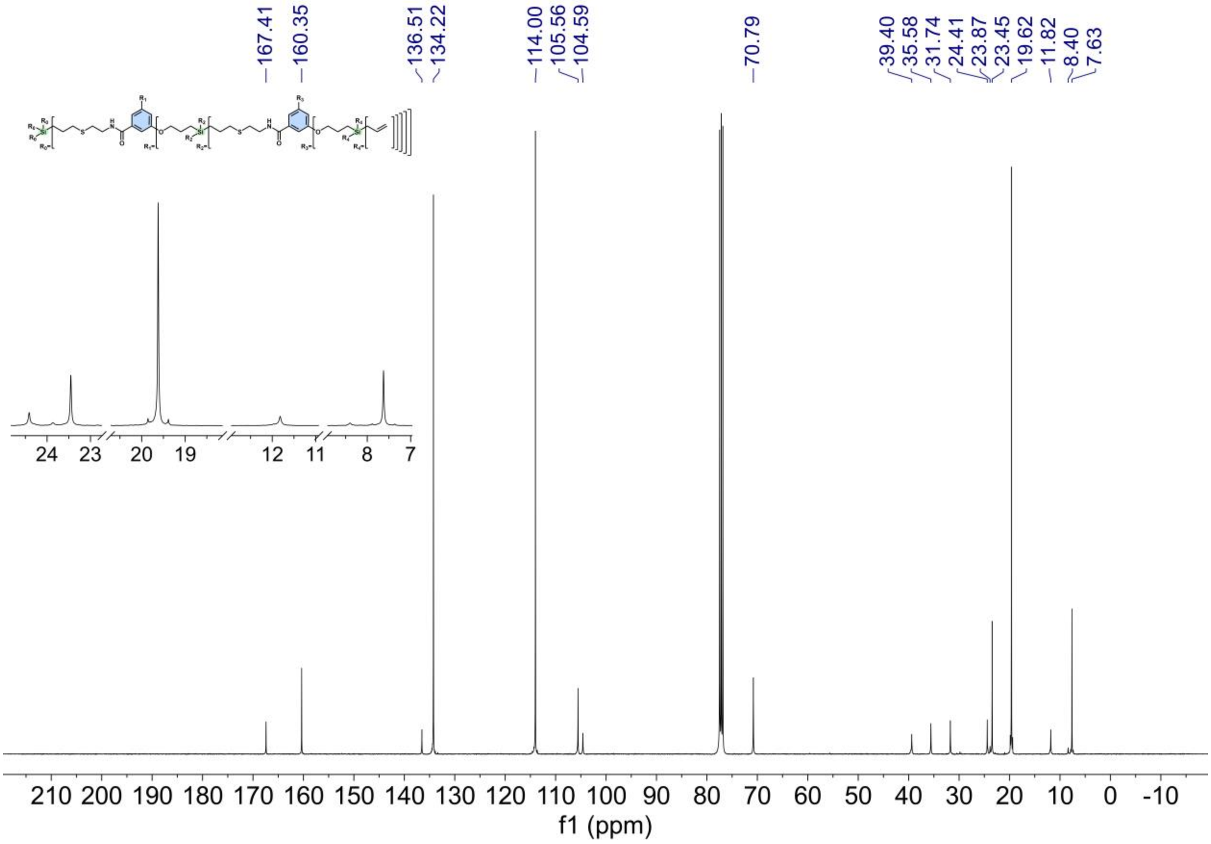

Figure S147: $^{13}C\ \{^1H\}$ NMR (101 MHz, $CDCl_3$) **G_2-6-6-A**

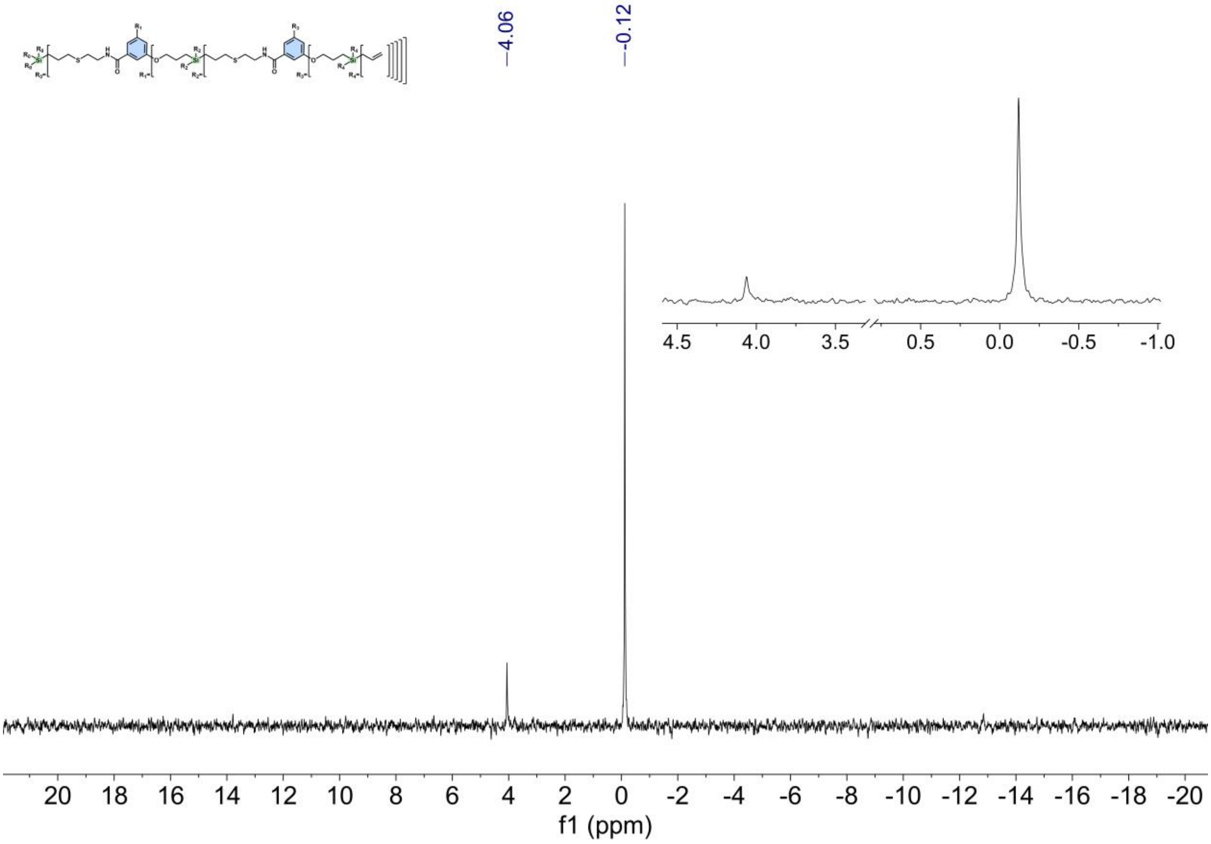

Figure S148: $^{29}Si\ \{^1H\}$ NMR (79 MHz, $CDCl_3$) **G_2-6-6-A**

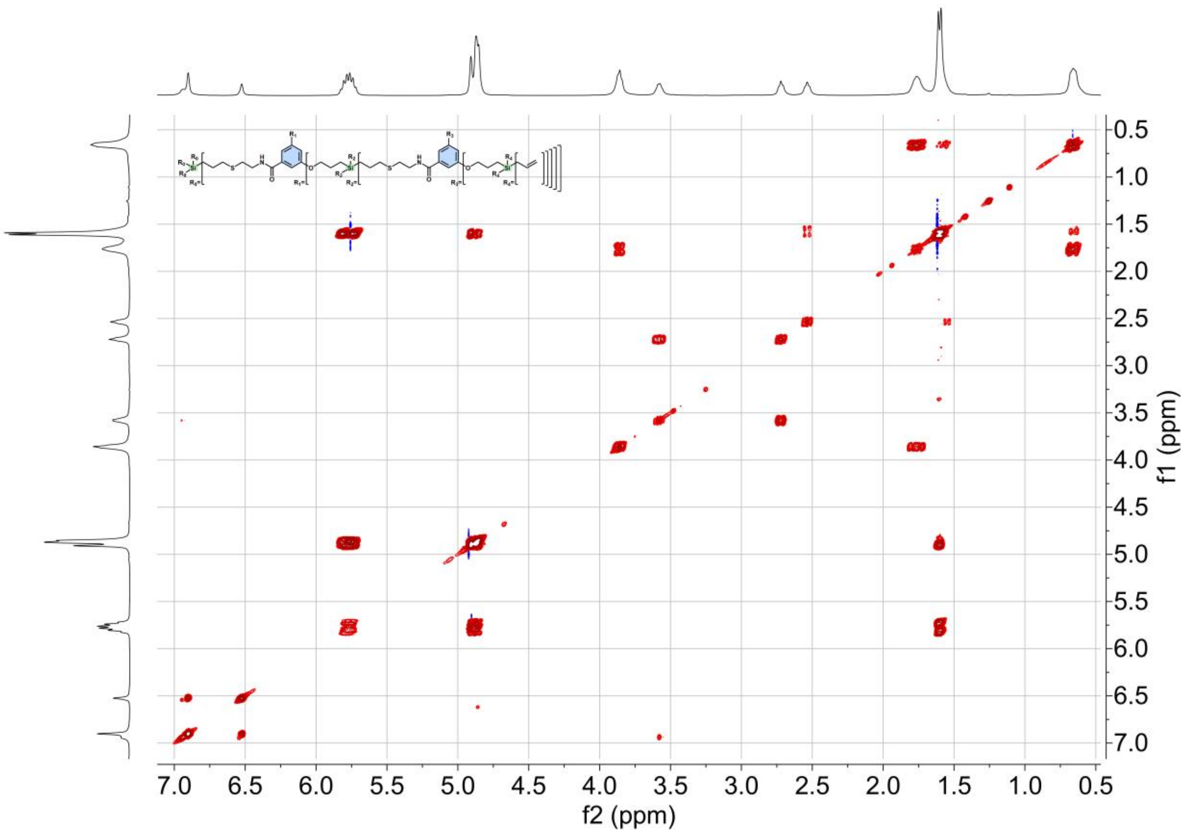

Figure S149: ^{1}H-^{1}H COSY NMR ($CDCl_3$) **G_2-6-6-A**

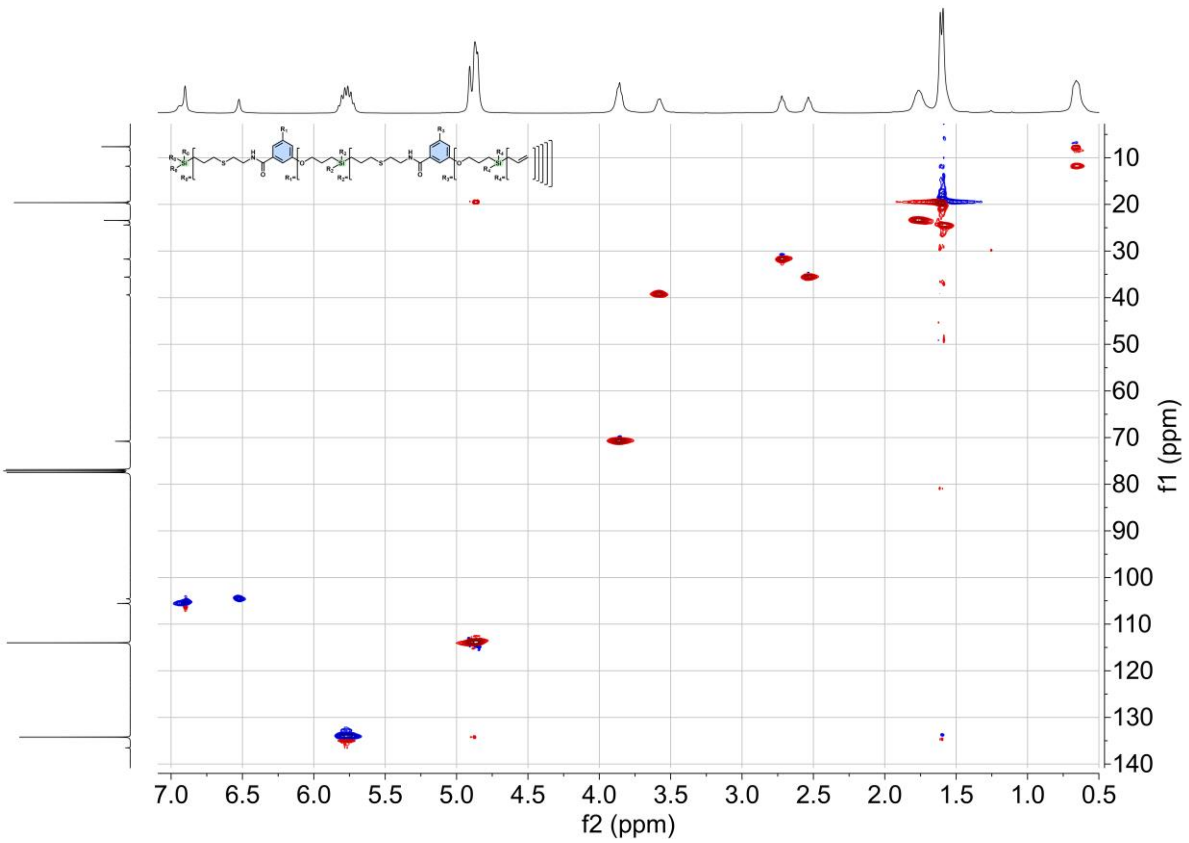

Figure S150: ^{1}H-^{13}C HSQC NMR ($CDCl_3$) **G_2-6-6-A**

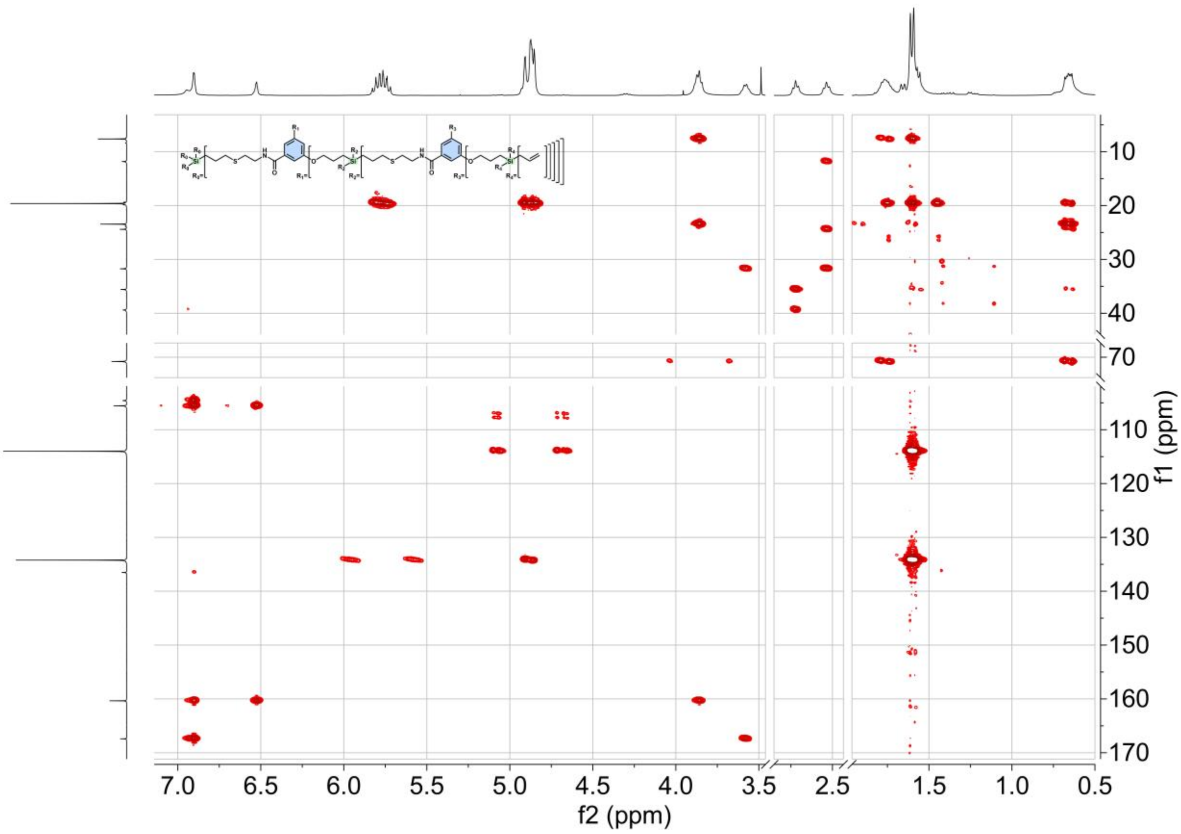

Figure S151: ^{1}H-^{13}C HMBC NMR ($CDCl_3$) **G_2-6-6-A**

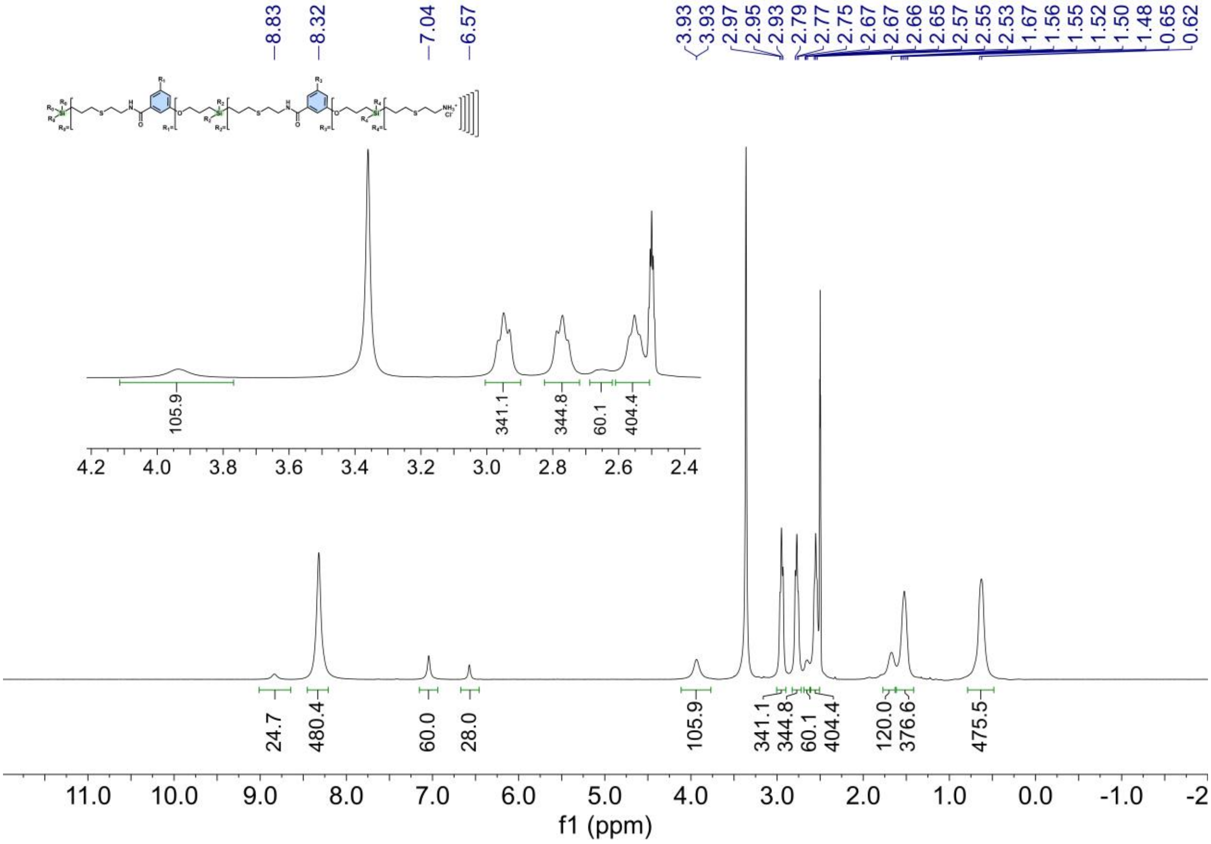

Figure S152: ^{1}H NMR (400 MHz, DMSO-d_6) **G_2-6-6-N**

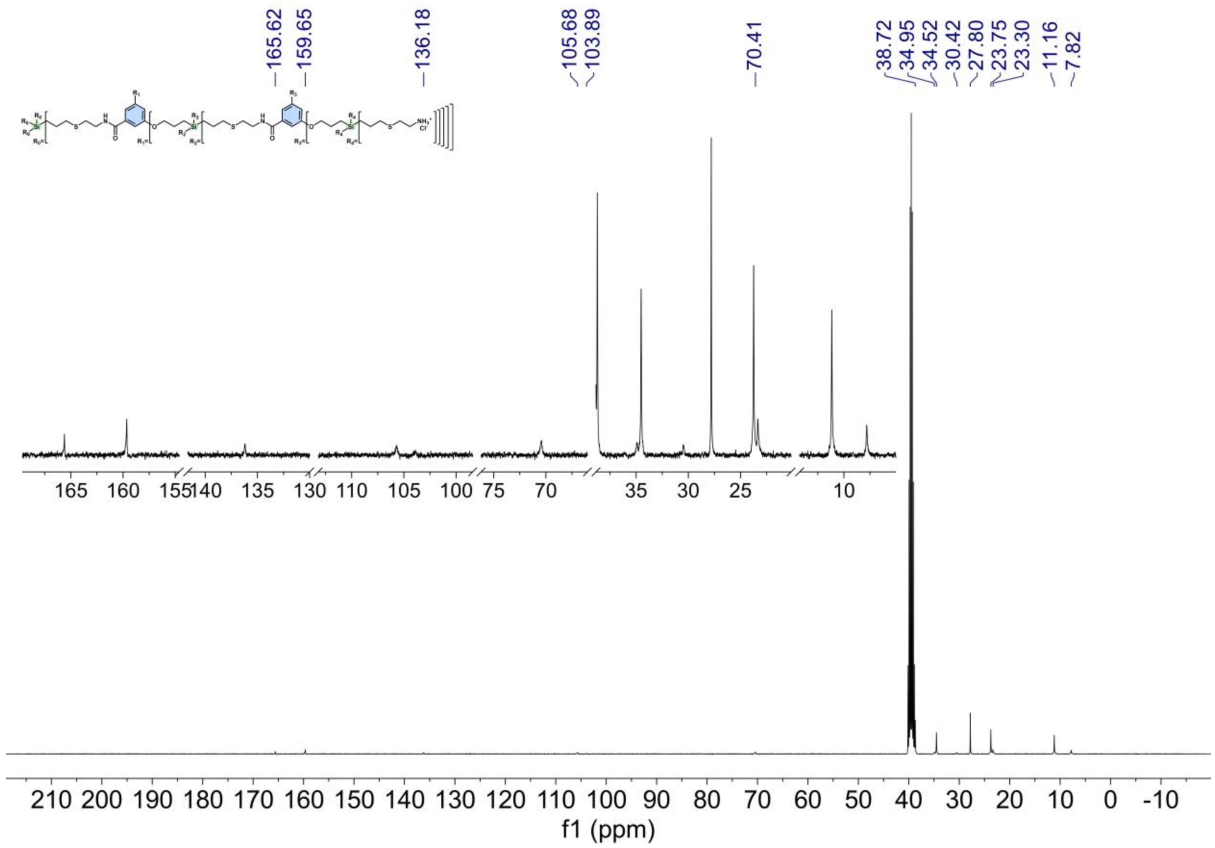

Figure S153 ^{13}C {^{1}H} NMR (101 MHz, DMSO-d_6) **G_2-6-6-N**

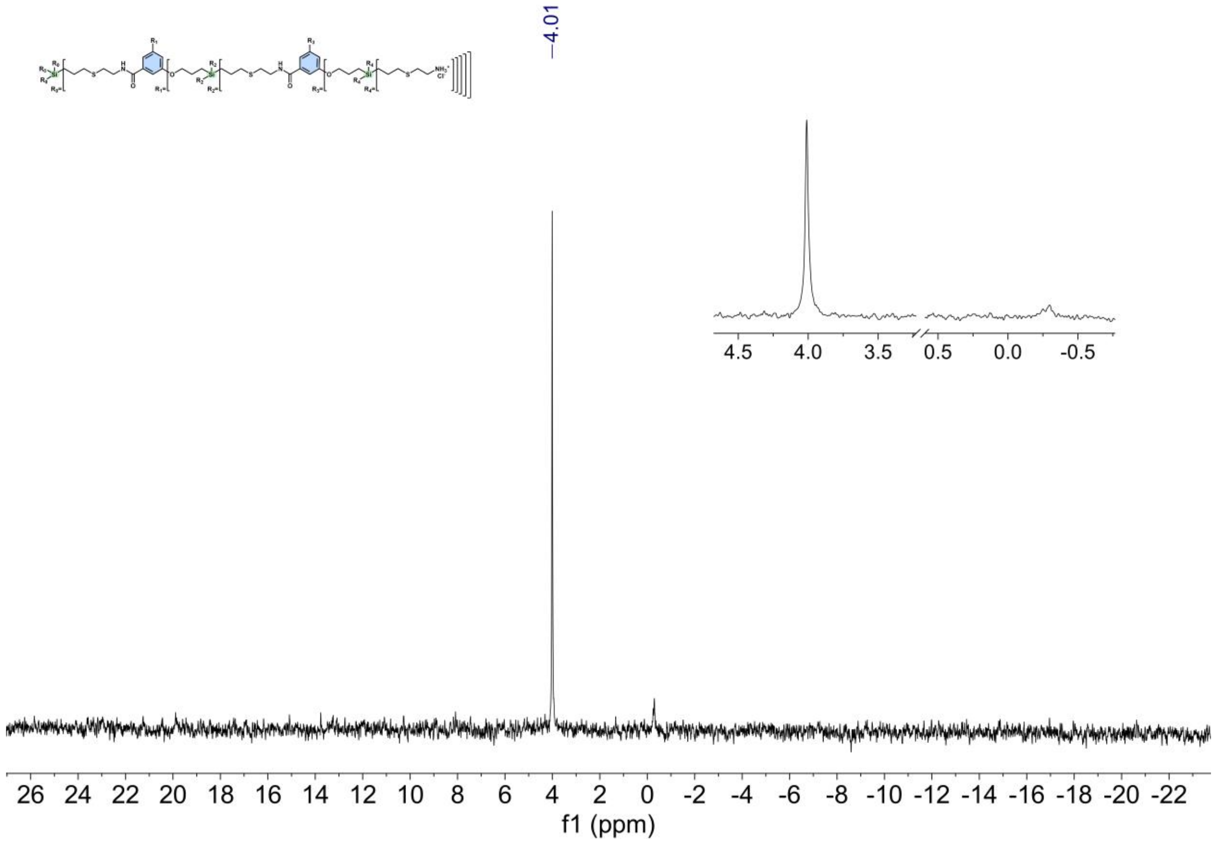

Figure S154: ^{29}Si {^{1}H} NMR (79 MHz, DMSO-d_6) **G_2-6-6-N**

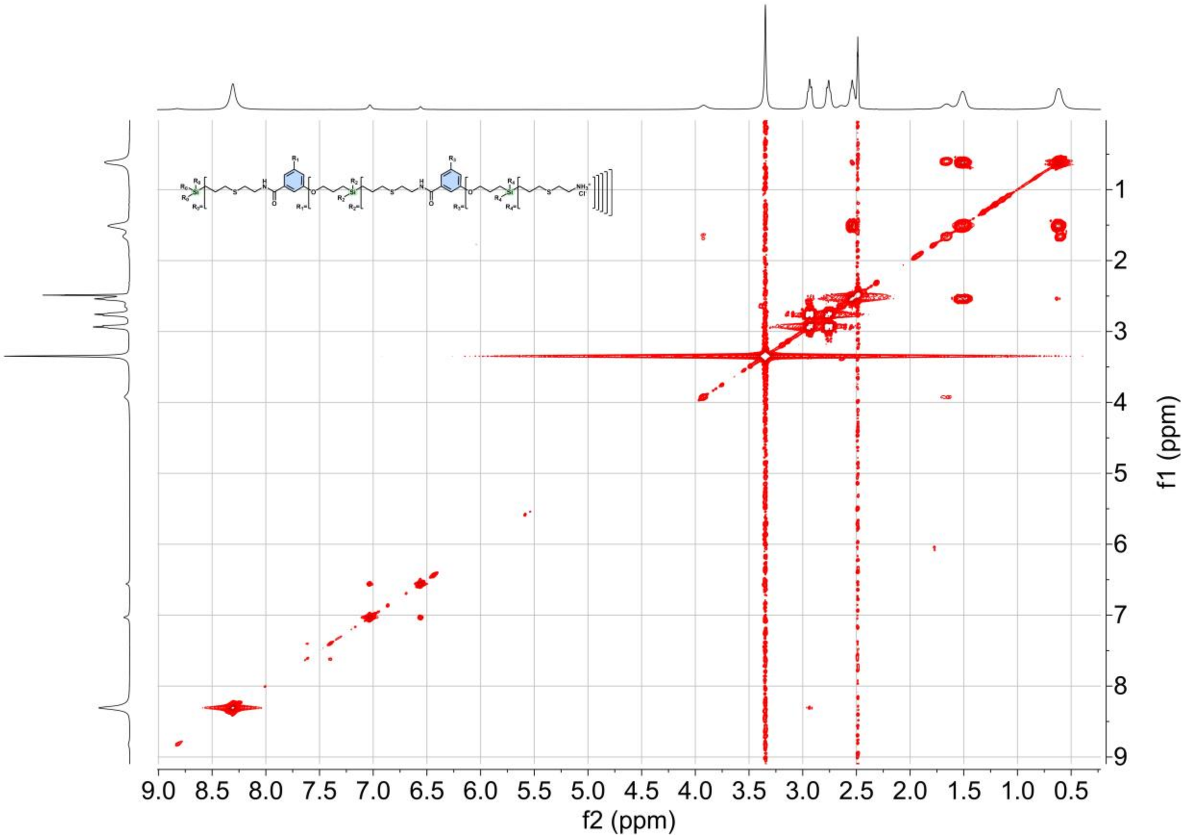

Figure S155: ^{1}H-^{1}H COSY NMR (DMSO-d_6) **G$_2$-6-6-N**

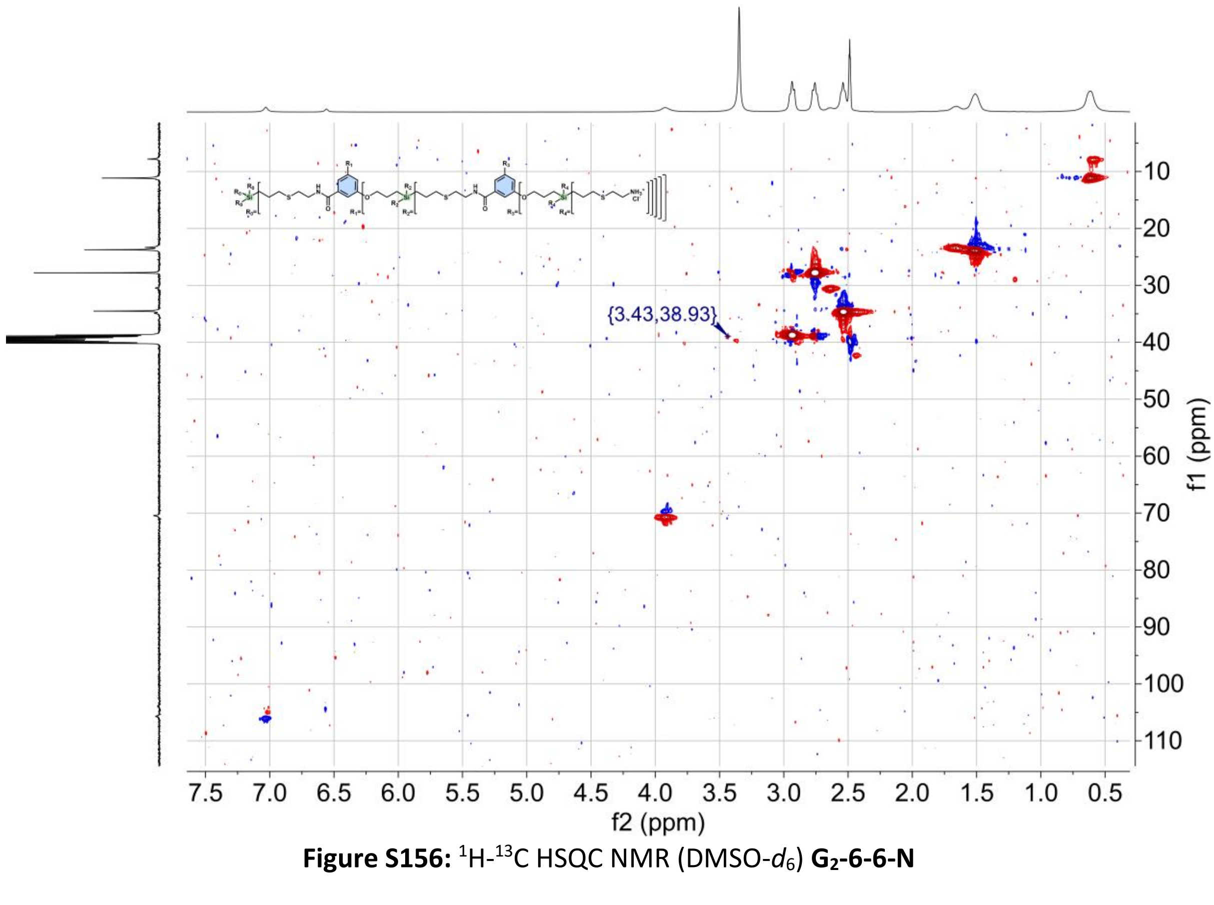

Figure S156: ^{1}H-^{13}C HSQC NMR (DMSO-d_6) **G$_2$-6-6-N**

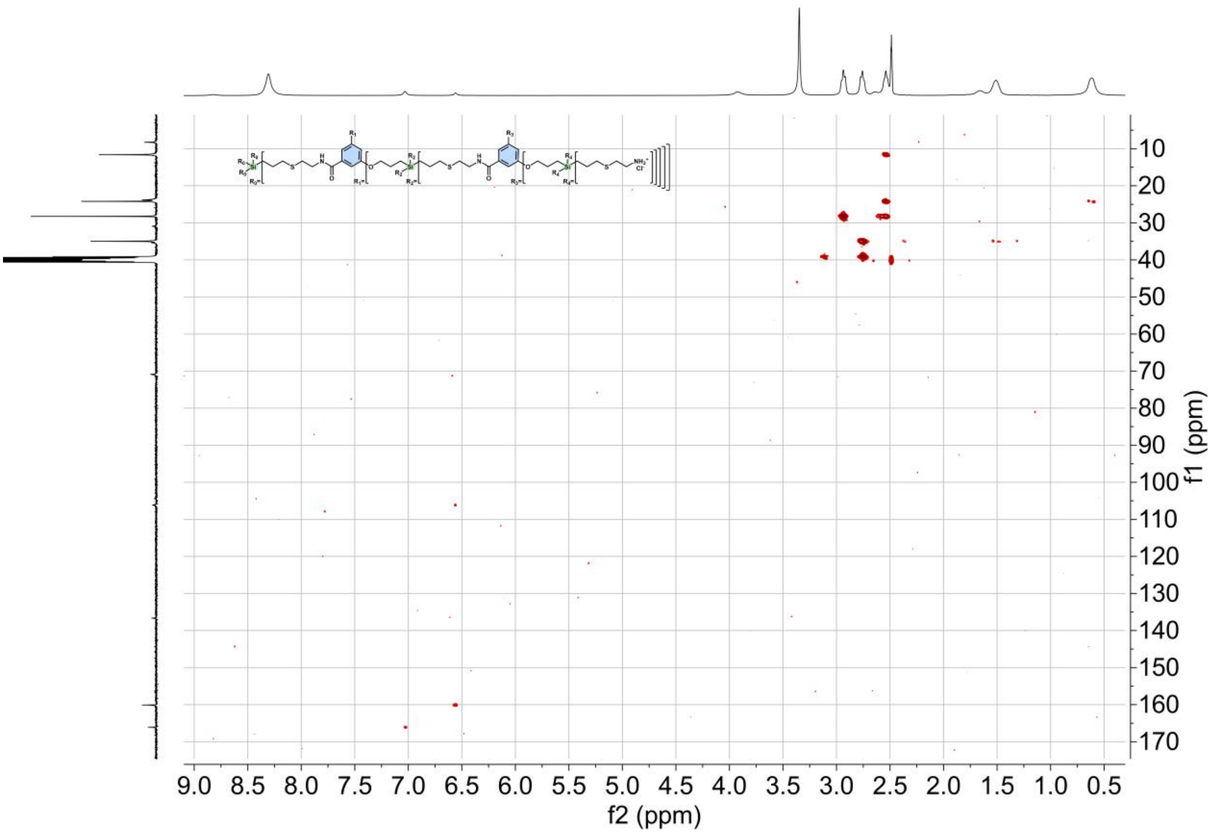

Figure S157: ^{1}H-^{13}C HMBC NMR (DMSO-d_6) **G_2-6-6-N**

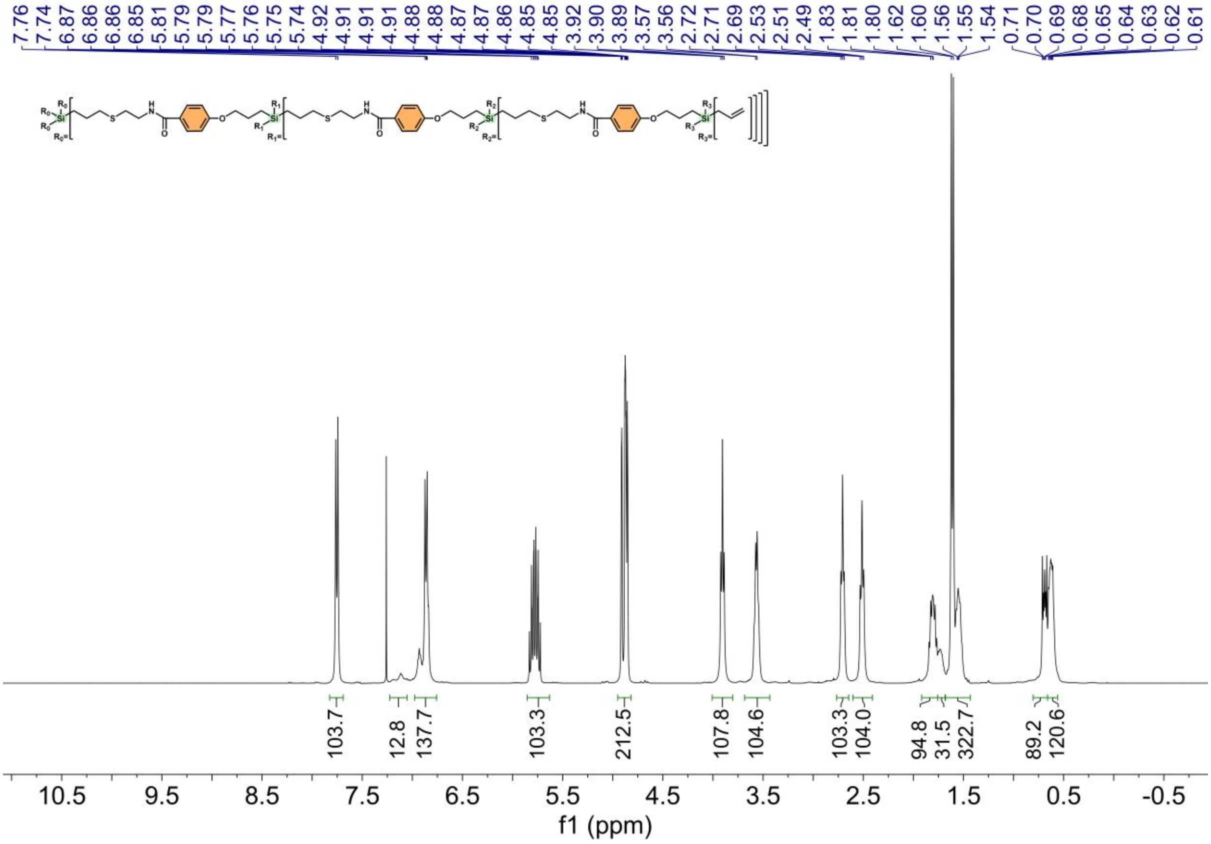

Figure S158: ^{1}H NMR (400 MHz, $CDCl_3$) **G_3-3-3-3-A**

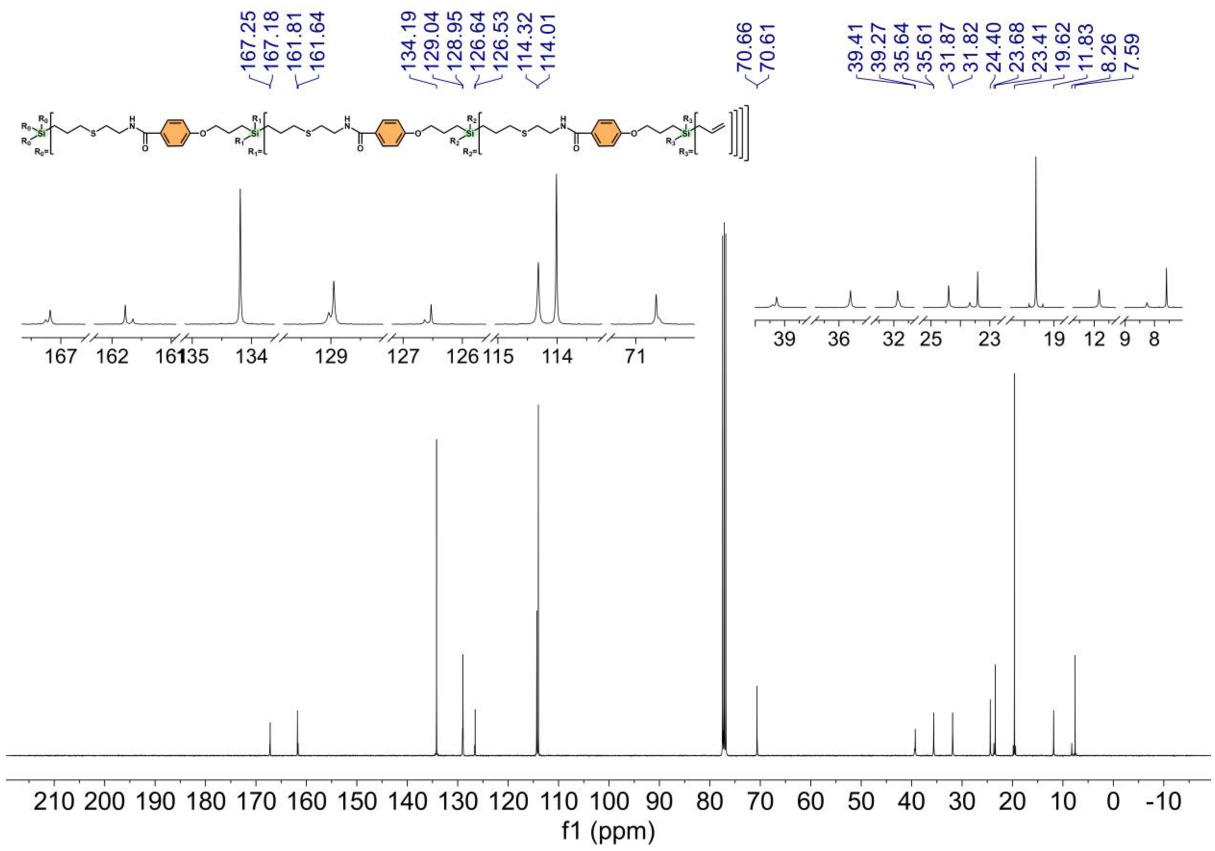

Figure S159: $^{13}C\ \{^{1}H\}$ NMR (101 MHz, $CDCl_3$) **G_3-3-3-3-A**

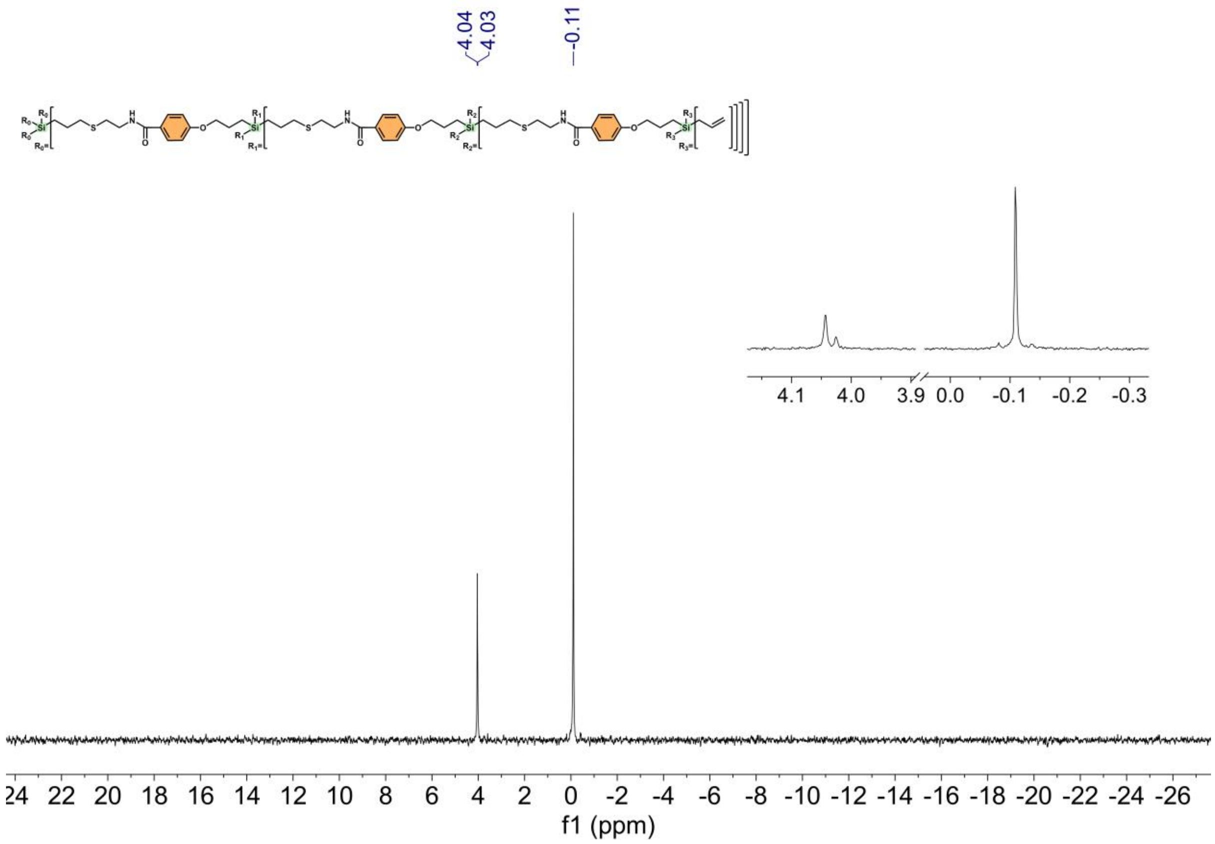

Figure S160: $^{29}Si\ \{^{1}H\}$ NMR (79 MHz, $CDCl_3$) **G_3-3-3-3-A**

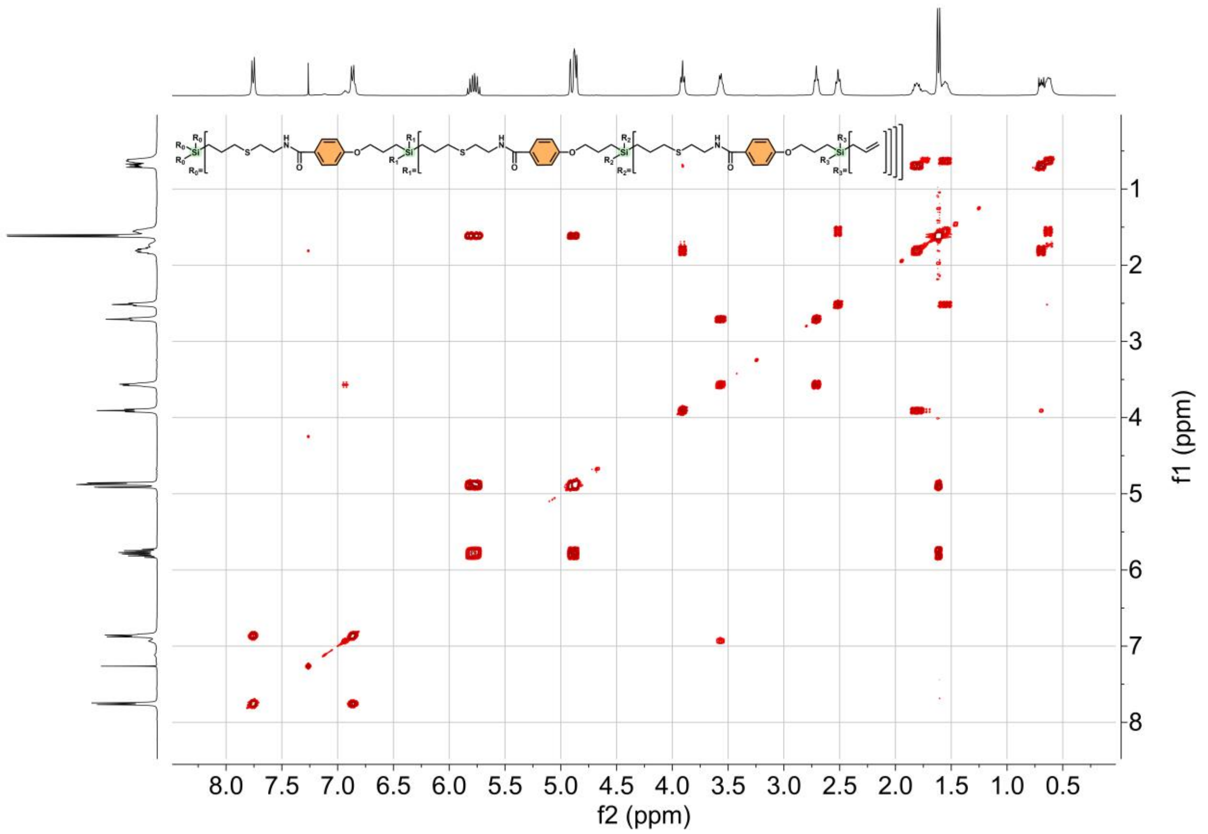

Figure S161: ^{1}H-^{1}H COSY NMR ($CDCl_3$) **G_3-3-3-3-A**

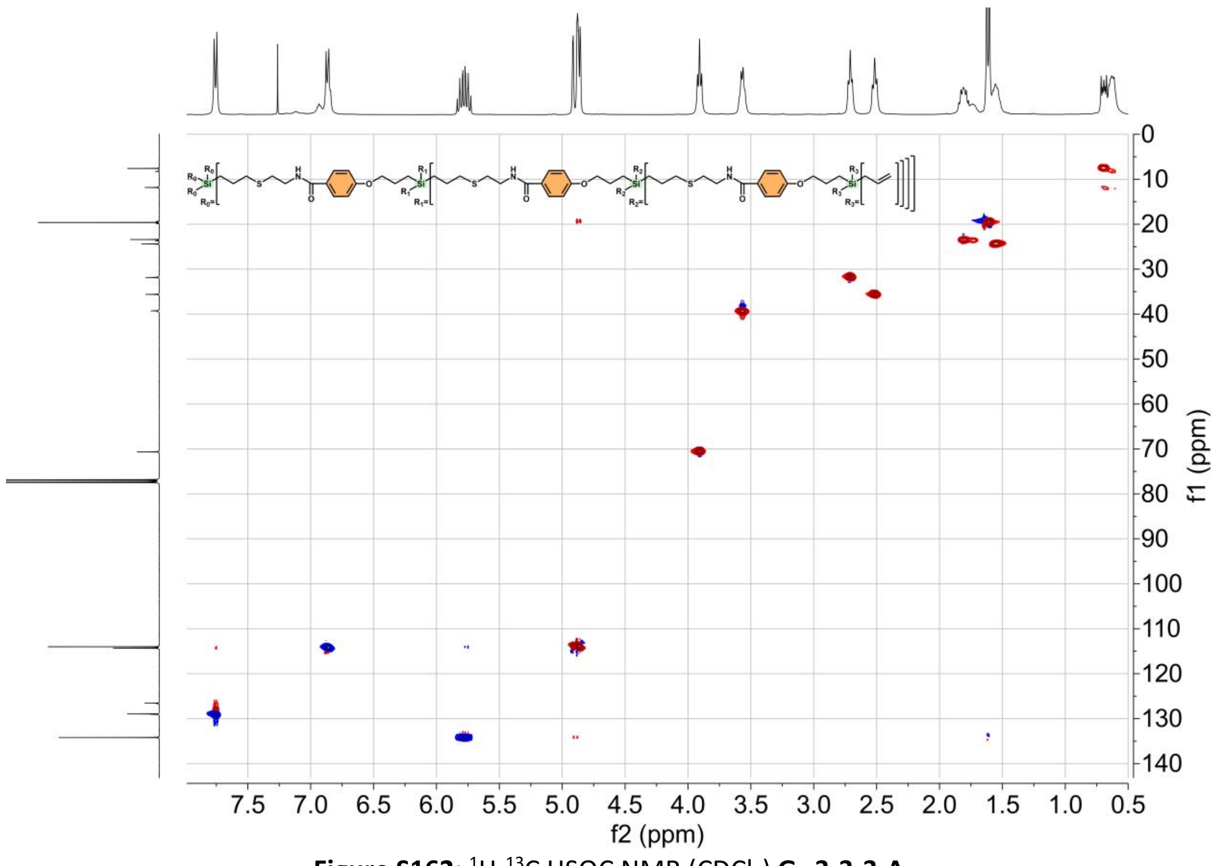

Figure S162: ^{1}H-^{13}C HSQC NMR ($CDCl_3$) **G_3-3-3-3-A**

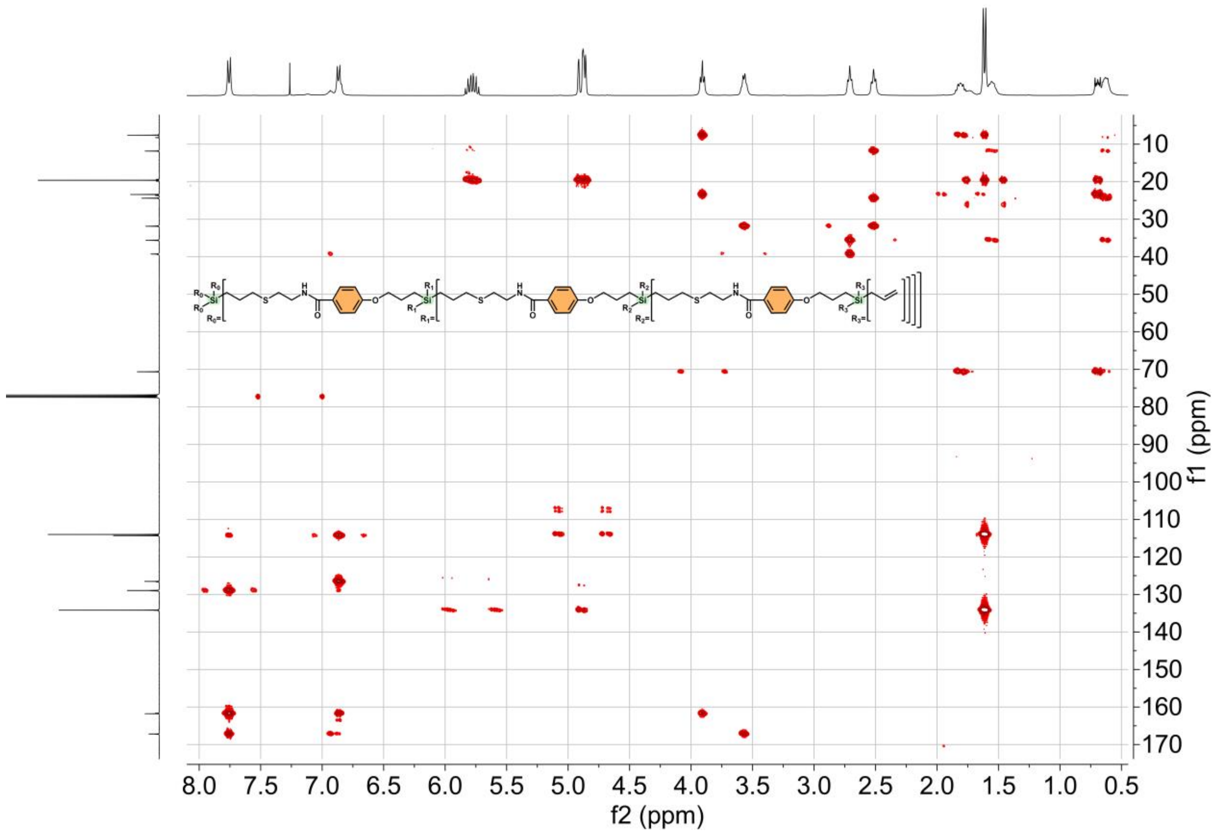

Figure S163: ^{1}H-^{13}C HMBC NMR (CDCl$_3$) **G$_3$-3-3-3-A**

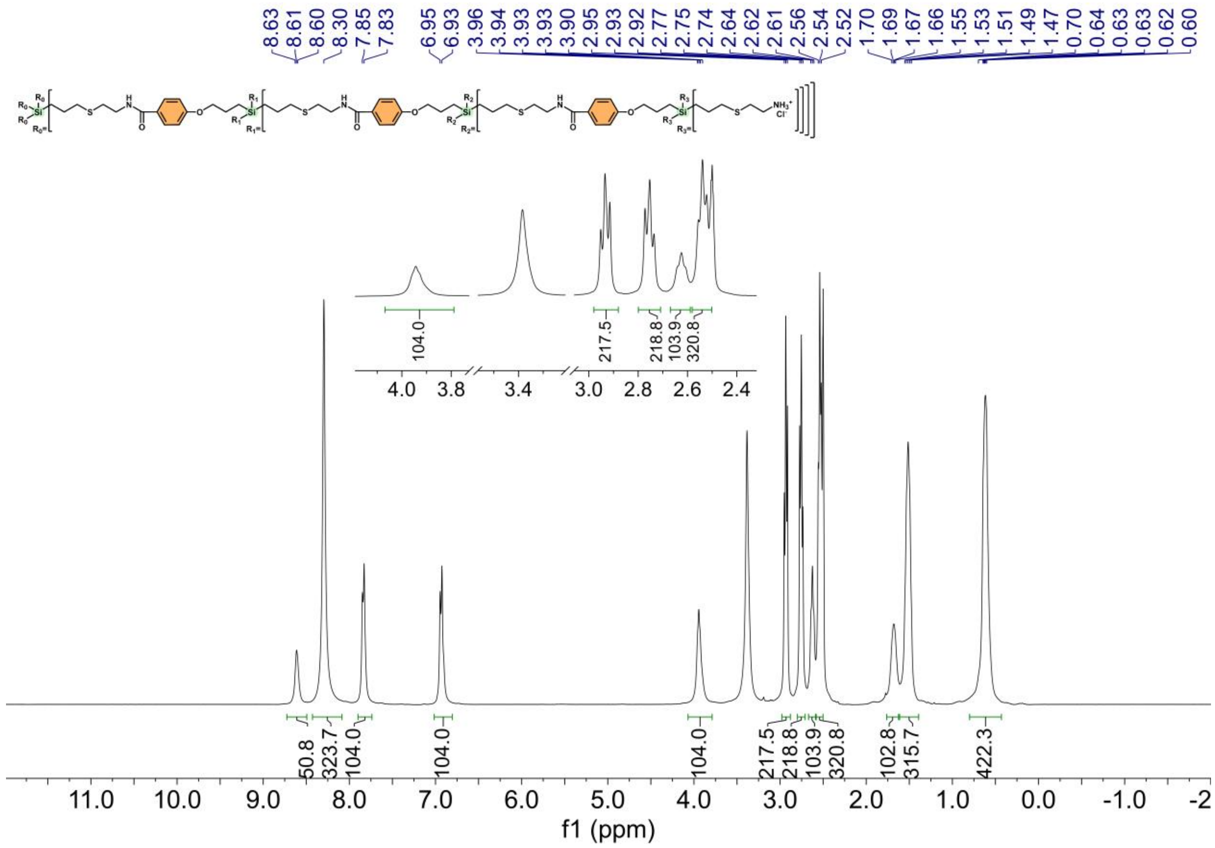

Figure S164: ^{1}H NMR (400 MHz, DMSO-d_6) **G$_3$-3-3-3-N**

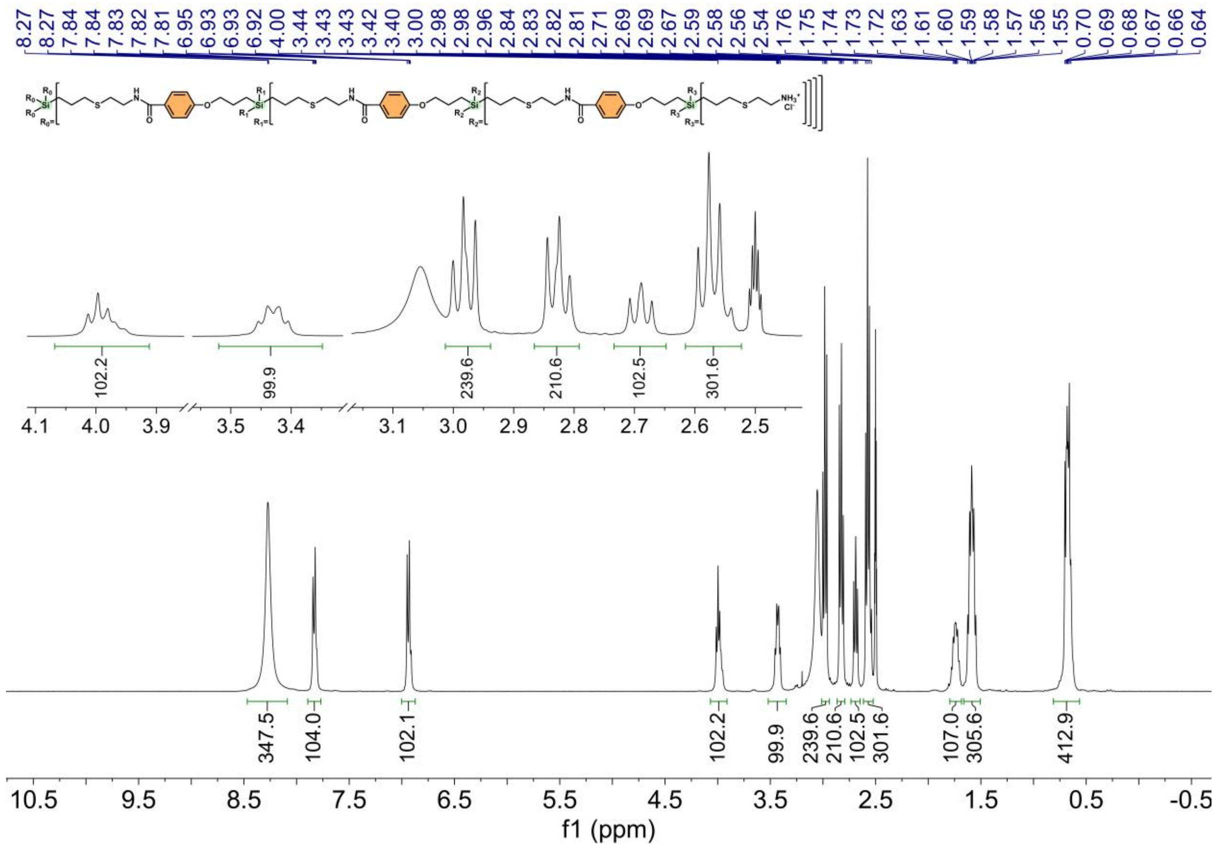

Figure S165: ^{1}H NMR (400 MHz, 100°C, DMSO-d_6) **G_3-3-3-3-N**

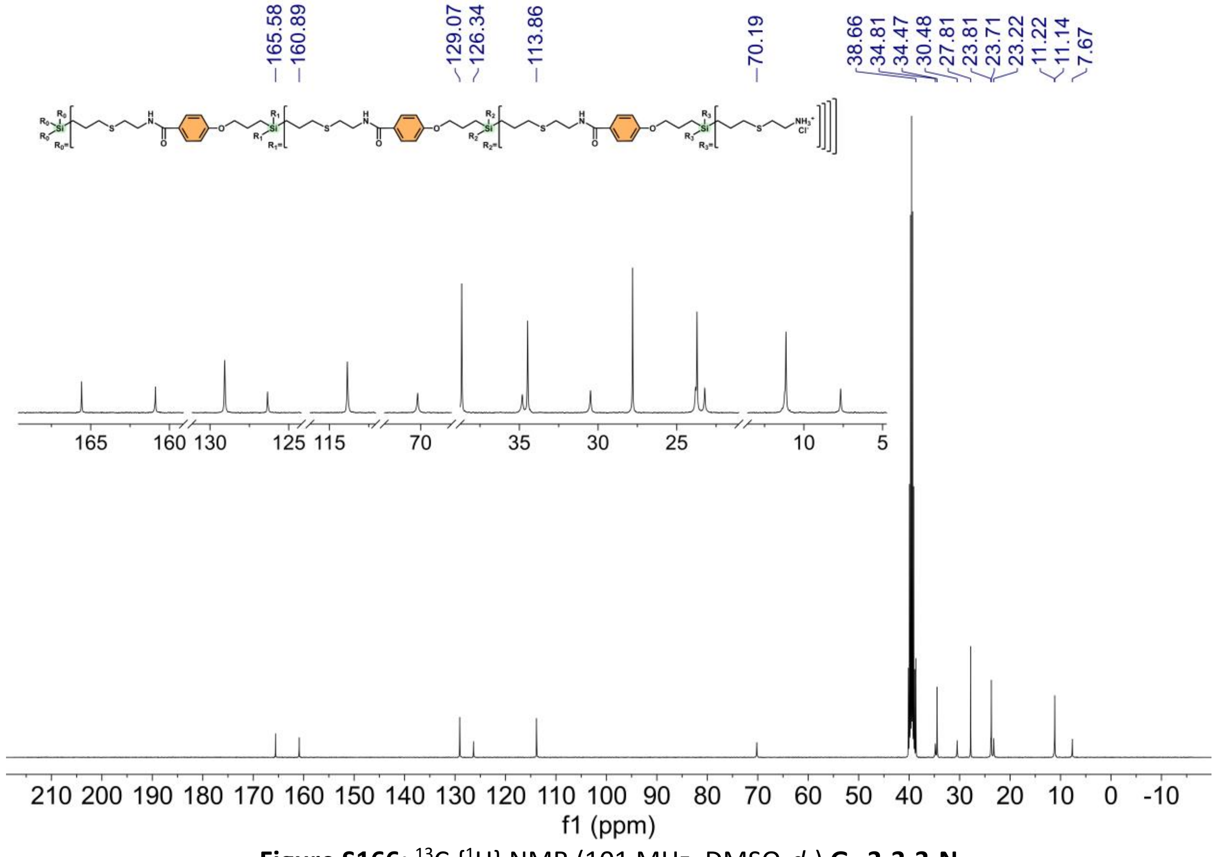

Figure S166: ^{13}C {^{1}H} NMR (101 MHz, DMSO-d_6) **G_3-3-3-3-N**

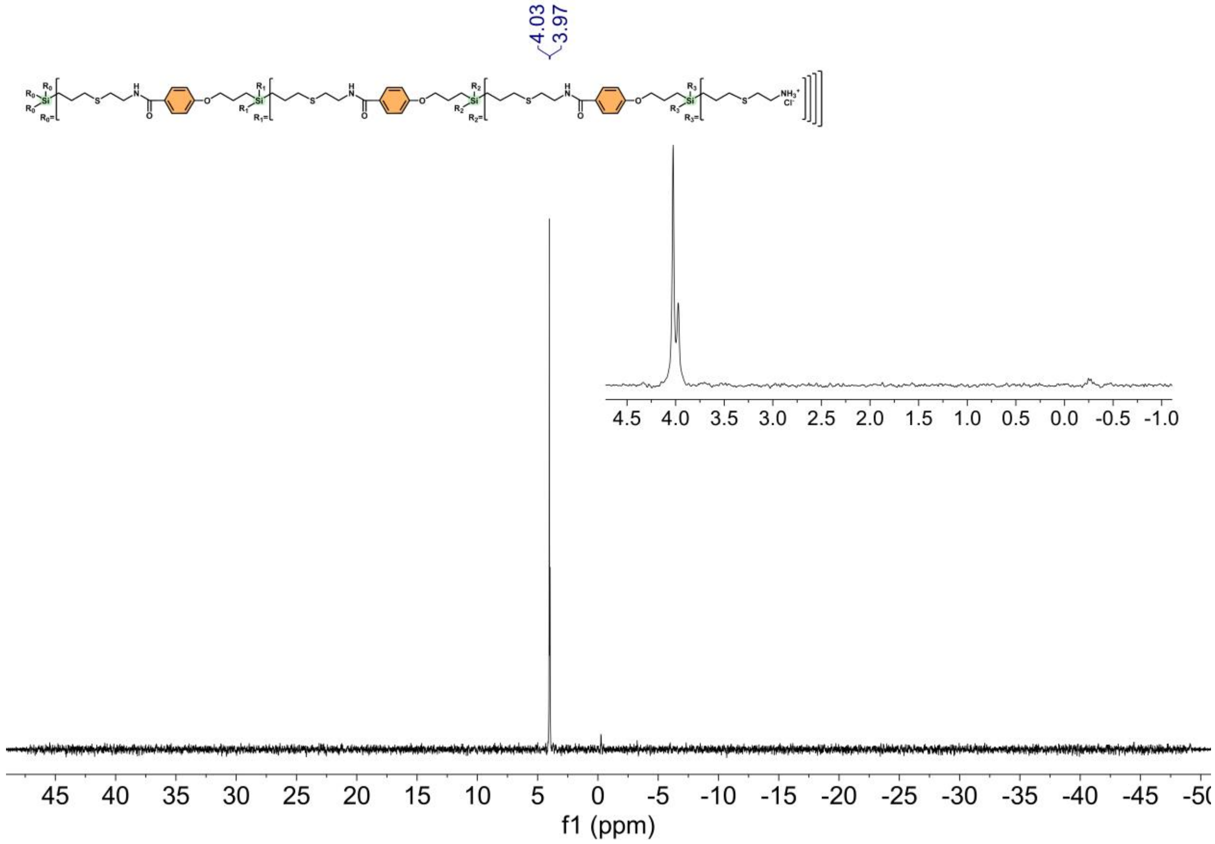

Figure S167: ^{29}Si {^{1}H} NMR (79 MHz, DMSO-d_6) **G_3-3-3-3-N**

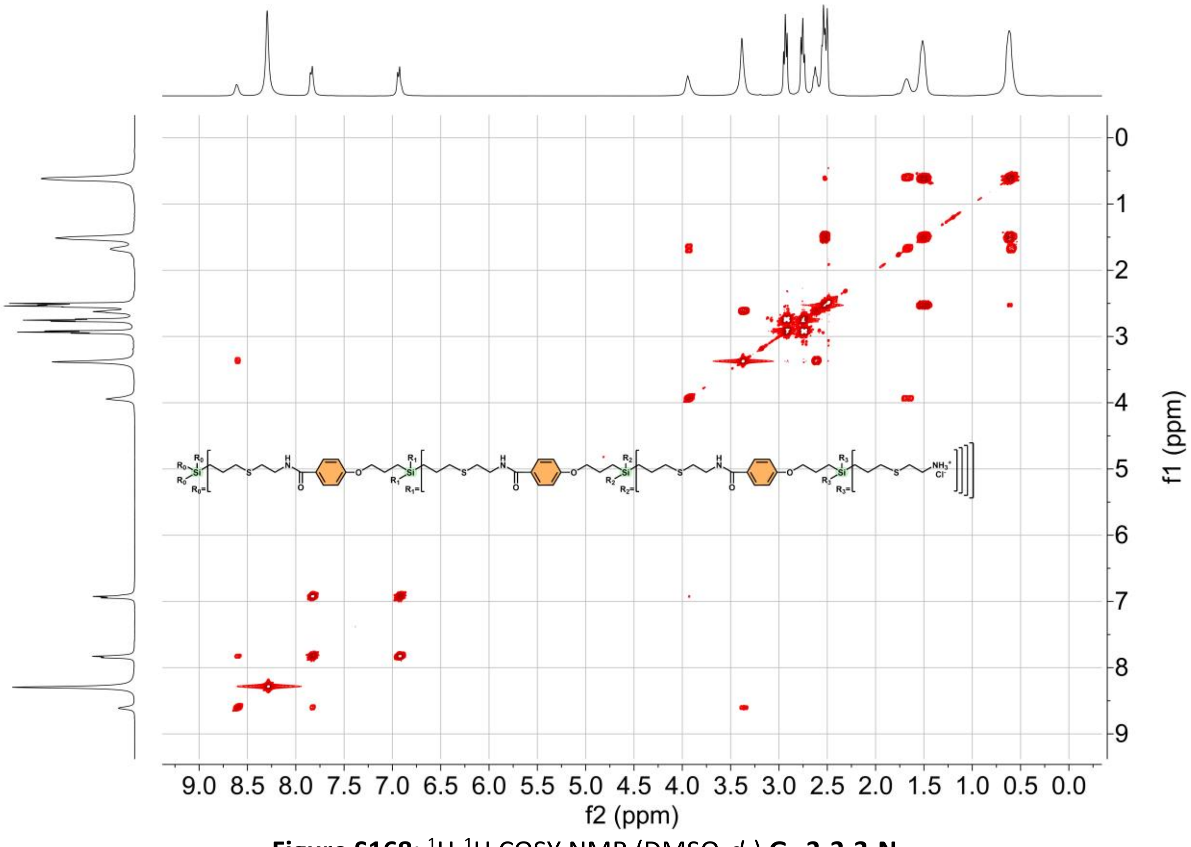

Figure S168: ^{1}H-^{1}H COSY NMR (DMSO-d_6) **G_3-3-3-3-N**

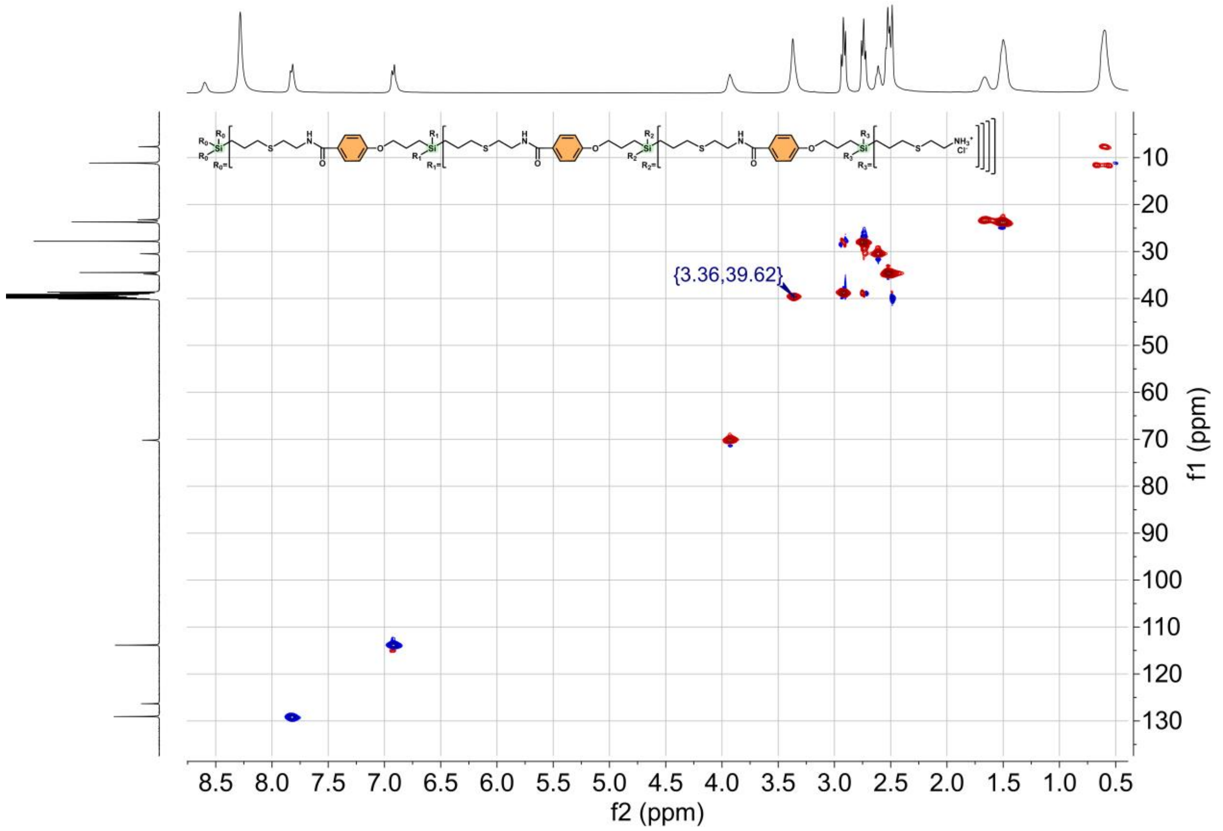

Figure S169: ^{1}H-^{13}C HSQC NMR (DMSO-d_6) **G_3-3-3-3-N**

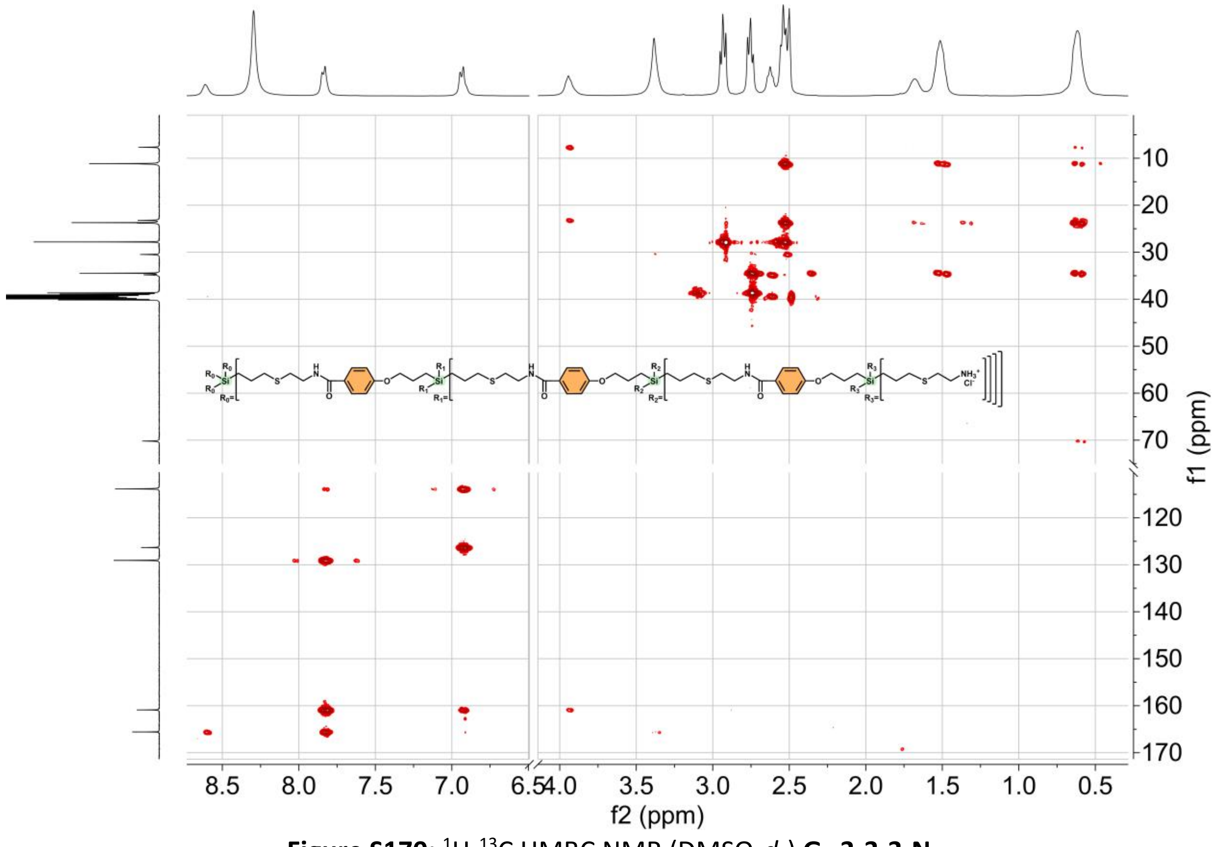

Figure S170: ^{1}H-^{13}C HMBC NMR (DMSO-d_6) **G_3-3-3-3-N**

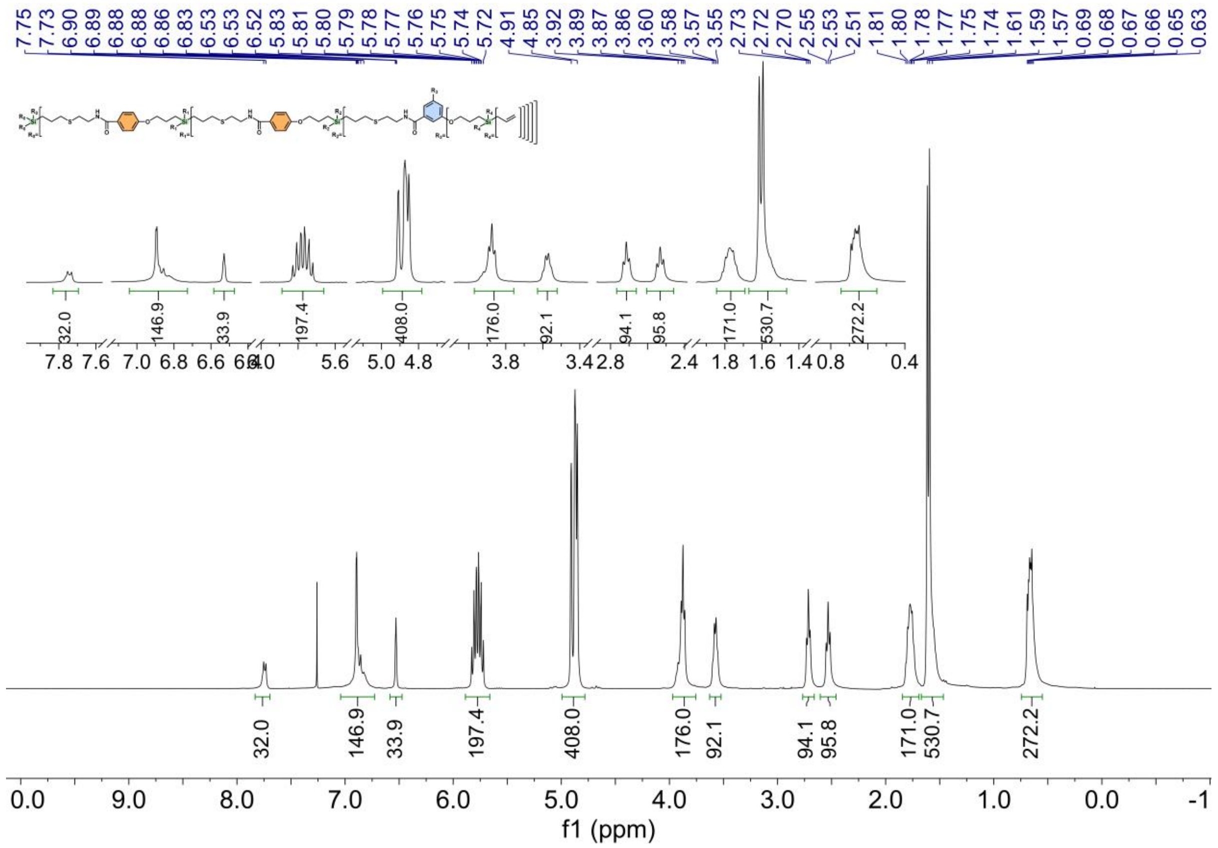

Figure S171: ^{1}H NMR (400 MHz, $CDCl_3$) **G_3-3-3-6-A**

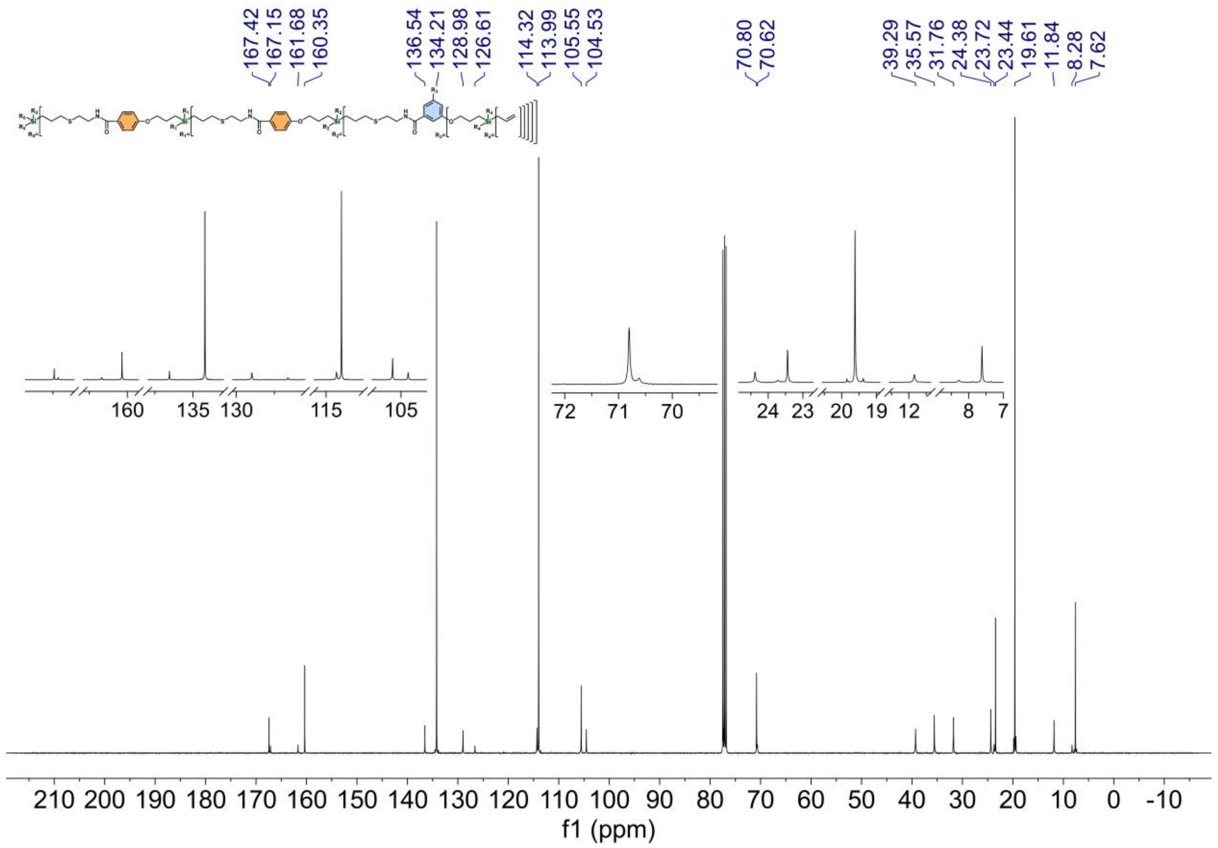

Figure S172: ^{13}C {^{1}H} NMR (101 MHz, $CDCl_3$) **G_3-3-3-6-A**

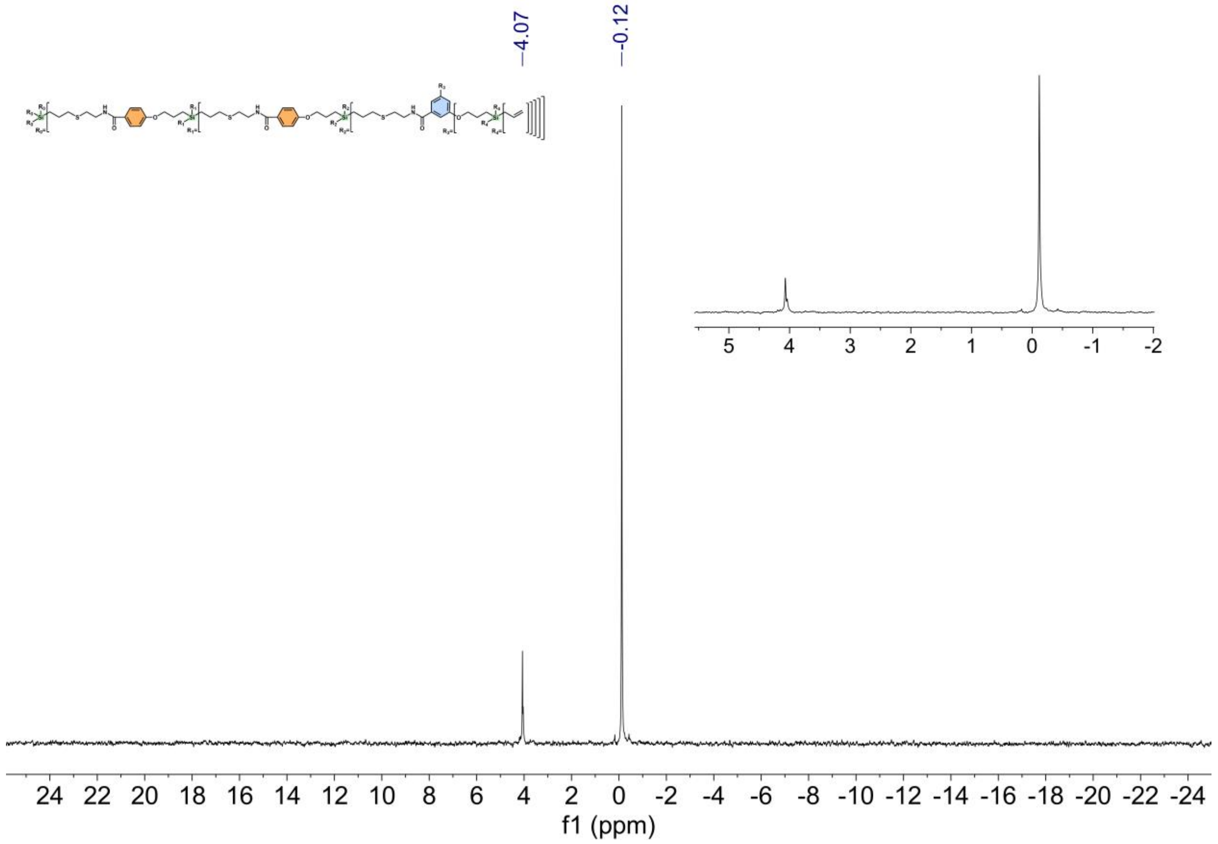

Figure S173: $^{29}Si\ \{^{1}H\}$ NMR (79 MHz, $CDCl_3$) **G_3-3-3-6-A**

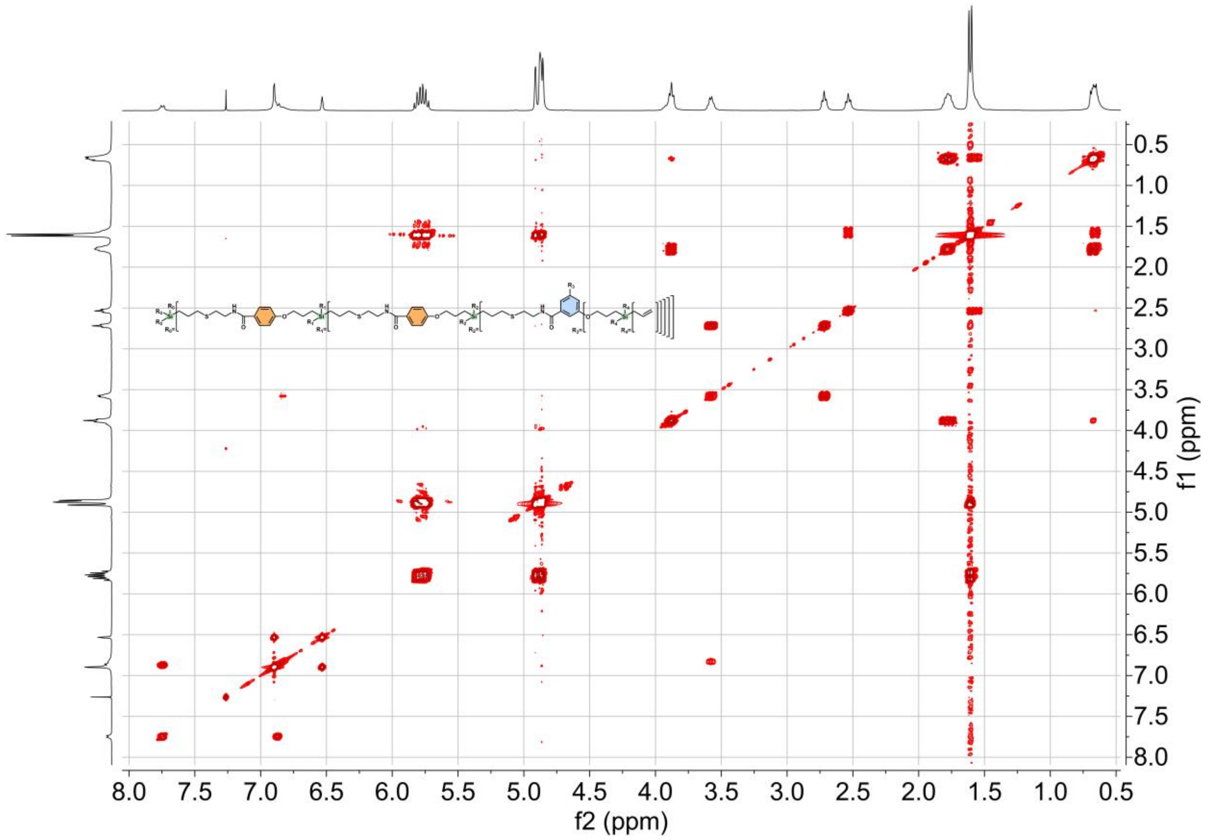

Figure S174: ^{1}H-^{1}H COSY NMR ($CDCl_3$) **G_3-3-3-6-A**

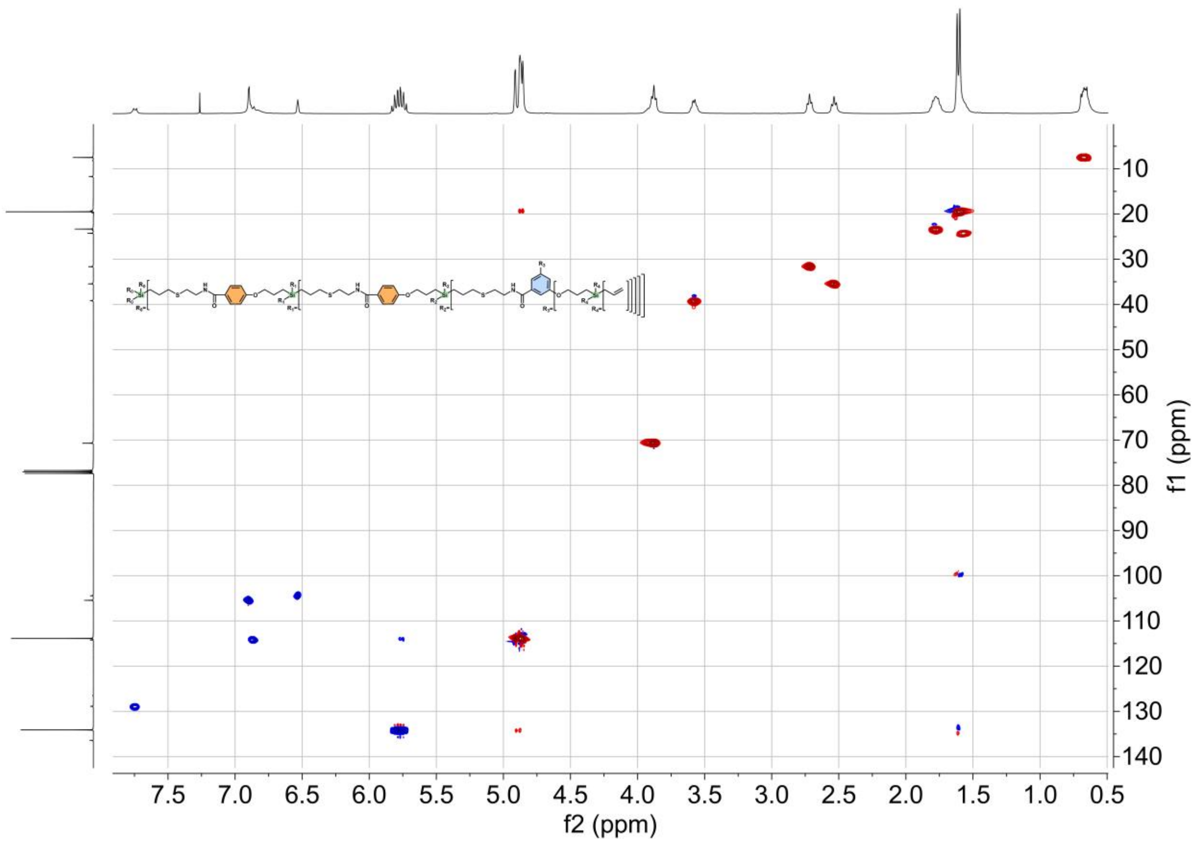

Figure S175: ^{1}H-^{13}C HSQC NMR ($CDCl_3$) **G_3-3-3-6-A**

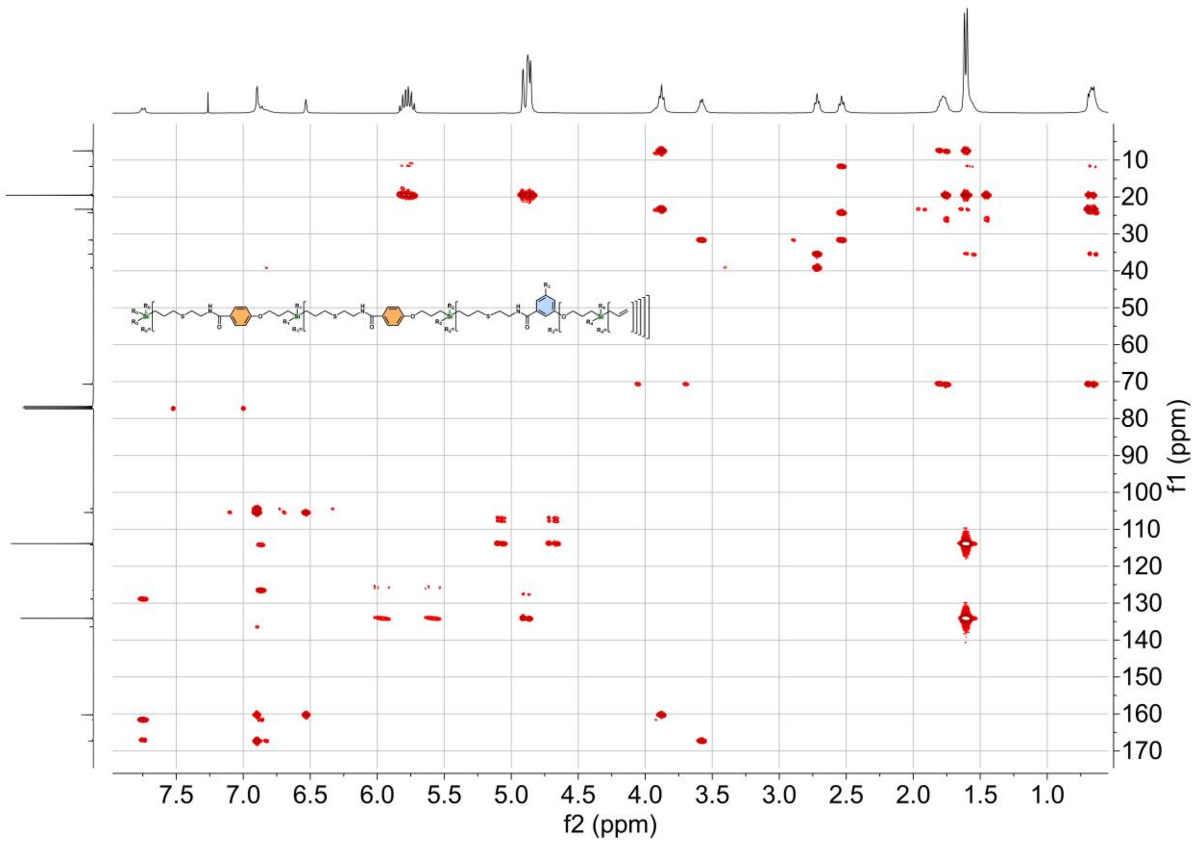

Figure S176: ^{1}H-^{13}C HMBC NMR ($CDCl_3$) **G_3-3-3-6-A**

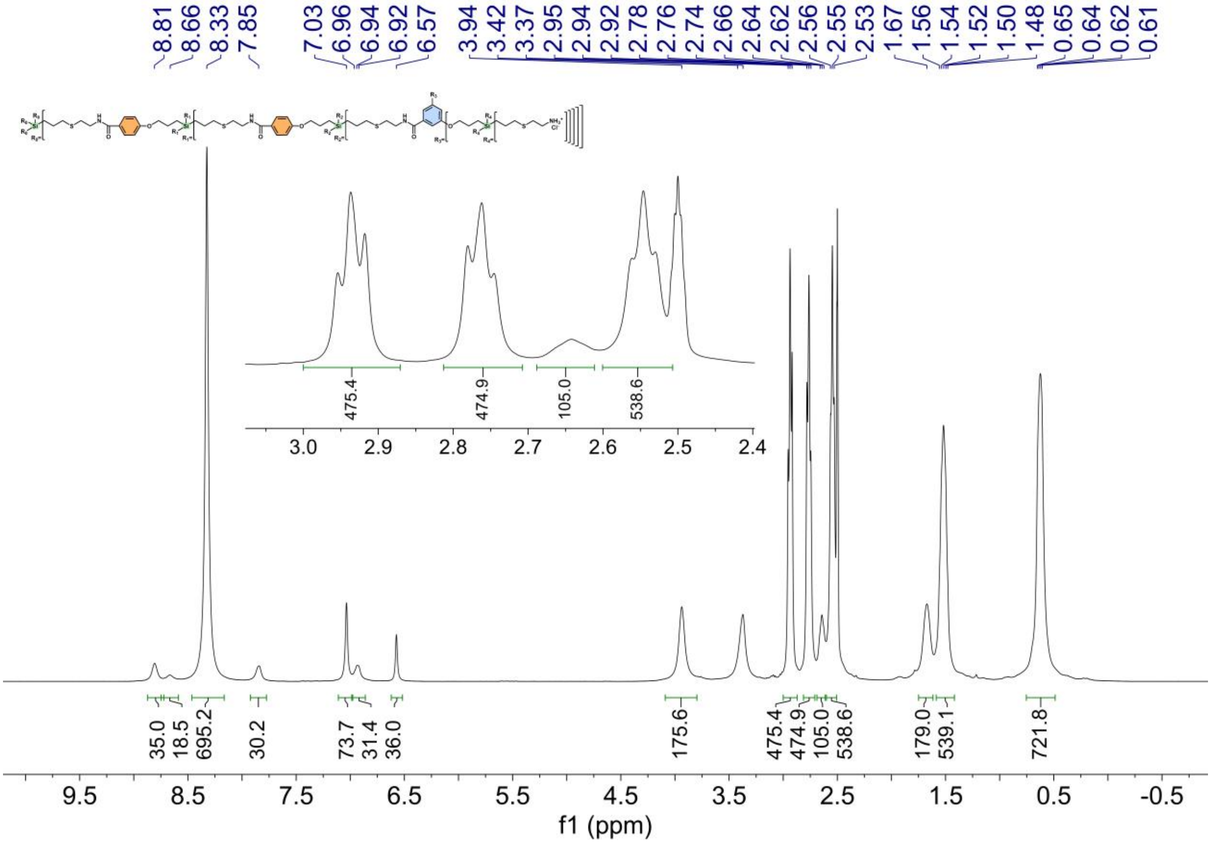

Figure S177: ^{1}H NMR (400 MHz, DMSO-d_6) **G_3-3-3-6-N**

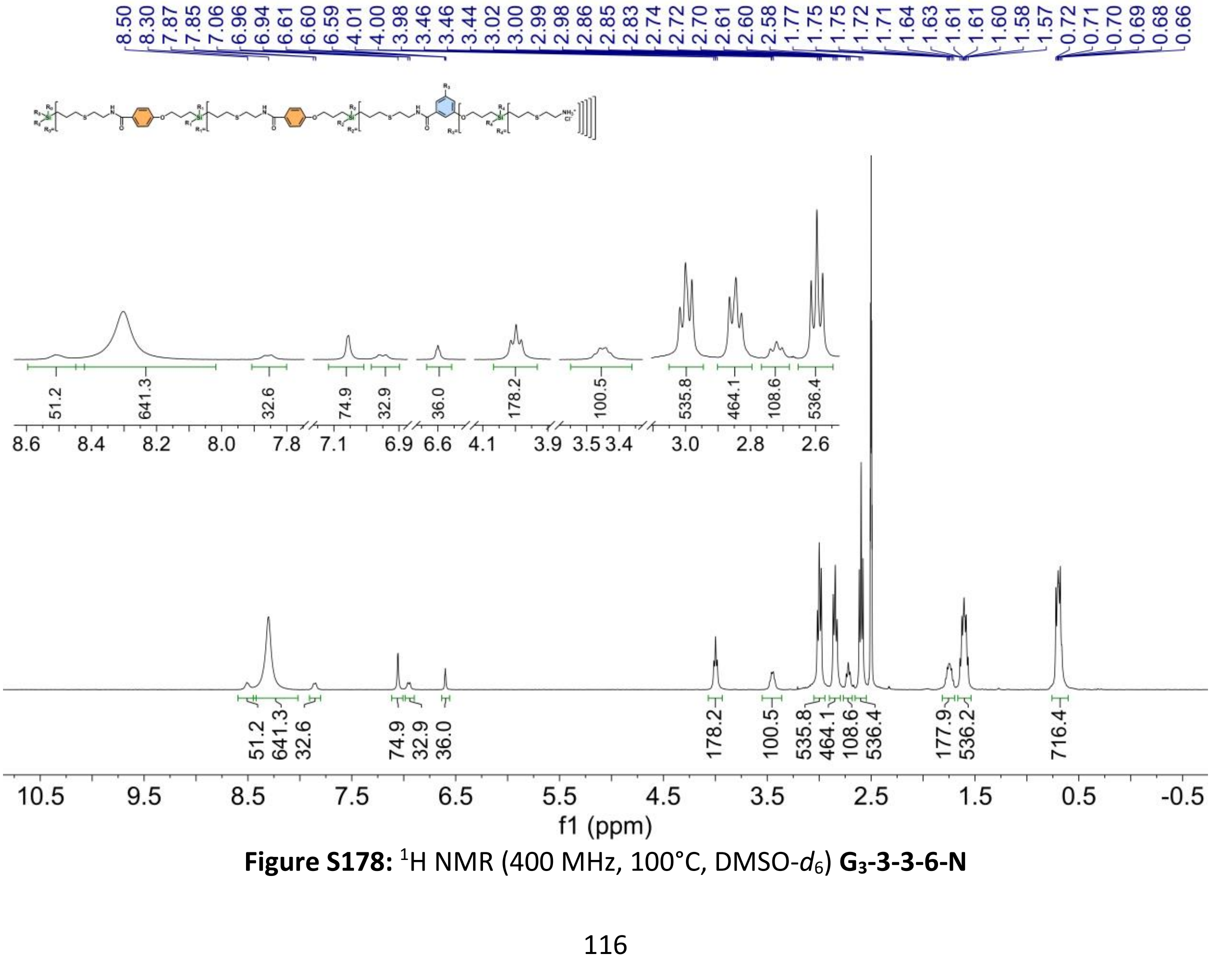

Figure S178: ^{1}H NMR (400 MHz, 100°C, DMSO-d_6) **G_3-3-3-6-N**

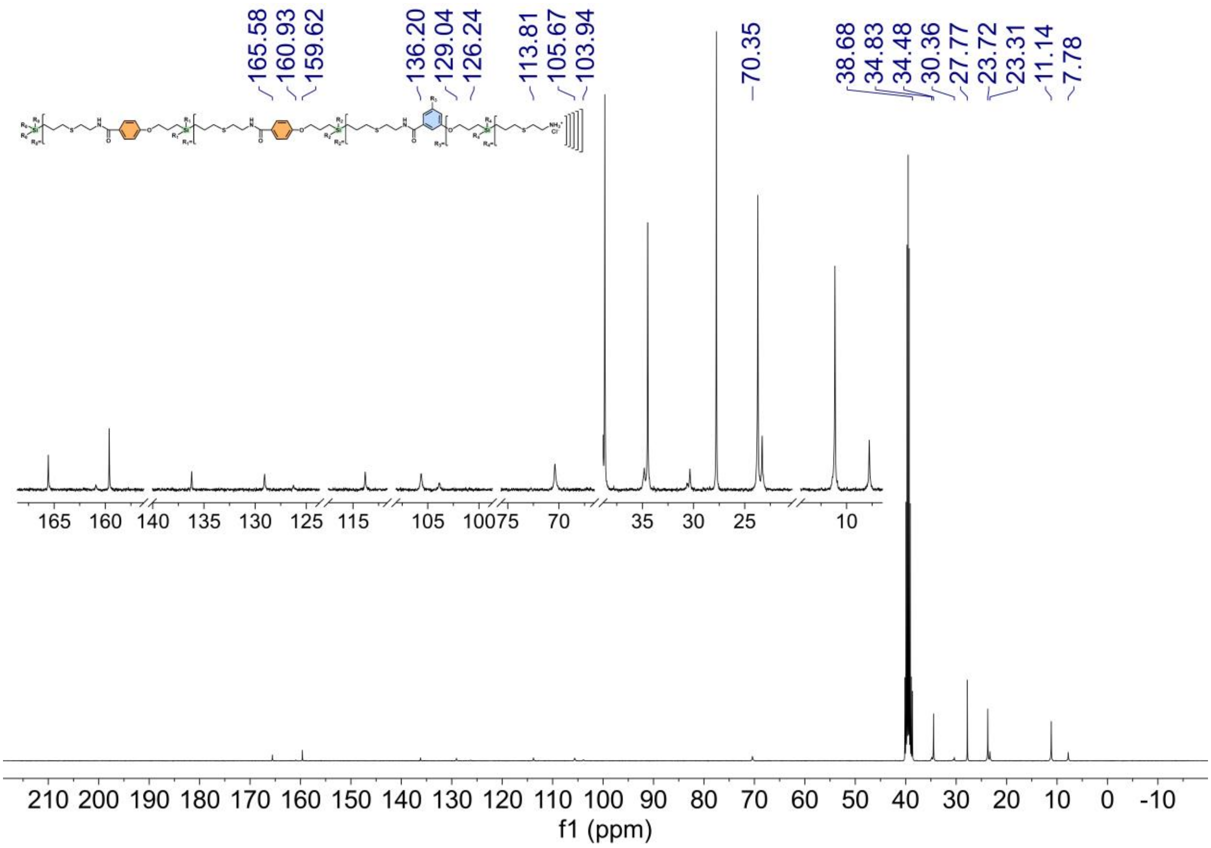

Figure S179: ^{13}C {^{1}H} NMR (101 MHz, DMSO-d_6) **G_3-3-3-6-N**

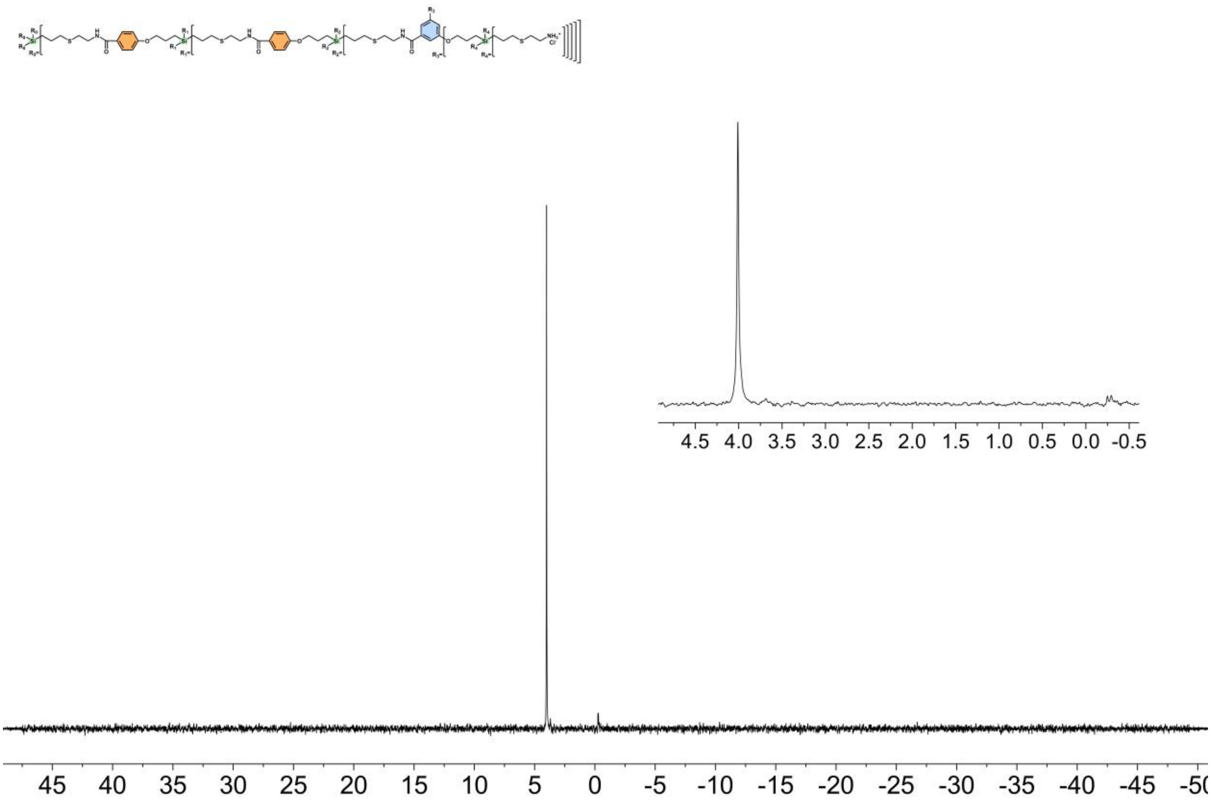

Figure S180: ^{29}Si {^{1}H} NMR (79 MHz, DMSO-d_6) **G_3-3-3-6-N**

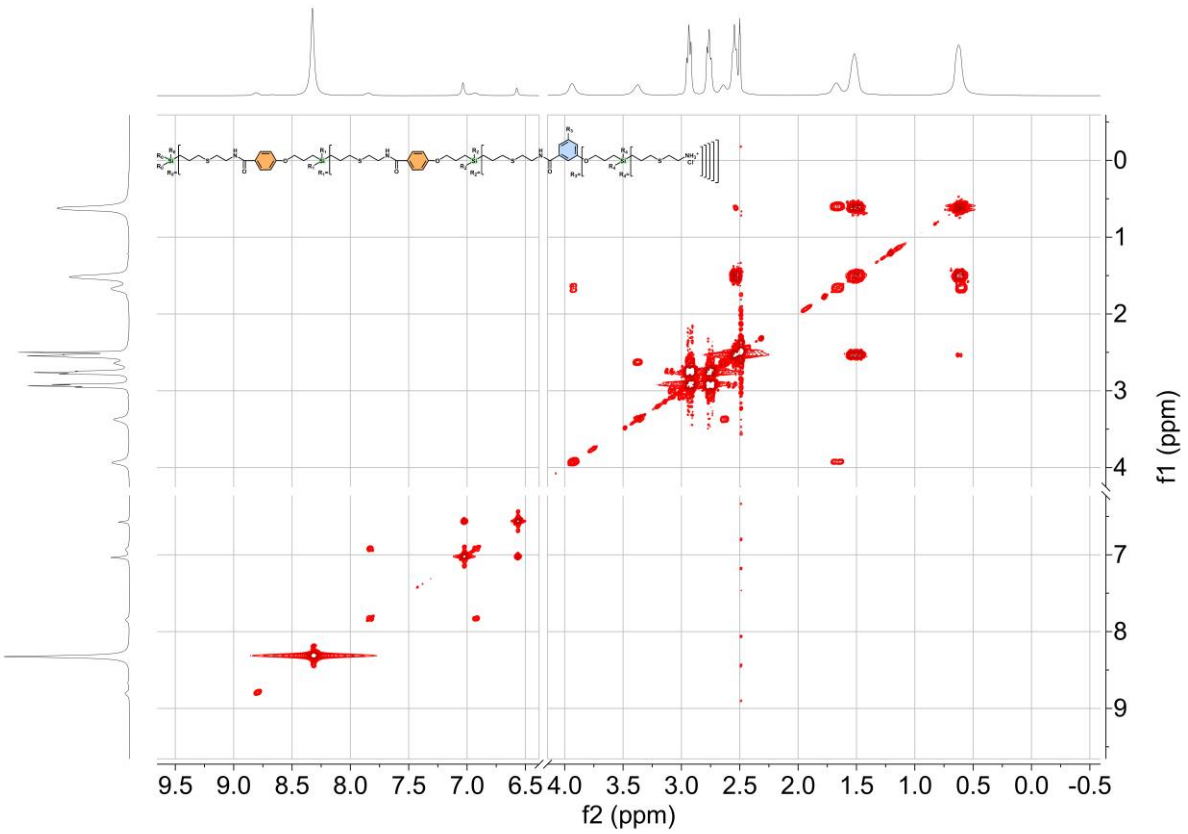

Figure S181: ^{1}H-^{1}H COSY NMR (DMSO-d_6) **G_3-3-3-6-N**

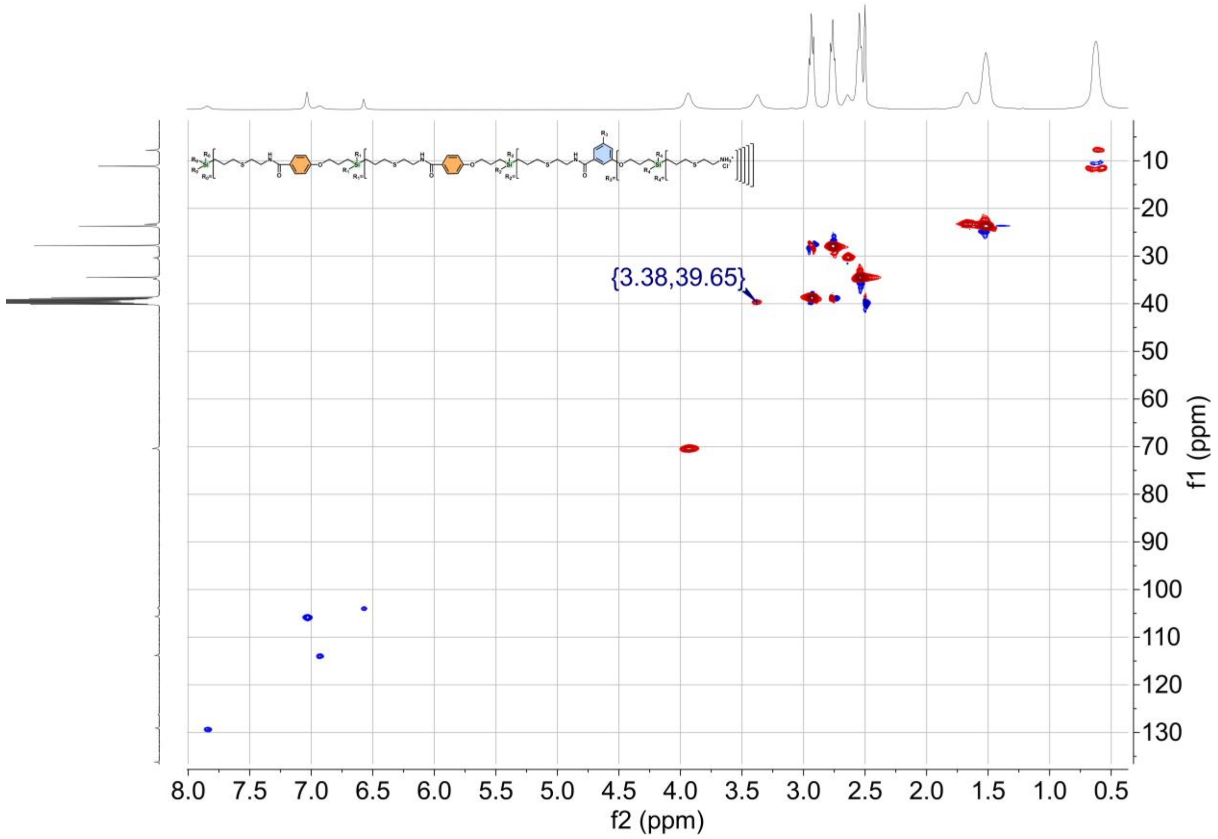

Figure S182: ^{1}H-^{13}C HSQC NMR (DMSO-d_6) **G_3-3-3-6-N**

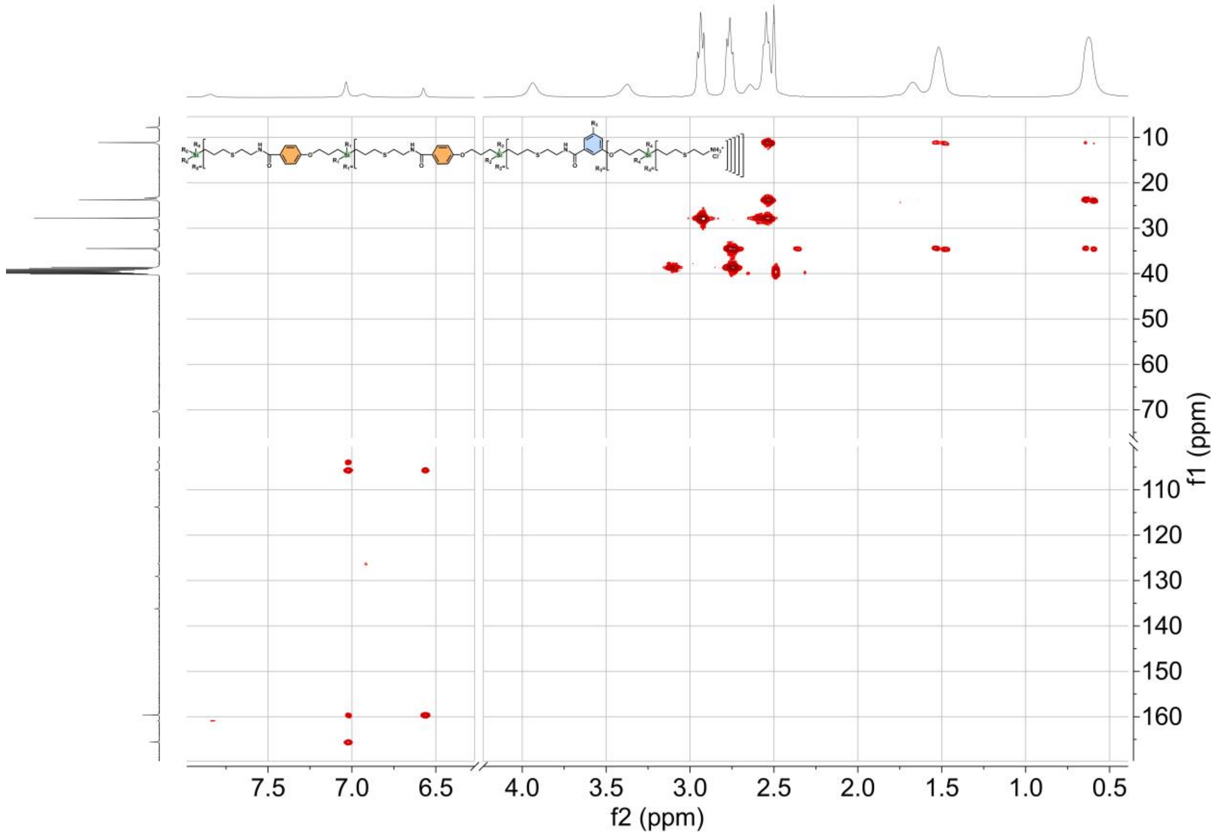

Figure S183: ^{1}H-^{13}C HMBC NMR (DMSO-d_6) **G_3-3-3-6-N**

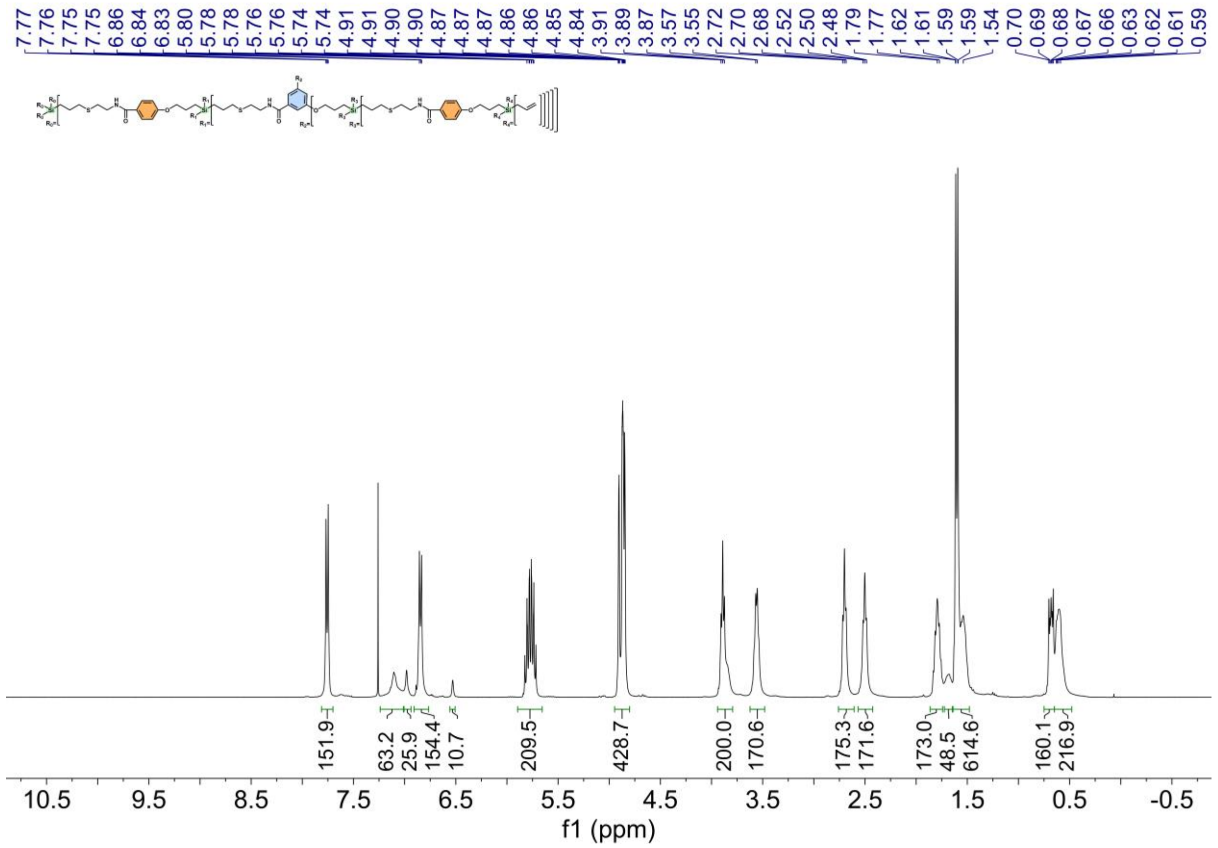

Figure S184: ^{1}H NMR (400 MHz, $CDCl_3$) **G_3-3-6-3-A**

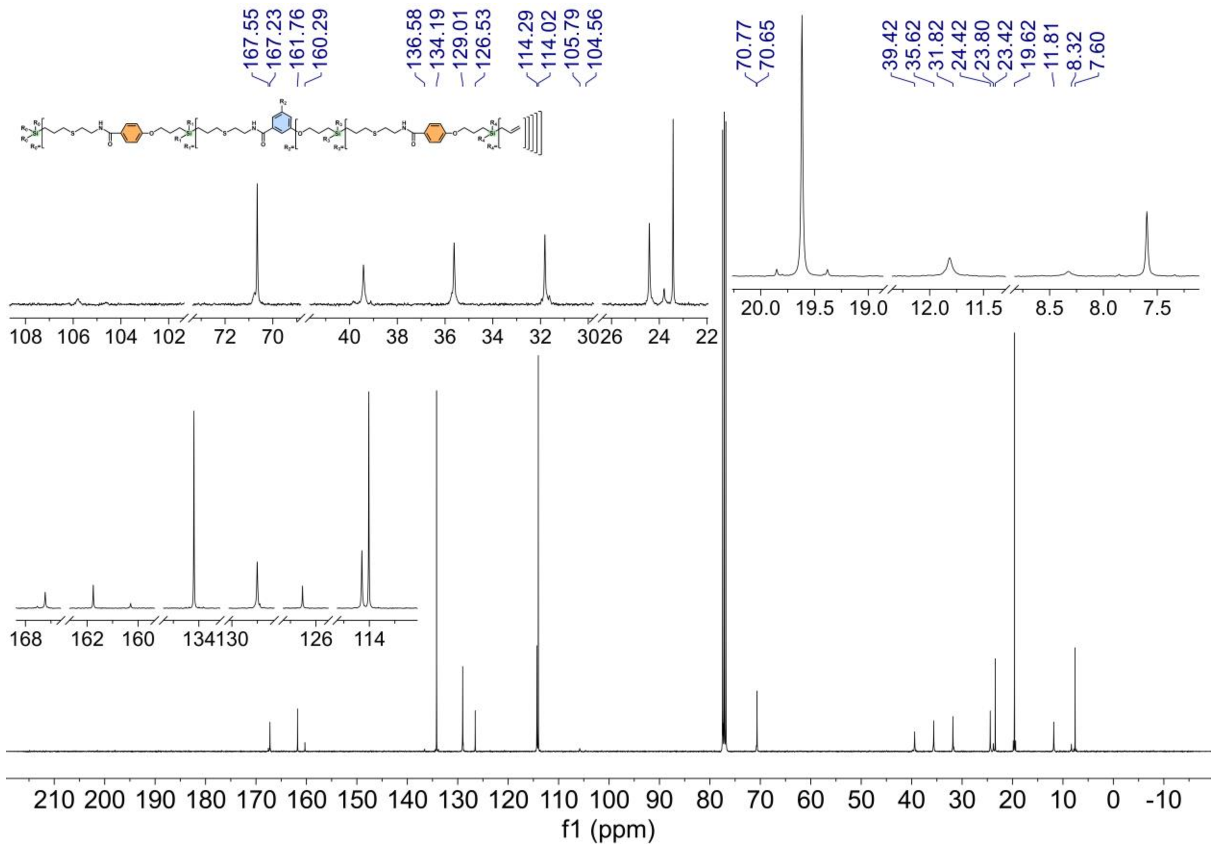

Figure S185: ^{13}C {^{1}H} NMR (101 MHz, $CDCl_3$) **G_3-3-6-3-A**

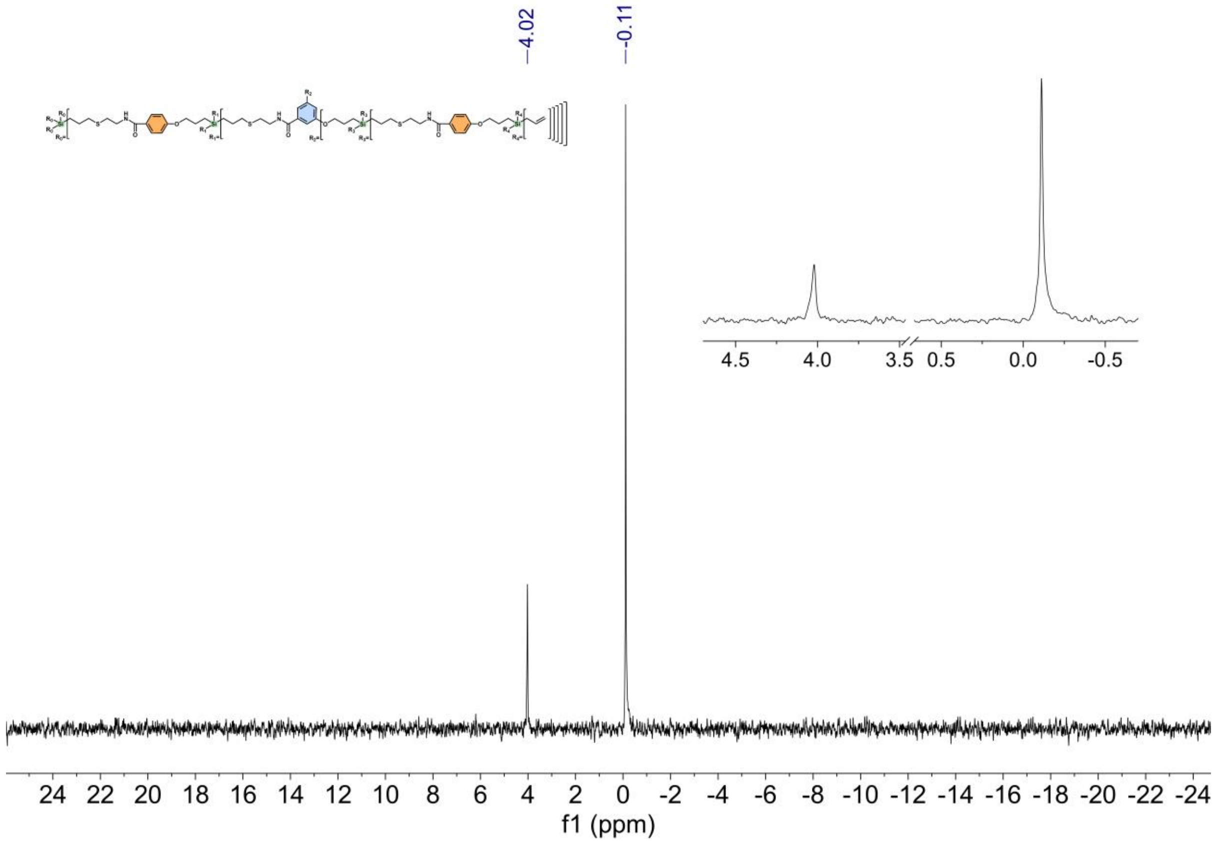

Figure S186: ^{29}Si {^{1}H} NMR (79 MHz, $CDCl_3$) **G_3-3-6-3-A**

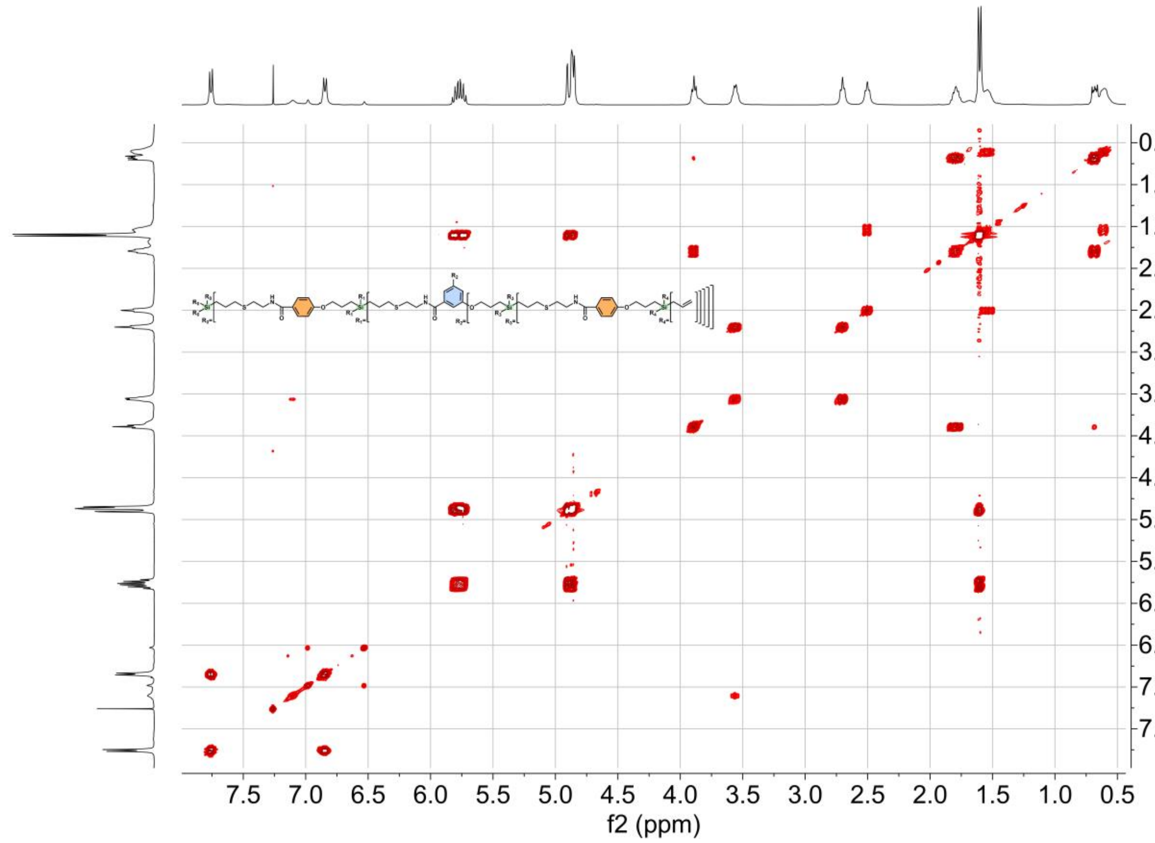

Figure S187: ^{1}H-^{1}H COSY NMR ($CDCl_3$) **G_3-3-6-3-A**

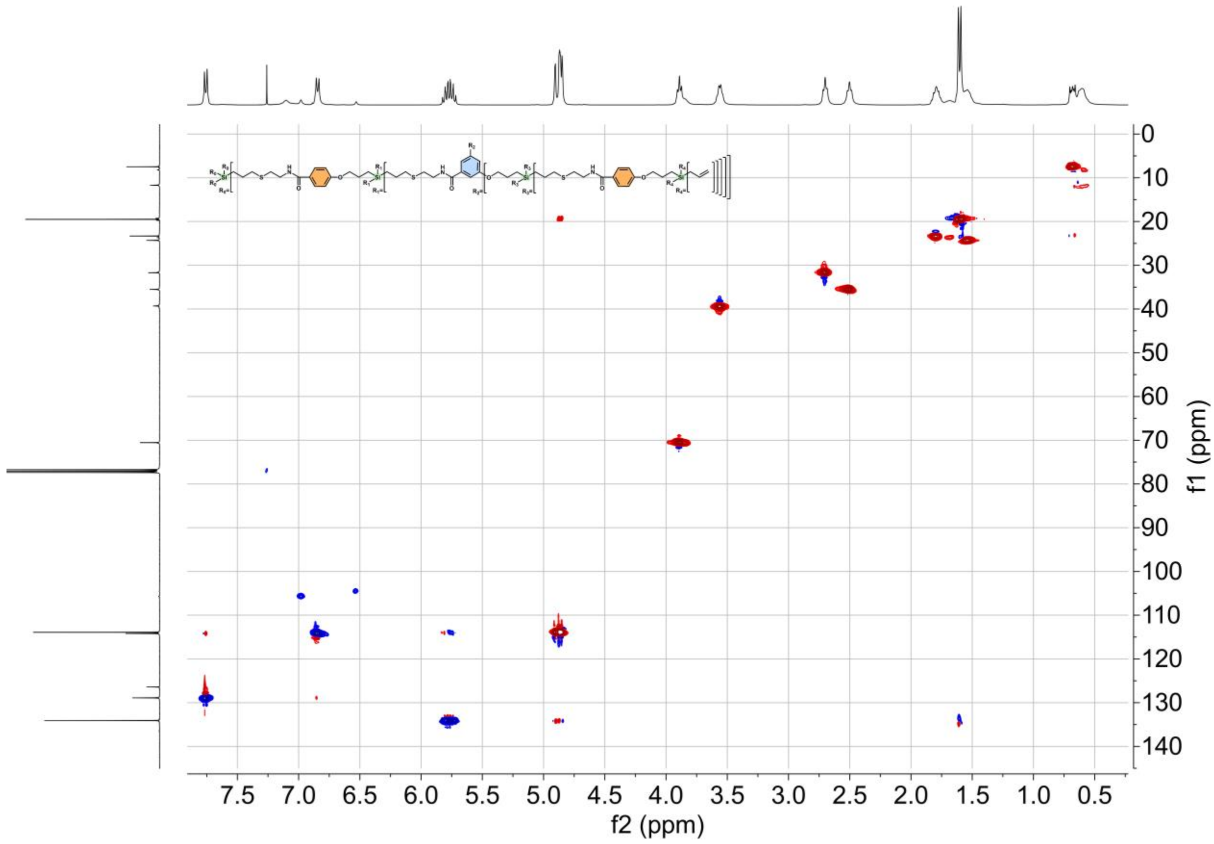

Figure S188: ^{1}H-^{13}C HSQC NMR ($CDCl_3$) **G_3-3-6-3-A**

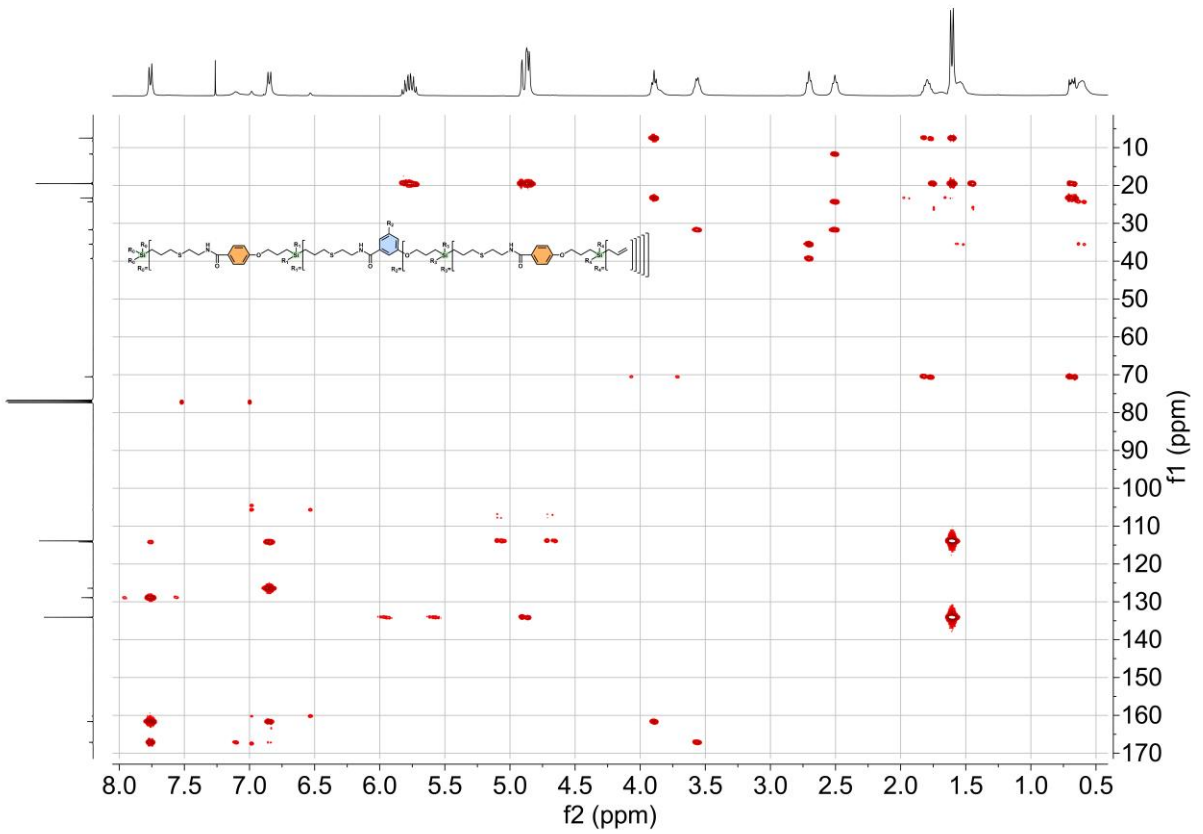

Figure S189: ^{1}H-^{13}C HMBC NMR ($CDCl_3$) **G_3-3-6-3-A**

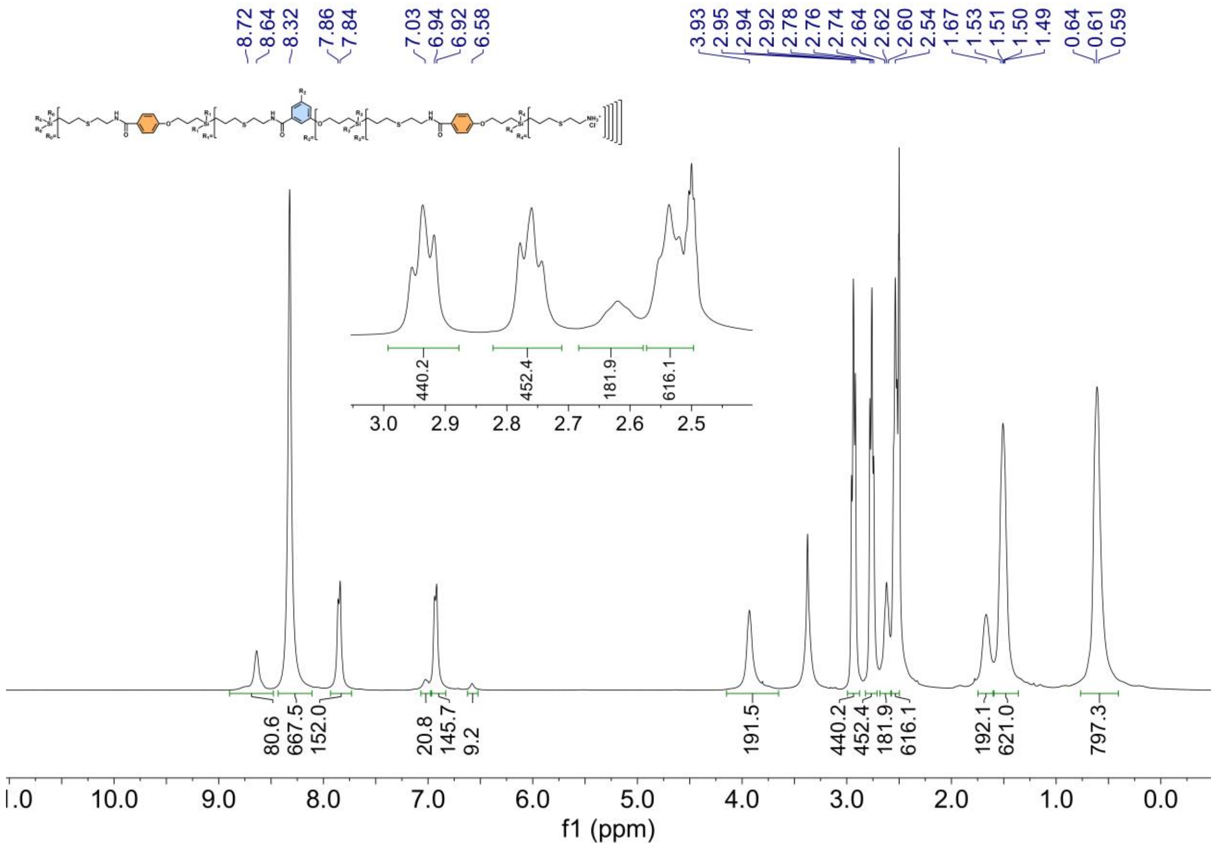

Figure S190: ^{1}H NMR (400 MHz, DMSO-d_6) **G_3-3-6-3-N**

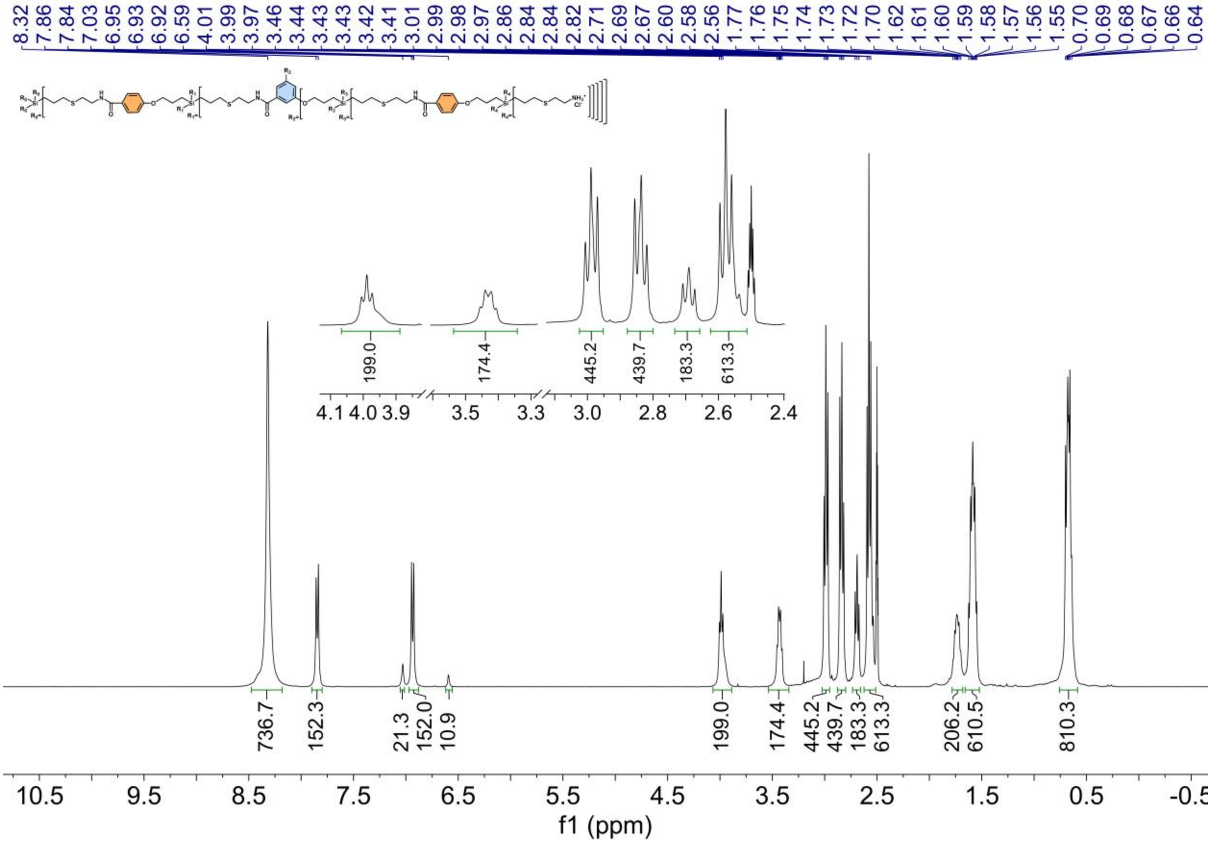

Figure S191: ^{1}H NMR (400 MHz, 100°C, DMSO-d_6) **G_3-3-6-3-N**

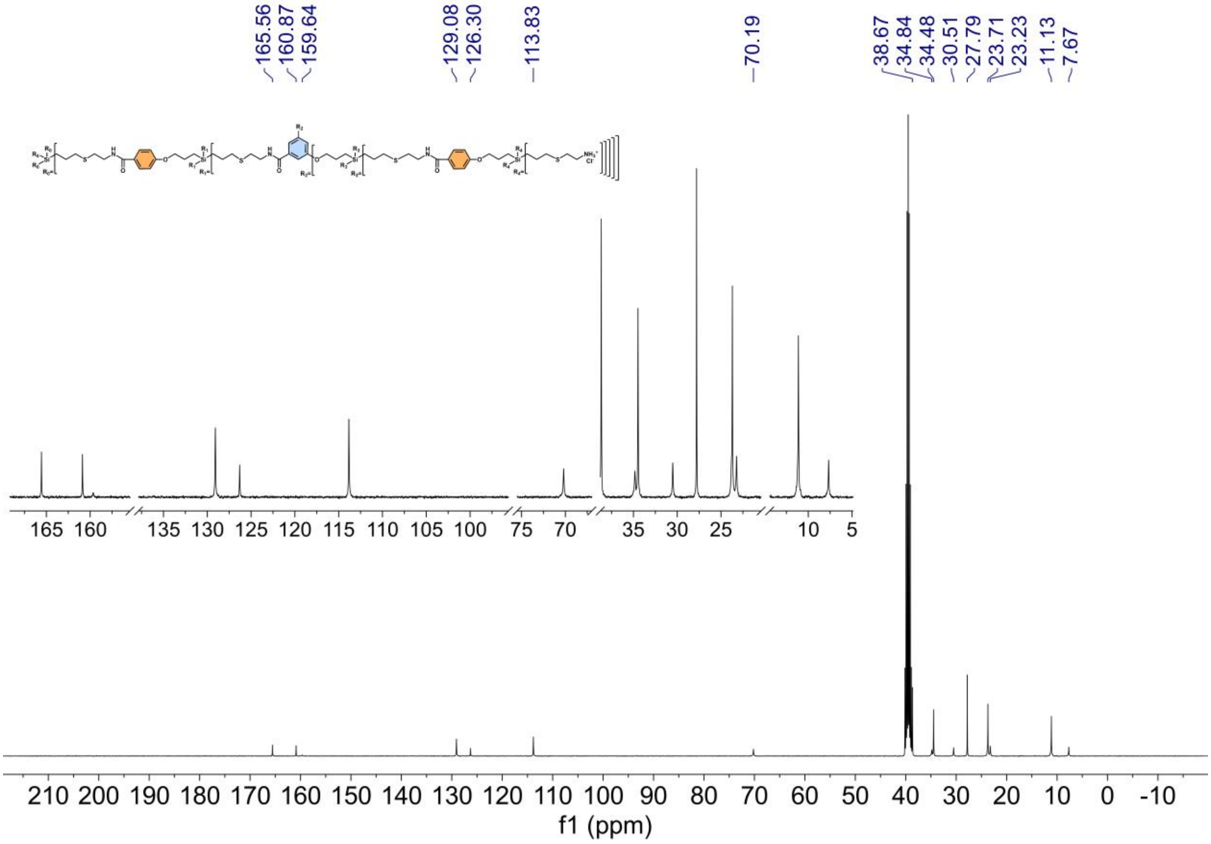

Figure S192: ^{13}C {^{1}H} NMR (101 MHz, DMSO-d_6) **G_3-3-6-3-N**

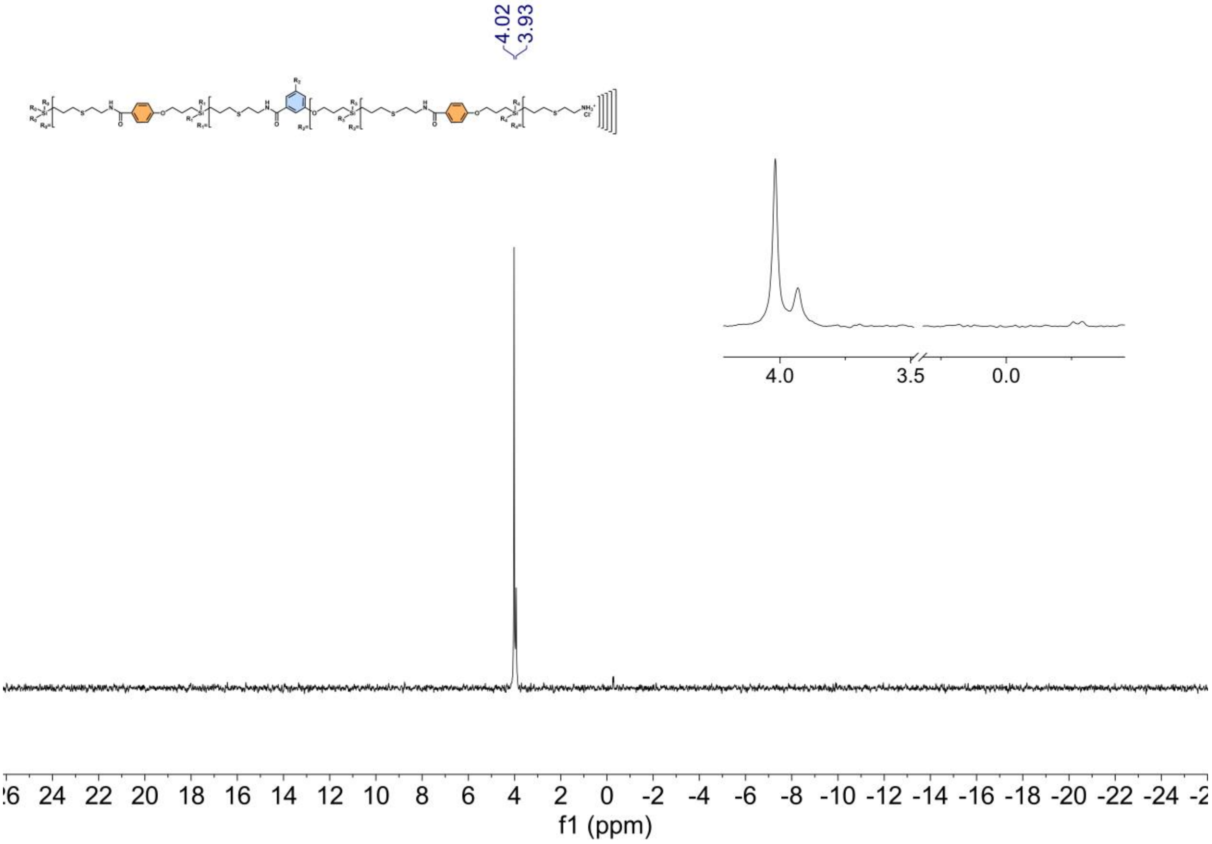

Figure S193: $^{29}Si\ \{^{1}H\}$ NMR (79 MHz, DMSO-d_6) **G_3-3-6-3-N**

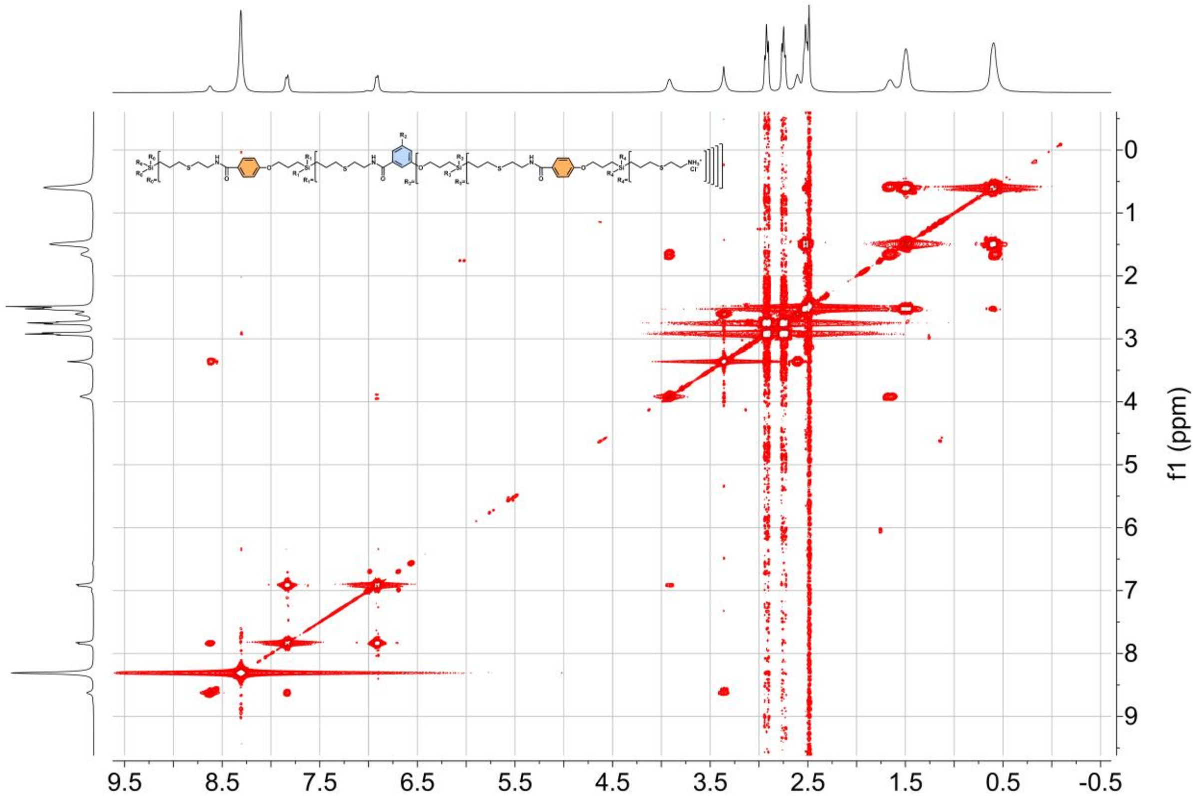

Figure S194: ^{1}H-^{1}H COSY NMR (DMSO-d_6) **G_3-3-6-3-N**

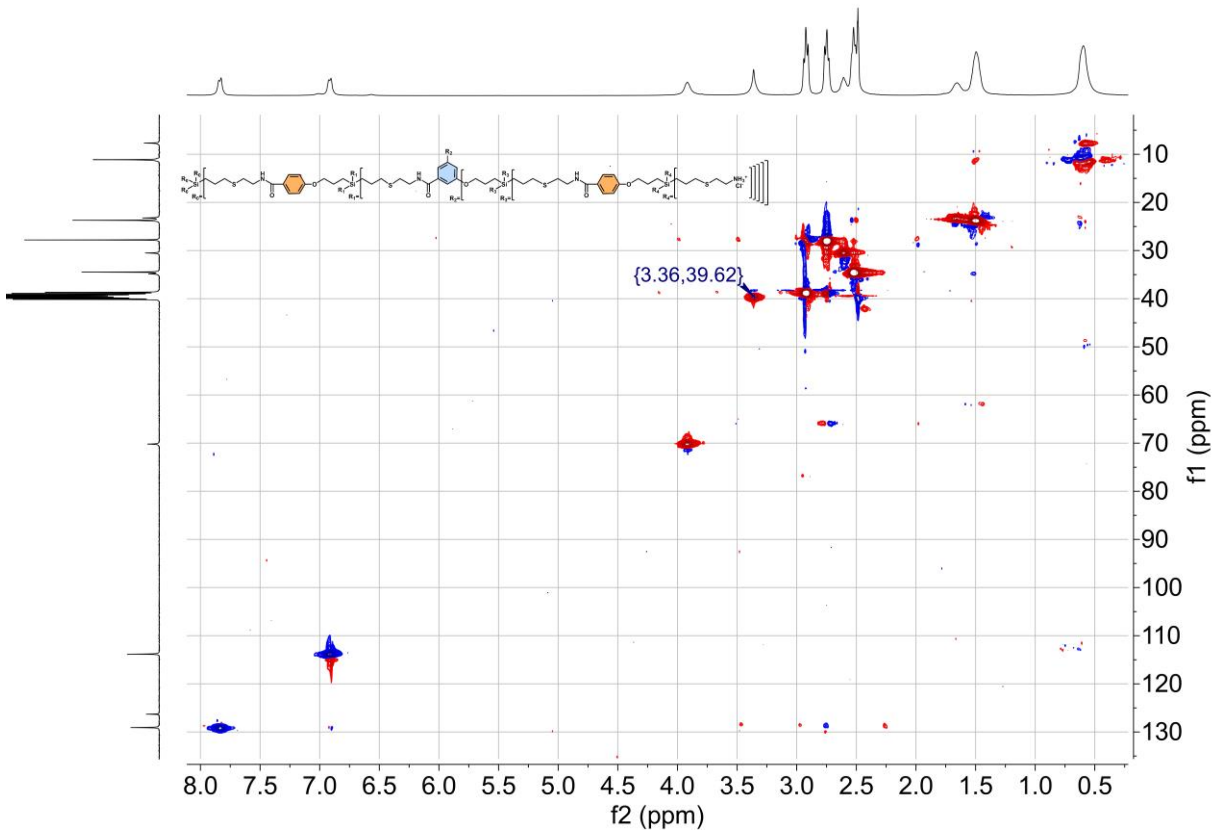

Figure S195: ^{1}H-^{13}C HSQC NMR (DMSO-d_6) **G_3-3-6-3-N**

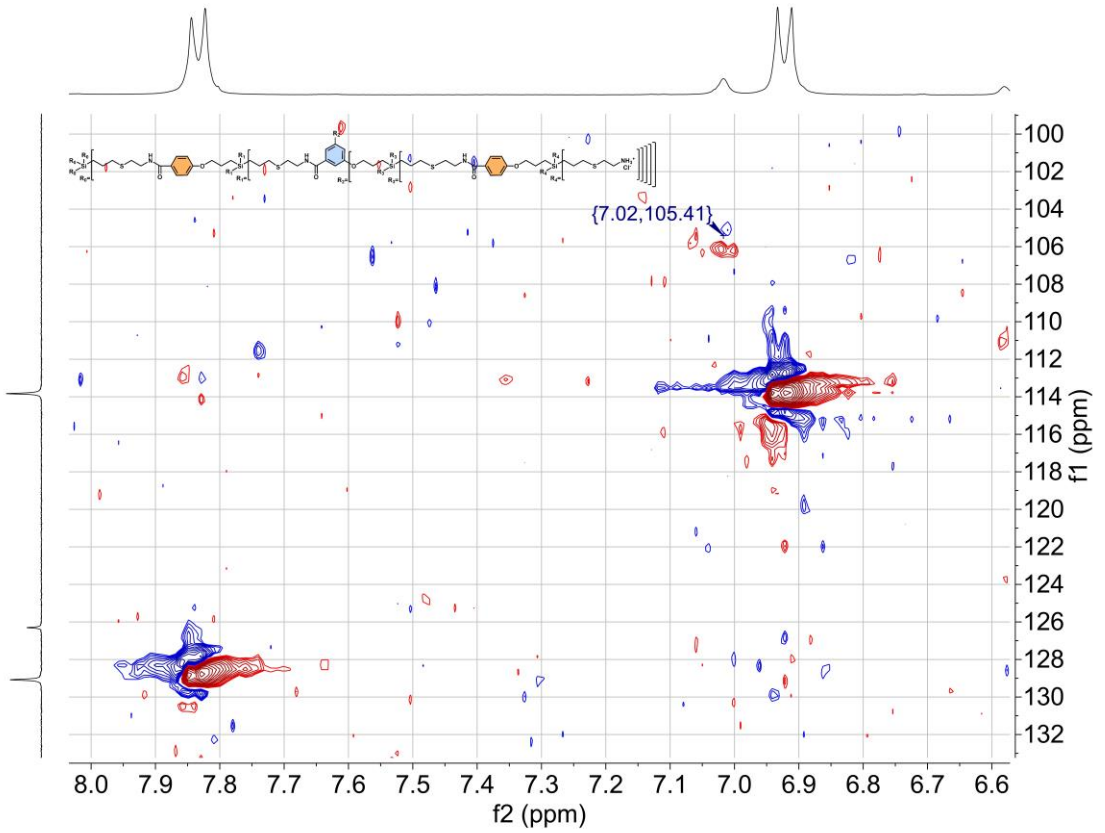

Figure S196: ^{1}H-^{13}C HSQC NMR (100°C, DMSO-d_6) **G_3-3-6-3-N**

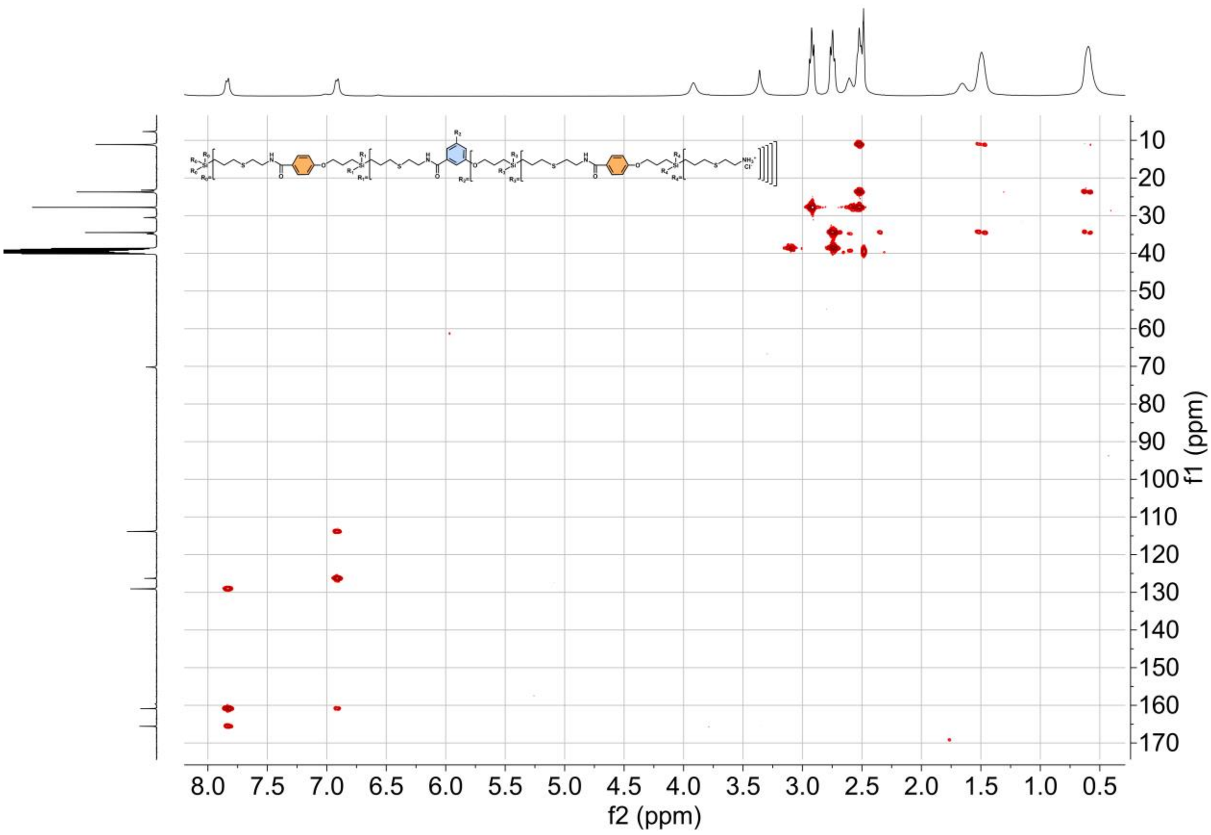

Figure S197: ^{1}H-^{13}C HMBC NMR (DMSO-d_6) **G_3-3-6-3-N**

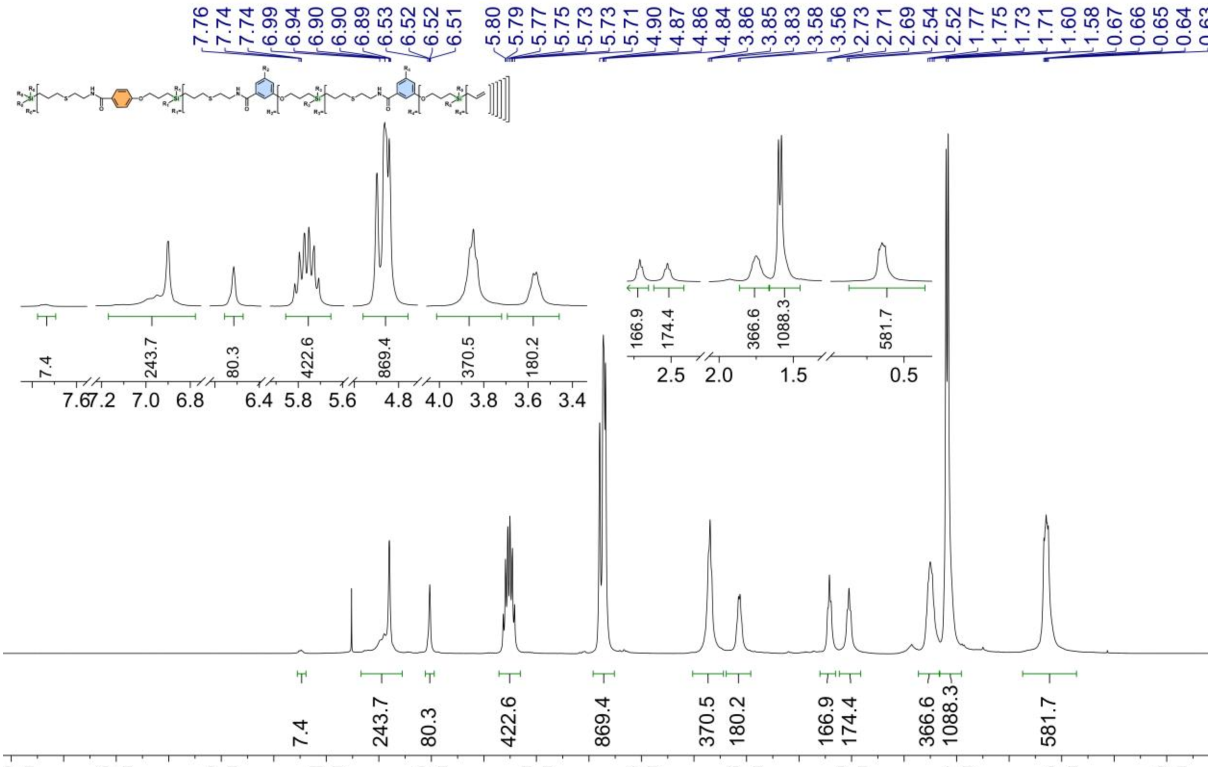

Figure S198: ^{1}H NMR (400 MHz, $CDCl_3$) **G_3-3-6-6-A**

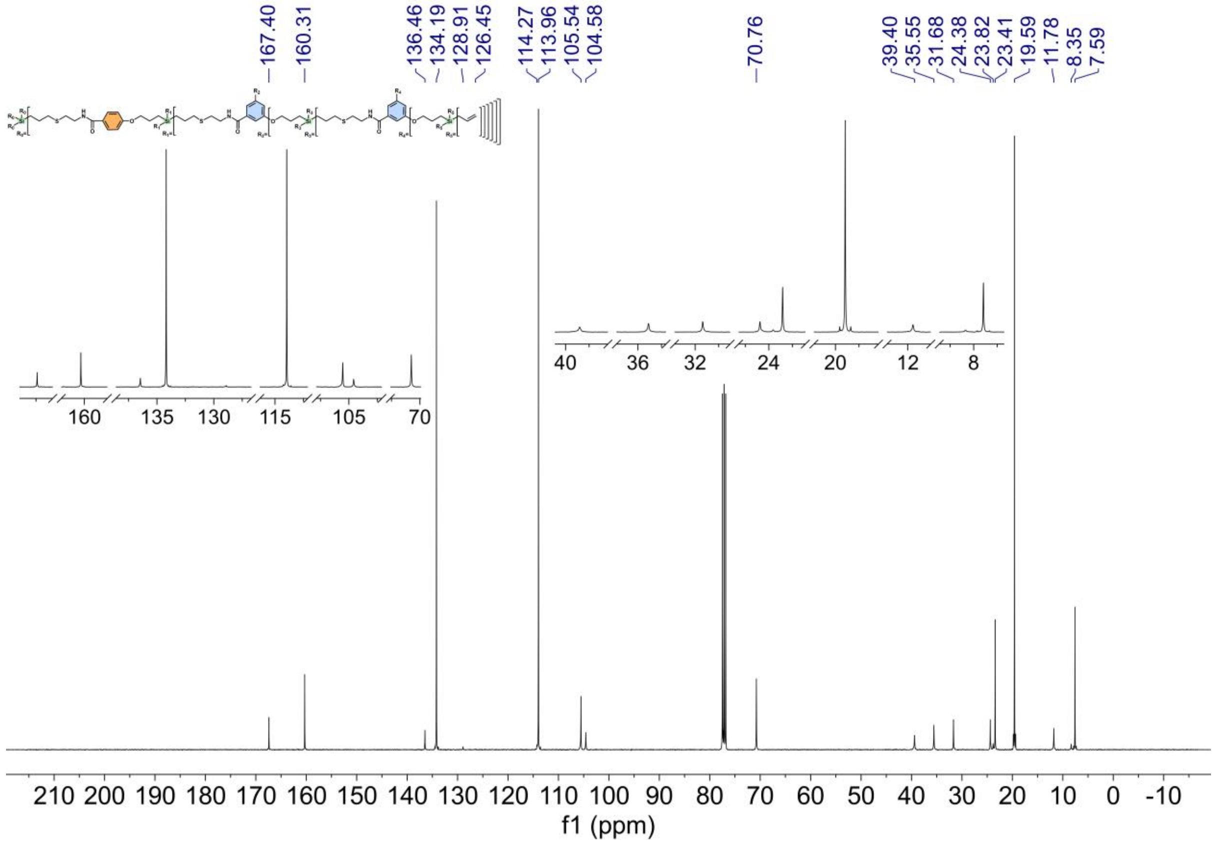

Figure S199: ^{13}C {^{1}H} NMR (101 MHz, $CDCl_3$) **G_3-3-6-6-A**

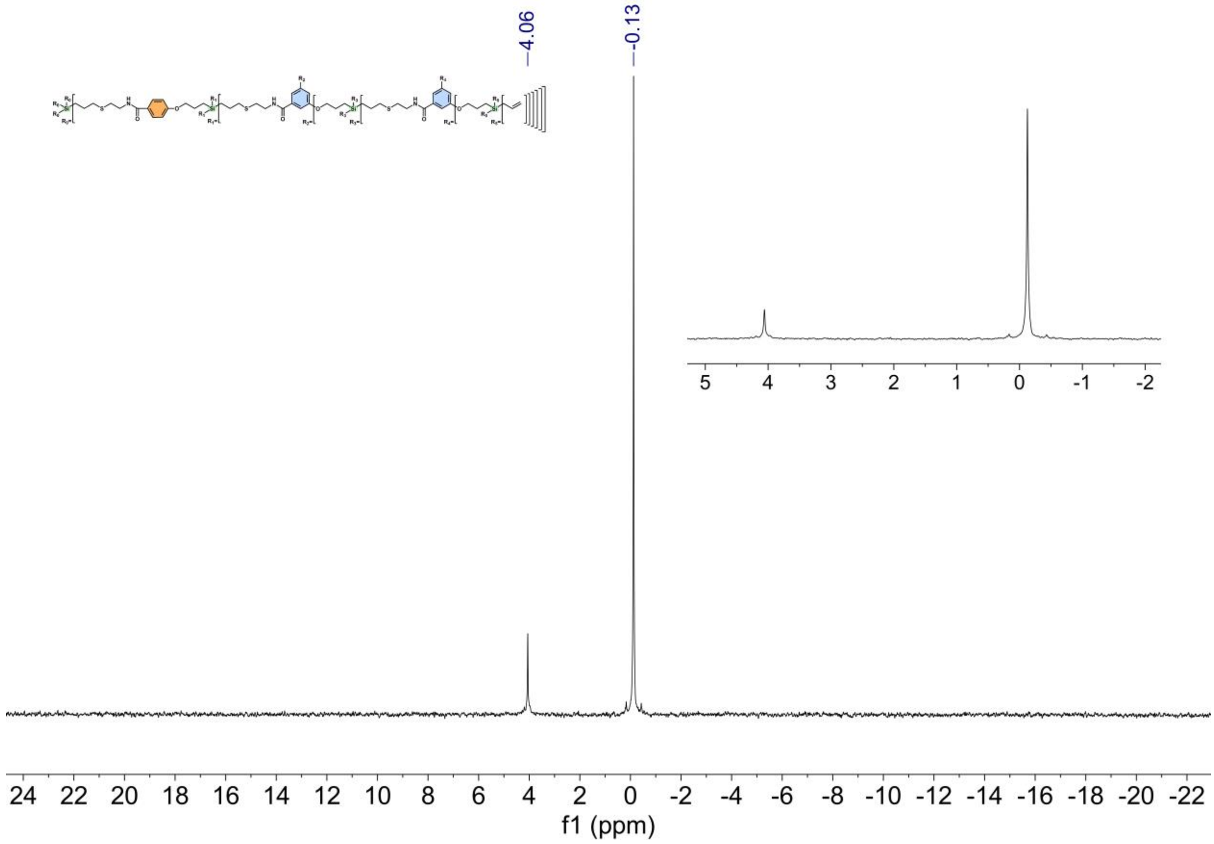

Figure S200: ^{29}Si {^{1}H} NMR (79 MHz, $CDCl_3$) **G_3-3-6-6-A**

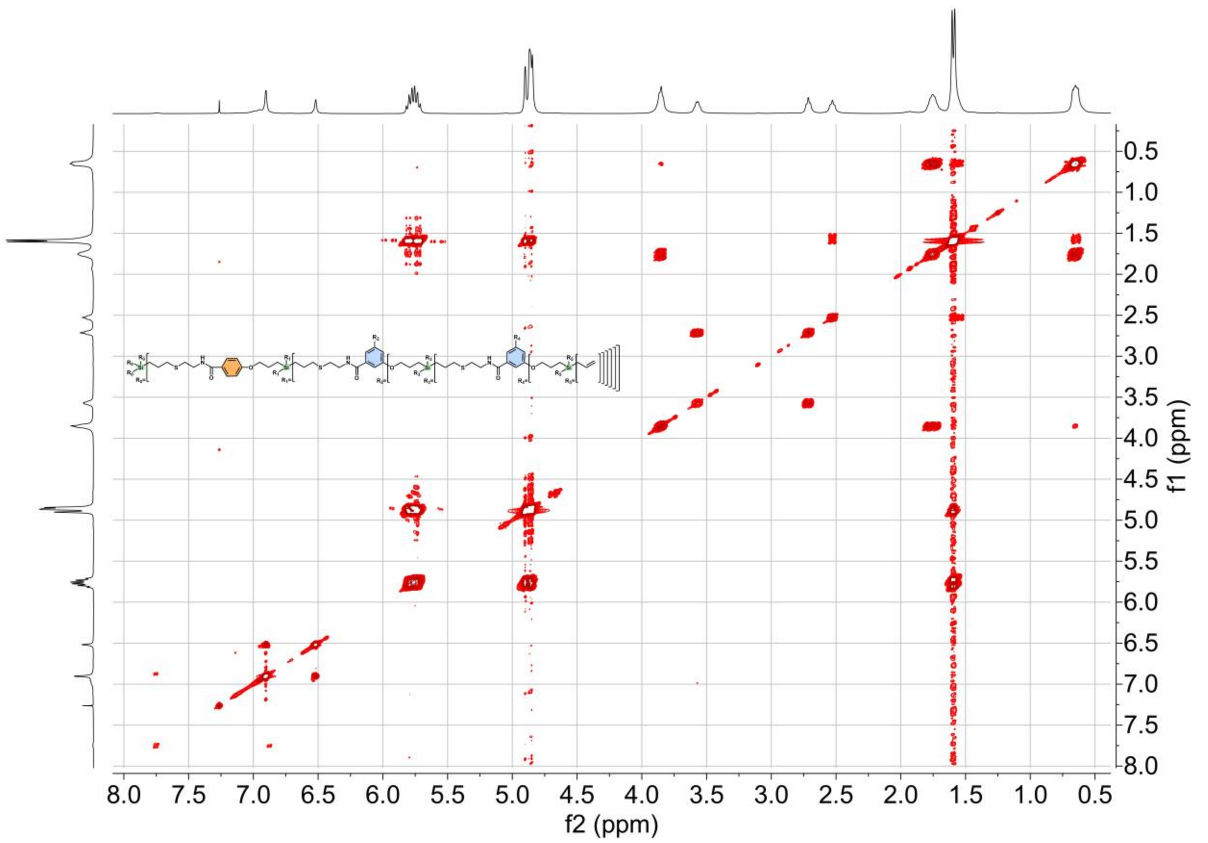

Figure S201: ^{1}H-^{1}H COSY NMR ($CDCl_3$) **G_3-3-6-6-A**

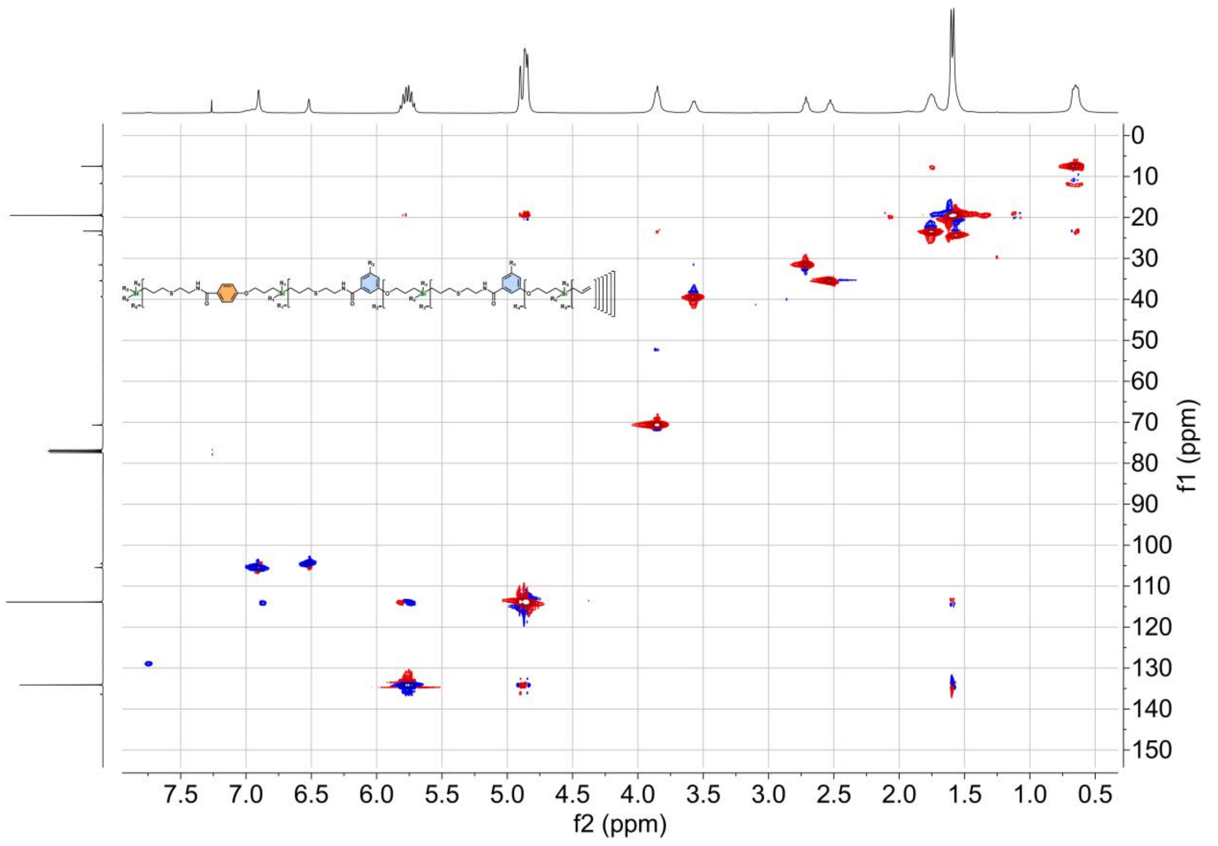

Figure S202: ^{1}H-^{13}C HSQC NMR ($CDCl_3$) **G_3-3-6-6-A**

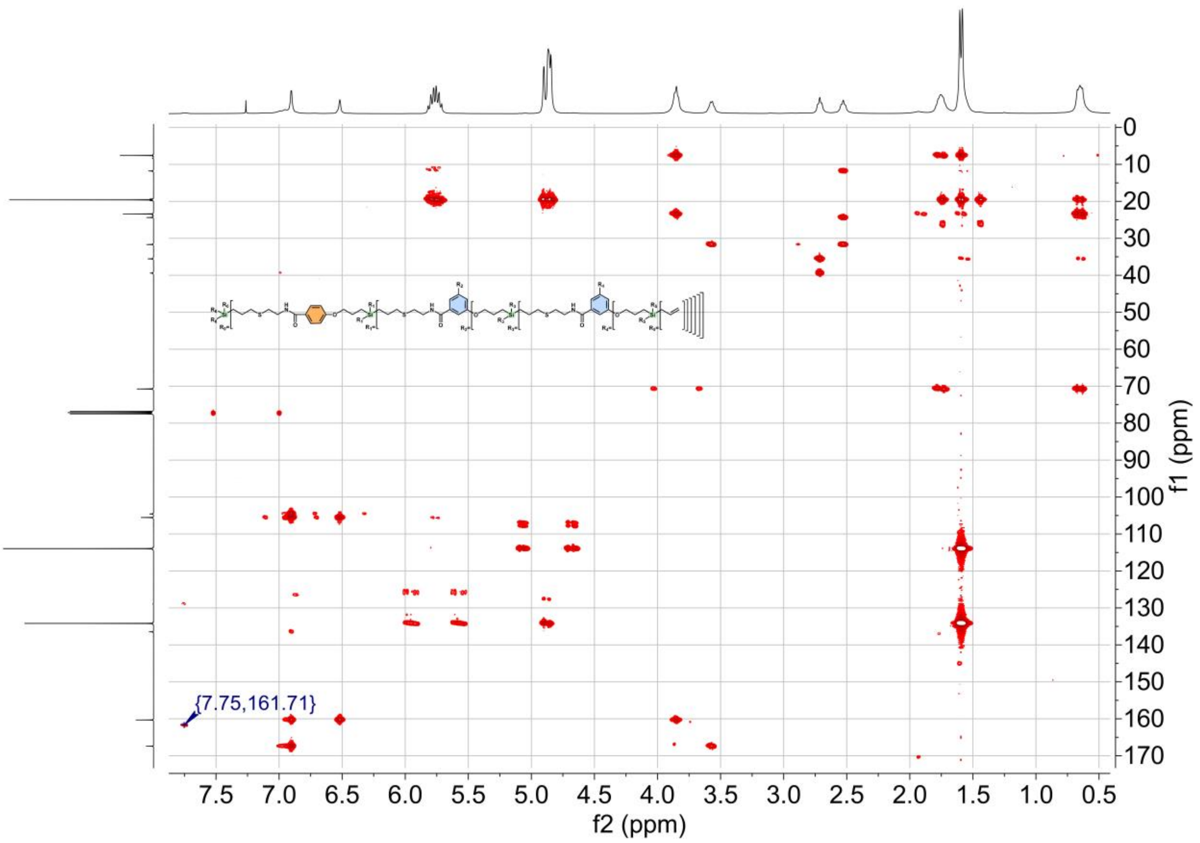

Figure S203: ^{1}H-^{13}C HMBC NMR ($CDCl_3$) **G_3-3-6-6-A**

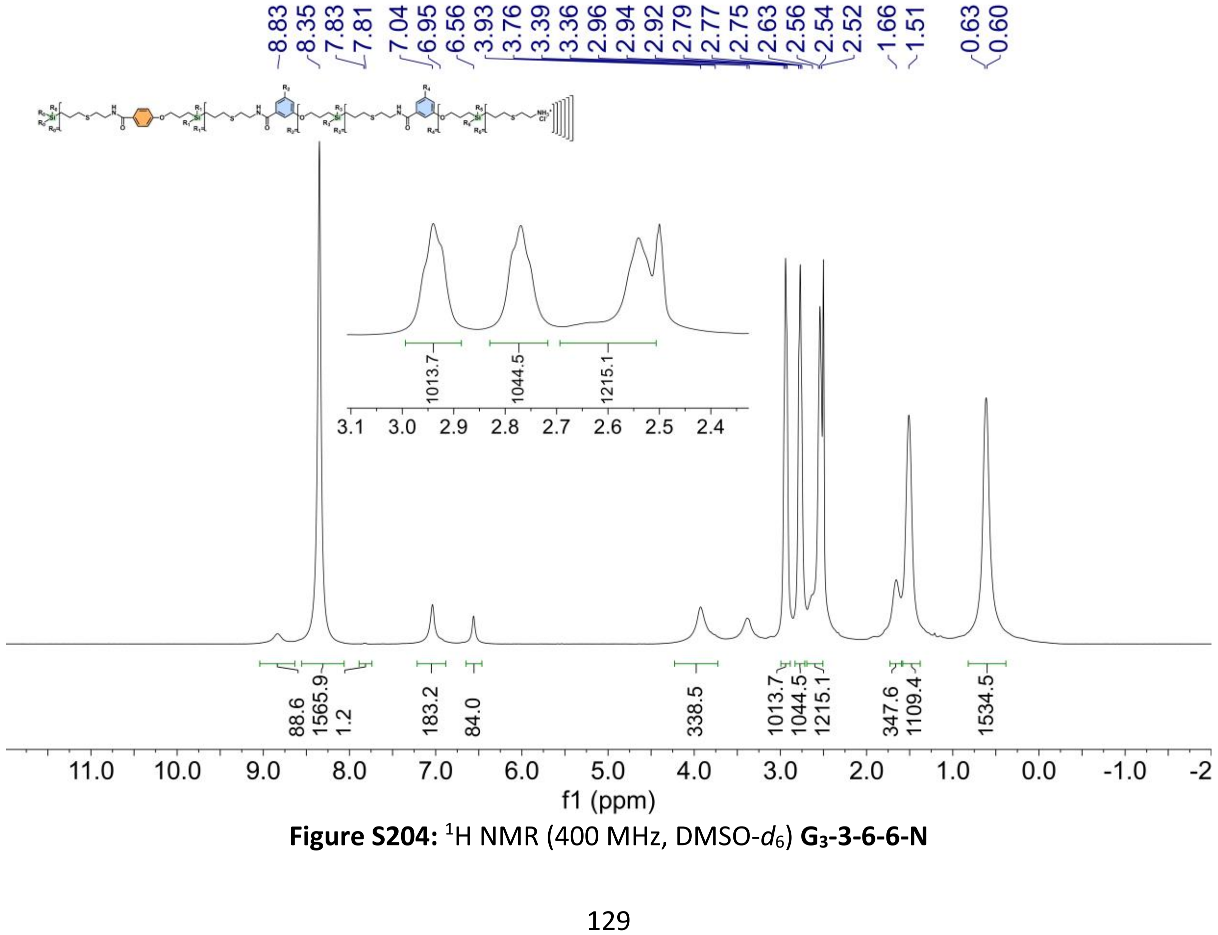

Figure S204: ^{1}H NMR (400 MHz, DMSO-d_6) **G_3-3-6-6-N**

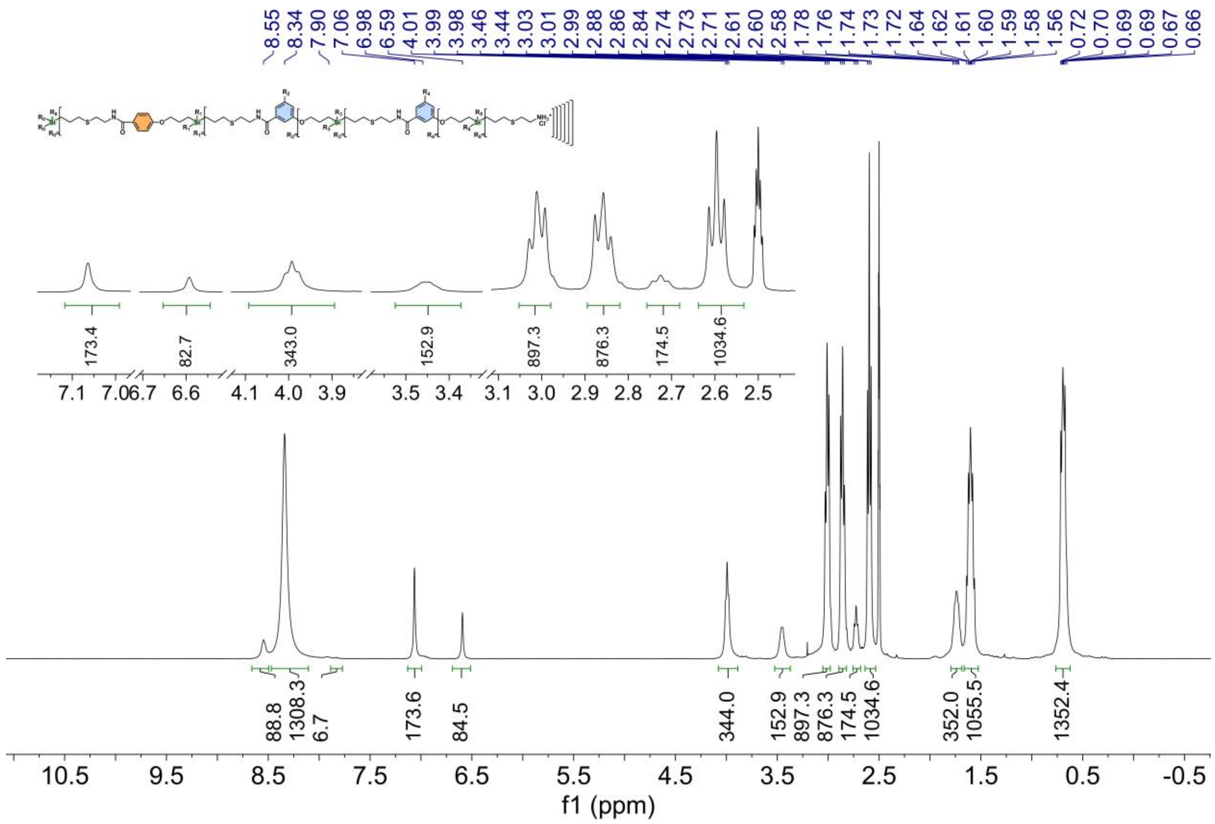

Figure S205: ^{1}H NMR (400 MHz, 100°C, DMSO-d_6) **G_3-3-6-6-N**

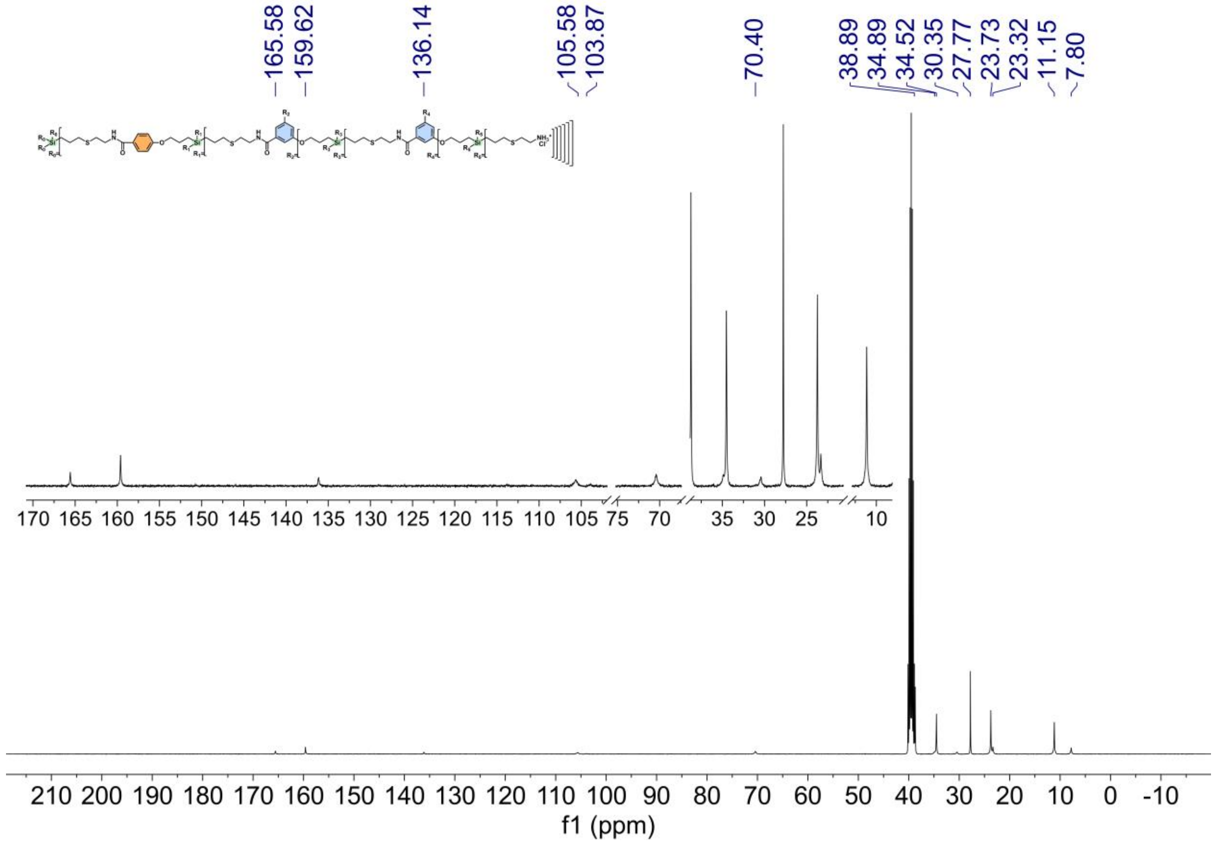

Figure S206: ^{13}C {^{1}H} NMR (101 MHz, DMSO-d_6) **G_3-3-6-6-N**

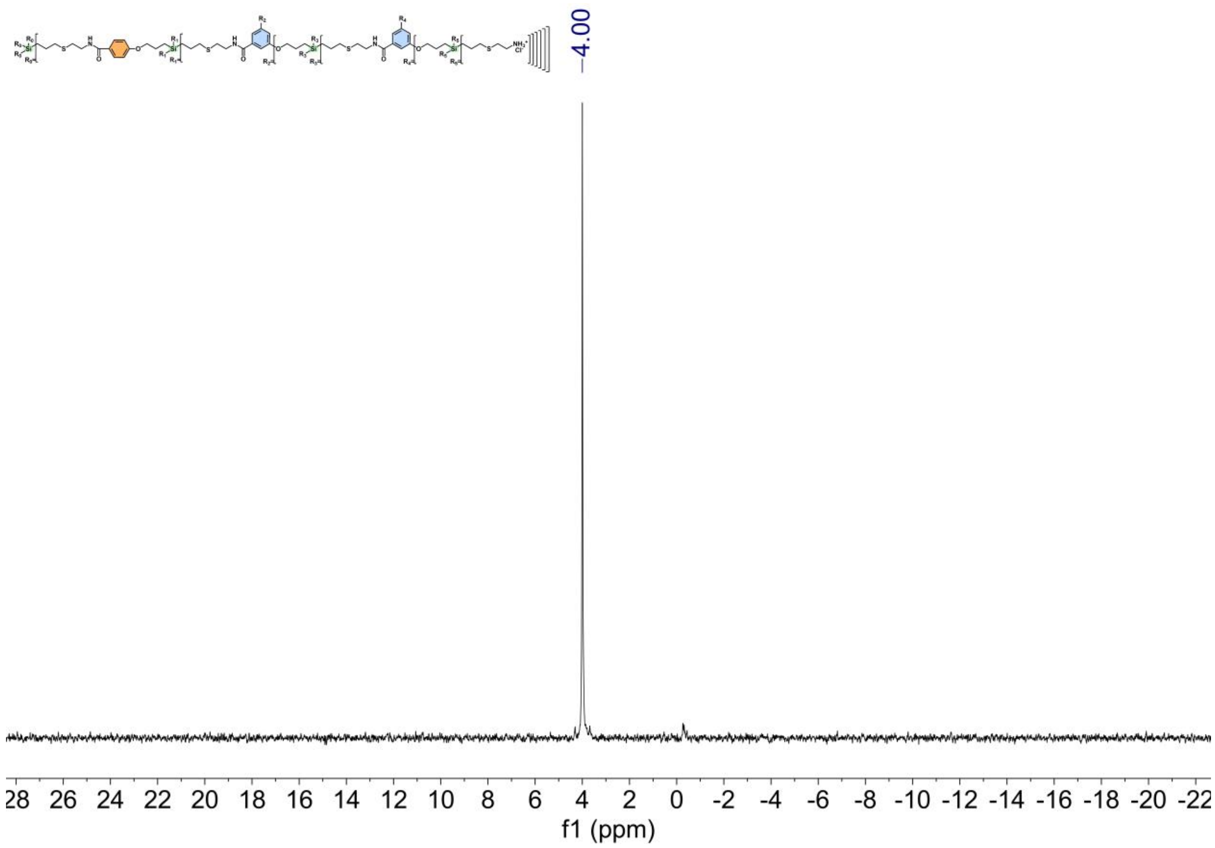

Figure S207: ^{29}Si {^{1}H} NMR (79 MHz, DMSO-d_6) **G_3-3-6-6-N**

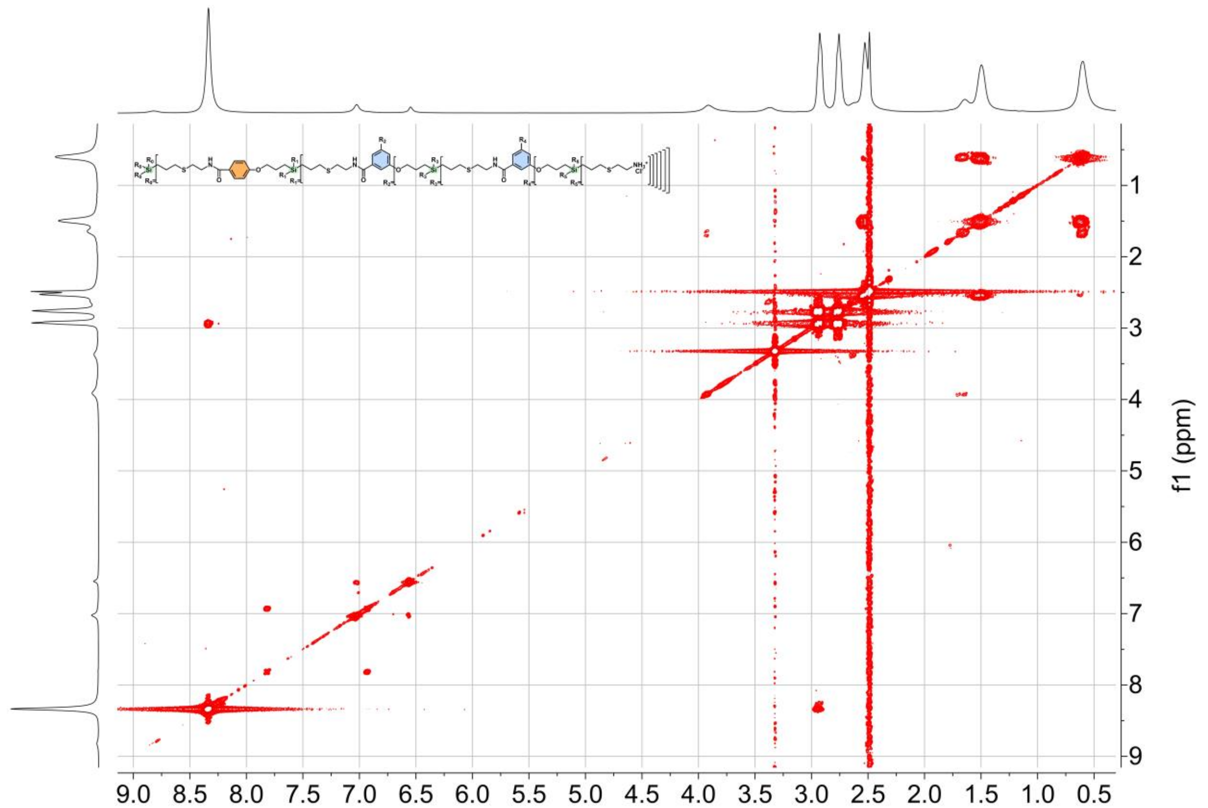

Figure S208: ^{1}H-^{1}H COSY NMR (DMSO-d_6) **G_3-3-6-6-N**

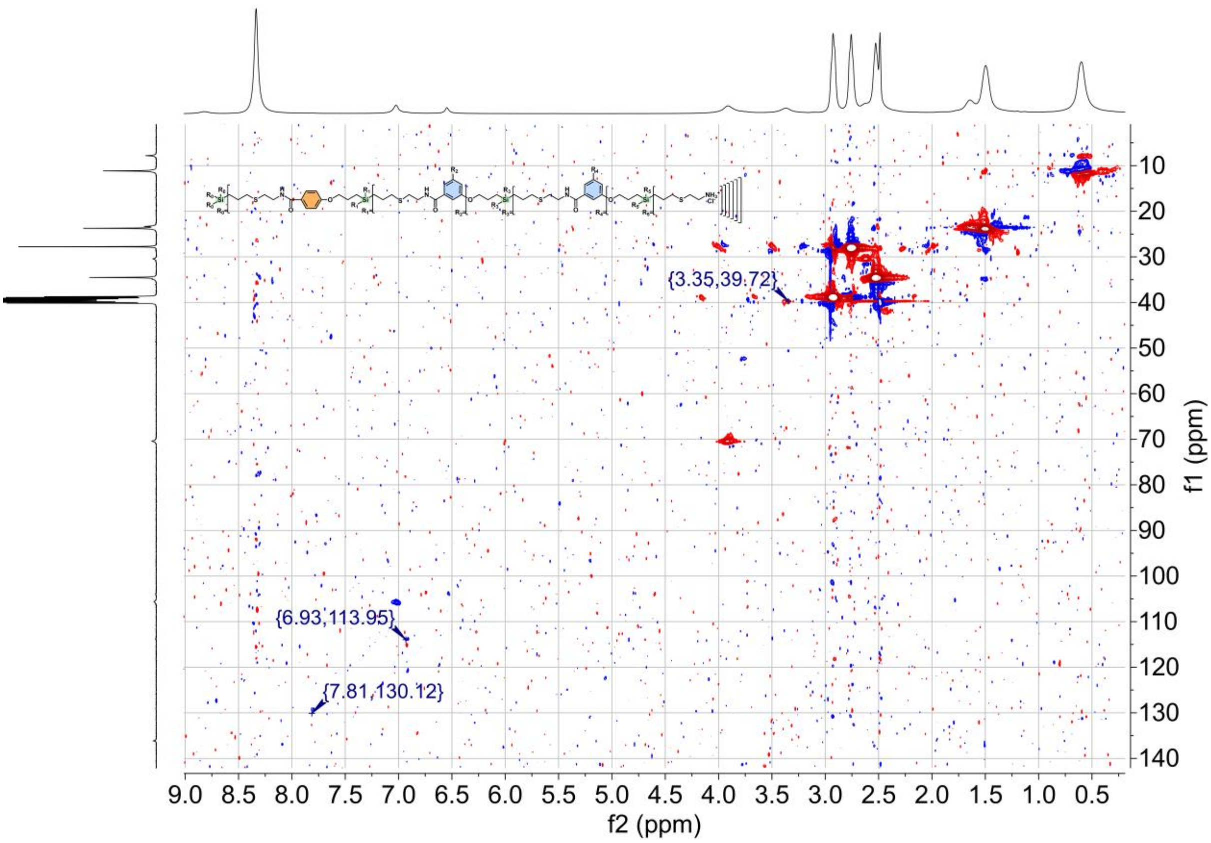

Figure S209: ^{1}H-^{13}C HSQC NMR (DMSO-d_6) **G_3-3-6-6-N**

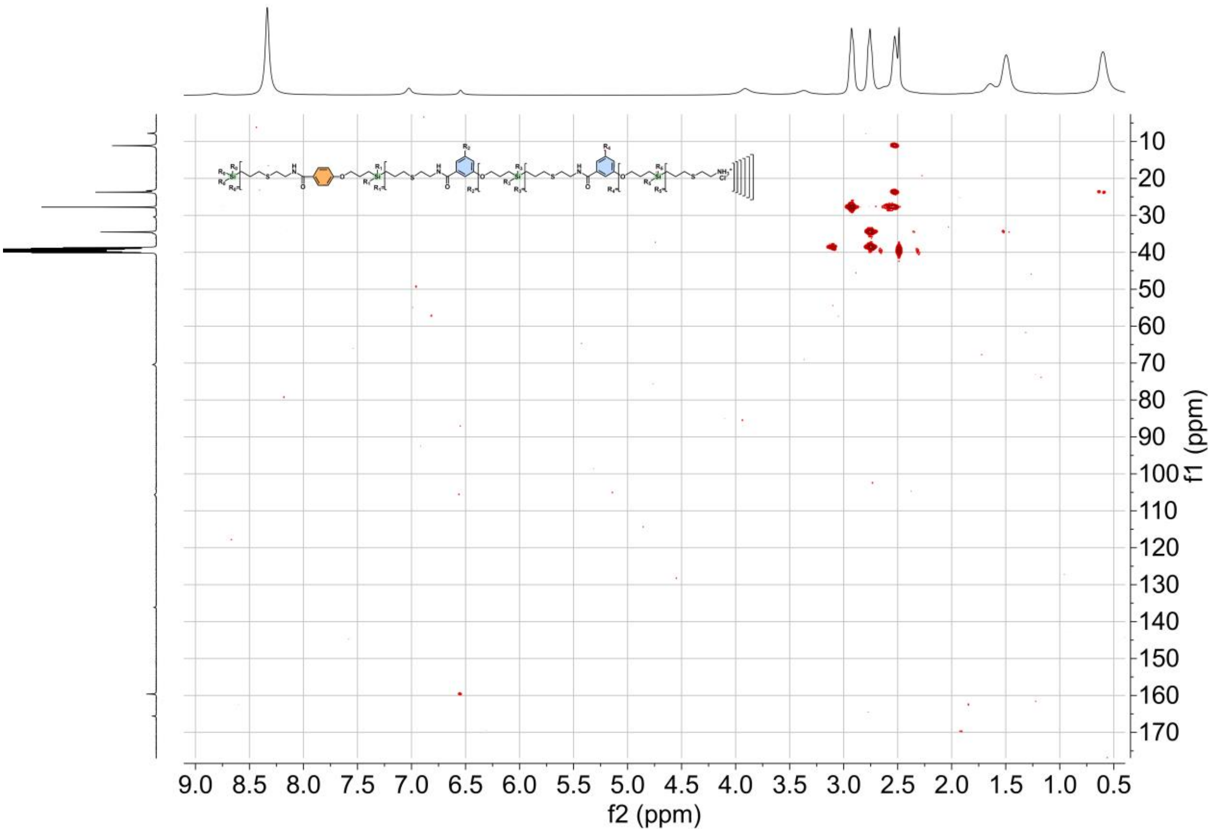

Figure S210: ^{1}H-^{13}C HMBC NMR (DMSO-d_6) **G_3-3-6-6-N**

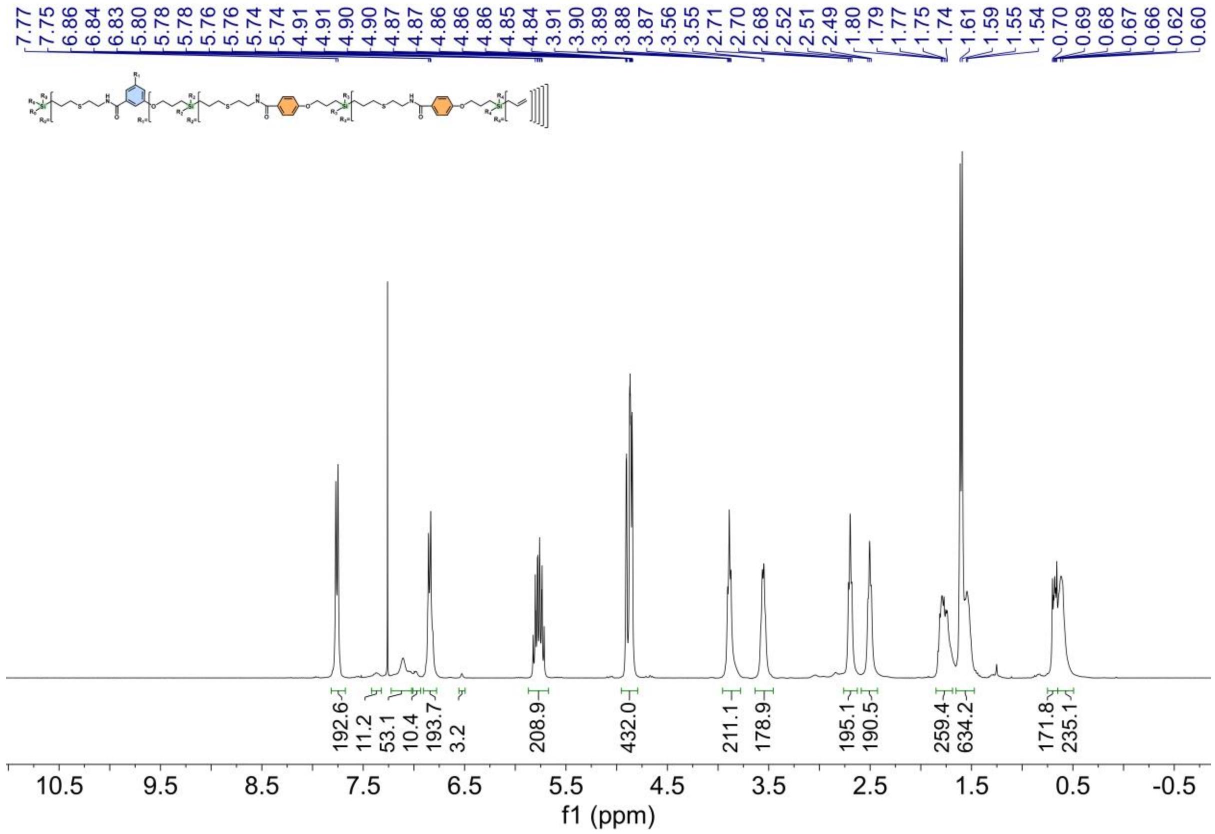

Figure S211: ^{1}H NMR (400 MHz, $CDCl_3$) **G_3-6-3-3-A**

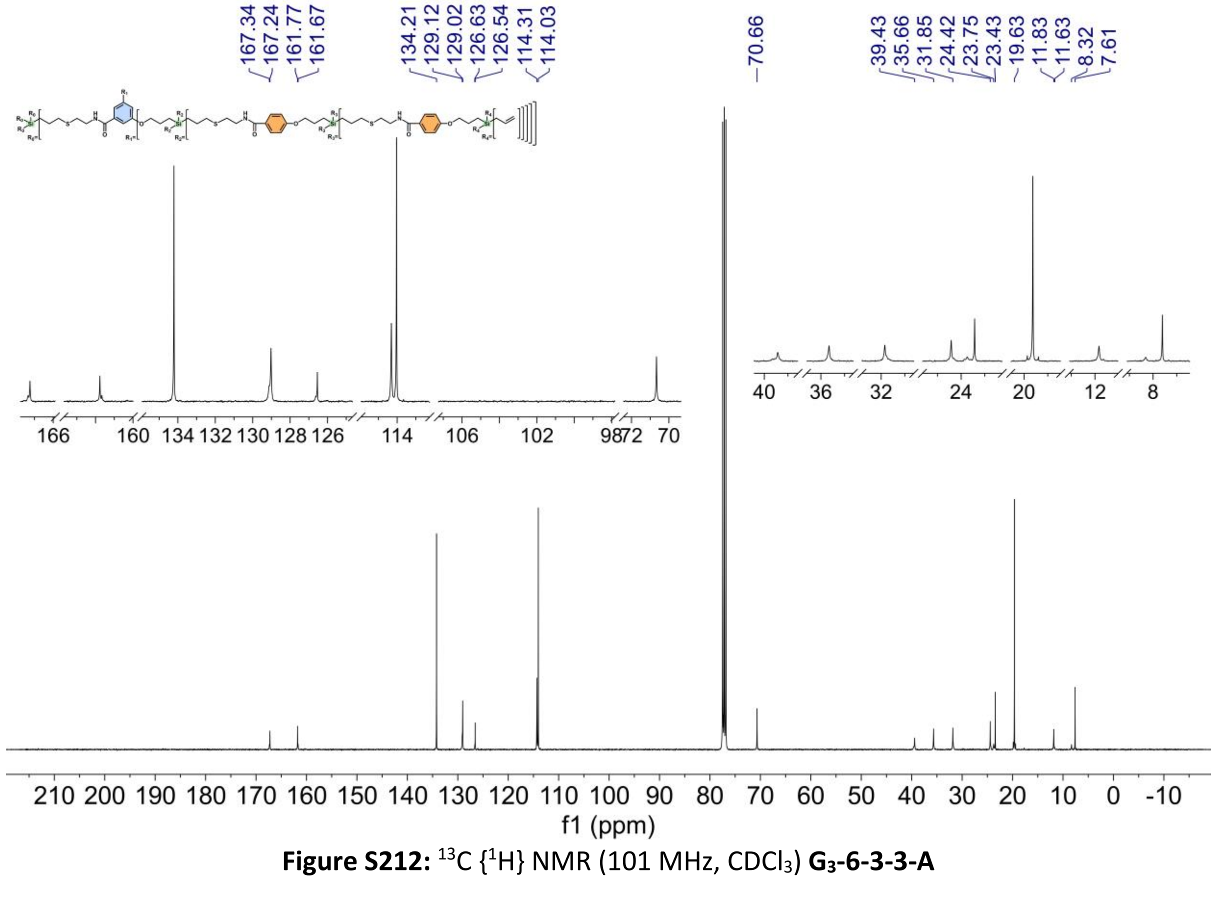

Figure S212: $^{13}C\{^{1}H\}$ NMR (101 MHz, $CDCl_3$) **G_3-6-3-3-A**

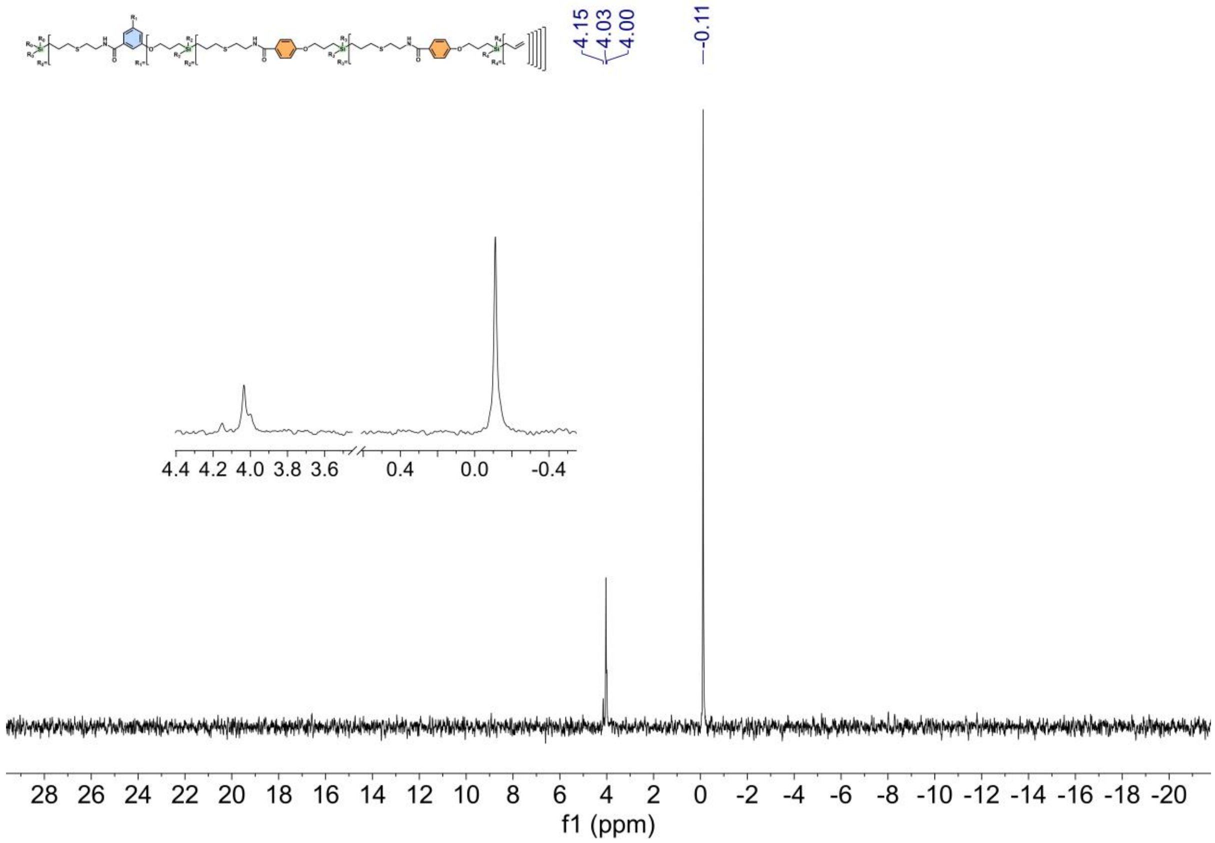

Figure S213: $^{29}Si\ \{^{1}H\}$ NMR (79 MHz, $CDCl_3$) **G_3-6-3-3-A**

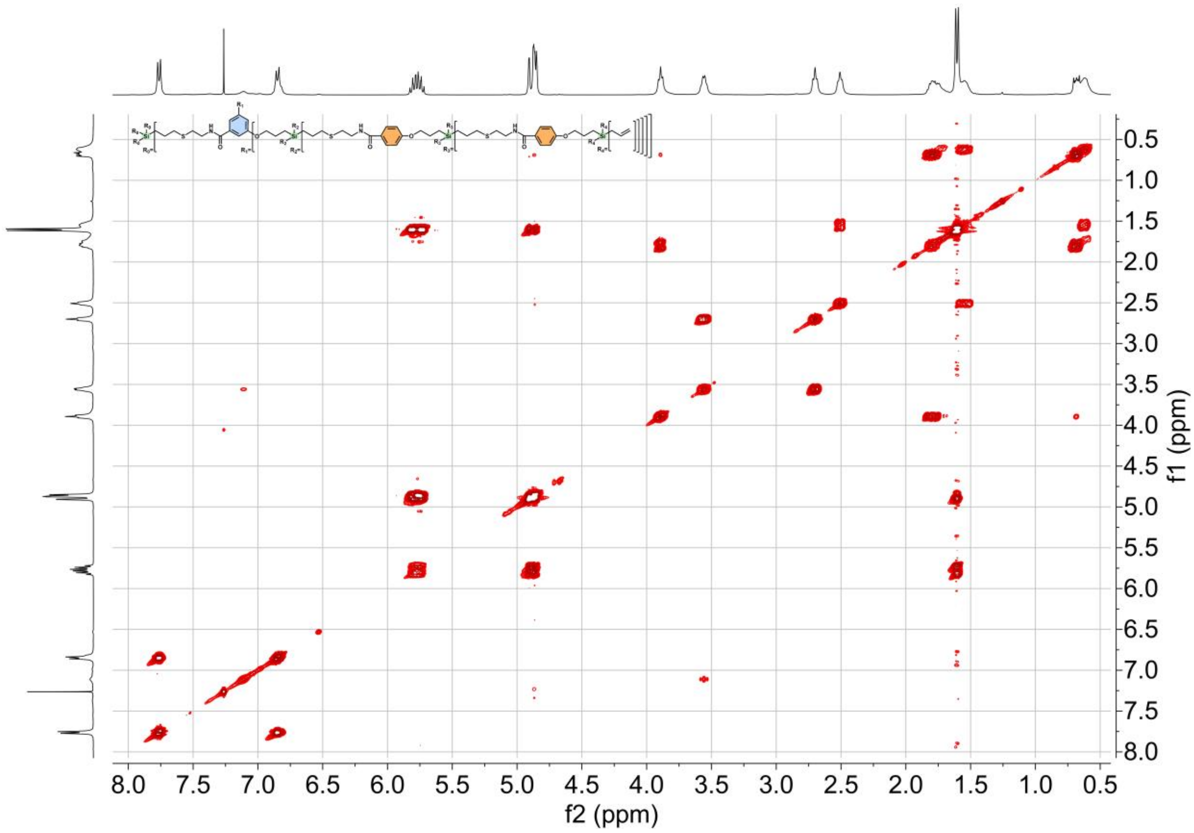

Figure S214: ^{1}H-^{1}H COSY NMR ($CDCl_3$) **G_3-6-3-3-A**

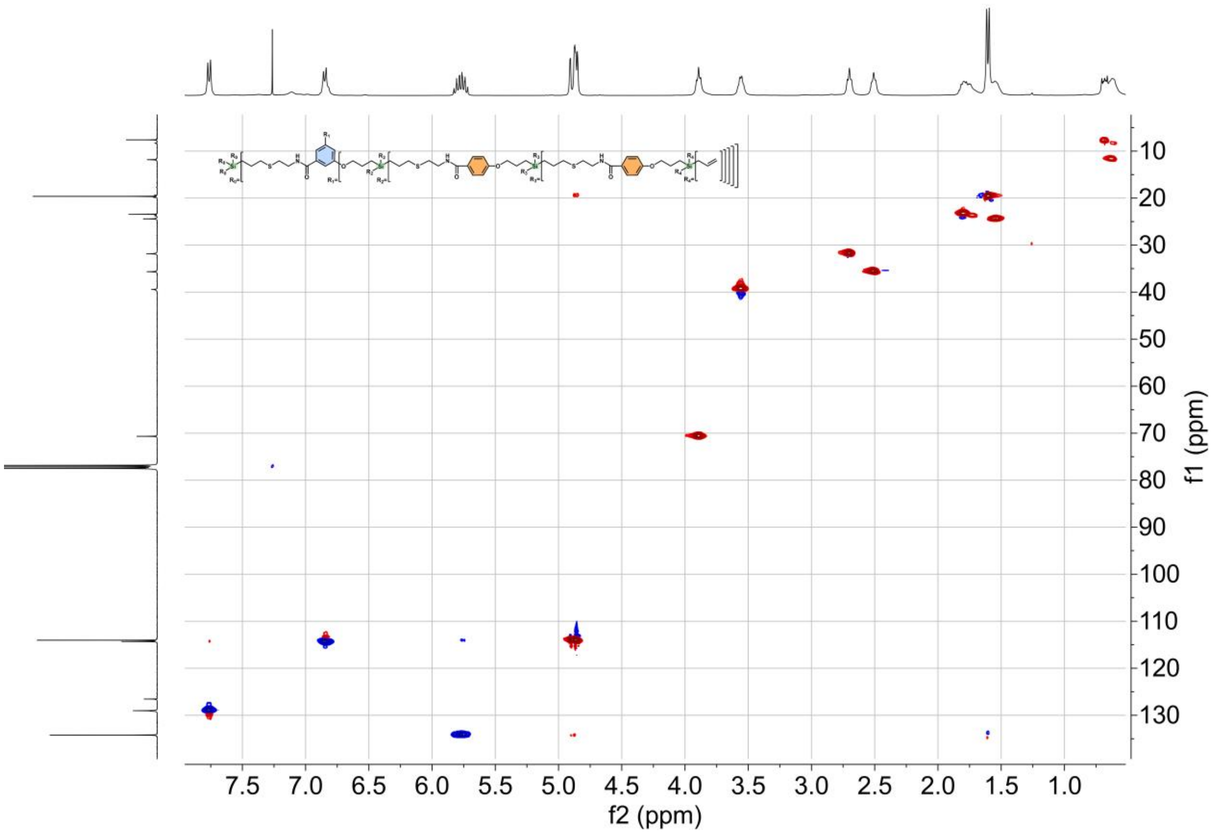

Figure S215: ^{1}H-^{13}C HSQC NMR ($CDCl_3$) **G_3-6-3-3-A**

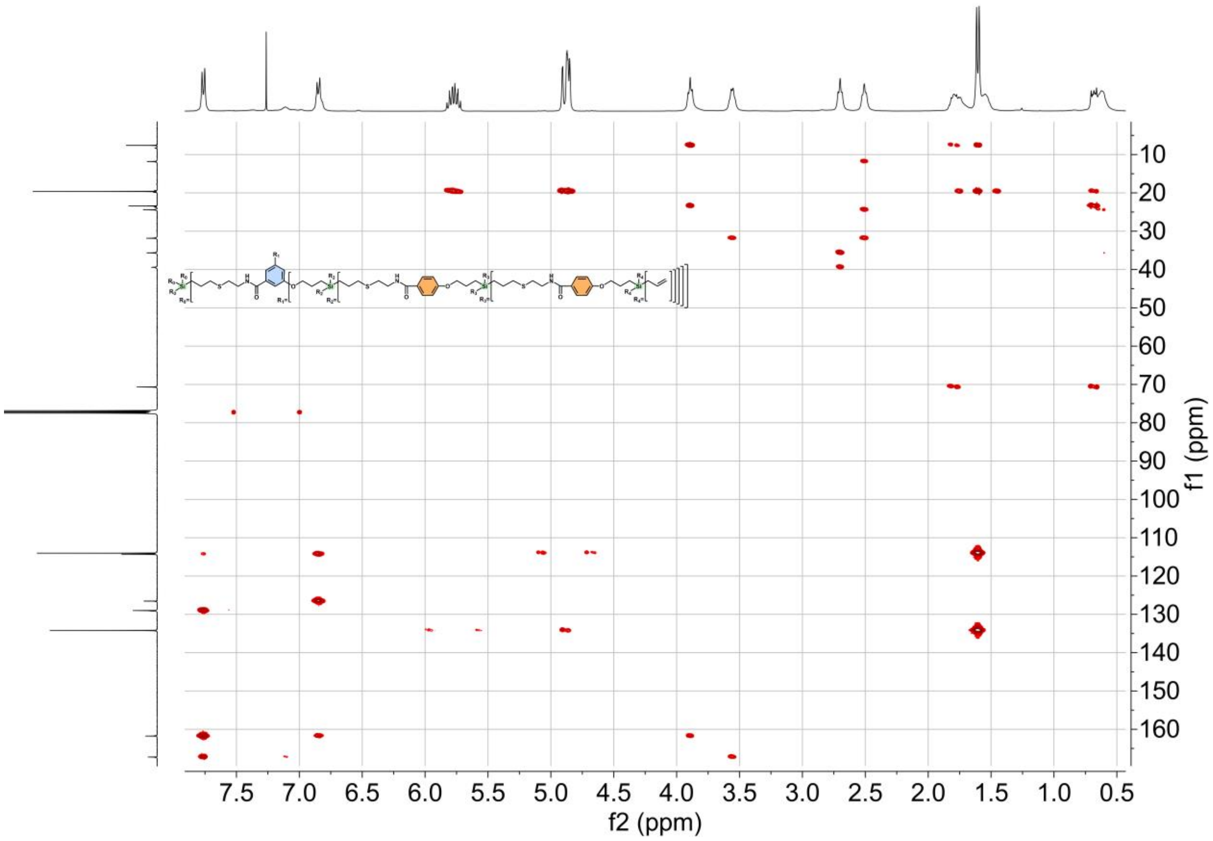

Figure S216: ^{1}H-^{13}C HMBC NMR ($CDCl_3$) **G_3-6-3-3-A**

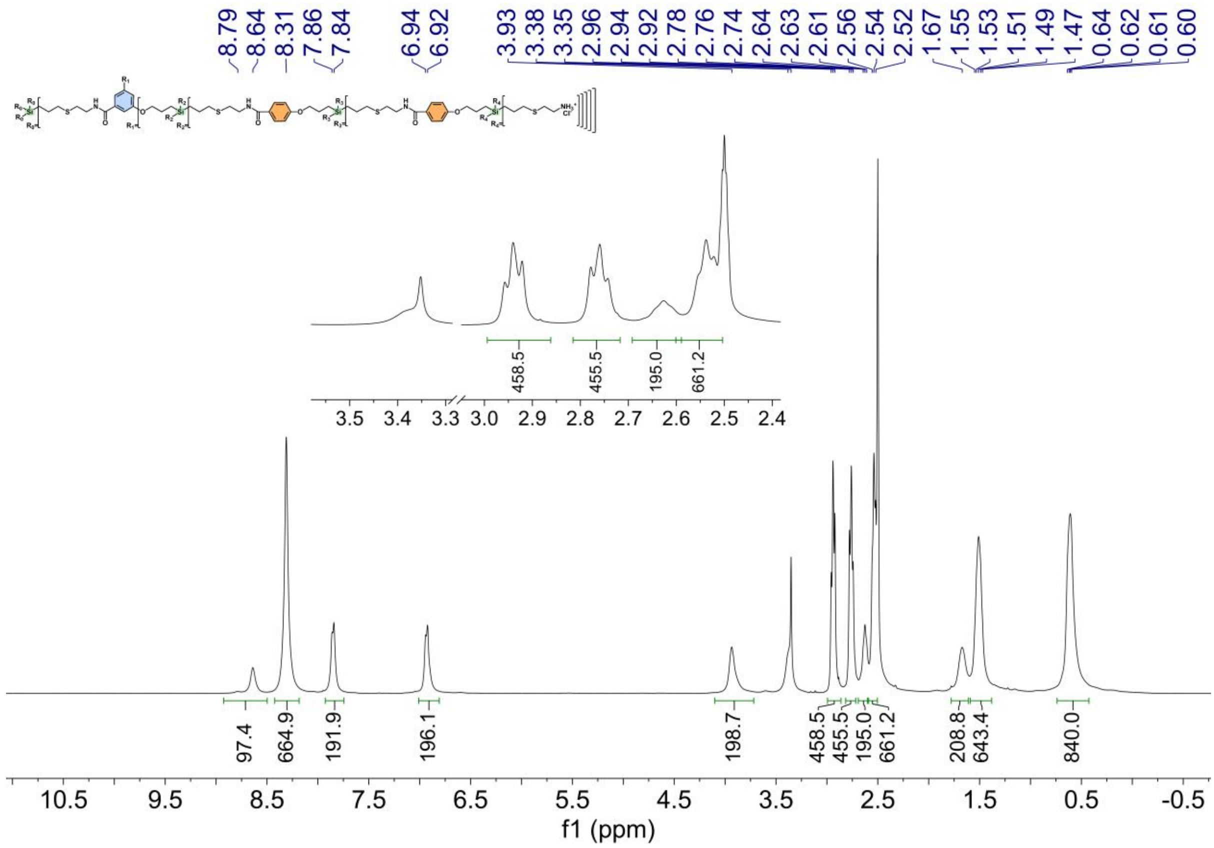

Figure S217: ^{1}H NMR (400 MHz, DMSO-d_6) **G$_3$-6-3-3-N**

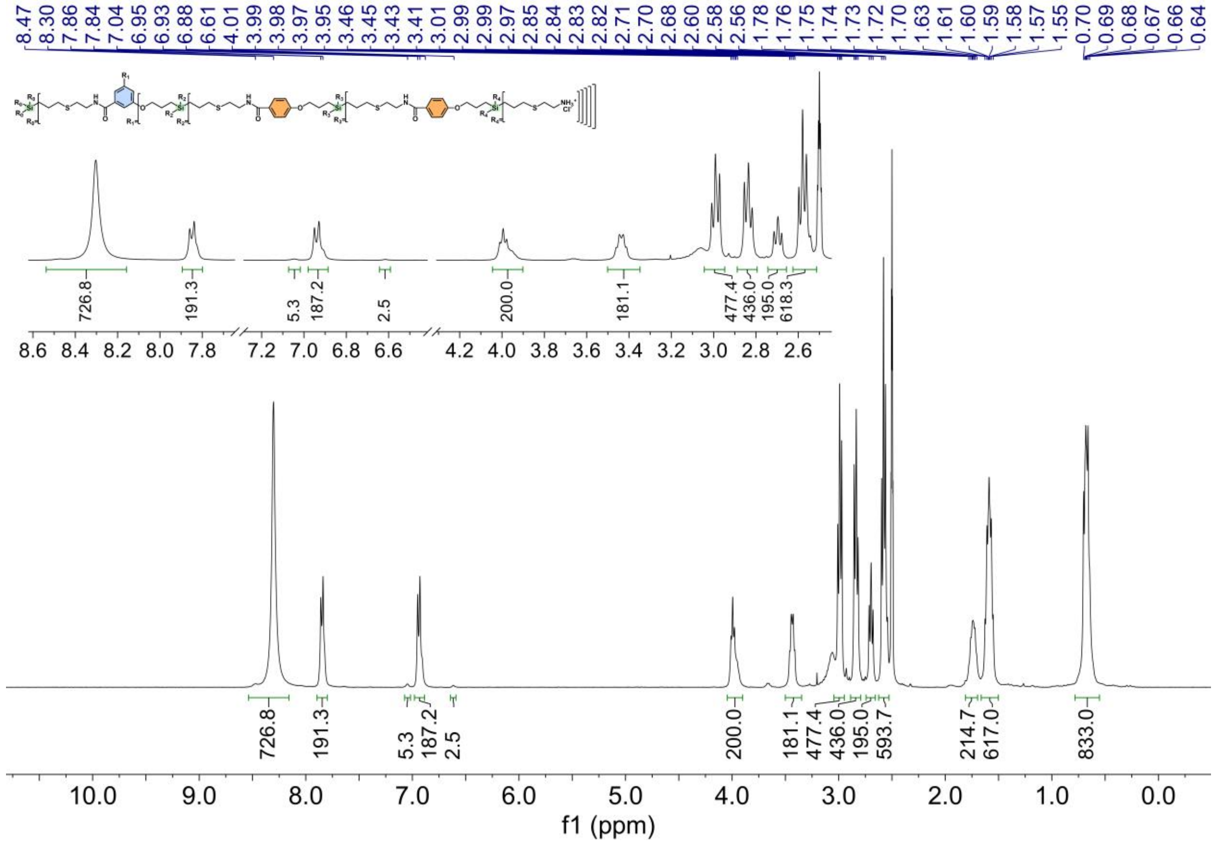

Figure S218: ^{1}H NMR (400 MHz, 100°C, DMSO-d_6) **G$_3$-6-3-3-N**

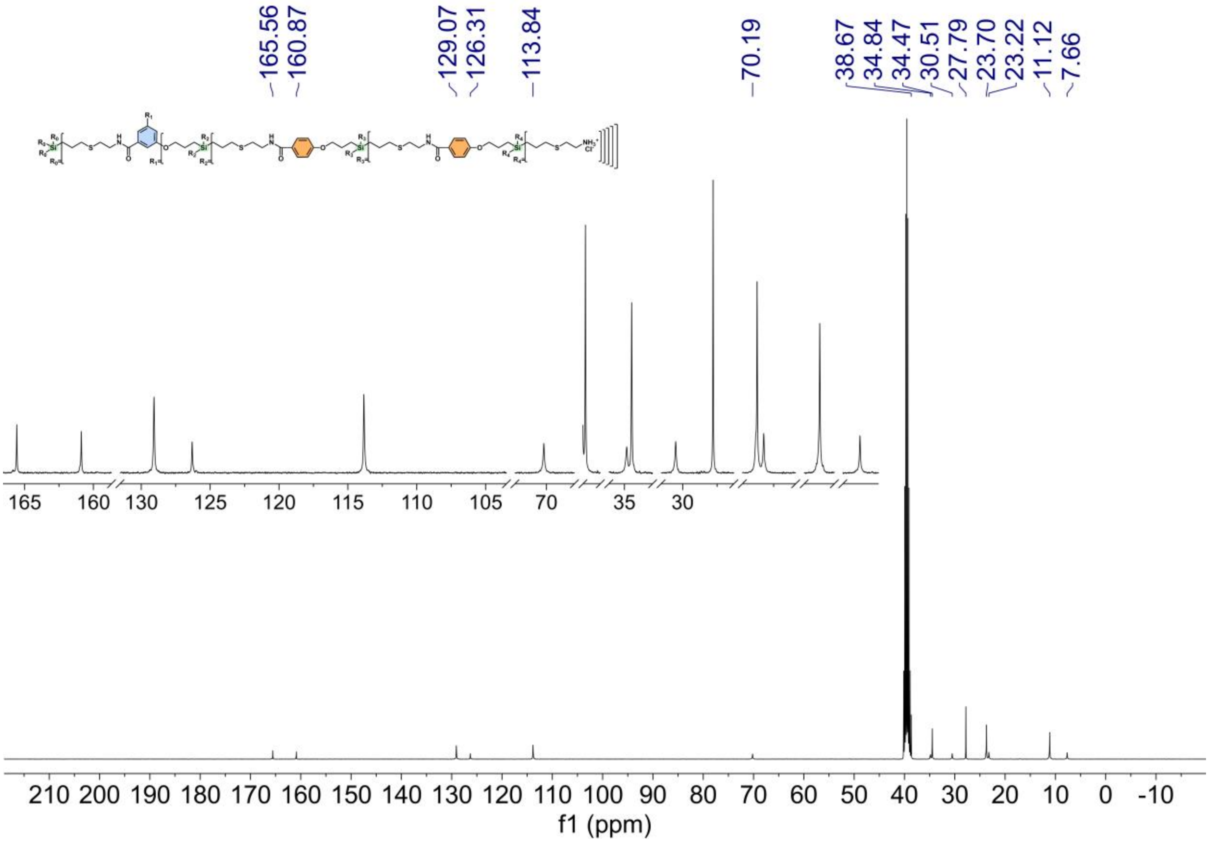

Figure S219: ^{13}C {^{1}H} NMR (101 MHz, DMSO-d_6) **G$_3$-6-3-3-N**

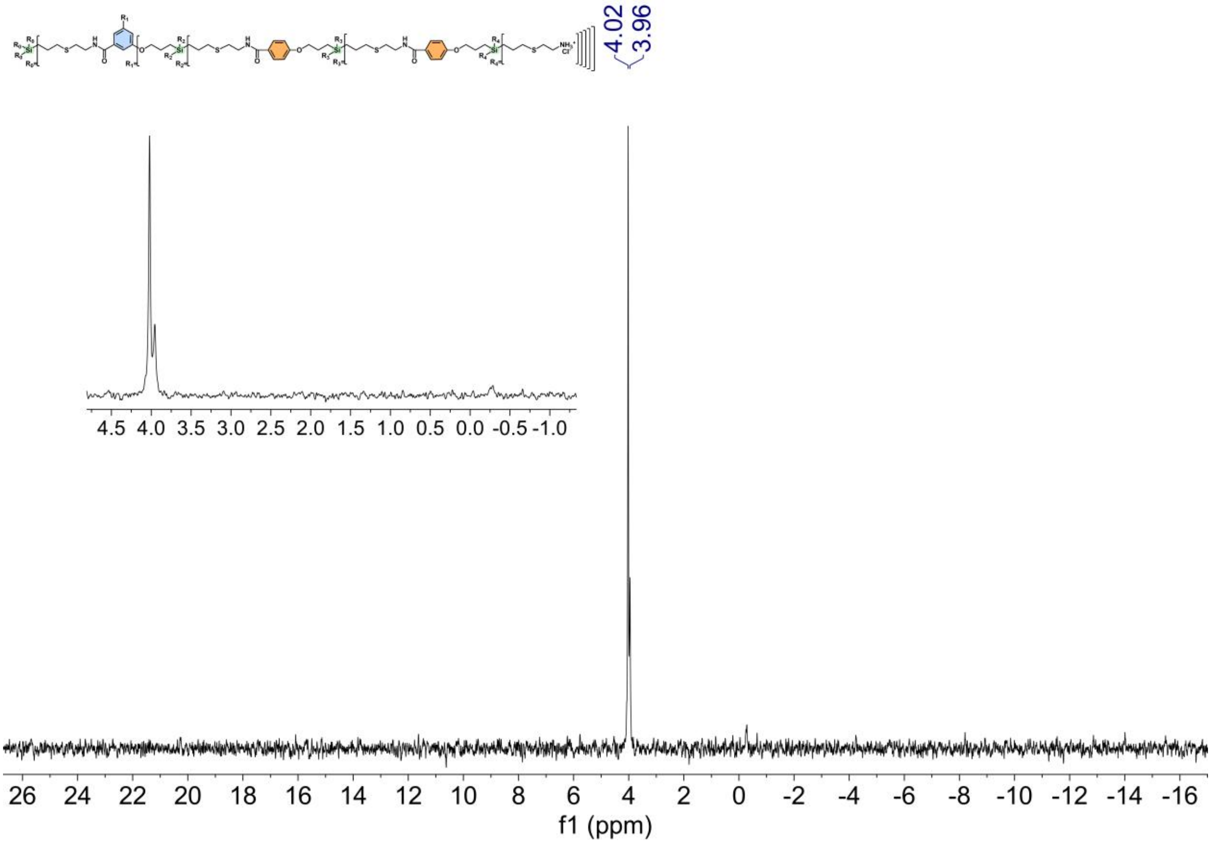

Figure S220: ^{29}Si {^{1}H} NMR (79 MHz, DMSO-d_6) **G$_3$-6-3-3-N**

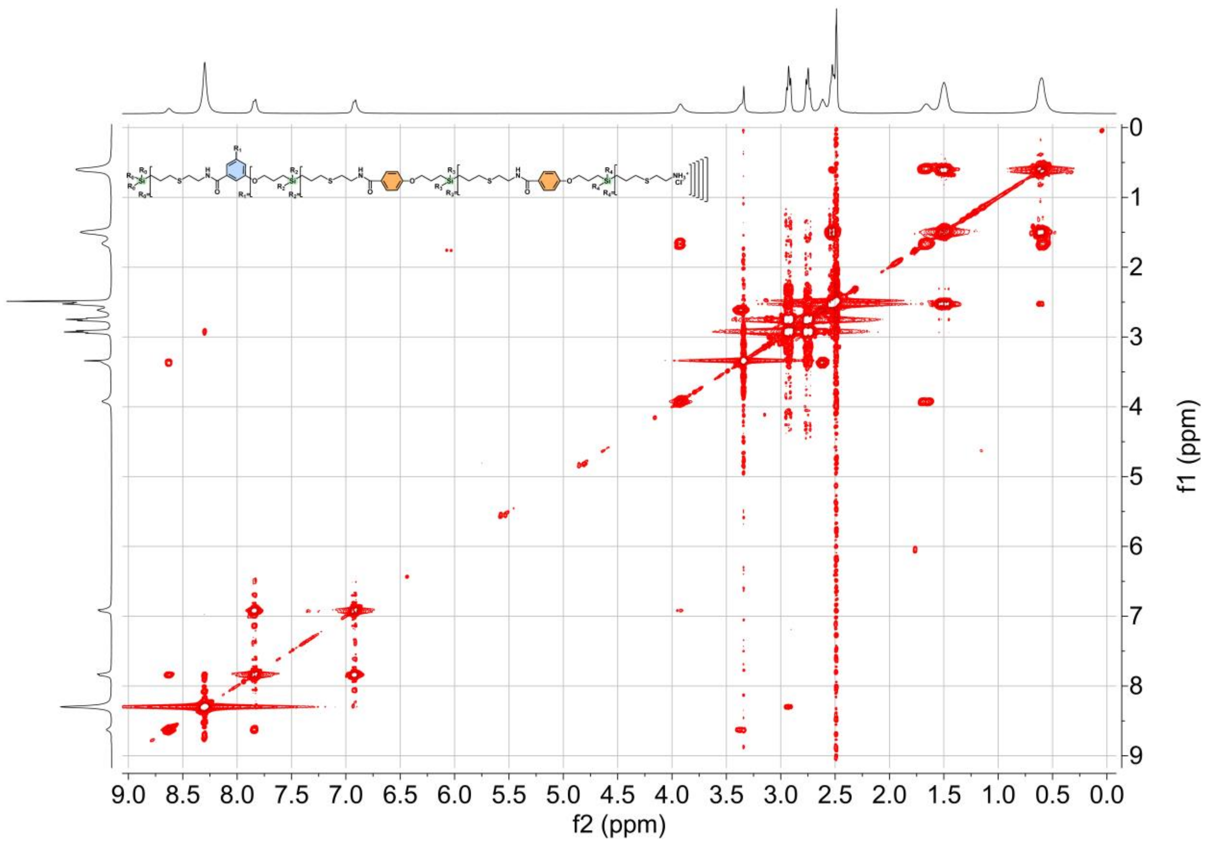

Figure S221: ^{1}H-^{1}H COSY NMR (DMSO-d_6) **G_3-6-3-3-N**

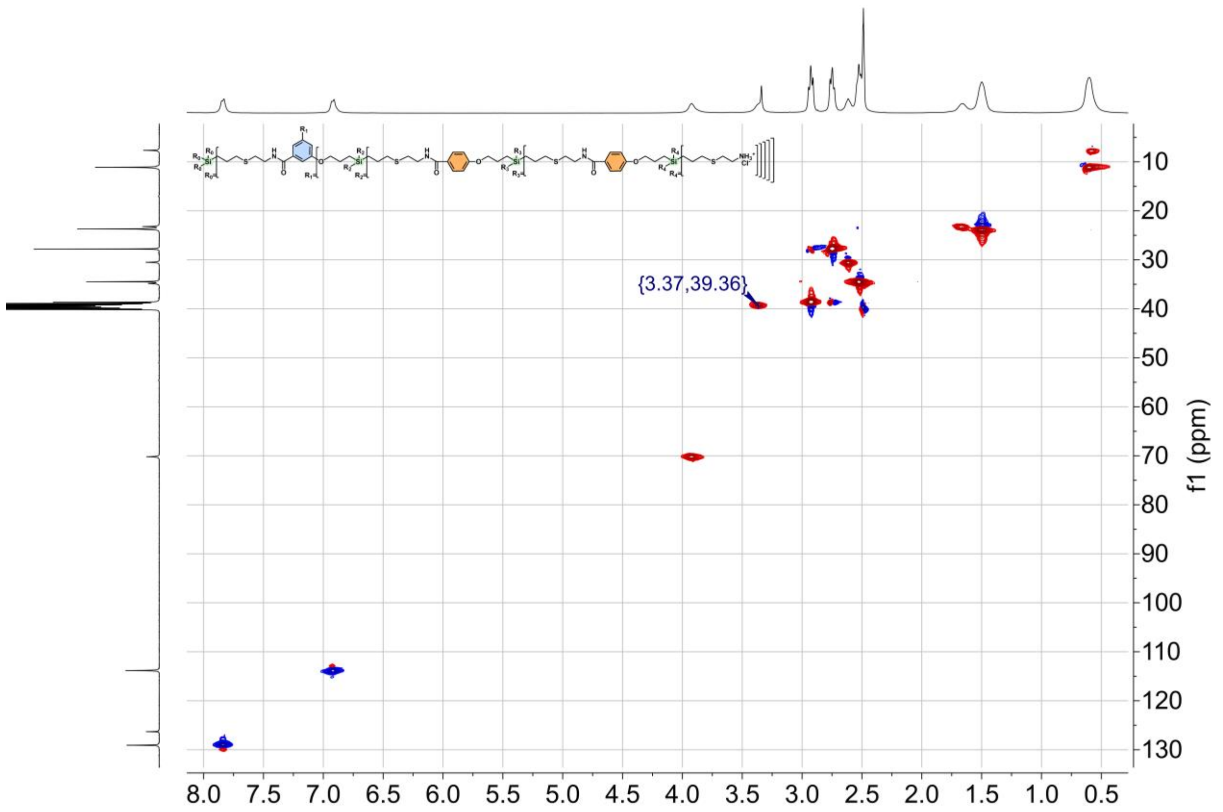

Figure S222: ^{1}H-^{13}C HSQC NMR (DMSO-d_6) **G_3-6-3-3-N**

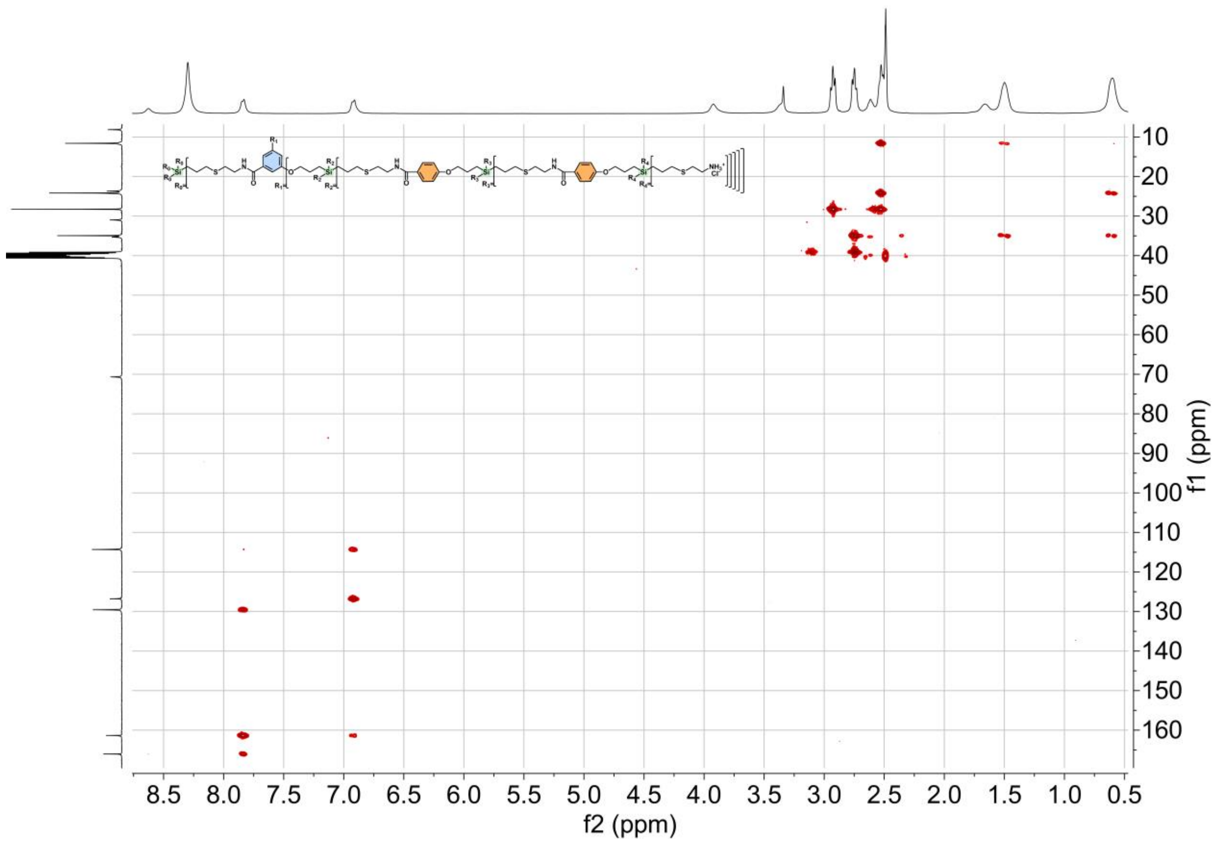

Figure S223: ^{1}H-^{13}C HMBC NMR (DMSO-d_6) **G$_3$-6-3-3-N**

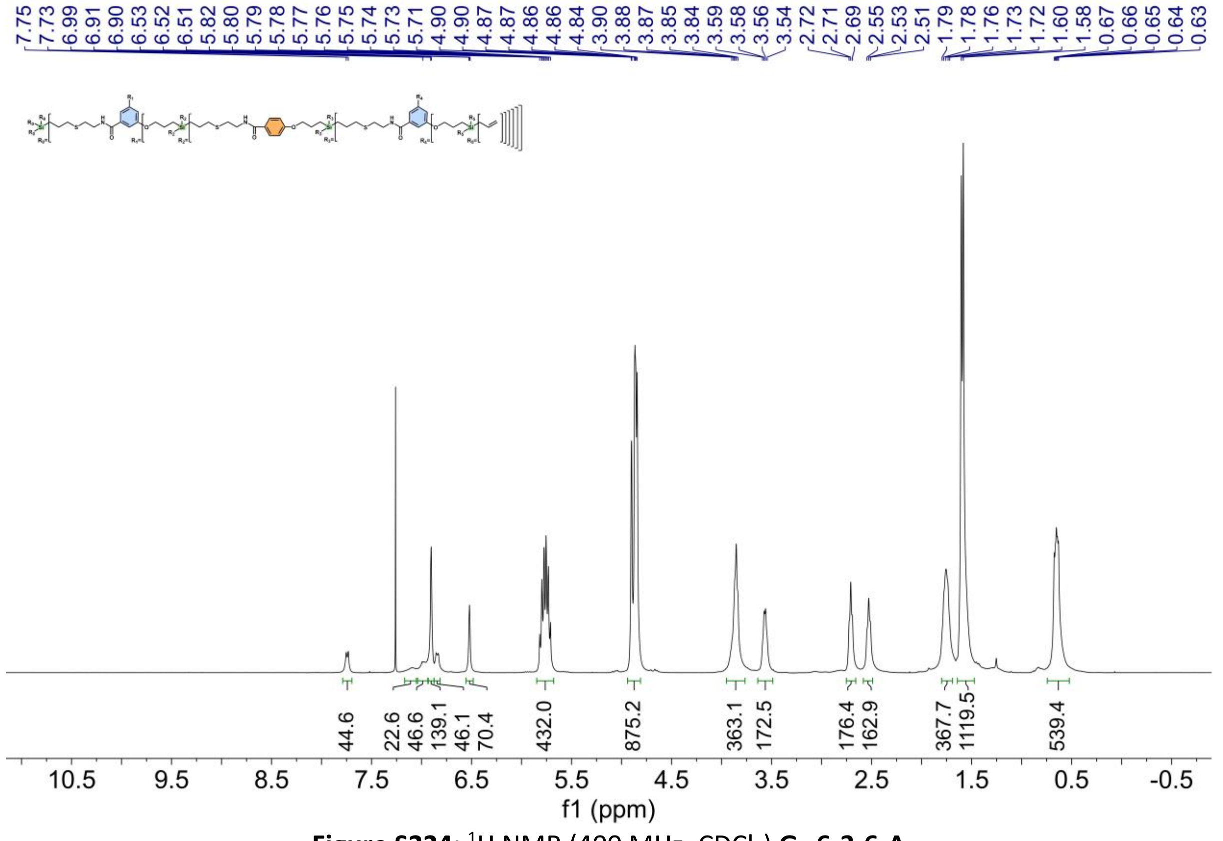

Figure S224: ^{1}H NMR (400 MHz, CDCl$_3$) **G$_3$-6-3-6-A**

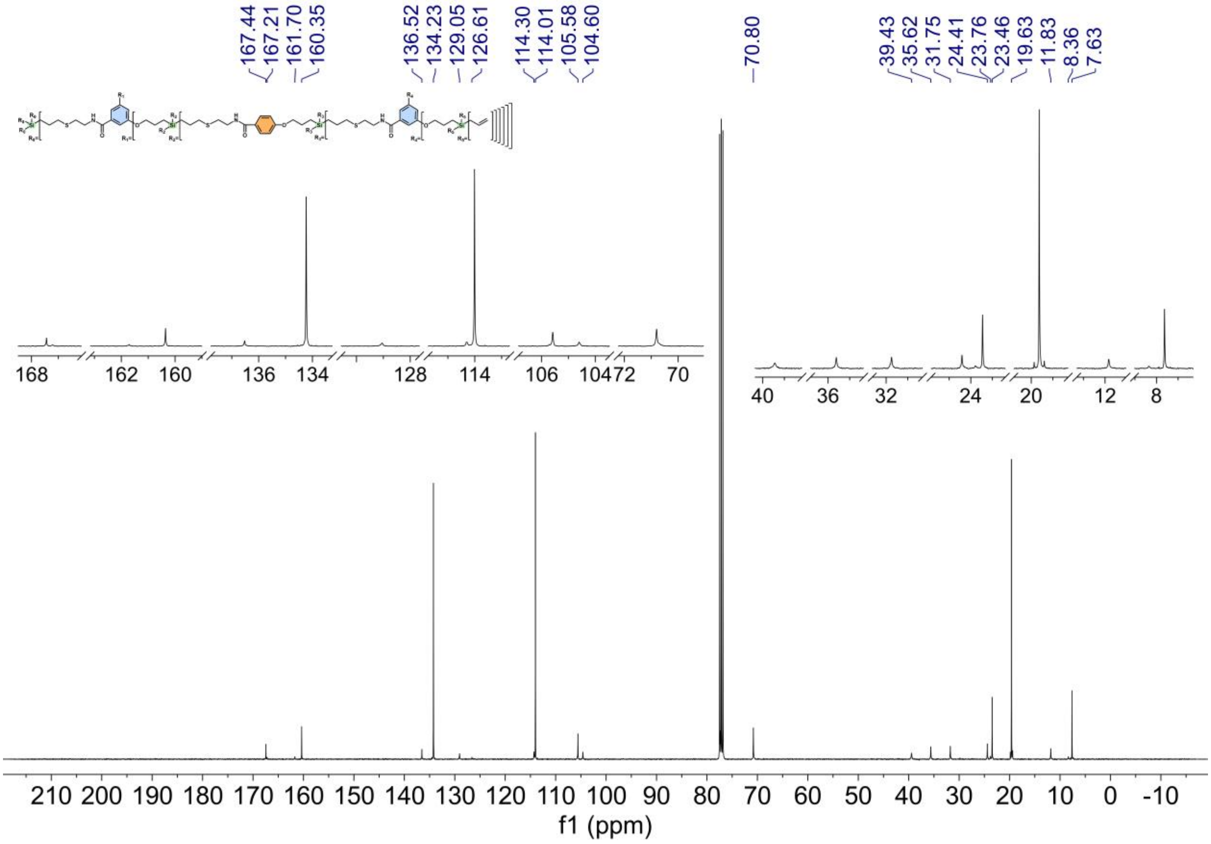

Figure S225: ^{13}C {^{1}H} NMR (101 MHz, $CDCl_3$) **G_3-6-3-6-A**

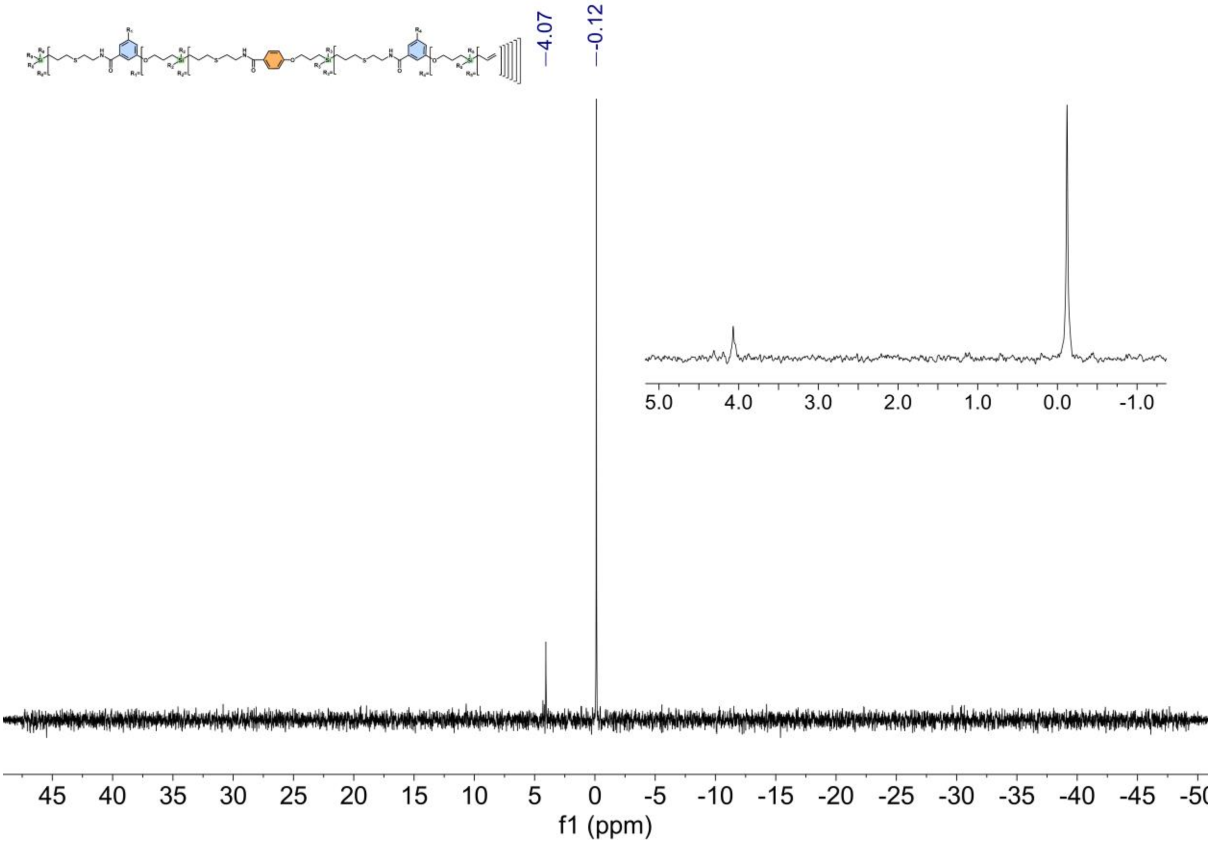

Figure S226: ^{29}Si {^{1}H} NMR (79 MHz, $CDCl_3$) **G_3-6-3-6-A**

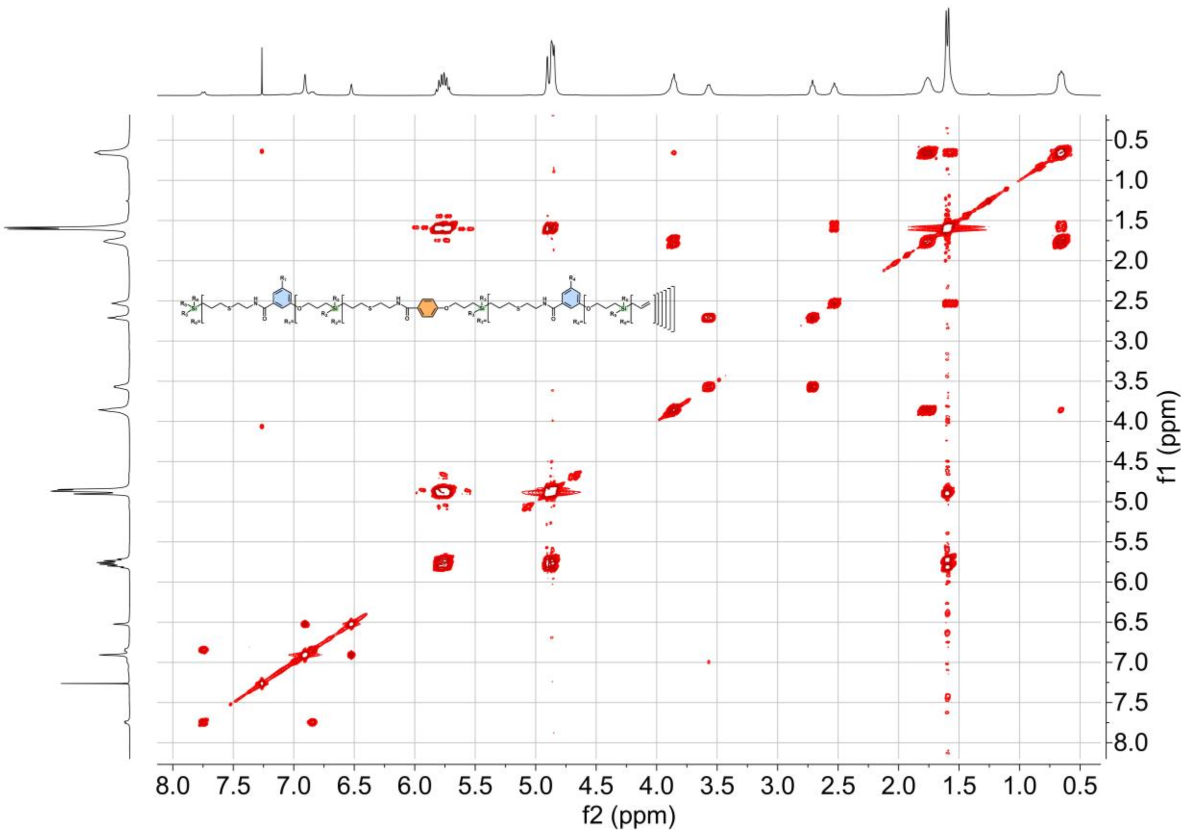

Figure S227: ^{1}H-^{1}H COSY NMR ($CDCl_3$) **G_3-6-3-6-A**

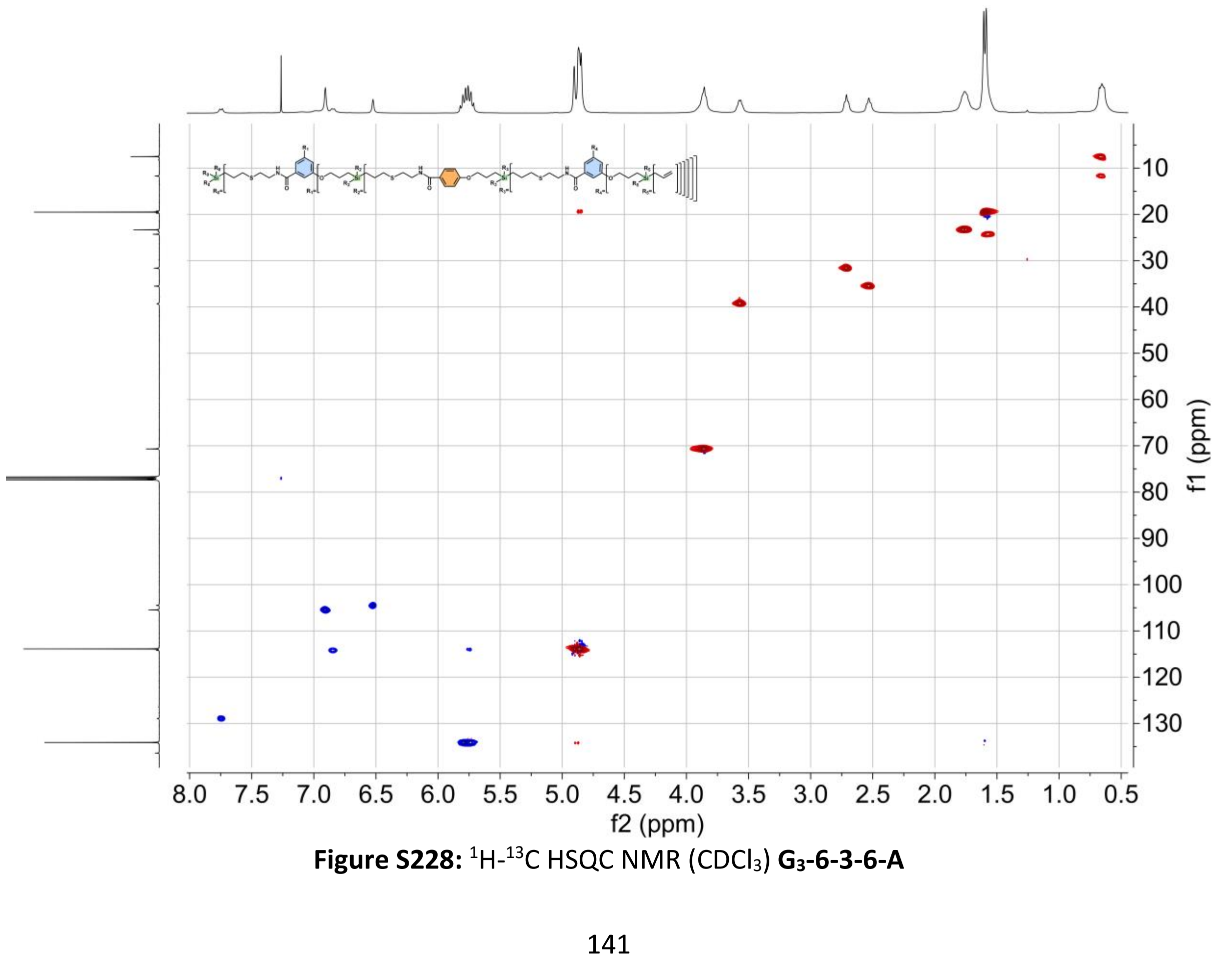

Figure S228: ^{1}H-^{13}C HSQC NMR ($CDCl_3$) **G_3-6-3-6-A**

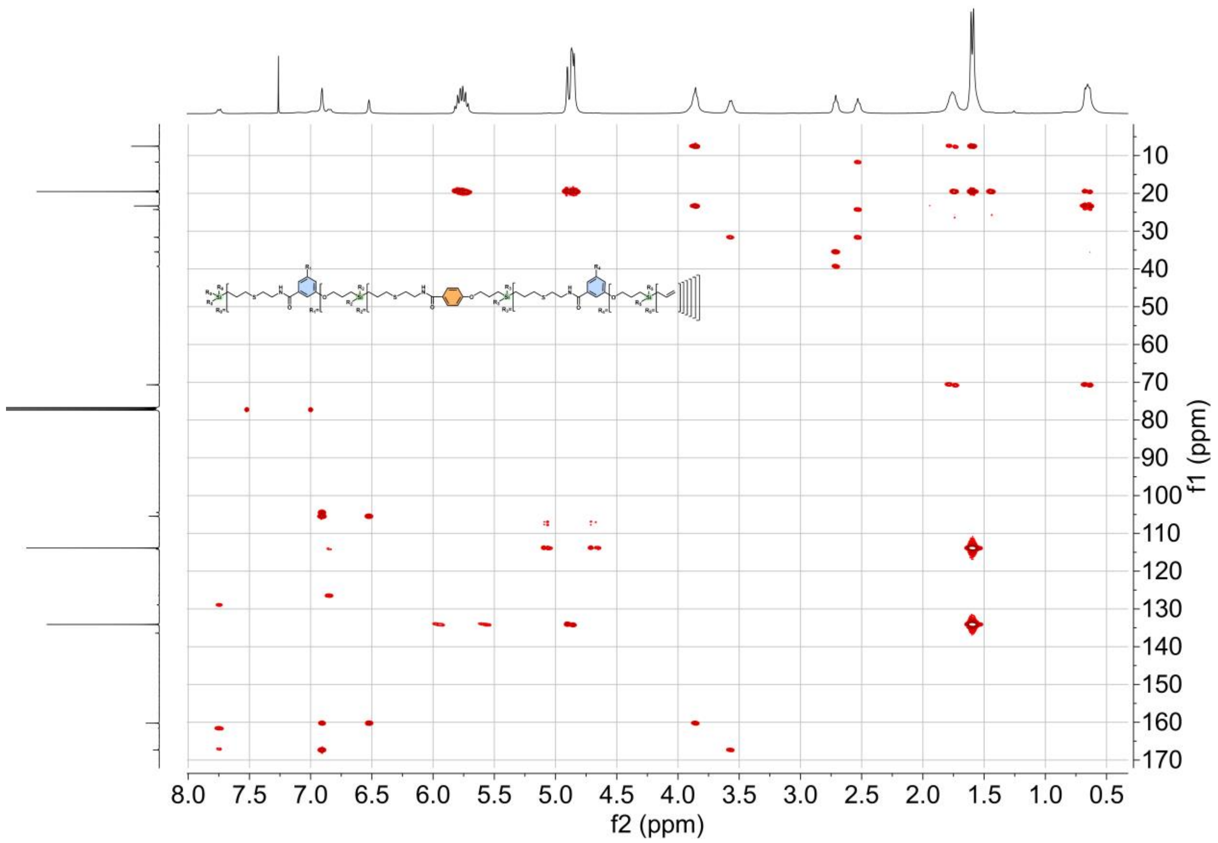

Figure S229: ^{1}H-^{13}C HMBC NMR ($CDCl_3$) **G_3-6-3-6-A**

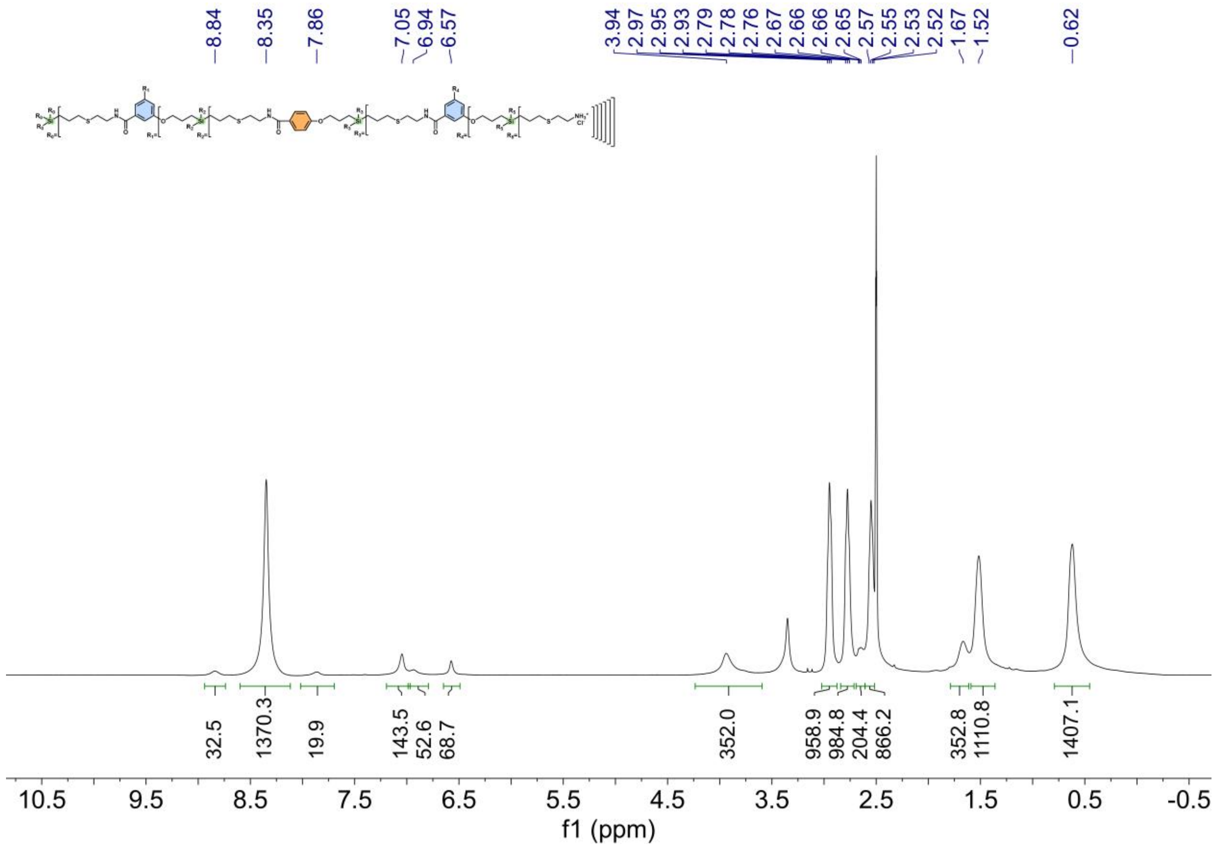

Figure S230: ^{1}H NMR (400 MHz, DMSO-d_6) **G_3-6-3-6-N**

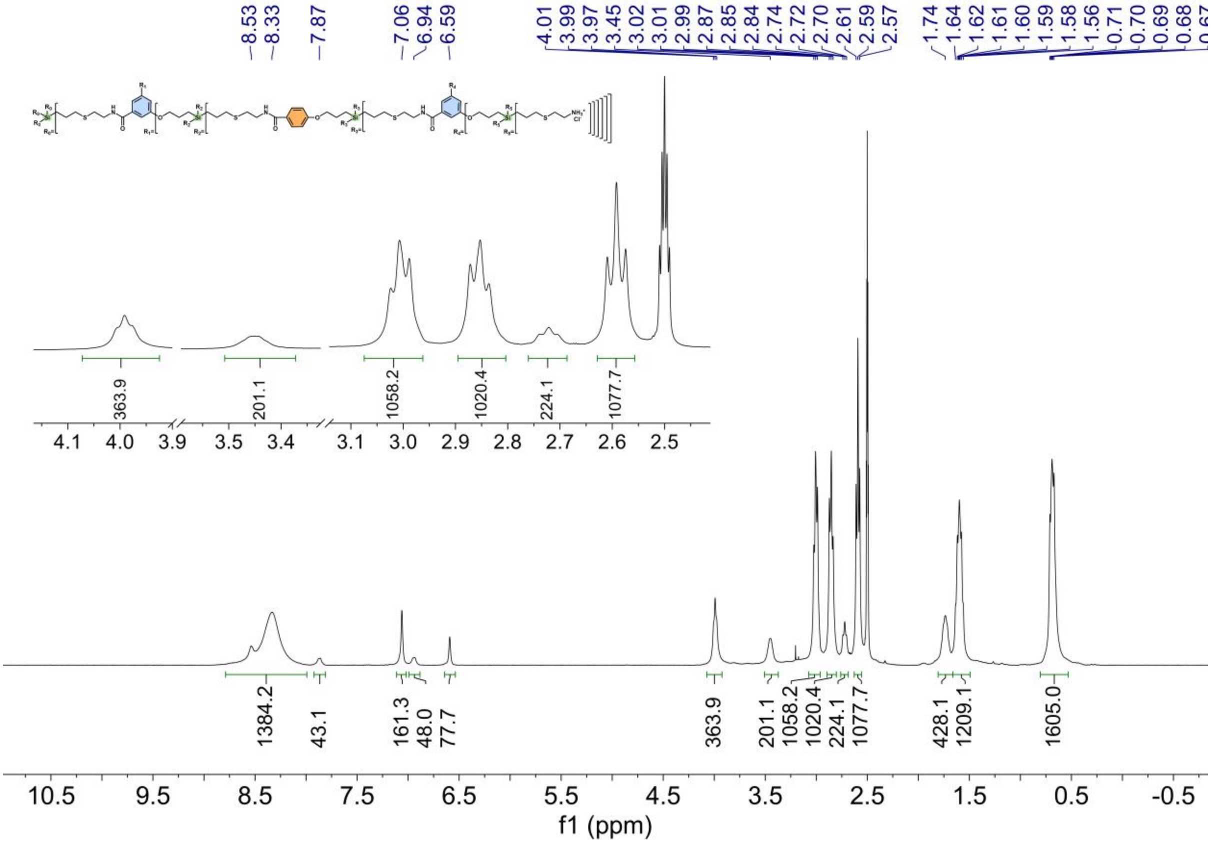

Figure S231: ^{1}H NMR (400 MHz, 100°C, DMSO-d_6) **G_3-6-3-6-N**

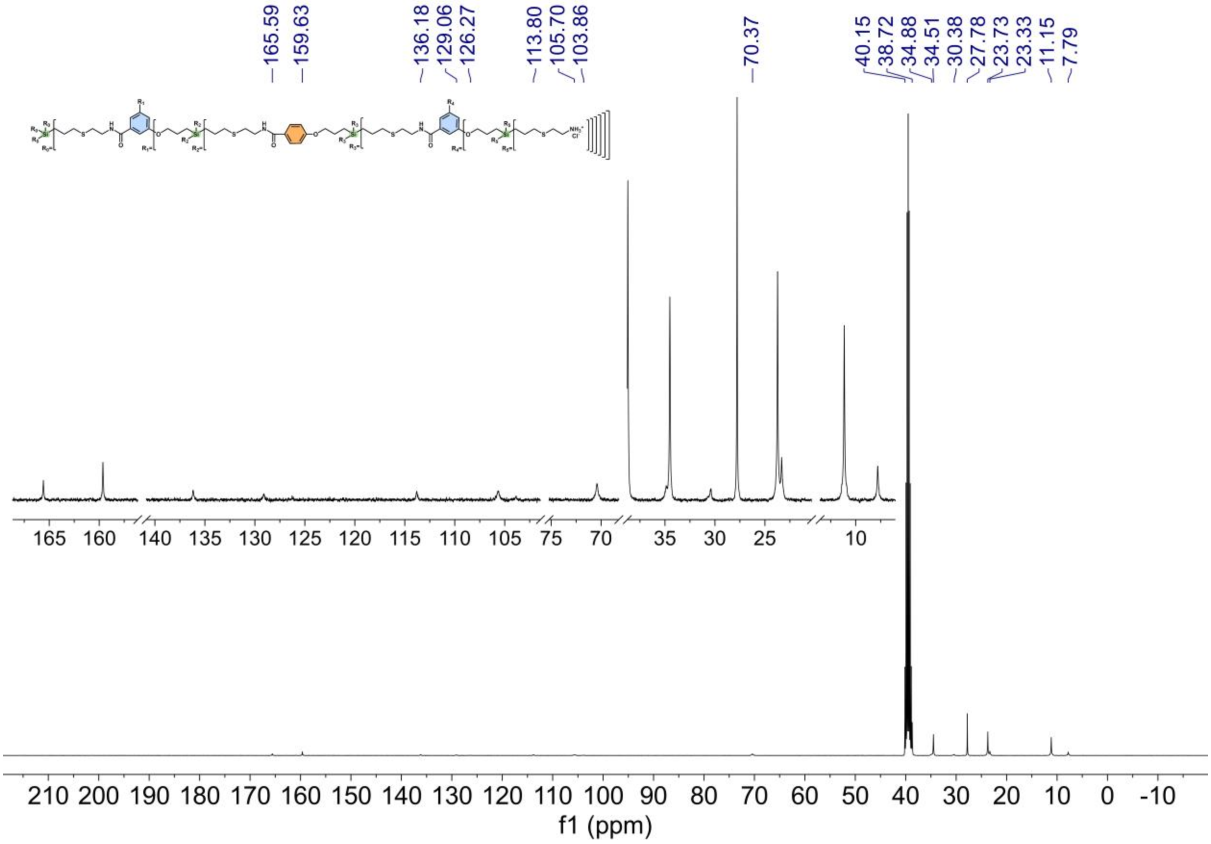

Figure S232: ^{13}C {^{1}H} NMR (101 MHz, DMSO-d_6) **G_3-6-3-6-N**

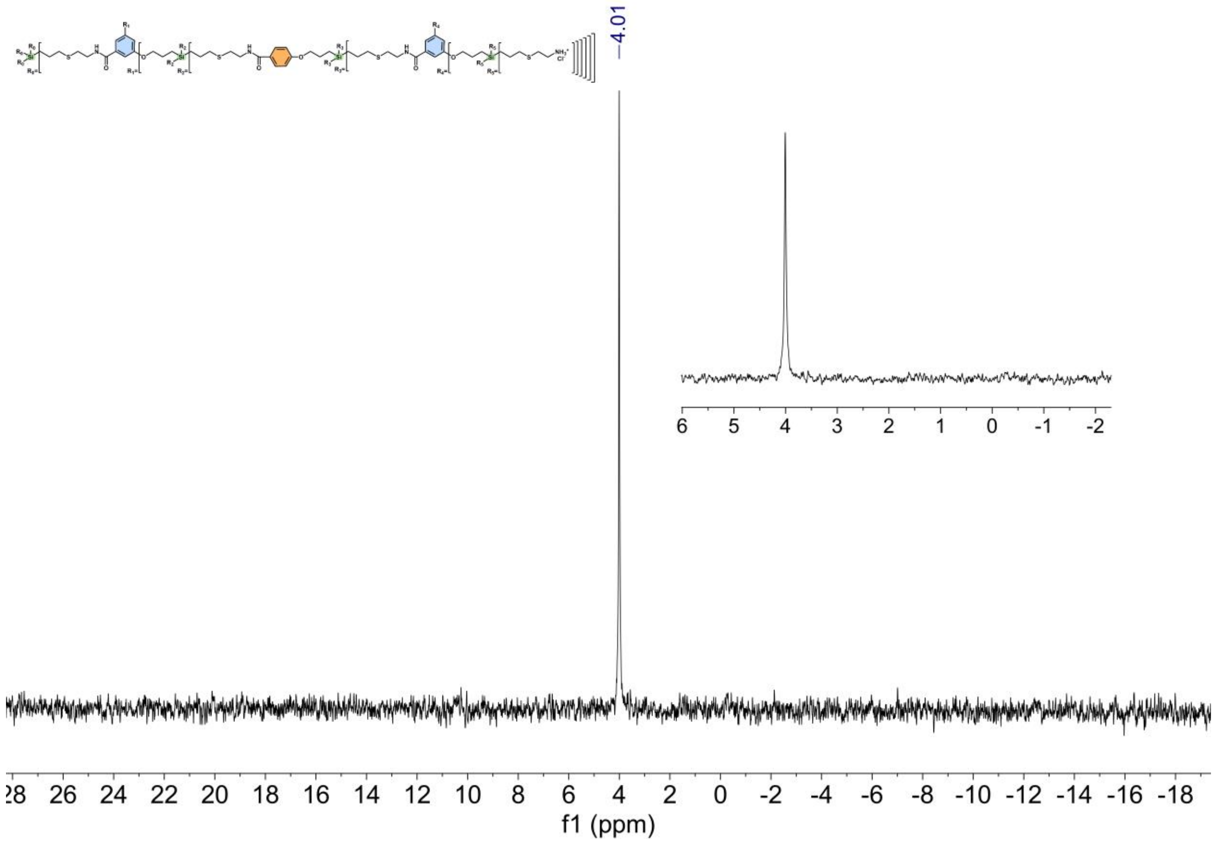

Figure S233: ^{29}Si {^{1}H} NMR (79 MHz, DMSO-d_6) **G_3-6-3-6-N**

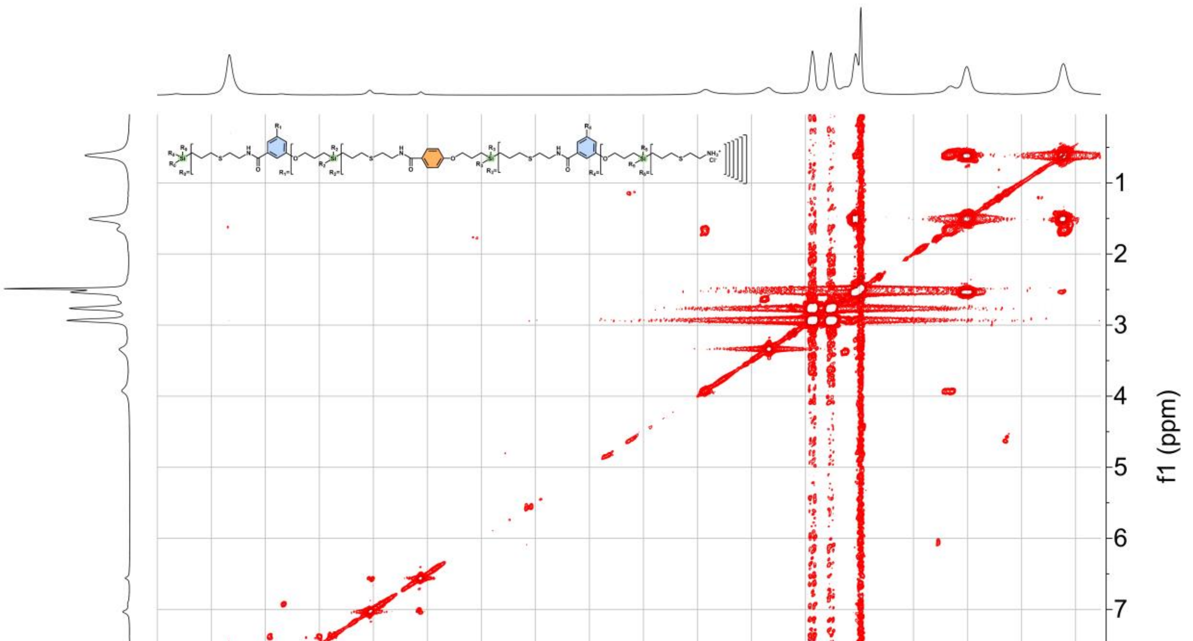

Figure S234: ^{1}H-^{1}H COSY NMR (DMSO-d_6) **G_3-6-3-6-N**

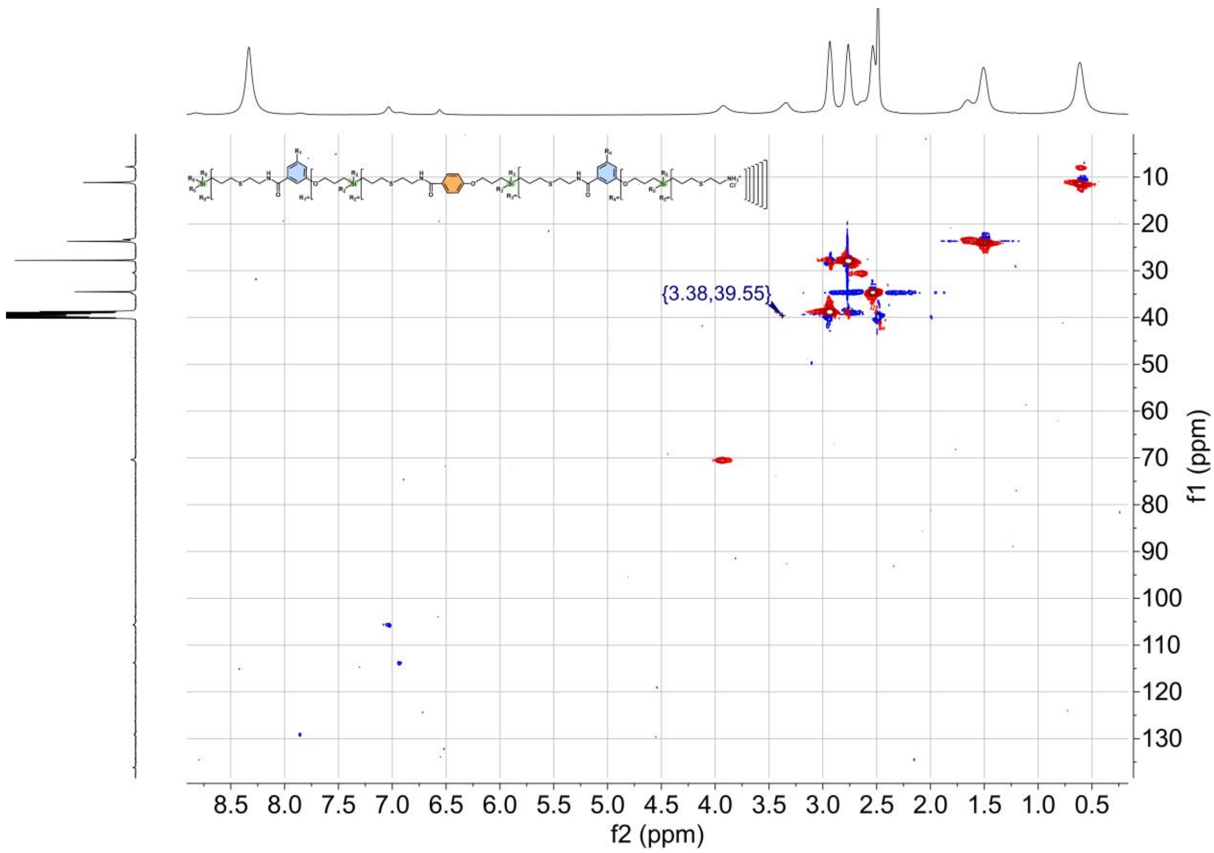

Figure S235: ^{1}H-^{13}C HSQC NMR (DMSO-d_6) **G_3-6-3-6-N**

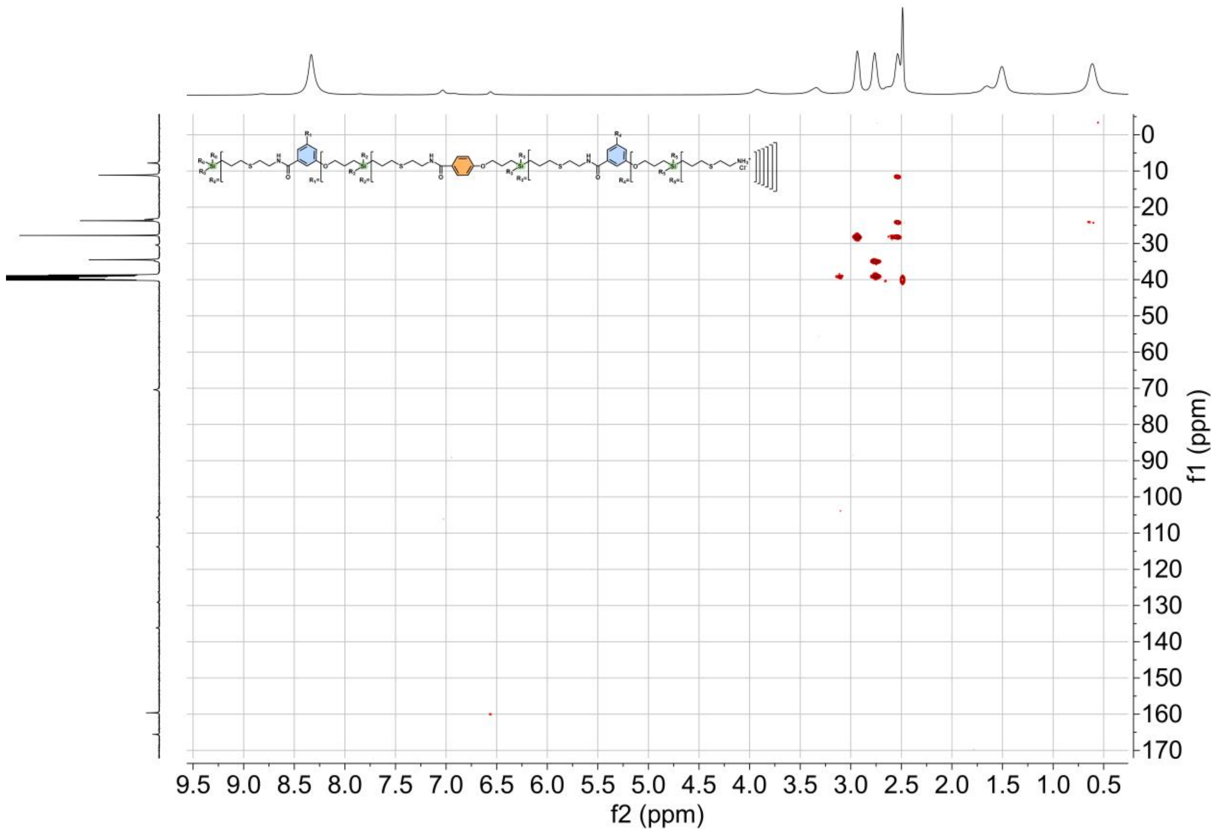

Figure S236: ^{1}H-^{13}C HMBC NMR (DMSO-d_6) **G_3-6-3-6-N**

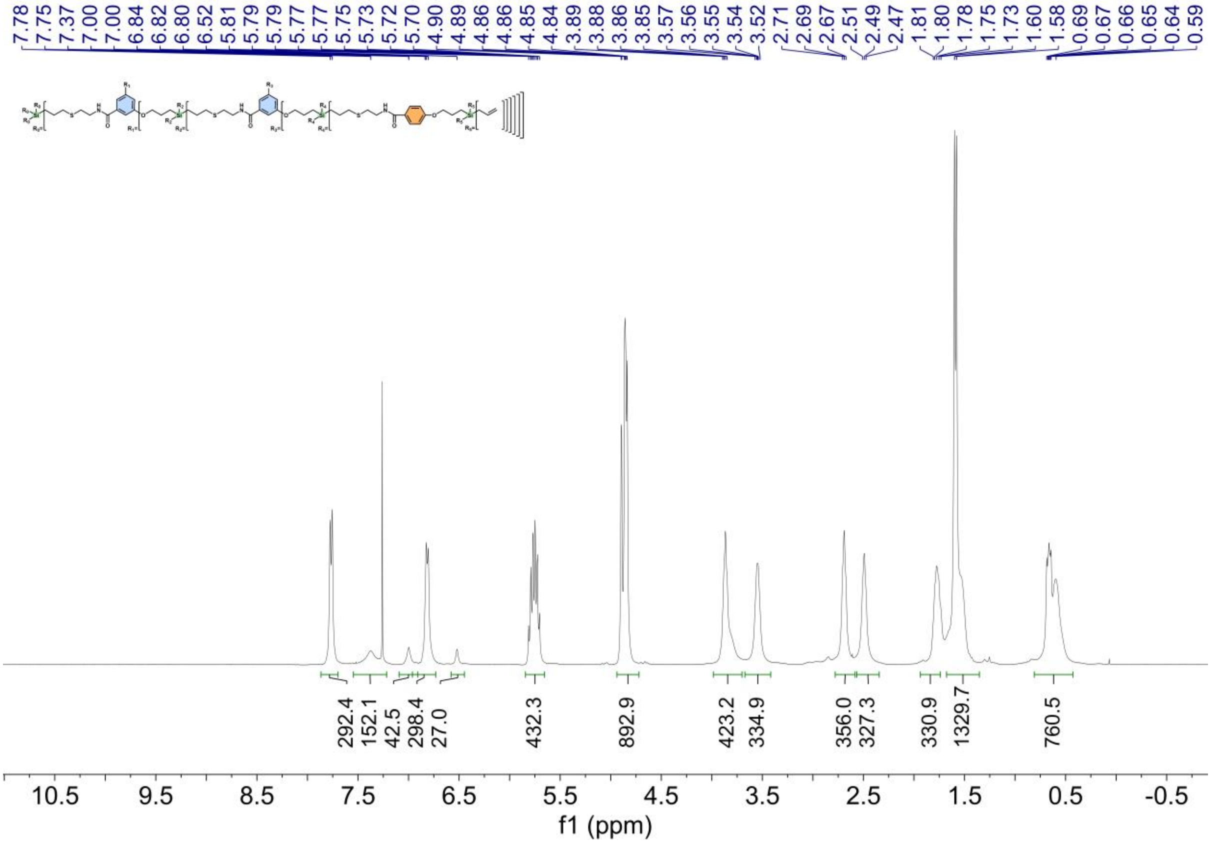

Figure S237: ^{1}H NMR (400 MHz, $CDCl_3$) **G_3-6-6-3-A**

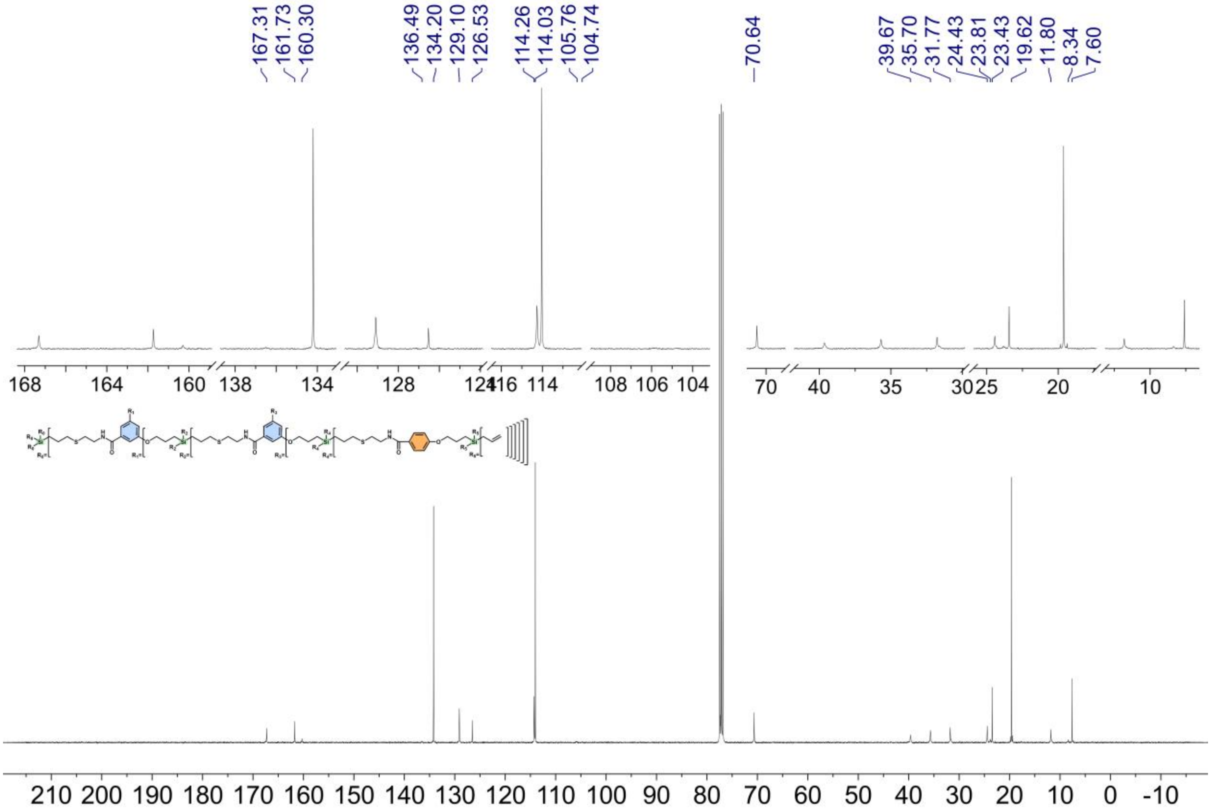

Figure S238: $^{13}C\ \{^{1}H\}$ NMR (101 MHz, $CDCl_3$) **G_3-6-6-3-A**

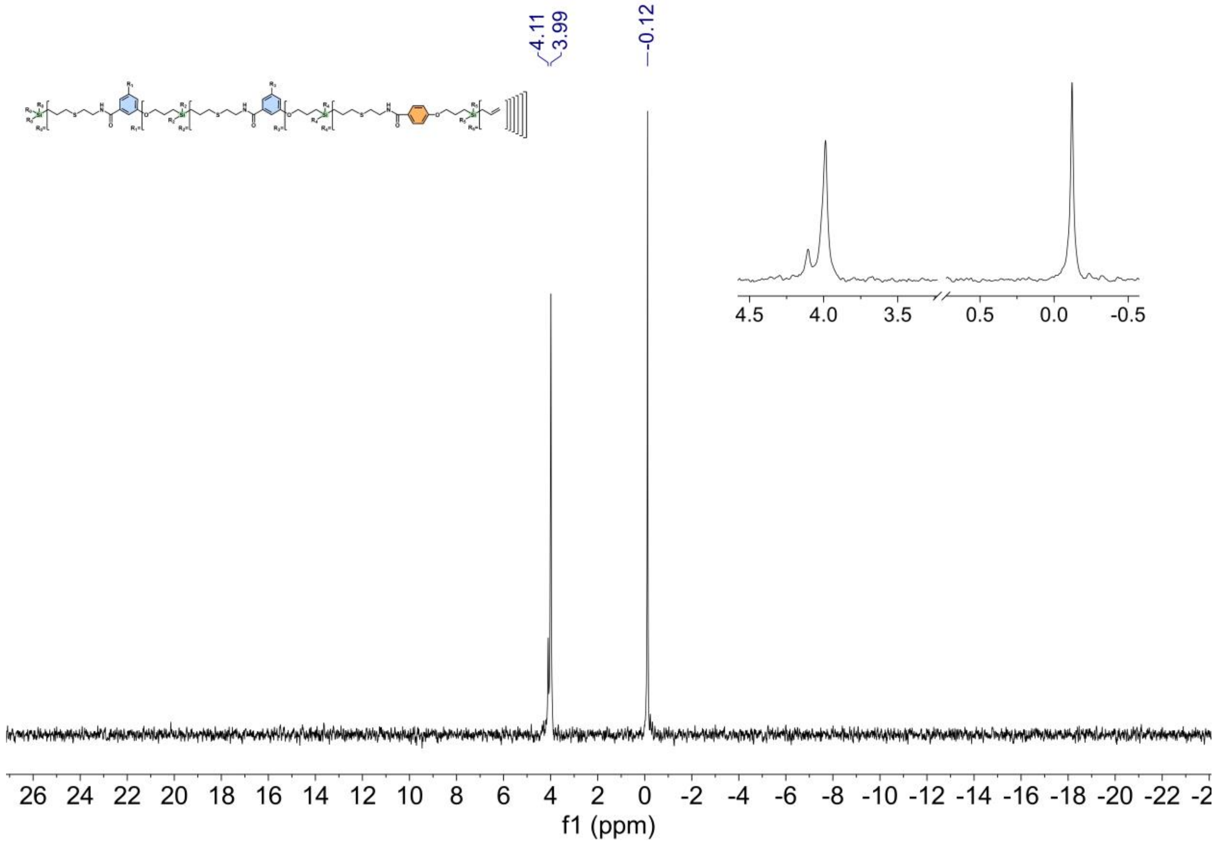

Figure S239: ^{29}Si {^{1}H} NMR (79 MHz, $CDCl_3$) **G_3-6-6-3-A**

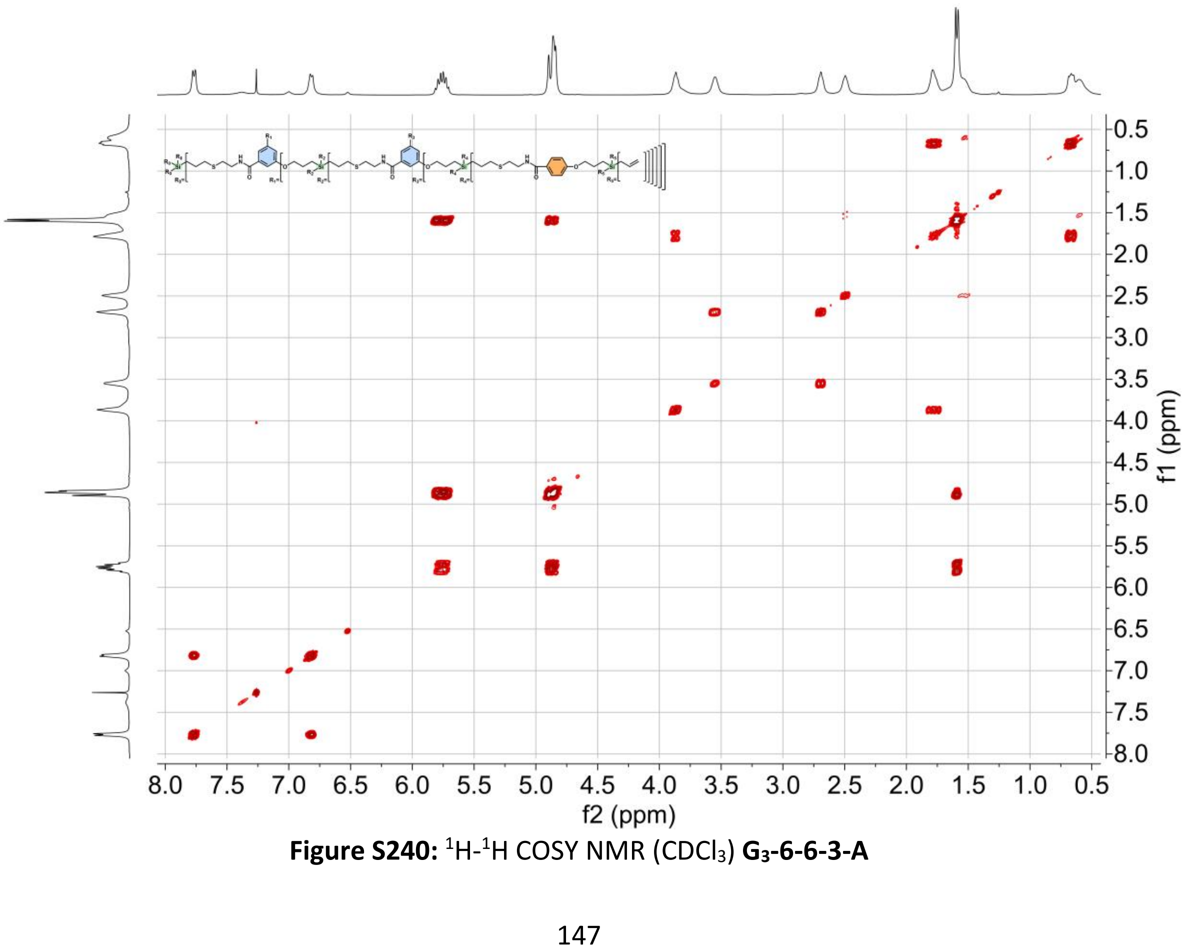

Figure S240: ^{1}H-^{1}H COSY NMR ($CDCl_3$) **G_3-6-6-3-A**

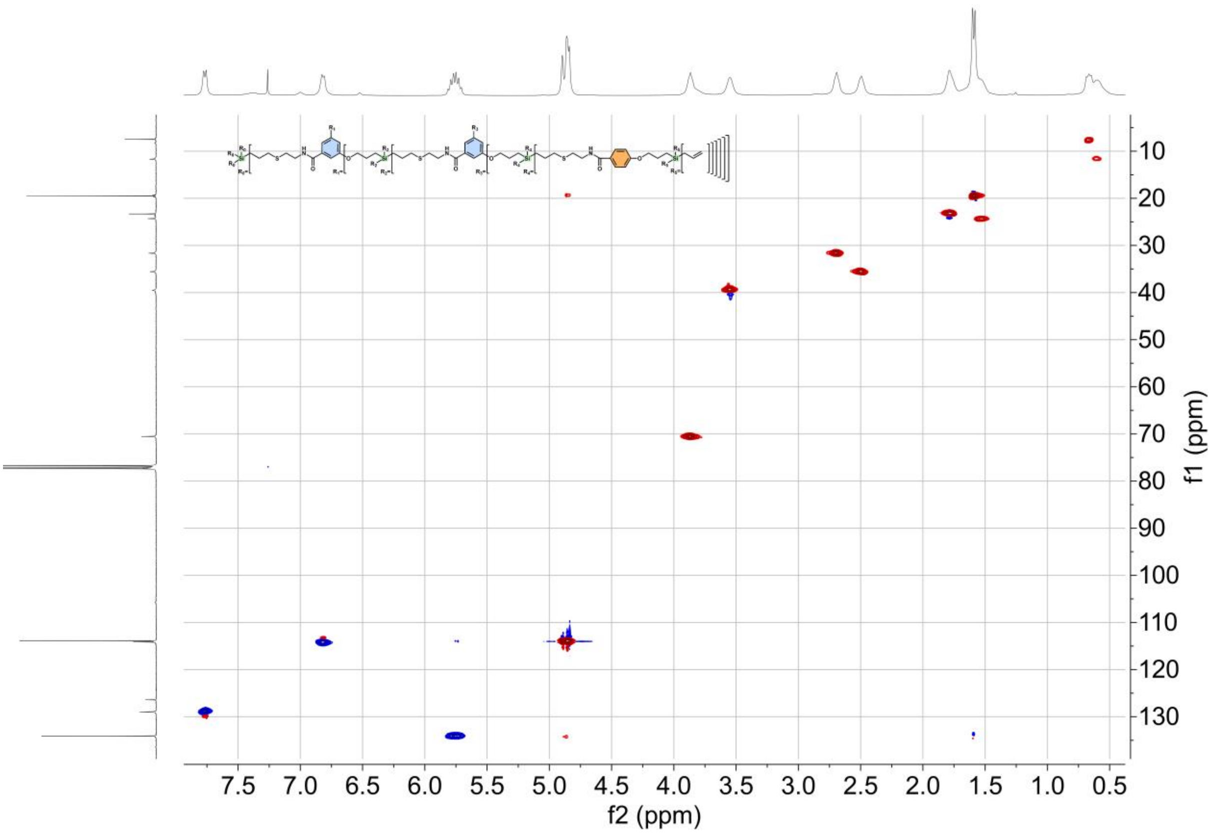

Figure S241: ^{1}H-^{13}C HSQC NMR ($CDCl_3$) **G_3-6-6-3-A**

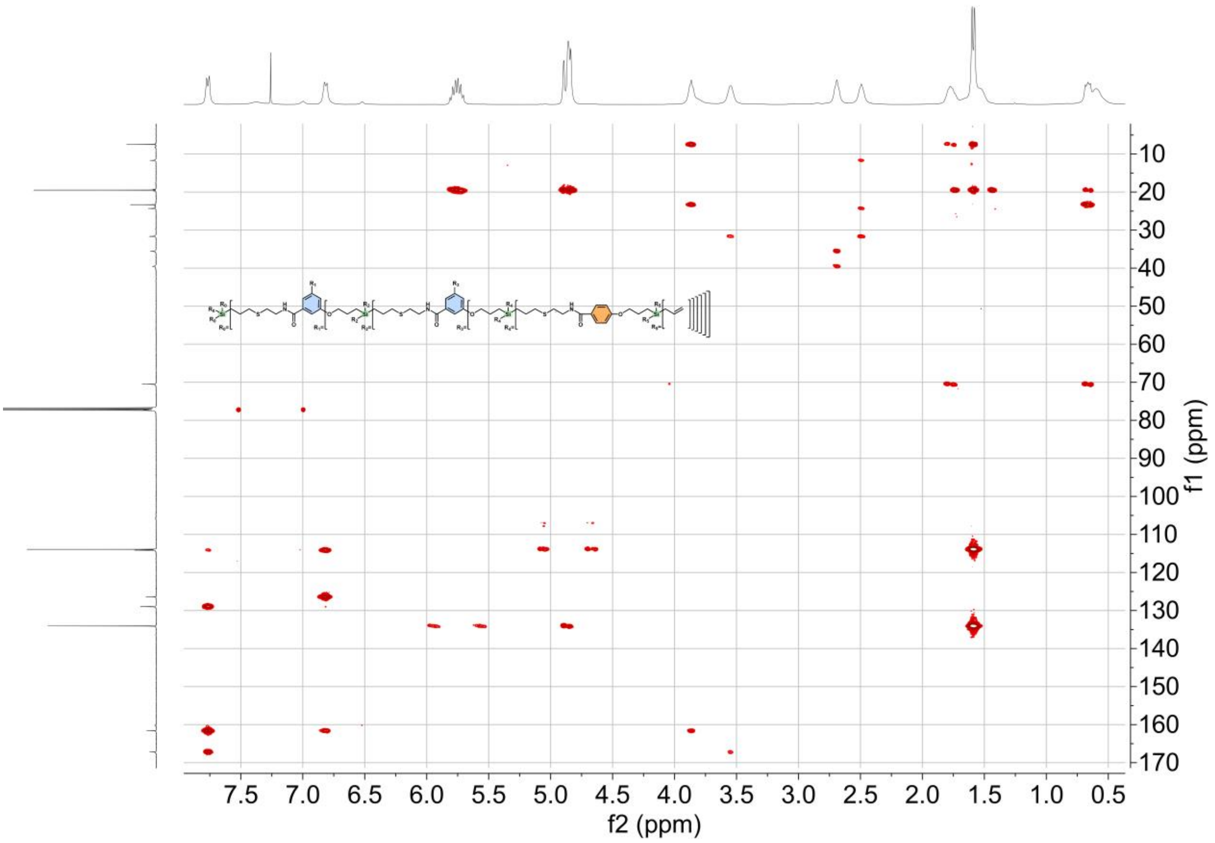

Figure S242: ^{1}H-^{13}C HMBC NMR ($CDCl_3$) **G_3-6-6-3-A**

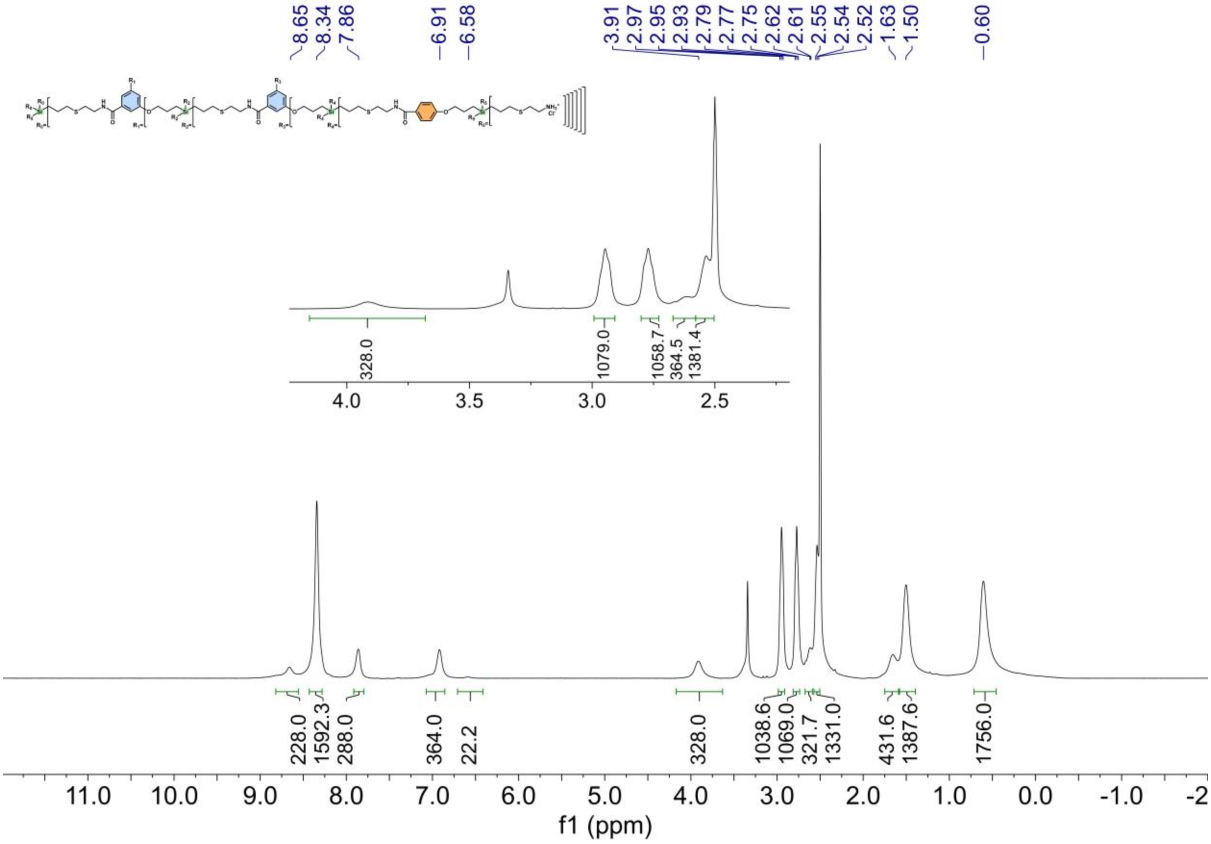

Figure S243: ^{1}H NMR (400 MHz, DMSO-d_6) **G$_3$-6-6-3-N**

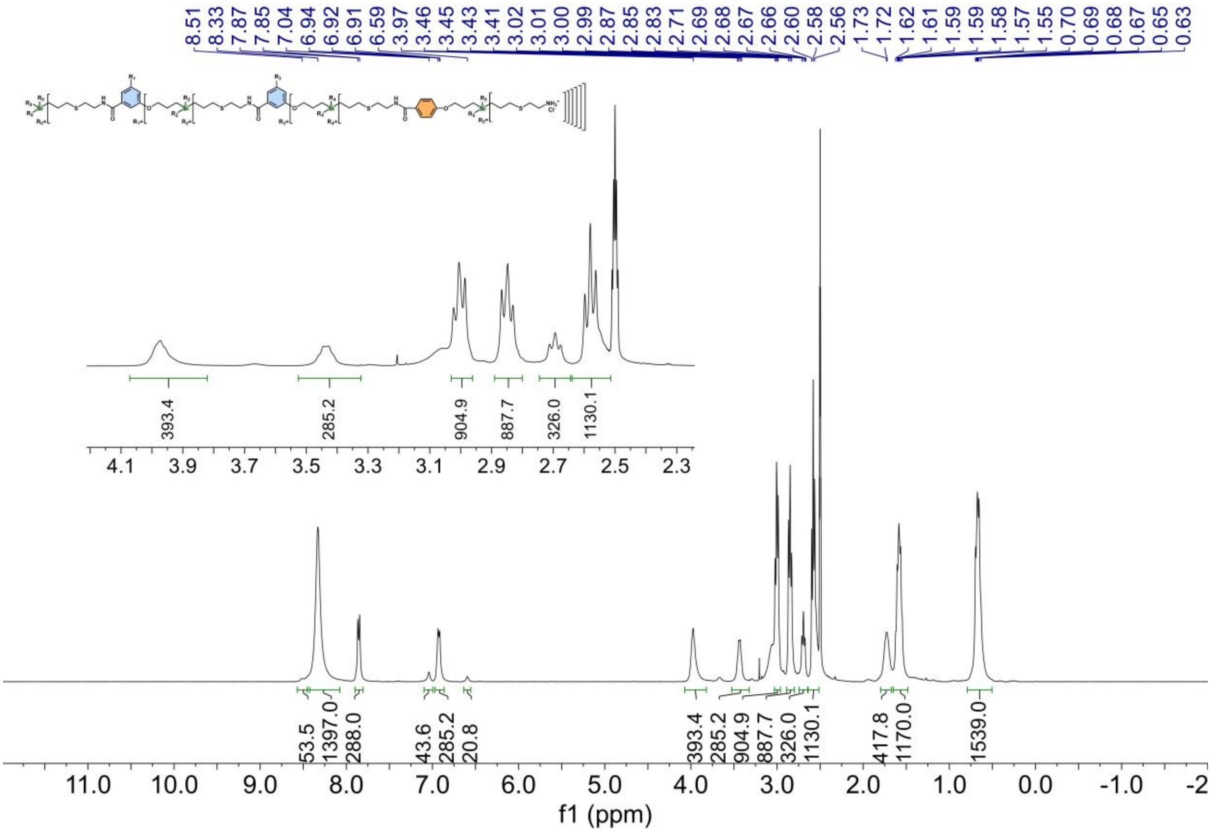

Figure S244: ^{1}H NMR (400 MHz, 100°C, DMSO-d_6) **G$_3$-6-6-3-N**

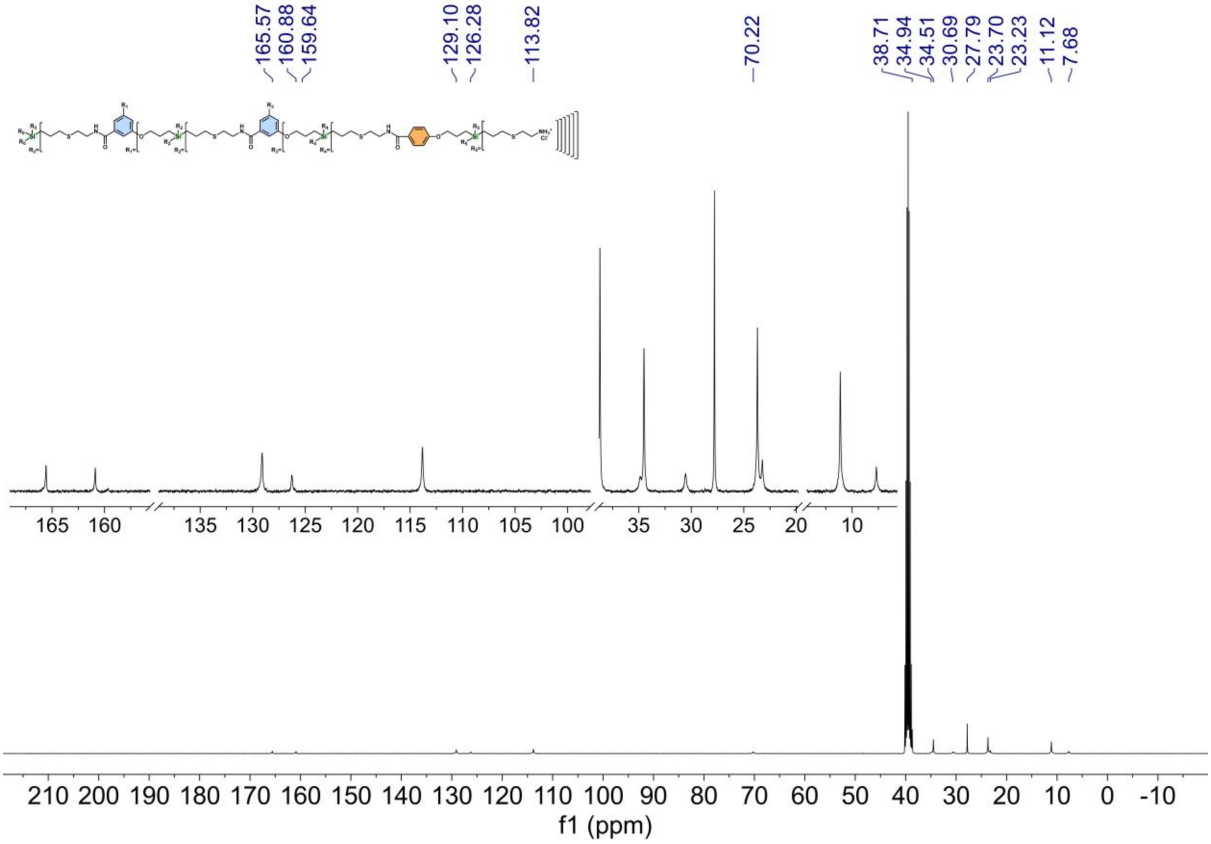

Figure S245: $^{13}C\ \{^1H\}$ NMR (101 MHz, DMSO-d_6) **G_3-6-6-3-N**

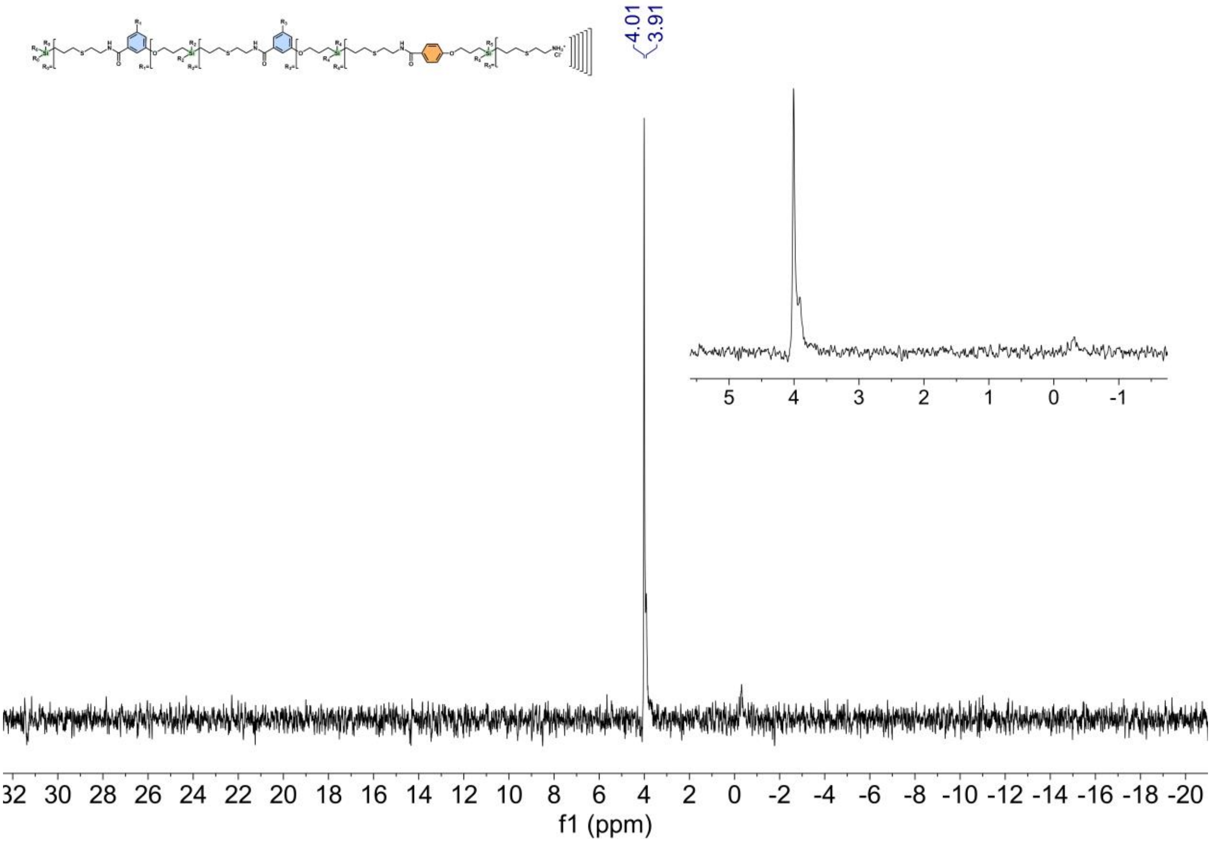

Figure S246: $^{29}Si\ \{^1H\}$ NMR (79 MHz, DMSO-d_6) **G_3-6-6-3-N**

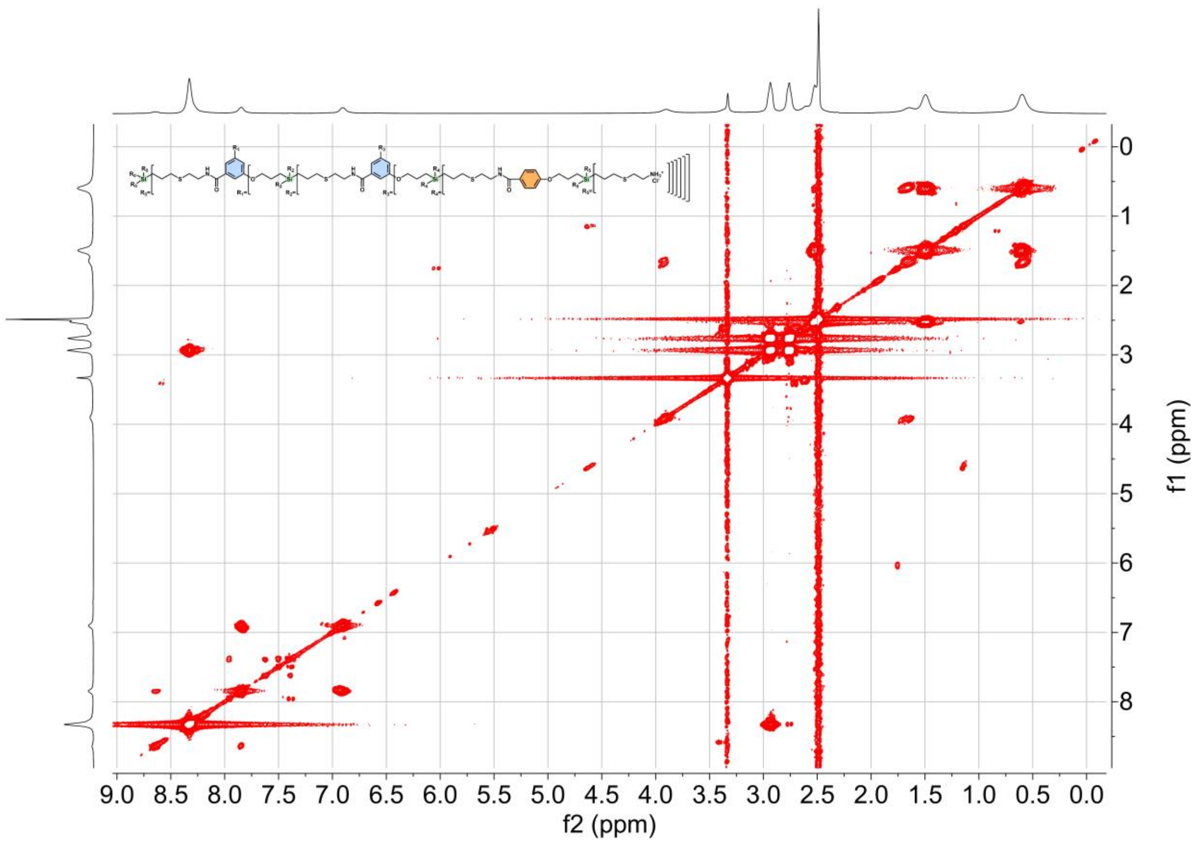

Figure S247: ^{1}H-^{1}H COSY NMR (DMSO-d_6) **G_3-6-6-3-N**

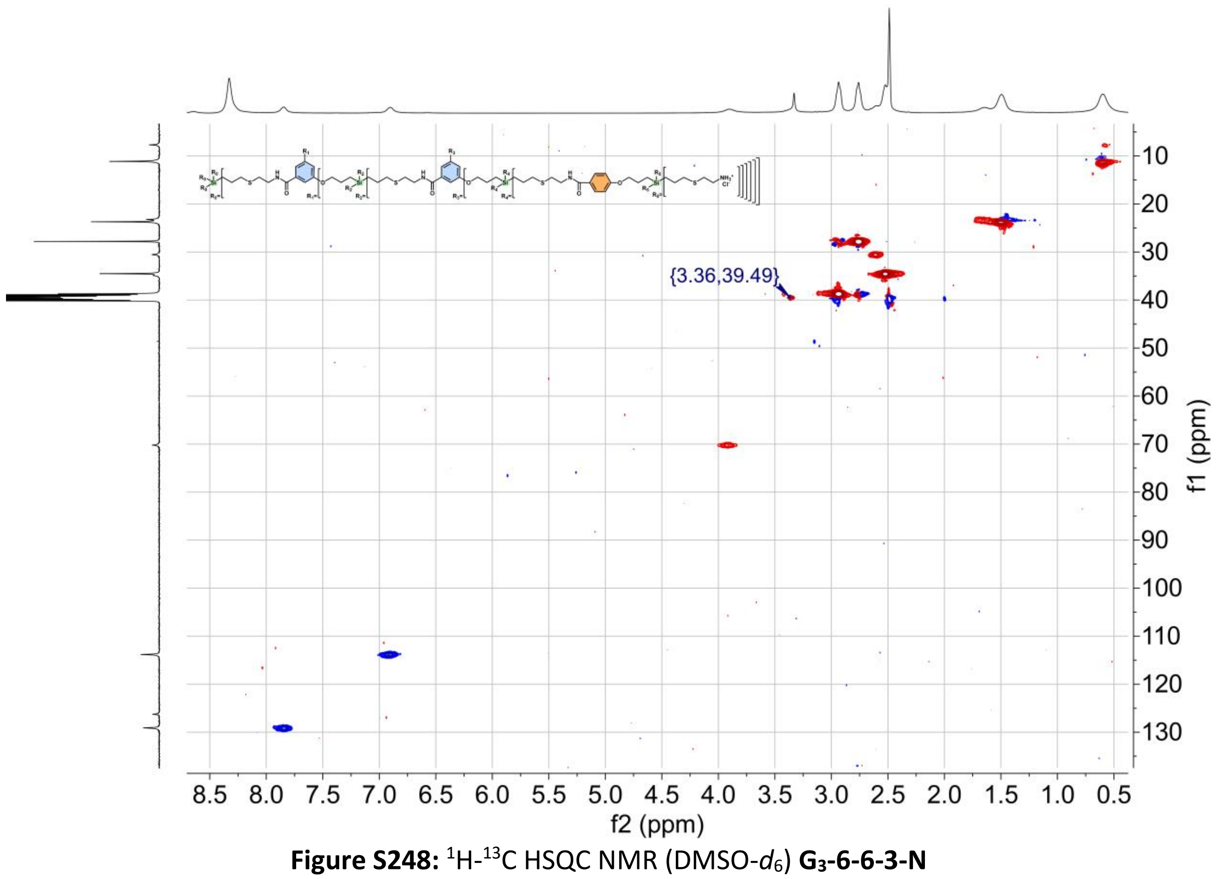

Figure S248: ^{1}H-^{13}C HSQC NMR (DMSO-d_6) **G_3-6-6-3-N**

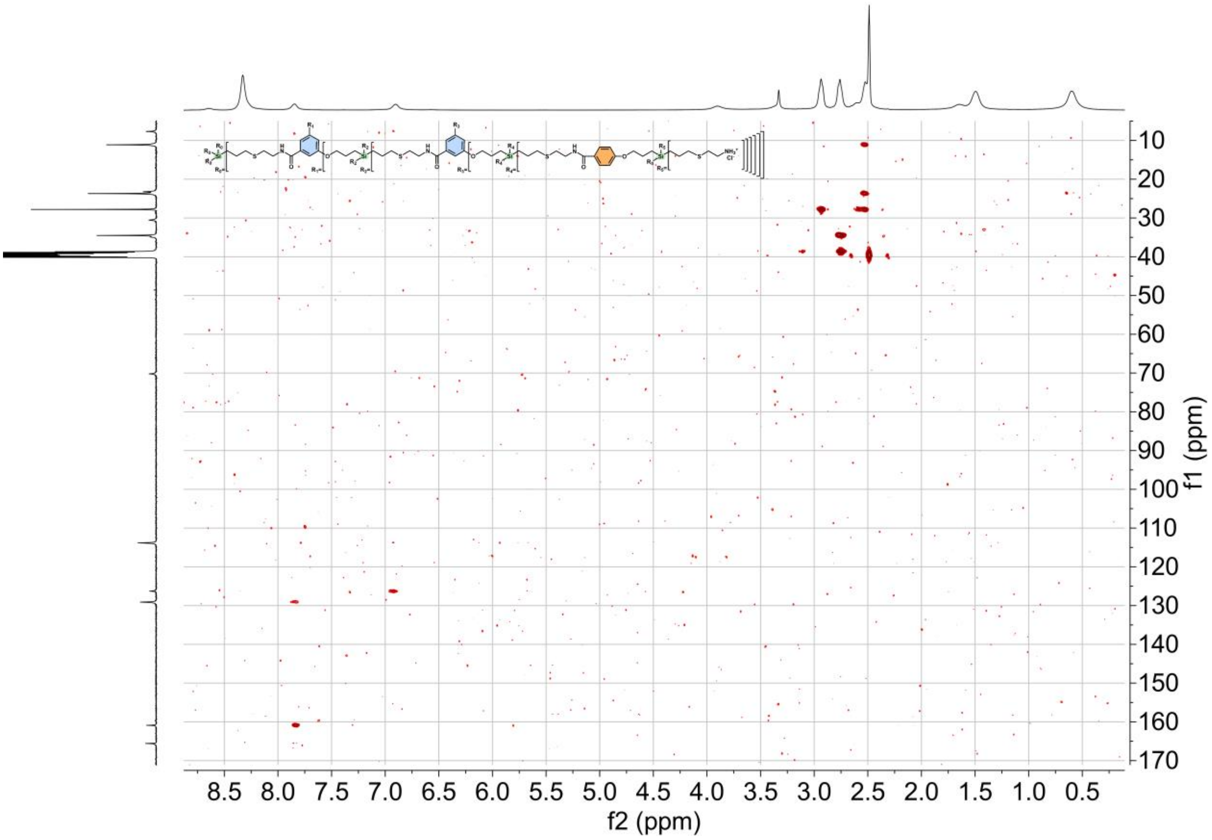

Figure S249: ^{1}H-^{13}C HMBC NMR (DMSO-d_6) **G_3-6-6-3-N**

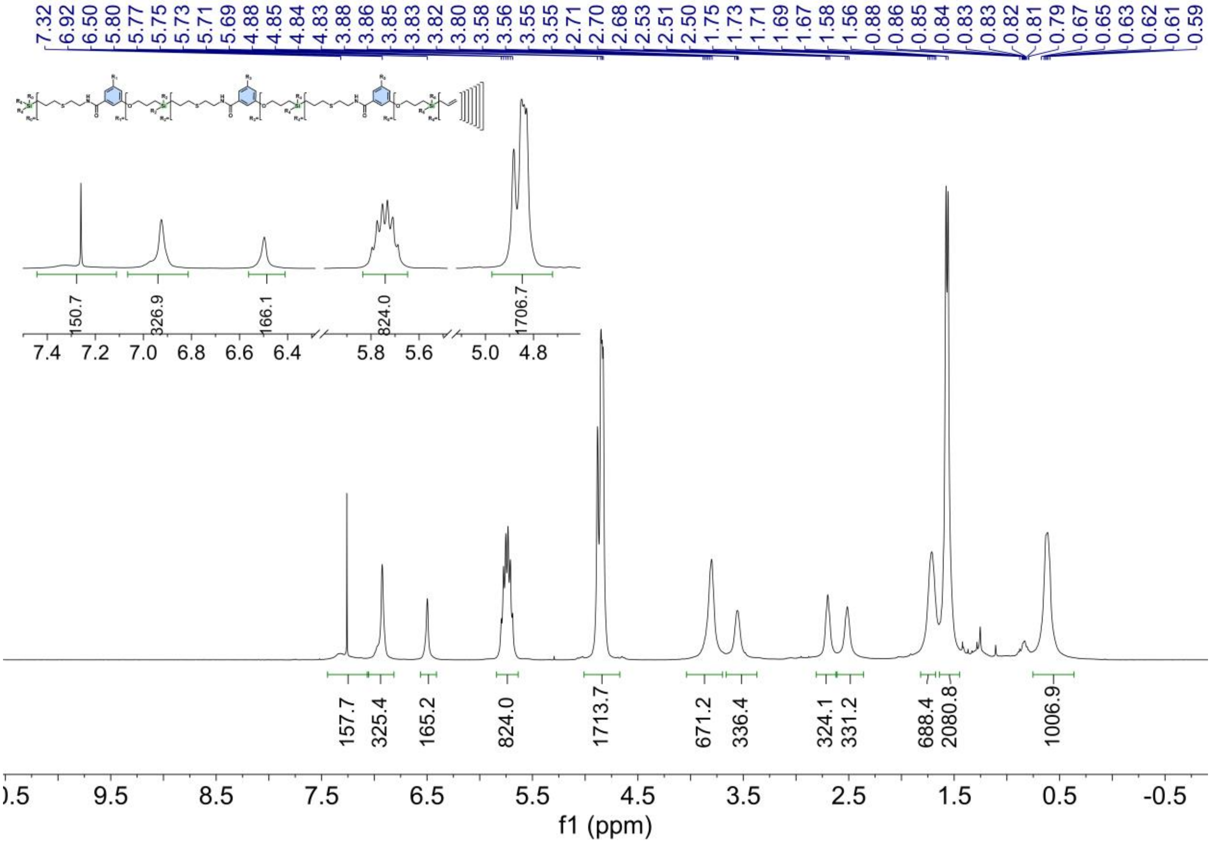

Figure S250: ^{1}H NMR (400 MHz, $CDCl_3$) **G_3-6-6-6-A**

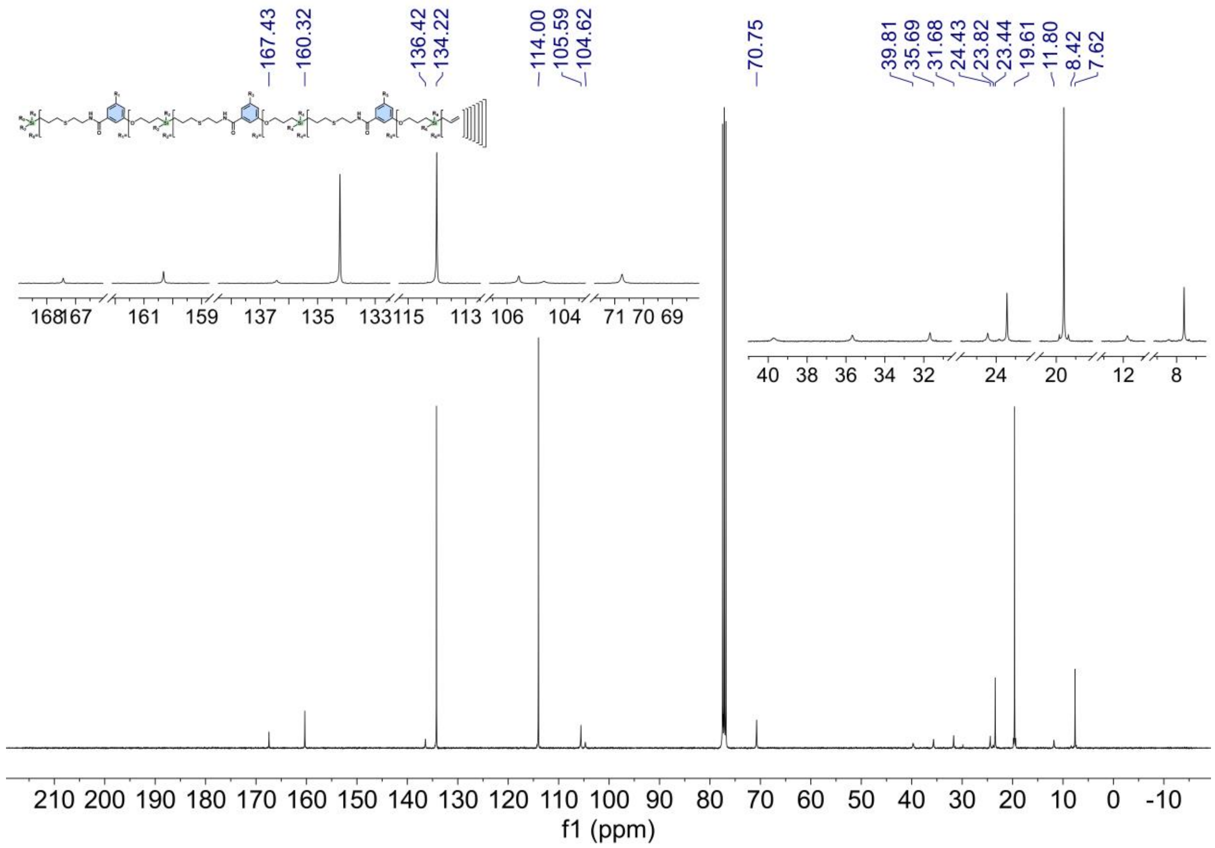

Figure S251: $^{13}C\{^1H\}$ NMR (101 MHz, $CDCl_3$) **G_3-6-6-6-A**

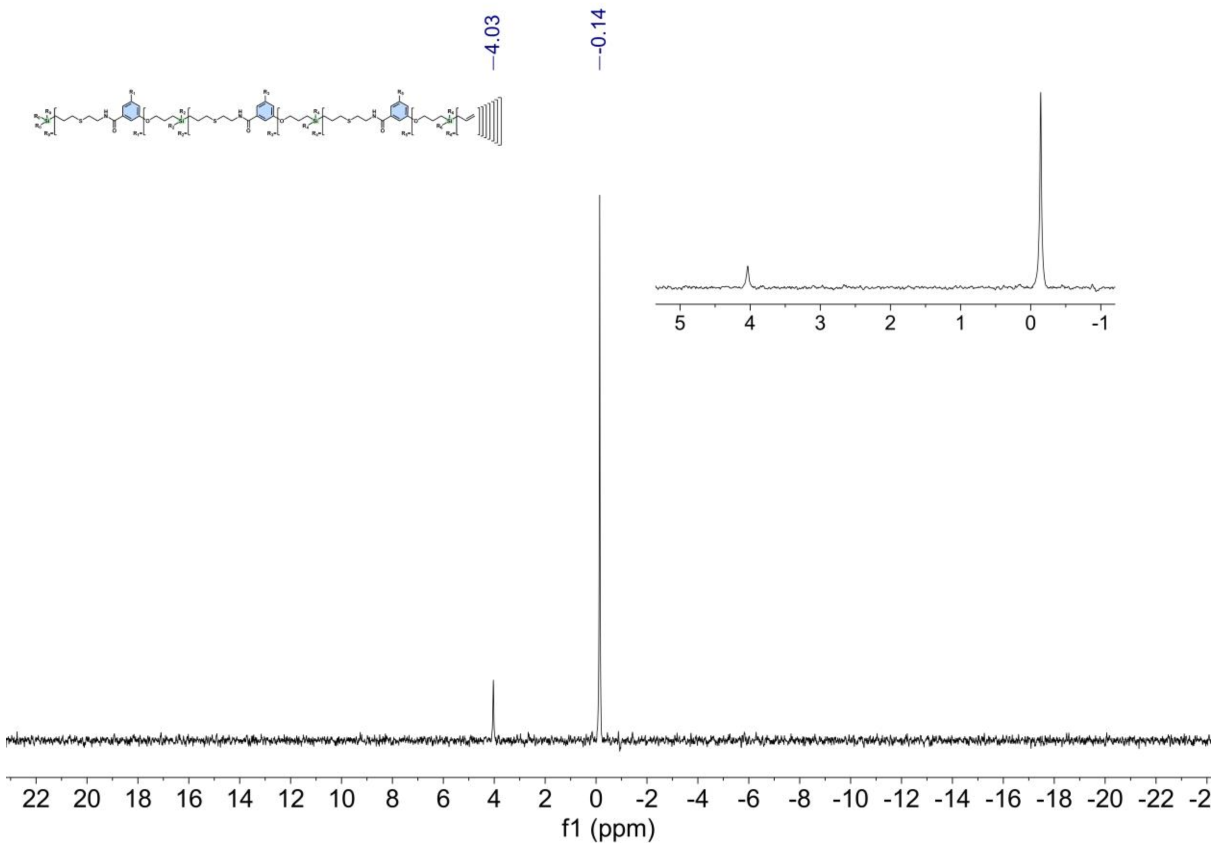

Figure S252: $^{29}Si\{^1H\}$ NMR (79 MHz, $CDCl_3$) **G_3-6-6-6-A**

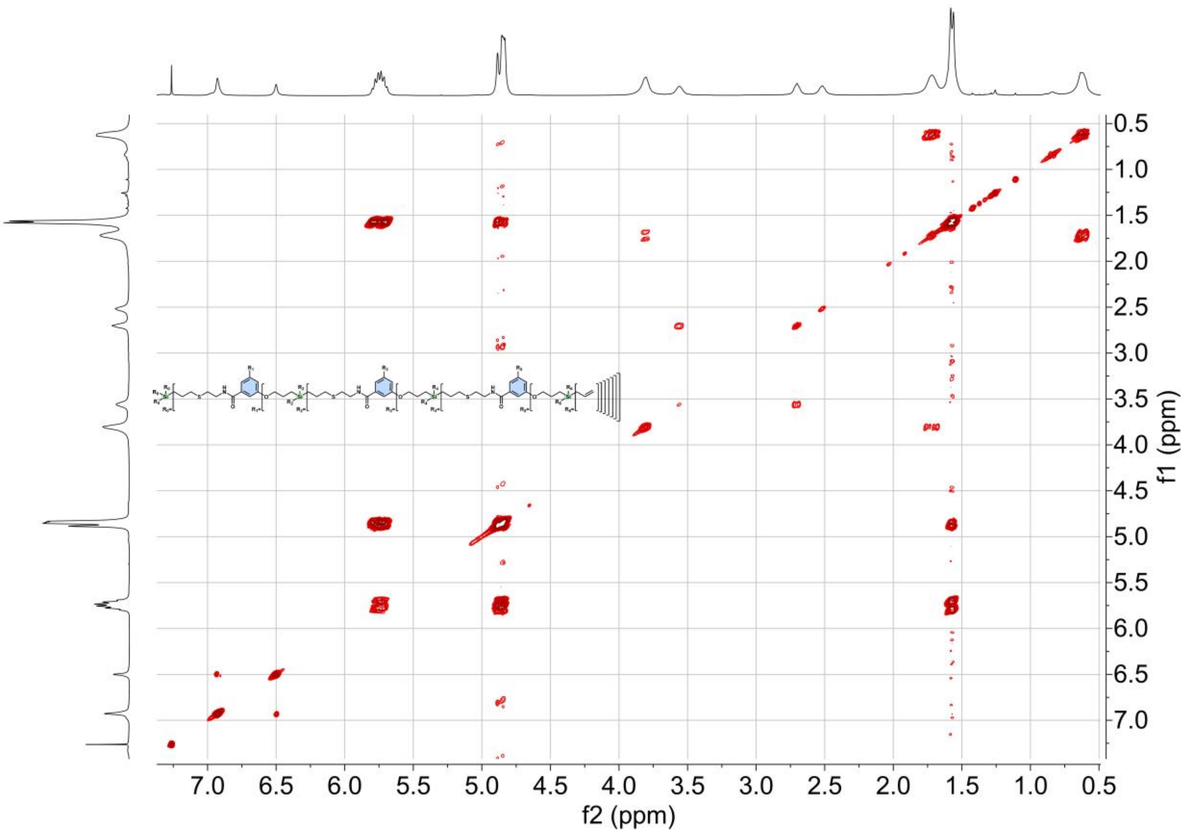

Figure S253: ^{1}H-^{1}H COSY NMR ($CDCl_3$) **G_3-6-6-6-A**

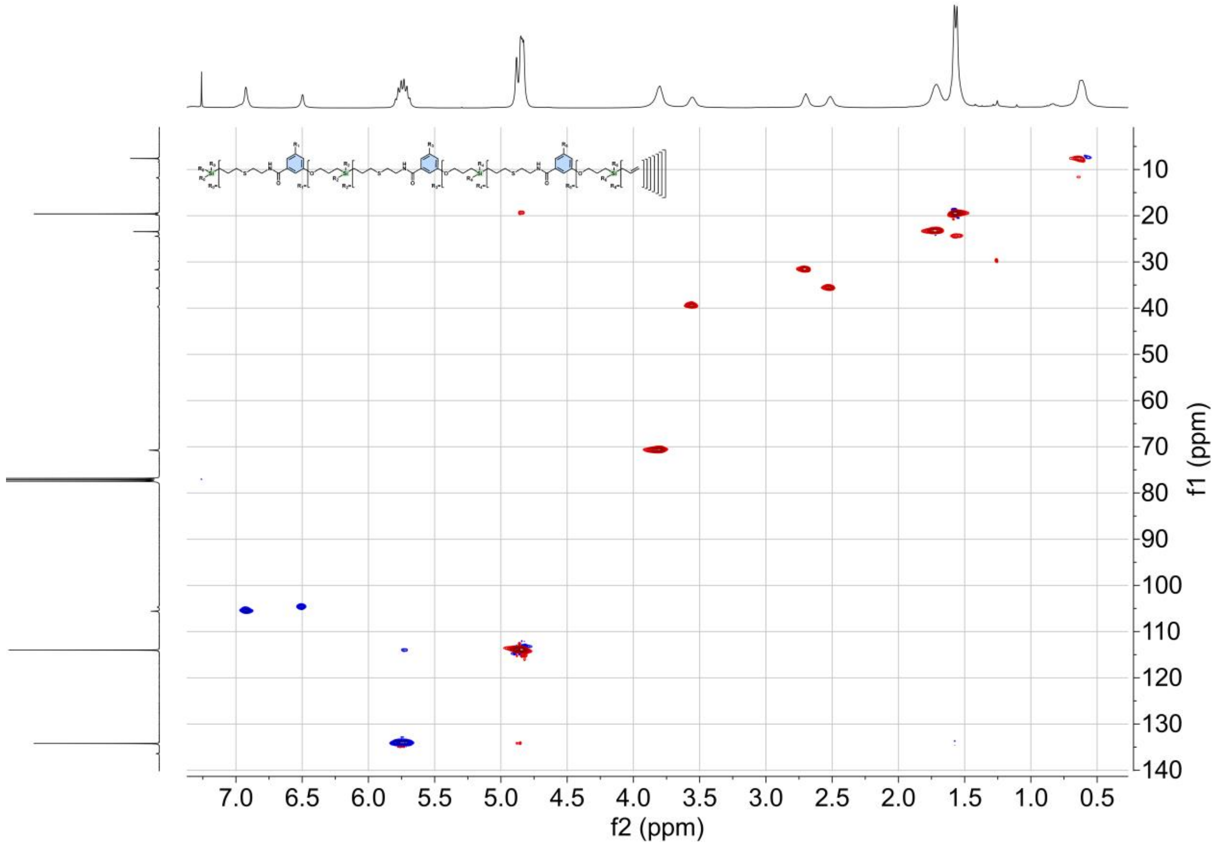

Figure S254: ^{1}H-^{13}C HSQC NMR ($CDCl_3$) **G_3-6-6-6-A**

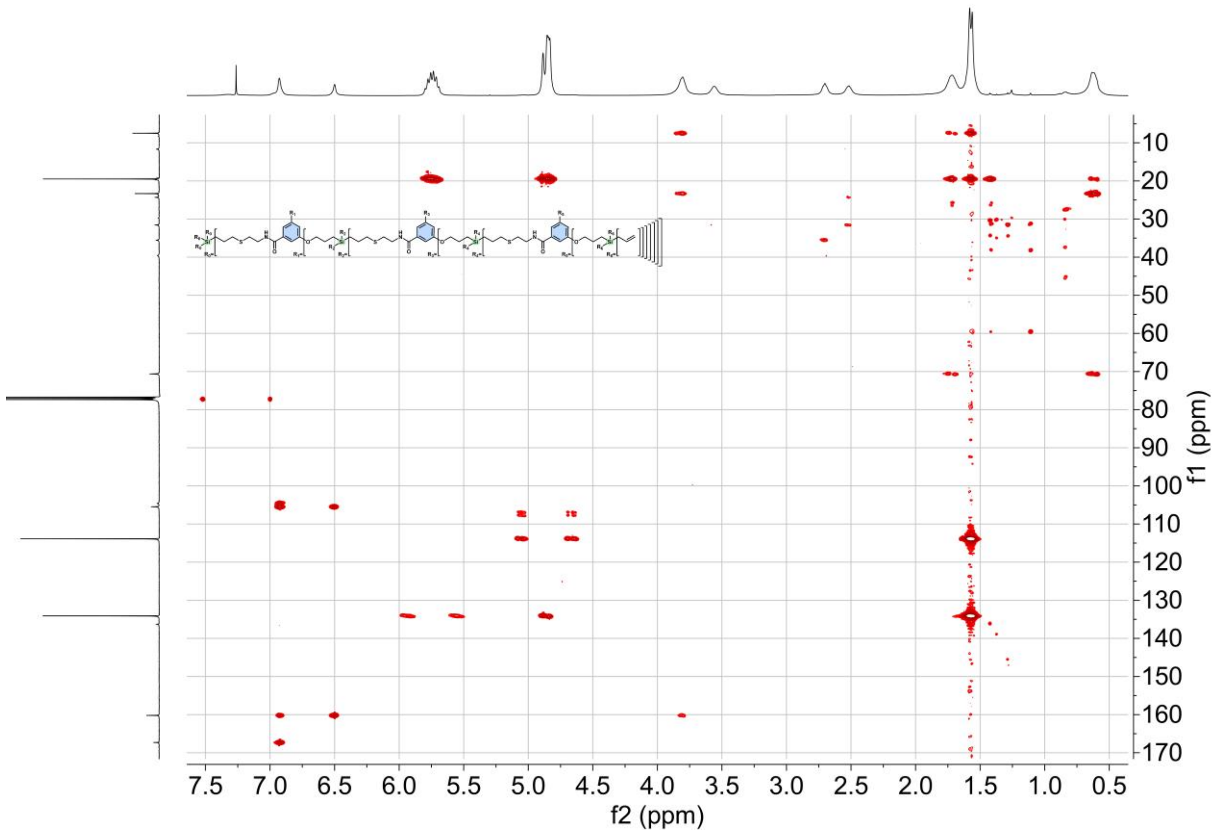

Figure S255 ^{1}H-^{13}C HMBC NMR ($CDCl_3$) **G_3-6-6-6-A**

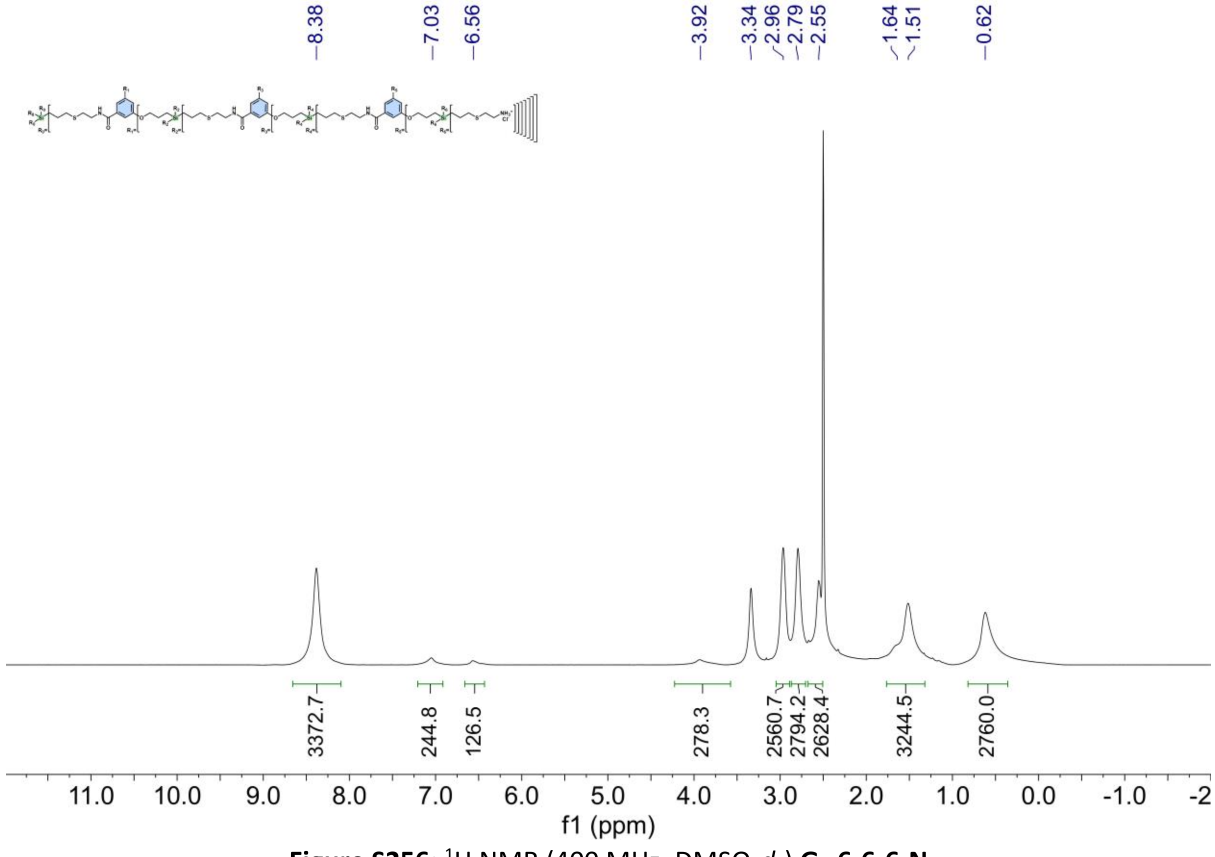

Figure S256: ^{1}H NMR (400 MHz, DMSO-d_6) **G_3-6-6-6-N**

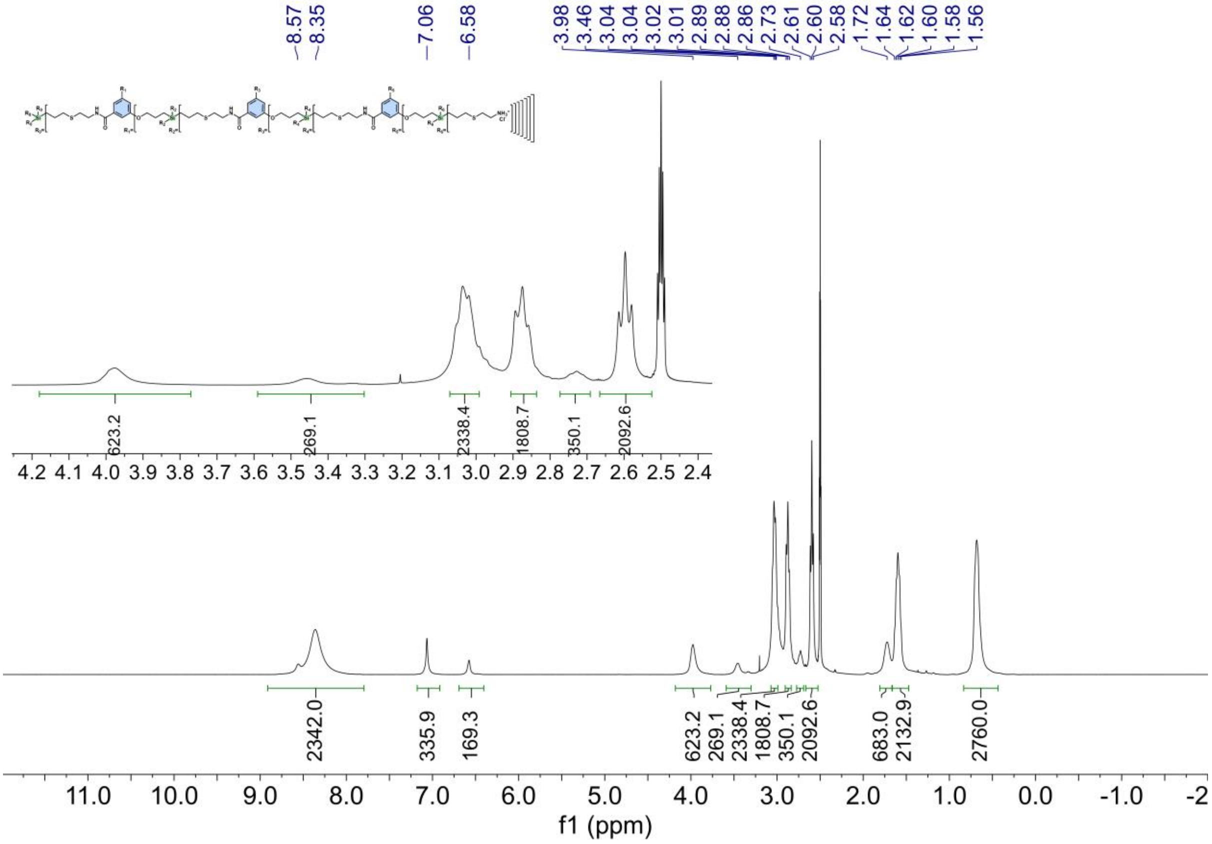

Figure S257: ^{1}H NMR (400 MHz, 100°C, DMSO-d_6) **G_3-6-6-6-N**

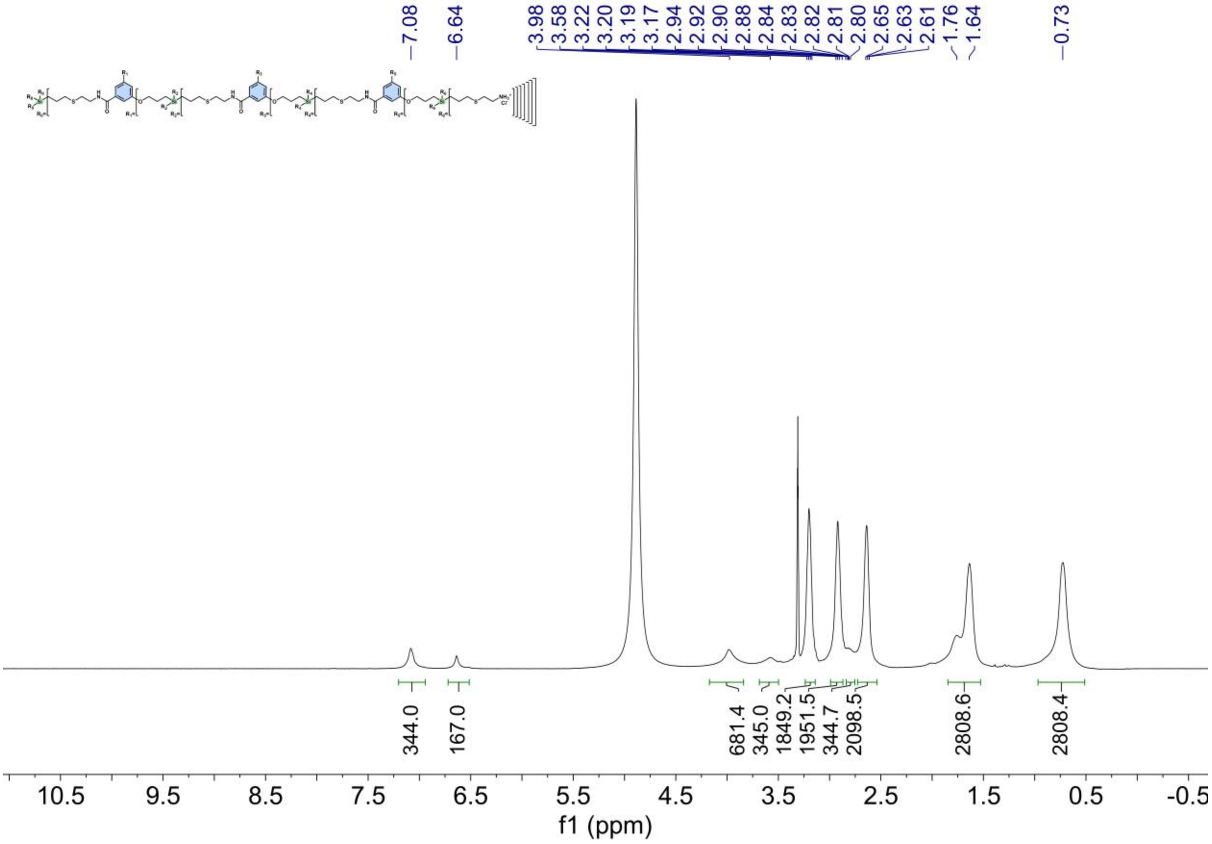

Figure S258: ^{1}H NMR (400 MHz, CD_3OD) **G_3-6-6-6-N**

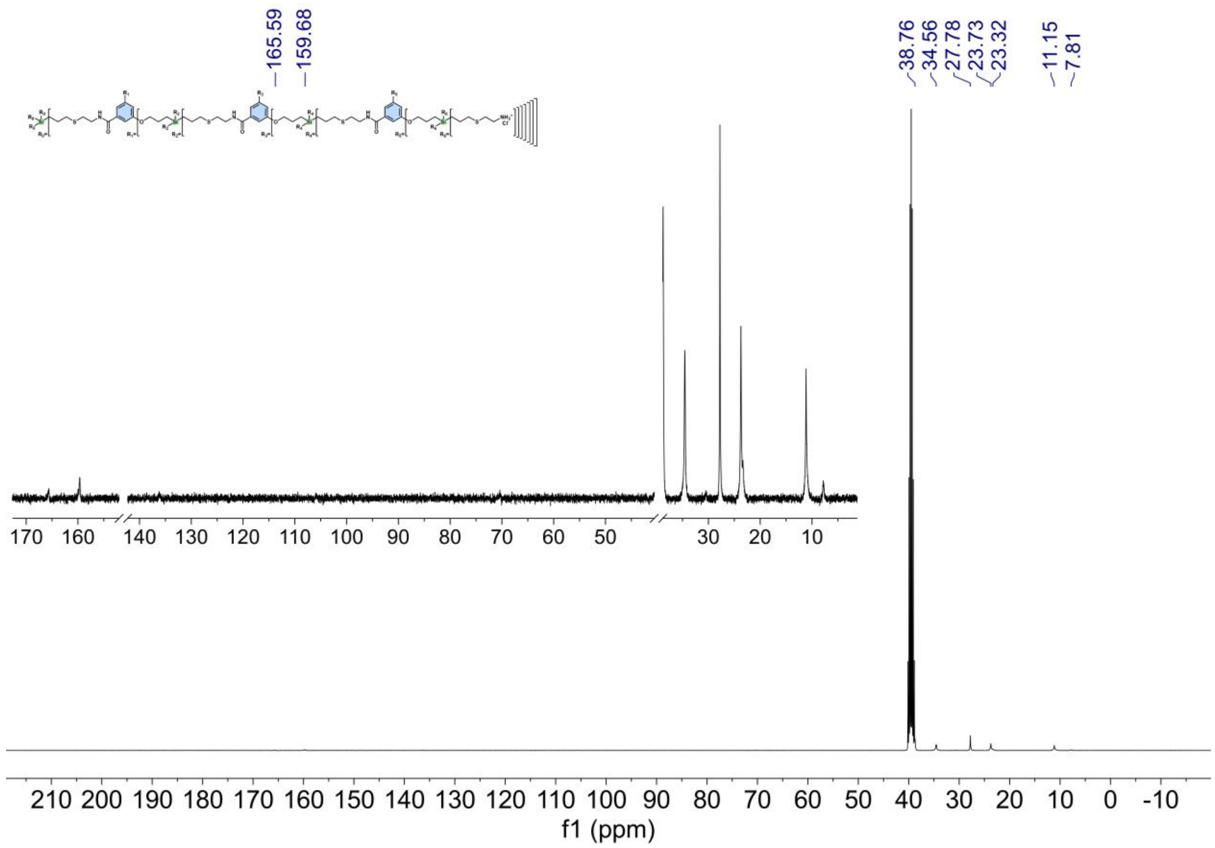

Figure S259: ^{13}C {^{1}H} NMR (101 MHz, DMSO-d_6) **G_3-6-6-6-N**

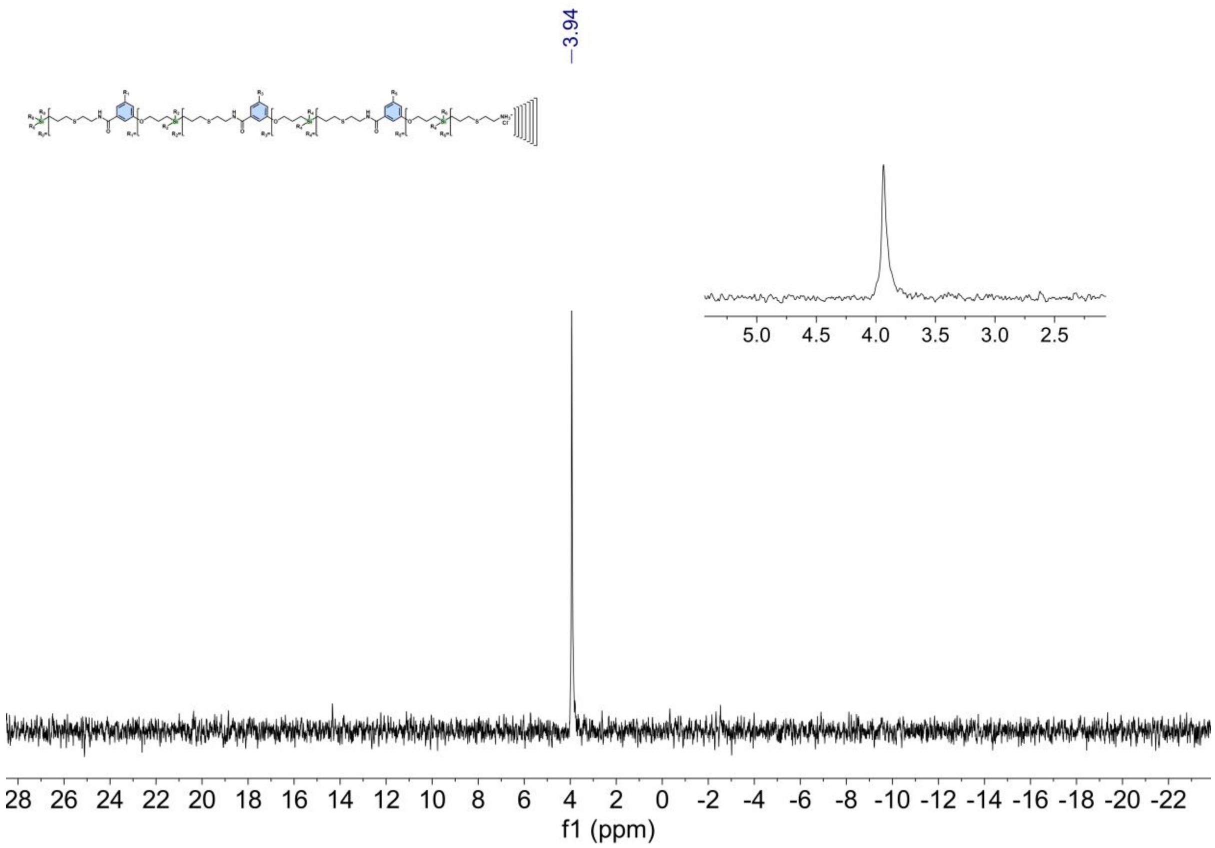

Figure S260: ^{29}Si {^{1}H} NMR (79 MHz, CD_3OD) **G_3-6-6-6-N**

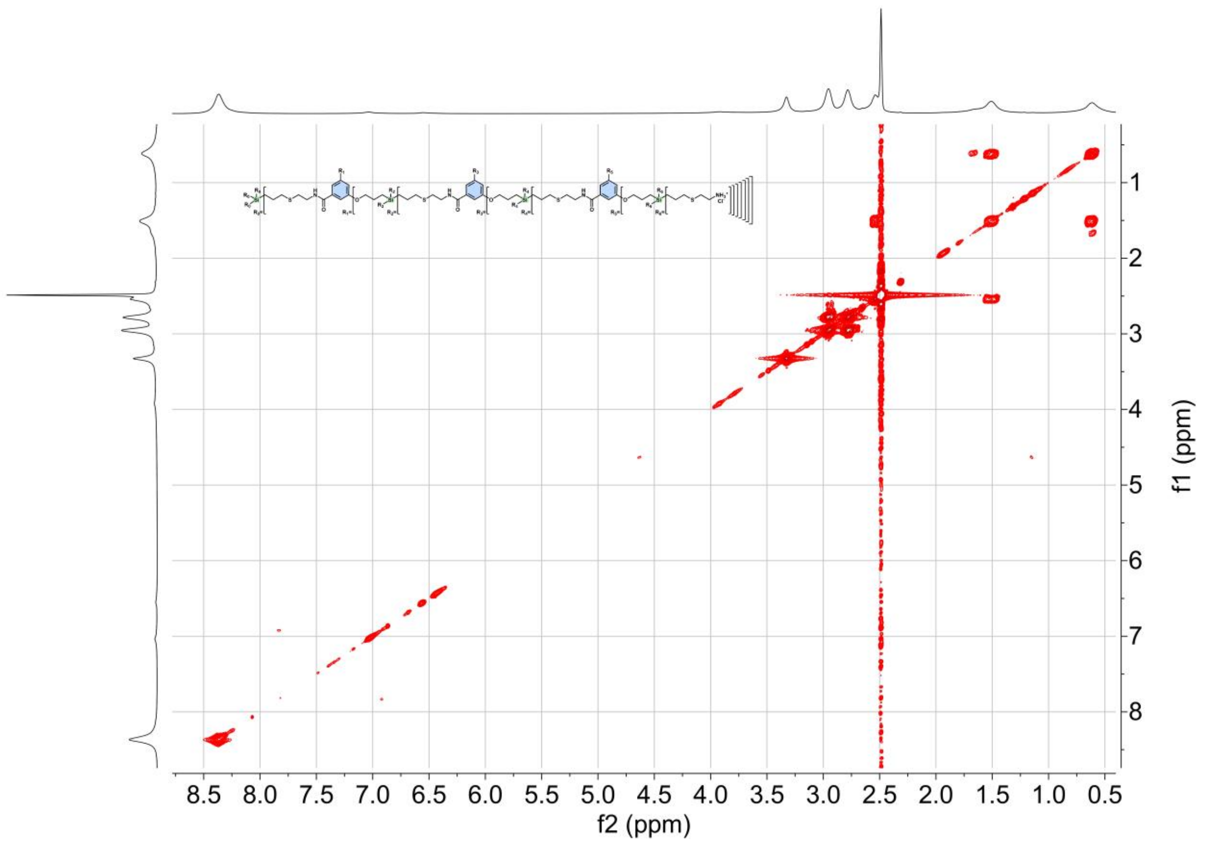

Figure S261: ^{1}H-^{1}H COSY NMR (DMSO-d_6) **G_3-6-6-6-N**

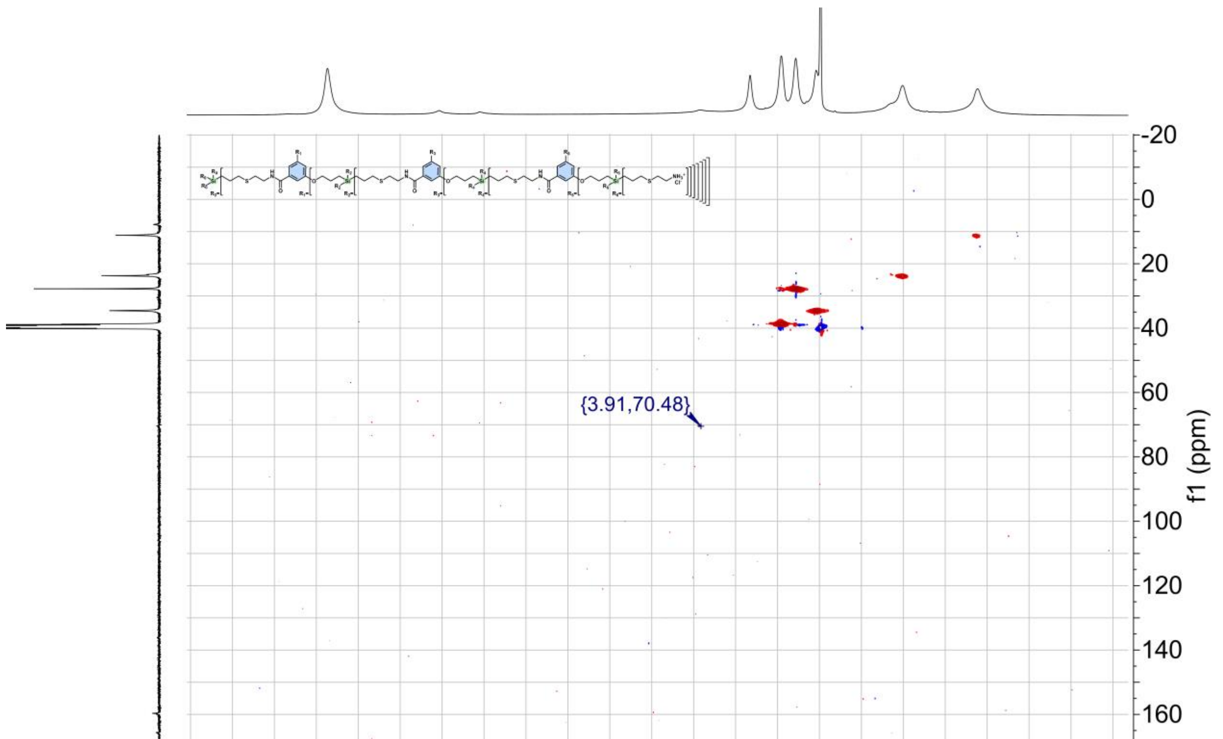

Figure S262: ^{1}H-^{13}C HSQC NMR (DMSO-d_6) **G_3-6-6-6-N**

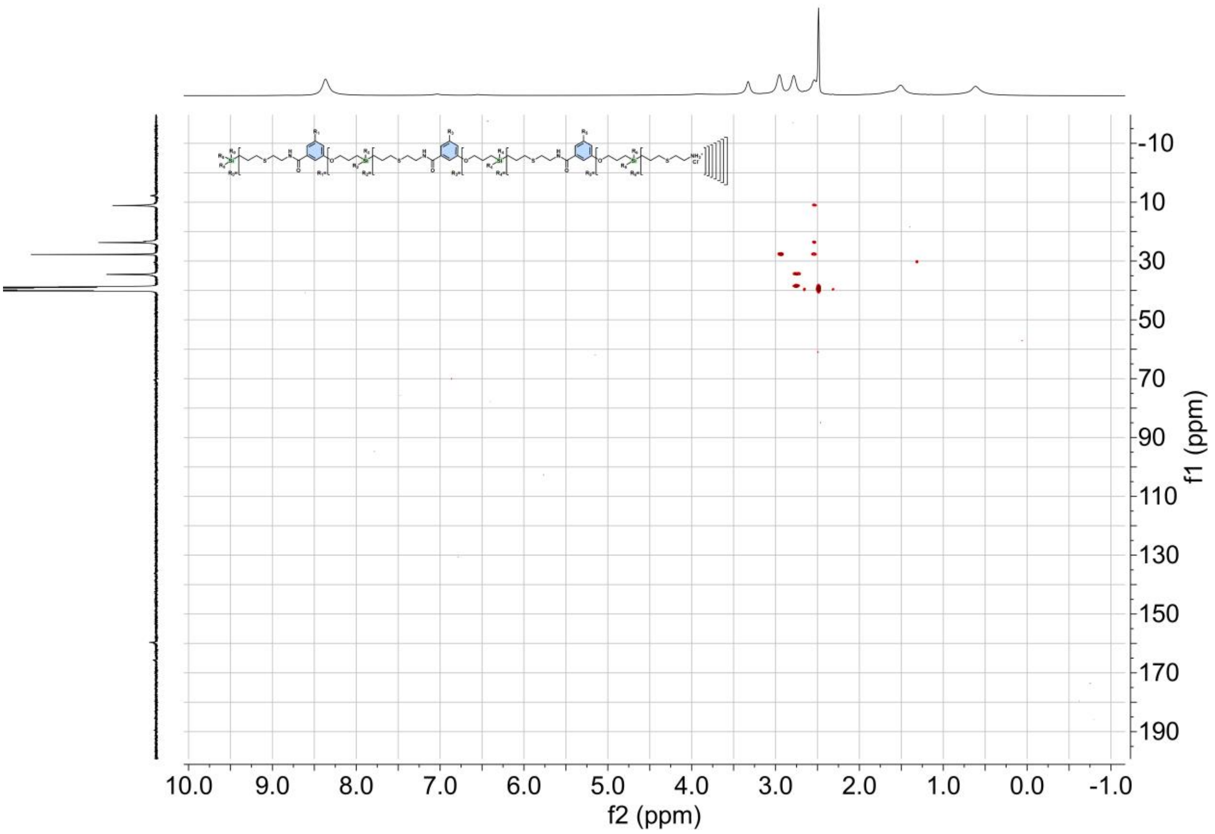

Figure S263: ^{1}H-^{13}C HMBC NMR (DMSO-d_6) **G_3-6-6-6-N**
